# An alkyl-swap platform for late-stage modification of secondary *N*-methylamines

**DOI:** 10.1038/s41557-026-02178-7

**Published:** 2026-06-15

**Authors:** Uroš Vezonik, Giulia Iannelli, Daniel Kaiser, Nuno Maulide

**Affiliations:** Institute of Organic Chemistry, University of Vienna, Währinger Straße 38, 1090 Vienna, Austria

**Keywords:** Synthetic chemistry methodology, Synthetic chemistry methodology

## Abstract

The selective functionalization of unprotected amines with simple, unactivated alkenes and alkynes remains a challenge in contemporary synthesis. Here we present a strategy for the peripheral modification of secondary *N*-methylamines that complements state-of-the-art approaches and tolerates a wide range of functional groups and native amine substrates. This method is primed for application in late-stage functionalization contexts, including the direct bioconjugation of fully functionalized, unprotected building blocks and complex peptides. The retrosynthetic flexibility unlocked by this method also enables the preparation of several commercial, high-grossing pharmaceuticals in a single synthetic step.

**Figure:**
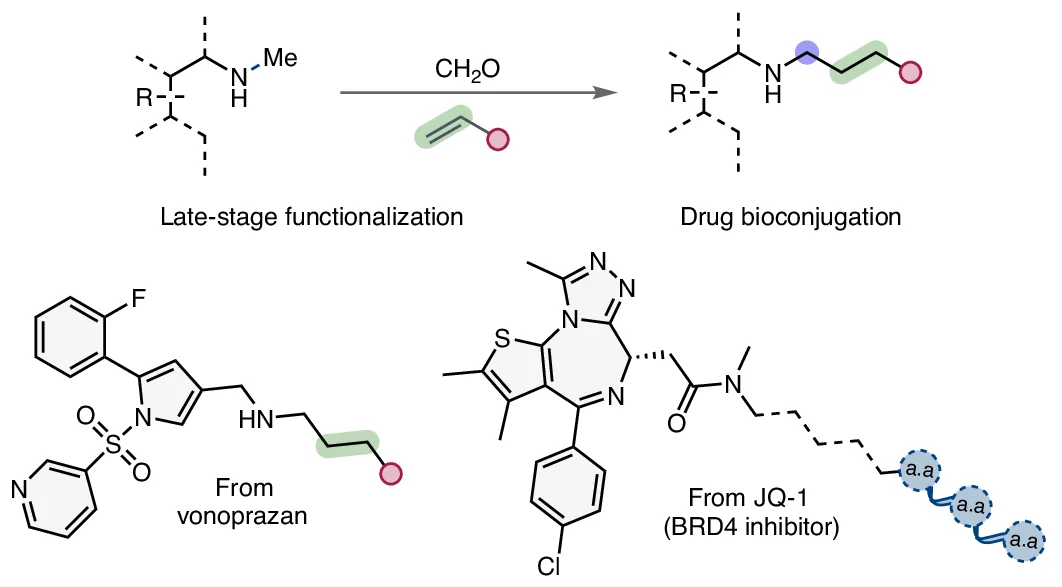

## Abstract (web summary)

The selective functionalization of unprotected amines with alkenes remains a challenge in contemporary synthesis. Now a strategy has been developed for an alkyl swap of *N*-methylamines, enabling late-stage bioconjugation and peptide modification. The resulting retrosynthetic disconnection also streamlines the preparation of several pharmaceuticals and medicinally relevant compounds.

## Main

**Fig. 1:**
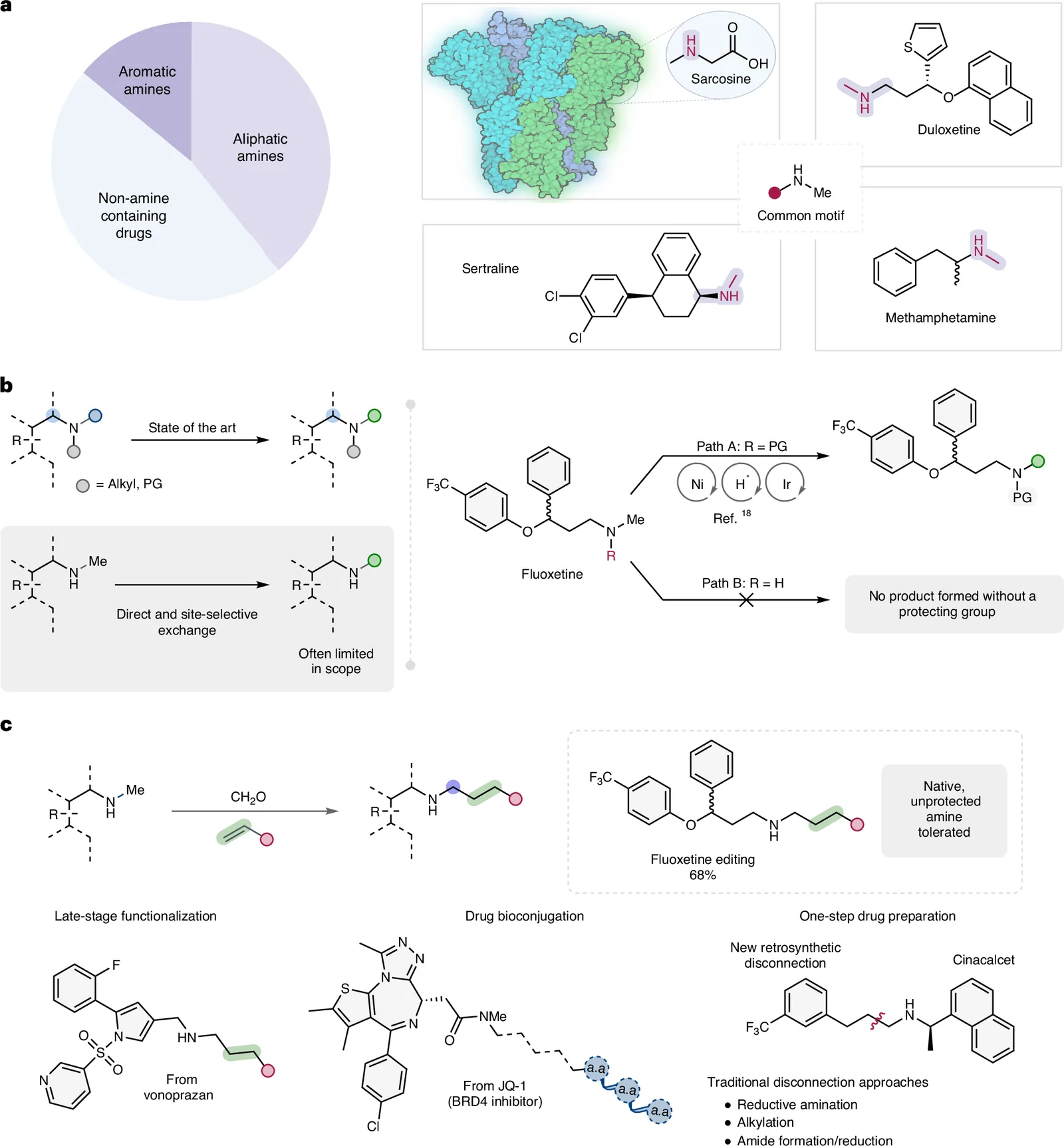
Widespread presence of alkylamines highlights the importance of their structural diversification. **a**, Selected examples of pharmaceuticals containing *N*-methylamines. **b**, Peripheral modification of amines. **c**, This work: peripheral modification of secondary *N*-methylamines with feedstock olefins by alkyl swap. PG, protecting group; R, substituent. Green shading indicates the alkene and the former position of the alkene in the product. Illustration in **a** created in BioRender; Iannelli, G. https://biorender.com/m1lacw4 (2026).

Alkylamines are ubiquitous in pharmaceutically and medicinally relevant compounds (Fig. 1a), where they often serve as key pharmacophores or modulate critical properties such as solubility, basicity and target engagement. Their central role in drug design has driven the development of medicinal chemistry strategies focused on fine-tuning alkylamine motifs to optimize biological activity and absorption, distribution, metabolism and excretion (ADME) profiles^1,2^.

Secondary alkylamines, being more membrane permeable and metabolically stable than primary amines, have found widespread use in a variety of biological settings^3^. This prevalence of biologically active compounds (including several high-grossing pharmaceuticals) containing secondary alkylamines invites late-stage functionalization as a means for rapid structural diversification. Consequently, peripheral functionalization of complex amines has emerged as a cornerstone of modern medicinal chemistry.

Despite this progress, the direct, site-selective replacement of alkyl groups at a late stage remains largely underexplored, highlighting a substantial unmet need in the molecular editing toolkit^4^. The elegant use of transition-metal catalysis, often in combination with directing group strategies, has enabled selective C(*sp*^3^)–H functionalization of amines at the β-, γ- and δ-positions^5–14^, as well as at more remote sites^15,16^. However, methods for achieving selective functionalization at the α-position remain limited to specific substrate classes and are restricted to tertiary, cyclic or protected amines (Fig. 1b)^17–34^.

In particular, selective functionalization at the methyl position of R-NHMe amines (especially yielding linear products) remains challenging. This is largely due to the intrinsic reactivity of the free NH group^35^, which is often incompatible with state-of-the-art approaches relying on photo- and metallaphoto-redox catalysis (Fig. 1b)^18,19,23–26,29–34^. A notable exception to this is a recent approach from Polyzos and co-workers, which relies on the photocatalytic transformation of activated olefins into alkyl carbanions that are subsequently coupled with iminium ions generated photocatalytically from amines^27^. Additionally, α-C–H functionalization of amines can also be achieved using early transition-metal catalysis, although such methods almost exclusively favour the formation of branched products and exhibit limited functional-group tolerance^29–34^.

Furthermore, functionalization at the α-position of alkylamines presents several inherent challenges, in part owing to the abundance of chemically similar C–H bonds, such as those in methyl, methylene and methine groups, adjacent to nitrogen, which makes achieving high regioselectivity particularly difficult.

In a seminal study, MacMillan and co-workers demonstrated α-C(*sp*^3^)–H functionalization of the NMe moiety of fluoxetine (Prozac) through photoredox catalysis in a late-stage context. The transformation strictly required a protecting group on the amine (Fig. 1b)^18^, as unprotected amines were found to not allow product formation (see Supplementary Section 7 for details). This illustrates a broader limitation: the difficulty in directly applying C(*sp*^3^)–H functionalization to unbiased, pharmaceutically relevant alkylamines without prior derivatization. We note that a singular exception is Schafer and colleagues’ functionalization of fluoxetine, which proceeded with selectivity for the branched product^33^. Owing to this dearth of methods allowing peripheral functionalization of secondary methylamines with linear selectivity, we envisioned an unconventional approach wherein the treatment of such a substrate with a formaldehyde equivalent and an unbiased, unactivated alkene would facilitate an alkyl swap. From a retrosynthetic logic, this can be formally seen as replacing a C–H bond of the methyl group with an alkyl moiety of choice (Fig. 1c).

Here we show how this approach circumvents the reactivity constraints of acidity and bond-dissociation energy by leveraging kinetic control in a hydricity-dependent process that proceeds with complete linear selectivity^18^. This development builds upon our previous work on redox-neutral transformations of alkenes, including hydroaminomethylation^36–39^. While this provided good functional group tolerance, it could not be applied to complex, polyfunctionalized and acid-sensitive reactants. This was primarily a result of the employed reaction conditions, which saw a requirement for the use of a large excess of both a strong Brønsted acid and aminals as iminium ion precursors, precluding the functionalization of valuable amine substrates.

We now advance this concept by employing complex secondary *N*-methylamines as core scaffolds and alkenes as coupling partners to unlock a platform in peripheral functionalization, thus paving the way for several examples of late-stage functionalization and direct drug bioconjugation. Moreover, we demonstrate how this transformation allows the expedient, one-step synthesis of several commercial blockbuster pharmaceuticals by opening a retrosynthetic disconnection along the aliphatic chain of an amine.

## Results and discussion

**Fig. 2:**
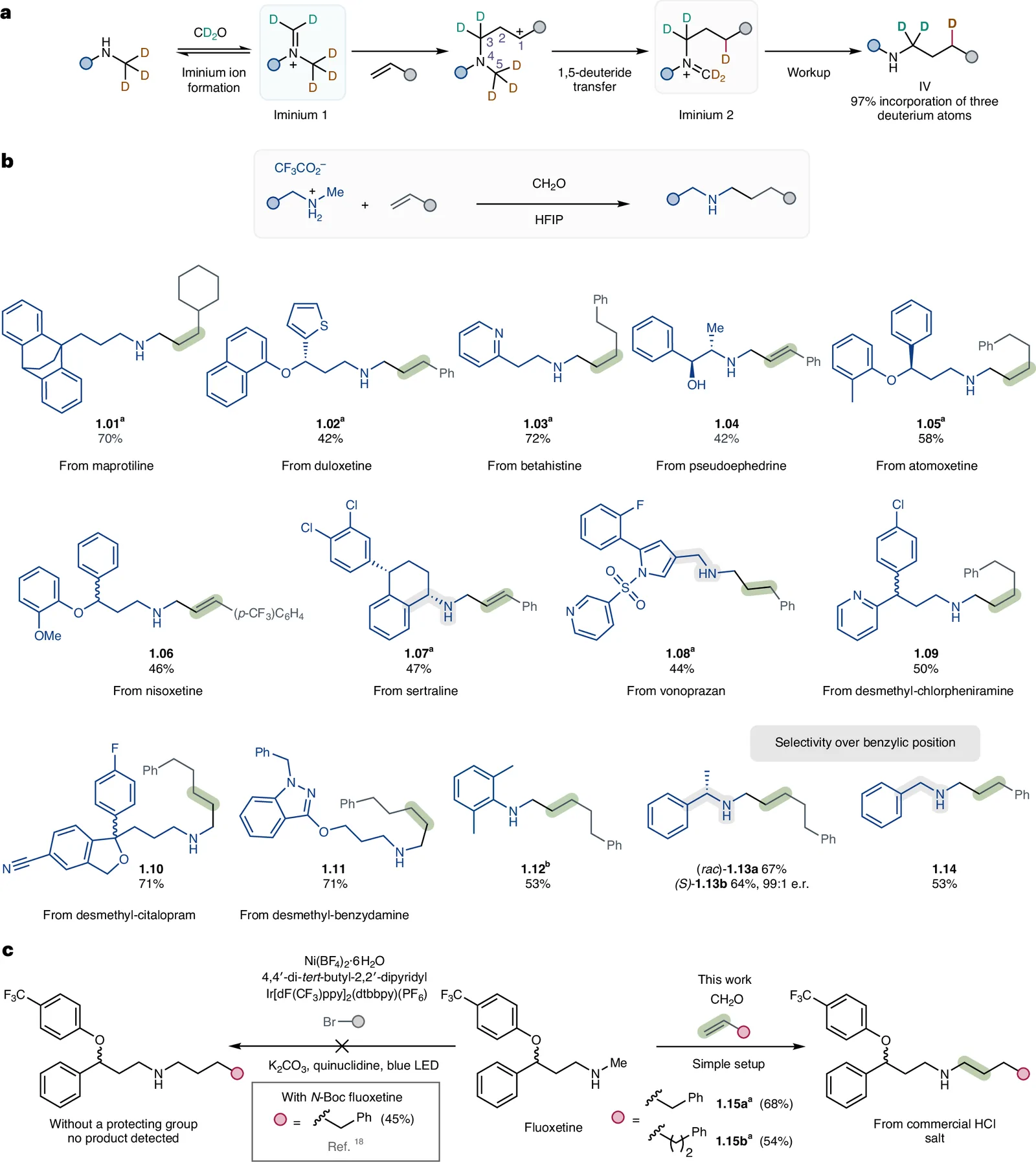
Mechanistic proposal and synthetic utility of the method. **a**, Mechanistic proposal supported by NMR and deuterium labelling. **b**, Late-stage functionalization of amines. **c**, Comparison with the state-of-the-art method. General reaction conditions: 1.0 equiv. amine salt, 1.1 equiv. alkene, 1.0 equiv. CH_2_O, HFIP (1.2 M), 75 °C, 16 h. ^a^Amine was used as a commercially available hydrochloride/fumarate salt. ^b^Reaction performed using 3.0 equiv. alkene and at 25 °C. Enantiomeric ratio (e.r.) determined using chiral high-performance liquid chromatography (HPLC). Boc, *tert-*butyloxycarbonyl; dtbbpy, 4,4′-di-*tert*-butyl-2,2′-bipyridine; HFIP, hexafluoroisopropanol; LED, light-emitting diode; (*p*-CF_3_)C_6_H_4_, *para*-(trifluoromethyl)phenyl; ppy, 2-phenylpyridine; *rac*, racemic. Green shading indicates the alkene and the former position of the alkene in the product. Grey shading indicates the presence of benzylic positions as potentially competing moieties^18^.

A schematic representation of the mechanistic pathway consistent with our experimental observations is shown in Fig. 2a. Here, nucleophilic addition of an olefin to an in situ-generated iminium ion (Iminium 1) initiates an internal redox process through a 1,5-hydride or 1,5-deuteride transfer, forming a second intermediate iminium ion (Iminium 2), which yields the desired secondary amine upon work-up. The reaction mechanism was investigated through a series of isotopic labelling experiments and NMR studies (see Supplementary Section 6 for details on these experiments). Use of both deuterated formaldehyde and an amine perdeuterated on the methyl group led to the formation of trideuterated product **IV** with 97% incorporation of deuterium in the expected positions. This provided clear evidence of the role of formaldehyde as a methylene donor. Additionally, the incorporation of one deuterium atom at the β-position of the former alkene unequivocally supports an intramolecular hydride/deuteride transfer as the formal reduction event.

The reaction proceeded efficiently when the coupling partners, the amine salt and alkene substrates, were combined with paraformaldehyde in hexafluoroisopropanol (HFIP) in almost equal amounts at 75 °C. A primary objective was to apply this alkyl-swap protocol, selectively transforming a secondary methylamine into the corresponding alkylamino moiety of choice, in a late-stage context to complex structures commonly found in pharmaceutical candidates and other biologically relevant molecules (Fig. 2b). To achieve this, we tested several amine-containing drugs under our standard reaction conditions. Remarkably, the reaction successfully produced amines **1.01–1.11**, resulting from direct functionalization of maprotiline, duloxetine, betahistine, pseudoephedrine, atomoxetine, nisoxetine, sertraline, vonoprazan and desmethylated chlorpheniramine, citalopram and benzydamine, respectively, in synthetically useful yields. Moreover, our survey of different aliphatic amines (see Supplementary Section 4 for additional scope entries) showed excellent to good yields, with several functional groups in proximity to the reactive center being well tolerated. The reaction is not inherently limited to the use of alkylamines, as shown by the formation of product **1.12**. However, competing Povarov reaction precludes the use of anilines lacking *ortho* substitution. By leveraging kinetic control of the internal redox process (see Supplementary Section 6 and Supplementary Fig. 6 for a detailed mechanistic proposal), the reaction design allows for selective functionalization at the desired methyl position, even for benzylamine derivatives (**1.07**, **1.08**, **1.13**, **1.14**). This important realization positions our method as one of only a few transformations that do not show a preference for reaction at the benzylic position^34^.

A comparison of our method with a previously reported pioneering approach for α-C–H alkylation of amines^18^, relying on protected substrates, is shown in Fig. 2c. When a fully unprotected amine, the antidepressant fluoxetine, was subjected to established C–H functionalization methods involving a combination of photoredox, nickel and hydrogen-atom-transfer catalysis, no C(*sp*^3^)–H alkylation products were obtained (confirming the reported need for Boc protection; see Supplementary Section 7 for details on these reactions). In stark contrast, the method presented here efficiently delivered the functionalized secondary amines **1.15a,b** in good yields.

**Fig. 3:**
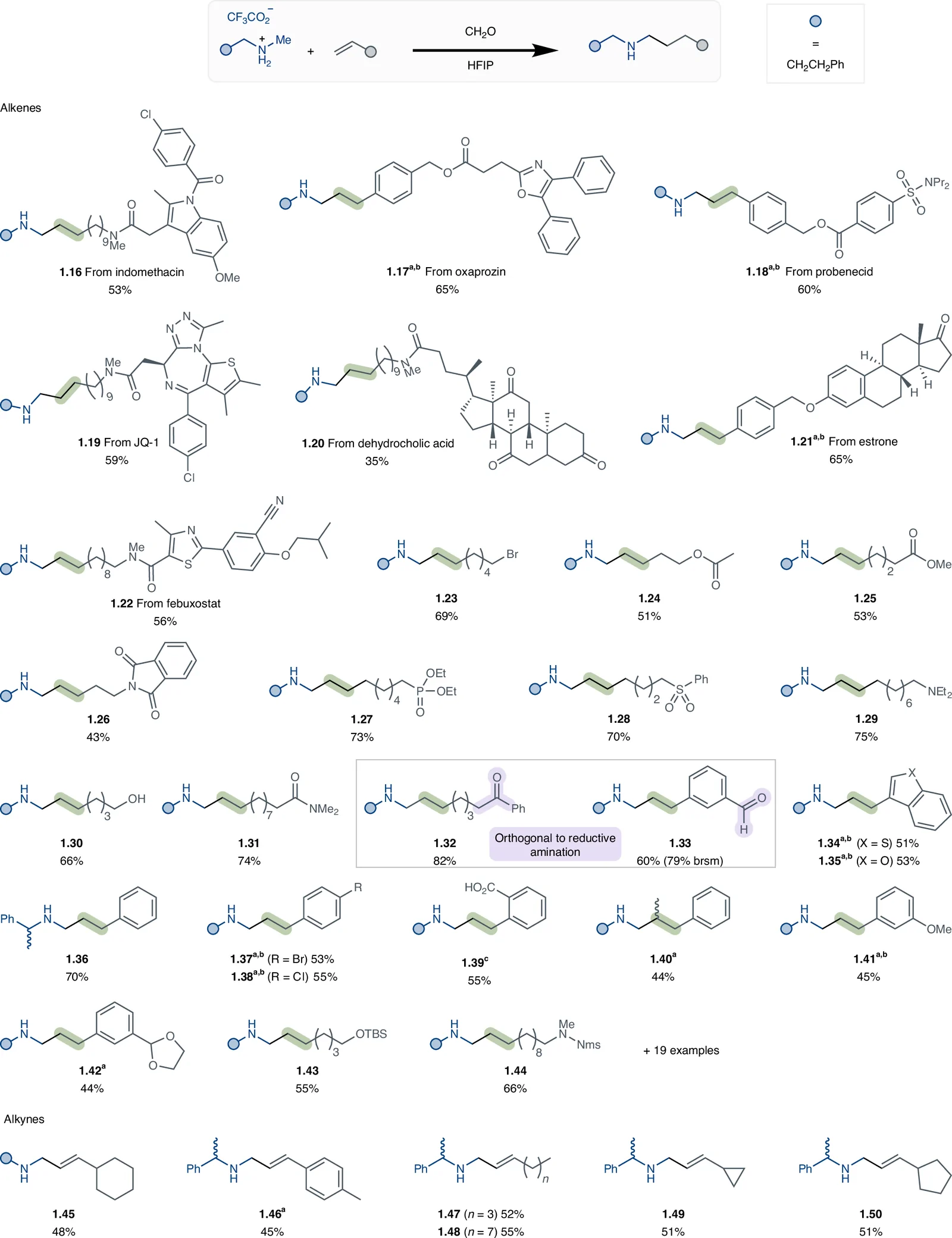
Late-stage functionalization of amines with complex alkenes and the reaction scope. General reaction conditions: 1.0 equiv. amine salt, 1.1 equiv. alkene, 1.0 equiv. CH_2_O, HFIP (1.2 M), 75 °C, 16 h. ^a^Reaction performed at 50 °C. ^b^Reaction performed using 1.5 equiv. of in situ-prepared iminium salt. ^c^The product was transformed into the *tert*-butyl carbamate (Boc) prior to isolation. brsm, based on recovered starting material; HFIP, hexafluoroisopropanol; OEt, ethoxy; OMe, methoxy; Pr, propyl. Green shading indicates the former position of the alkene in the product. Purple shading indicates the presence of carbonyls that would not be compatible with reductive amination protocols.

Encouraged by these results we next investigated the applicability of complex alkenes in the selective functionalization of amines. We proceeded to prepare various pharmaceuticals functionalized with an aliphatic or aromatic linker bearing a terminal alkene to serve as a handle for our reaction (Fig. 3). Pleasingly, applying our reaction protocol to the thus-obtained substrates yielded the expected compounds in generally good yields (**1.16–1.22**), demonstrating both the potential of this transformation to allow preparation of complex alkylamine architectures as well as its robustness in handling diverse functional groups. Indeed, a wide range of potentially reactive moieties, regardless of electronic or steric properties, were found to be well tolerated, as substrates bearing halide, acetate, ester, phthalimide, phosphonate, sulfone, amine, alcohol or amide moieties largely provided the desired products in high yields (**1.23–1.31**). We note that 1-phenylhept-6-en-1-one and 4-vinylbenzaldehyde (**1.32**, **1.33**), potentially challenging substrates due to the presence of ketone and aldehyde groups, similarly to dehydrocholic acid and estrone derivatives (**1.20**, **1.21**), exclusively afforded the desired products, thus showing the complementarity of this approach to amine synthesis through reductive amination. Furthermore, various vinyl-substituted (hetero)arenes were investigated (**1.34–1.42**), showing excellent tolerance of electron-neutral, electron-donating and electron-depleting substituents. Remarkably, the reaction also exhibited broad protecting-group tolerance, with a *tert*-butyldimethylsilyl (TBS) ether (**1.43**) and a nonafluoromesitylenesulfonyl (Nms)-protected amine (**1.44**)^40^, as well as an acetal (**1.42**) being preserved. Additionally, despite a slight decrease in yield, we were pleased to find that terminal alkynes produced secondary allylic amines (**1.45–1.50**, Fig. 3, bottom), exclusively as the (*E*)-configured stereoisomers.

**Fig. 4:**
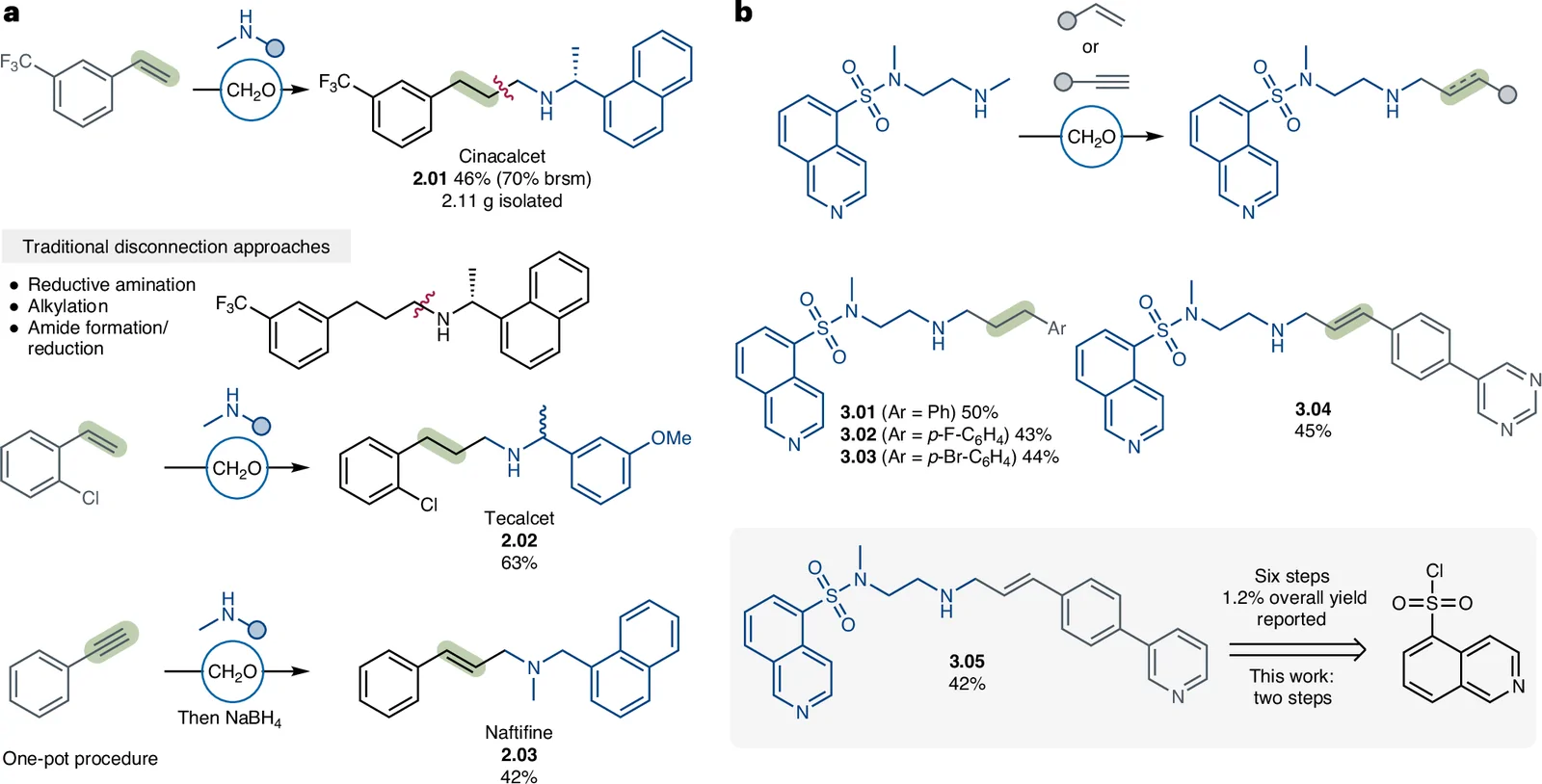
Expedient synthesis of pharmaceuticals and medicinally relevant compounds. **a**, One-step preparation of pharmaceuticals and a one-pot synthesis of naftifine. **b**, Expedient preparation of protein kinase A (PKA) and Fms-like tyrosine kinase 3 (FLT3) inhibitors. Ph, phenyl; *p*-F-C_6_H_4_, 4-fluorophenyl; *p*-Br-C_6_H_4_, 4-bromophenyl. Green shading indicates the alkene/alkyne and the former position of the alkene/alkyne in the product.

To further demonstrate the broad applicability of this retrosynthetic logic, we deployed the method for the expedient preparation of a range of marketed drugs directly from simple and readily available olefins and methylamines (Fig. 4a). Utilizing our disconnection, cinacalcet (**2.01**) and tecalcet (**2.02**) were furnished in good yields in a true single-step fashion, with the example of cinacalcet (**2.01**) also showing this process to be amenable to gram scale. By leveraging the reaction’s resting state (Iminium 2 in Fig. 2a, see also Supplementary Section 6 and Supplementary Fig. 6), and therefore the possibility to furnish tertiary amines, we were able to prepare the cinnamylamine-bearing antifungal drug naftifine (**2.03**) in good yield employing a reductive quench.

Furthermore, we have shown that this transformation enables the rapid preparation of a library of compounds based on an isoquinoline sulfonamide scaffold that has attracted considerable recent interest as multi-target inhibitors, including protein kinase A (PKA) and Fms-like tyrosine kinase 3 (FLT3) inhibition (**3.01–3.05**) (Fig. 4b)^41–44^. By employing a distinct disconnection strategy based on the use of alkenes and alkynes, the desired products can be obtained in only two steps, providing a notably shorter and more expedient synthetic route than that previously reported (as exemplified by **3.05**)^44^.

**Fig. 5:**
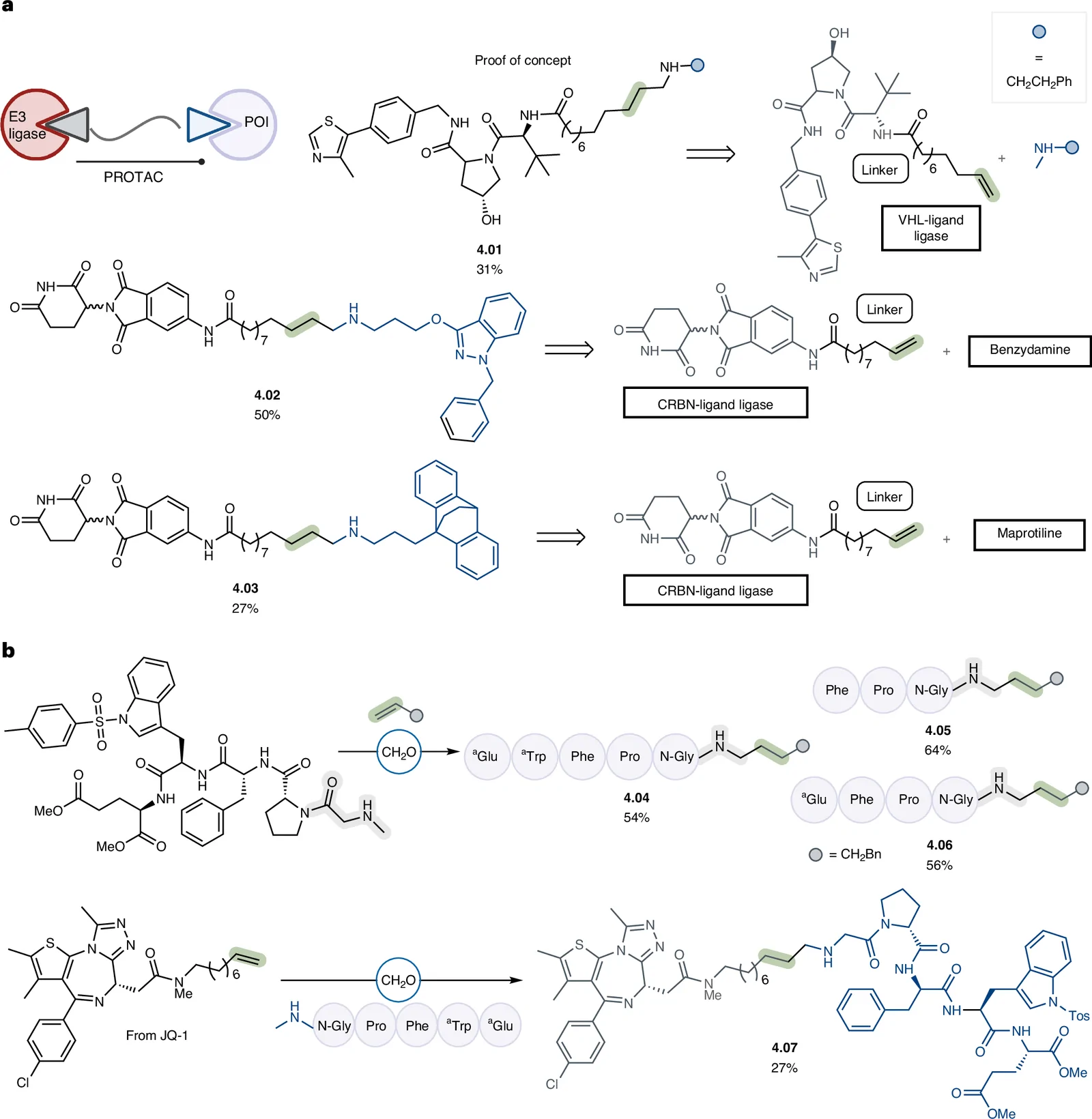
Demonstration of alkyl swap in bioconjugation. **a**, Streamlined preparation of proteolysis-targeting chimeras (PROTACs). **b**, Functionalization of peptides and synthesis of a model peptide–drug conjugate. ^a^C-terminal and/or side-chain protecting group. Bn, benzyl; OMe, methoxy; POI, protein of interest; Tos, toluenesulfonyl; VHL, Von Hippel–Lindau. Illustration in **a** created in BioRender; Iannelli, G. https://biorender.com/04g4ixv (2026). Green shading indicates the alkene and the former position of the alkene in the product.

Given the contemporary relevance of, and synthetic challenges associated with, proteolysis-targeting chimeras (PROTACs), heterobifunctional molecules able to place a given target protein in proximity to ubiquitin-proteasome machinery (UPS) for selective degradation, we sought to apply our methodology to their streamlined preparation (Fig. 5a)^45^. To enhance the suitability of certain substrates, such as ligase E3 ligands (Von Hippel–Lindau (VHL) and cereblon-based), we incorporated a conjugated alkyl linker, an indispensable structural element in PROTAC design, with a terminal alkene. These modified substrates successfully acted as reactive handles, yielding the desired degraders (**4.01**–**4.03**) bearing different (amino-)warheads. Additionally, this approach provides an orthogonal retrosynthetic disconnection compared with traditional amide coupling and/or click chemistry typically used for PROTAC synthesis.

To further showcase the functional group tolerance, we subjected sarcosine-containing peptides to this transformation (Fig. 5b), obtaining the corresponding alkylated products in good yields (**4.04–4.06**). Encouraged by these results, the method was also utilized for the synthesis of a model peptide–drug conjugate (**4.07**)^46^, where a pentapeptide was successfully coupled with the bromodomain-containing protein 4 (BRD4) inhibitor JQ-1.

In summary, we have shown that this method for the functionalization of native *N*-methylamines using simple and abundantly available alkenes and alkynes under mild conditions allows for broad functional group tolerance on a wide variety of substrates, ranging from simple carbon-heavy alkyl chains to elaborate and functionality-laden molecules such as peptides. This transformation represents an alternative retrosynthetic disconnection in unprotected and challenging settings, and its applicability to bioconjugation and late-stage functionalization are hallmarks of a method that holds clear potential for extensive application in synthesis.

## Methods

### Representative general procedure 1 for the synthesis of amines (1–4)

At ambient temperature, the corresponding secondary amine (as a salt, 1.0 equiv.), paraformaldehyde (1.0 equiv.) and hexafluoroisopropanol (1.2 M) were added to an oven-dried vial and this was followed by the addition of the corresponding alkene/alkyne (1.1–1.3 equiv.). The vial was sealed with a cap and the reaction was vigorously stirred at 75 °C for 16 h, after which it was allowed to cool to ambient temperature. After that, aqueous sodium hydroxide (3 M) was added until the pH reached 12. The product was extracted with dichloromethane (3×) and dried over anhydrous magnesium sulfate. The dried solution was filtered and the filtrate was concentrated under reduced pressure to afford the crude material, which was purified by flash column chromatography on silica gel with dichloromethane/DMA-mix (DMA-mix is a solvent mixture of dichloromethane, methanol and an aqueous solution of ammonium hydroxide in the ratio 9:1:0.1) to afford the analytically pure desired product.

## Online content

Any methods, additional references, Nature Portfolio reporting summaries, source data, extended data, supplementary information, acknowledgements, peer review information; details of author contributions and competing interests; and statements of data and code availability are available at https://doi.org/10.1038/s41557-026-02178-7.

## Supplementary information


Supplementary InformationSupplementary Sections 1–9, Figs. 1–11, Table 1, analytical data and references.


## Data Availability

All data are available within the Article and its Supplementary Information.

## References

[1] Vitaku, E., Smith, D. T. & Njardarson, J. T. Analysis of the structural diversity, substitution patterns, and frequency of nitrogen heterocycles among U.S. FDA approved pharmaceuticals. *J. Med. Chem.* **57**, 10257–10274 (2014). 25255204 10.1021/jm501100b

[2] Guillemard, L., Kaplaneris, N., Ackermann, L. & Johansson, M. J. Late-stage C–H functionalization offers new opportunities in drug discovery. *Nat. Rev. Chem.* **5**, 522–545 (2021). 37117588 10.1038/s41570-021-00300-6

[3] Foye, W. O., Lemke, T. L. & Williams, D. A. *Foye’s Principles of Medicinal Chemistry* (Lippincott Williams and Wilkins, 2006).

[4] Ma, C., Lindsley, C. W., Chang, J. & Yu, B. Rational molecular editing: a new paradigm in drug discovery. *J. Med. Chem.* **67**, 11459–11466 (2024). 38905482 10.1021/acs.jmedchem.4c01347

[5] Willcox, D. et al. A general catalytic β-C–H carbonylation of aliphatic amines to β-lactams. *Science* **354**, 851–857 (2016). 27856900 10.1126/science.aaf9621

[6] Cabrera-Pardo, J. R., Trowbridge, A., Nappi, M., Ozaki, K. & Gaunt, M. J. Selective palladium(II)-catalyzed carbonylation of methylene β-C–H bonds in aliphatic amines. *Angew. Chem. Int. Ed.* **56**, 11958–11962 (2017). 10.1002/anie.201706303 28707312

[7] McNally, A., Haffemayer, B., Collins, B. S. L. & Gaunt, M. J. Palladium-catalysed C–H activation of aliphatic amines to give strained nitrogen heterocycles. *Nature* **510**, 129–133 (2014). 24870240 10.1038/nature13389

[8] Huang, Z., Wang, C. & Dong, G. A hydrazone-based exo-directing-group strategy for β C–H oxidation of aliphatic amines. *Angew. Chem. Int. Ed.* **55**, 5299–5303 (2016). 10.1002/anie.201600912 27001210

[9] Wu, Y., Chen, Y.-Q., Liu, T., Eastgate, M. D. & Yu, J.-Q. Pd-catalyzed γ-C(sp^3^)–H arylation of free amines using a transient directing group. *J. Am. Chem. Soc.* **138**, 14554–14557 (2016). 27786439 10.1021/jacs.6b09653 PMC5535771

[10] Chu, J. C. K. & Rovis, T. Amide-directed photoredox-catalysed C–C bond formation at unactivated *sp*^3^ C–H bonds. *Nature* **539**, 272–275 (2016). 27732580 10.1038/nature19810 PMC5574171

[11] Choi, G. J., Zhu, Q., Miller, D. C., Gu, C. J. & Knowles, R. R. Catalytic alkylation of remote C–H bonds enabled by proton-coupled electron transfer. *Nature* **539**, 268–271 (2016). 27732585 10.1038/nature19811 PMC5704892

[12] Morcillo, S. P. et al. Photoinduced remote functionalization of amides and amines using electrophilic nitrogen radicals. *Angew. Chem. Int. Ed.* **57**, 12945–12949 (2018). 10.1002/anie.201807941 PMC6221136 30074300

[13] Stateman, L. M., Nakafuku, K. M. & Nagib, D. A. Remote C–H functionalization via selective hydrogen atom transfer. *Synthesis* **50**, 1569–1586 (2018). 29755145 10.1055/s-0036-1591930 PMC5940016

[14] Herbort, J. H., Bednar, T. N., Chen, A. D., RajanBabu, T. V. & Nagib, D. A. γ C–H functionalization of amines via triple H-atom transfer of a vinyl sulfonyl radical chaperone. *J. Am. Chem. Soc.* **144**, 13366–13373 (2022). 35820104 10.1021/jacs.2c05266 PMC9405708

[15] Lee, M. & Sanford, M. S. Platinum-catalyzed, terminal-selective C(sp^3^)–H oxidation of aliphatic amines. *J. Am. Chem. Soc.* **137**, 12796–12799 (2015). 26439251 10.1021/jacs.5b09099 PMC4828934

[16] Mbofana, C. T., Chong, E., Lawniczak, J. & Sanford, M. S. Iron-catalyzed oxyfunctionalization of aliphatic amines at remote benzylic C–H sites. *Org. Lett.* **18**, 4258–4261 (2016). 27529646 10.1021/acs.orglett.6b02003 PMC5356366

[17] Osberger, T. J., Rogness, D. C., Kohrt, J. T., Stepan, A. F. & White, M. C. Oxidative diversification of amino acids and peptides by small-molecule iron catalysis. *Nature* **537**, 214–219 (2016). 27479323 10.1038/nature18941 PMC5161617

[18] Le, C., Liang, Y., Evans, R. W., Li, X. & MacMillan, D. W. C. Selective *sp*^3^ C–H alkylation via polarity-match-based cross-coupling. *Nature* **547**, 79–83 (2017). 28636596 10.1038/nature22813 PMC5655994

[19] Shaw, M. H., Shurtleff, V. W., Terrett, J. A., Cuthbertson, J. D. & MacMillan, D. W. C. Native functionality in triple catalytic cross-coupling: sp^3^ C–H bonds as latent nucleophiles. *Science* **352**, 1304–1308 (2016). 27127237 10.1126/science.aaf6635 PMC5114852

[20] Chen, W., Ma, L., Paul, A. & Seidel, D. Direct α-C–H bond functionalization of unprotected cyclic amines. *Nat. Chem.* **10**, 165–169 (2018). 29359746 10.1038/nchem.2871 PMC5942596

[21] Paul, A. & Seidel, D. α-Functionalization of cyclic secondary amines: Lewis acid promoted addition of organometallics to transient imines. *J. Am. Chem. Soc.* **141**, 8778–8782 (2019). 31117670 10.1021/jacs.9b04325 PMC6688489

[22] Beatty, J. W. & Stephenson, C. R. J. Amine functionalization via oxidative photoredox catalysis: methodology development and complex molecule synthesis. *Acc. Chem. Res.* **48**, 1474–1484 (2015). 25951291 10.1021/acs.accounts.5b00068 PMC4440623

[23] Murugesan, K. et al. Photoredox-catalyzed site-selective generation of carbanions from C(sp^3^)–H bonds in amines. *ACS Catal.* **12**, 3974–3984 (2022).

[24] Zuo, Z. et al. Merging photoredox with nickel catalysis: Coupling of α-carboxyl sp^3^-carbons with aryl halides. *Science* **345**, 437–440 (2014). 24903563 10.1126/science.1255525 PMC4296524

[25] Miyake, Y., Nakajima, K. & Nishibayashi, Y. Visible-light-mediated utilization of α-aminoalkyl radicals: addition to electron-deficient alkenes using photoredox catalysts. *J. Am. Chem. Soc.* **134**, 3338–3341 (2012). 22296639 10.1021/ja211770y

[26] Thullen, S. M. & Rovis, T. A mild hydroaminoalkylation of conjugated dienes using a unified cobalt and photoredox catalytic system. *J. Am. Chem. Soc.* **139**, 15504–15508 (2017). 29048886 10.1021/jacs.7b09252

[27] Czyz, M. L. et al. Photocatalytic generation of alkyl carbanions from aryl alkenes. *Nat. Catal.* **7**, 1316–1329 (2024).

[28] Zheng, S., Wang, W. & Yuan, W. Remote and proximal hydroaminoalkylation of alkenes enabled by photoredox/nickel dual catalysis. *J. Am. Chem. Soc.* **144**, 17776–17782 (2022). 36136777 10.1021/jacs.2c08039

[29] Herzon, S. B. & Hartwig, J. F. Hydroaminoalkylation of unactivated olefins with dialkylamines. *J. Am. Chem. Soc.* **130**, 14940–14941 (2008). 18937477 10.1021/ja806367e PMC4852863

[30] DiPucchio, R. C., Rosca, S.-C. & Schafer, L. L. Hydroaminoalkylation for the catalytic addition of amines to alkenes or alkynes: diverse mechanisms enable diverse substrate scope. *J. Am. Chem. Soc.* **144**, 11459–11481 (2022). 35731810 10.1021/jacs.1c10397

[31] Roesky, P. W. Catalytic hydroaminoalkylation. *Angew. Chem. Int. Ed.* **48**, 4892–4894 (2009). 10.1002/anie.200900735 19437521

[32] Kubiak, R., Prochnow, I. & Doye, S. Titanium-catalyzed hydroaminoalkylation of alkenes by C–H bond activation at sp^3^ centers in the α-position to a nitrogen atom. *Angew. Chem. Int. Ed.* **48**, 1153–1156 (2009). 10.1002/anie.200805169 19116994

[33] Daneshmand, P. et al. Cyclic ureate tantalum catalyst for preferential hydroaminoalkylation with aliphatic amines: mechanistic insights into substrate controlled reactivity. *J. Am. Chem. Soc.* **142**, 15740–15750 (2020). 32786765 10.1021/jacs.0c04579

[34] Thompson, M. J., Yang, D., Higgins, J. B. M. & Schafer, L. L. Regioselective hydroaminoalkylation with silylated alkenes for β-amino acid synthesis. *J. Am. Chem. Soc.* **147**, 13415–13423 (2025). 40198753 10.1021/jacs.4c18706

[35] Zhao, H. & Leonori, D. Minimization of back-electron transfer enables the elusive sp^3^ C−H functionalization of secondary anilines. *Angew. Chem. Int. Ed.* **60**, 7669–7674 (2021). 10.1002/anie.202100051 PMC8048505 33459469

[36] Kaiser, D. et al. A General acid-mediated hydroaminomethylation of unactivated alkenes and alkynes. *Angew. Chem. Int. Ed.* **58**, 14639–14643 (2019). 10.1002/anie.201906910 PMC6790944 31482639

[37] Hsu, C. et al. Leveraging electron-deficient iminium intermediates in a general synthesis of valuable amines. *Angew. Chem. Int. Ed.* **61**, e202115435 (2022). 10.1002/anie.202115435 PMC9311413 35103377

[38] Li, J., Oost, R., Maryasin, B., González, L. & Maulide, N. A redox-neutral synthesis of ketones by coupling of alkenes and amides. *Nat. Commun.* **10**, 2327 (2019). 31127092 10.1038/s41467-019-10151-x PMC6534616

[39] Li, J., Preinfalk, A. & Maulide, N. Enantioselective redox-neutral coupling of aldehydes and alkenes by an iron-catalyzed “catch–release” tethering approach. *J. Am. Chem. Soc.* **141**, 143–147 (2019). 30576130 10.1021/jacs.8b12242 PMC6342409

[40] Spieß, P. et al. Nms-amides: An amine protecting group with unique stability and selectivity. *Chem. Eur. J.* **29**, e202301312 (2023). 37283481 10.1002/chem.202301312 PMC10946766

[41] Kuij, C. et al. Intracellular bacterial growth is controlled by a kinase network around PKB/AKT1. *Nature* **450**, 725–730 (2007). 18046412 10.1038/nature06345

[42] Reuveni, H. et al. Toward a PKB inhibitor: modification of a selective PKA inhibitor by rational design. *Biochemistry* **41**, 10304–10314 (2002). 12162746 10.1021/bi0202530

[43] Bakker, A. T. et al. Discovery of isoquinoline sulfonamides as allosteric gyrase inhibitors with activity against fluoroquinolone-resistant bacteria. *Nat. Chem.* **16**, 1462–1472 (2024). 38898213 10.1038/s41557-024-01516-x PMC11374673

[44] Grimm, S. H. et al. Comprehensive structure-activity-relationship of azaindoles as highly potent FLT3 inhibitors. *Bioorg. Med. Chem.* **27**, 692–699 (2019). 30661740 10.1016/j.bmc.2019.01.006

[45] Békés, M., Langley, D. R. & Crews, C. M. PROTAC targeted protein degraders: the past is prologue. *Nat. Rev. Drug Discov.* **21**, 181–200 (2022). 35042991 10.1038/s41573-021-00371-6 PMC8765495

[46] Rizvi, S. F. A., Zhang, L., Zhang, H. & Fang, Q. Peptide-drug conjugates: design, chemistry, and drug delivery system as a novel cancer theranostic. *ACS Pharmacol. Transl. Sci.* **7**, 309–334 (2024). 38357281 10.1021/acsptsci.3c00269 PMC10863443

