# Supplementary material for: An alkyl-swap platform for late-stage modification of secondary N-methylamines
Source: Nat Chem. 2026 Jun 15;18(9):1650–7. doi: 10.1038/s41557-026-02178-7 (PMC13524786; doi:10.1038/s41557-026-02178-7)

nature chemistry

Supplementary information

https://doi.org/10.1038/s41557-026-02178-7

An alkyl-swap platform for late-stage modification of secondary *N*-methylamines

In the format provided by the authors and unedited

Contents

1. **General Information** 2
2. **Optimization** 3
3. **Preparation of starting materials** 5
   - 3.1 **General Procedure S1: demethylation** 5
   - 3.2 **General Procedure S2: amide coupling** 6
   - 3.3 **General Procedure S3: preparation of peptides** 7
   - 3.4 **Starting Materials: Analytical Data** 9
4. **Additional scope entries** 24
5. **General Procedures for preparation of products** 25
   - 5.1 **General Procedure 1 for functionalization of alkenes/alkynes with ammonium salts** 25
   - 5.2 **General Procedure 2 for functionalization of alkenes/alkynes with ammonium salts** 25
   - 5.3 **General Procedure 3 for functionalization of alkenes/alkynes with free amines** 26
   - 5.4 **General Procedure 4 for functionalization of alkenes/alkynes with free amines** 27
   - 5.5 **Comparison of General Procedures 1 & 2 and 3 & 4** 28
   - 5.6 **Representative example of the reaction** 29
   - 5.7 **Products: Analytical Data** 30
6. **Mechanistic studies** 117
   - 6.1 **Deuterium labeling** 119
   - 6.2 **NMR analysis** 123
   - 6.3 **Formaldehyde recycling** 128
7. **Comparison with the state-of-the-art approach** 129
8. **References** 131
9. **NMR spectra** 134

1. General Information

Unless otherwise stated, all glassware was dried in the oven before use. All solvents were distilled from appropriate drying agents prior to use. All reagents were used as received from commercial suppliers unless otherwise stated. Reaction progress was monitored by thin layer chromatography (TLC) performed on aluminum plates coated with silica gel F254 with 0.2 mm thickness. Chromatograms were visualized by fluorescence quenching with UV light at 254 nm or by staining using potassium permanganate. Flash column chromatography was performed using silica gel 60 (230-400 mesh, Merck and co.), or pre-packed columns. Preparative high-performance liquid chromatography was performed using XBridge^{TM} Prep. C18 (5 µm, 30 × 150 mm). Neat infrared spectra were recorded using a Perkin-Elmer Spectrum 100 FT-IR spectrometer. Wavenumbers (ν_{max}) are reported in cm^{-1}. Mass spectra were obtained using a Finnigan MAT 8200 (70 eV) or an Agilent 5973 (70 eV) spectrometer, using electrospray ionization (ESI). All ^{1}H NMR and ^{13}C NMR spectra were recorded using a Bruker AV-400, AV-600 or AV-700 spectrometer at 300 K. Chemical shifts are given in parts per million (ppm, δ), referenced to the solvent peaks of: $CDCl_3$ [defined at δ = 7.26 ppm (^{1}H NMR) and δ = 77.16 (^{13}C NMR)], MeOD [defined at δ = 3.31 ppm (^{1}H NMR) and δ = 49.00 (^{13}C NMR)], DMSO-*d6* [defined at δ = 2.50 ppm (^{1}H NMR) and δ = 39.52 ppm (^{13}C NMR)] or CD_2Cl_2 [defined at δ = 5.32 ppm (^{1}H NMR) and δ = 54.00 ppm (^{13}C NMR)]. Coupling constants are quoted in Hz (*J*). ^{1}H NMR splitting patterns are designated as singlet (s), doublet (d), triplet (t), quartet (q), heptet (hept), as they appeared in the spectrum. If the appearance of a signal differs from the expected splitting pattern, the observed pattern is designated as apparent (app). Splitting patterns that could not be interpreted or easily visualized are designated as multiplet (m) or broad (br). When rotameric effects are visible in measured NMR spectra, chemical shifts of equivalent atoms are reported in square brackets ([ ]). Amine substrates are denoted as **SX.XXa** and alkene substrates are denoted as **SX.XXb**. All substrates are commercially available unless stated otherwise. Amine substrates are denoted as **SX.XXa** and alkene/alkyne substrates are denoted as **SX.XXb**. All substrates are commercially available unless stated otherwise. Substrates **S1.12a**[1], **S1.17b**,[2] **S1.18b**,[2] **S1.21b**,[3] **S1.27b**,[4] **S1.28b,**[5] **S1.29b,**[4] **S1.31b**,[6] **S1.32b**,[7] **S1.34b**,[8] **S1.35b**,[8] **S1.42b**,[9] **S1.43b,**[10] and **S3.04b**[11] were prepared according to previously described procedures.

2. Optimization

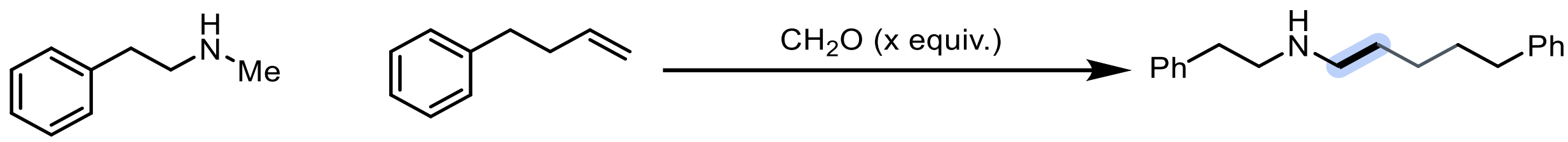

1.51a 1.51b 1.51

Entry	Equiv. of alkene	Equiv. of CH_2O	Acid	Counteranion[a]	Time	Temp.	Solvent	Yield (%)[b]
**1**	1.1	1.0	TFA (1 equiv.)	/	16 h	25 °C	HFIP (1.2M)	**n.d.**
**2**	1.3	1.0	TFA (1 equiv.)	/	16 h	75 °C	ACN (1.2M)	**<5%**
**3**	1.3	1.0	TFA (1 equiv.)	/	16 h	75 °C	DCM (1.2M)	**n.d.**
**4**	1.3	1.0	TFA (1 equiv.)	/	16 h	75 °C	HFIP (1.2M)	**79***
**5**	1.3	1.0	/	/	16 h	75 °C	HFIP (1.2M)	**n.d.**
**6**	1.1	1.0	TFA (1 equiv.)	/	16 h	75 °C	HFIP (1.2M)	**90 (86)**
**7**	1.1	1.0	TFA (1 equiv.)	/	16 h	75 °C	HFIP (0.6M)	**80**
**8**	1.1	1.0	TFA (1 equiv.)	/	2 h	100 °C	HFIP (1.2M)	**68**
**9**	1.1	1.0	TFA (0.5 equiv.)	/	16 h	75 °C	HFIP (1.2M)	**42**
**10**	1.1	1.0	TFA (10 equiv.)	/	16 h	75 °C	HFIP (1.2M)	**n.d.**
**11**	1.1	2.0	TFA (1 equiv.)	/	16 h	75 °C	HFIP (1.2M)	**55**
**12**	1.1	1.0	AcOH (1 equiv.)	/	16 h	75 °C	HFIP (1.2M)	**82**
**13**	1.1	1.0[c]	TFA (1 equiv.)	/	16 h	75 °C	HFIP (1.2M)	**55**
**14**	1.1	1.0[d]	TFA (1 equiv.)	/	16 h	75 °C	HFIP (1.2M)	**74**
**15**	1.1	1.0	/	CF_3COO^-	16 h	75 °C	HFIP (1.2M)	**87**
**16**	1.1	1.0	/	Cl^-	16 h	75 °C	HFIP (1.2M)	**86**

**17**	1.1	1.0	/	CH_3COO^-	16 h	75 °C	HFIP (1.2M)	**83**
**18**	1.1	0.5	TFA (1 equiv.)	/	16 h	75 °C	HFIP (1.2M)	**68**
**19**	1.1	0.4	TFA (1 equiv.)	/	16 h	75 °C	HFIP (1.2M)	**55**
**20**	1.1	0.3	TFA (1 equiv.)	/	16 h	75 °C	HFIP (1.2M)	**47**
**21**	1.1	0.2	TFA (1 equiv.)	/	16 h	75 °C	HFIP (1.2M)	**38**
**22**	1.1	0.1	TFA (1 equiv.)	/	16 h	75 °C	HFIP (1.2M)	**27**
**23**	1.1	1.0	TFA (1 equiv.)	/	2 h	75 °C	HFIP (1.2M)	**33**

Supplementary Table S1. Optimization of reaction parameters. All reactions were carried out on a 0.2 mmol scale. [a]The corresponding ammonium salt was used as the starting material. [b]NMR yield determined using mesitylene as internal standard; the number in parentheses corresponds to the isolated yield. [c]37% water solution of formaldehyde was used as a source of formaldehyde. [d] 1,3,5-Trioxane was used as a source of formaldehyde. *Additional by-products are formed due to unselectivity in the case of a second hydride transfer.

3. Preparation of starting materials

3.1 General Procedure S1: demethylation

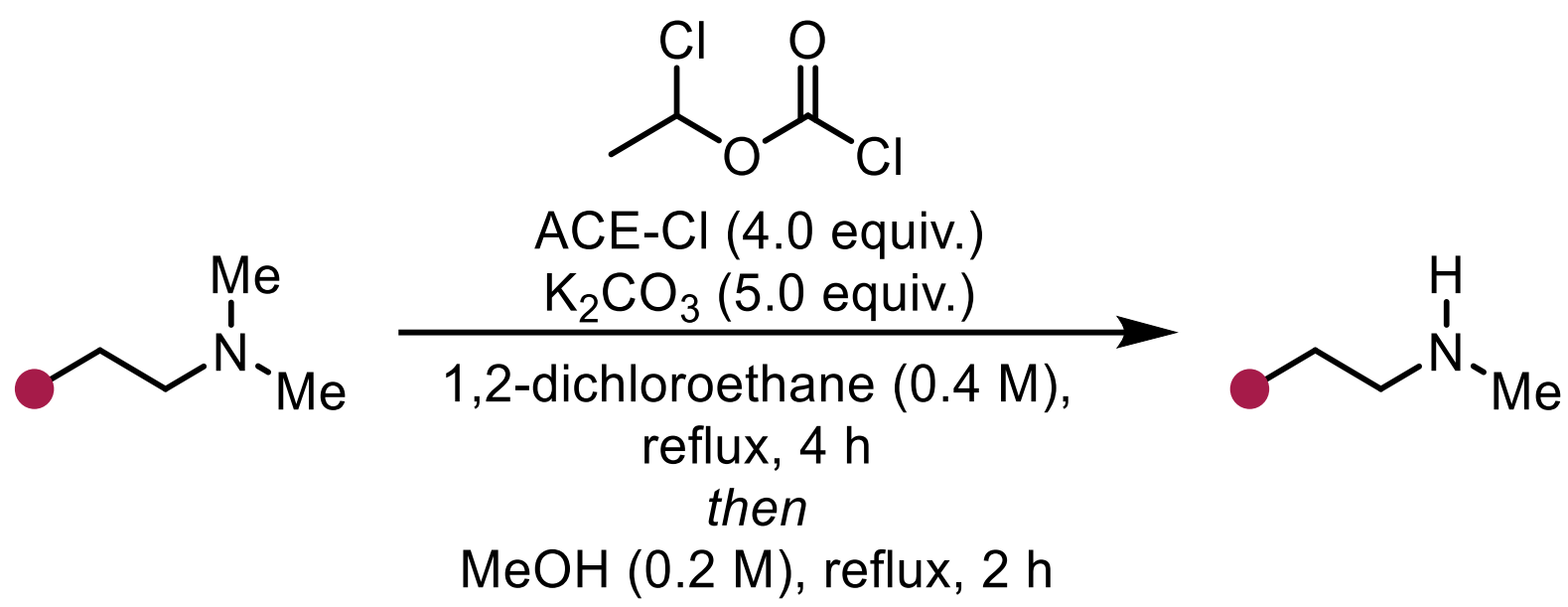

Starting materials **S1.08a–S1.10a** were prepared using a slightly modified previously reported procedure.[12]

At ambient temperature and under an atmosphere of argon, potassium carbonate (5.0 equiv.) was added to a solution of the corresponding dimethylamine (1.0 equiv.) in anhydrous 1,2 dichloroethane (0.4 M). α-Chloroethylchloroformate (ACE-Cl, 2.0 equiv.) was added dropwise, and the resulting suspension was heated to reflux and stirred at that temperature for 2 h, after which a second aliquot of ACE-Cl (2.0 equiv.) was added. The reaction was stirred at reflux for an additional 2 h and was then allowed to cool to 25 °C. The solids were filtered off, and the solvent was evaporated under reduced pressure. The crude material was then dissolved in methanol (0.2 M), and the solution heated at reflux, with stirring, for 2 h. Subsequently, the mixture was allowed to cool to ambient temperature and then concentrated under reduced pressure. To the resulting residue, aqueous sodium hydroxide (1 M) was added until the pH reached 10. The product was extracted with dichloromethane (3 ×), the organic phases were combined, and the resulting solution was dried over magnesium sulfate. Following filtration through cotton, the filtrate was concentrated under reduced pressure to afford the crude material, which was purified by flash column chromatography on silica gel to afford the analytically pure desired product.

3.2 General Procedure S2: amide coupling

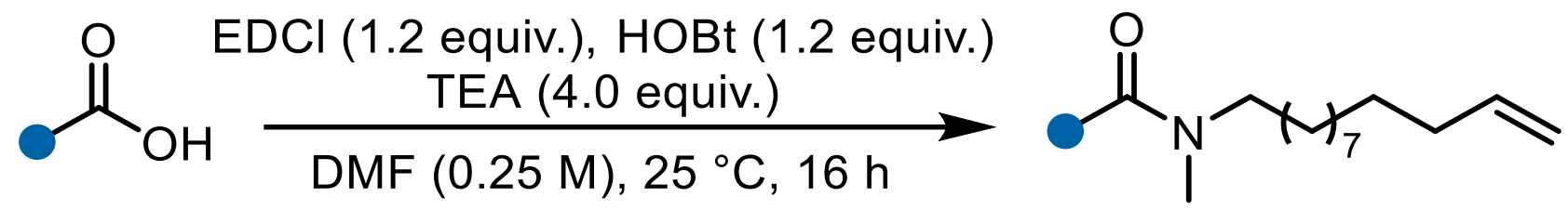

A mixture of the corresponding carboxylic acid (1.0 equiv.), *N*-methyl undec-10-en-1-amine (1.0 equiv.), 1-(3-dimethylaminopropyl)-3-ethylcarbodiimide hydrochloride (EDCl, 1.2 equiv.), 1 hydroxybenzotriazole hydrate (HOBt, 1.2 equiv.), and triethylamine (4.0 equiv.) in dimethylformamide (0.25 M) was stirred for 12 h at 25 °C. Subsequently, water (15 mL per mmol substrate) was added, followed by extraction of the resulting solution with dichloromethane (3 ×). The organic layers were combined, dried over magnesium sulfate and filtered through cotton. The filtrate was concentrated under reduced pressure to afford the crude material, which was purified by flash column chromatography on silica gel to afford the analytically pure desired products.

3.3 General Procedure S3: preparation of peptides

All peptides used in this work were synthesized using the following synthetic route:

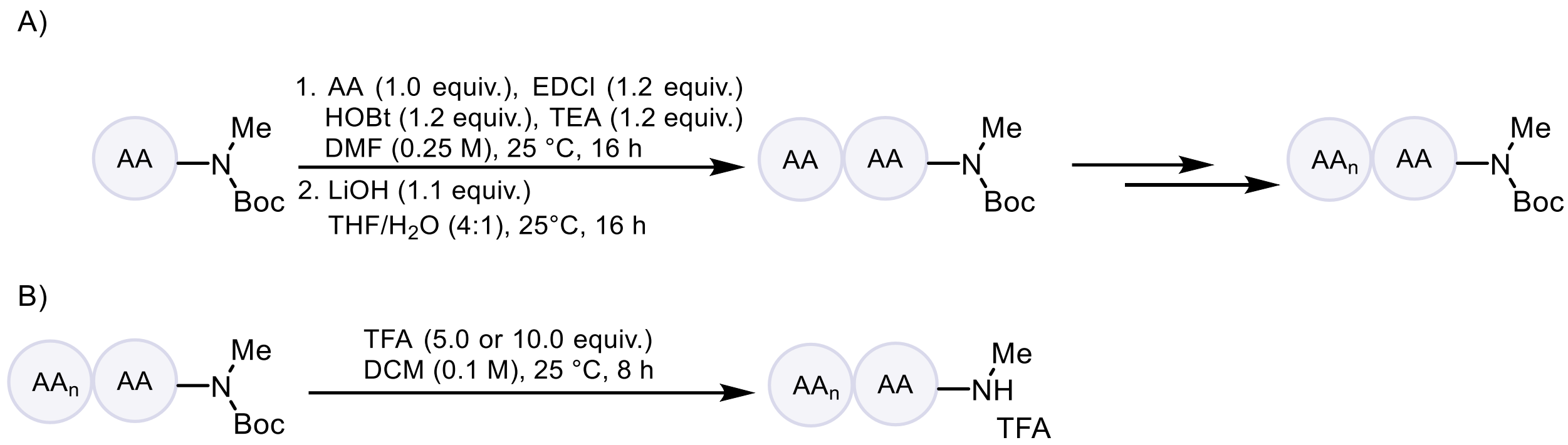

Supplementary Fig. S1. General workflow for peptide preparation.

A) A mixture of the corresponding amino acid AA (1.0 equiv.), amine (1.0 equiv.), 1-(3-dimethylaminopropyl)-3-ethylcarbodiimide hydrochloride (EDCl, 1.2 equiv.), 1-hydroxybenzotriazole hydrate (HOBt, 1.2 equiv.), triethylamine (4.0 equiv.) in dimethylformamide (0.25 M) was stirred for 12 h at 25 °C. Water (30 mL) was then added, and the product was extracted with dichloromethane (3 × 5 mL) and dried over magnesium sulfate. The dried solution was filtered, and the filtrate was concentrated under reduced pressure to afford the crude material, which was purified by flash column chromatography on silica gel with dichloromethane/methanol in ratio 90:10 to afford the analytically pure desired product.

A solution of lithium hydroxide (1.1 equiv.) in tetrahydrofuran/water (4:1, 0.5 M) was added to the ester-protected compound and the resulting solution was stirred at 25 °C for 16 h. After that, aqueous HCl (3 M) was added until the pH reached 3. The product was extracted with ethyl acetate (3 × 5 mL) and dried over magnesium sulfate. The dried solution was filtered, and the filtrate was concentrated under reduced pressure to afford the crude product, which was used in the next step without any further purification steps.

B) To a stirred solution of the corresponding Boc-protected amine (1.0 equiv.) in dichloromethane (0.1 M), TFA (5.0 or 10.0 equiv.) was added, and the resulting mixture was stirred at ambient temperature for 8 h. Subsequently, removal of excess TFA under reduced

pressure afforded the desired amine as the TFA salt. The compound was used in the next step without further purification.

3.4 Starting Materials: Analytical Data

3-(4-Chlorophenyl)-*N*-methyl-3-(pyridin-2-yl)propan-1-amine (S1.09a)

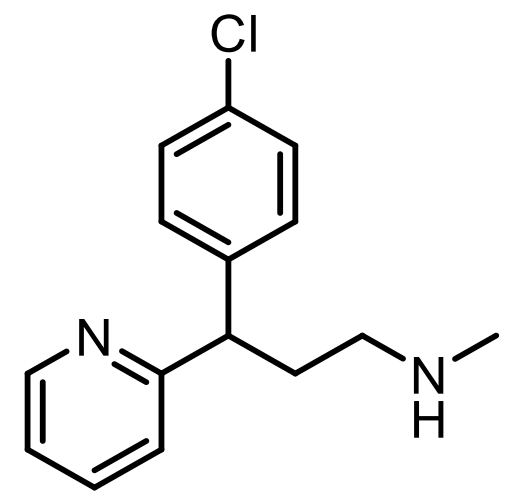

$C_{15}H_{17}ClN_2$
MW: 260.1080

Prepared following general procedure **S1** using Chlorpheniramine (550 mg, 2.00 mmol, 1.00 equiv.). Purification by flash column chromatography (dichloromethane/methanol 97:3 to 90:10) gave the title compound (62.4 mg, 0.240 mmol, 12%) as a yellow oil.

^{1}H NMR (400 MHz, CDCl$_3$) δ 8.69 – 8.45 (m, 1H), 7.55 (td, *J* = 7.7, 1.9 Hz, 1H), 7.31 – 7.20 (m, 4H), 7.17 – 7.04 (m, 2H), 4.13 (t, *J* = 7.6 Hz, 1H), 2.50 (t, *J* = 7.1 Hz, 2H), 2.47 – 2.39 (m, 1H), 2.36 (s, 3H), 2.29 – 2.09 (m, 1H). *The NH proton was not observed.*

The spectroscopic properties are consistent with the data available in the literature.[13]

1-(4-Fluorophenyl)-1-(3-(methylamino)propyl)-1,3-dihydroisobenzofuran-5-carbonitrile (S1.10a)

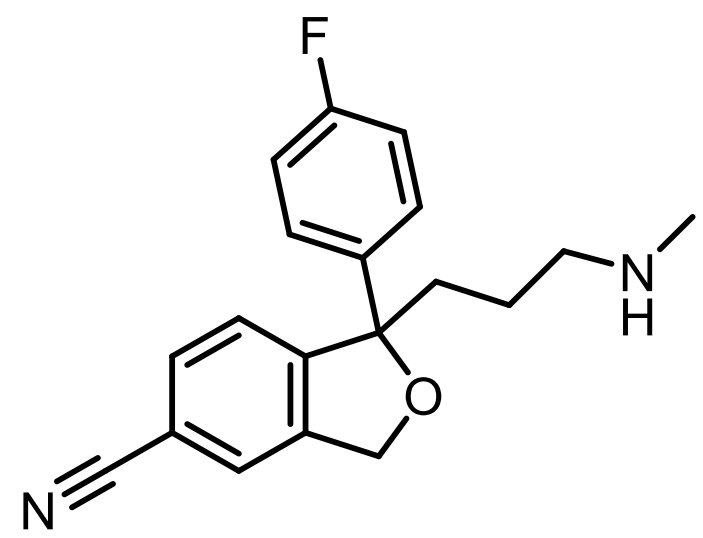

$C_{19}H_{19}FN_2O$
MW: 310.3724

Prepared following general procedure **S1** using Citalopram (500 mg, 1.23 mmol, 1.00 equiv.). Purification by flash column chromatography (dichloromethane/methanol 97:3 to 90:10) gave the title compound (230 mg, 0.741 mmol, 60%) as a white amorphous solid.

^{1}H NMR (400 MHz, $CDCl_3$) δ 7.59 (d, *J* = 7.9 Hz, 1H), 7.50 (s, 1H), 7.45 – 7.38 (m, 3H), 7.05 – 6.96 (m, 2H), 5.17 (q, *J* = 13.0 Hz, 2H), 2.59 (t, *J* = 7.1 Hz, 2H), 2.42 – 2.35 (m, 3H), 2.31 – 2.10 (m, 2H), 1.62 – 1.46 (m, 1H), 1.46 – 1.31 (m, 1H). *The NH proton was not observed.*

The spectroscopic properties are consistent with the data available in the literature.[14]

3-((1-Benzyl-1*H*-indazol-3-yl)oxy)-*N*-methylpropan-1-amine (S1.11a)

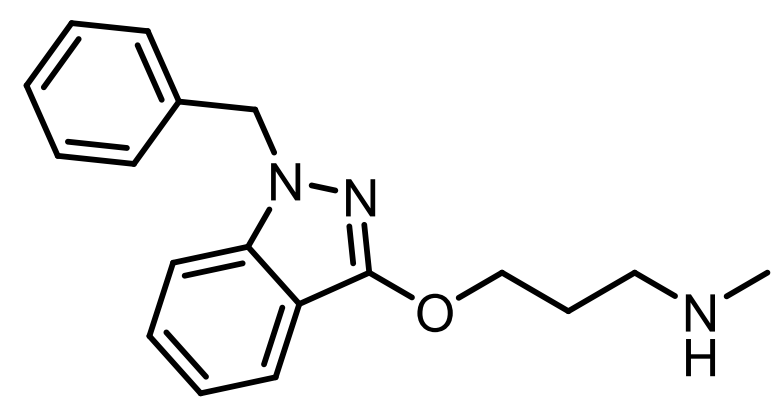

$C_{18}H_{21}N_3O$
MW: 295.3860

Prepared following general procedure **S1** using Benzydamine (618 mg, 2.00 mmol, 1.00 equiv.). gave the title compound (230 mg, 0.741 mmol, 60%) as a yellow oil.

^{1}H NMR (400 MHz, CDCl$_3$) δ 7.71 (dt, *J* = 8.0, 1.1 Hz, 1H), 7.35 – 7.27 (m, 4H), 7.22 – 7.17 (m, 3H), 7.11 – 7.03 (m, 1H), 5.41 (s, 2H), 4.51 (t, *J* = 6.3 Hz, 2H), 2.83 (t, *J* = 7.0 Hz, 2H), 2.49 (s, 3H), 2.09 (app p, *J* = 6.6 Hz, 2H), 1.56 (br s, 1H).

^{13}C NMR (176 MHz, CDCl$_3$) δ 156.1, 141.6, 137.5, 128.6, 127.5, 127.4, 127.1, 120.2, 119.2, 113.2, 108.8, 67.4, 52.4, 49.0, 29.6.

HRMS (ESI$^+$): exact mass calculated for $[M+H]^+$ ($C_{31}H_{39}{}^{35}ClN_2O_3Na$) requires *m/z* 296.1757, found *m/z* 296.1760.

IR (neat) ν_{max}: 3255, 3087, 2980, 2893, 1522, 1488, 1333, 1275, 1187, 1034, 911 cm^{-1}.

2-(1-(4-Chlorobenzoyl)-5-methoxy-2-methyl-1*H*-indol-3-yl)-*N*-methyl-*N*-(undec-10-en-1-yl)acetamide (S1.16b)

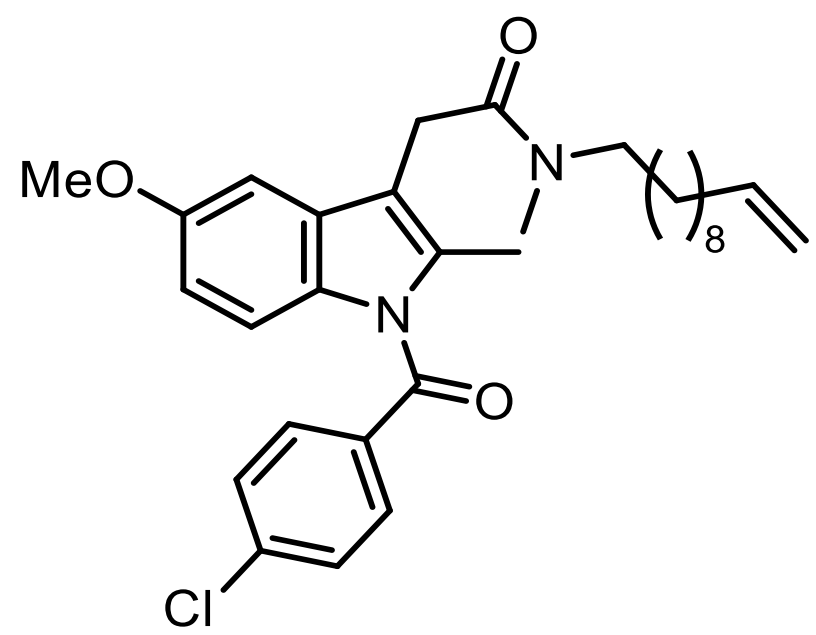

$C_{31}H_{39}ClN_2O_3$
MW: 522.2649

Prepared following general procedure **S2** using Indomethacin (716 mg, 2.00 mmol, 1.00 equiv.). Purification by flash column chromatography (dichloromethane/methanol 97:3 to 90:10) gave the title compound (773.0 mg, 1.48 mmol, 74%) as a yellow oil.

Rotameric effects are visible in the measured NMR spectra.

^{1}H NMR (600 MHz, CDCl$_3$) δ 7.73 – 7.61 (m, 2H), 7.50 – 7.42 (m, 2H), 7.01 (dd, *J* = 7.8, 2.5 Hz, 1H), 6.81 (dd, *J* = 9.0, 4.1 Hz, 1H), 6.64 (dd, *J* = 9.1, 2.6 Hz, 1H), 5.88 – 5.72 (m, 1H), 5.02 – 4.89 (m, 2H), 3.81 (s, 3H), 3.70 (d, *J* = 8.6 Hz, 2H), 3.42 – 3.27 (m, 2H), 3.03 (s, 1.5H), 2.94 (s, 1.5H), 2.37 (app d, *J* = 1.7 Hz, 3H), 2.03 (dtdd, *J* = 8.9, 5.1, 2.6, 1.5 Hz, 2H), 1.57 – 1.47 (m, 2H), 1.43 – 1.31 (m, 2H), 1.33 – 1.18 (m, 10H).

^{13}C NMR (151 MHz, CDCl$_3$) δ [169.99, 169.84], [168.42, 168.37], [156.11, 156.09], [139.34, 139.24], 139.31, [135.32, 135.28], [134.14, 134.11], 131.3 (2C), [131.04, 131.01], 130.97, 129.2 (2C), [114.97, 114.93], [114.32, 114.24], [113.88, 113.66], [111.69, 111.64], [101.76, 101.73], 55.8, [50.37, 48.45], 35.8, [33.92, 33.90], 33.8, [30.97, 30.35], [29.62, 29.57], [29.53, 29.52], [29.22, 29.20], [29.03, 29.01], 28.6, [26.99, 26.97], 13.6.

HRMS (ESI$^+$): exact mass calculated for $[M+H]^+$ ($C_{31}H_{39}{}^{35}ClN_2O_3Na$) requires *m/z* 545.2541, found *m/z* 545.2550.

IR (neat) ν_{max}: 2944, 2875, 1689, 1673, 1574, 1491, 1388, 1097, 944 cm^{-1}.

(*S*)-2-(4-(4-Chlorophenyl)-2,3,9-trimethyl-6*H*-thieno[3,2-*f*][1,2,4]triazolo[4,3-*a*][1,4]diazepin-6-yl)-*N*-methyl-*N*-(undec-10-en-1-yl)acetamide (S1.19b)

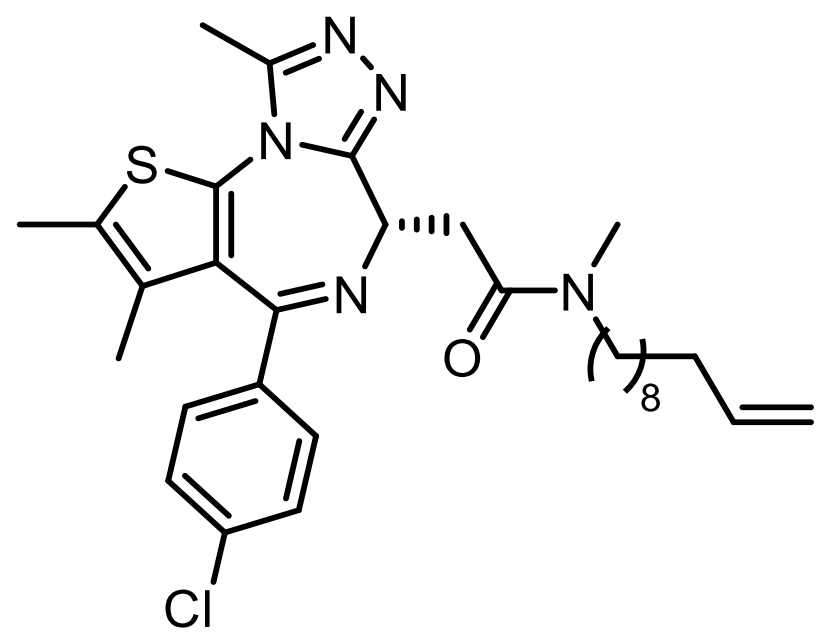

$C_{31}H_{40}ClN_5OS$
MW: 566.2050

Prepared following general procedure **S2** using (+)-JQ1 carboxylic acid (263 mg, 0.660 mmol, 1.00 equiv.). Purification by flash column chromatography (dichloromethane/methanol 97:3 to 90:10) gave the title compound (193 mg, 0.340 mmol, 52%) as a yellow oil.

Rotameric effects are visible in the measured NMR spectra.

^{1}H NMR (600 MHz, CDCl$_3$) δ 7.40 (d, *J* = 8.5 Hz, 2H), 7.32 (d, *J* = 8.7 Hz, 2H), 5.81 (app dqd, *J* = 10.0, 6.7, 1.7 Hz, 1H), 5.04 – 4.87 (m, 2H), 4.81 (dd, *J* = 13.4, 6.2 Hz, 1H), 3.69 – 3.53 (m, 3H), 3.52 – 3.32 (m, 2H), 3.21 (s, 1.5H), 2.98 (s, 1.5H), 2.66 (d, *J* = 0.9 Hz, 3H), 2.39 (s, 3H), 2.05 – 1.96 (m, 2H), 1.71 (dd, *J* = 15.1, 7.3 Hz, 1H), 1.67 (d, *J* = 4.7 Hz, 3H), 1.56 – 1.52 (m, 1H), 1.41 – 1.33 (m, 3H), 1.32 – 1.20 (m, 8H).

^{13}C NMR (151 MHz, CDCl$_3$) δ [170.43, 170.33], [163.67, 163.65], 156.2, [149.90, 149.89], 139.4, [137.09, 137.04], 136.7, [132.41, 132.39], 131.1, 130.7, 130.6, 129.9 (2C), 128.8 (2C), [114.26, 114.25], [54.82, 54.61], 50.5, 48.1, [35.94, 35.37], [35.71, 33.87], 33.9, 29.7, 29.2, 29.0, 28.8, 27.5, [27.06, 27.00], [14.54, 14.53], [13.21, 13.20], [12.02, 12.00].

HRMS (ESI$^+$): exact mass calculated for $[M+H]^+$ ($C_{31}H_{40}{}^{35}ClN_5OS$) requires *m/z* 566.2715, found *m/z* 566.2706.

IR (neat) ν_{max}: 2923, 2853, 1639, 1591, 1528, 1440, 1088, 965, 732, 560 cm^{-1}.

(*4R*)-4-(*(8R,9S,10S,13R,14S,17R*)-10,13-Dimethyl-3,7,12-trioxohexadecahydro-1*H*-cyclopenta[*a*]phenanthren-17-yl)-*N*-methyl-*N*-(undec-10-en-1-yl)pentanamide (S1.20b)

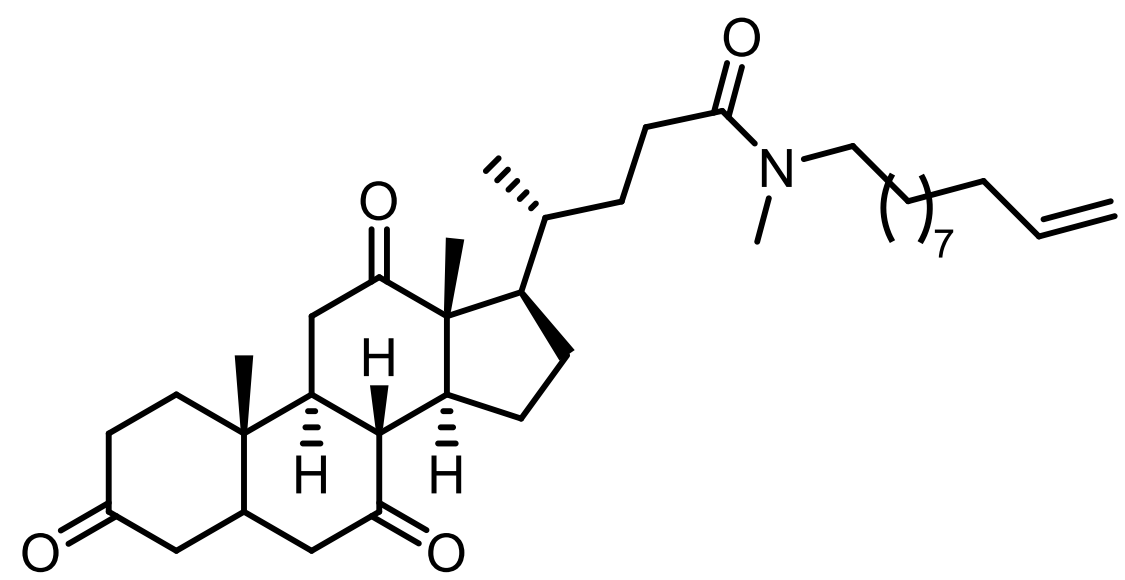

$C_{36}H_{57}NO_4$
MW: 567.8550

Prepared following general procedure **S2** using dehydrocholic acid (805 mg, 2.00 mmol, 1.00 equiv.). Purification by flash column chromatography (dichloromethane/methanol 97:3 to 90:10) gave the title compound (738.5 mg, 1.30 mmol, 65%) as a yellow oil.

Rotameric effects are visible in the measured NMR spectra.

^{1}H NMR (700 MHz, $CDCl_3$) δ 5.79 – 5.70 (m, 1H), 4.93 (dt, *J* = 17.2, 1.9 Hz, 1H), 4.88 – 4.84 (m, 1H), 3.34 – 3.25 (m, 1H), 3.20 (dq, *J* = 21.6, 7.8 Hz, 1H), 2.92 (s, 1.5H), 2.87 (dd, *J* = 13.4, 5.7 Hz, 2H), 2.84 (s, 1.5H), 2.83 – 2.77 (m, 2H), 2.41 – 2.12 (m, 10H), 2.13 – 2.04 (m, 3H), 2.01 – 1.95 (m, 6H), 1.94 – 1.73 (m, 4H), 1.56 (td, *J* = 14.6, 4.5 Hz, 1H), 1.52 – 1.48 (m, 1H), 1.46 – 1.41 (m, 1H), 1.35 (s, 3H), 1.34 – 1.29 (m, 3H), 1.26 – 1.18 (m, 7H), 1.02 (t, *J* = 2.1 Hz, 3H), 0.80 (t, *J* = 6.7 Hz, 3H).

^{13}C NMR (176 MHz, $CDCl_3$) δ [212.18, 212.15], [212.00, 208.75], [209.13, 209.11], [176.69, 173.10], [139.20, 139.14], [114.17, 114.11], [56.92, 56.90], [51.79, 51.76], [50.04, 47.78], [48.97, 46.81], [45.74, 45.70], [45.54, 45.51], [44.97, 42.77], [38.65, 38.63], [36.46, 35.25], [36.00, 35.55], [35.48, 35.39], [33.79, 33.76], 33.41, [31.28, 31.01], [30.56, 30.50], 30.42, 29.92, [29.49, 29.46], [29.39, 29.36], 29.35, [29.08, 29.05], [28.90, 28.87], [28.52, 27.61], [27.57, 27.53], 27.28, [26.85, 26.75], 25.2, [21.89, 18.65], [18.86, 18.84], [11.90, 11.88].

HRMS (ESI$^+$): exact mass calculated for $[M+H]^+$ ($C_{36}H_{57}NO_4Na$) requires *m/z* 590.4180, found *m/z* 590.4171.

IR (neat) ν_{max}: 2963, 2881, 1711, 1700, 1688, 1522, 1386, 1211, 1055, 986 cm^{-1}.

2-(3-Cyano-4-isobutoxyphenyl)-*N*,4-dimethyl-*N*-(undec-10-en-1-yl)thiazole-5-carboxamide (S1.22b)

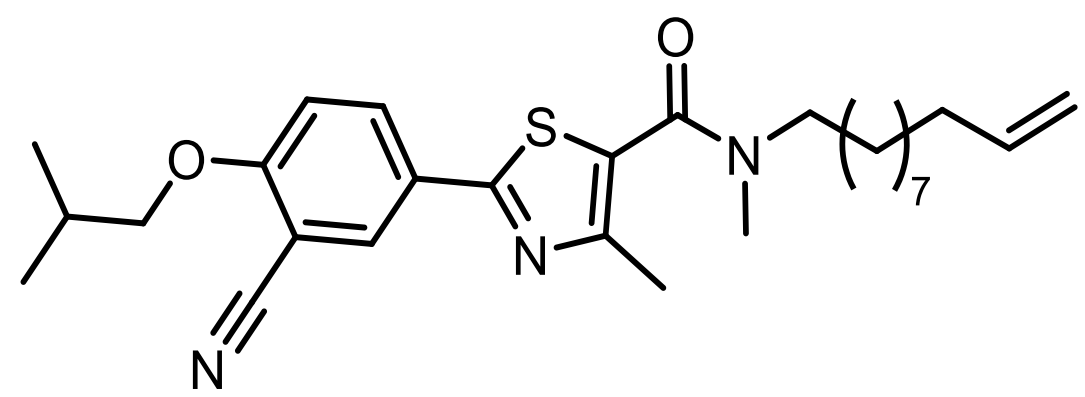

$C_{28}H_{39}N_3O_2S$
MW: 481.6990

Prepared following general procedure **S2** using Febuxostat (633 mg, 2.00 mmol, 1.00 equiv.). Purification by flash column chromatography (dichloromethane/methanol 97:3 to 90:10) gave the title compound (675.1 mg, 1.40 mmol, 70%) as a yellow oil.

^{1}H NMR (400 MHz, CDCl$_3$) δ 8.12 (d, *J* = 2.3 Hz, 1H), 8.03 (dd, *J* = 8.8, 2.3 Hz, 1H), 7.00 (d, *J* = 8.9 Hz, 1H), 5.80 (ddt, *J* = 16.9, 10.1, 6.6 Hz, 1H), 5.06 – 4.88 (m, 2H), 3.89 (d, *J* = 6.5 Hz, 2H), 3.57 – 3.35 (m, 2H), 3.06 (s, 3H), 2.46 (s, 3H), 2.20 (dt, *J* = 13.1, 6.5 Hz, 1H), 2.07 – 1.96 (m, 2H), 1.63 (app br s, 2H), 1.38 – 1.21 (m, 12H), 1.09 (d, *J* = 6.7 Hz, 6H).

^{13}C NMR (101 MHz, CDCl$_3$) δ 164.7, 163.5, 162.2, 152.7, 139.3, 132.44, 132.40, 131.9, 126.3, 115.6, 114.29, 114.26, 114.2, 112.7, 103.0, 75.7, 33.9, 29.54, 29.48, 29.4, 29.2 (2C), 29.0, 28.3 (2C), 26.8, 19.2, 16.5.

HRMS (ESI$^+$): exact mass calculated for $[M+H]^+$ ($C_{28}H_{39}N_3O_2SNa$) requires *m/z* 504.2655, found *m/z* 504.2662.

IR (neat) ν_{max}: 2988, 2834, 2251, 1701, 1512, 1456, 1376, 1211, 1087, 981 cm^{-1}.

N-Methyl-2,4,6-tris(trifluoromethyl)-N-(undec-10-en-1-yl)benzenesulfonamide (1.44b)

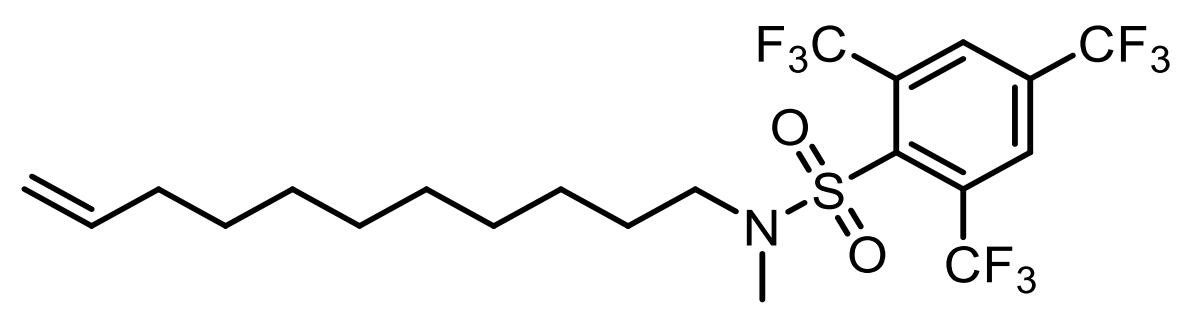

$C_{21}H_{26}F_9NO_2S$
MW: 527.4896

Starting material **S3.01a** was prepared using a slightly modified previously reported procedure.[15]

N-methylundec-10-en-1-amine (183 mg, 1.00 mmol, 1.00 equiv.) was dissolved in dichloromethane (10 mL) at room temperature. DMAP (24 mg, 0.20 mmol, 0.2 mmol) and triethylamine (0.42 mL, 3.0 mmol, 3.0 equiv.) were added at this temperature. Then, the mixture was cooled to 0 °C and 2,4,6-tris(trifluoromethyl)sulfonyl chloride (419 mg, 1.10 mmol, 1.10 equiv.) was added in one portion. The reaction was allowed to slowly warm to ambient temperature over 16 h. The volatiles were removed under reduced pressure and the residue was purified by flash column chromatography on silica gel with a gradient of heptane/ethyl acetate (100:0 to 70:30) to afford the pure product as a colorless amorphous powder (370 mg, 0.701 mmol, 70%).

^{1}H NMR (400 MHz, CDCl$_3$) δ 8.27 (s, 2H), 5.81 (ddt, J = 16.9, 10.1, 6.7 Hz, 1H), 4.99 (dq, J = 17.1, 1.7 Hz, 1H), 4.93 (ddt, *J* = 10.2, 2.3, 1.2 Hz, 1H), 3.37 – 3.22 (m, 2H), 2.74 (s, 3H), 2.12 – 1.98 (m, 2H), 1.74 – 1.59 (m, 2H), 1.45 – 1.23 (m, 12H).

^{13}C NMR (101 MHz, CDCl$_3$) δ 145.2, 139.4, 133.4 (q, *J* = 39.2 Hz), 133.1 (q, *J* = 39.6 Hz, 2C), 129.4 (2C), 126.3, 122.2 (q, *J* = 276.1 Hz, 2C), 114.3, 51.5, 34.5, 33.9, 29.54, 29.52, 29.4, 29.2, 29.0, 27.5, 26.7.

^{19}F NMR (376 MHz, CDCl$_3$) δ -55.75 (6F), -63.56 (3F).

HRMS (ESI$^+$): exact mass calculated for $[M+Na]^+$ ($C_{21}H_{26}F_9O_2SNa^+$) requires m/z 550.1433, found m/z 550.1434.

IR (neat) ν_{max}: 2914, 2848, 1466, 1360, 1282, 1187, 914, 703, 613 cm^{-1}.

N-Methyl-N-(2-(methylamino)ethyl)isoquinoline-5-sulfonamide (S3.01a)

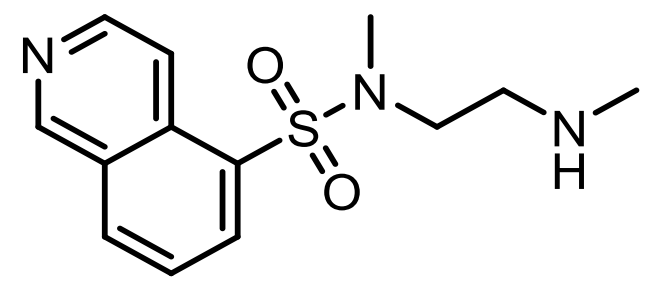

$C_{13}H_{17}N_3O_2S$
MW: 279.3580

Starting material **S3.01a** was prepared using a slightly modified previously reported procedure.[16]

N,N'-dimethylethylenediamine (2.69 mL, 25.0 mmol, 5.00 equiv.) and triethylamine (2.09 mL, 15.0 mmol, 3.00 equiv.) were dissolved in anhydrous dichloromethane (10 mL) in round bottom flask. The solution was cooled in an ice bath to 0 °C and then isoquinoline-5-sulfonyl chloride hydrochloride (1.16 g, 5.00 mmol, 1 equiv.) was added in three portions over 20 mins. The ice bath was then removed and the solution was allowed to warm to room temperature and stirred overnight. The volatiles were removed under reduced pressure and the residue was purified by flash column chromatography (dichloromethane/methanol 97:3 to 90:10) on silica gel to afford the desired product (922 mg, 3.30 mmol, 66%).

^{1}H NMR (400 MHz, $CDCl_3$) δ 9.37 (d, *J* = 1.0 Hz, 1H), 8.73 (d, *J* = 6.2 Hz, 1H), 8.54 (d, *J* = 6.2 Hz, 1H), 8.42 (dd, *J* = 7.5, 1.2 Hz, 1H), 8.24 (d, *J* = 8.1 Hz, 1H), 7.75 (dd, *J* = 8.2, 7.4 Hz, 1H), 3.51 – 3.35 (m, 2H), 3.09 – 2.86 (m, 5H), 2.56 (s, 3H).

The spectroscopic properties are consistent with the data available in the literature.[16]

3-(4-Ethynylphenyl)pyridine (S3.05b)

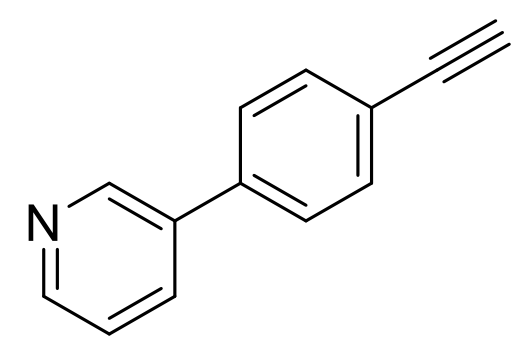

$C_{13}H_9N$
MW: 179.2220

Starting material **S3.05b** was prepared using a slightly modified previously reported procedure.[11]

4-Pyrid-3-ylbenzaldehyde (366 mg, 2.00 mmol, 1.00 equiv.) was dissolved in anhydrous MeOH (15 mL) and stirred under argon at rt. Anhydrous K_2CO_3 (829 mg, 6.00 mmol, 3.00 equiv.) and Bestmann–Ohira reagent (768 mg, 4.00 mmol, 2.00 equiv) were added to the reaction mixture and stirring was continued overnight. The solvent was evaporated, and the remaining residue was dissolved in EtOAc and washed with saturated NH_4Cl aq. solution and water. The organic layer was dried over Na_2SO_4, filtered off and concentrated under reduced pressure. The crude product was purified by flash column chromatography (heptane/ethyl acetate 20:80 to 100:0) on silica gel to afford the desired product (331 mg, 1.85 mmol, 92%).

^{1}H NMR (600 MHz, $CDCl_3$) δ 8.84 (dd, *J* = 2.4, 0.9 Hz, 1H), 8.60 (dd, *J* = 4.9, 1.6 Hz, 1H), 7.86 (ddd, *J* = 7.9, 2.4, 1.6 Hz, 1H), 7.70 – 7.59 (m, 1H), 7.59 – 7.46 (m, 3H), 7.37 (ddd, *J* = 7.9, 4.8, 0.9 Hz, 1H), 3.16 (s, 1H).

The spectroscopic properties are consistent with the data available in the literature.[17]

1-((*S*)-2-(Dodec-11-enamido)-3,3-dimethylbutanoyl)-4-hydroxy-*N*-(4-(4-methylthiazol-5 yl)benzyl)pyrrolidine-2-carboxamide (4.01b)

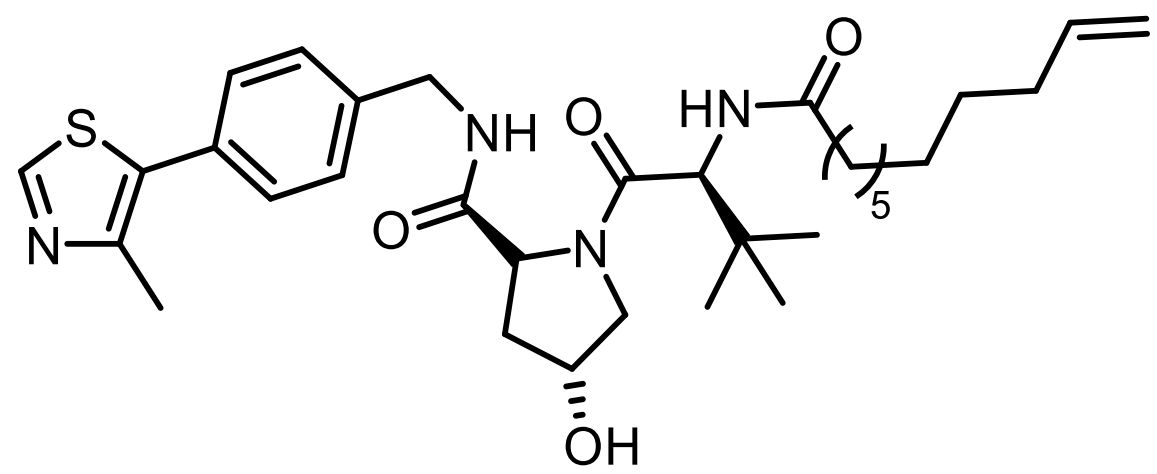

$C_{33}H_{48}N_4O_4S$
MW: 596.8310

A mixture of 10-undecenoyl chloride (46.2 µL, 0.210 mmol, 1.00 equiv.), VHL-ligand ligase (111 mg, 0.260 mmol, 1.20 equiv.), and triethylamine (60.0 µL, 0.420 mmol, 2.00 equiv.) in dichloromethane (0.2 M) was stirred for 12 h at 25 °C. Subsequently, water (30 mL) was added, and the product was extracted with dichloromethane (3 × 5 mL) and dried over magnesium sulfate. The filtrate was concentrated under reduced pressure to afford the crude material, which was used in the next step, for which the yield of this reaction was assumed to be quantitative, without further purification.

^{1}H NMR (600 MHz, CDCl$_3$) δ 8.67 (s, 1H), 7.41 – 7.30 (m, 5H), 6.17 (d, *J* = 8.8 Hz, 1H), 5.78 (ddt, *J* = 16.9, 10.2, 6.7 Hz, 1H), 5.00 – 4.85 (m, 2H), 4.68 (t, *J* = 7.9 Hz, 1H), 4.53 (dd, *J* = 14.2, 7.6 Hz, 3H), 4.32 (dd, *J* = 15.0, 5.3 Hz, 1H), 4.03 (d, *J* = 11.3 Hz, 1H), 3.61 (dd, *J* = 11.2, 3.8 Hz, 1H), 2.53 – 2.43 (m, 4H), 2.15 (t, *J* = 7.7 Hz, 2H), 2.12 – 2.04 (m, 1H), 2.04 – 1.96 (m, 2H), 1.56 (dd, *J* = 15.7, 7.6 Hz, 2H), 1.33 (dd, *J* = 14.2, 7.0 Hz, 2H), 1.25 (s, 9H), 0.94 – 0.88 (m, 9H).
^{13}C NMR (151 MHz, CDCl$_3$) δ 173.8, 171.9, 170.9, 150.5, 148.6, 139.2, 138.2, 131.7, 131.0, 129.6 (2C), 128.2 (2C), 114.1, 70.1, 58.7, 57.5, 56.8, 43.3, 36.6, 36.1, 35.2, 33.9, 29.4, 29.34, 29.30, 29.1, 28.9, 26.5 (3C), 25.7, 16.2.
HRMS (ESI$^+$): exact mass calculated for $[M+H]^+$ ($C_{33}H_{48}N_4O_4S$) requires *m/z* 597.3469, found *m/z* 597.3471.
IR (neat) ν_{max}: 3290, 3011, 2965, 2850, 1730, 1650, 1201, 1028, 751, 700 cm^{-1}.

N-(2-(2,6-Dioxopiperidin-3-yl)-1,3-dioxoisoindolin-5-yl)dodec-11-enamide (4.02b)

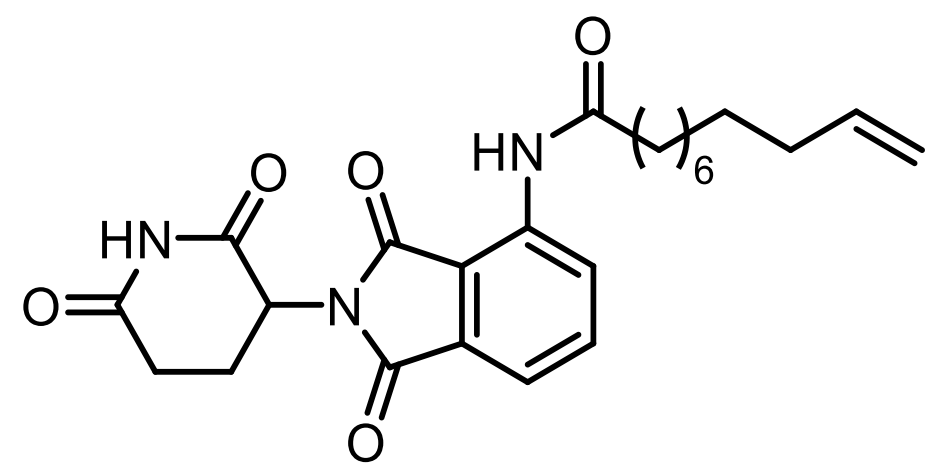

$C_{24}H_{29}N_3O_5$
MW: 439.5120

A mixture of 10-undecenoyl chloride (259 µL, 1.10 mmol, 1.10 equiv.), Pomalidomide (273 mg, 1.00 mmol, 1.00 equiv.), and triethylamine (59.7 µL, 0.420 mmol, 2.00 equiv.) in tetrahydrofuran (0.2 M) was stirred for 4 h at reflux. Subsequently, water (30 mL) was added, and the product was extracted with ethyl acetate (3 × 10 mL) and dried over magnesium sulfate. The dried solution was filtered and the filtrate was concentrated under reduced pressure to afford the crude material, which was purified by flash column chromatography (dichloromethane/methanol 97:3 to 90:10) on silica gel to afford the analytically pure desired product (352 mg, 0.800 mmol, 80%).

^{1}H NMR (400 MHz, DMSO-*d$_6$*) δ 11.15 (br s, 1H), 9.68 (br s, 1H), 8.47 (d, *J* = 8.3 Hz, 1H), 7.82 (t, *J* = 7.8 Hz, 1H), 7.61 (d, *J* = 7.2 Hz, 1H), 5.85 – 5.70 (m, 1H), 5.14 (dd, *J* = 12.5, 5.1 Hz, 1H), 4.95 (dd, *J* = 24.7, 13.7 Hz, 2H), 2.91 (dd, *J* = 21.5, 9.1 Hz, 1H), 2.64 – 2.55 (m, 2H), 2.47 – 2.40 (m, *J* = 7.3 Hz, 2H), 2.12 – 1.90 (m, 3H), 1.73 – 1.53 (m, 2H), 1.41 – 1.18 (m, 10H).

^{13}C NMR (101 MHz, DMSO-*d$_6$*) δ 172.7, 172.0, 169.7, 167.7, 166.6, 138.8, 136.6, 136.1, 131.4, 126.2, 118.3, 116.9, 114.6, 48.9, 36.5, 33.2, 30.9, 28.7 (2C), 28.5 (2C), 28.2, 24.8, 21.9.

HRMS (ESI$^+$): exact mass calculated for $[M+Na]^+$ ($C_{24}H_{29}N_3O_5Na$) requires *m/z* 462.1999, found *m/z* 462.1997.

IR (neat) ν_{max}: 2853, 1701, 1643, 1517, 1451, 1365, 1292, 1192, 1110, 1031, 954, 774 cm^{-1}.

Me-Gly-Pro-Phe-Trp(Tos)-Glu(OMe) (S4.04a)

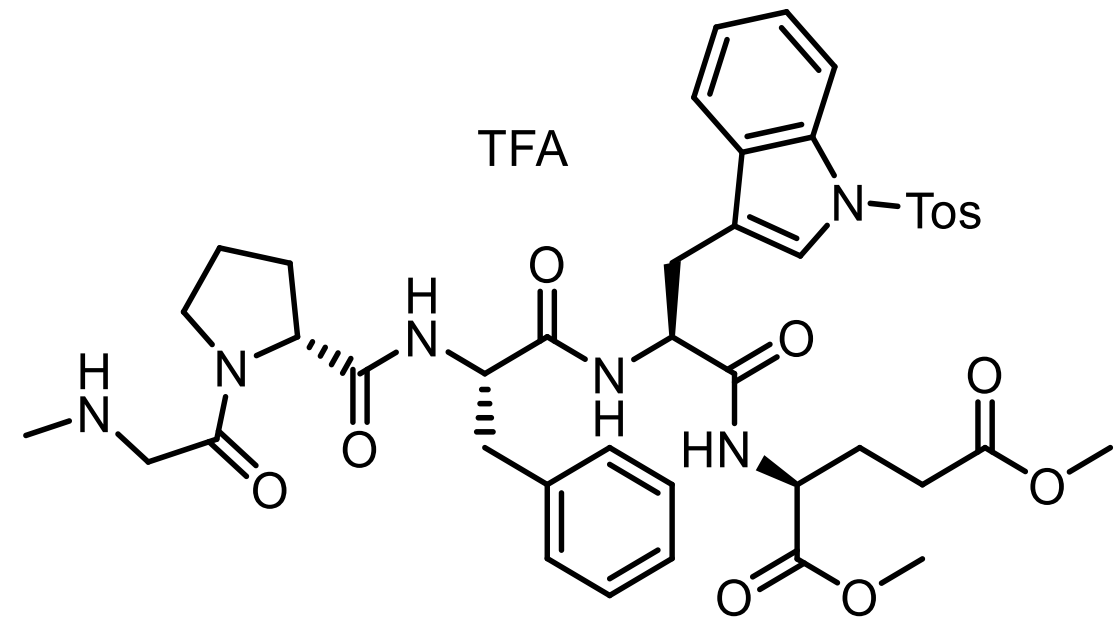

free amine
$C_{42}H_{50}N_6O_{10}S$
MW: 830.9540

The compound was obtained as the TFA salt following general procedure **S3** (steps A and B).

^{1}H NMR (400 MHz, MeOD) δ 8.46 (dd, *J* = 19.1, 7.9 Hz, 1H), 8.21 – 8.12 (m, 1H), 8.05 (d, *J* = 7.6 Hz, 1H), 7.96 – 7.84 (m, 1H), 7.82 – 7.71 (m, 2H), 7.66 – 7.51 (m, 2H), 7.36 – 7.05 (m, 10H), 4.72 – 4.63 (m, 1H), 4.60 – 4.50 (m, 1H), 4.48 – 4.33 (m, 2H), 3.98 (d, *J* = 11.7 Hz, 1H), 3.68 – 3.64 (m, 6H), 3.58 – 3.44 (m, 2H), 3.22 (dd, *J* = 14.7, 5.8 Hz, 1H), 3.16 – 3.05 (m, 2H), 2.93 (dd, *J* = 14.1, 8.7 Hz, 1H), 2.75 (s, 2H), 2.40 (t, *J* = 7.5 Hz, 2H), 2.34 – 2.28 (m, 5H), 2.21 – 2.02 (m, 2H), 1.99 – 1.86 (m, 3H), 1.85 – 1.75 (m, 1H).

^{13}C NMR (101 MHz, MeOD) δ 175.1, 174.7, 173.8, 173.4, 173.3, 173.0, 172.9, 165.4, 146.7, 138.3, 136.4, 132.3, 131.0 (2C), 130.3 (2C), 130.2, 129.5 (2C), 128.0 (2C), 127.8, 125.8, 124.4, 120.7, 119.3, 114.7, 61.6, 56.2, 54.4, 53.1, 52.9, 52.2, 50.6, 47.6, 38.3, 33.8, 30.9, 30.6, 28.2, 27.6, 25.6, 21.5.

HRMS (ESI$^+$): exact mass calculated for $[M+H]^+$ ($C_{42}H_{51}N_6O_{10}S$) requires *m/z* 831.3382, found *m/z* 831.3380.

IR (neat) ν_{max}: 3199, 3012, 2833, 1735, 1640, 1511, 1360, 1210, 970, 702 cm^{-1}.

Me-Gly-Pro-Phe (S4.05a)

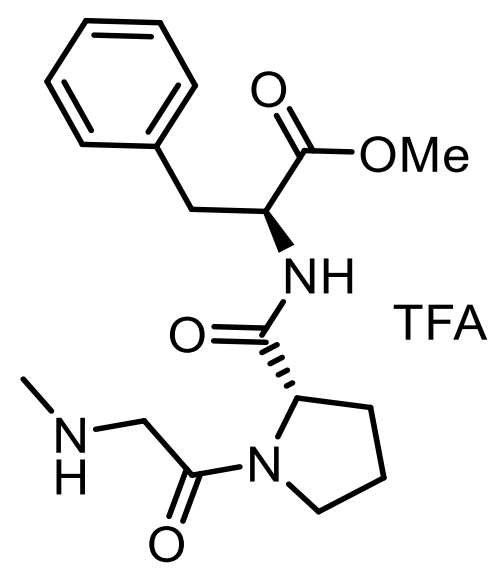

free amine
$C_{18}H_{25}N_3O_4$
MW: 347.4150

The compound was obtained as the TFA salt following general procedure **S3** (steps A and B).

Rotameric effects are visible in the measured NMR spectra.

^{1}H NMR (600 MHz, DMSO-d_6) δ 8.97 – 8.87 (m, 0.73H), 8.83 (d, J = 7.9 Hz, 0.37H), 8.63 (br s, 0.17H), 8.48 (d, J = 8.4 Hz, 0.63H), 7.36 – 7.17 (m, 5H), 4.52 (ddd, J = 9.9, 8.2, 5.1 Hz, 1H), 4.45 – 4.28 (m, 1H), 3.98 – 3.88 (m, 1H), 3.68 – 3.59 (m, 3H), 3.38 (d, J = 8.5 Hz, 4H), 3.10 (ddd, J = 21.4, 13.8, 5.3 Hz, 1H), 2.92 (ddd, J = 28.1, 13.8, 10.0 Hz, 1H), 2.56 – 2.51 (m, 3H), 2.16 – 2.02 (m, 0.32H), 1.97 – 1.87 (m, 0.68H), 1.81 – 1.65 (m, 2H), 1.55 – 1.41 (m, 1H).

^{13}C NMR (101 MHz, DMSO-d_6) δ 171.90, 171.82, 171.15, 170.82, 163.84, 163.67, 137.20, 129.22, 129.06, 128.27, 128.17, 126.62, 126.52, 64.94, 59.29, 58.66, 53.55, 53.23, 53.13, 52.18, 51.96, 48.73, 48.58, 46.78, 45.86, 36.96, 36.16, 32.81, 32.75, 31.58, 29.51, 23.74, 21.91, 15.19.

HRMS (ESI$^+$): exact mass calculated for $[M+H]^+$ ($C_{18}H_{26}N_3O_4$) requires m/z 348.1918, found m/z 348.1920.

IR (neat) ν_{max}: 3211, 3089, 2854, 1722, 1700, 1623, 1507, 1051, 1022, 956 cm^{-1}.

Me-Gly-Pro-Phe-Glu(OMe) (S4.06a)

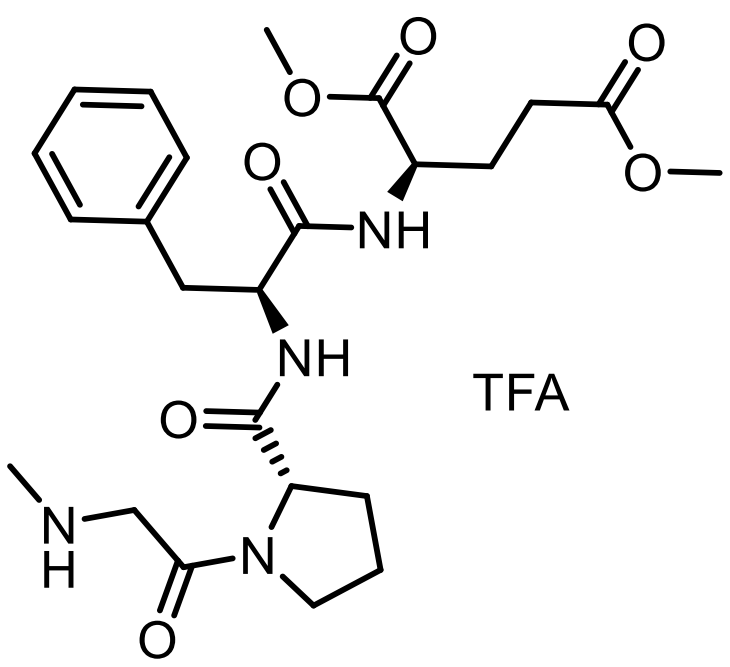

free amine
$C_{24}H_{34}N_4O_7$
MW: 490.5570

The compound was obtained as the TFA salt following general procedure **S3** (steps A and B).

^{1}H NMR (600 MHz, MeOD) δ 7.36 – 7.14 (m, 5H), 4.62 – 4.55 (m, 1H), 4.48 – 4.33 (m, 2H), 4.05 – 3.90 (m, 2H), 3.82 – 3.64 (m, 6H), 3.61 – 3.52 (m, 1H), 3.52 – 3.44 (m, 1H), 3.25 – 3.12 (m, 1H), 3.06 – 2.95 (m, 1H), 2.80 – 2.72 (m, 3H), 2.47 – 2.33 (m, 2H), 2.29 – 2.08 (m, 2H), 2.03 – 1.81 (m, 4H). *The NH protons were not observed.*

^{13}C NMR (151 MHz, MeOD) δ 175.0, 173.8, 173.4, 173.2, 165.5, 138.1, 130.4 (2C), 129.5 (2C), 127.8, 61.5, 56.0, 52.9, 52.3, 50.6, 49.6, 47.7, 38.3, 33.7, 30.8, 30.6, 27.7, 25.4.

HRMS (ESI$^+$): exact mass calculated for $[M+H]^+$ ($C_{24}H_{35}N_4O_7$) requires *m/z* 491.2500, found *m/z* 491.2498.

IR (neat) ν_{max}: 3221, 3101, 2822, 1730, 1640, 1513, 1211, 1328, 1209, 977, 777 cm^{-1}.

4. Additional scope entries

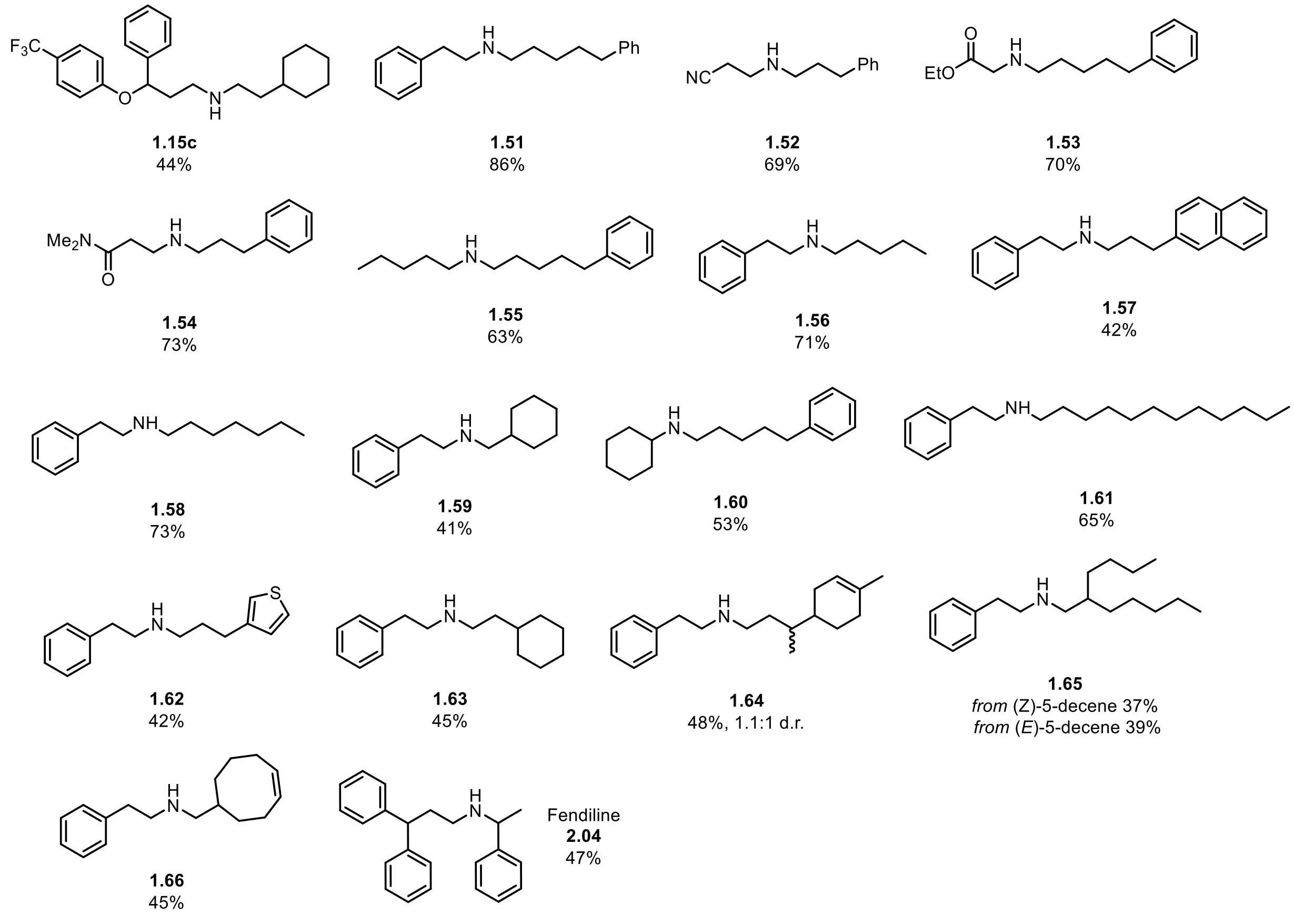

Supplementary Fig. S2. Additional scope entries.

5. General Procedures for preparation of products

5.1 General Procedure 1 for functionalization of alkenes/alkynes with ammonium salts

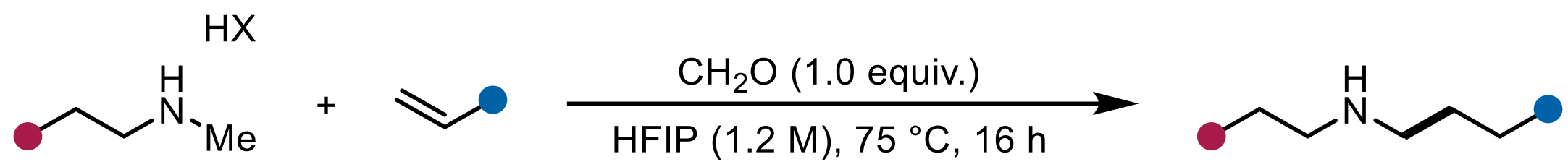

HX = TFA, HCl, acetic acid

At ambient temperature, the corresponding secondary amine (as a salt, 1.0 equiv.), paraformaldehyde (1.0 equiv.) and hexafluoroisopropanol (1.2 M) were added to an oven dried vial, followed by the addition of the corresponding alkene/alkyne (1.1 – 1.3 equiv.). The vial was sealed with a cap, and the reaction was vigorously stirred at 75 °C for 16 h, after which it was again allowed to cool to ambient temperature. After that, aqueous sodium hydroxide (3 M) was added until the pH reached 12. The product was extracted with dichloromethane (3 × 5 mL) and dried over magnesium sulfate. The dried solution was filtered, and the filtrate was concentrated under reduced pressure to afford the crude material, which was purified by flash column chromatography on silica gel with dichloromethane/DMA-mix (DMA-mix = solvent mixture of dichloromethane, methanol, aqueous solution of ammonium hydroxide in the ratio 9:1:0.1) to afford the analytically pure desired product.

5.2 General Procedure 2 for functionalization of alkenes/alkynes with ammonium salts

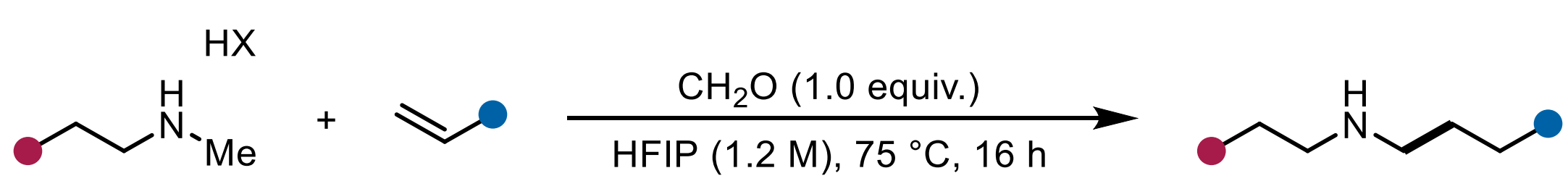

HX = TFA, HCl, acetic acid

At ambient temperature, the corresponding secondary amine (as a salt, 1.0 equiv.), paraformaldehyde (1.0 equiv.) and hexafluoroisopropanol (1.2 M) were added to an oven dried vial. The resulting solution was heated at 40 °C for 2 h, after which it was allowed to cool to ambient temperature, followed by the addition of the corresponding alkene/alkyne (1.1 –

1.3 equiv.). The vial was sealed with a cap, and the reaction was vigorously stirred at 75 °C for 16 h, after which it was again allowed to cool to ambient temperature. After that, aqueous sodium hydroxide (3 M) was added until the pH reached 12. The product was extracted with dichloromethane (3 × 5 mL) and dried over magnesium sulfate. The dried solution was filtered, and the filtrate was concentrated under reduced pressure to afford the crude material, which was purified by flash column chromatography on silica gel with dichloromethane/DMA-mix (DMA-mix = solvent mixture of dichloromethane, methanol, aqueous solution of ammonium hydroxide in the ratio 9:1:0.1) to afford the analytically pure desired product.

5.3 General Procedure 3 for functionalization of alkenes/alkynes with free amines

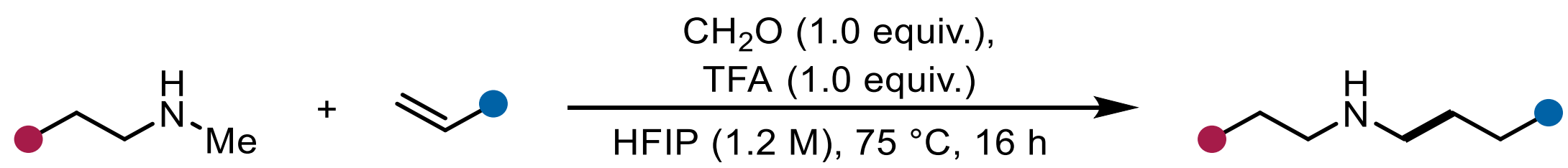

At ambient temperature, the corresponding secondary amine (1.0 equiv.), paraformaldehyde (1.0 equiv.) and hexafluoroisopropanol (1.2 M) were added to an oven dried vial, followed by the addition of the corresponding alkene/alkyne (1.1 – 1.3 equiv.). TFA (or acetic acid) was added then added dropwise to the suspension (1.0 equiv.). The vial was sealed, and the reaction was vigorously stirred at 75 °C for 16 h, after which it was allowed to cool to room temperature. After that, aqueous sodium hydroxide (3 M) was added until the pH reached 12. The product was extracted with dichloromethane (3 × 5 mL) and dried over magnesium sulfate. The filtrate was concentrated under reduced pressure to afford the crude material, which was purified by flash column chromatography on silica gel with dichloromethane/DMA-mix (DMA-mix = solvent mixture of dichloromethane, methanol, aqueous solution of ammonium hydroxide in the ratio 9:1:0.1) to afford the analytically pure desired product.

5.4 General Procedure 4 for functionalization of alkenes/alkynes with free amines

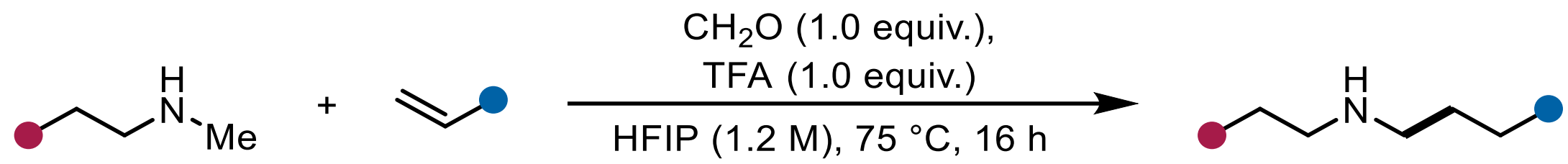

At ambient temperature, the corresponding secondary amine (1.0 equiv.), paraformaldehyde (1.0 equiv.) and hexafluoroisopropanol (1.2 M) were added to an oven dried vial. The suspension was cooled to 0 °C and TFA (or acetic acid) was added dropwise (1.0 equiv.). The resulting solution was heated at 40 °C for 2 h, cooled down to room temperature and then the corresponding alkene/alkyne (1.1 – 1.3 equiv.) was added to the reaction mixture. The vial was sealed, and the reaction was vigorously stirred at 75 °C for 16 h, after which it was allowed to cool to room temperature. After that, aqueous sodium hydroxide (3 M) was added until the pH reached 12. The product was extracted with dichloromethane (3 × 5 mL) and dried over magnesium sulfate. The filtrate was concentrated under reduced pressure to afford the crude material, which was purified by flash column chromatography on silica gel with dichloromethane/DMA-mix (DMA-mix = solvent mixture of dichloromethane, methanol, aqueous solution of ammonium hydroxide in the ratio 9:1:0.1) to afford the analytically pure desired product.

5.5 Comparison of General Procedures 1 & 2 and 3 & 4

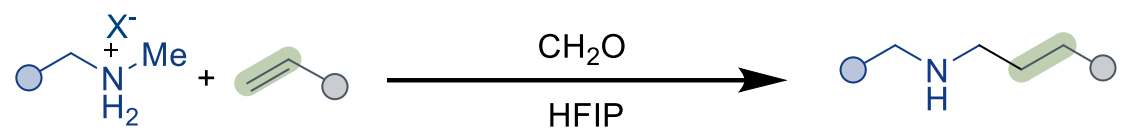

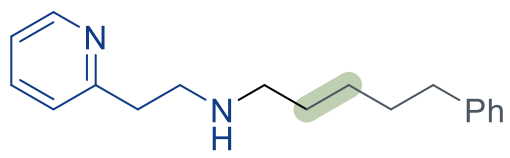

1.03
General procedure 1: 68% yield
General procedure 2: 72% yield

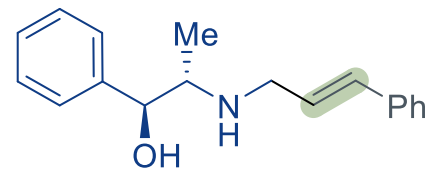

1.04
General procedure 3: 39% yield
General procedure 4: 42% yield

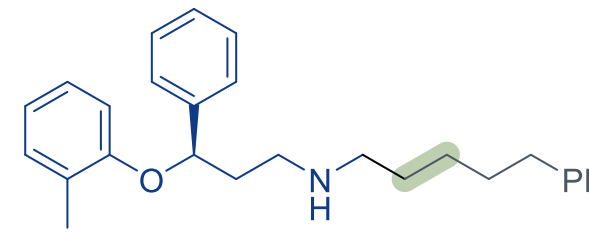

1.05
General procedure 1: 54% yield
General procedure 2: 58% yield

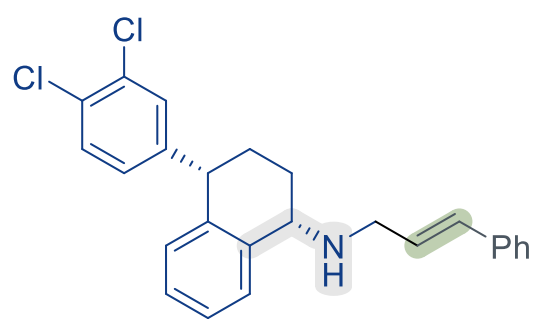

1.07
General procedure 1: 42% yield
General procedure 2: 47% yield

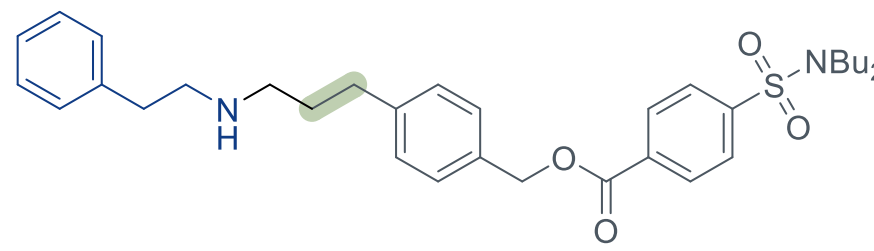

1.18
General procedure 3: **57%** yield
General procedure 4: 60% yield

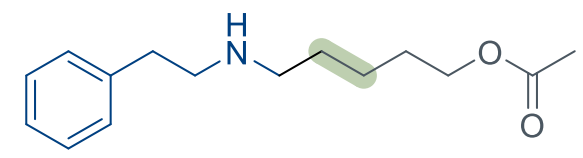

1.24
General procedure 3: **48%** yield
General procedure 4: 51% yield

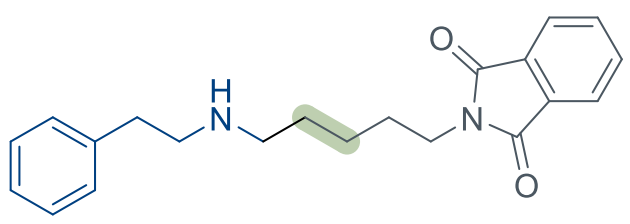

1.26
General procedure 3: **42%** yield
General procedure 4: 43% yield

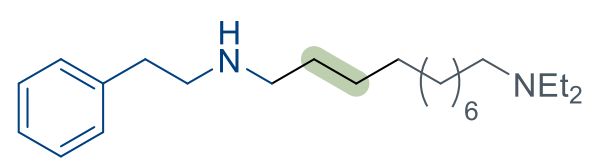

1.29
General procedure 3: **70%** yield
General procedure 4: 75% yield

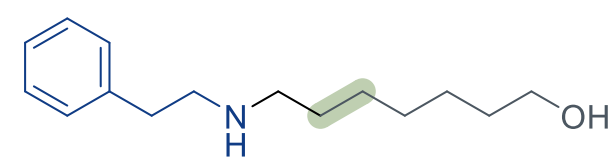

1.30
General procedure 3: **67%** yield
General procedure 4: **66%** yield

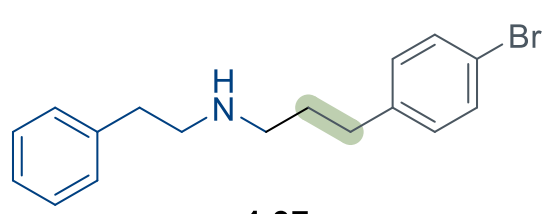

1.37
General procedure 3: **50%** yield
General procedure 4: 53% yield

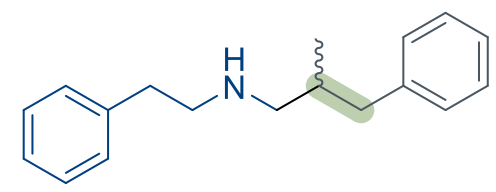

1.40
General procedure 3: **39%** yield
General procedure 4: 44% yield

Supplementary Fig. S3. Comparison between GP1&2 (direct addition) and GP3&4 (pre-stirring prior to alkene/alkyne addition).

5.6 Representative example of the reaction

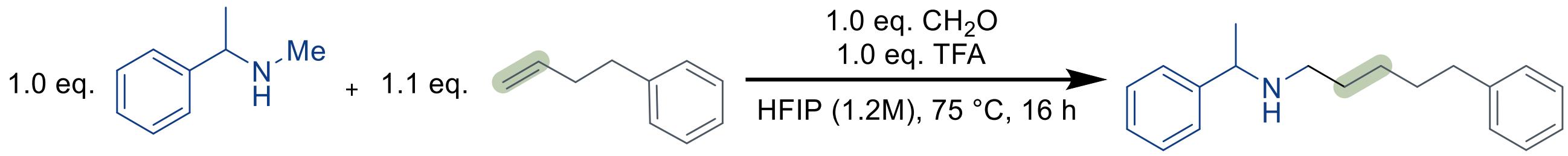

SM amine **21%** recovered

SM alkene **24%** recovered

1.13 67%

N-Methyl-1-phenylethan-1-amine (43.6 µL, 0.30 mmol, 1.00 equiv.), paraformaldehyde (9.0 mg, 0.30 mmol, 1.0 equiv.) and hexafluoroisopropanol (0.25 mL, 1.2 M) were added to an oven dried vial. The suspension was cooled to 0 °C and trifluoroacetic acid (22.3 µL, 0.30 mmol, 1.00 equiv.) was added dropwise. The resulting solution was heated at 40 °C for 2 h, cooled to room temperature and then 4-phenyl-1-butene (50.0 µL, 0.330 mmol, 1.10 equiv.) was added to the reaction mixture (**Supplementary Fig. S3a**). The vial was sealed, and the reaction was vigorously stirred at 75 °C for 16 h, after which it was allowed to cool to room temperature (**Supplementary Fig. S3b**). After that, aqueous sodium hydroxide (3 M) was added until the pH reached 12. The product was extracted with dichloromethane (3 × 5 mL) and dried over magnesium sulfate. The filtrate was concentrated under reduced pressure to afford the crude material (**Supplementary Fig. S3c**), which was purified by flash column chromatography on silica gel with dichloromethane/DMA-mix (DMA-mix = solvent mixture of dichloromethane, methanol, aqueous solution of ammonium hydroxide in the ratio 9:1:0.1) to afford the analytically pure desired product (53.9 mg, 0.202 mmol, 67%) as a yellow oil.

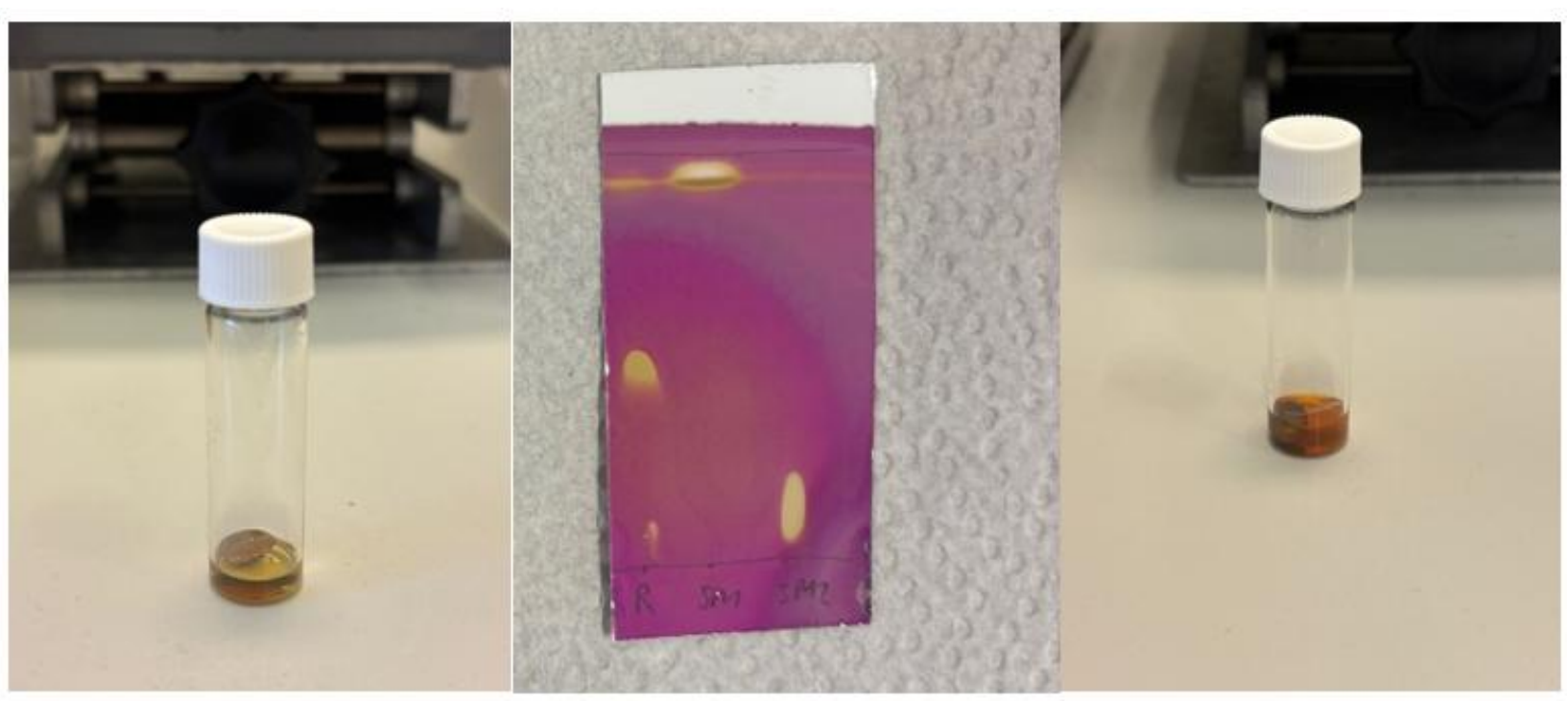

Supplementary Fig. S4 A. Reaction mixture before heating. **B.** TLC analysis of the crude mixture (eluent DCM:MeOH = 20:1, desired product Rf = 0.49). Left lane (R) = reaction mixture; middle lane (SM) = alkene; right lane (SM2) = amine. **C.** Reaction mixture after 16 h of heating.

5.7 Products: Analytical Data

3-(9,10-Ethanoanthracen-9(10*H*)-yl)-*N*-(3-cyclohexylpropyl)propan-1-amine (1.01)

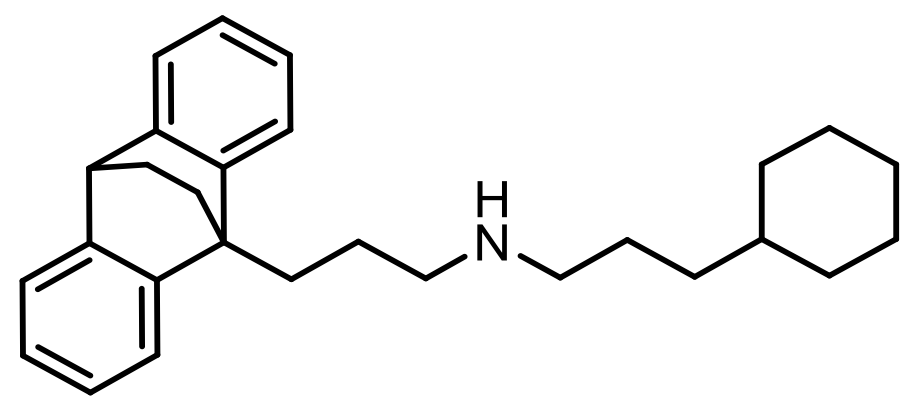

$C_{28}H_{37}N$
MW: 387.6110

Prepared following general procedure **2** using Maprotiline hydrochloride (94.2 mg, 0.300 mmol, 1.00 equiv.), cyclohexylethylene (45 µL, 0.330mmol, 1.1 equiv.) and paraformaldehyde (9.0 mg, 0.30 mmol, 1.0 equiv.). Purification by flash column chromatography (dichloromethane/DMA-mix 95:5 to 0:100) gave the title compound (81.4 mg, 0.210 mmol, 70%) as a pale-yellow oil.

^{1}H NMR (400 MHz, $CDCl_3$) δ 7.38 – 7.21 (m, 4H), 7.19 – 7.00 (m, 4H), 4.28 (t, J = 2.7 Hz, 1H), 2.98 (t, *J* = 7.4 Hz, 2H), 2.79 – 2.73 (m, 2H), 2.71 – 2.67 (m, 1H), 2.54 – 2.46 (m, 2H), 2.12 – 2.00 (m, 2H), 1.89 – 1.80 (m, 2H), 1.80 – 1.56 (m, 8H), 1.33 – 1.11 (m, 9H).

^{13}C NMR (101 MHz, $CDCl_3$) δ 145.5, 145.1, 125.4, 125.4, 123.5, 121.5, 50.9, 50.3, 44.9, 44.7, 37.7, 35.2, 33.5, 33.4, 29.8, 29.0, 27.8, 26.8, 26.5, 25.1.

HRMS (ESI$^+$): exact mass calculated for $[M+H]^+$ ($C_{28}H_{38}N$) requires *m/z* 388.2999, found *m/z* 388.2995.

IR (neat) ν_{max}: 3018, 2920, 2848, 1739, 1448, 1261, 1138, 1033, 937, 889 cm^{-1}.

(*S*)-3-(Naphthalen-1-yloxy)-*N*-(3-phenylpropyl)-3-(thiophen-2-yl)propan-1-amine (1.02)

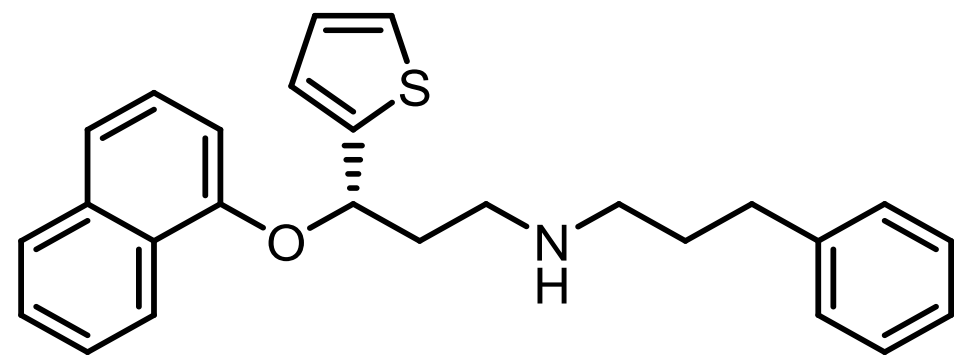

$C_{26}H_{27}NOS$
MW: 401.5680

Prepared following general procedure **2** using Duloxetine hydrochloride (66.8 mg, 0.200 mmol, 1.00 equiv.), styrene (25.3 mg, 0.220 mmol, 1.10 equiv.), paraformaldehyde (6.0 mg, 0.20 mmol, 1.0 equiv.), trifluoroacetic acid (15.0 µL, 0.20 mmol, 1.0 equiv.) and HFIP (0.17 mL). Purification by flash column chromatography (dichloromethane/DMA-mix 95:5 to 0:100) gave the title compound (33.7 mg, 83.9 µmol, 42%) as a yellow oil.

^{1}H NMR (600 MHz, CD_2Cl_2) δ 8.37 – 8.31 (m, 1H), 7.83 – 7.75 (m, 1H), 7.52 – 7.45 (m, 2H), 7.40 (d, *J =* 8.2 Hz, 1H), 7.29 (d, *J =* 7.9 Hz, 1H), 7.25 – 7.21 (m, 3H), 7.18 – 7.11 (m, 3H), 7.11 – 7.07 (m, 1H), 6.97 – 6.92 (m, 1H), 6.90 (d, *J =* 7.7 Hz, 1H), 5.86 (dd, *J =* 7.7, 5.3 Hz, 1H), 2.92 – 2.81 (m, 2H), 2.69 – 2.59 (m, 4H), 2.50 – 2.41 (m, 1H), 2.26 – 2.16 (m, 1H), 1.82 – 1.74 (m, 2H). *The NH proton was not observed.*

^{13}C NMR (151 MHz, CD_2Cl_2) δ 153.9, 145.9, 142.9, 135.2, 128.9, 128.8 (2C), 128.0 (2C), 127.2, 126.9, 126.7, 126.27, 126.25, 125.8, 125.5, 125.3, 122.6, 121.1, 107.7, 75.2, 49.7, 46.4, 39.4, 34.0, 32.0.

HRMS (ESI$^+$): exact mass calculated for $[M+H]^+$ ($C_{26}H_{27}NOS$) requires *m/z* 402.1886, found *m/z* 402.1890.

IR (neat) ν_{max}: 3253, 3065, 2954, 2873, 1599, 1511, 1473, 1305, 1211, 1053 cm^{-1}.

5-Phenyl-*N*-(2-(pyridin-2-yl)ethyl)pentan-1-amine (1.03)

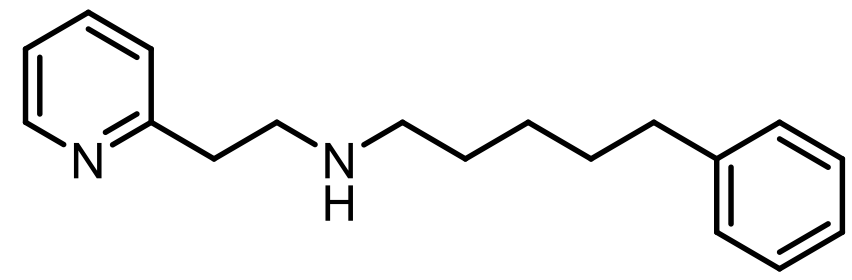

$C_{18}H_{24}N_2$
MW: 268.4040

Prepared following general procedure **2** using Betahistine dihydrochloride (62.7 mg, 0.300 mmol, 1.00 equiv.), 4-phenyl-1-butene (58.6 µL, 0.390 mmol, 1.30 equiv.) and paraformaldehyde (9.0 mg, 0.30 mmol, 1.0 equiv.). Purification by flash column chromatography (dichloromethane/DMA-mix 95:5 to 0:100) gave the title compound (58.0 mg, 0.220 mmol, 72%) as a pale-yellow oil.

^{1}H NMR (600 MHz, $CDCl_3$) δ 8.51 (ddd, *J* = 4.8, 1.6, 0.8 Hz, 1H), 7.58 (td, *J* = 7.6, 1.8 Hz, 1H), 7.28 – 7.23 (m, 2H), 7.18 – 7.14 (m, 4H), 7.11 (ddd, *J* = 7.4, 4.9, 1.0 Hz, 1H), 3.05 – 2.97 (m, 4H), 2.66 – 2.63 (m, 2H), 2.61 – 2.58 (m, 2H), 2.24 (br s, 1H), 1.62 (dt, *J* = 15.5, 7.7 Hz, 2H), 1.55 – 1.50 (m, 2H), 1.38 – 1.31 (m, 2H).

^{13}C NMR (151 MHz, $CDCl_3$) 160.3, 149.3, 142.7, 136.5, 128.4 (2C), 128.3 (2C), 125.7, 123.3, 121.3, 49.7, 49.4, 38.2, 35.9, 31.4, 29.8, 27.0.

HRMS (ESI$^+$): exact mass calculated for $[M+H]^+$ ($C_{18}H_{25}N_2$) requires *m/z* 269.2012, found *m/z* 269.2014.

IR (neat) ν_{max}: 3350, 3313, 3214, 3000, 2967, 2872, 1388, 1028, 892 cm^{-1}.

(1*S*,2*S*)-2-(Cinnamylamino)-1-phenylpropan-1-ol (1.04)

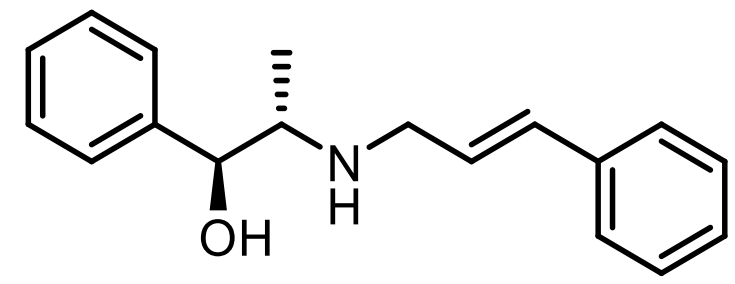

$C_{18}H_{21}NO$
MW: 267.3720

Prepared following general procedure **3** using (1*S*,2*S*)-Pseudoephedrine (74.4 mg, 0.300 mmol, 1.00 equiv.), ethynylbenzene (36.2 µL, 0.330 mmol, 1.10 equiv.), trifluoroacetic acid (22.3 µL, 0.300 mmol, 1.00 equiv.) and paraformaldehyde (9.0 mg, 0.30 mmol, 1.0 equiv.).

The crude mixture was then dissolved in absolute ethanol (1.5 mL), followed by addition of hydrazine monohydrate (45 µL, 0.90 mmol, 3.0 equiv.). The solution was stirred for 3 hours at 80 °C and then cooled down to ambient temperature. The reaction mixture was concentrated under reduced pressure to afford the crude product which was directly purified by flash column chromatography (dichloromethane/DMA-mix 95:5 to 0:100) to gave the title compound (33.7 mg, 0.126 mmol, 42%) as a yellow oil.

^{1}H NMR (400 MHz, $CDCl_3$) δ 7.44 – 7.27 (m, 9H), 7.25 – 7.16 (m, 1H), 6.66 – 6.50 (m, 1H), 6.31 (dt, *J* = 15.9, 6.5 Hz, 1H), 4.35 (d, *J* = 8.5 Hz, 1H), 3.67 (ddd, *J* = 13.8, 6.3, 1.5 Hz, 1H), 3.45 (ddd, *J* = 13.8, 6.7, 1.4 Hz, 1H), 3.11 – 2.83 (m, 1H), 1.25 (s, 1H), 1.05 (d, *J* = 6.5 Hz, 3H). *The NH proton was not observed.*

^{13}C NMR (101 MHz, $CDCl_3$) δ 141.7, 136.7, 133.3, 128.8 (2C), 128.6 (2C), 128.1, 128.0 (2C), 127.2, 126.6 (2C), 125.9, 59.5, 49.0, 29.9, 15.9.

HRMS (ESI$^+$): exact mass calculated for $[M+H]^+$ ($C_{18}H_{22}NO$) requires *m/z* 268.1693, found *m/z* 268.1696.

IR (neat) ν_{max}: 2963, 2872, 2352, 1622, 1577, 1562, 1341, 1222, 1088, 1023, 956, 834 cm^{-1}.

(*R*)-5-Phenyl-*N*-(3-phenyl-3-(*o*-tolyloxy)propyl)pentan-1-amine (1.05)

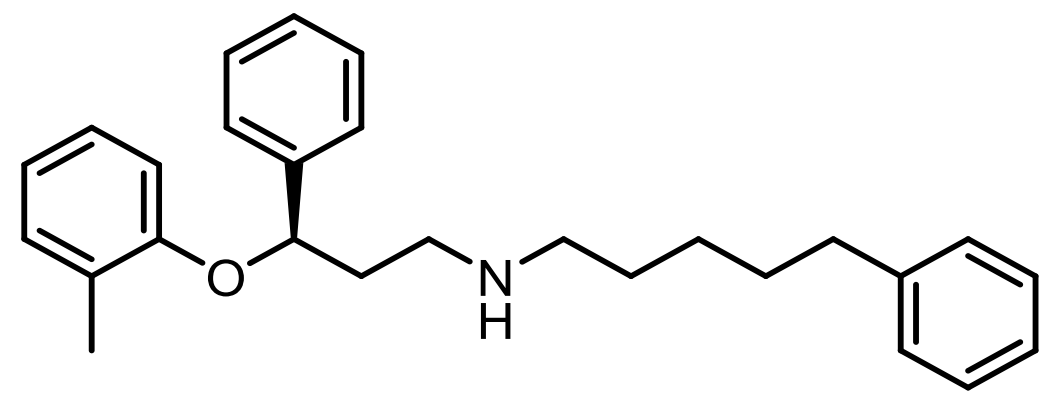

$C_{27}H_{33}NO$
MW: 387.2562

Prepared following general procedure **2** using Atomoxetine hydrochloride (38.3 mg, 0.100 mmol, 1.00 equiv.), 4-phenyl-1-butene (16.5 µL, 0.110 mmol, 1.10 equiv.) and paraformaldehyde (3.0 mg, 0.10 mmol, 1.0 equiv.). Purification by flash column chromatography (dichloromethane/DMA-mix 95:5 to 0:100) gave the title compound (22.5 mg, 58.0 µmol, 58%) as a pale-yellow oil.

^{1}H NMR (400 MHz, CDCl$_3$) δ 7.35 – 7.28 (m, 4H), 7.28 – 7.21 (m, 3H), 7.18 – 7.15 (m, 3H) 7.11 (d, *J* = 7.2 Hz, 1H), 6.98 – 6.93 (m, 1H), 6.77 (t, *J* = 7.3 Hz, 1H), 6.60 (d, *J* = 8.1 Hz, 1H), 5.26 (dd, *J* = 8.1, 4.5 Hz, 1H), 2.83 (t, *J* = 7.0 Hz, 2H), 2.60 (dt, *J* = 10.4, 7.7 Hz, 4H), 2.31 (s, 3H), 2.27 – 2.03 (m, 3H), 1.65 – 1.57 (m, 2H), 1.57 – 1.48 (m, 2H), 1.38 – 1.29 (m, 2H).

^{13}C NMR (151 MHz, CDCl$_3$) δ 156.1, 142.7, 142.0, 130.8 (2C), 128.7(2C), 128.5, 128.4, 127.6 (2C), 127.1 (2C), 126.7, 125.9, 125.8, 120.4, 113.0, 78.3, 49.9, 46.4, 38.7, 36.0, 31.4, 29.7, 27.1, 16.7.

HRMS (ESI$^+$): exact mass calculated for $[M+H]^+$ ($C_{27}H_{34}NO$) requires *m/z* 388.2635, found *m/z* 388.2633.

IR (neat) ν_{max}: 3323, 3303, 3011, 2955, 2880, 2810, 1420, 1247, 1050, 820 cm^{-1}.

(*E*)-*N*-(3-(2-methoxyphenoxy)-3-phenylpropyl)-3-(4-(trifluoromethyl)phenyl)prop-2-en-1-amine (1.06)

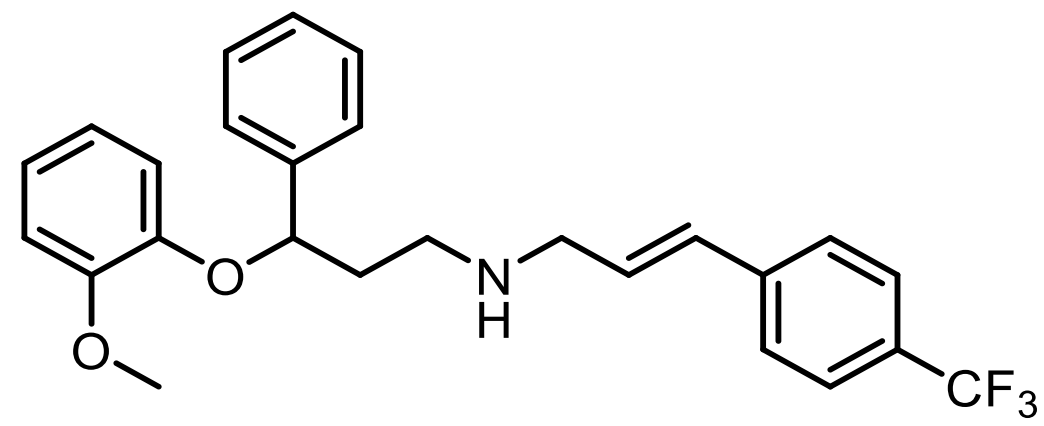

$C_{26}H_{26}F_3NO_2$
MW: 441,4942

Prepared following general procedure **4** using Nisoxetine (74.4 mg, 0.300 mmol, 1.00 equiv.), 4-ethynyl-trifluorotoluene (53.8 µL, 0.330 mmol, 1.10 equiv.), trifluoroacetic acid (22.3 µL, 0.300 mmol, 1.00 equiv.) and paraformaldehyde (9.0 mg, 0.30 mmol, 1.0 equiv.). Purification by flash column chromatography (dichloromethane/DMA-mix 95:5 to 0:100) gave the title compound (60.9 mg, 0.138 mmol, 46%) as a yellow oil.

^{1}H NMR (400 MHz, CDCl$_3$) 7.55 (d, *J* = 8.2 Hz, 2H), 7.42 (d, *J* = 8.2 Hz, 2H), 7.39 – 7.24 (m, 5H), 6.85 – 6.73 (m, 2H), 6.70 (ddd, *J* = 8.0, 5.9, 3.0 Hz, 1H), 6.65 (dd, *J* = 7.6, 1.3 Hz, 1H), 6.59 (d, *J* = 16.0 Hz, 1H), 6.43 (dt, *J* = 15.9, 6.2 Hz, 1H), 5.24 (dd, *J* = 8.5, 4.4 Hz, 1H), 3.82 (s, 3H), 3.51 (dd, *J* = 6.1, 1.4 Hz, 2H), 3.00 – 2.86 (m, 2H), 2.35 – 2.27 (m, 1H), 2.19 – 2.07 (m, 1H), 2.02 (br s, 1H).

^{13}C NMR (101 MHz, CDCl$_3$) δ 150.0, 147.5, 141.7, 140.5, 130.7 (3C), 130.3, 130.2 (2C), 129.6, 129.3, 128.8 (2C), 128.6, 127.72, 126.6 (2C), 126.0, 125.72, 125.67 (q, *J* = 3.8 Hz), 81.2, 56.1, 51.6, 46.4, 38.1. *Some ^{19}F coupling with the carbons were not observed.*

^{19}F NMR (376 MHz, CDCl$_3$) δ -62.49.

HRMS (ESI$^+$): exact mass calculated for $[M+H]^+$ ($C_{26}H_{27}F_3NO_2$) requires *m/z* 442.1988, found *m/z* 442.1987.

IR (neat) ν_{max}: 3029, 2921, 2847, 1730, 1671, 1593, 1502, 1365, 1252, 1067, 1016, 745 cm^{-1}.

(1*S*,4*S*)-*N*-cinnamyl-4-(3,4-dichlorophenyl)-1,2,3,4-tetrahydronaphthalen-1-amine (1.07)

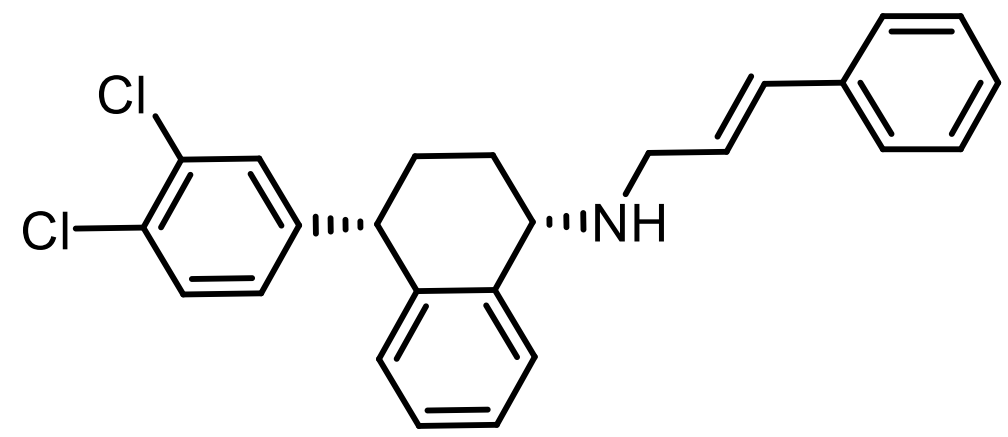

$C_{25}H_{23}Cl_2N$
MW: 408.3660

Prepared following general procedure **2** using Sertraline hydrochloride (103.0 mg, 0.300 mmol, 1.00 equiv.), ethynylbenzene (36.2 µL, 0.330 mmol, 1.10 equiv.) and paraformaldehyde (9.0 mg, 0.30 mmol, 1.0 equiv.). Purification by flash column chromatography (dichloromethane/DMA-mix 95:5 to 0:100) gave the title compound (57.6 mg, 0.141 mmol, 47%) as a pale-yellow oil.

^{1}H NMR (400 MHz, CDCl$_3$) δ 7.40 (dt, *J* = 8.3, 1.1 Hz, 3H), 7.37 – 7.30 (m, 3H), 7.28 (d, *J* = 2.1 Hz, 1H), 7.25 – 7.19 (m, 2H), 7.12 (td, *J* = 7.5, 1.5 Hz, 1H), 6.99 (dd, *J* = 8.3, 2.1 Hz, 1H), 6.81 (dt, *J* = 7.8, 1.2 Hz, 1H), 6.61 (dt, *J* = 15.9, 1.5 Hz, 1H), 6.38 (ddd, *J* = 15.8, 6.6, 5.9 Hz, 1H), 4.00 (dd, *J* = 9.3, 5.6 Hz, 1H), 3.92 (t, *J* = 4.3 Hz, 1H), 3.58 (dd, *J* = 5.9, 1.6 Hz, 1H), 3.54 (dd, *J* = 6.6, 1.5 Hz, 1H), 2.20 – 2.07 (m, 1H), 2.07 – 1.96 (m, 2H), 1.94 – 1.81 (m, 1H), 1.36 (br s, 1H).
^{13}C NMR (101 MHz, CDCl$_3$) δ 147.7, 139.9, 138.9, 137.3, 132.4, 131.4, 130.9, 130.5 (2C), 130.2, 130.0, 129.4, 129.1, 128.7, 128.4, 127.5, 127.4, 126.8 (2C), 126.4, 54.9, 49.8, 45.4, 28.7, 26.7.
HRMS (ESI$^+$): exact mass calculated for $[M+H]^+$ ($C_{25}H_{24}{}^{35}Cl_2N$) requires *m/z* 408.1280, found *m/z* 408.1277.
IR (neat) ν_{max}: 3024, 292, 2850, 1739, 1677, 1487, 1447, 1217, 1156, 1071, 966, 877 cm^{-1}.

N-((5-(2-Fluorophenyl)-1-(pyridin-3-ylsulfonyl)-1H-pyrrol-3-yl)methyl)-3-phenylpropan-1-amine (1.08)

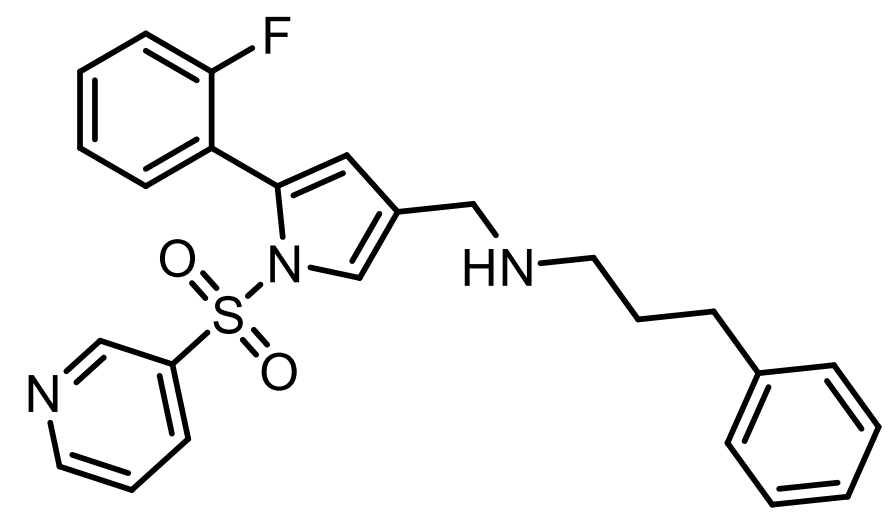

$C_{25}H_{24}FN_3O_2S$
MW: 449.5444

Prepared following general procedure **1** using Vonoprazan fumarate (138.0 mg, 0.300 mmol, 1.00 equiv.), styrene (51.7 µL, 0.450 mmol, 1.50 equiv.), and paraformaldehyde (9.0 mg, 0.30 mmol, 1.0 equiv.). The reaction was performed using a solvent system HFIP:DCE = 1:1 (0.6 M). The reaction time was shortened to 2 h. Purification by flash column chromatography (dichloromethane/DMA-mix 95:5 to 0:100) gave the title compound (59.3 mg, 0.132 mmol, 44%) as a pale-yellow oil.

^{1}H NMR (600 MHz, $CDCl_3$) δ 8.77 (dd, *J* = 4.9, 1.6 Hz, 1H), 8.61 (d, *J* = 2.4 Hz, 1H), 7.86 – 7.68 (m, 1H), 7.51 (d, *J* = 1.9 Hz, 1H), 7.46 – 7.39 (m, 1H), 7.38 – 7.32 (m, 1H), 7.31 – 7.24 (m, 3H), 7.23 – 7.11 (m, 5H), 7.04 (t, *J* = 8.9 Hz, 1H), 6.40 (d, *J* = 1.9 Hz, 1H), 3.80 (s, 2H), 2.85 – 2.72 (m, 2H), 2.69 (t, *J* = 7.5 Hz, 2H), 2.19 (br s, 1H), 2.07 – 1.96 (m, 2H).

^{13}C NMR (151 MHz, $CDCl_3$) δ 153.3, 145.0, 134.6, 128.5, 128.42, 128.37 (2C), 127.5 (2C), 126.6, 126.4, 126.1, 126.0, 125.7, 125.3, 124.9, 124.8, 124.7, 122.1, 120.7, 107.0, 48.1, 38.5, 36.2, 22.6.

^{19}F coupling with the carbons was not observed.

^{19}F NMR (659 MHz, $CDCl_3$) δ –111.16.

HRMS (ESI$^+$): exact mass calculated for $[M+H]^+$ ($C_{25}H_{25}FN_3O_2S$) requires *m/z* 450.1646, found *m/z* 450.1652.

IR (neat) ν_{max}: 2920, 2848, 2812, 1675, 1448, 1365, 1311, 1217, 1202, 1138, 1071, 1033, 1012, 969, 889, 798 cm^{-1}.

N-(3-(4-Chlorophenyl)-3-(pyridin-2-yl)propyl)-5-phenylpentan-1-amine (1.09)

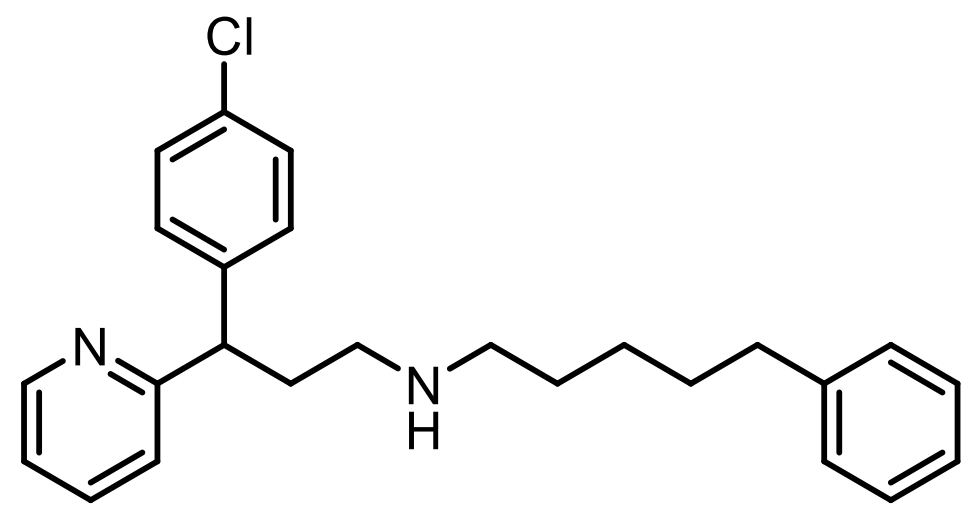

$C_{25}H_{29}ClN_2$
MW: 392.9710

Prepared following general procedure **4** using **S1.09a** (52.2 mg, 0.200 mmol, 1.00 equiv.), 4-phenyl-1-butene (50.0 µL, 0.330 mmol, 1.10 equiv.) trifluoroacetic acid (15.0 µL, 0.200 mmol, 1.00 equiv.) and paraformaldehyde (6.0 mg, 0.20 mmol, 1.0 equiv.). Purification by flash column chromatography (dichloromethane/DMA-mix 95:5 to 0:100) gave the title compound (39.2 mg, 0.100 mmol, 50%) as a brown oil.

^{1}H NMR (400 MHz, CDCl$_3$) δ 8.51 (br s, 1H), 8.49 – 8.44 (m, 1H), 7.54 (td, *J* = 7.7, 1.8 Hz, 1H), 7.30 – 7.02 (m, 11H), 4.16 (dd, *J* = 8.4, 6.5 Hz, 1H), 2.88 – 2.71 (m, 4H), 2.69 – 2.55 (m, 1H), 2.54 (t, *J* = 7.6 Hz, 2H), 2.49 – 2.33 (m, 1H), 1.71 – 1.61 (m, 2H), 1.59 – 1.49 (m, 2H), 1.34 – 1.24 (m, 2H).

^{13}C NMR (126 MHz, CDCl$_3$) δ 168.4, 161.4, 149.3, 142.1, 141.0, 137.1, 133.0, 129.4 (2C), 129.1 (2C), 128.5 (2C), 126.0 (2C), 123.6, 122.1, 50.3, 47.4, 45.8, 35.8, 31.0, 30.9, 26.5, 26.1.

HRMS (ESI$^+$): exact mass calculated for $[M+H]^+$ ($C_{26}H_{30}{}^{35}ClN_2$) requires *m/z* 393.2092, found *m/z* 393.2090.

IR (neat) ν_{max}: 3339, 3264, 3051, 2973, 2865, 1611, 1588, 1583, 1499, 1379, 1211, 1052, 964 cm^{-1}.

1-(4-Fluorophenyl)-1-(3-((5-phenylpentyl)amino)propyl)-1,3-dihydroisobenzofuran-5-carbonitrile (1.10)

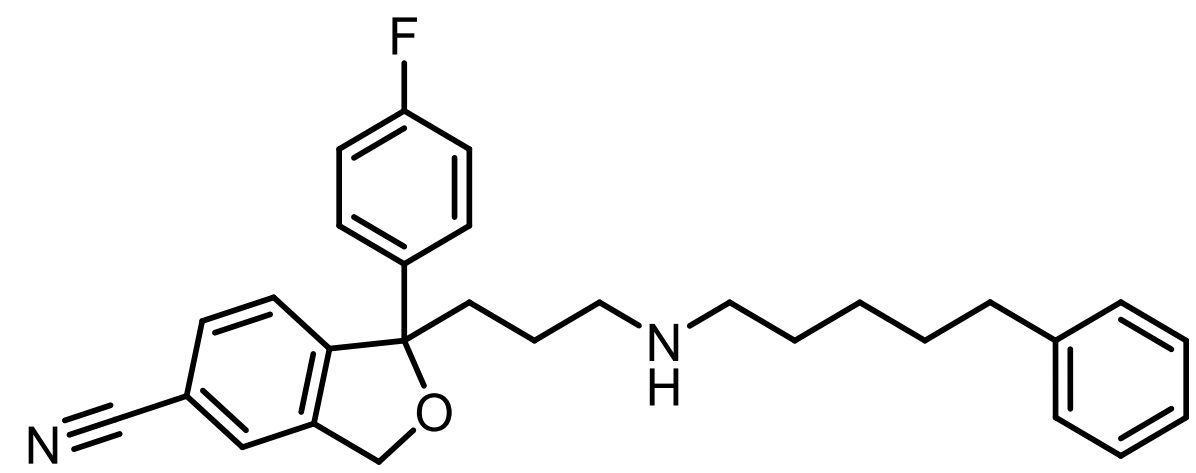

$C_{29}H_{31}FN_2O$
MW: 442.5784

Prepared following general procedure **4** using **S1.10a** (93.1 mg, 0.300 mmol, 1.00 equiv.), 4-phenyl-1-butene (58.6 µL, 0.330 mmol, 1.10 equiv.), trifluoroacetic acid (22.3 µL, 0.300 mmol, 1.00 equiv.) and paraformaldehyde (9.01 mg, 0.300 mmol, 1.00 equiv.). Purification by flash column chromatography (dichloromethane/DMA-mix 95:5 to 0:100) gave the title compound (94.3 mg, 0.213 mmol, 71%) as a colourless oil.

^{1}H NMR (600 MHz, $CDCl_3$) δ 7.61 – 7.56 (m, 1H), 7.49 (s, 1H), 7.43 – 7.34 (m, 3H), 7.27 (dd, *J* = 4.7, 3.0 Hz, 2H), 7.20 – 7.12 (m, 3H), 7.03 – 6.96 (m, 2H), 5.16 (dd, *J* = 29.2, 12.9 Hz, 2H), 2.64 – 2.55 (m, 4H), 2.54 – 2.49 (m, 2H), 2.27 – 2.18 (m, 1H), 2.18 – 2.08 (m, 1H), 1.66 – 1.56 (m, 2H), 1.55 – 1.45 (m, 4H), 1.39 – 1.28 (m, 3H).

^{13}C NMR (151 MHz, $CDCl_3$) δ 162.2 (d, *J* = 246.4 Hz), 149.5, 142.7, 140.5, 139.7 (d, *J* = 3.1 Hz), 132.0, 128.5 (2C), 128.4 (2C), 126.9 (d, *J* = 7.0 Hz, 2C), 125.8, 125.3, 122.9, 118.8, 115.5 (d, *J* = 21.4 Hz, 2C), 111.9, 91.3, 71.4, 49.9 (2C), 39.2, 36.0, 31.5, 29.9, 27.1, 24.7.

^{19}F NMR (377 MHz, $CDCl_3$) δ –115.34.

HRMS (ESI$^+$): exact mass calculated for $[M+H]^+$ ($C_{29}H_{32}FN_2O$) requires *m/z* 443.2493, found *m/z* 443.2489.

IR (neat) ν_{max}: 3061, 3025, 2828, 2854, 2228, 1670, 1600, 1506, 1224, 833, 699, 540 cm^{-1}.

***N*-(3-((1-Benzyl-1*H*-indazol-3-yl)oxy)propyl)-5-phenylpentan-1-amine (1.11)**

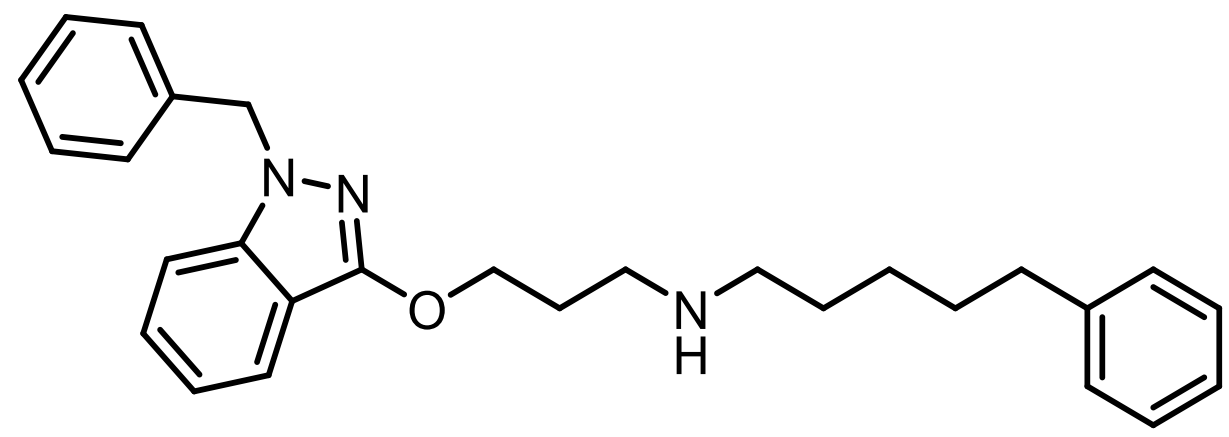

$C_{28}H_{33}N_3O$
MW: 427.5920

Prepared following general procedure **4** using **S1.11a** (59.1 mg, 0.200 mmol, 1.00 equiv.), 4-phenyl-1-butene (33.0 µL, 0.220 mmol, 1.1 equiv.), trifluoroacetic acid (15.0 µL, 0.200 mmol, 1.00 equiv.) and paraformaldehyde (6.0 mg, 0.30 mmol, 1.0 equiv.). Purification by flash column chromatography (dichloromethane/DMA-mix 95:5 to 0:100) gave the title compound (62.4 mg, 0.140 mmol, 71%) as a pale-yellow oil.

^{1}H NMR (600 MHz, CDCl$_3$) δ 7.65 (dd, *J* = 8.1, 1.1 Hz, 1H), 7.31 (ddd, *J* = 8.2, 6.9, 1.1 Hz, 1H), 7.29 – 7.25 (m, 2H), 7.24 – 7.11 (m, 9H), 7.04 (ddd, *J* = 7.8, 6.8, 0.8 Hz, 1H) 5.37 (s, 2H), 4.48 (t, *J* = 6.0 Hz, 2H), 2.98 (t, *J* = 7.1 Hz, 2H), 2.73 – 2.69 (m, 2H), 2.57 (t, *J* = 7.7 Hz, 2H), 2.24 (app p, *J* = 6.6 Hz, 2H), 1.73 – 1.61 (m, 2H), 1.63 – 1.57 (m, 2H), 1.35 – 1.30 (m, 2H). *The NH proton was not observed.*

^{13}C NMR (151 MHz, CDCl$_3$) 156.2, 142.5, 141.8, 137.5, 128.8 (2C), 128.5 (2C), 128.4 (2C), 127.8 (2C), 127.7, 127.0, 125.9, 120.2, 119.5, 113.0, 67.0, 52.4, 49.1, 46.3, 35.9, 31.2, 31.0, 28.2, 26.8, 26.7.

HRMS (ESI$^+$): exact mass calculated for [M+H]$^+$ ($C_{28}H_{34}N_3O$) requires *m/z m/z* 428.2696, found *m/z* 428.2694.

IR (neat) ν_{max}: 2985, 2846, 1700, 1562, 1432, 1300, 1211, 1067, 943, 867, 721 cm^{-1}.

2,6-Dimethyl-*N*-(5-phenylpentyl)aniline (1.12)

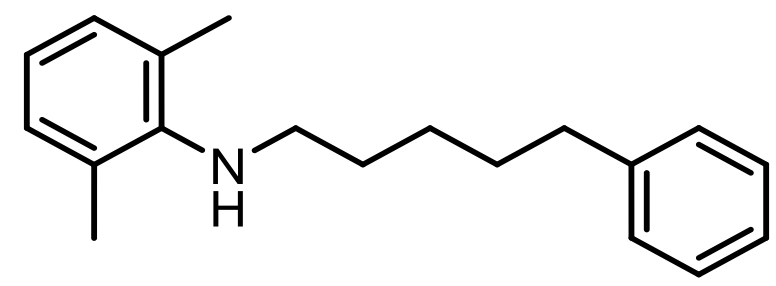

$C_{19}H_{25}N$
MW: 267.4160

Prepared following the general **4** procedure using ethyl *N*,2,6-trimethylaniline (43.6 µL, 0.300 mmol, 1.00 equiv.), 4-phenyl-1-butene (150.1 µL, 0.900 mmol, 3.00 equiv.) and paraformaldehyde (9.0 mg, 0.30 mmol, 1.00 equiv.). The reaction was conducted at 25 °C. Purification by flash column chromatography (dichloromethane/DMA-mix 95:5 to 0:100) gave the title compound (42.5 mg, 0.159 mmol, 53%) as a yellow oil.

^{1}H NMR (600 MHz, CDCl$_3$) δ 7.31 – 7.26 (m, 2H), 7.20 – 7.15 (m, 3H), 6.98 (d, *J* = 7.5 Hz, 2H), 6.80 (t, *J* = 7.5 Hz, 1H), 2.96 (t, *J* = 7.2 Hz, 2H), 2.62 (t, *J* = 7.6 Hz, 2H), 2.26 (s, 6H), 1.69 – 1.64 (m, 2H), 1.64 – 1.58 (m, 2H), 1.55 (b s, 1H), 1.46 – 1.39 (m, 2H).

^{13}C NMR (151 MHz, CDCl$_3$) δ 146.5, 142.7, 129.3, 128.9 (2C), 128.5 (2C), 128.4 (2C), 125.8, 121.7 (2C), 48.7, 36.1, 31.5, 31.2, 27.0, 18.7 (2C).

HRMS (ESI$^+$): exact mass calculated for $[M+H]^+$ ($C_{16}H_{26}N$) requires *m/z* 268.2060, found *m/z* 268.2059.

IR (neat) ν_{max}: 3286, 3251, 3147, 2986, 2857, 1521, 1346, 1057, 981, 860, 643 cm^{-1}.

5-Phenyl-*N*-(1-phenylethyl)pentan-1-amine (1.13)

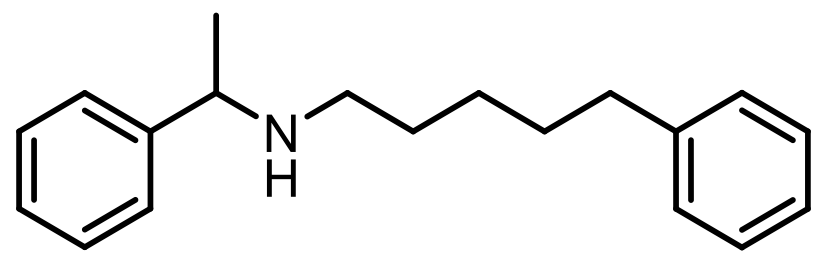

$C_{19}H_{25}N$
MW: 267.4160

Prepared following general procedure **4** using *N*-methyl-1-phenylethan-1-amine (43.6 µL, 0.30 mmol, 1.00 equiv.), 4-phenyl-1-butene (50.0 µL, 0.330 mmol, 1.10 equiv.), trifluoroacetic acid (22.3 µL, 0.30 mmol, 1.00 equiv.) and paraformaldehyde (9.0 mg, 0.30 mmol, 1.0 equiv.). Purification by flash column chromatography (dichloromethane/DMA-mix 95:5 to 0:100) gave the title compound (53.9 mg, 0.202 mmol, 67%) as a yellow oil.

Following general procedure **4** using (*S*)-*N*-methyl-1-phenylethan-1-amine, the title compound was isolated with 64% yield.

^{1}H NMR (700 MHz, $CDCl_3$) δ 7.33 – 7.28 (m, 4H), 7.27 – 7.23 (m, 3H), 7.17 – 7.12 (m, 3H), 3.76 (q, *J* = 6.0 Hz, 1H), 2.60 – 2.56 (m, 2H), 2.53 – 2.47 (m, 1H), 2.47 – 2.38 (m, 1H), 1.60 – 1.47 (m, 5H), 1.40 (d, *J* = 5.8 Hz, 3H), 1.34 – 1.28 (m, 2H).

^{13}C NMR (176 MHz, $CDCl_3$) δ 142.8 (2C), 128.7 (2C), 128.5 (2C), 128.4, 127.2, 126.8 (2C), 125.8 (2C), 58.6, 47.7, 36.0, 31.4, 30.0, 27.1, 24.1.

HRMS (ESI$^+$): exact mass calculated for $[M+H]^+$ ($C_{19}H_{26}N$) requires *m/z* 268.2060, found *m/z* 268.2062.

IR (neat) ν_{max}: 3350, 3301, 3022, 2931, 2883, 2825, 1488, 1455, 1370, 1299, 1250, 1193, 1023, 790 cm^{-1}.

Chiral HPLC: enantiomeric ratio (er) 99:01, see below for detailed conditions.

Method Description:
Column:Lux-3 Cellulose-3
250x4,6mm Particle Size 3 micrometer
Solvent System: 50%(n-Heptan/IPA/DEA 93:7:0,1):50%(n-Heptan+0,1%IPA)
Flow=1 ml/min T=25°C

Chromatogram

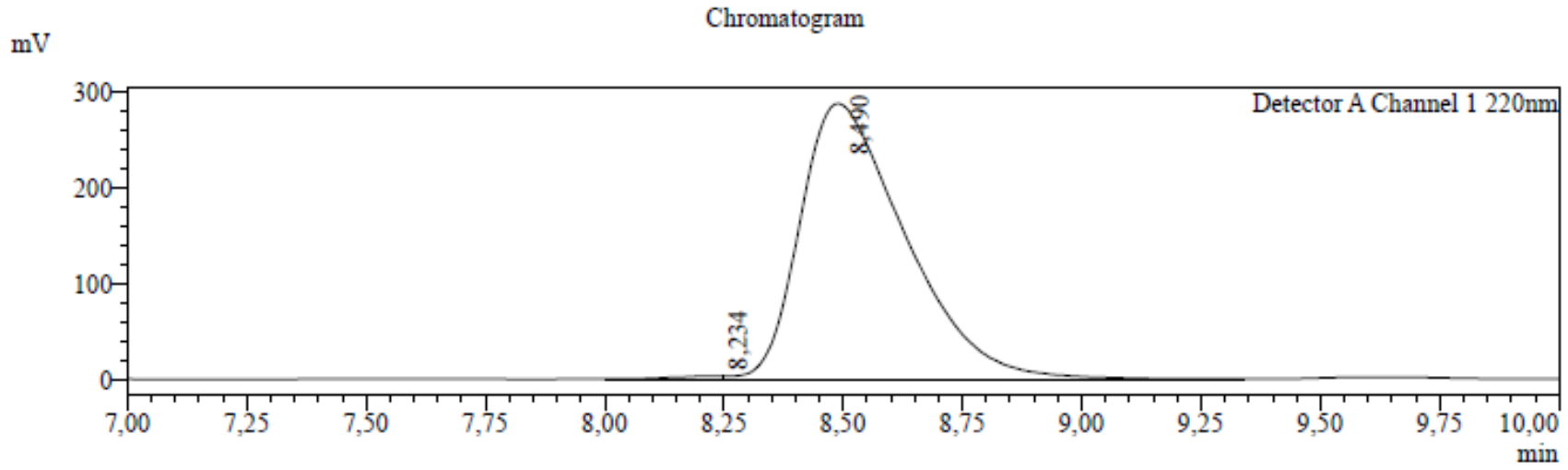

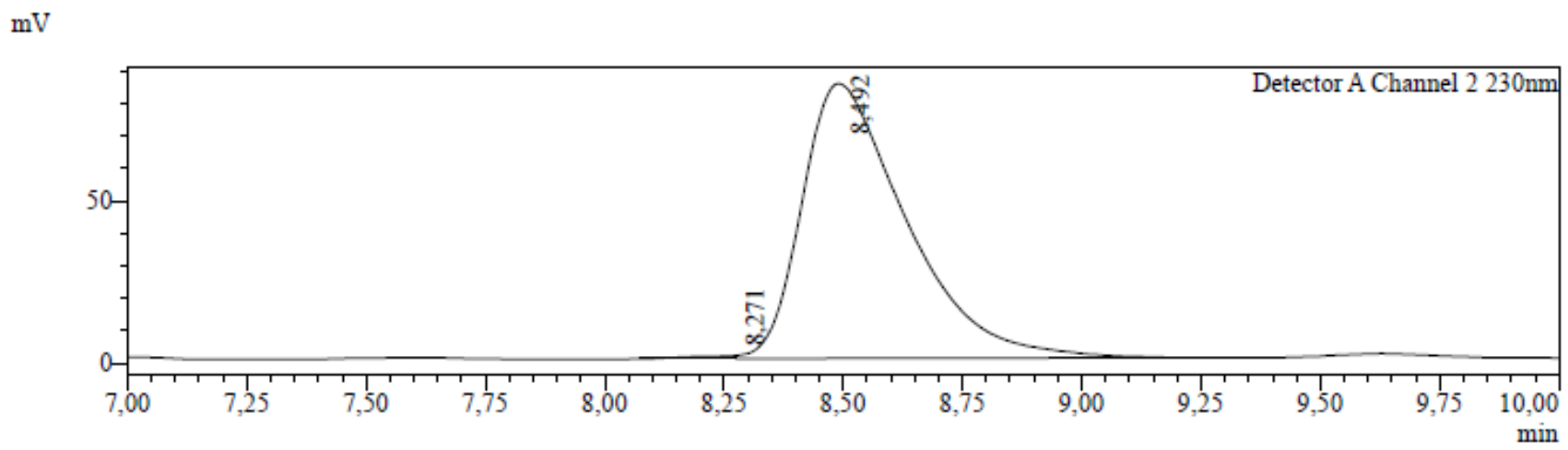

Peak Table

Detector A Channel 1 220nm

Peak#	Ret. Time	Area	Area%
1	8,234	21142	0,479
2	8,490	4389008	99,521
Total		4410150	100,000

Detector A Channel 2 230nm

Peak#	Ret. Time	Area	Area%
1	8,271	5883	0,456
2	8,492	1285296	99,544
Total		1291179	100,000

Method Description:
Column:Lux-3 Cellulose-3
250x4,6mm Particle Size 3 micrometer
Solvent System: 50%(n-Heptan/IPA/DEA 93:7:0,1):50%(n-Heptan+0,1%IPA)
Flow=1 ml/min T=25°C

Chromatogram

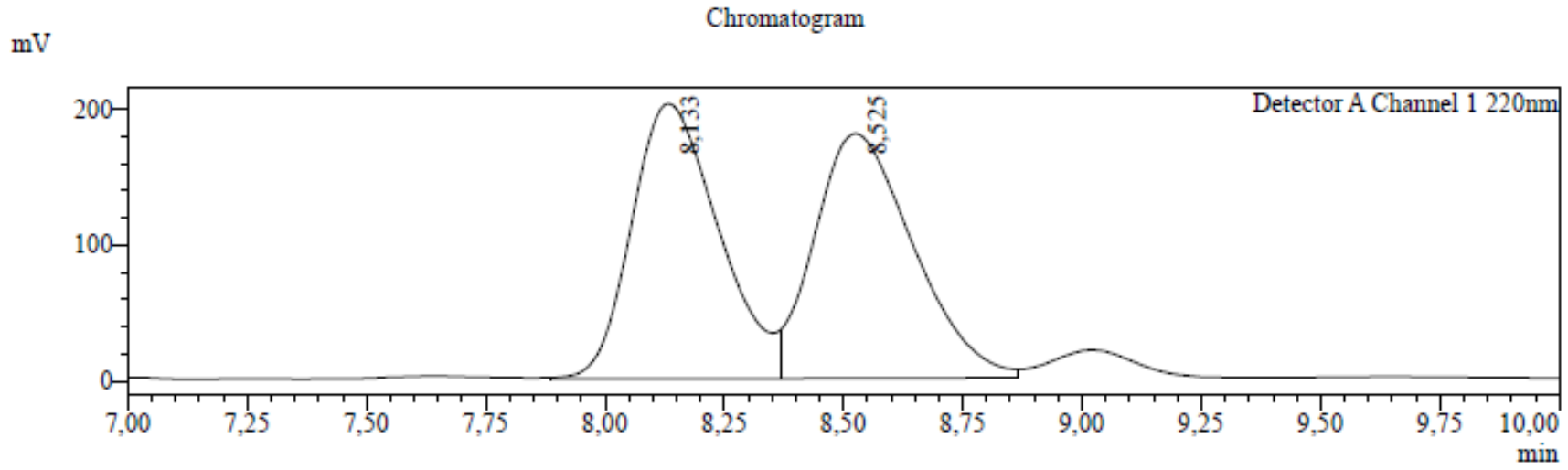

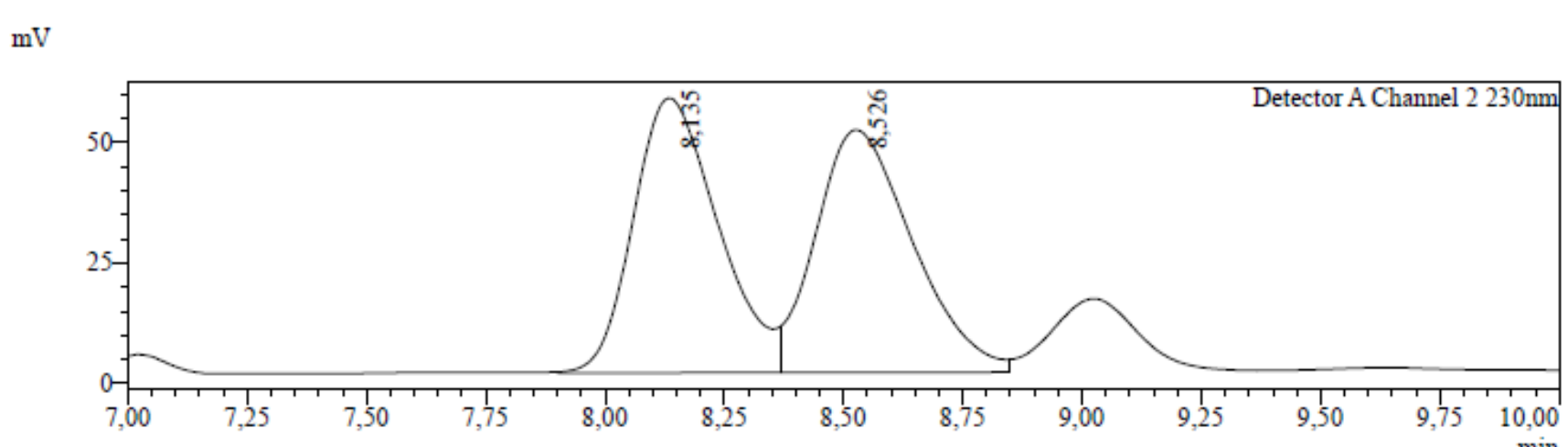

Peak Table

Detector A Channel 1 220nm

Peak#	Ret. Time	Area	Area%
1	8,133	2575281	49,942
2	8,525	2581265	50,058
Total		5156546	100,000

Detector A Channel 2 230nm

Peak#	Ret. Time	Area	Area%
1	8,135	707372	49,891
2	8,526	710476	50,109
Total		1417848	100,000

5-Phenyl-*N*-(1-phenylethyl)pentan-1-amine (1.14)

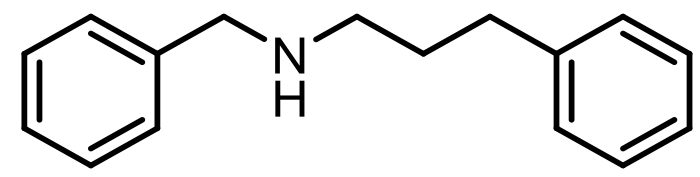

$C_{16}H_{19}N$
MW: 225.3350

Prepared following slightly modified general procedure **4** using *N*-benzylmethylamine (50.3 mg, 0.30 mmol, 1.00 equiv.), styrene (34.5 µL, 0.33 mmol, 1.10 equiv.), trifluoroacetic acid (22.3 µL, 0.30 mmol, 1.00 equiv.) and paraformaldehyde (9.0 mg, 0.30 mmol, 1.0 equiv.). The reaction was conducted for 4 hours. Purification by flash column chromatography (dichloromethane/DMA-mix 95:5 to 0:100) gave the title compound with an average yield of 53% as a yellow oil.

Run 1: 56% (37.9 mg, 0.168 mmol); Run 2: 51% (34.5 mg, 0.153 mmol); Run 3: 51% (34.6 mg, 0.154 mmol).

^{1}H NMR (400 MHz, CDCl$_3$) δ 7.36 – 7.23 (m, 7H), 7.22 – 7.16 (m, 3H), 3.79 (s, 2H), 2.81 – 2.57 (m, 4H), 1.97 – 1.78 (m, 2H), 1.57 (br s, 1H).

^{13}C NMR (101 MHz, CDCl$_3$) δ 142.3, 140.5, 128.53 (3C), 128.47 (2C), 128.3 (2C), 127.1 (2C), 125.9, 54.1, 49.1, 33.8, 31.8.

The spectroscopic properties are consistent with the data available in the literature.[18]

4-Phenyl-*N*-(3-phenyl-3-(4-(trifluoromethyl)phenoxy)propyl)butan-1-amine (1.15a)

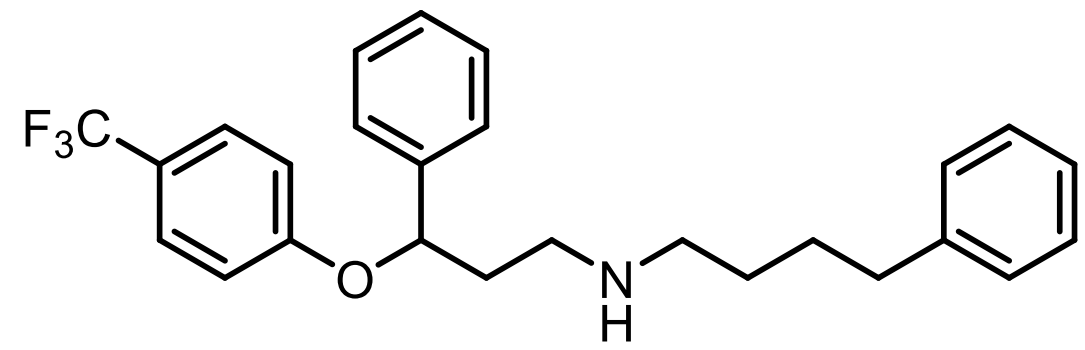

$C_{26}H_{28}F_3NO$
MW. 427.5112

Prepared following general procedure **2** using Fluoxetine hydrochloride (89.9 mg, 0.26 mmol, 1.30 equiv.), allylbenzene (27.0 µL, 0.200 mmol, 1.00 equiv.) and paraformaldehyde (7.8 mg, 0.26 mmol, 1.3 equiv.). Purification by flash column chromatography (dichloromethane/DMA-mix 95:5 to 0:100) gave the title compound (58.2 mg, 0.136 mmol, 68%) as a pale-yellow oil.

^{1}H NMR (600 MHz, $CDCl_3$) δ 7.42 (d, *J* = 8.6 Hz, 2H), 7.36 – 7.30 (m, 4H), 7.29 – 7.22 (m, 4H), 7.21 – 7.13 (m, 3H), 6.89 (d, *J* = 8.5 Hz, 1H), 5.31 (dd, *J* = 8.2, 4.7 Hz, 1H), 2.80 (t, *J* = 6.9 Hz, 2H), 2.65 (t, *J* = 7.2 Hz, 2H), 2.60 (t, *J* = 7.6 Hz, 2H), 2.23 (dq, *J* = 14.4, 7.0 Hz, 1H), 2.11 – 2.02 (m, 1H), 1.70 – 1.59 (m, 2H), 1.59 – 1.52 (m, 2H). *The NH proton was not observed.*

^{13}C NMR (151 MHz, $CDCl_3$) δ 160.8, 142.6, 141.2, 129.2, 128.73 (2C), 128.65, 128.2 (2C), 127.1 (q, *J* = 3.4 Hz, 2C), 126.14 (2C), 126.11 (2C), 124.7 (q, *J* = 271.4 Hz), 123.2 (q, *J* = 32.7 Hz, 2C), 116.1, 79.0, 50.0, 46.3, 38.8, 36.1, 29.6, 29.4.

^{19}F NMR (565 MHz, CDCl3) δ –61.07.

HRMS (ESI$^+$): exact mass calculated for $[M+H]^+$ ($C_{26}H_{29}F_3NO$) requires *m/z* 428.2196, found *m/z* 428.2200.

IR (neat) ν_{max}: 2943, 2866, 1611, 1581, 1562, 1498, 1311, 1254, 1133, 1100, 911 cm^{-1}.

5-Phenyl-*N*-(3-phenyl-3-(4-(trifluoromethyl)phenoxy)propyl)pentan-1-amine (1.15b)

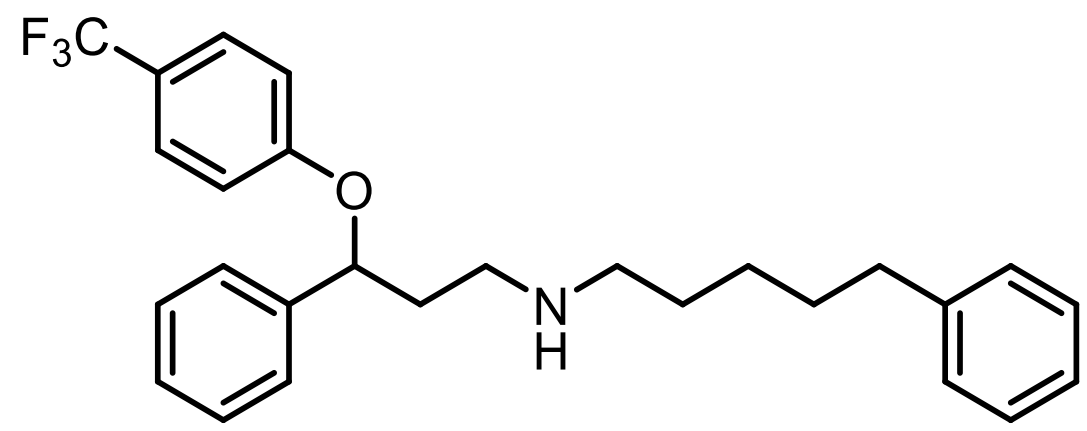

$C_{27}H_{30}F_3NO$
MW: 441.5382

Prepared following general procedure **2** using Fluoxetine hydrochloride (104 mg, 0.30 mmol, 1.00 equiv.), 4-phenyl-1-butene (58.6 µL, 0.330 mmol, 1.10 equiv.) and paraformaldehyde (9.01 mg, 0.300 mmol, 1.00 equiv.). Purification by flash column chromatography (dichloromethane/DMA-mix 95:5 to 0:100) gave the title compound (72.1 mg, 0.163 mmol, 54%) as a colourless oil.

^{1}H NMR (400 MHz, CDCl$_3$) δ 7.42 (d, *J* = 8.7 Hz, 2H), 7.30 (app dd, *J* = 5.9, 4.6 Hz, 5H), 7.27 – 7.22 (m, 2H), 7.16 (dd, *J* = 15.8, 7.3 Hz, 3H), 6.88 (d, *J* = 8.7 Hz, 2H), 5.32 (dt, *J* = 8.4, 4.2 Hz, 1H), 2.88 (t, *J* = 7.1 Hz, 2H), 2.73 – 2.63 (m, 2H), 2.62 – 2.50 (m, 2H), 2.30 (td, *J* = 14.5, 7.0 Hz, 1H), 2.21 – 2.07 (m, 1H), 1.59 (app dq, *J* = 15.3, 7.6 Hz, 4H), 1.38 – 1.22 (m, *J* = 21.3, 14.6, 7.0 Hz, 2H). *The NH proton was not observed.*

^{13}C NMR (151 MHz, CDCl$_3$) δ 160.3, 142.4, 140.3, 129.0 (2C), 128.5 (2C), 128.4 (2C), 128.2, 126.9 (q, *J* = 3.1 Hz, 2C), 125.9 (2C), 125.8, 125.3 (q, *J* = 271.0 Hz), 123.2 (q, *J* = 32.5 Hz), 115.9 (2C), 78.3, 49.2, 45.6, 37.2, 35.9, 31.2, 28.3, 26.8.

^{19}F NMR (377 MHz, CDCl$_3$) δ –61.59.

HRMS (ESI$^+$): exact mass calculated for $[M+H]^+$ ($C_{27}H_{31}F_3NO$) requires *m/z* 442.2352, found *m/z* 442.2357.

IR (neat) ν_{max}: 2930, 2855, 1613, 1323, 1159, 1066, 835, 699 cm^{-1}.

N-(2-Cyclohexylethyl)-3-phenyl-3-(4-(trifluoromethyl)phenoxy)propan-1-amine (1.15c)

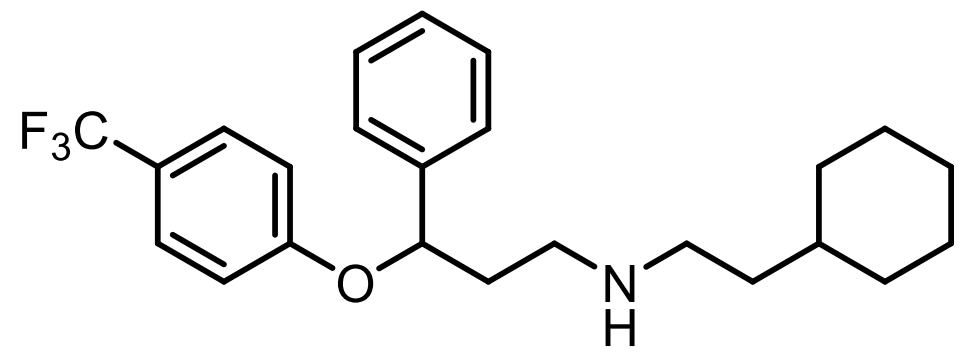

$C_{24}H_{30}F_3NO$
MW: 405.5052

Prepared following general procedure **2** using Fluoxetine hydrochloride (69.2 mg, 0.200 mmol, 1.00 equiv.), methylenecyclohexane (26.4 µL, 0.220 mmol, 1.10 equiv.) and paraformaldehyde (6.0 mg, 0.20 mmol, 1.0 equiv.). Purification by flash column chromatography (dichloromethane/DMA-mix 95:5 to 0:100) gave the title compound (35.7 mg, 88.0 µmol, 44%) as a brown oil.

^{1}H NMR (600 MHz, CDCl$_3$) δ7.43 (d, *J* = 8.6 Hz, 2H), 7.33 (d, *J* = 4.4 Hz, 4H), 7.27 – 7.24 (m, 1H), 6.90 (d, *J* = 8.6 Hz, 2H), 5.30 (dd, *J* = 8.2, 4.7 Hz, 1H), 2.78 (t, *J* = 6.9 Hz, 2H), 2.61 (td, *J* = 7.3, 3.3 Hz, 2H), 2.25 – 2.15 (m, 1H), 2.12 – 1.96 (m, 1H), 1.73 – 1.60 (m, 5H), 1.36 (q, *J* = 7.2 Hz, 2H), 1.31 – 1.07 (m, 5H), 0.95 – 0.78 (m, 2H).
^{13}C NMR (151 MHz, CDCl$_3$) δ 160.7, 141.1, 128.9 (2C), 128.0 (2C), 126.9 (q, *J* = 3.9 Hz, 2C), 126.3 (q, *J* = 271.3 Hz), 125.9, 122.9 (q, *J* = 32.7 Hz, 2C), 115.9, 78.9, 47.7, 46.3, 38.9, 37.7, 35.8, 33.5, 26.7 (2C), 26.4 (2C).
^{19}F NMR (565 MHz, CDCl$_3$) δ –61.57.
HRMS (ESI$^+$): exact mass calculated for $[M+H]^+$ ($C_{24}H_{31}F_3NO$) requires *m/z* 406.2352, found *m/z* 406.2358.
IR (neat) ν_{max}: 3301, 3100, 2934, 2851, 1616, 1588, 1288, 1144, 1138, 861, 712 cm^{-1}.

2-(1-(4-Chlorobenzoyl)-5-methoxy-2-methyl-1*H*-indol-3-yl)-*N*-methyl-*N*-(12-(phenethylamino)dodecyl)acetamide (1.16)

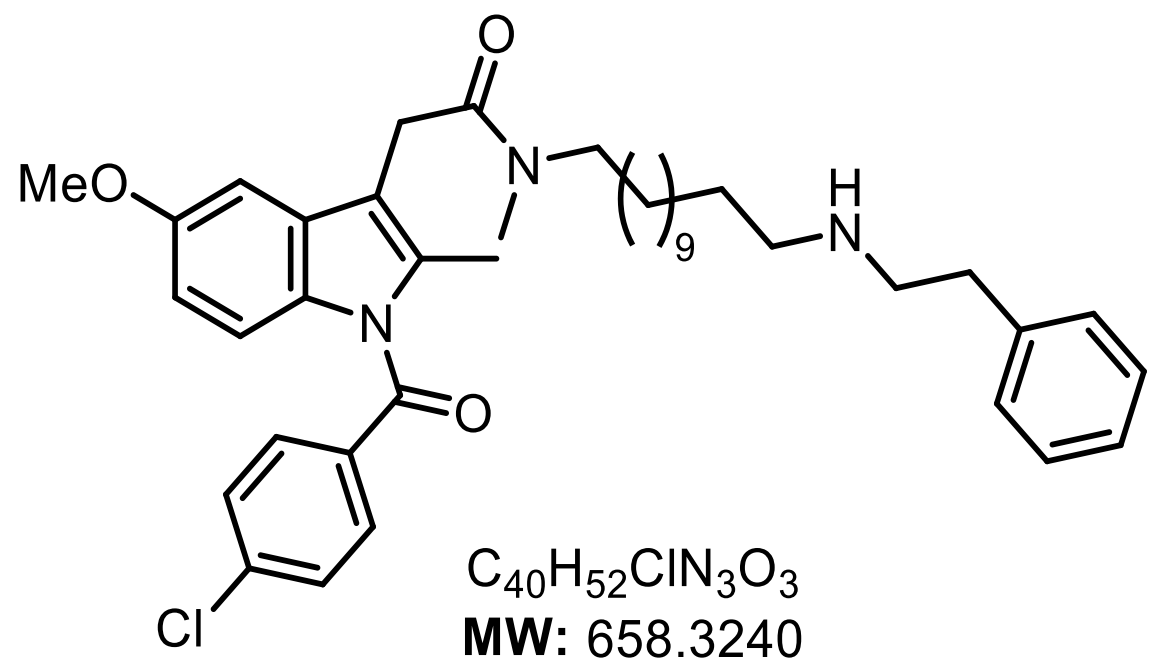

Prepared following general procedure **4** using methyl *N*-methyl-phenethylamine (21.8 µL, 0.150 mmol, 1.50 equiv.), **S1.16b** (52.2 mg, 0.100 mmol, 1.00 equiv.), trifluoroacetic acid (11.1 µL, 0.150 mmol, 1.50 equiv.) and paraformaldehyde (5.1 mg, 0.15 mmol, 1.5 equiv.). Purification by flash column chromatography (dichloromethane/DMA-mix 95:5 to 0:100) gave the title compound (34.9 mg, 53.0 µmol, 53%) as a brown oil.

Rotameric effects are visible in the measured NMR spectra.

^{1}H NMR (600 MHz, CDCl$_3$) δ 8.51 (br s, 1H), 7.69 – 7.59 (m, 2H), 7.49 – 7.44 (m, 2H), 7.31 – 7.27 (m, 2H), 7.24 – 7.17 (m, 3H), 7.00 (dd, *J* = 10.4, 2.6 Hz, 1H), 6.81 (t, *J* = 8.7 Hz, 1H), 6.63 (dt, *J* = 9.0, 1.7 Hz, 1H), 3.81 (s, 3H), 3.70 (d, *J* = 11.3 Hz, 2H), 3.43 – 3.35 (m, 1H), 3.35 – 3.30 (m, 1H), 3.12 – 3.05 (m, 3H), 3.03 (s, 1.5H), 2.94 (s, 1.5H), 2.90 – 2.85 (m, 2H), 2.37 (app d, *J* = 1.8 Hz, 3H), 1.75 – 1.68 (m, 2H), 1.60 – 1.46 (m, 2H), 1.33 – 1.14 (m, 17H).

^{13}C NMR (151 MHz, CDCl$_3$) δ [170.01, 169.91], [168.45, 168.41], 156.1, 139.3, 137.1, [135.36, 135.33], [134.14, 134.12], 131.3, [131.07, 131.03], 131.0 (2C), 129.2 (2C), 129.0 (2C), 128.8 (2C), 127.2, [114.98, 114.96], [113.91, 113.66], [111.67, 111.63], [101.79, 101.77], 55.8, 50.4, [49.10, 49.07], 48.5, 47.9, [35.84, 33.82], [32.78, 32.76], 31.0, 30.3, [29.67, 29.65], [29.59, 29.56], [29.52, 29.48], [29.23, 29.21], [28.68, 27.41], [27.00, 26.99], [26.95, 26.91], [26.47, 26.45], 13.6.

HRMS (ESI$^+$): exact mass calculated for $[M+H]^+$ ($C_{40}H_{53}{}^{35}ClN_3O_3$) requires *m/z* 658.3770, found *m/z* 658.3763.

IR (neat) ν_{max}: 3272, 2961, 2882, 1697, 1597, 1452, 1343, 1292, 1087, 923, 865 cm^{-1}.

4-(3-(Phenethylamino)propyl)benzyl 3-(4,5-diphenyloxazol-2-yl)propanoate (1.17)

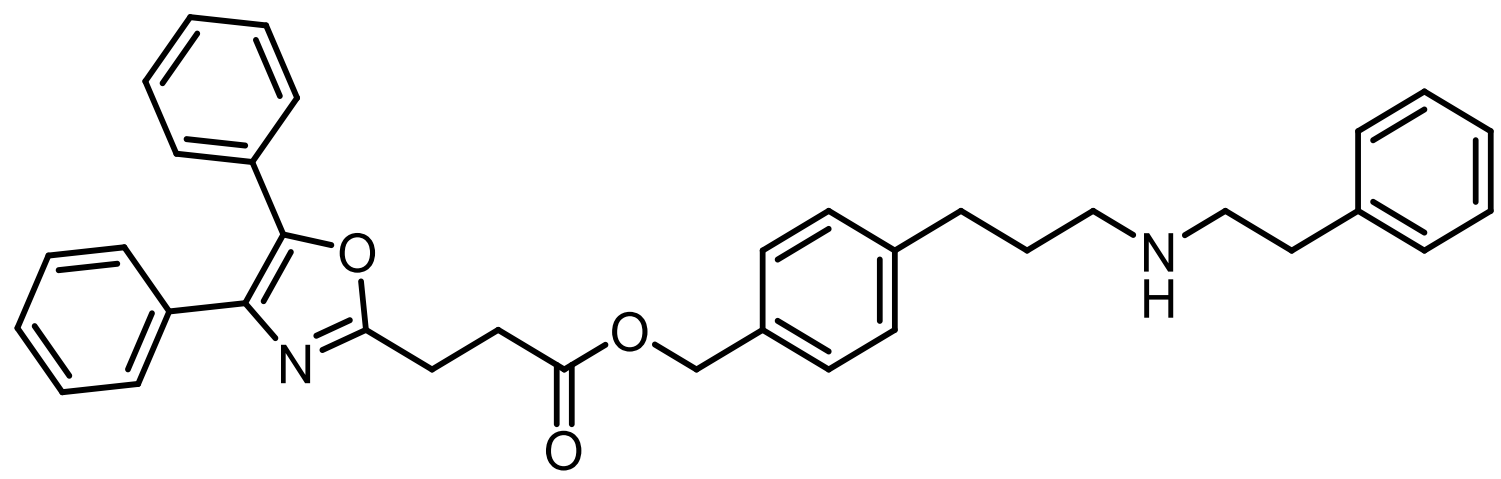

$C_{36}H_{36}N_2O_3$
MW: 544.6950

Prepared following general procedure **4** using *N*-methyl-phenethylamine (65.4 µL, 0.450 mmol, 1.50 equiv.), 4-vinylbenzyl 3-(4,5-diphenyloxazol-2-yl)propanoate (123.0 mg, 0.300 mmol, 1.00 equiv.), trifluoroacetic acid (33.4 µL, 0.450 mmol, 1.50 equiv.) and paraformaldehyde (13.5 mg, 0.450 mmol, 1.50 equiv.). The reaction time was shortened to 4 hours. The reaction was conducted at 50 °C. Purification by flash column chromatography (dichloromethane/DMA-mix 95:5 to 0:100) gave the title compound (103.0 mg, 0.189 mmol, 65%) as a yellow-brown oil.

^{1}H NMR (400 MHz, $CDCl_3$) δ 7.65 – 7.58 (m, 2H), 7.57 – 7.52 (m, 2H), 7.39 – 7.26 (m, 8H), 7.24 – 7.15 (m, 5H), 7.08 (d, *J* = 8.1 Hz, 2H), 5.10 (s, 2H), 3.19 (dd, *J* = 8.1, 6.8 Hz, 2H), 3.04 – 2.91 (m, 6H), 2.78 (t, *J* = 7.6 Hz, 2H), 2.62 (t, *J* = 7.6 Hz, 2H), 1.98 (m, 2H). *The NH proton was not observed.*
^{13}C NMR (101 MHz, $CDCl_3$) δ 172.0, 161.8, 153.5, 145.6, 141.2, 135.2, 133.7, 132.6, 129.1, 128.82 (2C), 128.77 (2C), 128.68 (2C), 128.65 (2C), 128.6 (3C), 128.2 (3C), 128.1 (2C), 126.8, 126.6 (2C), 66.5, 50.4, 48.6 (2C), 34.6, 33.0, 29.7, 23.7.
HRMS (ESI$^+$): exact mass calculated for $[M+H]^+$ ($C_{36}H_{37}N_2O_3$) requires *m/z* 545.2799, found *m/z* 545.2801.
IR (neat) ν_{max}: 3255, 3211, 2976, 2863, 1700, 1611, 1555, 1422, 1311, 1050, 973 cm^{-1}.

4-(3-(Phenethylamino)propyl)benzyl 4-(*N*,*N*-dipropylsulfamoyl)benzoate (1.18)

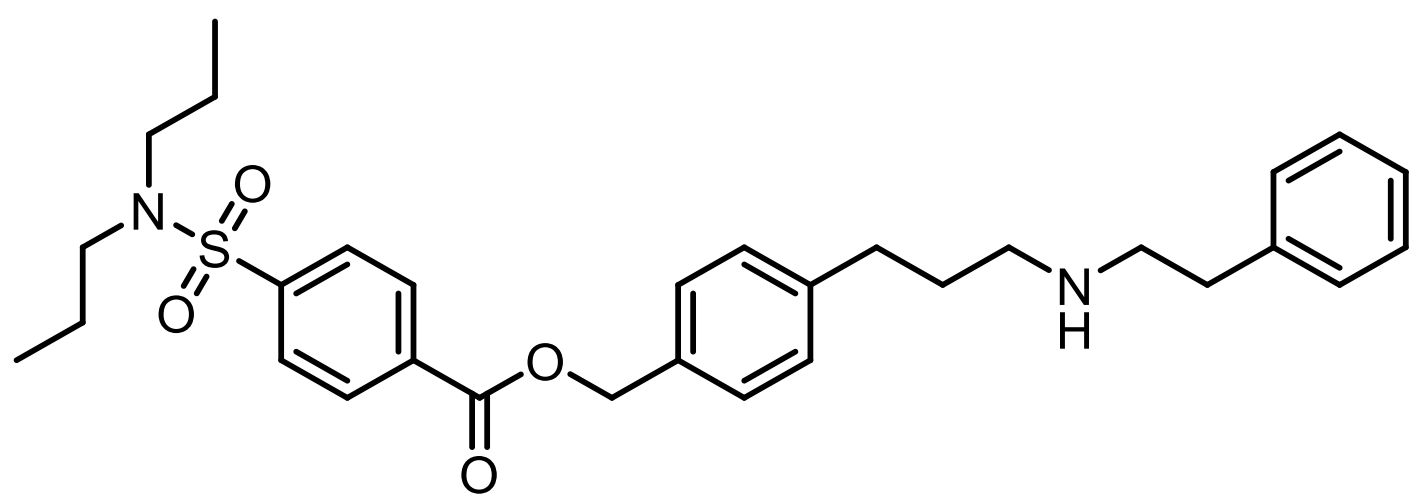

$C_{31}H_{40}N_2O_4S$
MW: 536.7310

Prepared following general procedure **4** using *N*-methyl-phenethylamine (43.6 µL, 0.300 mmol, 1.50 equiv.), 4-vinylbenzyl 4-(*N*,*N*-dipropylsulfamoyl)benzoate (80.3 mg, 0.200 mmol, 1.00 equiv.), trifluoroacetic acid (22.3 µL, 0.300 mmol, 1.50 equiv.) and paraformaldehyde (9.0 mg, 0.30 mmol, 1.5 equiv.). The reaction time was shortened to 4 hours. The reaction was conducted at 50 °C. Purification by flash column chromatography (dichloromethane/DMA-mix 95:5 to 0:100) gave the title compound (64.4 mg, 0.120 mmol, 60%) as a yellow-brown oil.

^{1}H NMR (400 MHz, CDCl$_3$) δ 8.18 – 8.13 (m, 2H), 7.88 – 7.82 (m, 2H), 7.34 – 7.26 (m, 4H), 7.25 – 7.14 (m, 5H), 5.31 (s, 2H), 3.20 – 3.06 (m, 4H), 3.06 – 2.94 (m, 4H), 2.88 – 2.77 (m, 2H), 2.66 (t, *J* = 7.6 Hz, 2H), 2.14 – 1.99 (m, 2H), 1.65 – 1.46 (m, 4H), 0.86 (t, *J* = 7.4 Hz, 6H). *The NH proton was not observed.*

^{13}C NMR (101 MHz, CDCl$_3$) δ 165.2, 144.5 (2C), 141.4, 138.1, 133.6 (2C), 133.5, 130.5, 128.9 (2C), 128.84, 128.79 (2C), 128.76 (2C), 127.1 (2C), 126.9, 67.3, 50.3, 50.1, 48.4 (2C), 34.3, 33.0, 32.8, 22.1 (2C), 11.3 (2C).

HRMS (ESI$^+$): exact mass calculated for $[M+H]^+$ ($C_{31}H_{41}N_2O_4S$) requires *m/z* 537.2783, found *m/z* 537.2786.

IR (neat) ν_{max}: 3278, 3053, 2957, 2878, 1600, 1589, 1522, 1476, 1201, 1066, 897 cm^{-1}.

(*S*)-2-(4-(4-Chlorophenyl)-2,3,9-trimethyl-6*H*-thieno[3,2-*f*][1,2,4]triazolo[4,3-*a*][1,4]diazepin-6-yl)-*N*-methyl-*N*-(6-(phenethylamino)hexyl)acetamide (1.19)

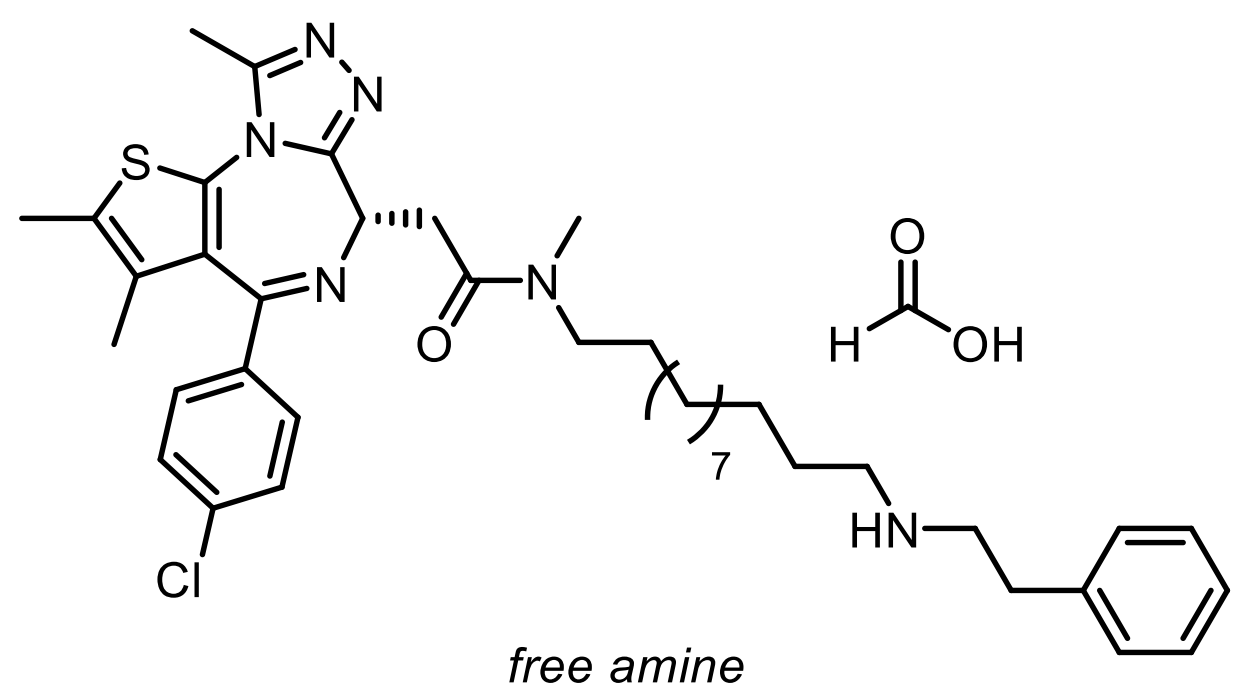

free amine
$C_{40}H_{53}ClN_6OS$
MW: 701.4150

Prepared following general procedure **4** using *N*-methyl-phenethylamine (43.6 µL, 0.300 mmol, 1.00 equiv.), **S1.19b** (187 mg, 0.330 mmol, 1.10 equiv.), TFA (22.3 µL, 0.300 mmol, 1.00 equiv.) and paraformaldehyde (9.01 mg, 0.30 mmol, 1.00 equiv.). Purification by preparative high-performance liquid chromatography (water + 0.1% formic acid/acetonitrile 95:5 to 50:50) gave the title compound as a formate salt (132 mg, 0.177 mmol, 59%) as a pale-yellow oil.

Rotameric effects are visible in the measured NMR spectra.

^{1}H NMR (400 MHz, $CDCl_3$) δ 8.48 (br s, 1H), 7.39 (d, *J* = 8.5 Hz, 2H), 7.33 – 7.27 (m, 4H), 7.24 – 7.17 (m, 3H), 4.81 (q, *J* = 6.3 Hz, 1H), 3.68 – 3.53 (m, 2H), 3.52 – 3.44 (m, 1H), 3.44 – 3.35 (m, 1H), 3.20 (s, 1.5H), 3.15 – 3.08 (m, 2H), 3.08 – 3.01 (m, 2H), 2.98 (s, 1.5H), 2.92 – 2.84 (m, 2H), 2.69 – 2.60 (m, 3H), 2.43 – 2.30 (m, 3H), 1.78 – 1.63 (m, 6H), 1.58– 1.47 (m, 1H), 1.41 – 1.03 (m, 17H).

^{13}C NMR (151 MHz, $CDCl_3$) δ [170.4, 170.3], 167.9, [163.7, 163.6], 156.2, [149.92, 149.90], [137.1, 137.0], 136.7, [132.34, 132.30], 131.1, [130.75, 130.72], 129.9 (2C), 128.9 (2C), 128.8 (4C), 127.1, [54.8, 54.6], 50.5, [49.02, 49.00], 48.1, 47.8, [35.9, 35.3], [35.7, 33.9], 32.7, 29.66, [29.61, 26.60], [26.56, 29.54], [29.50, 29.49], [29.2, 29.1], 28.8, 27.5, 27.0, [26.94, 26.91], [26.4, 26.3], [14.53, 14.51], 13.2, [12.0, 11.9].

HRMS (ESI$^+$): exact mass calculated for $[M+H]^+$ ($C_{40}H_{54}{}^{35}ClN_6OS$) requires *m/z* 701.3763, found *m/z* 701.3750.

IR (neat) ν_{max}: 3008, 2198, 1639, 1590, 1486, 1413, 1171, 987, 699, 539 cm^{-1}.

(4*R*)-4-((8*R*,9*S*,10*S*,13*R*,14*S*,17*R*)-10,13-Dimethyl-3,7,12-trioxohexadecahydro-1*H*-cyclopenta[*a*]phenanthren-17-yl)-*N*-methyl-*N*-(12-(phenethylamino)dodecyl)pentanamide (1.20)

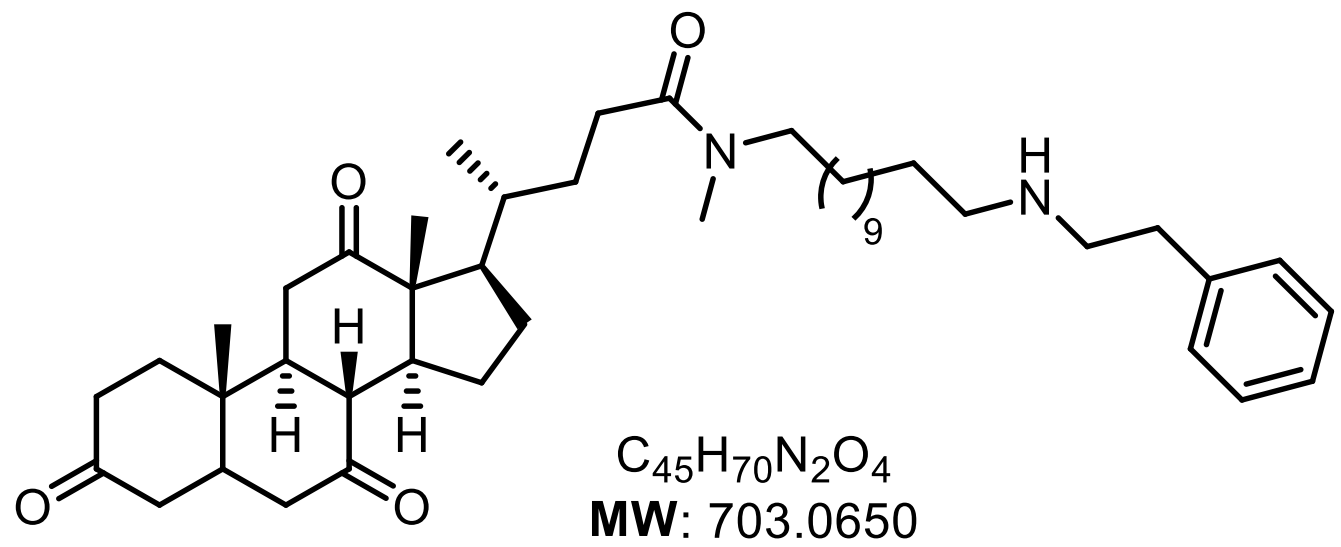

Prepared following general procedure **4** using *N*-methyl-phenethylamine (21.8 µL, 0.150 mmol, 1.50 equiv.), **S1.20b** (56.8 mg, 0.100 mmol, 1.00 equiv.), trifluoroacetic acid (11.1 µL, 0.150 mmol, 1.50 equiv.) and paraformaldehyde (5.1 mg, 0.15 mmol, 1.5 equiv.). Purification by flash column chromatography (dichloromethane/DMA-mix 95:5 to 0:100) gave the title compound (24.6 mg, 35.0 µmol, 35%) as a brown oil.

Rotameric effects are visible in the measured NMR spectra.

^{1}H NMR (600 MHz, $CDCl_3$) δ 8.45 (br s, 1H), 7.31 – 7.28 (m, 2H), 7.25 – 7.19 (m, 3H), 3.41 – 3.20 (m, 3H), 3.16 – 3.05 (m, 4H), 2.97 (s, 1.5H), 2.94 – 2.81 (m, 6.5H), 2.71 – 2.59 (m, 1H), 2.43 – 2.19 (m, 8H), 2.17 – 2.08 (m, 2H), 2.06 – 2.01 (m, 3H), 1.99 – 1.93 (m, 1H), 1.91 – 1.81 (m, 2H), 1.80 – 1.70 (m, 2H), 1.61 (td, *J* = 14.1, 4.5 Hz, 1H), 1.55 (dt, *J* = 12.8, 6.6 Hz, 1H), 1.52 – 1.45 (m, 1H), 1.40 (s, 3H), 1.38 – 1.17 (m, 18H), 1.08 (s, 3H), 0.88 – 0.84 (m, 3H).

^{13}C NMR (151 MHz, $CDCl_3$) δ [212.34, 212.31], 209.2, 208.9, [173.31, 173.19], 137.0, 129.0 (2C), 128.8 (2C), [127.22, 127.21], 57.1, [51.93, 51.91], 50.2, 49.2, [49.12, 49.10], [47.98, 47.93], 47.0, [45.91, 45.84], 45.7, 45.1, 43.0, 38.8, [36.64, 36.17], [35.67, 35.64], [35.53, 35.43], [33.55, 32.77], 31.1, [30.72, 30.65], 30.0, [29.85, 29.64], [29.57, 29.53], [29.51, 29.50], 29.48, [29.21, 29.19], 28.7, 27.7, 27.4, 27.0, 26.9, 26.8, 26.5, 26.4, 25.3, 22.1, [19.03, 19.01], [12.08, 12.06].

HRMS (ESI$^+$): exact mass calculated for $[M+H]^+$ ($C_{45}H_{71}N_2O_4$) requires *m/z* 703.5408, found *m/z* 703.5414.

IR (neat) ν_{max}: 3277, 2965, 2871, 1703, 1687, 1683, 1522, 1417, 1324, 1097, 965 cm^{-1}.

(8*R*,9*S*,13*S*,14*S*)-13-Methyl-3-((4-(3-(phenethylamino)propyl)benzyl)oxy)-6,7,8,9,11,12,13,14,15,16-decahydro-17*H*-cyclopenta[*a*]phenanthren-17-one (1.21)

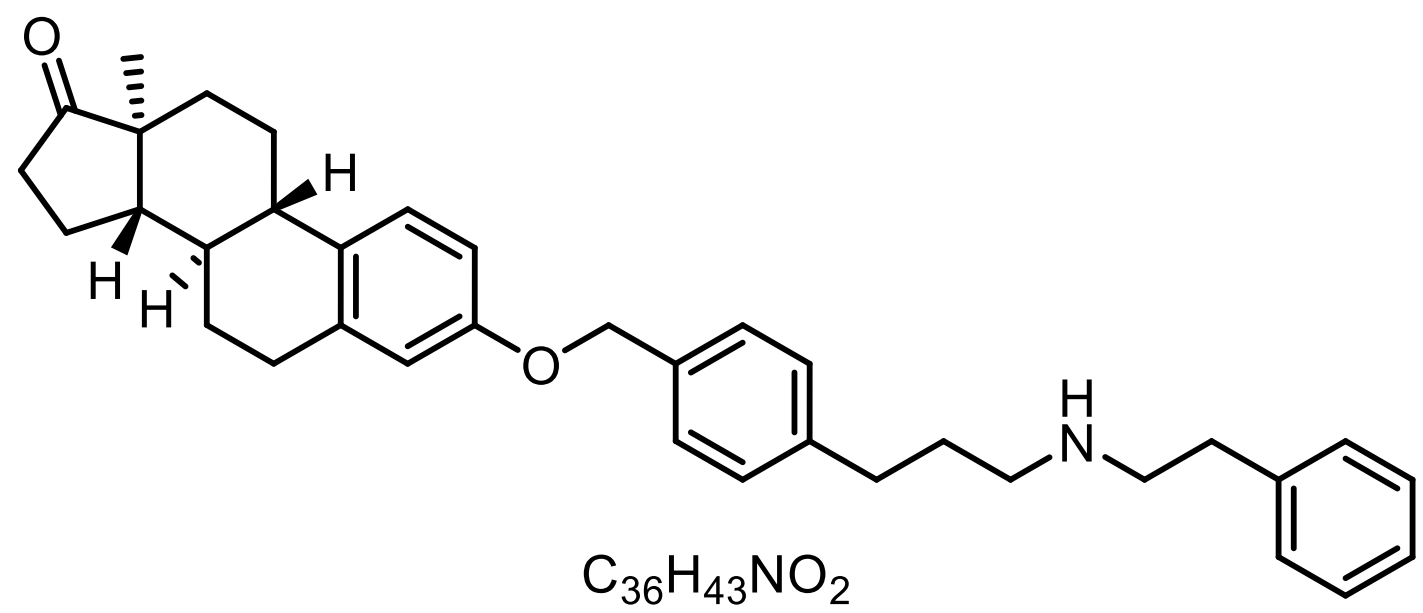

$C_{36}H_{43}NO_2$
MW: 521.7450

Prepared following general procedure **4** using *N*-methyl-phenethylamine (65.4 µL, 0.450 mmol, 1.50 equiv.), **S1.24b** (116.0 mg, 0.300 mmol, 1.00 equiv.), trifluoroacetic acid (33.4 µL, 0.450 mmol, 1.50 equiv.) and paraformaldehyde (13.5 mg, 0.450 mmol, 1.50 equiv.). The reaction time was shortened to 4 h. The reaction was conducted at 50 °C. Purification by flash column chromatography (dichloromethane/DMA-mix 95:5 to 0:100) gave the title compound (101.9 mg, 0.195 mmol, 65%) as a yellow-brown oil.

^{1}H NMR (400 MHz, CDCl$_3$) δ 7.34 – 7.25 (m, 4H), 7.24 – 7.13 (m, 6H), 6.77 (dd, *J* = 8.5, 2.8 Hz, 1H), 6.72 (d, *J* = 2.7 Hz, 1H), 4.96 (s, 2H), 3.04 – 2.92 (m, 4H), 2.93 – 2.86 (m, 2H), 2.76 (d, *J* = 7.5 Hz, 2H), 2.65 (t, *J* = 7.6 Hz, 2H), 2.50 (dd, *J* = 18.8, 8.6 Hz, 1H), 2.42 – 2.36 (m, 1H), 2.31 – 2.22 (m, 1H), 2.20 – 1.89 (m, 7H), 1.70 – 1.34 (m, 6H), 0.91 (s, 3H).

^{13}C NMR (101 MHz, CDCl$_3$) δ 157.0, 141.0, 138.8, 137.9, 135.1, 132.4, 128.82 (2C), 128.76 (2C), 128.67 (2C), 127.9 (2C), 126.7, 126.5, 115.0, 112.5, 69.9, 50.6, 50.5, 48.7, 48.2, 44.1, 38.5, 36.0, 35.0, 33.1, 31.7, 30.1, 29.8, 26.7, 26.1, 21.7, 14.0. *The signal of the carbonyl carbon was not observed.*

HRMS (ESI$^+$): exact mass calculated for $[M+H]^+$ ($C_{36}H_{44}NO_2$) requires *m/z* 522.3367, found *m/z* 522.3371.

IR (neat) ν_{max}: 3278, 2968, 2860, 1681, 1677, 1611, 1489, 1211, 1093, 965 cm^{-1}.

(4*R*)-4-((*8R,9S,10S,13R,14S,17R*)-10,13-Dimethyl-3,7,12-trioxohexadecahydro-1*H*-cyclopenta[*a*]phenanthren-17-yl)-*N*-methyl-*N*-(12-(phenethylamino)dodecyl)pentanamide (1.22)

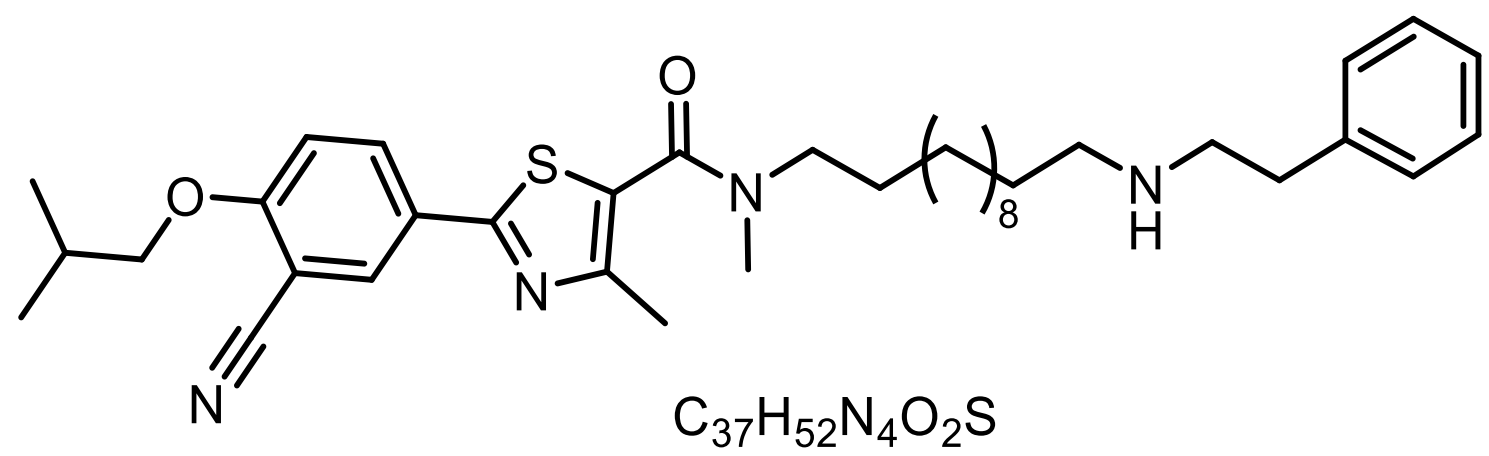

$C_{37}H_{52}N_4O_2S$
MW: 616.9090

Prepared following general procedure **4** using *N*-methyl-phenethylamine (21.8 µL, 0.150 mmol, 1.50 equiv.), **S1.22b** (48.2 mg, 0.100 mmol, 1.00 equiv.), trifluoroacetic acid (11.1 µL, 0.150 mmol, 1.50 equiv.) and paraformaldehyde (5.1 mg, 0.15 mmol, 1.5 equiv.). Purification by flash column chromatography (dichloromethane/DMA-mix 95:5 to 0:100) gave the title compound (34.5 mg, 56.0 µmol, 56%) as a brown oil.

Rotameric effects are visible in the measured NMR spectra.

^{1}H NMR (600 MHz, CDCl$_3$) δ 8.11 (d, *J* = 2.3 Hz, 1H), 8.03 (dd, *J* = 8.8, 2.4 Hz, 1H), 7.29 (t, *J* = 7.4 Hz, 2H), 7.25 – 7.18 (m, 3H), 7.00 (d, *J* = 8.4 Hz, 1H), 3.89 (d, *J* = 6.5 Hz, 2H), 3.54 – 3.29 (m, 2H), 3.14 – 3.01 (m, 6H), 2.85 (t, *J* = 7.9 Hz, 2H), 2.46 (s, 3H), 2.24 – 2.17 (m, 1H), 1.76 – 1.66 (m, 2H), 1.64 – 1.57 (m, 2H), 1.35 – 1.17 (m, 18H), 1.09 (d, *J* = 6.7 Hz, 6H).
^{13}C NMR (151 MHz, CDCl$_3$) δ 164.8, 163.6, 162.3, 152.7, 137.3 (2C), 132.5, 131.9, 128.9 (2C), 128.8 (2C), 127.1, 126.3, 115.7, 112.8, 103.0, 75.8, 49.3, 48.1, 33.1, 31.1, 29.8, 29.60 (2C), 29.59 (2C), 29.55, 29.50, 29.4 (2C), 29.2, 28.3, 26.9, 26.8, 19.2, 16.6.
HRMS (ESI$^+$): exact mass calculated for $[M+H]^+$ ($C_{37}H_{53}N_4O_2S$) requires *m/z* 617.3884, found *m/z* 617.3890.
IR (neat) ν_{max}: 3299, 2973, 2841, 2260, 1699, 1476, 1343, 1222, 1064, 978 cm^{-1}.

8-Bromo-*N*-phenethyloctan-1-amine (1.23)

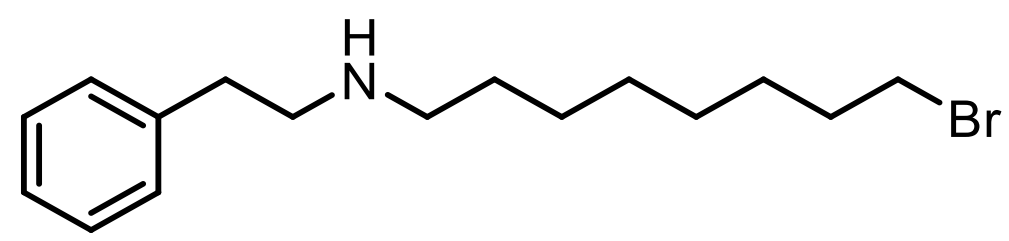

$C_{16}H_{26}BrN$
MW: 312.2950

Prepared following general procedure **4** using *N*-methyl-phenethylamine (43.6 µL, 0.300 mmol, 1.00 equiv.), 7-bromo-1-heptene (51.3 µL, 0.330 mmol, 1.10 equiv.), trifluoroacetic acid (22.3 µL, 0.300 mmol, 1.00 equiv.) and paraformaldehyde (9.01 mg, 0.300 mmol, 1.00 equiv.). Purification by flash column chromatography (dichloromethane/DMA-mix 95:5 to 0:100) gave the title compound (65.2 mg, 0.210 mmol, 69%) as a colourless oil.

^{1}H NMR (400 MHz, $CDCl_3$) δ 7.33 – 7.27 (m, 2H), 7.25 – 7.21 (m, 3H), 3.37 (t, *J* = 6.8 Hz, 2H), 3.19 – 3.12 (m, 2H), 3.12 – 3.06 (m, 2H), 2.89 – 2.83 (m, 2H), 1.87 – 1.70 (m, 4H), 1.45 – 1.15 (m, 9H).
^{13}C NMR (101 MHz, $CDCl_3$) δ 137.7, 128.9 (2C), 128.8 (2C), 127.0, 49.9, 48.7, 34.1, 33.7, 32.8, 29.1, 28.7, 28.1, 27.3, 27.0.
HRMS (ESI$^+$): exact mass calculated for $[M+H]^+$ ($C_{16}H_{27}N^{79}Br$) requires *m/z* 312.1321, found *m/z* 312.1315.
IR (neat) ν_{max}: 3024, 2928, 2854, 2785, 1496, 1454, 1378, 1265, 1030, 747, 696, 510 cm^{-1}.

5-(Phenethylamino)pentyl acetate (1.24)

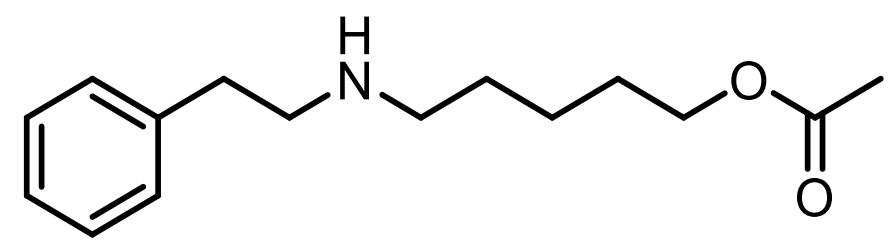

$C_{15}H_{23}NO_2$
MW: 249.3540

Prepared following general procedure **4** using *N*-methyl-phenethylamine (43.6 µL, 0.300 mmol, 1.00 equiv.), 3-butenyl acetate (41.4 µL, 0.330 mmol, 1.10 equiv.), trifluoroacetic acid (22.3 µL, 0.300 mmol, 1.00 equiv.) and paraformaldehyde (9.01 mg, 0.300 mmol, 1.00 equiv.). Purification by flash column chromatography (dichloromethane/DMA-mix 95:5 to 0:100) gave the title compound (38.1 mg, 0.153 mmol, 51%) as a pale-yellow oil.

^{1}H NMR (400 MHz, $CDCl_3$) δ 7.30 – 7.25 (m, 2H), 7.22 – 7.15 (m, 3H), 4.02 (t, *J* = 6.7 Hz, 2H), 2.95 – 2.78 (m, 4H), 2.68 – 2.59 (m, 2H), 2.02 (s, 3H), 1.65 – 1.47 (m, 4H), 1.40 – 1.30 (m, 2H). *The NH proton was not observed.*

^{13}C NMR (101 MHz, $CDCl_3$) δ 171.3, 139.8, 128.8 (2C), 128.7 (2C), 126.4, 64.5, 51.1, 49.6, 36.1, 29.4, 28.6, 23.8, 21.1.

HRMS (ESI$^+$): exact mass calculated for $[M+H]^+$ ($C_{15}H_{24}NO_2$) requires *m/z* 250.1802, found *m/z* 250.1801.

IR (neat) ν_{max}: 2933, 2857, 1734, 1543, 1384, 1236, 1033, 784 cm^{-1}.

Methyl 7-(phenethylamino)heptanoate (1.25)

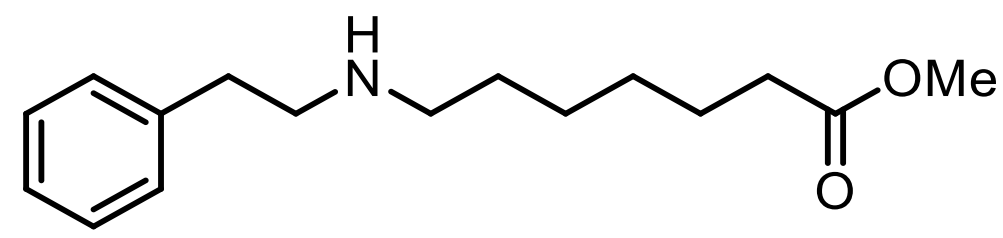

$C_{16}H_{25}NO_2$
MW: 263.3810

Prepared following general procedure **4** using *N*-methyl-phenethylamine (43.6 µL, 0.300 mmol, 1.00 equiv.), *N*-3-methyl 5-hexenoate (46.3 µL, 0.330 mmol, 1.10 equiv.), trifluoroacetic acid (22.3 µL, 0.300 mmol, 1.00 equiv.) and paraformaldehyde (9.01 mg, 0.300 mmol, 1.00 equiv.). Purification by flash column chromatography (dichloromethane/DMA-mix 95:5 to 0:100) gave the title compound (42.1 mg, 0.160 mmol, 53%) as a yellow oil.

^{1}H NMR (400 MHz, $CDCl_3$) δ 7.32 – 7.27 (m, 2H), 7.20 (dt, *J* = 9.2, 4.0 Hz, 3H), 3.66 (s, 3H), 2.95 – 2.88 (m, 2H), 2.88 – 2.83 (m, 2H), 2.70 – 2.59 (m, 2H), 2.33 – 2.19 (m, 2H), 1.66 – 1.55 (m, 2H), 1.56 – 1.46 (m, 2H), 1.36 – 1.26 (m, 4H). *The NH proton was not observed.*

^{13}C NMR (101 MHz, $CDCl_3$) δ 174.3, 139.7, 128.8 (2C), 128.7 (2C), 126.4, 51.6, 51.1, 49.6, 35.9, 34.1, 29.4, 29.1, 27.0, 25.0.

HRMS (ESI$^+$): exact mass calculated for $[M+H]^+$ ($C_{16}H_{26}NO_2$) requires *m/z* 264.1958, found *m/z* 264.1951.

IR (neat) ν_{max}: 2930, 2855, 1735, 1495, 1254, 1170, 1029, 747 cm^{-1}.

2-(5-(Phenethylamino)pentyl)isoindoline-1,3-dione (1.26)

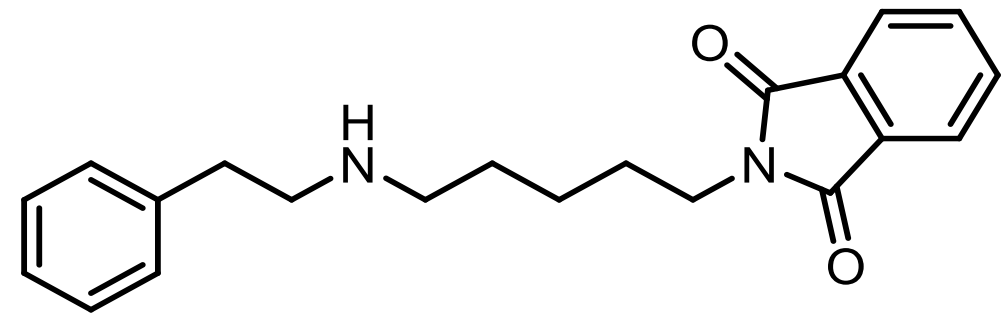

$C_{21}H_{24}N_2O_2$
MW: 336.4350

Prepared following general procedure **4** using *N*-methyl-phenethylamine (43.6 µL, 0.300 mmol, 1.00 equiv.), *N*-(3-buten-1-yl)phthalimide (67.8 mg, 0.33 mmol, 1.10 equiv.), trifluoroacetic acid (22.3 µL, 0.30 mmol, 1.00 equiv.) and paraformaldehyde (9.01 mg, 0.30 mmol, 1.00 equiv.). Purification by flash column chromatography (dichloromethane/DMA-mix 95:5 to 0:100) gave the title compound (43.2 mg, 0.128 mmol, 43%) as a yellow oil.

^{1}H NMR (400 MHz, CDCl$_3$) δ 7.86 – 7.79 (m, 2H), 7.74 – 7.64 (m, 2H), 7.31 – 7.27 (m, 2H), 7.22 – 7.15 (m, 3H), 3.67 (t, *J* = 7.2 Hz, 2H), 2.95 – 2.81 (m, 4H), 2.65 (dd, *J* = 15.9, 8.5 Hz, 2H), 1.72 – 1.61 (m, 2H), 1.57 (dt, *J* = 15.0, 7.5 Hz, 2H), 1.42 – 1.28 (m, 2H). *The NH proton was not observed.*
^{13}C NMR (101 MHz, CDCl$_3$) δ 168.6 (2C), 139.7 (2C), 134.0 (2C), 132.3, 128.8 (2C), 128.7 (2C), 126.4, 123.3 (2C), 51.0, 49.5, 38.0, 36.0, 29.2, 28.6, 24.7.
HRMS (ESI$^+$): exact mass calculated for $[M+H]^+$ ($C_{21}H_{25}N_2O_2$) requires *m/z* 337.1911, found *m/z* 337.1909.
IR (neat) ν_{max}: 2931, 2858, 1707, 1650, 1612, 1548, 1370, 746, 701 cm^{-1}.

Diethyl (9-(phenethylamino)nonyl)phosphonate (1.27)

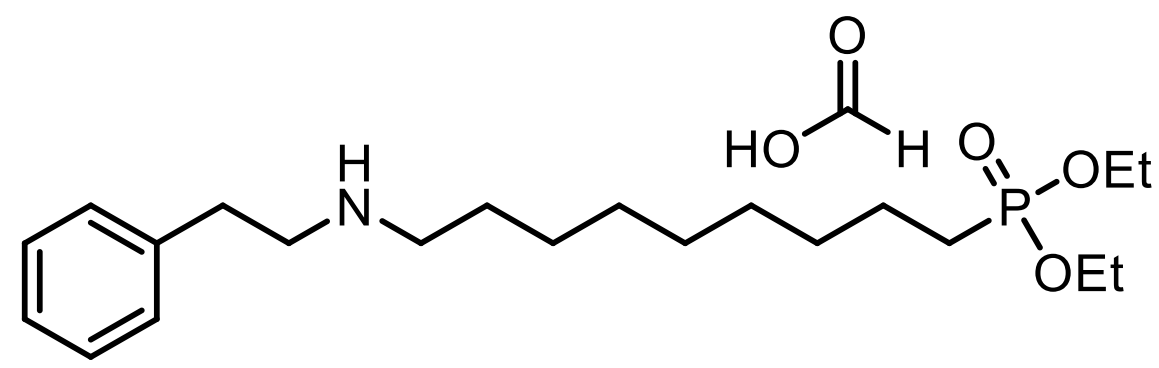

free amine
$C_{21}H_{38}NO_3P$
MW: 383.5128

Prepared following general procedure **4** using *N*-methyl-2-phenylethan-1-amine (43.6 µL, 0.30 mmol, 1.00 equiv.), diethyl oct-7-en-1-ylphosphonate (81.9 mg, 0.33 mmol, 1.10 equiv.), paraformaldehyde (9.0 mg, 0.30 mmol, 1.00 equiv.), trifluoroacetic acid (23.0 µL, 0.30 mmol, 1.00 equiv.) and HFIP (0.25 mL). Purification by preparative high-performance liquid chromatography (water + 0.1% formic acid/acetonitrile 95:5 to 60:40) gave the title compound as a formate salt (94.1 mg, 0.219 mmol, 73%) as a yellow oil.

^{1}H NMR (400 MHz, $CDCl_3$) δ 8.44 (s, 1H), 7.35 – 7.26 (m, 2H), 7.25 – 7.17 (m, 3H), 4.17 – 3.99 (m, 6H), 3.22 – 3.01 (m, 5H), 2.96 – 2.81 (m, 3H), 1.86 – 1.64 (m, 6H), 1.60 – 1.45 (m, 2H), 1.31 (t, *J* = 7.1 Hz, 6H), 1.29 – 1.19 (m, 4H).

^{13}C NMR (101 MHz, $CDCl_3$) δ 167.6 (*formate*), 136.9, 129.0 (2C), 128.8 (2C), 127.2, 61.6 (d, *J* = 6.6 Hz, 2C), 49.1, 47.8, 32.6, 30.7, 30.5, 29.1 (d, *J* = 12.3 Hz), 26.8, 26.4, 26.2, 25.0, 22.5 (d, *J* = 5.7 Hz), 16.6 (d, *J* = 6.2 Hz, 2C).

HRMS (ESI$^+$): exact mass calculated for $[M+H]^+$ ($C_{21}H_{39}NO_3P^+$) requires *m/z* 384.2662, found *m/z* 384.2670.

IR (neat) ν_{max}: 3254, 2956, 2845, 1600, 1533, 1464, 1311, 1254, 1063 cm^{-1}.

_N_-Phenethyl-9-(phenylsulfonyl)nonan-1-amine (1.28)

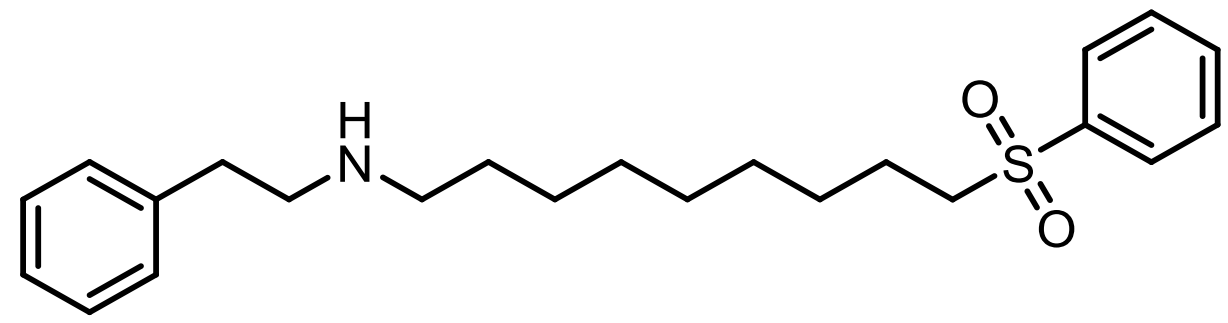

$C_{23}H_{33}NO_2S$
MW: 387.5820

Prepared following general procedure **4** using *N*-methyl-2-phenylethan-1-amine (43.6 µL, 0.30 mmol, 1.00 equiv.), (oct-7-en-1-ylsulfonyl)benzene (83.3 mg, 0.33 mmol, 1.10 equiv.), paraformaldehyde (9.0 mg, 0.30 mmol, 1.00 equiv.), trifluoroacetic acid (23.0 µL, 0.30 mmol, 1.00 equiv.) and HFIP (0.25 mL). Purification by flash column chromatography (dichloromethane/DMA-mix 95:5 to 0:100) gave the title compound (81.1 mg, 0.22 mmol, 70%) as a yellow oil.

^{1}H NMR (400 MHz, $CDCl_3$) δ7.93 – 7.89 (m, 2H), 7.68 – 7.63 (m, 1H), 7.60 – 7.54 (m, 2H), 7.32 – 7.27 (m, 2H), 7.23 – 7.18 (m, 3H), 3.12 – 3.03 (m, 2H), 2.93 – 2.85 (m, 2H), 2.85 – 2.78 (m, 2H), 2.65 – 2.58 (m, 2H), 1.75 – 1.64 (m, 3H), 1.49 – 1.43 (m, 2H), 1.32 (dd, *J* = 14.1, 7.0 Hz, 2H), 1.27 – 1.20 (m, 8H).

^{13}C NMR (151 MHz, $CDCl_3$) δ 140.1, 139.4, 133.7, 129.4 (2C), 128.9 (2C), 128.6 (2C), 128.2 (2C), 126.3, 56.5, 51.3, 49.9, 36.3, 30.0, 29.5, 29.3, 29.1, 28.4, 27.4, 22.8.

HRMS (ESI$^+$): exact mass calculated for $[M+H]^+$ ($C_{23}H_{43}NO_2S^+$) requires *m/z* 388.2305, found *m/z* 388.2308.

IR (neat) ν_{max}: 3289, 3201, 3033, 2978, 2289, 1732, 1622, 1509, 1288, 1072, 1022, 933 cm^{-1}.

_N_,_N_-Diethyl-N^{11}-phenethylundecane-1,11-diamine (1.29)

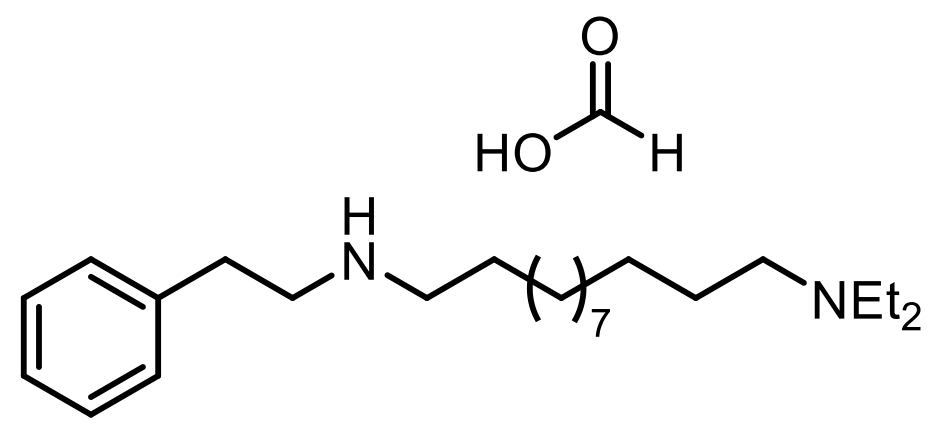

free amine
$C_{24}H_{44}N_2$
MW: 360.6300

Prepared following general procedure **4** using *N*-methyl-phenethylamine (43.6 µL, 0.300 mmol, 1.00 equiv.), *N*,*N*-diethyldec-9-en-1-amine (74.4 mg, 0.330 mmol, 1.10 equiv.), trifluoroacetic acid (22.3 µL, 0.300 mmol, 1.00 equiv.) and paraformaldehyde (9.01 mg, 0.300 mmol, 1.00 equiv.). Purification by preparative high-performance liquid chromatography (water + 0.1% formic acid/acetonitrile 95:5 to 60:40) gave the title compound as a formate salt (88.1 mg, 0.221 mmol, 75%) as a pale-yellow oil.

^{1}H NMR (400 MHz, $CDCl_3$) δ 8.59 (s, 1H), 7.31 – 7.27 (m, 2H), 7.24 – 7.18 (m, 3H), 3.14 – 2.94 (m, 7H), 2.94 – 2.70 (m, 4H), 1.67 (d, *J* = 5.7 Hz, 4H), 1.38 – 1.09 (m, 24H).

^{13}C NMR (101 MHz, $CDCl_3$) δ 168.7, 137.7, 128.9 (2C), 128.8 (2C), 127.0, 50.9, 49.4, 48.2, 45.7 (2C), 33.4, 29.8, 29.4 (2C), 29.3, 29.2, 29.1, 27.1, 27.0, 26.9, 23.6, 8.8 (2C).

HRMS (ESI$^+$): exact mass calculated for $[M+H]^+$ ($C_{24}H_{45}N_2$) requires *m/z* 361.3577, found *m/z* 361.3577.

IR (neat) ν_{max}: 2923, 2852, 1584, 1494, 1369, 751, 700 cm^{-1}.

7-(Phenethylamino)heptan-1-ol (1.30)

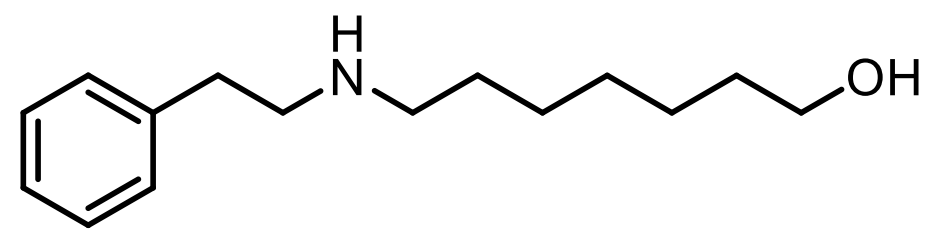

$C_{15}H_{25}NO$
MW: 235.3710

Prepared following general procedure **4** using *N*-methyl-2-phenylethan-1-amine (43.6 µL, 0.30 mmol, 1.00 equiv.), hex-5-en-1-ol (33.0 mg, 0.33 mmol, 1.10 equiv.), paraformaldehyde (9.0 mg, 0.30 mmol, 1.00 equiv.), trifluoroacetic acid (23.0 µL, 0.30 mmol, 1.00 equiv.) and HFIP (0.25 mL). Purification by flash column chromatography (dichloromethane/DMA-mix 95:5 to 0:100) gave the title compound (46.7 mg, 0.20 mmol, 66%) as a yellow oil.

^{1}H NMR (400 MHz, $CDCl_3$) δ 7.32 – 7.26 (m, 2H), 7.24 – 7.17 (m, 3H), 3.62 (t, *J* = 6.6 Hz, 2H), 2.90 – 2.85 (m, 2H), 2.84 – 2.77 (m, 2H), 2.61 (t, *J* = 7.6 Hz, 2H), 1.60 – 1.43 (m, 4H), 1.38 – 1.25 (m, 6H). *The NH and OH protons were not observed.*

^{13}C NMR (101 MHz, $CDCl_3$) δ 140.2, 128.9 (2C), 128.6 (2C), 126.3, 63.1, 51.4, 50.0, 36.5, 32.9, 30.1, 29.4, 27.4, 25.8.

HRMS (ESI$^+$): exact mass calculated for $[M+H]^+$ ($C_{15}H_{26}NO^+$) requires *m/z* 236.2009, found *m/z* 236.2012.

IR (neat) ν_{max}: 3336, 3012, 2976, 2888, 1532, 1467, 1340, 1211, 1053, 985, 786 cm^{-1}.

_N,N_-Dimethyl-12-(phenethylamino)dodecanamide (1.31)

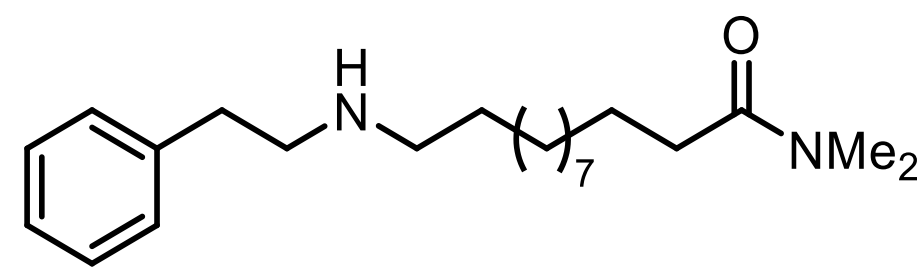

$C_{22}H_{38}N_2O$
MW: 346.5590

Prepared following general procedure **4** using *N*-methyl-phenethylamine (43.6 µL, 0.300 mmol, 1.00 equiv.), *N,N*-dimethylundec-10-enamide (69.7 mg, 0.330 mmol, 1.10 equiv.), trifluoroacetic acid (22.3 µL, 0.300 mmol, 1.00 equiv.) and paraformaldehyde (9.01 mg, 0.300 mmol, 1.00 equiv.). Purification by flash column chromatography (dichloromethane/DMA-mix 95:5 to 0:100) gave the title compound (80.2 mg, 0.222 mmol, 74%) as a colourless oil.

^{1}H NMR (400 MHz, CDCl$_3$) δ 7.31 – 7.26 (m, 2H), 7.23 – 7.16 (m, 3H), 3.22 – 3.04 (m, 3H), 2.99 (s, 3H), 2.93 (s, 3H), 2.91 – 2.80 (m, 1H), 2.32 – 2.24 (m, 2H), 1.79 (br s, 1H), 1.66 – 1.55 (m, 2H), 1.38 – 1.14 (m, 18H).

^{13}C NMR (101 MHz, CDCl$_3$) δ 173.4, 140.0, 128.8 (2C), 128.6 (2C), 126.3, 51.2, 49.9, 37.4, 36.2, 35.5, 33.5, 29.8, 29.6 (6C), 27.4, 25.3.

HRMS (ESI$^+$): exact mass calculated for $[M+H]^+$ ($C_{22}H_{39}N_2O$) requires *m/z* 347.3057, found *m/z* 347.3055.

IR (neat) ν_{max}: 2922, 2851, 1643, 1462, 1395, 1265, 1141, 747, 699 cm^{-1}.

8-(Phenethylamino)-1-phenyloctan-1-one (1.32)

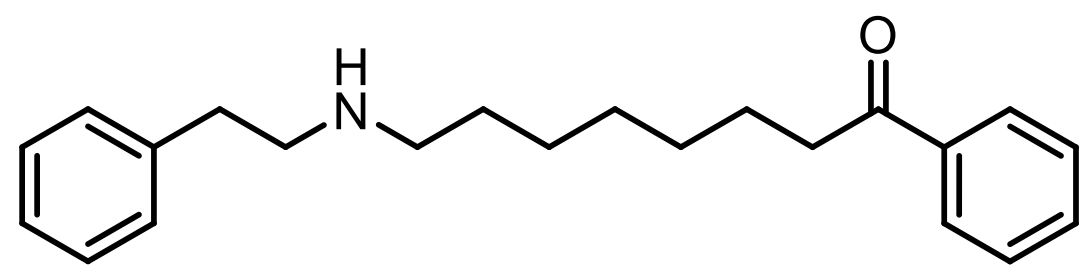

$C_{22}H_{29}NO$
MW: 323.4800

Prepared following general procedure **4** using *N*-methyl-2-phenylethan-1-amine (43.6 µL, 0.300 mmol, 1.00 equiv.), 1-phenylhept-6-en-1-one (62.0 mg, 0.330 mmol, 1.10 equiv.), trifluoroacetic acid (22.3 µL, 0.300 mmol, 1.00 equiv.) and paraformaldehyde (9.0 mg, 0.30 mmol, 1.0 equiv.). Purification by flash column chromatography (dichloromethane/DMA-mix 95:5 to 0:100) gave the title compound (79.4 mg, 0.250 mmol, 82%) as a yellow oil.

^{1}H NMR (600 MHz, $CDCl_3$) δ 7.97 – 7.94 (m, 2H), 7.55 (ddd, *J* = 8.6, 2.4, 1.2 Hz, 1H), 7.48 – 7.42 (m, 2H), 7.31 – 7.27 (m, 2H), 7.21 (d, *J* = 7.5 Hz, 3H), 2.97 – 2.92 (m, 2H), 2.91 – 2.86 (m, 2H), 2.85 – 2.79 (m, 2H), 2.64 – 2.61 (m, *J* = 7.3 Hz, 2H), 1.95 – 1.77 (br s, 1H), 1.75 –1.69 (m, 2H), 1.51 – 1.46 (m, 2H), 1.39 – 1.28 (m, 6H).

^{13}C NMR (151 MHz, $CDCl_3$) δ 200.7, 140.1, 137.2 (2C), 133.0, 128.9 (2C), 128.7, 128.6 (2C), 128.2 (2C), 126.3, 51.3, 50.0, 38.7, 36.4, 30.0, 29.5, 29.4, 27.3, 24.4.

HRMS (ESI^{+}): exact mass calculated for $[M+H]^{+}$ ($C_{22}H_{30}NO$) requires *m/z* 324.2322, found *m/z* 324.2320.

IR (neat) ν_{max}: 3315, 3222, 3011, 2983, 2988, 1722, 1520, 1465, 1352, 1150, 1091, 812 cm^{-1}.

3-(3-(Phenethylamino)propyl)benzaldehyde (1.33)

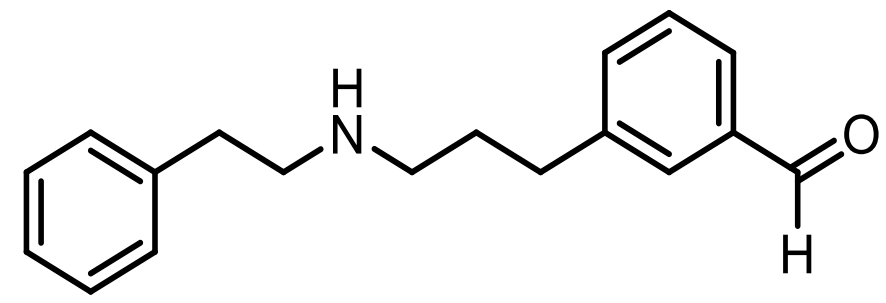

$C_{18}H_{21}NO$
MW: 267.3720

Prepared following general procedure **4** using *N*-methyl-2-phenylethan-1-amine (43.6 µL, 0.30 mmol, 1.00 equiv.), 3-vinylbenzaldehyde (43.6 mg, 0.33 mmol, 1.10 equiv.), paraformaldehyde (9.0 mg, 0.30 mmol, 1.0 equiv.), trifluoroacetic acid (23.0 µL, 0.30 mmol, 1.00 equiv.) and HFIP (0.25 mL). Purification by flash column chromatography (dichloromethane/DMA-mix 95:5 to 0:100) the title compound (48.1 mg, 0.18 mmol, 60%, (79% based on recovered starting material)) as a yellow oil.

^{1}H NMR (600 MHz, $CDCl_3$) δ 9.99 (s, 1H), 7.71 – 7.69 (m, 1H), 7.68 – 7.66 (m, 1H), 7.45 – 7.40 (m, 2H), 7.33 – 7.26 (m, 2H), 7.24 – 7.16 (m, 3H), 2.90 – 2.86 (m, 2H), 2.81 (t, *J* = 6.8 Hz, 2H), 2.72 (t, *J* = 7.6 Hz, 2H), 2.66 (t, *J* = 7.2 Hz, 2H), 1.86 – 1.80 (m, 2H), 1.54 (br s, 1H).
^{13}C NMR (151 MHz, $CDCl_3$) δ 192.7, 143.3, 140.1, 136.7, 134.8, 129.5, 129.2, 128.9 (2C), 128.7 (2C), 127.8, 126.4, 51.2, 49.2, 36.5, 33.4, 31.5.
HRMS (ESI$^+$): exact mass calculated for $[M+H]^+$ ($C_{18}H_{22}NO^+$) requires *m/z* 268.1696, found *m/z* 268.1698.
IR (neat) ν_{max}: 3282, 2955, 2892, 1732, 1701, 1598, 1444, 1387, 1066, 953, 912 cm^{-1}.

3-(Benzo[*b*]thiophen-3-yl)-*N*-phenethylpropan-1-amine (1.34)

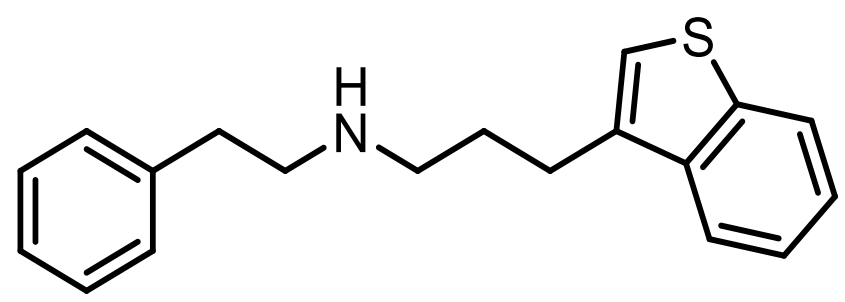

$C_{19}H_{21}NS$
MW: 295.4440

Prepared following general procedure **4** using *N*-methyl-2-phenylethan-1-amine (60.8 mg, 0.45 mmol, 1.00 equiv.), 3-vinylbenzo[b]thiophene (50.0 mg, 0.30 mmol, 1.00 equiv.), paraformaldehyde (13.5 mg, 0.450 mmol, 1.50 equiv.), trifluoroacetic acid (33.4 µL, 0.45 mmol, 1.50 equiv.) and HFIP (0.25 mL). The reaction was conducted at 50 °C. Purification by flash column chromatography (dichloromethane/DMA-mix 95:5 to 0:100) gave the title compound (45.2 mg, 0.153 mmol, 51%) as a yellow oil.

^{1}H NMR (600 MHz, CDCl$_3$) δ7.83 (dd, *J* = 7.4, 0.7 Hz, 1H), 7.71 (d, *J* = 7.7 Hz, 1H), 7.38 – 7.30 (m, 2H), 7.29 – 7.26 (m, 2H), 7.23 – 7.17 (m, 3H), 7.09 (s, 1H), 3.02 – 2.94 (m, 4H), 2.88 (t, *J* = 7.5 Hz, 2H), 2.85 – 2.82 (m, 2H), 2.15 – 2.05 (m, 2H). *The NH proton was not observed.*

^{13}C NMR (151 MHz, CDCl$_3$) δ 140.71, 140.66, 138.9 (2C), 128.83 (2C), 128.81, 126.7 (2C), 124.4 (2C), 124.1, 123.0, 121.8, 50.5, 48.8, 34.8, 27.8, 26.1.

HRMS (ESI$^+$): exact mass calculated for $[M+H]^+$ ($C_{19}H_{22}NS^+$) requires *m/z* 296.1467, found *m/z* 296.1465.

IR (neat) ν_{max}: 3444, 3277, 3033, 2996, 2855, 1555, 1432, 1331, 1289, 1055, 987 cm^{-1}.

3-(Benzofuran-3-yl)-*N*-phenethylpropan-1-amine (1.35)

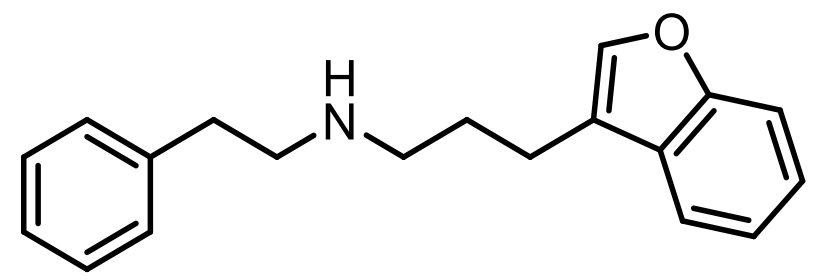

$C_{19}H_{21}NO$
MW: 279.3830

Prepared following general procedure **4** using *N*-methyl-2-phenylethan-1-amine (60.8 mg, 0.45 mmol, 1.00 equiv.), 3-vinylbenzofuran (43.2 mg, 0.300 mmol, 1.00 equiv.), paraformaldehyde (13.5 mg, 0.450 mmol, 1.50 equiv.), trifluoroacetic acid (33.4 µL, 0.45 mmol, 1.50 equiv.) and HFIP (0.25 mL). The reaction was conducted at 50 °C. Purification by flash column chromatography (dichloromethane/DMA-mix 95:5 to 0:100) gave the title compound (44.4 mg, 0.159 mmol, 53%) as a yellow oil.

^{1}H NMR (400 MHz, $CDCl_3$) δ 7.84 – 7.79 (m, 1H), 7.72 – 7.66 (m, 1H), 7.38 – 7.24 (m, 4H), 7.20 – 7.15 (m, 3H), 7.01 (br s, 1H), 2.91 – 2.81 (m, 2H), 2.84 – 2.76 (m, 4H), 2.73 – 2.67 (m, 2H), 1.99 – 1.86 (m, 2H), 1.67 (br s, 1H).

^{13}C NMR (151 MHz, $CDCl_3$) δ 141.2, 140.3, 138.1 (2C), 128.6 (2C), 128.4, 126.5 (2C), 124.1 (2C), 124.0, 123.8, 121.7, 50.4, 46.8, 33.6, 27.5, 26.0.

HRMS (ESI$^+$): exact mass calculated for $[M+H]^+$ ($C_{19}H_{22}NO^+$) requires *m/z* 280.1696, found *m/z* 280.1700.

IR (neat) ν_{max}: 3211, 3030, 2967, 2841, 1522, 1456, 1321, 1187, 1098, 961 cm^{-1}.

3-Phenyl-*N*-(1-phenylethyl)propan-1-amine (1.36)

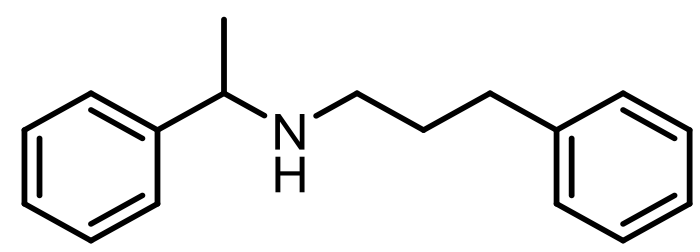

$C_{17}H_{21}N$
MW: 239.3620

Prepared following general procedure **4** using *N*-methyl-1-phenylethan-1-amine (40.6 mg, 0.30 mmol, 1.00 equiv.), styrene (34.5 µL, 0.33 mmol, 1.10 equiv.), trifluoroacetic acid (22.3 µL, 0.30 mmol, 1.00 equiv.) and paraformaldehyde (9.0 mg, 0.30 mmol, 1.0 equiv.). Purification by flash column chromatography (dichloromethane/DMA-mix 95:5 to 0:100) gave the title compound (50.3 mg, 0.210 mmol, 70%) as a yellow oil.

^{1}H NMR (700 MHz, $CDCl_3$) δ 7.35 – 7.28 (m, 4H), 7.28 – 7.22 (m, 3H), 7.19 – 7.13 (m, 3H), 3.75 (q, *J* = 6.6 Hz, 1H), 2.70 – 2.61 (m, 1H), 2.61 – 2.52 (m, 2H), 2.51 – 2.44 (m, 1H), 1.85 – 1.73 (m, 2H), 1.43 (br s, 1H), 1.34 (d, *J* = 6.6 Hz, 3H).

^{13}C NMR (176 MHz, $CDCl_3$) δ 142.4 128.6 (2C), 128.5 (3C), 128.4 (3C), 127.0, 126.7, 125.9, 58.5, 47.5, 33.8, 32.0, 24.5.

The spectroscopic properties are consistent with the data available in the literature.[18]

3-(4-Bromophenyl)-*N*-phenethylpropan-1-amine (1.37)

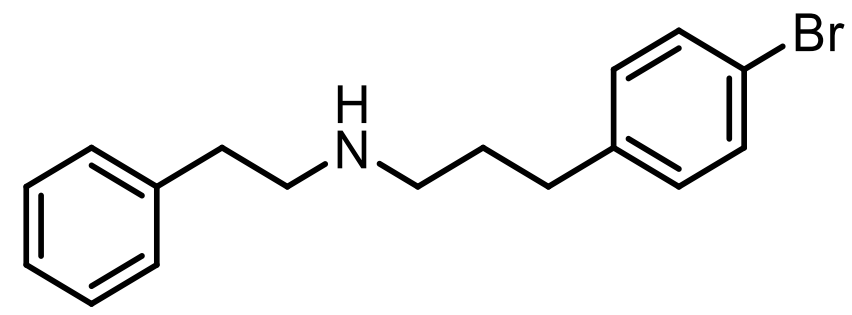

$C_{17}H_{20}BrN$
MW: 318.2580

Prepared following general procedure **4** using *N*-methyl-2-phenylethan-1-amine (60.8 mg, 0.45 mmol, 1.00 equiv.), 4-bromostyrene (39.0 µL, 0.300 mmol, 1.00 equiv.), paraformaldehyde (13.5 mg, 0.450 mmol, 1.50 equiv.), trifluoroacetic acid (33.4 µL, 0.45 mmol, 1.50 equiv.) and HFIP (0.25 mL). The reaction was conducted at 50 °C. Purification by flash column chromatography (dichloromethane/DMA-mix 95:5 to 0:100) gave the title compound (50.7 mg, 0.160 mmol, 53%) as a yellow oil.

^{1}H NMR (600 MHz, $CDCl_3$) δ 7.33 – 7.20 (m, 9H), 2.91 – 2.85 (m, 2H), 2.83 – 2.77 (m, 2H), 2.68 – 2.60 (m, 4H), 2.20 – 1.91 (br s, 1H), 1.86 – 1.78 (m, 2H).

^{13}C NMR (151 MHz, $CDCl_3$) δ 141.1, 140.1, 131.5, 130.3, 128.9 (2C), 128.7 (2C), 126.4 (2C), 119.6 (2C), 51.2, 49.2, 36.4, 33.1, 31.5.

HRMS (ESI$^+$): exact mass calculated for $[M+H]^+$ ($C_{17}H_{21}{}^{79}BrN$) requires *m/z* 318.0852, found *m/z* 318.0851.

IR (neat) ν_{max}: 3288, 3240, 3001, 2976, 2836, 1477, 1391, 1336, 1111, 1079, 1005, 976, 867 cm^{-1}.

3-(4-Chlorophenyl)-*N*-phenethylpropan-1-amine (1.38)

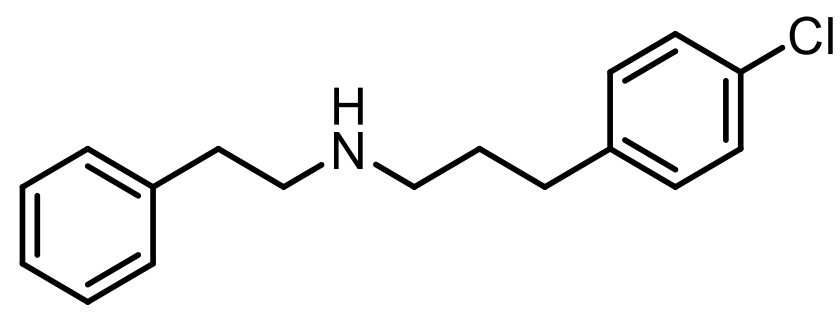

$C_{17}H_{20}ClN$
MW: 273.8040

Prepared following general procedure **4** using *N*-methyl-2-phenylethan-1-amine (60.8 mg, 0.45 mmol, 1.00 equiv.), 4-chlorostyrene (36.0 µL, 0.300 mmol, 1.00 equiv.), paraformaldehyde (13.5 mg, 0.450 mmol, 1.50 equiv.), trifluoroacetic acid (33.4 µL, 0.45 mmol, 1.50 equiv.) and HFIP (0.25 mL). The reaction was conducted at 50 °C. Purification by flash column chromatography (dichloromethane/DMA-mix 95:5 to 0:100) gave the title compound (45.2 mg, 0.165 mmol, 55%) as a yellow oil.

^{1}H NMR (600 MHz, $CDCl_3$) δ 7.34 – 7.30 (m, 2H), 7.27 – 7.23 (m, 2H), 7.19 – 7.13 (m, 3H), 6.99 – 6.94 (m, 2H), 2.87 – 2.79 (m, 2H), 2.78 – 2.72 (m, 2H), 2.61 – 2.57 (m, 2H), 2.57 – 2.50 (m, 2H), 1.77 – 1.69 (m, 2H), 1.55 (br s, 1H).
^{13}C NMR (151 MHz, $CDCl_3$) δ 141.8, 139.4, 130.2, 129.2 (2C), 128.3 (2C), 127.9 (2C), 125.6, 118.9 (2C), 51.4, 47.3, 36.4, 34.0, 28.5.
HRMS (ESI$^+$): exact mass calculated for $[M+H]^+$ ($C_{17}H_{21}{}^{35}ClN$) requires *m/z* 274.1357, found *m/z* 274.1357.
IR (neat) ν_{max}: 3275, 2967, 2843, 1589, 1467, 1353, 1289, 1177, 1083, 968 cm^{-1}.

2-(3-((*tert*-Butoxycarbonyl)(phenethyl)amino)propyl)benzoic acid (1.39)

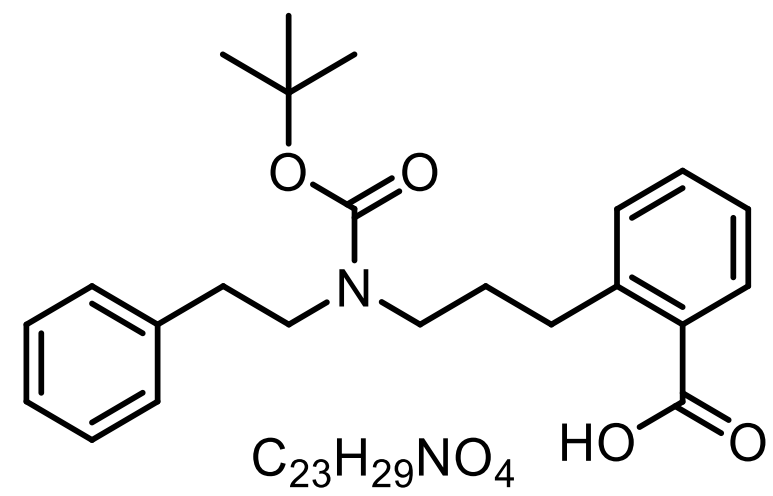

$C_{23}H_{29}NO_4$
MW: 383.4880

Prepared following general procedure **4** using *N*-methyl-2-phenylethan-1-amine (43.6 µL, 0.30 mmol, 1.00 equiv.), 2-vinylbenzoic acid (48.9 mg, 0.33 mmol, 1.10 equiv.), paraformaldehyde (9.0 mg, 0.30 mmol, 1.0 equiv.), trifluoroacetic acid (23.0 µL, 0.30 mmol, 1.00 equiv.) and HFIP (0.25 mL).

The crude mixture was then dissolved in anhydrous dichloromethane (1.5 mL), followed by addition of triethylamine (84 µL, 0.60 mmol, 2.0 equiv.) and di-tert-butyldicarbonate (131 mg, 0.60 mmol, 2.0 equiv.). The solution was stirred for 5 hours at 23 °C, diluted with 5 mL of dichloromethane and the reaction mixture was basified with 5 mL of saturated aqueous solution of $NaHCO_3$. The resulting biphasic mixture was separated, and the aqueous phase was extracted with dichloromethane (3 x 20 mL). The filtrate was concentrated under reduced pressure to afford the crude product. Purification by flash column chromatography (heptane/ethyl acetate 95:5 to 0:100) gave the title compound (63.4 mg, 0.17 mmol, 55%) as a yellow oil.

Rotameric effects are visible in the measured NMR spectra.

^{1}H NMR (400 MHz, $CDCl_3$) δ 8.02 (d, *J* = 7.2 Hz, 1H), 7.52 – 7.43 (m, 1H), 7.31 – 7.23 (m, 4H), 7.24 – 7.10 (m, 3H), 3.34 (m, 3.5H), 2.99 (m, 2H), 2.90 – 2.74 (m, 2.5H), 1.85 (m, 2H), 1.44 (br s, 9H). *The COOH proton was not observed.*

^{13}C NMR (101 MHz, $CDCl_3$) δ 171.8, [156.1, 155.7], 145.0, 139.5, 133.0 (2C), [131.9, 131.2], 129.0 (2C), 128.6 (2C), 126.3 (2C), 126.2, [79.5, 79.4], 49.3 (2C), 34.2, 32.2, 32.1, 28.6 (3C).

HRMS (ESI$^+$): exact mass calculated for $[M+Na]^+$ ($C_{23}H_{29}NO_4Na^+$) requires *m/z* 406.1989, found *m/z* 406.1983.

IR (neat) ν_{max}: 3349, 3256, 2975, 2868, 1733, 1582, 1461, 1322, 1087, 867, 628 cm^{-1}.

2-Methyl-*N*-phenethyl-3-phenylpropan-1-amine (1.40)

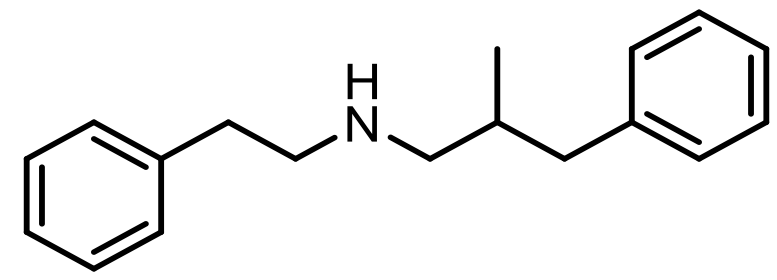

$C_{18}H_{23}N$
MW: 253.1830

Prepared following general procedure **4** using *N*-methyl-2-phenylethan-1-amine (43.6 µL, 0.300 mmol, 1.00 equiv.), (*Z*)-prop-1-en-1-ylbenzene (39.0 µL, 0.300 mmol, 1.00 equiv.), trifluoroacetic acid (22.3 µL, 0.300 mmol, 1.00 equiv.) and paraformaldehyde (9.0 mg, 0.30 mmol, 1.0 equiv.) The reaction was conducted at 50 °C. Purification by flash column chromatography (dichloromethane/DMA-mix 95:5 to 0:100) gave the title compound (33.5 mg, 0.130 mmol, 44%) as a yellow oil.

^{1}H NMR (600 MHz, CDCl$_3$) δ 7.31 – 7.25 (m, 4H), 7.23 – 7.16 (m, 4H), 7.13 – 7.10 (m, 2H), 2.90 – 2.86 (m, 2H), 2.84 – 2.79 (m, 2H), 2.67 (dd, *J* = 13.4, 6.2 Hz, 1H), 2.61 (dd, *J* = 11.8, 6.0 Hz, 1H), 2.49 (dd, *J* = 11.8, 7.2 Hz, 1H), 2.38 (dd, *J* = 13.5, 8.1 Hz, 1H), 2.02 – 1.92 (m, 1H), 0.87 (d, *J* = 6.7 Hz, 3H). *The NH proton was not observed.*

^{13}C NMR (151 MHz, CDCl$_3$) δ 140.7, 139.6, 129.1 (2C), 128.7 (2C), 128.5 (2C), 128.2 (2C), 126.2, 125.9, 55.6, 51.1, 41.5, 36.0, 35.0, 18.0.

HRMS (ESI$^+$): exact mass calculated for $[M+H]^+$ ($C_{18}H_{24}N$) requires *m/z* 254.1903, found *m/z* 254.1905.

IR (neat) ν_{max}: 3273, 3233, 3021, 2978, 2843, 1451, 1377, 1355, 1082, 1052, 950, 864 cm^{-1}.

3-(4-Methoxyphenyl)-*N*-phenethylpropan-1-amine (1.41)

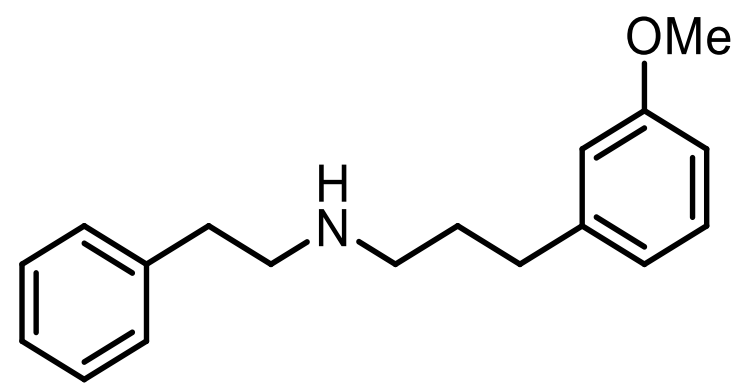

$C_{18}H_{23}NO$
MW: 269.3880

Prepared following general procedure **4** using *N*-methyl-2-phenylethan-1-amine (60.8 mg, 0.45 mmol, 1.00 equiv.), 3-methoxystyrene (40.3 mg, 0.300 mmol, 1.00 equiv.), paraformaldehyde (13.5 mg, 0.450 mmol, 1.50 equiv.), trifluoroacetic acid (33.4 µL, 0.45 mmol, 1.50 equiv.) and HFIP (0.25 mL). The reaction was conducted at 50 °C. Purification by flash column chromatography (dichloromethane/DMA-mix 95:5 to 0:100) gave the title compound (36.4 mg, 0.14 mmol, 45%) as a yellow oil.

^{1}H NMR (700 MHz, $CDCl_3$) δ 7.31 – 7.28 (m, 2H), 7.23 – 7.16 (m, 4H), 6.76 – 6.70 (m, 3H), 3.79 (s, 3H), 2.90 – 2.86 (m, 2H), 2.81 (t, *J* = 7.2 Hz, 2H), 2.68 – 2.65 (m, 2H), 2.61 (dd, *J* = 16.1, 8.4 Hz, 2H), 1.81 (dt, *J* = 14.8, 7.5 Hz, 2H), 1.65 (br s, 1H).

^{13}C NMR (176 MHz, $CDCl_3$) δ 159.8, 143.8, 140.1, 129.4 (2C), 128.9 (2C), 128.6, 126.3, 120.9, 114.3, 111.2, 55.3, 51.2, 49.4, 36.4, 33.8, 31.5.

HRMS (ESI$^+$): exact mass calculated for $[M+H]^+$ ($C_{18}H_{24}NO$) requires *m/z* 270.1852, found *m/z* 270.1854.

IR (neat) ν_{max}: 3333, 3312, 3014, 2957, 2834, 2803, 1422, 1322, 1255, 1100, 1022, 980 cm^{-1}.

3-(3-(Phenethylamino)propyl)benzaldehyde(1.42)

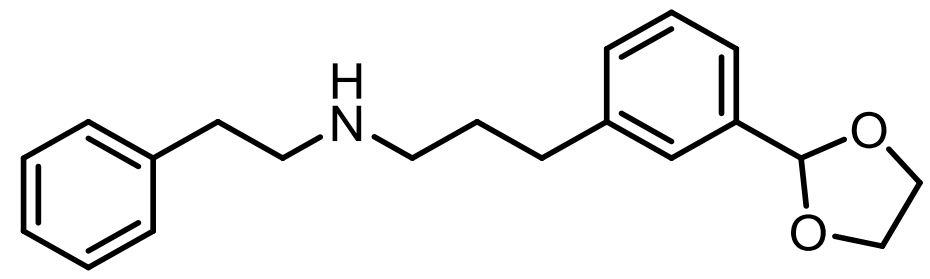

$C_{20}H_{25}NO_2$
MW: 311.4250

Prepared following the slightly modified general procedure **4** using *N*-methyl-2-phenylethan-1-amine (43.6 µL, 0.30 mmol, 1.00 equiv.), 3-vinylbenzaldehyde (43.6 mg, 0.33 mmol, 1.10 equiv.), paraformaldehyde (9.0 mg, 0.30 mmol, 1.0 equiv.), acetic acid (18 µL, 0.30 mmol, 1.00 equiv.) and HFIP (0.25 mL). The reaction was conducted at 50 °C. Purification by flash column chromatography (dichloromethane/DMA-mix 95:5 to 0:100) gave the title compound (43.0 mg, 0.138 mmol, 44%) as a yellow oil.

^{1}H NMR (600 MHz, $CDCl_3$) δ 7.31 – 7.27 (m, 5H), 7.23 – 7.19 (m, 3H), 7.17 – 7.14 (m, 1H), 5.78 (s, 1H), 4.16 – 4.08 (m, 2H), 4.07 – 3.99 (m, 2H), 2.88 (td, *J* = 7.0, 1.1 Hz, 2H), 2.81 (t, *J* = 6.8 Hz, 2H), 2.65 (dt, *J* = 12.0, 7.6 Hz, 4H), 1.88 – 1.78 (m, 2H) 1.64 (br s, 1H).

^{13}C NMR (151 MHz, $CDCl_3$) δ 142.4, 140.1, 138.0, 129.4, 128.9 (2C), 128.6 (2C), 128.5, 126.5, 126.3, 124.2, 104.0, 65.5 (2C), 51.2, 49.4, 36.4, 33.7, 31.5.

HRMS (ESI$^+$): exact mass calculated for $[M+H]^+$ ($C_{20}H_{26}NO_2^+$) requires *m/z* 312.1958, found *m/z* 312.1963.

IR (neat) ν_{max}: 3282, 2955, 2892, 1732, 1701, 1598, 1444, 1387, 1066, 953, 912 cm^{-1}.

7-((*tert*-Butyldimethylsilyl)oxy)-*N*-phenethylheptan-1-amine(1.43)

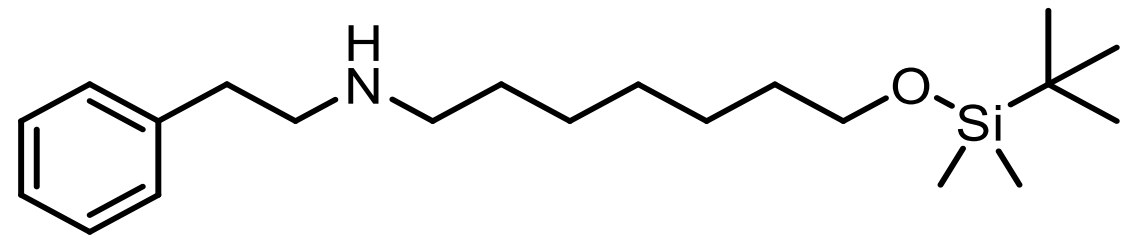

$C_{21}H_{39}NOSi$
MW: 349.6340

Prepared following the slightly modified general procedure **4** using *N*-methyl-2-phenylethan-1-amine (43.6 µL, 0.30 mmol, 1.00 equiv.), *tert*-butyl(hex-5-en-1-yloxy)dimethylsilane (70.6 mg, 0.33 mmol, 1.10 equiv.), paraformaldehyde (9.0 mg, 0.30 mmol, 1.0 equiv.), acetic acid (18 µL, 0.30 mmol, 1.0 equiv.) and HFIP (0.25 mL). Purification by flash column chromatography (dichloromethane/DMA-mix 95:5 to 0:100) gave the title compound (57.4 mg, 0.17 mmol, 55%) as a yellow oil.

^{1}H NMR (400 MHz, $CDCl_3$) δ 7.32 – 7.27 (m, 2H), 7.23 – 7.18 (m, 3H), 3.58 (t, *J* = 6.6 Hz, 2H), 2.89 (dd, *J* = 10.9, 4.2 Hz, 2H), 2.82 (dd, *J* = 10.9, 4.2 Hz, 2H), 2.62 (t, *J* = 7.3 Hz, 2H), 1.48 (m, 4H), 1.27 (m, 6H), 0.89 (s, 9H), 0.04 (s, 6H). *The NH proton was not observed.*

^{13}C NMR (101 MHz, $CDCl_3$) δ 140.1, 128.9 (2C), 128.6 (2C), 126.3, 63.4, 51.3, 50.0, 36.4, 33.0, 30.0, 29.9, 29.5, 26.1 (3C), 25.9, 18.5, -5.1 (2C).

HRMS (ESI^{+}): exact mass calculated for $[M+H]^{+}$ ($C_{21}H_{40}NOSi^{+}$) requires *m/z* 350.2874, found *m/z* 350.2872.

IR (neat) ν_{max}: 3272, 3166, 3000, 2965, 2842, 1522, 1467, 1359, 1221, 1065, 945, 836 cm^{-1}.

N-Methyl-N-(12-(phenethylamino)dodecyl)-2,4,6-tris(trifluoromethyl)benzenesulfonamide(1.44)

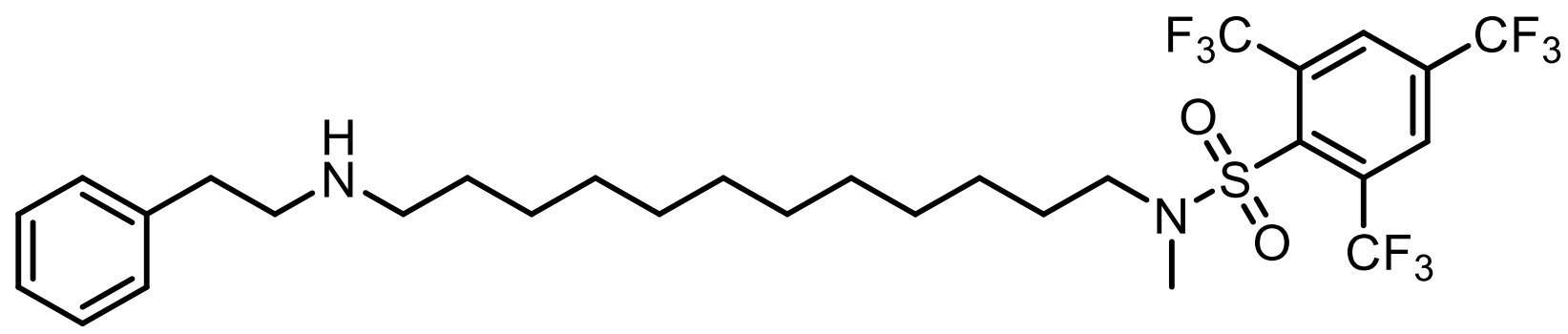

$C_{30}H_{39}F_9N_2O_2S$
MW: 662.6996

Prepared following general procedure **4** using *N*-methyl-2-phenylethan-1-amine (29.0 µL, 0.200 mmol, 1.00 equiv.), *N*-methyl-2,4,6-tris(trifluoromethyl)-*N*-(undec-10-en-1-yl)benzenesulfonamide (115.6 mg, 0.220 mmol, 1.10 equiv.), paraformaldehyde (6.0 mg, 0.20 mmol, 1.0 equiv.), trifluoroacetic acid (15 µL, 0.20 mmol, 1.0 equiv.) and HFIP (0.17 mL). Purification by flash column chromatography (dichloromethane/DMA-mix 95:5 to 0:100) gave the title compound (87.5 mg, 0.132 mmol, 66%) as a yellow oil.

^{1}H NMR (400 MHz, CDCl$_3$) δ 8.26 (s, 2H), 7.32 – 7.26 (m, 2H), 7.24 – 7.11 (m, 3H), 3.32 – 3.22 (m, 2H), 2.92 – 2.78 (m, 4H), 2.74 (s, 3H), 2.65 – 2.57 (m, 2H), 1.70 – 1.60 (m, 2H), 1.51 – 1.41 (m, 3H), 1.37 – 1.21 (m, 16H).

^{13}C NMR (101 MHz, CDCl$_3$) δ 145.2, 140.3, 134.1 (q, *J* = 35.4 Hz, 2C), 133.2 (q, *J* = 34.0 Hz, 2C) 129.2 (3C), 126.3 (3C), 122.2 (q, *J* = 275.7 Hz, 2C), 122.1 (q, *J* = 273.3 Hz), 51.5, 51.4, 50.1, 36.5, 34.4, 30.2, 29.7, 29.6, 29.4 (5C), 27.5, 26.7.

^{19}F NMR (376 MHz, CDCl$_3$) δ –55.75 (6F), –63.56 (3F).

HRMS (ESI$^+$): exact mass calculated for $[M+H]^+$ ($C_{30}H_{40}F_9N_2O_2S^+$) requires *m/z* 663.2661, found *m/z* 663.2666.

IR (neat) ν_{max}: 3367, 3249, 2968, 2875, 1490, 1356, 1201, 1057, 965, 872 cm^{-1}.

(*E*)-3-Cyclohexyl-*N*-phenethylprop-2-en-1-amine (1.45)

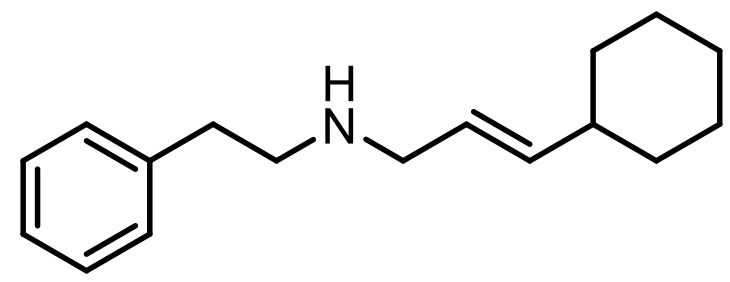

$C_{17}H_{25}N$
MW: 243.3940

Prepared following general procedure **4** using *N*-methyl-1-phenylethan-1-amine (43.6 µL, 0.300 mmol, 1.00 equiv.), ethynylcyclohexane (35.7 mg, 0.330 mmol, 1.10 equiv.), trifluoroacetic acid (22.3 µL, 0.300 mmol, 1.00 equiv.) and paraformaldehyde (9.0 mg, 0.30 mmol, 1.0 equiv.). Purification by flash column chromatography (dichloromethane/DMA-mix 95:5 to 0:100) gave the title compound (35.0 mg, 0.140 mmol, 48%) as a yellow oil.

^{1}H NMR (600 MHz, $CDCl_3$) δ 7.31 – 7.28 (m, 2H), 7.22 – 7.19 (m, 3H), 5.52 (dd, *J* = 15.5, 6.4 Hz, 1H), 5.48 – 5.41 (m, 1H), 3.21 (d, *J* = 6.1 Hz, 2H), 2.90 – 2.86 (m, 2H), 2.85 – 2.81 (m, *J* = 6.7 Hz, 2H), 1.92 (ddd, *J* = 10.9, 9.1, 3.2 Hz, 1H), 1.80 (s, 1H), 1.74 – 1.67 (m, 5H), 1.66 – 1.61 (m, 1H), 1.29 – 1.10 (m, 2H), 1.09 – 1.01 (m, *J* = 13.4, 10.0 Hz, 2H).

^{13}C NMR (151 MHz, $CDCl_3$) δ 140.0, 139.0, 128.7 (2C), 128.5, 126.1, 125.0 (2C), 51.4, 50.4, 40.4, 36.2 (2C), 32.9 (2C), 26.2, 26.0.

HRMS (ESI$^+$): exact mass calculated for $[M+H]^+$ ($C_{17}H_{26}N$) requires *m/z* 244.2060, found *m/z* 244.2062.

IR (neat) ν_{max}: 3243, 3053, 2977, 2939, 2853, 1452, 1358, 1222, 1188, 1079, 988, 799 cm^{-1}.

(*E*)-*N*-(2-Phenylpropyl)-3-(*p*-tolyl)prop-2-en-1-amine (1.46)

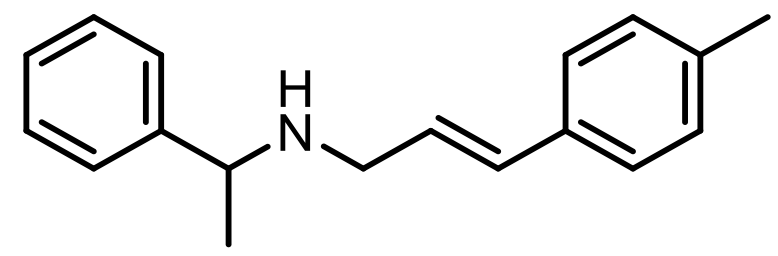

$C_{18}H_{21}N$
MW: 251.3730

Prepared following general procedure **4** using *N*-methyl-1-phenylethan-1-amine (43.6 µL, 0.300 mmol, 1.00 equiv.), 1-ethynyl-4-methylbenzene (41.8 µL, 0.330 mmol, 1.10 equiv.), trifluoroacetic acid (22.3 µL, 0.300 mmol, 1.00 equiv.) and paraformaldehyde (9.0 mg, 0.30 mmol, 1.0 equiv.). Purification by flash column chromatography (dichloromethane/DMA-mix 95:5 to 0:100) gave the title compound (34.0 mg, 0.140 mmol, 45%) as a yellow oil.

^{1}H NMR (600 MHz, $CDCl_3$) δ 7.38 – 7.32 (m, 3H), 7.27 – 7.23 (m, 4H), 7.11 (d, *J* = 7.8 Hz, 3H), 6.43 (dt, *J* = 16.0, 1.7 Hz, 1H), 6.23 (dt, *J* = 15.8, 6.4 Hz, 1H), 3.86 (q, *J* = 6.6 Hz, 1H), 3.25 (td, *J* = 4.5, 2.0 Hz, 2H), 2.33 (s, 3H), 1.39 (d, *J* = 6.6 Hz, 3H).

^{13}C NMR (151 MHz, $CDCl_3$) δ 145.6, 137.2, 134.5, 131.2, 129.4 (2C), 128.6 (2C), 127.6, 127.1, 126.8 (2C), 126.3 (2C), 57.7, 49.9, 24.4, 21.3.

HRMS (ESI$^+$): exact mass calculated for $[M+H]^+$ ($C_{18}H_{22}N$) requires *m/z* 252.1747, found *m/z* 252.1745.

IR (neat) ν_{max}: 3250, 3035, 2972, 2956, 2866, 1456, 1372, 1276, 1200, 1112, 1023, 877 cm^{-1}.

(*E*)-*N*-(1-Phenylethyl)hept-2-en-1-amine (1.47)

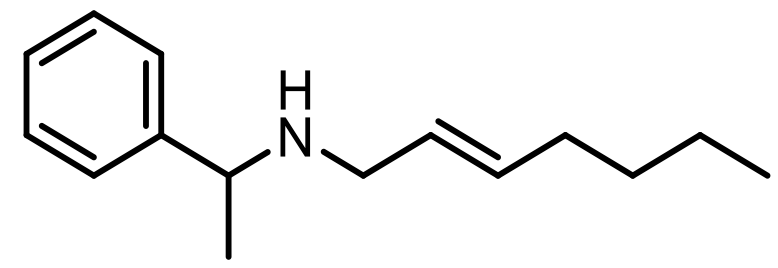

$C_{15}H_{23}N$
MW: 217.3560

Prepared following general procedure **4** using *N*-methyl-1-phenylethanamine (38.7 µL, 0.300 mmol, 1.00 equiv.), 1-hexyne (37.9 µL, 0.330 mmol, 1.10 equiv.), trifluoroacetic acid (22.3 µL, 0.300 mmol, 1.00 equiv.) and paraformaldehyde (9.01 mg, 0.300 mmol, 1.00 equiv.). Purification by flash column chromatography (dichloromethane/DMA-mix 95:5 to 0:100) gave the title compound (33.7 mg, 0.155 mmol, 52%) as a colourless oil.

^{1}H NMR (400 MHz, $CDCl_3$) δ 7.35 – 7.28 (m, 4H), 7.24 – 7.21 (m, 1H), 5.55 – 5.43 (m, 2H), 3.79 (q, *J* = 6.6 Hz, 1H), 3.11 – 2.97 (m, 2H), 2.00 (dd, *J* = 12.2, 7.1 Hz, 2H), 1.41 – 1.20 (m, 8H), 0.96 – 0.78 (m, 3H).

^{13}C NMR (101 MHz, $CDCl_3$) δ 145.7, 133.0, 128.6 (2C), 128.2, 127.0, 126.8 (2C), 57.7, 49.7, 32.2, 31.6, 24.3, 22.5, 14.1.

HRMS (ESI^+): exact mass calculated for $[M+H]^+$ ($C_{15}H_{24}N$) requires *m/z* 218.1903, found *m/z* 219.1901.

IR (neat) ν_{max}: 2957, 2871, 1451, 1368, 1119, 970, 760, 699 cm^{-1}.

(*E*)-*N*-(1-Phenylethyl)undec-2-en-1-amine (1.48)

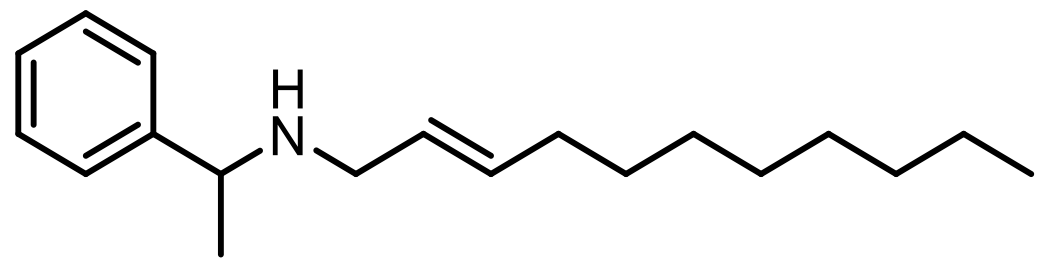

$C_{19}H_{31}N$
MW: 273.4640

Prepared following general procedure **4** using *N*-methyl-1-phenylethanamine (38.7 µL, 0.300 mmol, 1.00 equiv.), 1-decyne (48.0 mg, 0.330 mmol, 1.10 equiv.), trifluoroacetic acid (22.3 µL, 0.300 mmol, 1.00 equiv.) and paraformaldehyde (9.01 mg, 0.300 mmol, 1.00 equiv.). Purification by flash column chromatography (dichloromethane/DMA-mix 95:5 to 0:100) gave the title compound (44.6 mg, 0.163 mmol, 55%) as a colourless oil.

^{1}H NMR (400 MHz, $CDCl_3$) δ 7.35 – 7.27 (m, 4H), 7.25 – 7.18 (m, 1H), 5.57 – 5.41 (m, 2H), 3.80 (q, *J* = 6.6 Hz, 1H), 3.09 – 2.90 (m, 2H), 2.05 – 1.92 (m, 2H), 1.74 (br s, 1H), 1.46 – 1.37 (m, 3H) 1.35 – 1.16 (m, 12H), 0.88 (t, *J* = 6.9 Hz, 3H).

^{13}C NMR (101 MHz, $CDCl_3$) δ 145.6, 133.1, 128.6 (2C), 128.1, 127.0, 126.8 (2C), 57.6, 49.7, 32.5, 32.0, 29.6, 29.4 (2C), 29.3, 24.3, 22.8, 14.3.

HRMS (ESI$^+$): exact mass calculated for $[M+H]^+$ ($C_{19}H_{32}N$) requires *m/z* 274.2529, found *m/z* 274.2524.

IR (neat) ν_{max}: 2956, 2853, 1461, 1451, 969, 759, 722, 699 cm^{-1}.

(*E*)-3-Cyclopropyl-N-(1-phenylethyl)prop-2-en-1-amine (1.49)

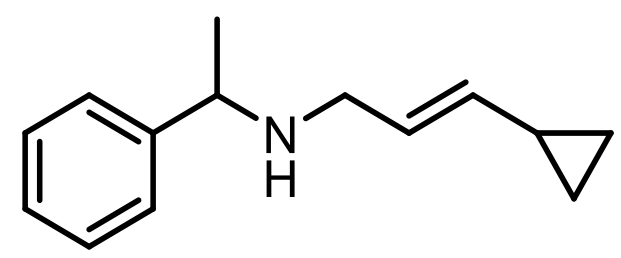

$C_{14}H_{19}N$
MW: 201.3130

Prepared following general procedure **4** using *N*-methyl-1-phenylethan-1-amine (43.6 µL, 0.300 mmol, 1.00 equiv.), ethynylcyclopropane (15.5 µL, 0.330 mmol, 1.10 equiv.), paraformaldehyde (9.0 mg, 0.30 mmol, 1.0 equiv.), trifluoroacetic acid (23.0 µL, 0.300 mmol, 1.00 equiv.) and HFIP (0.25 mL). Purification by flash column chromatography (dichloromethane/DMA-mix 95:5 to 0:100) gave the title compound (30.8 mg, 0.15 mmol, 51%) as a yellow oil.

^{1}H NMR (600 MHz, $CDCl_3$) δ 7.34 – 7.27 (m, 4H), 7.25 – 7.21 (m, 1H), 5.59 (dt, *J* = 15.2, 6.5 Hz, 1H), 5.07 (dd, *J* = 15.3, 8.7 Hz, 1H), 3.79 (q, *J* = 6.6 Hz, 1H), 3.02 (dd, *J* = 6.5, 1.2 Hz, 2H), 1.43 – 1.37 (m, 2H), 1.35 (d, *J* = 6.6 Hz, 3H), 0.70 – 0.64 (m, 2H), 0.35 – 0.30 (m, 2H).

^{13}C NMR (151 MHz, $CDCl_3$) δ 139.4, 138.6, 128.9 (2C), 128.7 (2C), 126.5, 123.7, 51.2, 50.0, 35.6, 13.6, 6.9 (2C).

HRMS (ESI^{+}): exact mass calculated for $[M+H]^+$ ($C_{14}H_{20}N^+$) requires *m/z* 202.1590, found *m/z* 202.1592.

IR (neat) ν_{max}: 3261, 3041, 2958, 2858, 1432, 1350, 1252, 1177, 1053, 1023, 922 cm^{-1}.

(*E*)-3-Cyclopentyl-*N*-(1-phenylethyl)prop-2-en-1-amine (1.50)

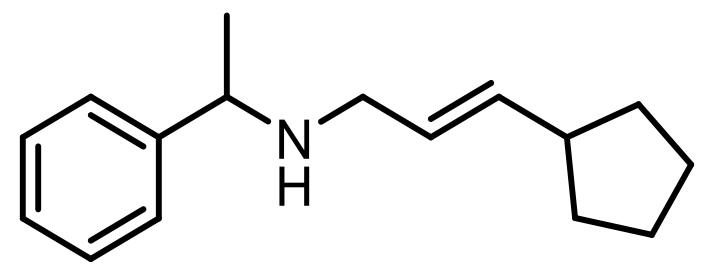

$C_{16}H_{23}N$
MW: 229.3670

Prepared following general procedure **4** using *N*-methyl-1-phenylethanamine (38.7 µL, 0.300 mmol, 1.00 equiv.), cyclopentylacetylene (31.1 mg, 0.330 mmol, 1.10 equiv.), trifluoroacetic acid (22.3 µL, 0.300 mmol, 1.00 equiv.) and paraformaldehyde (9.01 mg, 0.300 mmol, 1.00 equiv.). Purification by flash column chromatography (dichloromethane/DMA-mix 95:5 to 0:100) gave the title compound (34.7 mg, 0.151 mmol, 51%) as a colourless oil.

^{1}H NMR (400 MHz, $CDCl_3$) δ 7.35 – 7.28 (m, 4H), 7.25 – 7.20 (m, 1H), 5.49 (dd, *J* = 5.2, 3.6 Hz, 2H), 3.79 (q, *J* = 6.6 Hz, 1H), 3.07 – 2.97 (m, 2H), 2.46 – 2.29 (m, 1H), 1.82 – 1.69 (m, 2H), 1.68 – 1.47 (m, 5H), 1.35 (t, *J* = 7.0 Hz, 3H), 1.30 – 1.19 (m, 2H).

^{13}C NMR (101 MHz, $CDCl_3$) δ 145.6, 137.5, 128.6 (2C), 127.0, 126.8 (2C), 126.3, 57.7, 49.8 (2C), 43.2, 33.2, 25.2 (2C), 24.3.

HRMS (ESI^{+}): exact mass calculated for $[M+H]^{+}$ ($C_{16}H_{24}N$) requires *m/z* 230.1903, found *m/z* 230.1900.

IR (neat) ν_{max}: 2951, 2866, 1669, 1598, 1491, 1369, 1120, 1057, 1036, 777, 696, 575 cm^{-1}.

N-Phenethyl-5-phenylpentan-1-amine (1.51)

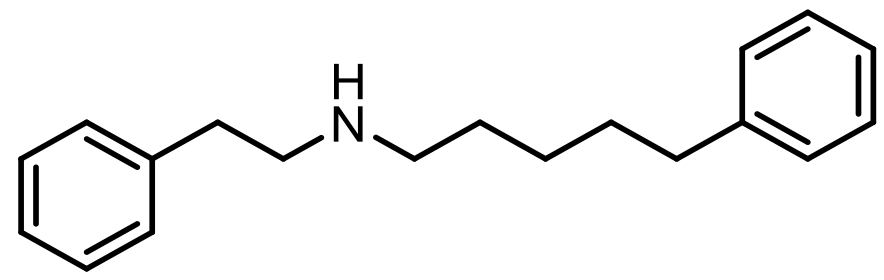

$C_{19}H_{25}N$
MW: 267.4160

Prepared following general procedure **4** using *N*-methyl-2-phenylethan-1-amine (43.6 µL, 0.300 mmol, 1.00 equiv.), 4-phenyl-1-butene (50.0 µL, 0.330 mmol, 1.10 equiv.), trifluoroacetic acid (22.3 µL, 0.300 mmol, 1.00 equiv.) and paraformaldehyde (9.0 mg, 0.30 mmol, 1.0 equiv.). Purification by flash column chromatography (dichloromethane/DMA-mix 95:5 to 0:100) gave the title compound (69.0 mg, 0.258 mmol, 86%) as a yellow oil.

^{1}H NMR (600 MHz, CDCl$_3$) δ 7.30 – 7.25 (m, 4H), 7.23 – 7.14 (m, 6H), 2.91 – 2.86 (m, 4H), 2.85 – 2.81 (m, 4H), 1.85 (s, 1H), 1.62 (dt, *J* = 15.5, 7.7 Hz, 2H), 1.53 (dt, *J* = 15.0, 7.5 Hz, 2H), 1.38 – 1.30 (m, 2H).

^{13}C NMR (151 MHz, CDCl$_3$) 142.6, 139.9, 128.7 (2C), 128.5 (2C), 128.4, 128.2, 126.2 (2C), 125.6 (2C), 51.1, 49.7, 36.1, 35.9, 31.3, 29.7, 27.0.

The spectroscopic properties are consistent with the data available in the literature.[19]

3-((3-Phenylpropyl)amino)propanenitrile (1.52)

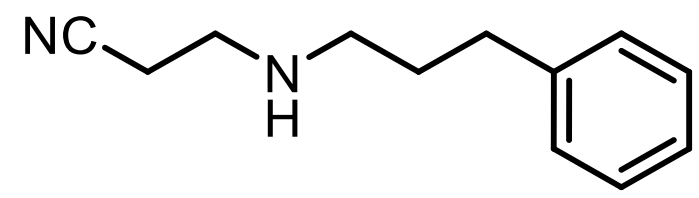

$C_{14}H_{20}N_2$
MW: 188.2740

Prepared following general procedure **4** using 3-(methylamino)propanenitrile (25.2 mg, 0.300 mmol, 1.00 equiv.), styrene (34.5 µL, 0.33 mmol, 1.10 equiv.), trifluoroacetic acid (22.3 µL, 0.30 mmol, 1.00 equiv.) and paraformaldehyde (9.0 mg, 0.30 mmol, 1.0 equiv.). Purification by flash column chromatography (dichloromethane/DMA-mix 95:5 to 0:100) gave the title compound (39.0 mg, 0.207 mmol, 69%) as a yellow oil.

^{1}H NMR (600 MHz, $CDCl_3$) δ 7.32 – 7.26 (m, 2H), 7.23 – 7.16 (m, 3H), 2.92 (t, *J* = 6.6 Hz, 2H), 2.73 – 2.63 (m, 4H), 2.50 (t, *J* = 6.6 Hz, 2H), 1.92 – 1.76 (m, 2H), 1.36 (br s, 1H).
^{13}C NMR (151 MHz, $CDCl_3$) 141.9, 128.5 (2C), 128.5 (2C), 126.0, 118.8, 48.7, 45.2, 33.6, 31.7, 18.9.
HRMS (ESI$^+$): exact mass calculated for $[M+H]^+$ ($C_{12}H_{17}N_2$) requires *m/z* 189.1386, found *m/z* 189.1390.
IR (neat) ν_{max}: 3298, 2984, 2853, 2278, 1563, 1489, 1462, 1321, 1265, 1033, 945 cm^{-1}.

Ethyl (5-phenylpentyl)glycinate (1.53)

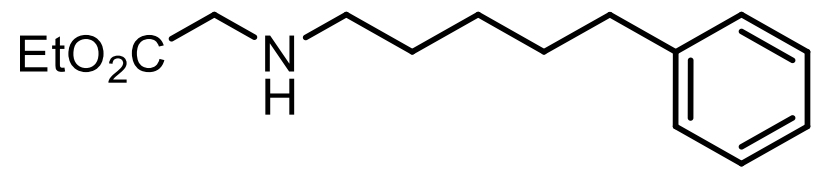

$C_{15}H_{23}NO_2$
MW: 249.3540

Prepared following general procedure **1** using ethyl sarcosinate hydrochloride (46.1 mg, 0.300 mmol, 1.00 equiv.), 4-phenyl-1-butene (50.0 µL, 0.330 mmol, 1.10 equiv.) and paraformaldehyde (9.0 mg, 0.30 mmol, 1.0 equiv.). Purification by flash column chromatography (dichloromethane/DMA-mix 95:5 to 0:100) gave the title compound (53.4 mg, 0.210 mmol, 70%) as a yellow oil.

^{1}H NMR (400 MHz, $CDCl_3$) δ 7.32 – 7.25 (m, 2H), 7.22 – 7.14 (m, 3H), 4.20 (q, *J* = 7.2 Hz, 2H), 3.41 (s, 2H), 2.62 (td, *J* = 7.5, 2.7 Hz, 4H), 2.35 (br s, 1H), 1.72 – 1.49 (m, 4H), 1.44 – 1.34 (m, 2H), 1.28 (t, *J* = 7.1 Hz, 3H).
^{13}C NMR (101 MHz, $CDCl_3$) 172.3, 142.7, 128.5 (2C), 128.3 (2C), 125.7, 60.9, 50.9, 49.6, 35.9, 31.4, 29.8, 26.9, 14.3.
HRMS (ESI$^+$): exact mass calculated for $[M+H]^+$ ($C_{15}H_{24}NO_2$) requires *m/z* 250.1802, found *m/z* 250.1806.
IR (neat) ν_{max}: 3320, 3264, 3032, 2959, 2867, 1744, 1453, 1363, 1257, 1111, 1022, 952 cm^{-1}.

_N,N_-Dimethyl-3-((3-phenylpropyl)amino)propanamide (1.54)

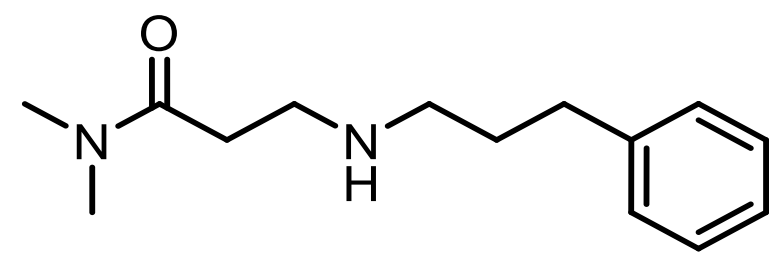

$C_{14}H_{22}N_2O$
MW: 234.3430

Prepared following general procedure **4** using *N*,*N*-dimethyl-3-(methylamino)propanamide (39.1 mg, 0.300 mmol, 1.00 equiv.), styrene (34.5 µL, 0.33 mmol, 1.10 equiv.), trifluoroacetic acid (22.3 µL, 0.30 mmol, 1.00 equiv.) and paraformaldehyde (9.0 mg, 0.30 mmol, 1.0 equiv.). Purification by flash column chromatography (dichloromethane/DMA-mix 95:5 to 0:100) gave the title compound (51.3 mg, 0.219 mmol, 73%) as a yellow oil.

Rotameric effects are visible in the measured NMR spectra.

^{1}H NMR (600 MHz, $CDCl_3$) δ 7.30 – 7.25 (m, 2H), 7.22 – 7.16 (m, 3H), 2.99 (s, 3H), 2.95 (s, 3H), 2.88 (t, *J* = 6.2 Hz, 2H), 2.66 (t, *J* = 7.5 Hz, 4H), 2.52 (t, *J* = 6.2 Hz, 2H), 1.91 – 1.77 (m, 4H).
^{13}C NMR (151 MHz, $CDCl_3$) 172.1, 142.3, 128.5 (2C), 128.4 (2C), 125.8, 49.7, 45.5, 37.2, 35.4, 33.8 33.5, 31.8.
HRMS (ESI$^+$): exact mass calculated for $[M+H]^+$ ($C_{14}H_{23}N_2O$) requires *m/z* 235.1805, found *m/z* 235.1805.
IR (neat) ν_{max}: 3301, 2963, 2864, 1711, 1611, 1567, 1342, 1211, 1065, 983 cm^{-1}.

_N_-Pentyl-5-phenylpentan-1-amine (1.55)

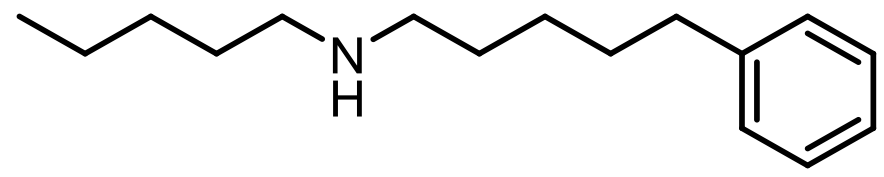

$C_{16}H_{27}N$
MW: 233.3990

Prepared following general procedure **4** using *N*-methylpentan-1-amine (30.4 mg, 0.300 mmol, 1.00 equiv.), 4-phenyl-1-butene (50.0 µL, 0.330 mmol, 1.10 equiv.), trifluoroacetic acid (22.3 µL, 0.300 mmol, 1.00 equiv.) and paraformaldehyde (9.0 mg, 0.30 mmol, 1.0 equiv.). Purification by flash column chromatography (dichloromethane/DMA-mix 95:5 to 0:100) gave the title compound (44.0 mg, 0.190 mmol, 63%) as a yellow oil.

^{1}H NMR (600 MHz, $CDCl_3$) δ 7.31 – 7.28 (m, 2H), 7.22 – 7.18 (m, 3H), 2.66 – 2.58 (m, 6H), 1.66 (dt, *J* = 9.1, 7.7 Hz, 2H), 1.57 – 1.47 (m, 4H), 1.42 – 1.28 (m, 7H), 0.92 (t, *J* = 7.1 Hz, 3H).
^{13}C NMR (151 MHz, $CDCl_3$) δ 142.7, 128.4 (2C), 128.2, 125.6 (2C), 50.1, 50.0, 35.9, 31.4, 30.1, 29.9, 29.6, 27.1, 22.6, 14.0.
HRMS (ESI$^+$): exact mass calculated for $[M+H]^+$ ($C_{16}H_{28}N$) requires *m/z* 234.2216, found *m/z* 234.2219.
IR (neat) ν_{max}: 3300, 3278, 3000, 2944, 2885, 2842, 1503, 1475, 1461, 1285, 1122, 788 cm^{-1}.

***N*-Phenethylpentan-1-amine (1.56)**

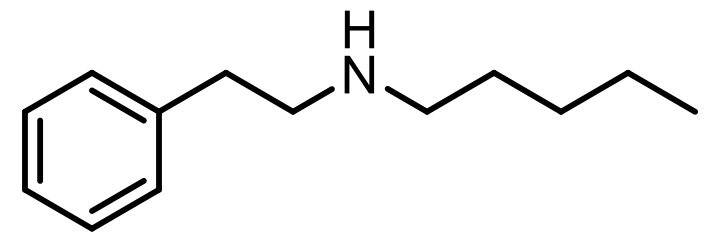

$C_{13}H_{21}N$
MW: 191.3180

Prepared following general procedure **4** using *N*-methyl-phenethylamine (43.6 µL, 0.300 mmol, 1.00 equiv.), 1-butene (16.5 µL, 0.330 mmol, 1.10 equiv., 2.0 M in THF), trifluoroacetic acid (22.3 µL, 0.300 mmol, 1.00 equiv.) and paraformaldehyde (9.0 mg, 0.30 mmol, 1.0 equiv.). Purification by flash column chromatography (dichloromethane/DMA-mix 95:5 to 0:100) gave the title compound (41.0 mg, 0.214 mmol, 71%) as a pale-yellow oil.

^{1}H NMR (400 MHz, $CDCl_3$) δ 7.32 – 7.27 (m, 2H), 7.23 – 7.17 (m, 3H), 2.94 – 2.87 (m, 2H), 2.88 – 2.80 (m, 2H), 2.68 – 2.60 (m, 2H), 1.56 – 1.43 (m, 2H), 1.36 – 1.18 (m, 5H), 0.88 (t, *J* = 6.9 Hz, 3H).
^{13}C NMR (101 MHz, $CDCl_3$) δ 140.0, 128.8 (2C), 128.6 (2C), 126.3, 51.2, 49.9, 36.2, 29.6, 29.6, 22.7, 14.1.
HRMS (ESI$^+$): exact mass calculated for $[M+H]^+$ ($C_{13}H_{22}N$) requires *m/z* 192.1747, found *m/z* 192.1745.
IR (neat) ν_{max}: 3027, 2954, 2854, 1634, 1495, 1377, 1125, 1030, 746, 696 cm^{-1}.

3-(Naphthalen-2-yl)-*N*-phenethylpropan-1-amine (1.57)

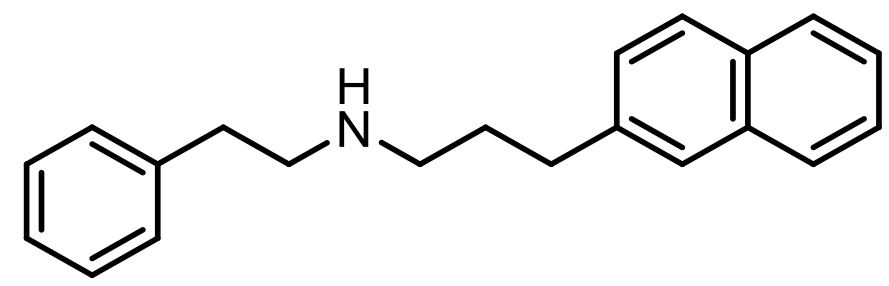

$C_{21}H_{23}N$
MW: 289.4220

Prepared following general procedure **4** using *N*-methyl-2-phenylethan-1-amine (43.6 µL, 0.300 mmol, 1.00 equiv.), 2-vinylnaphthalene (41.7 µL, 0.300 mmol, 1.00 equiv.), trifluoroacetic acid (22.3 µL, 0.300 mmol, 1.00 equiv.) and paraformaldehyde (9.0 mg, 0.30 mmol, 1.0 equiv.). Purification by flash column chromatography /DMA-mix 95:5 to 0:100) gave the title compound (36.6 mg, 0.130 mmol, 42%) as a yellow oil.

^{1}H NMR (600 MHz, $CDCl_3$) δ 7.79 (d, *J* = 7.9 Hz, 1H), 7.75 (dd, *J* = 8.1, 3.3 Hz, 2H), 7.59 – 7.56 (m, 1H, 1H), 7.43 (ddd, *J* = 11.9, 7.7, 1.2 Hz, 2H), 7.31 – 7.27 (m, 3H), 7.22 – 7.16 (m, *J* = 13.9, 7.2 Hz, 3H), 2.91 (t, *J* = 7.1 Hz, 2H), 2.84 (t, *J* = 7.1 Hz, 2H), 2.79 (t, *J* = 7.6 Hz, 2H), 2.72 (t, *J* = 7.2 Hz, 2H), 1.96 – 1.90 (m, 2H), 1.63 – 1.61 (br s, 1H).

^{13}C NMR (151 MHz, $CDCl_3$) δ 139.6, 139.2, 133.5 (2C), 131.9, 128.6, 128.5 (2C), 127.9, 127.5, 127.3, 127.1, 126.2, 126.2, 125.9, 125.1, 50.9, 49.0, 35.9, 33.6, 31.0.

HRMS (ESI$^+$): exact mass calculated for $[M+H]^+$ ($C_{21}H_{24}N$) requires *m/z* 290.1903, found *m/z* 290.1905.

IR (neat) ν_{max}: 3321, 3256, 3033, 2986, 2910, 1511, 1455, 1422, 1252, 1177, 1083, 820 cm^{-1}.

***N*-Phenethylheptan-1-amine (1.58)**

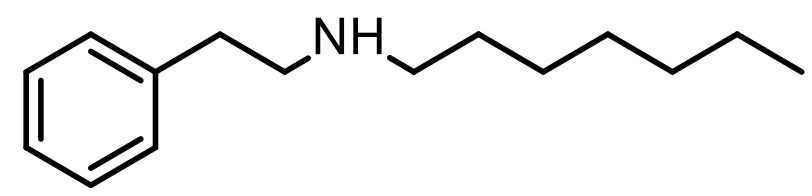

$C_{15}H_{25}N$
MW: 219.3720

Prepared following general procedure **4** using *N*-methyl-2-phenylethan-1-amine (43.6 µL, 0.300 mmol, 1.00 equiv.), 1-hexene (41.0 µL, 0.330 mmol, 1.10 equiv.), trifluoroacetic acid (22.3 µL, 0.300 mmol, 1.00 equiv.) and paraformaldehyde (9.0 mg, 0.30 mmol, 1.0 equiv.). Purification by flash column chromatography (dichloromethane/DMA-mix 95:5 to 0:100) gave the title compound (48.2 mg, 0.220 mmol, 73%) as a yellow oil.

^{1}H NMR (600 MHz, $CDCl_3$) δ 7.31 – 7.27 (m, 2H), 7.22 – 7.19 (m, 3H), 2.92 – 2.86 (m, 2H), 2.86 – 2.80 (m, 2H), 2.63 (t, *J* = 7.7 Hz, 2H), 1.51 – 1.46 (m, 2H), 1.33 – 1.21 (m, 9H), 0.87 (t, *J* = 7.0 Hz, 3H).

^{13}C NMR (151 MHz, $CDCl_3$) δ 140.1, 128.9 (2C), 128.6, 126.3 (2C), 51.3, 50.0, 36.3, 32.0, 31.8, 30.0, 29.3, 26.9, 14.2.

The spectroscopic properties are consistent with the data available in the literature.[20]

N-(Cyclohexylmethyl)-2-phenylethan-1-amine (1.59)

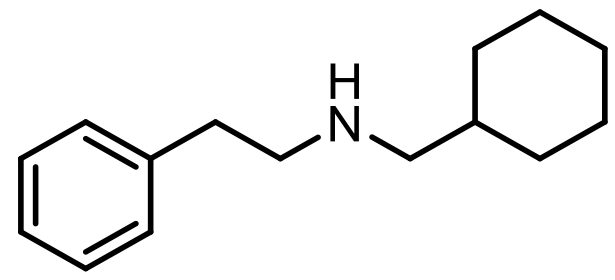

$C_{15}H_{23}N$
MW: 217.3560

Prepared following general procedure **4** using *N*-methyl-2-phenylethan-1-amine (43.6 µL, 0.300 mmol, 1.00 equiv.), cyclohexene (33.8 µL, 0.330 mmol, 1.10 equiv.), trifluoroacetic acid (22.3 µL, 0.300 mmol, 1.00 equiv.) and paraformaldehyde (9.0 mg, 0.30 mmol, 1.0 equiv.). Purification by flash column chromatography (dichloromethane/DMA-mix 95:5 to 0:100) gave the title compound (26.6 mg, 0.120 mmol, 41%) as a yellow oil.

^{1}H NMR (600 MHz, $CDCl_3$) δ 7.30 – 7.27 (m, *J* = 9.8, 5.3 Hz, 2H), 7.23 – 7.19 (m, 3H), 2.94 – 2.90 (m, 2H), 2.89 – 2.86 (m, 2H), 2.52 (d, *J* = 6.7 Hz, 2H), 1.75 – 1.67 (m, 4H), 1.66 – 1.62 (m, 1H), 1.54 – 1.46 (m, 1H), 1.26 – 1.18 (m, 2H), 1.14 (ddt, *J* = 25.1, 12.7, 3.1 Hz, 1H), 0.93 – 0.86 (m, 2H). *The NH proton was not observed.*

^{13}C NMR (151 MHz, $CDCl_3$) δ 139.9, 129.0 (2C), 128.8 (2C), 126.6, 56.6, 51.5, 37.8, 36.1, 31.7 (2C), 26.9 (2C), 26.3.

The spectroscopic properties are consistent with the data available in the literature.[21]

_N_-(5-Phenylpentyl)cyclohexanamine (1.60)

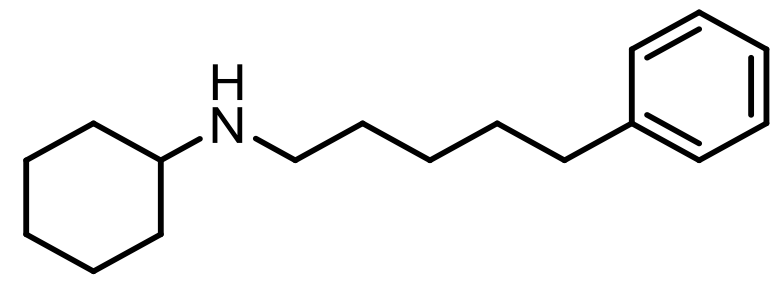

$C_{17}H_{27}N$
MW: 245.4100

Prepared following general procedure **4** using *N*-methylcyclohexanamine (34.0 mg, 0.300 mmol, 1.00 equiv.), 4-phenyl-1-butene (50.0 µL, 0.33 mmol, 1.10 equiv.), trifluoroacetic acid (22.3 µL, 0.300 mmol, 1.00 equiv.) and paraformaldehyde (9.0 mg, 0.30 mmol, 1.0 equiv.). Purification by flash column chromatography (dichloromethane/DMA-mix 95:5 to 0:100) gave the title compound (39.1 mg, 0.160 mmol, 53%) as a yellow oil.

^{1}H NMR (600 MHz, $CDCl_3$) δ 7.30 – 7.25 (m, 2H), 7.19 – 7.15 (m, 3H), 2.64 – 2.58 (m, 4H), 2.39 (tt, *J* = 10.5, 3.7 Hz, 1H), 1.87 (dd, *J* = 12.6, 2.5 Hz, 2H), 1.74 – 1.69 (m, 2H), 1.66 – 1.58 (m, 3H), 1.51 (dt, *J* = 15.0, 7.5 Hz, 2H), 1.36 (dt, *J* = 15.2, 7.6 Hz, 2H), 1.28 – 1.21 (m, 2H), 1.18 – 1.11 (m, 2H), 1.05 (app qd, *J* = 12.7, 3.3 Hz, 2H).

^{13}C NMR (151 MHz, $CDCl_3$) δ 142.9, 128.5 (2C), 128.4, 125.8 (2C), 57.1, 47.1, 36.0, 33.9, 31.6 (2C), 30.6, 27.3 (2C), 26.4, 25.3.

HRMS (ESI$^+$): exact mass calculated for $[M+H]^+$ ($C_{17}H_{28}N$) requires *m/z* 246.2216, found *m/z* 246.2216.

IR (neat) ν_{max}: 3333, 3320, 3011, 2954, 2880, 2855, 1520, 1477, 1456, 1366, 1288, 1111, 1089, 798 cm^{-1}.

N-Phenethyldodecan-1-amine (1.61)

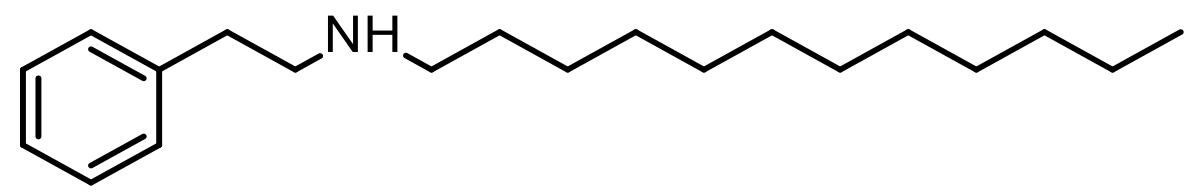

$C_{20}H_{35}N$
MW: 289.5070

Prepared following general procedure **4** using *N*-methyl-2-phenylethan-1-amine (43.6 µL, 0.30 mmol, 1.00 equiv.), 1-undecene (67.9 µL, 0.33 mmol, 1.10 equiv.), trifluoroacetic acid (22.3 µL, 0.30 mmol, 1.00 equiv.) and paraformaldehyde (9.0 mg, 0.30 mmol, 1.0 equiv.). Purification by flash column chromatography (dichloromethane/DMA-mix 95:5 to 0:100) gave the title compound (56.4 mg, 0.20 mmol, 65%) as a yellow oil.

^{1}H NMR (600 MHz, CDCl$_3$) δ 7.31 – 7.27 (m, 2H), 7.22 – 7.19 (m, *J* = 7.2 Hz, 3H), 2.91 – 2.87 (m, 2H), 2.86– 2.83 (m, 2H), 2.63 (t, *J* = 7.4 Hz, 2H), 1.52 – 1.46 (m, 2H), 1.31 – 1.24 (m, 19H), 0.88 (t, *J* = 7.0 Hz, 3H).

^{13}C NMR (151 MHz, CDCl$_3$) δ 140.1, 128.9 (2C), 128.6, 126.3 (2C), 51.3, 50.0, 36.3, 32.1, 30.0, 29.81, 29.79, 29.76, 29.73, 29.68, 29.5, 27.5, 22.8, 14.3.

The spectroscopic properties are consistent with the data available in the literature.[23]

***N*-Phenethyl-3-(thiophen-3-yl)propan-1-amine (1.62)**

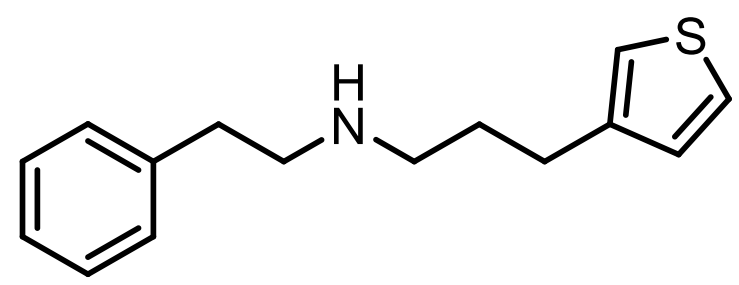

$C_{15}H_{19}NS$
MW: 245.3840

Prepared following general procedure **4** using *N*-methyl-2-phenylethan-1-amine (43.6 µL, 0.30 mmol, 1.00 equiv.), 3-vinylthiophene (36.4 mg, 0.33 mmol, 1.10 equiv.), trifluoroacetic acid (22.3 µL, 0.30 mmol, 1.00 equiv.) and paraformaldehyde (9.0 mg, 0.30 mmol, 1.0 equiv.). Purification by flash column chromatography (dichloromethane/DMA-mix 95:5 to 0:100) gave the title compound (30.8 mg, 0.13 mmol, 42%) as a yellow oil.

^{1}H NMR (600 MHz, CDCl$_3$) δ 7.29 (app t, *J* = 7.5 Hz, 2H), 7.23 – 7.19 (m, 4H), 6.92 – 6.90 (m, 2H), 2.96 (t, *J* = 7.4 Hz, 2H), 2.89 (t, *J* = 7.3 Hz, 2H), 2.74 (t, *J* = 7.6 Hz, 2H), 2.66 (t, *J* = 7.6 Hz, 2H), 1.92 – 1.88 (m, 2H). *The NH proton was not observed.*

^{13}C NMR (151 MHz, CDCl$_3$) δ 141.9, 139.4, 128.9, 128.8 (2C), 128.2, 126.6, 125.7 (2C), 120.4, 50.8, 49.0, 35.5, 29.8, 27.9.

HRMS (ESI$^+$): exact mass calculated for $[M+H]^+$ ($C_{15}H_{20}NS$) requires *m/z* 246.1311, found *m/z* 246.1307.

IR (neat) ν_{max}: 3290, 3211, 3005, 2986, 2867, 1452, 1375, 1341, 1162, 1052, 832, 712 cm^{-1}.

N-Phenethyl-3-(thiophen-3-yl)propan-1-amine (1.63)

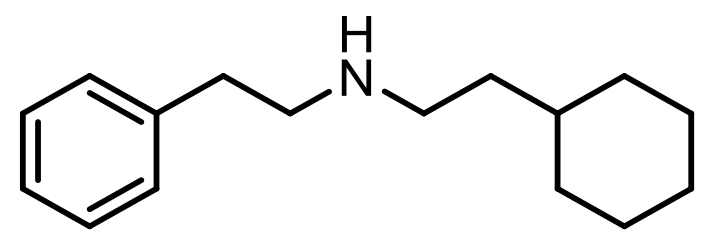

$C_{16}H_{25}N$
MW: 231.3830

Prepared following general procedure **4** using *N*-methyl-2-phenylethan-1-amine (43.6 µL, 0.300 mmol, 1.00 equiv.), methylenecyclohexane (36.1 µL, 0.300 mmol, 1.0 equiv.), trifluoroacetic acid (22.3 µL, 0.300 mmol, 1.00 equiv.) and paraformaldehyde (9.0 mg, 0.30 mmol, 1.00 equiv.). Purification by flash column chromatography (dichloromethane/DMA-mix 95:5 to 0:100) gave the title compound (31.4 mg, 0.140 mmol, 45%) as a yellow oil.

^{1}H NMR (600 MHz, $CDCl_3$) δ 7.32 – 7.27 (m, 2H), 7.22 – 7.17 (m, 3H), 2.92 – 2.86 (m, 2H), 2.82 (dd, *J* = 7.6, 6.0 Hz, 2H), 2.67 – 2.61 (m, 2H), 1.70 – 1.61 (m, 5H), 1.56 (br s, 1H), 1.42 – 1.33 (m, 2H), 1.31 – 1.08 (m, 4H), 0.95 – 0.86 (m, 2H).

^{13}C NMR (151 MHz, $CDCl_3$) δ 140.2, 128.8 (2C), 128.6, 126.3 (2C), 51.4, 47.6, 37.7, 36.4, 35.9, 33.5 (2C), 26.7 (2C), 26.4.

HRMS (ESI$^+$): exact mass calculated for $[M+H]^+$ ($C_{16}H_{26}N$) requires *m/z* 232.2060, found *m/z* 232.2065.

IR (neat) ν_{max}: 3288, 3223, 3033, 2981, 2852, 1432, 1351, 1311, 1172, 955, 844 cm^{-1}.

3-(4-Methylcyclohex-3-en-1-yl)-*N*-phenethylbutan-1-amine(1.64)

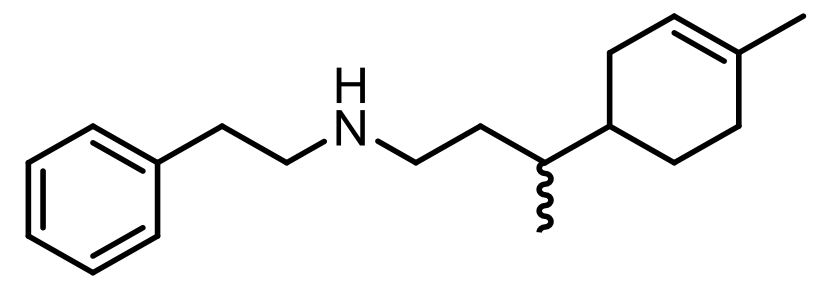

$C_{19}H_{29}N$
MW: 271.4480

Prepared following general procedure **4** using *N*-methyl-1-phenylethan-1-amine (43.6 µL, 0.30 mmol, 1.00 equiv.), (*S*)-limonene (54 µL, 0.33 mmol, 1.10 equiv.), paraformaldehyde (9.0 mg, 0.30 mmol, 1.00 equiv.), trifluoroacetic acid (23.0 µL, 0.30 mmol, 1.00 equiv.) and HFIP (0.25 mL). Purification by flash column chromatography (dichloromethane/DMA-mix 95:5 to 0:100) gave the title compound as a mixture of two diastereomers (39.0 mg, 0.14 mmol, *d.r.* = 1.1:1, 48%) as a yellow oil.

The products consist of two diastereomers in a ratio of 1.1:1. Signals observed in the NMR spectra for both diastereomers are listed below.

^{1}H NMR (700 MHz, $CDCl_3$) δ 7.32 – 7.28 (m, 2H), 7.24 – 7.17 (m, 3H), 5.39 – 5.34 (m, 1H), 2.91 – 2.80 (m, 4H), 2.67 (ddd, *J* = 11.1, 10.1, 5.2 Hz, 1H), 2.59 (ddd, *J* = 11.3, 9.7, 6.0 Hz, 1H), 2.01 – 1.87 (m, 3H), 1.78 – 1.66 (m, 2H), 1.63 (s, 3H), 1.60 – 1.54 (m, 1H), 1.50 – 1.33 (m, 4H), 1.32 – 1.19 (m, 3H), 0.86 – 0.82 (m, 3H).

^{13}C NMR (176 MHz, $CDCl_3$) δ 140.3, 134.2, 128.9, 128.6, 126.3, 121.2, 121.1, 51.47, 51.46, 48.40, 48.35, 38.8, 38.6, 36.6, 35.5, 35.4, 34.6, 31.1, 31.0, 29.5, 27.7, 27.2, 25.6, 23.6, 16.6, 16.2.

HRMS (ESI$^+$): exact mass calculated for $[M+H]^+$ ($C_{19}H_{30}N^+$) requires *m/z* 272.2373, found *m/z* 272.2373.

IR (neat) ν_{max}: 3350, 3301, 3022, 2931, 2883, 2825, 1488, 1455, 1370, 1299, 1250, 1193, 1023, 790 cm^{-1}.

2-Butyl-*N*-phenethylheptan-1-amine (1.65)

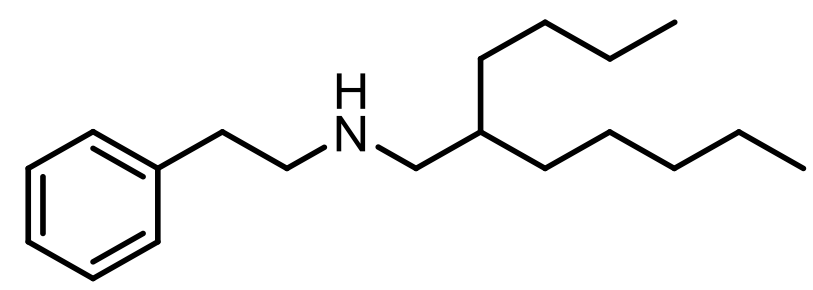

$C_{19}H_{33}N$
MW: 275.4800

Prepared following general procedure **4** using *N*-methyl-2-phenylethan-1-amine (43.6 µL, 0.30 mmol, 1.00equiv.), **(*Z*)-5-decene** (73.4 µL, 0.390 mmol, 1.30 equiv.), trifluoroacetic acid (22.3 µL, 0.300 mmol, 1.00 equiv.) and paraformaldehyde (9.0 mg, 0.30 mmol, 1.0 equiv.). Purification by flash column chromatography (dichloromethane/DMA-mix 95:5 to 0:100) gave the title compound (30.5 mg, 0.11 mmol, 37%) as a yellow oil.

Prepared following general procedure **4** using *N*-methyl-2-phenylethan-1-amine (43.6 µL, 0.300 mmol, 1.00 equiv.), **(*E*)-5-decene** (74.0 µL, 0.390 mmol, 1.30 equiv.), trifluoroacetic acid (22.3 µL, 0.300 mmol, 1.00 equiv.) and paraformaldehyde (9.0 mg, 0.30 mmol, 1.00 equiv.). Purification by flash column chromatography (dichloromethane/DMA-mix 95:5 to 0:100) gave the title compound (32.1 mg, 0.12 mmol, 39%) as a yellow oil.

^{1}H NMR (600 MHz, $CDCl_3$) δ 7.32 – 7.27 (m, 2H), 7.23 – 7.18 (m, 3H), 2.89 (ddd, *J* = 7.1, 5.6, 1.8 Hz, 2H), 2.85 (dd, *J* = 8.0, 5.2 Hz, 2H), 2.54 (d, *J* = 6.3 Hz, 2H), 1.49 (app t, *J* = 5.8 Hz, 1H), 1.30 – 1.20 (m, 15H), 0.89 – 0.86 (m, 6H).

^{13}C NMR (151 MHz, $CDCl_3$) δ 140.0, 128.7 (2C), 128.5, 126.2 (2C), 53.4, 51.3, 37.7, 36.0, 32.3, 32.1, 31.8, 28.9, 26.3, 23.1, 22.7, 14.10, 14.09.

HRMS (ESI$^+$): exact mass calculated for $[M+H]^+$ ($C_{19}H_{34}N$) requires *m/z* 276.2686, found *m/z* 276.2686.

IR (neat) ν_{max}: 3273, 3227, 3015, 2969, 2855, 1445, 1371, 1353, 1222, 1145, 1037, 932, 850, 777 cm^{-1}.

N-(Cyclooct-4-en-1-ylmethyl)-2-phenylethan-1-amine (1.66)

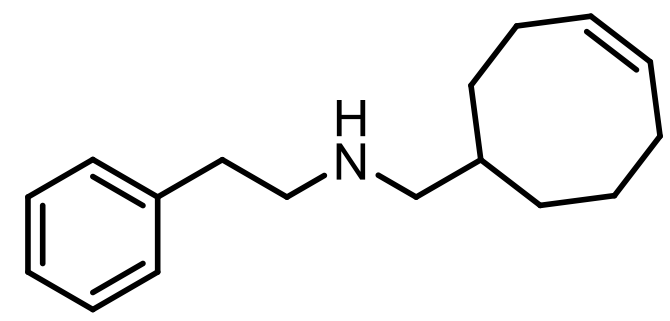

$C_{17}H_{25}N$
MW: 243.3940

Prepared following general procedure **3** using *N*-methyl-2-phenylethan-1-amine (43.6 µL, 0.30 mmol, 1.00equiv.), 1,5-cyclooctadiene (40.5 µL, 0.330 mmol, 1.10 equiv.), trifluoroacetic acid (22.3 µL, 0.300 mmol, 1.00 equiv.) and paraformaldehyde (9.0 mg, 0.30 mmol, 1.0 equiv.). Purification by flash column chromatography (dichloromethane/DMA-mix 95:5 to 0:100) gave the title compound (32.9 mg, 0.135 mmol, 45%) as a yellow oil.

^{1}H NMR (600 MHz, $CDCl_3$) δ 7.32 – 7.27 (m, 2H), 7.25 – 7.18 (m, 3H), 5.63 (tt, *J* = 6.1, 1.5 Hz, 2H), 2.91 – 2.77 (m, 4H), 2.47 (dd, *J* = 11.5, 6.5 Hz, 1H), 2.42 (dd, *J* = 11.5, 7.2 Hz, 1H), 2.31 (ddd, *J* = 10.5, 4.9, 2.3 Hz, 1H), 2.19 – 2.01 (m, 3H), 1.72 – 1.41 (m, 5H), 1.39 – 1.24 (m, 2H), 1.13 – 1.07 (m, 1H).

^{13}C NMR (151 MHz, $CDCl_3$) δ 140.3, 130.4, 130.2, 128.8, 128.6 (2C), 126.2 (2C), 57.7, 51.4, 37.8, 36.5, 33.6, 31.6, 28.3, 26.1, 25.0.

HRMS (ESI$^+$): exact mass calculated for $[M+H]^+$ ($C_{17}H_{26}N$) requires *m/z* 244.2060, found *m/z* 244.2060.

IR (neat) ν_{max}: 2977, 2867, 2853, 1621, 1611, 1543, 1522, 1500, 1322, 1309, 1277, 1260, 1177, 1068, 969, 825 cm^{-1}.

Cinacalcet (2.01)

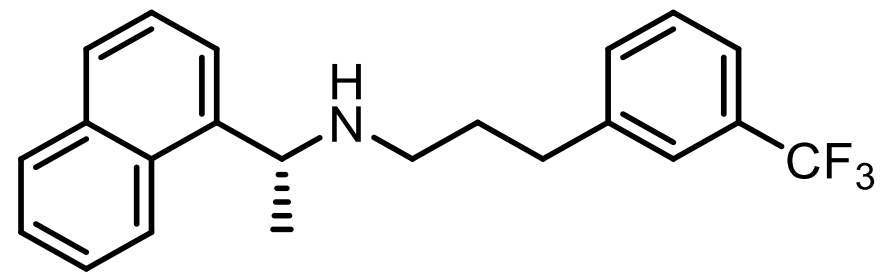

$C_{22}H_{22}F_3N$
MW: 357.4202

Prepared following general procedure **2** using ethyl (*R*)-*N*-methyl-1-(naphthalen-1-yl)ethan-1-amine (2.40 g, 12.80 mmol, 1.00 equiv.), 1-(trifluoromethyl)-3-vinylbenzene (24.7 mL, 16.6 mmol, 1.30 equiv.), trifluoroacetic acid (980 µL, 12.8 mmol, 1.00 equiv.) and paraformaldehyde (384 mg, 12.8 mmol, 1.00 equiv.). The reaction time was 48 h. Purification by flash column chromatography (dichloromethane/DMA-mix 95:5 to 0:100) gave the title compound (2.11 g, 5.90 mmol, 46%, (70% based on recovered starting material)) as a yellow oil.

^{1}H NMR (600 MHz, $CDCl_3$) δ 8.19 (d, *J* = 8.6 Hz, 1H), 7.88 (dd, *J* = 8.0, 1.6 Hz, 1H), 7.75 (d, *J* = 8.1 Hz, 1H), 7.66 – 7.63 (m, 1H), 7.53 – 7.48 (m, 1H), 7.49 – 7.46 (m, 2H), 7.43 – 7.40 (m, 2H), 7.35 (t, *J* = 7.6 Hz, 1H), 7.33 – 7.29 (m, 1H), 4.62 (q, *J* = 6.7 Hz, 1H), 2.78 – 2.69 (m, 1H), 2.69 – 2.63 (m, 2H), 2.63 – 2.55 (m, 1H), 1.84 (app p, *J* = 7.4 Hz, 2H), 1.53 (br s, 1H), 1.50 (d, *J* = 6.6 Hz, 3H).
^{13}C NMR (151 MHz, $CDCl_3$) δ 143.3, 134.1, 131.9, 131.5, 130.73 (q, *J* = 32.1 Hz), 129.1, 128.8, 127.4, 125.92, 125.85, 125.5, 125.20 (q, *J* = 3.8 Hz), 124.8 (q, *J* = 251.8 Hz) 123.1, 122.83, 122.79 (q, *J* = 4.4 Hz), 53.9, 47.4, 33.6, 32.1, 23.8.
^{19}F (376 MHz, $CDCl_3$): δ –62.55.
Chiral HPLC: enantiomeric ratio (er) 99:1, see below for detailed conditions.
The spectroscopic properties are consistent with the data available in the literature.[18]

Method Description:
Column:Lux-3 Cellulose-3
250x4,6mm Particle Size 3 micrometer
Solvent System:n-Heptan/IPA/DEA 98:2:0,1
Flow=0,7 ml/min T=25°C

Chromatogram

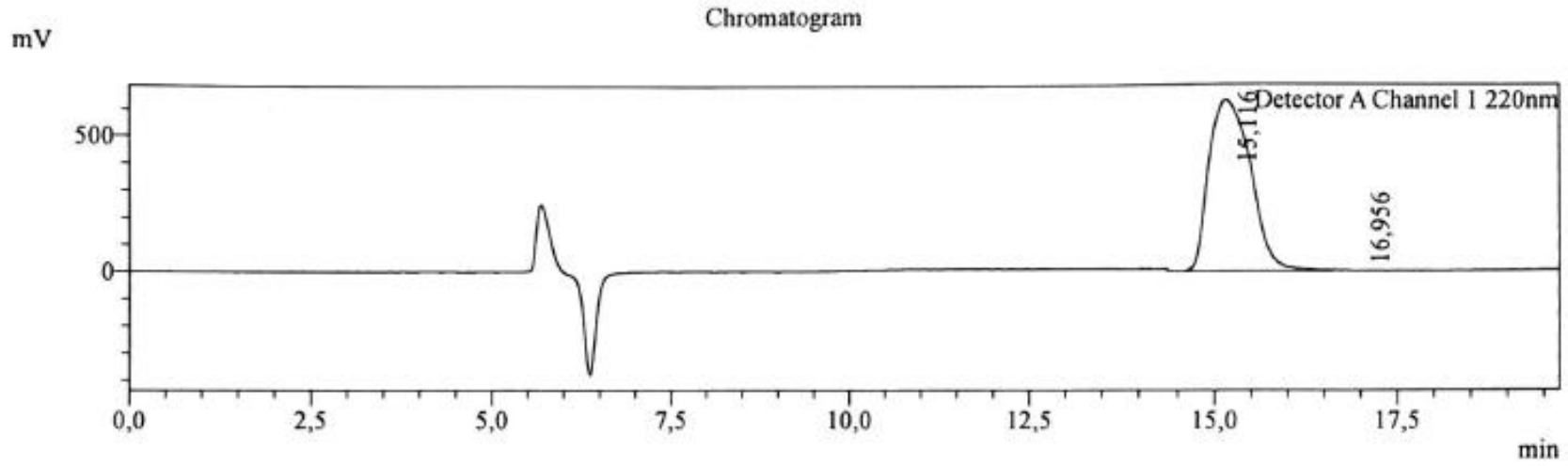

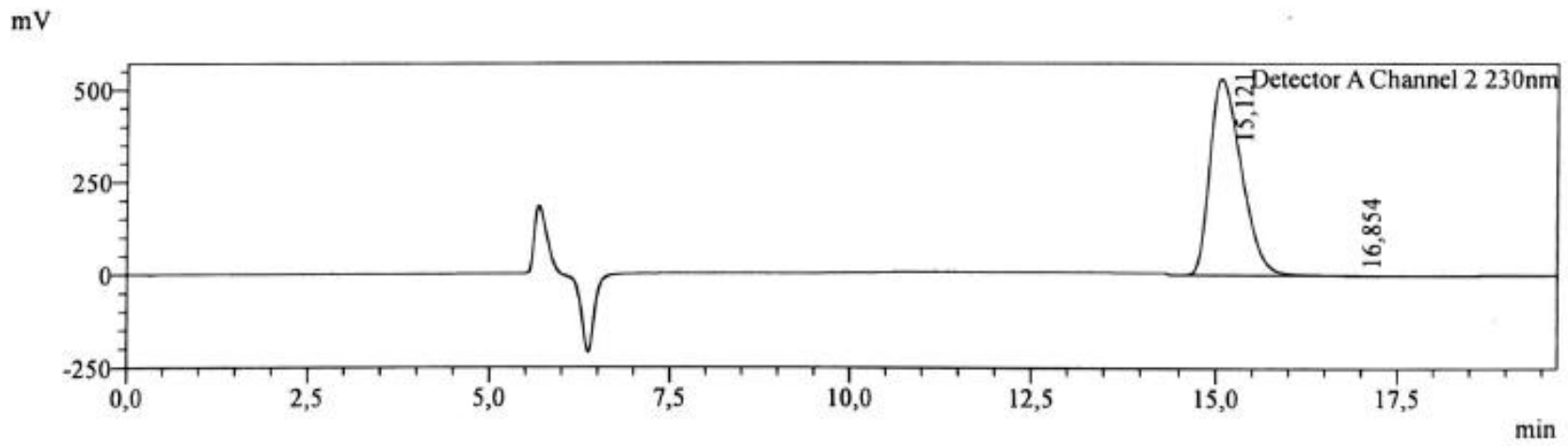

Peak Table

Detector A Channel 1 220nm

Peak#	Ret. Time	Area	Area%
1	15,116	24649621	99,975
2	16,956	6250	0,025
Total		24655872	100,000

Detector A Channel 2 230nm

Peak#	Ret. Time	Area	Area%
1	15,121	15879039	99,946
2	16,854	8551	0,054
Total		15887589	100,000

Method Description:
Column:Lux-3 Cellulose-3
250x4,6mm Particle Size 3 micrometer
Solvent System:n-Heptan/IPA/DEA 98:2:0,1
Flow=0,7 ml/min T=25°C

Chromatogram

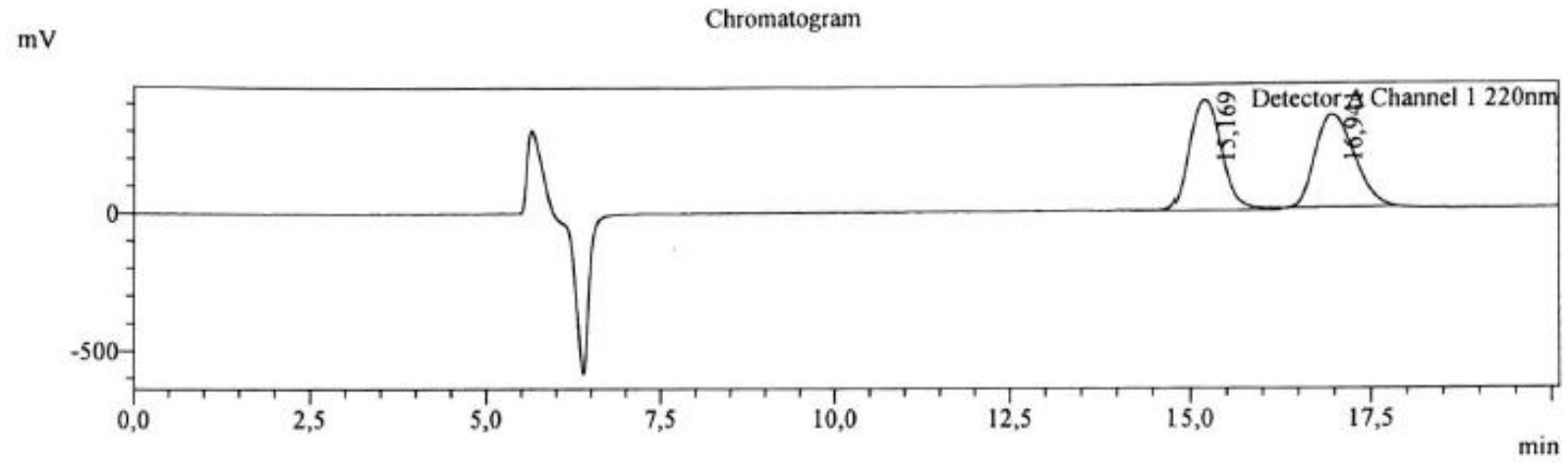

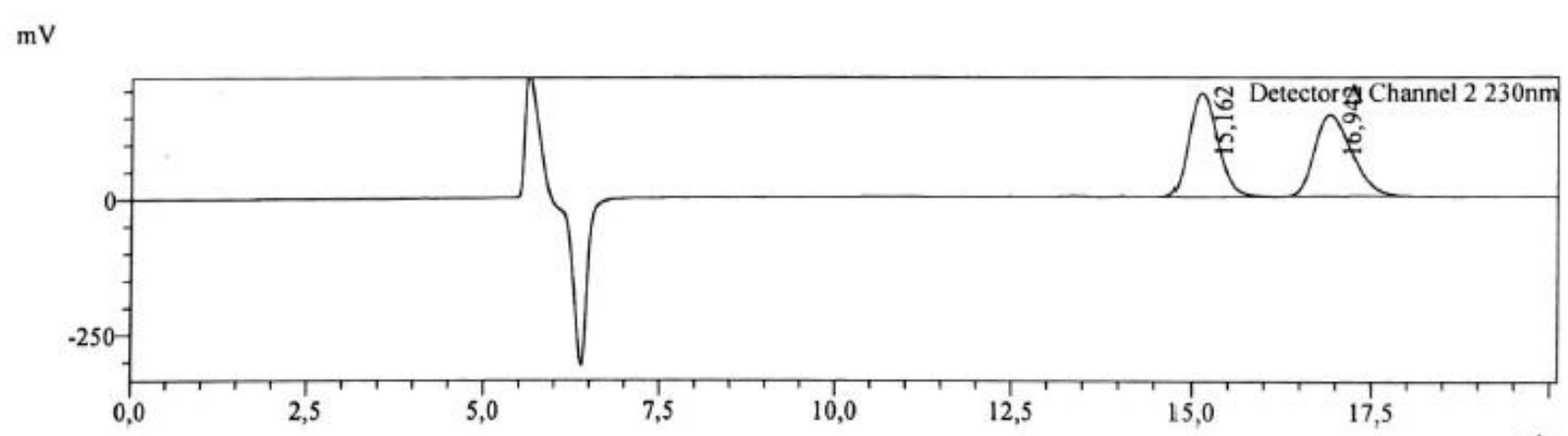

Peak Table

Detector A Channel 1 220nm

Peak#	Ret. Time	Area	Area%
1	15,169	12559041	49,488
2	16,941	12818982	50,512
Total		25378023	100,000

Detector A Channel 2 230nm

Peak#	Ret. Time	Area	Area%
1	15,162	5316377	49,852
2	16,942	5347928	50,148
Total		10664305	100,000

Tecalcet (2.02)

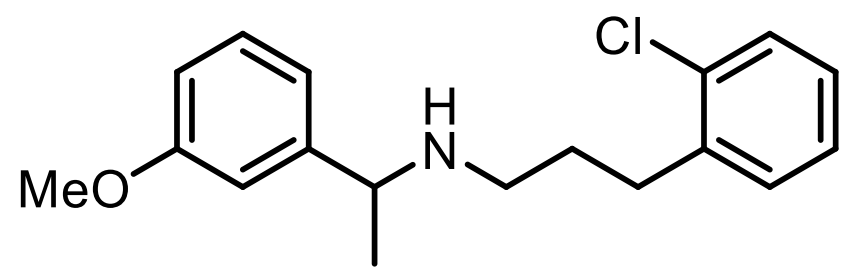

$C_{18}H_{22}ClNO$
MW: 303.830

Prepared following general procedure **3** using 1-(3-methoxyphenyl)-*N*-methylethan-1-amine (49.6 mg, 0.300 mmol, 1.00 equiv.), 2-chlorostyrene (76.0 µL, 0.600 mmol, 2.0 equiv.), trifluoroacetic acid (22.3 µL, 0.300 mmol, 1.00 equiv.) and paraformaldehyde (9.0 mg, 0.300 mmol, 1.00equiv.). Purification by flash column chromatography (dichloromethane/DMA-mix 95:5 to 0:100) gave the title compound (57.3 mg, 0.190 mmol, 63%) as a yellow oil.

^{1}H NMR (600 MHz, CDCl$_3$) δ 7.32 (dd, *J* = 7.7, 1.4 Hz, 1H), 7.25 – 7.22 (m, 1H), 7.18 – 7.14 (m, 2H), 7.15 – 7.08 (m, 1H), 6.91 – 6.89 (m, 2H), 6.80 – 6.77 (m, 1H), 3.82 (s, 3H), 3.75 (q, *J* = 6.6 Hz, 1H), 2.77 (ddd, *J* = 13.7, 9.3, 6.3 Hz, 1H), 2.73 – 2.67 (m, 1H), 2.58 (ddd, *J* = 11.5, 7.8, 6.0 Hz, 1H), 2.54 – 2.47 (m, 1H), 1.82 – 1.73 (m, 2H), 1.48 (s, 1H), 1.35 (d, *J* = 6.6 Hz, 3H).
^{13}C NMR (151 MHz, CDCl$_3$) δ 160.0, 147.8, 140.0, 134.1, 130.5, 129.6, 129.5, 127.4, 126.9, 119.2, 112.4, 112.3, 58.5, 55.4, 47.5, 31.5, 30.3, 24.5.
The spectroscopic properties are consistent with the data available in the literature.[18]

Naftifine (2.03)

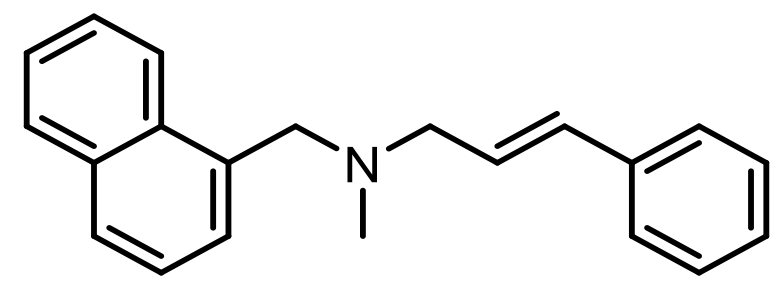

$C_{21}H_{21}N$
MW: 287.4060

Prepared following the slightly modified general procedure **4** using *N*-methyl-1-(naphthalen-1-yl)methanamine (77.0 mg, 0.450 mmol, 1.5 equiv.), ethynylbenzene (33 µL, 0.30 mmol, 1.0 equiv.), trifluoroacetic acid (33.5 µL, 0.450 mmol, 1.50 equiv.) and paraformaldehyde (13.5 mg, 0.450 mmol, 1.50 equiv.). The reaction mixture was then cooled to 0 °C and $NaBH_4$ (34.1 mg, 0.900 mmol, 3.00 equiv.) was added, followed by stirring for 15 min at room temperature. Purification by flash column chromatography (dichloromethane/DMA-mix 95:5 to 0:100) gave the title compound (36.2 mg, 0.130 mmol, 42%) as a yellow oil.

^{1}H NMR (600 MHz, $CDCl_3$) δ 8.37 (d, *J* = 8.5 Hz, 1H), 7.90 (dd, *J* = 8.1, 0.5 Hz, 1H), 7.83 (d, *J* = 8.1 Hz, 1H), 7.58 (ddd, *J* = 8.3, 6.8, 1.4 Hz, 1H), 7.53 (ddd, *J* = 11.4, 8.5, 6.1 Hz, 2H), 7.48 – 7.42 (m, 3H), 7.37 (dd, *J* = 10.6, 4.8 Hz, 2H), 7.27 (ddd, *J* = 12.0, 3.5, 2.4 Hz, 1H), 6.63 (d, *J* = 15.9 Hz, 1H), 6.43 (dt, *J* = 15.9, 6.7 Hz, 1H), 4.00 (s, 2H), 3.33 (dd, *J* = 6.7, 1.2 Hz, 2H), 2.34 (s, 3H).
^{13}C NMR (151 MHz, $CDCl_3$) δ 137.3, 135.0, 134.0, 132.8, 132.6, 128.7, 128.6 (2C), 128.1 (2C), 127.8, 127.6, 127.5, 126.4, 126.0, 125.7, 125.2, 124.8, 60.5, 60.2, 42.6.
The spectroscopic properties are consistent with the data available in the literature.[4]

Fendiline (2.04)

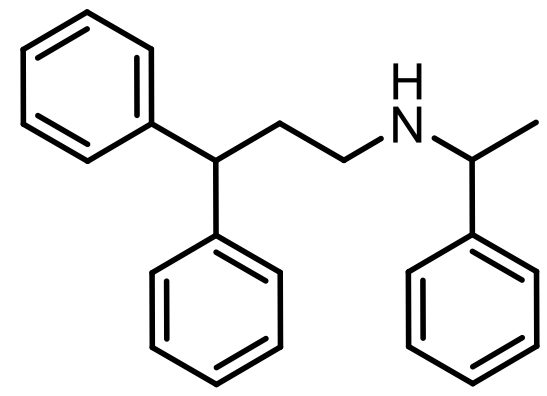

$C_{23}H_{25}N$
MW: 315.4600

Prepared following general procedure **4** using *N*-methyl-1-phenylethanamine (38.7 µL, 0.300 mmol, 1.00 equiv.), 1,1-diphenylethylene (58.3 µL, 0.330 mmol, 1.10 equiv.), trifluoroacetic acid (22.3 µL, 0.300 mmol, 1.00 equiv.) and paraformaldehyde (9.01 mg, 0.300 mmol, 1.00 equiv.). Purification by flash column chromatography (dichloromethane/DMA-mix 95:5 to 0:100) gave the title compound (44.0 mg, 0.139 mmol, 47%) as a colourless oil.

^{1}H NMR (400 MHz, $CDCl_3$) δ 7.35 – 7.11 (m, 15H), 4.00 (t, *J* = 7.8 Hz, 1H), 3.71 (q, *J* = 6.6 Hz, 1H), 2.56 – 2.37 (m, 2H), 2.32 – 2.12 (m, 2H), 1.53 (br s, 1H), 1.31 (d, *J* = 7.5 Hz, 3H).

^{13}C NMR (101 MHz, $CDCl_3$) δ 145.8, 145.1, 144.8, 128.5 (4C), 128.0 (2C), 127.9 (2C), 126.9, 126.6 (2C), 126.3 (2C), 126.2 (2C), 58.2, 49.1, 46.1, 36.1, 24.4.

The spectroscopic properties are consistent with the data available in the literature.[18]

N-Methyl-N-(2-((3-phenylpropyl)amino)ethyl)isoquinoline-5-sulfonamide (3.01)

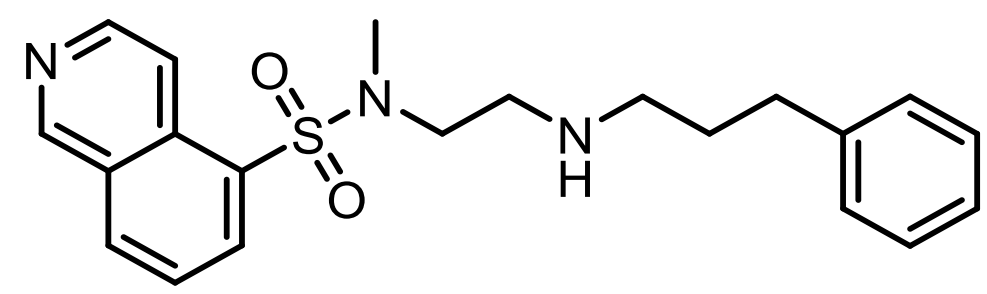

$C_{21}H_{25}N_3O_2S$
MW: 383.5100

Prepared following general procedure **3** using **S3.01a** (41.9 mg, 0.150 mmol, 1.50 equiv.), styrene (11.5 µL, 0.100 mmol, 1.00 equiv.), paraformaldehyde (4.5 mg, 0.15 mmol, 1.5 equiv.) and trifluoroacetic acid (15 µL, 0.20 mmol, 2.0 equiv.). Purification by flash column chromatography (dichloromethane/DMA-mix 95:5 to 0:100) gave the title compound (19.2 mg, 0.0501 mmol, 50%) as a pale-yellow oil.

^{1}H NMR (400 MHz, CDCl$_3$) δ 9.34 (br s, 1H), 8.68 (d, *J* = 6.1 Hz, 1H), 8.50 (d, *J* = 6.2 Hz, 1H), 8.38 (dd, *J* = 7.4, 1.2 Hz, 1H), 8.19 (d, *J* = 8.2 Hz, 1H), 7.70 (t, *J* = 7.8 Hz, 1H), 7.27 (d, *J* = 7.4 Hz, 3H), 7.24 – 7.13 (m, 3H), 3.27 (t, *J* = 6.2 Hz, 2H), 2.88 (s, 3H), 2.78 (t, *J* = 6.2 Hz, 2H), 2.59 (dt, *J* = 11.0, 7.5 Hz, 4H), 1.72 (p, *J* = 7.4 Hz, 2H), 1.63 (br s, 3H).

^{13}C NMR (101 MHz, CDCl$_3$) δ 153.4, 145.3, 142.1, 133.81, 133.76, 133.5 (2C), 132.0, 129.3 (2C), 128.5, 126.03, 126.0 (2C), 117.8, 49.7, 49.2, 47.3, 35.0, 33.6, 31.6.

HRMS (ESI$^+$): exact mass calculated for $[M+H]^+$ ($C_{21}H_{26}N_3O_2S$) requires *m/z* 384.1740, found *m/z* 384.1742.

IR (neat) ν_{max}: 2933, 2852, 1652, 1555, 1512, 1488, 1476, 1322, 1262, 1177, 1065, 955 cm^{-1}.

***N*-(2-((3-(4-Fluorophenyl)propyl)amino)ethyl)-*N*-methylisoquinoline-5-sulfonamide (3.02)**

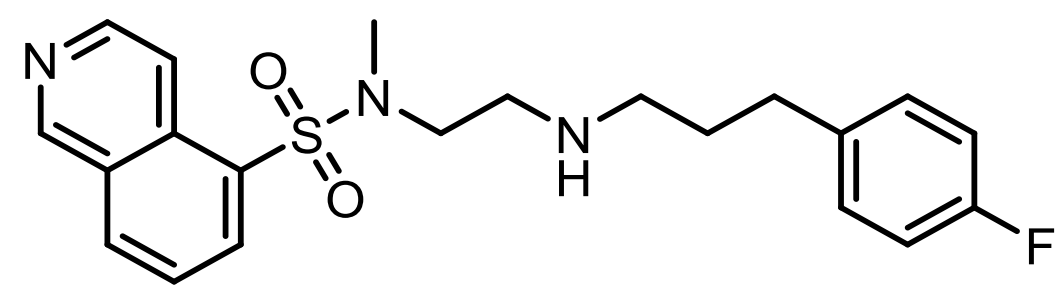

$C_{21}H_{24}FN_3O_2S$
MW: 401.5004

Prepared following general procedure **3** using **S3.01a** (41.9 mg, 0.150 mmol, 1.50 equiv.), 4-fluorostyrene (12.0 µL, 0.100 mmol, 1.00 equiv.), paraformaldehyde (4.5 mg, 0.15 mmol, 1.5 equiv.) and trifluoroacetic acid (15 µL, 0.20 mmol, 2.0 equiv.). Purification by flash column chromatography (dichloromethane/DMA-mix 95:5 to 0:100) gave the title compound (17.3 mg, 0.0431 mmol, 43%) as a pale-yellow oil.

^{1}H NMR (400 MHz, $CDCl_3$) δ 9.34 (s, 1H), 8.69 (d, *J* = 6.2 Hz, 1H), 8.50 (dd, *J* = 6.1, 1.0 Hz, 1H), 8.38 (dd, *J* = 7.4, 1.2 Hz, 1H), 8.20 (dt, *J* = 8.3, 1.1 Hz, 1H), 7.70 (dd, *J* = 8.2, 7.4 Hz, 1H), 7.14 – 7.07 (m, 2H), 7.01 – 6.91 (m, 2H), 3.27 (t, *J* = 6.2 Hz, 2H), 2.88 (s, 3H), 2.80 (t, *J* = 6.2 Hz, 2H), 2.58 (t, *J* = 6.9 Hz, 4H), 1.78 – 1.58 (m, 3H).

^{13}C NMR (101 MHz, $CDCl_3$) δ 153.4, 145.4, 137.6, 133.83, 133.80, 133.5, 132.0, 129.8 (2C), 129.8, 126.0 (2C), 117.8, 115.3, 115.1, 49.6, 49.0, 47.2, 35.0, 32.8, 31.7.

^{19}F coupling with the carbons was not observed.

^{19}F NMR (376 MHz, $CDCl_3$) δ -117.76.

HRMS (ESI$^+$): exact mass calculated for $[M+H]^+$ ($C_{21}H_{25}N_3O_2FS$) requires *m/z* 402.1646, found *m/z* 402.1642.

IR (neat) ν_{max}: 2928, 2857, 1673, 1616, 1563, 1509, 1455, 1328, 1217, 1134, 1040, 833, 762 cm^{-1}.

***N*-(2-((3-(4-Bromophenyl)propyl)amino)ethyl)-*N*-methylisoquinoline-5-sulfonamide (3.03)**

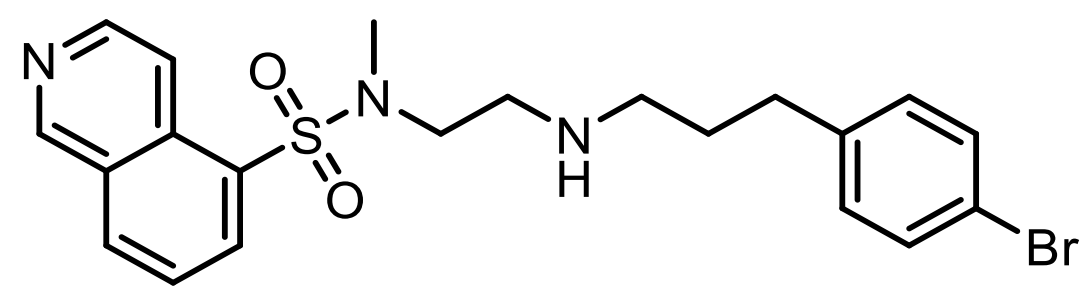

$C_{21}H_{24}BrN_3O_2S$
MW: 462.4060

Prepared following general procedure **3** using **S3.01a** (41.9 mg, 0.150 mmol, 1.50 equiv.), 4-bromostyrene (13.0 µL, 0.100 mmol, 1.00 equiv.), paraformaldehyde (4.5 mg, 0.15 mmol, 1.5 equiv.) and trifluoroacetic acid (15 µL, 0.20 mmol, 2.0 equiv.). Purification by flash column chromatography (dichloromethane/DMA-mix 95:5 to 0:100) gave the title compound (20.3 mg, 0.0439 mmol, 44%) as a pale-yellow oil.

^{1}H NMR (400 MHz, $CDCl_3$) δ 9.34 (br s, 1H), 8.69 (d, *J* = 6.2 Hz, 1H), 8.49 (dt, *J* = 6.2, 1.0 Hz, 1H), 8.37 (dd, *J* = 7.4, 1.3 Hz, 1H), 8.20 (dt, *J* = 8.3, 1.1 Hz, 1H), 7.70 (dd, *J* = 8.2, 7.4 Hz, 1H), 7.44 – 7.36 (m, 2H), 7.07 – 7.01 (m, 2H), 3.26 (t, *J* = 6.2 Hz, 2H), 2.87 (s, 3H), 2.77 (t, *J* = 6.2 Hz, 2H), 2.56 (td, *J* = 7.4, 2.3 Hz, 4H), 1.77 – 1.61 (m, 2H), 1.44 (br s, 2H).

^{13}C NMR (101 MHz, $CDCl_3$) δ 153.4, 145.3, 141.1, 133.80, 133.77, 133.5, 132.0, 131.5, 130.3 (2C), 129.3, 126.0 (2C), 119.7, 117.8, 49.7, 49.0, 47.2, 34.9, 33.0, 31.6.

HRMS (ESI$^+$): exact mass calculated for $[M+Na]^+$ ($C_{21}H_{24}N_3O_2{}^{79}BrSNa$)) requires *m/z* 462.0845, found *m/z* 462.0847.

IR (neat) ν_{max}: 2930, 2882, 2857, 1673, 1616, 1562, 1456, 1364, 1270, 1134, 101, 990, 918 cm^{-1}.

(*E*)-*N*-Methyl-*N*-(2-((3-(4-(pyrimidin-5-yl)phenyl)allyl)amino)ethyl)isoquinoline-5-sulfonamide (3.04)

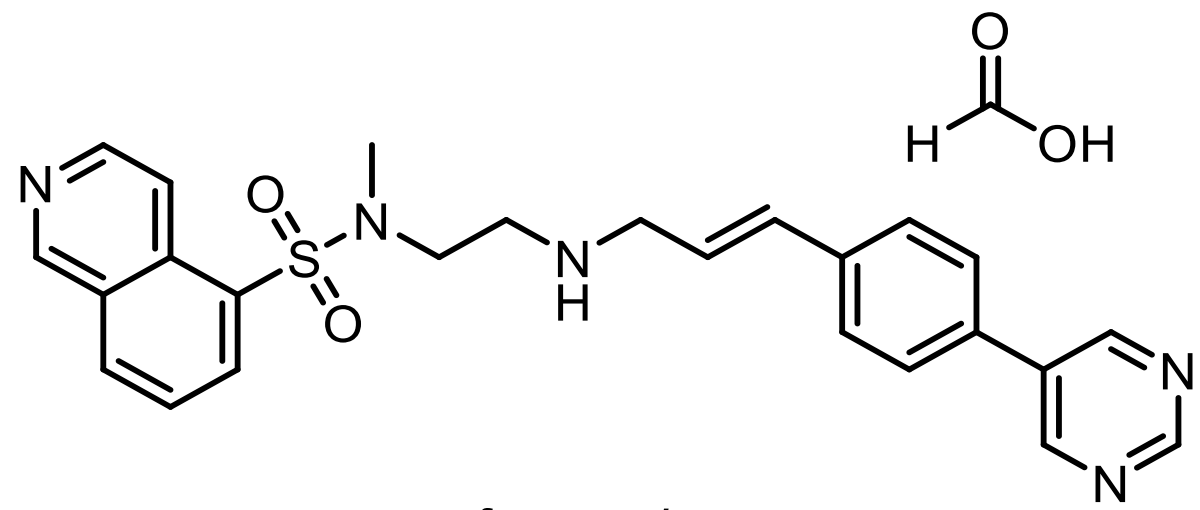

Prepared following general procedure **3** using **S3.01a** (41.9 mg, 0.150 mmol, 1.50 equiv.), 5-(4-ethynylphenyl)pyrimidine (18.0 mg, 0.100 mmol, 1.00 equiv.), paraformaldehyde (4.5 mg, 0.15 mmol, 1.5 equiv.) and trifluoroacetic acid (19 µL, 0.25 mmol, 2.5 equiv.). Purification by preparative high-performance liquid chromatography (water + 0.1% formic acid/acetonitrile 95:5 to 60:40) gave the title compound as a formate salt (22.8 mg, 0.0451 mmol, 45%) as a pale-yellow oil.

^{1}H NMR (400 MHz, CDCl$_3$) δ 9.31 (s, 1H), 9.19 (s, 1H), 8.92 (s, 1H), 8.66 (d, *J* = 6.2 Hz, 1H), 8.50 (dt, *J* = 6.1, 1.0 Hz, 1H), 8.34 (dd, *J* = 7.4, 1.2 Hz, 2H), 8.18 (dt, *J* = 8.3, 1.1 Hz, 1H), 7.69 (dd, *J* = 8.2, 7.4 Hz, 2H), 7.60 – 7.44 (m, 4H), 6.66 (d, *J* = 15.9 Hz, 1H), 6.34 (dt, *J* = 15.8, 6.8 Hz, 1H), 3.70 – 3.60 (m, 2H), 3.41 (t, *J* = 6.1 Hz, 2H), 3.09 (t, *J* = 6.1 Hz, 2H), 2.90 (s, 3H).

^{13}C NMR (101 MHz, CDCl$_3$) δ 166.2, 157.6, 154.9, 154.8, 153.3, 145.3, 136.8, 134.8, 134.1, 134.0, 133.8, 132.8, 132.0 (2C), 129.3, 127.8 (2C), 127.4, 126.2 (2C), 123.9, 117.7, 50.3, 47.9, 45.0, 35.5.

HRMS (ESI$^+$): exact mass calculated for [M+H]$^+$ ($C_{26}H_{26}N_4O_2S$) requires *m/z* 459.1849, found *m/z* 459.1846.

IR (neat) ν_{max}: 3031, 2970, 2359, 1738, 1672, 1615, 1451, 1415, 1202, 1202, 1072, 990 cm^{-1}.

(*E*)-*N*-Methyl-*N*-(2-((3-(4-(pyridin-3-yl)phenyl)allyl)amino)ethyl)isoquinoline-5-sulfonamide (3.05)

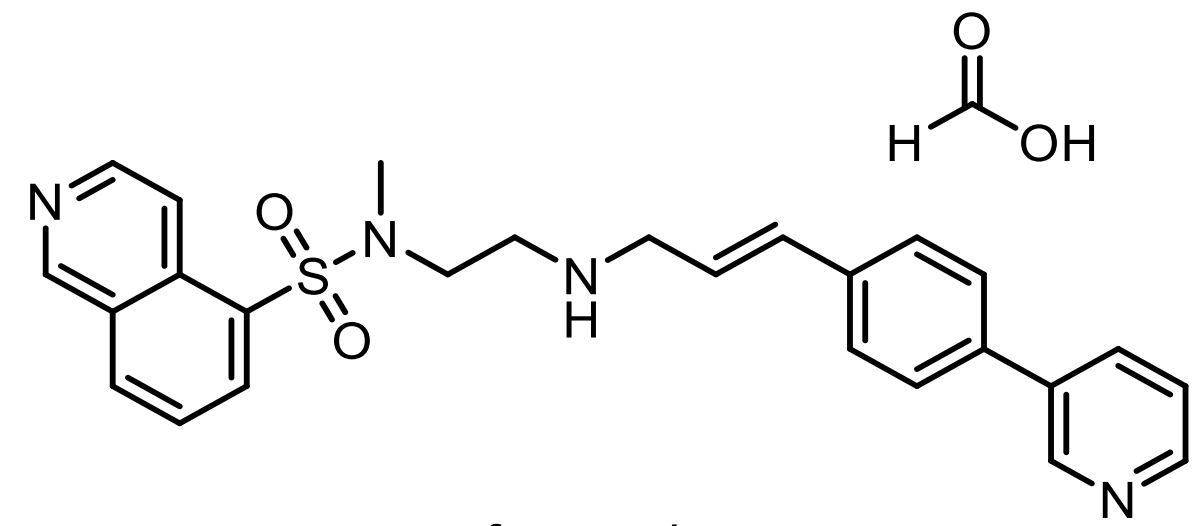

free amine
$C_{26}H_{26}N_4O_2S$
MW: 458.5800

Prepared following general procedure **3** using **S3.01a** (41.9 mg, 0.150 mmol, 1.50 equiv.), 3-(4-ethynylphenyl)pyridine (17.9 mg, 0.100 mmol, 1.00 equiv.), paraformaldehyde (4.5 mg, 0.15 mmol, 1.5 equiv.) and trifluoroacetic acid (19 µL, 0.25 mmol, 2.5 equiv.). Purification by preparative high-performance liquid chromatography (water + 0.1% formic acid/acetonitrile 95:5 to 60:40) gave the title compound as a formate salt (21.2 mg, 0.042 mmol, 42%) as a pale-yellow oil.

^{1}H NMR (400 MHz, CDCl$_3$) δ 9.32 (br s, 1H), 8.83 (dd, *J* = 2.4, 0.9 Hz, 1H), 8.67 (d, *J* = 6.3 Hz, 1H), 8.59 (dd, *J* = 4.8, 1.6 Hz, 1H), 8.52 (d, *J* = 1.0 Hz, 1H), 8.36 – 8.32 (m, 2H), 8.23 – 8.16 (m, 1H), 7.87 (ddd, *J* = 7.9, 2.4, 1.6 Hz, 1H), 7.69 (dd, *J* = 8.2, 7.4 Hz, 1H), 7.59 – 7.51 (m, 2H), 7.47 (d, *J* = 8.4 Hz, 2H), 7.38 (ddd, *J* = 7.9, 4.8, 0.9 Hz, 1H), 6.66 (d, *J* = 15.9 Hz, 1H), 6.32 (dd, *J* = 15.1, 7.4 Hz, 1H), 3.64 (d, *J* = 6.8 Hz, 2H), 3.41 (d, *J* = 6.1 Hz, 2H), 3.10 (d, *J* = 6.0 Hz, 2H), 2.90 (s, 3H). *The NH proton was not observed.*

^{13}C NMR (101 MHz, CDCl$_3$) δ 165.9, 153.3, 148.2, 147.7, 145.2, 137.5, 136.0, 135.1, 134.7, 134.7, 134.1, 132.8, 132.0, 129.3, 127.53 (2C), 127.50 (2C), 126.2, 123.9, 123.2, 117.8, 50.3, 47.9, 45.0, 35.5.

HRMS (ESI$^+$): exact mass calculated for $[M+H]^+$ ($C_{26}H_{26}N_4O_2S$) requires *m/z* 459.1849, found *m/z* 459.1846.

IR (neat) ν_{max}: 3030, 2970, 2922, 2359, 1738, 1670, 1582, 1474, 1327, 1158, 1043, 898 cm^{-1}.

1-((*S*)-3,3-Dimethyl-2-(12-(phenethylamino)dodecanamido)butanoyl)-4-hydroxy-*N*-(4-(4-methylthiazol-5-yl)benzyl)pyrrolidine-2-carboxamide (4.01)

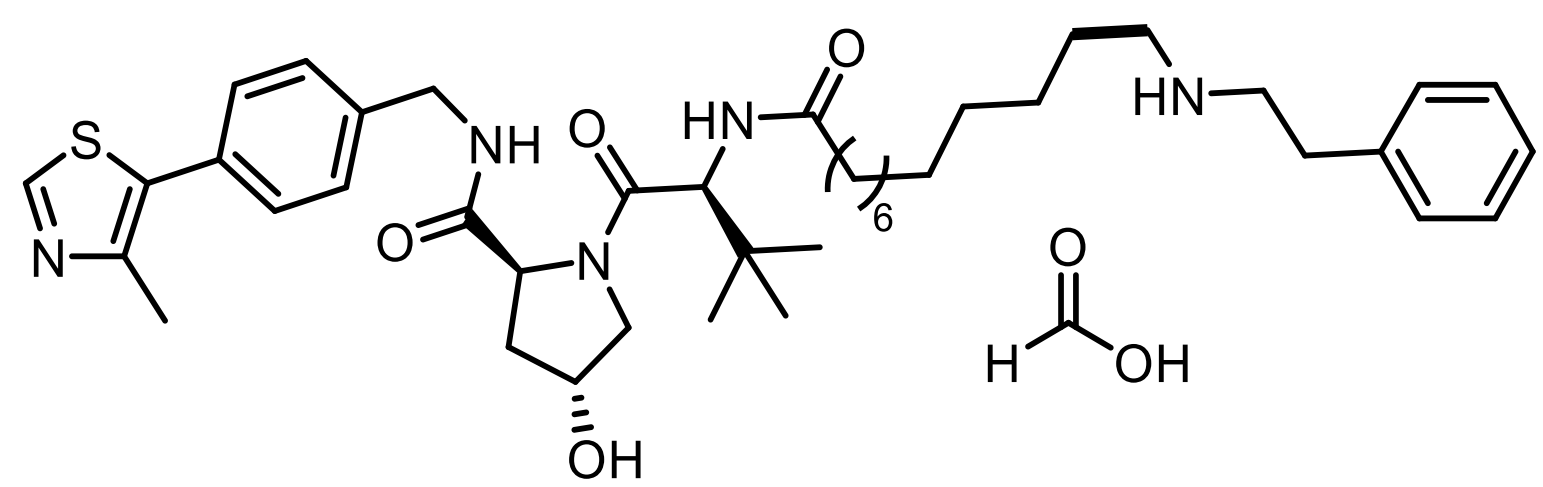

free amine
$C_{42}H_{61}N_5O_4S$
MW: 732.0410

Prepared following the slightly modified general procedure **2** using *N*-methyl-phenethylamine (29.1 µL, 0.200 mmol, 2.00 equiv.), **S4.01b** (59.7 mg, 0.100 mmol, 1.00 equiv.), trifluoroacetic acid (22.3 µL, 0.300 mmol, 3.00 equiv.) and paraformaldehyde (6.01 mg, 0.200 mmol, 2.00 equiv.). Purification by preparative high-performance liquid chromatography (water + 0.1% formic acid/acetonitrile 95:5 to 60:40) gave the title compound as a formate salt (24.12 mg, 0.031 mmol, 31%) as a pale-yellow oil.

^{1}H NMR (600 MHz, MeOD) δ 8.87 (s, 1H), 8.42 (s, 1H), 7.48 – 7.45 (m, 2H), 7.43 – 7.40 (m, 2H), 7.36 – 7.33 (m, 2H), 7.28 (m, 3H), 4.64 (s, 1H), 4.58 – 4.53 (m, 1H), 4.51 – 4.48 (m, 1H), 4.35 (dd, *J* = 15.2, 7.9 Hz, 1H), 3.90 (d, *J* = 11.1 Hz, 1H), 3.81 (dd, *J* = 10.9, 3.9 Hz, 1H), 3.24 (dd, *J* = 15.6, 7.8 Hz, 2H), 3.03 – 2.95 (m, 4H), 2.47 (s, 3H), 2.32 – 2.20 (m, 3H), 2.11 – 2.06 (m, 1H), 1.71 – 1.65 (m, 2H), 1.65 – 1.53 (m, 2H), 1.42 – 1.27 (m, 16H), 1.03 (s, 9H). *The NH and OH protons were not observed.*

^{13}C NMR (151 MHz, MeOD) δ 176.0, 174.5, 172.3, 168.7, 152.8, 149.0, 140.3, 137.7, 133.4, 131.5, 130.4 (2C), 130.0 (2C), 129.7 (2C), 128.9 (2C), 128.3, 71.1, 60.8, 58.9, 58.0, 49.9, 43.7, 38.9, 36.7, 36.6, 35.3, 33.4, 30.6, 30.5 (2C), 30.4, 30.3, 30.2, 27.6, 27.3, 27.1 (3C), 27.0, 15.8.

HRMS (ESI$^+$): exact mass calculated for $[M+H]^+$ ($C_{42}H_{62}N_5O_4S$) requires *m/z* 732.4517, found *m/z* 732.4495.

IR (neat) ν_{max}: 3293, 2924, 2853, 1626, 1536, 1479, 1435, 1395, 1371, 1340, 1278, 1219, 1200, 1121, 1087, 1030, 847, 800, 752, 665, 537 cm^{-1}.

12-((3-((1-Benzyl-1*H*-indazol-3-yl)oxy)propyl)amino)-*N*-(2-(2,6-dioxopiperidin-3-yl)-1,3-dioxoisoindolin-5-yl)dodecanamide (4.02)

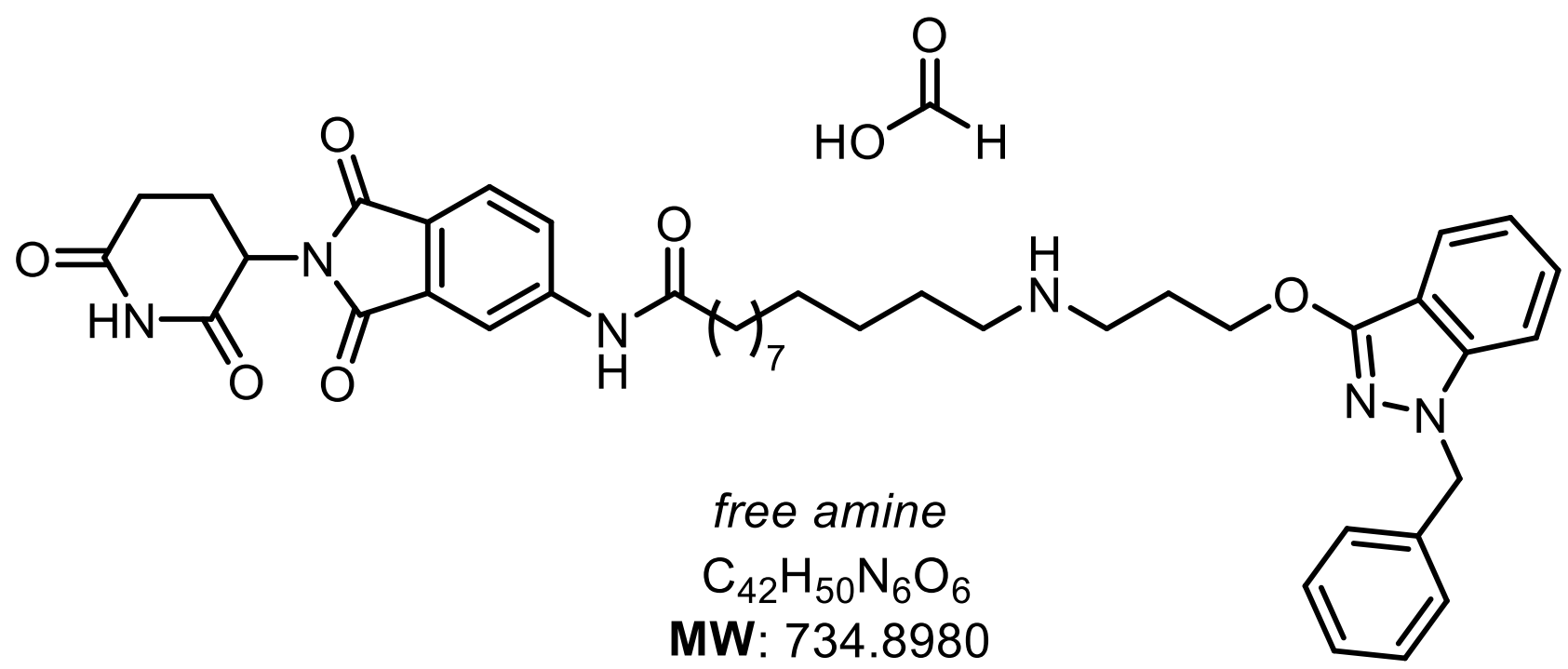

Prepared following the slightly modified general procedure **2** using **S1.11a** (118 mg, 0.400 mmol, 2.00 equiv.), **S4.02b** (87.9 mg, 0.200 mmol, 1.00 equiv.), trifluoroacetic acid (44.6 µL, 0.600 mmol, 3.00 equiv.) and paraformaldehyde (12.0 mg, 0.400 mmol, 2.00 equiv.). Purification by preparative high-performance liquid chromatography (water + 0.1% formic acid/acetonitrile 95:5 to 60:40) gave the title compound as a formate salt (78.1 mg, 0.100 mmol, 50%) as a pale-yellow oil.

^{1}H NMR (400 MHz, CDCl$_3$) δ 9.44 (s, 1H), 8.81 (d, *J* = 8.5 Hz, 1H), 8.53 (s, 1H), 7.71 – 7.65 (m, 1H), 7.62 (d, *J* = 8.1 Hz, 1H), 7.55 – 7.48 (m, 1H), 7.32 – 7.27 (m, 1H), 7.24 – 7.20 (m, 3H), 7.19 – 6.96 (m, 5H), 5.35 (s, 2H), 4.96 – 4.88 (m, 1H), 4.46 (t, *J* = 5.7 Hz, 2H), 3.12 (t, *J* = 7.3 Hz, 2H), 2.84 (t, *J* = 7.8 Hz, 2H), 2.47 – 2.40 (m, 3H), 2.30 (dd, *J* = 13.4, 7.0 Hz, 2H), 2.16 – 2.09 (m, 1H), 1.80 – 1.60 (m, 5H), 1.42 – 1.15 (m, 16H).

^{13}C NMR (101 MHz, CDCl$_3$) δ 172.6, 171.4, 169.3, 168.4, 168.3, 166.9, 155.9, 141.7, 138.0, 137.5, 136.5, 131.3, 128.8 (2C), 127.7, 127.6, 127.1 (2C), 125.4, 120.1, 119.5, 118.5, 115.5, 112.8, 109.0, 66.4, 52.4, 49.4, 47.9, 45.1, 38.2, 31.6, 29.5, 29.4, 29.3, 29.2, 29.1, 29.0, 26.7, 26.4 (2C), 25.3, 22.8.

HRMS (ESI$^+$): exact mass calculated for $[M+H]^+$ ($C_{42}H_{51}N_6O_6$) requires *m/z* 735.3864, found *m/z* 735.3839.

IR (neat) ν_{max}: 2924, 2853, 1701, 1617, 1526, 1478, 1443, 1396, 1349, 1292, 1258, 1186, 1116, 1102, 1027, 822, 745, 666, 534 cm^{-1}.

11-((3-(9,10-Ethanoanthracen-9(10*H*)-yl)propyl)amino)-*N*-(2-(2,6-dioxopiperidin-3-yl)-1,3-dioxoisoindolin-5-yl)undecanamide (4.03)

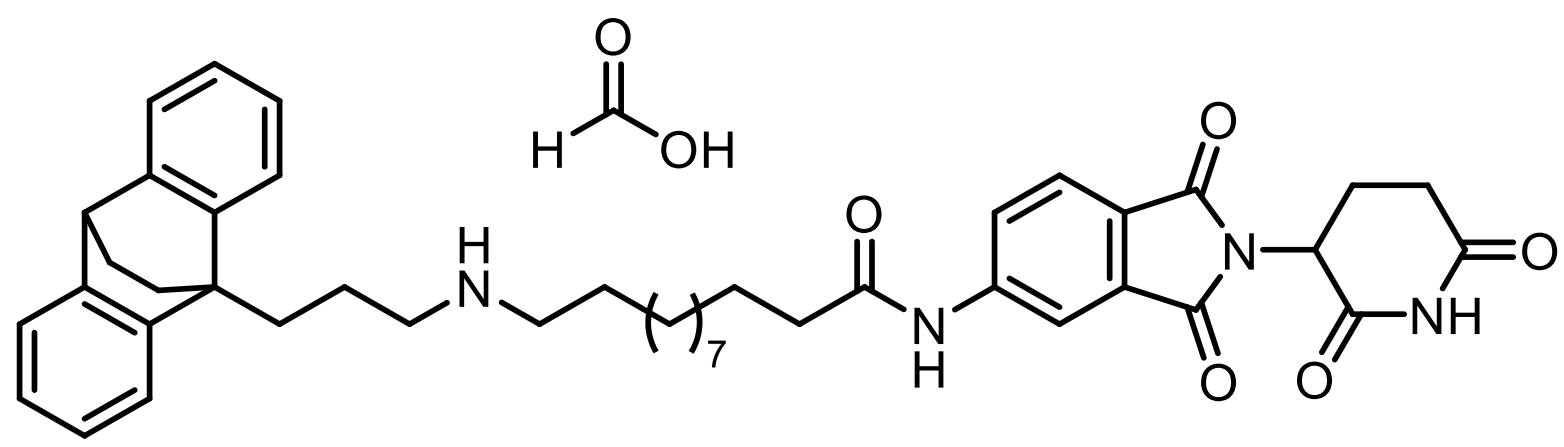

free amine
$C_{44}H_{52}N_4O_5$
MW: 716.9230

Prepared following general procedure **2** using Maprotiline hydrochloride (31.4 mg, 0.100 mmol, 1.00 equiv.), **S3.03b** (48.3 mg, 0.110 mmol, 1.10 equiv.) and paraformaldehyde (3.00 mg, 0.100 mmol, 1.00 equiv.). Purification by preparative high-performance liquid chromatography (water + 0.1% formic acid/acetonitrile 95:5 to 60:40) gave the title compound as a formate salt (20.6 mg, 0.027 mmol, 27%) as a pale-yellow oil.

^{1}H NMR (400 MHz, CDCl$_3$) δ 9.45 (s, 1H), 8.82 (d, *J* = 8.5 Hz, 1H), 8.56 (s, 1H), 7.72 – 7.67 (m, 1H), 7.52 (d, *J* = 7.3 Hz, 1H), 7.23 (d, *J* = 7.2 Hz, 2H), 7.15 (d, *J* = 7.1 Hz, 2H), 7.12 – 7.01 (m, 5H), 4.91 (dd, *J* = 12.2, 5.3 Hz, 1H), 4.24 (s, 1H), 3.19 – 3.11 (m, 2H), 2.94 – 2.86 (m, 2H), 2.83 – 2.62 (m, 2H), 2.51 – 2.36 (m, 4H), 2.14 (dd, *J* = 15.1, 9.7 Hz, 3H), 1.74 (dd, *J* = 14.4, 7.5 Hz, 4H), 1.70 – 1.60 (m, 2H), 1.51 – 1.44 (m, 2H), 1.44 – 1.10 (m, 16H).

^{13}C NMR (176 MHz, CDCl$_3$) δ 172.6, 171.3, 169.3, 168.5, 168.3, 166.9, 144.9 (2C), 144.8 (2C), 138.0, 136.6, 131.3, 125.6 (2C), 125.5 (2C), 125.4, 123.6 (2C), 121.2 (2C), 118.5, 115.5, 49.4, 48.5, 47.8, 44.6 (2C), 38.2, 31.6, 29.4 (2C), 29.3, 29.2, 29.1, 29.0, 28.5, 27.7, 26.8, 26.3 (2C), 25.4, 22.8, 21.9.

HRMS (ESI$^+$): exact mass calculated for $[M+H]^+$ ($C_{44}H_{53}N_4O_5$) requires *m/z* 717.4010, found *m/z* 717.3998.

IR (neat) ν_{max}: 2925, 2853, 1702, 1617, 1526, 1478, 1456, 1425, 1397, 1349, 1323, 1293, 1260, 1198, 1117, 1030, 747 cm^{-1}.

Dimethyl *N*-(5-phenylpentyl)glycylprolylphenylalanyl-1-tosyltryptophylglutamate (4.04)

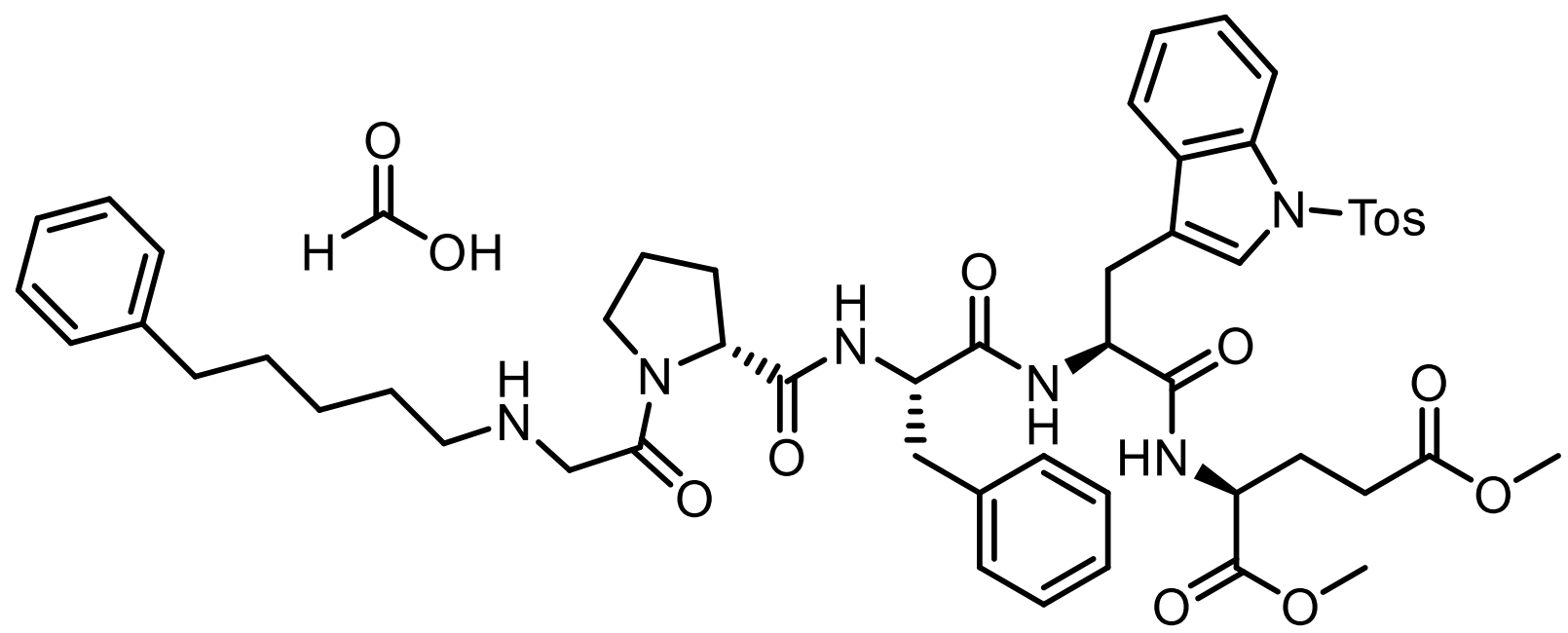

free amine
$C_{52}H_{62}N_6O_{10}S$
MW: 963.1600

Prepared following general procedure **2** using **4.04a** (83.1 mg, 0.100 mmol, 1.00 equiv.), 4-phenyl-1-butene (16.5 µL, 0.110 mmol, 1.10 equiv.), and paraformaldehyde (3.00 mg, 0.100 mmol, 1.00 equiv.). Purification by preparative high-performance liquid chromatography (water + 0.1% formic acid/acetonitrile 95:5 to 50:50) gave the title compound as a formate salt (54.5 mg, 0.054 mmol, 54%) as a colourless oil.

Rotameric effects are visible in the measured NMR spectra.

^{1}H NMR (400 MHz, CDCl$_3$) δ 8.34 (s, 1H), 7.91 – 7.87 (m, 2H), 7.66 (dt, *J* = 18.9, 9.3 Hz, 3.3H), 7.51 – 7.29 (m, 2.7H), 7.25 – 6.99 (m, 10H), 6.77 (d, *J* = 7.4 Hz, 1H), 6.46 – 5.99 (br s, 3H), 4.76 – 4.48 (m, 2H), 4.48 – 4.32 (m, 1H), 4.23 – 4.07 (m, 1H), 3.98 (app d, *J* = 14.1 Hz, 1H), 3.72 – 3.49 (m, 6H), 3.20 – 3.16 (m, 4H), 3.02 (dd, *J* = 14.8, 5.8 Hz, 1H), 2.93 – 2.78 (m, 3H), 2.53 (t, *J* = 7.4 Hz, 2H), 2.38 – 2.13 (m, 6H), 2.11 – 2.00 (m, 1H), 1.99 – 1.81 (m, 3H), 1.82 – 1.65 (m, 3H), 1.65 – 1.42 (m, 4H), 1.42 – 1.27 (m, 2H).

^{13}C NMR (101 MHz, CDCl$_3$) δ 173.8, 171.9, 171.5, 171.2, 170.9, 168.0, 166.5, 144.9, 142.1, 138.0, 135.3, 135.2, 130.9, 130.0, 129.9 (2C), 129.3, 128.5 (2C), 128.5 (2C), 128.4 (2C), 126.9, 126.6, 125.9, 124.9, 124.3, 123.4, 120.0, 118.3, 113.6, 61.9, 55.2, 53.8, 52.7, 52.1, 51.9, 48.6, 48.4, 47.1, 36.9, 35.7, 30.9, 30.0, 29.6, 27.4, 26.9, 26.5, 26.4, 24.3, 21.6.

HRMS (ESI$^+$): exact mass calculated for $[M+H]^+$ ($C_{52}H_{63}N_6O_{10}S$) requires *m/z* 963.4321, found *m/z* 963.4311.

IR (neat) ν_{max}: 3292, 3024, 2828, 1736, 1648, 1495, 1364, 1169, 974, 699, 595, 486 cm^{-1}.

Methyl (*S*)-2-phenyl-3-((*R*)-1-((5-phenylpentyl)glycyl)pyrrolidine-3-carboxamido)propanoate (4.05)

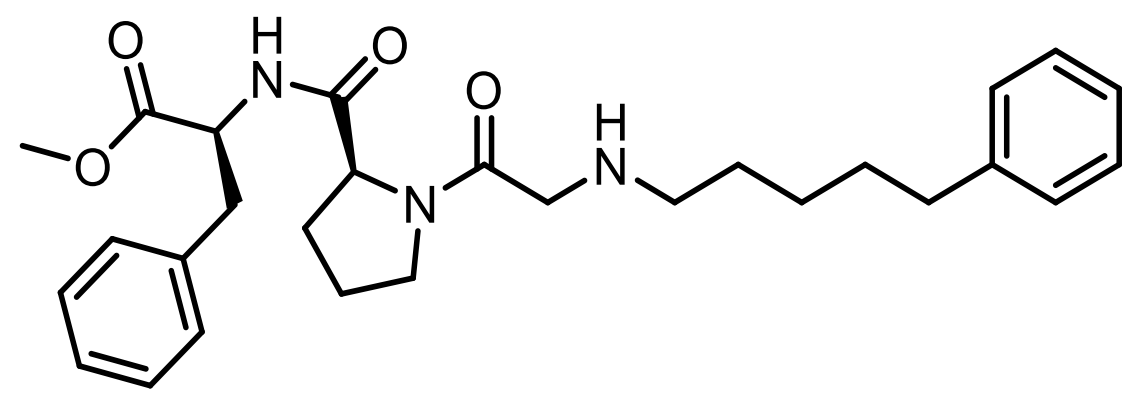

$C_{28}H_{37}N_3O_4$
MW: 479.6210

Prepared following general procedure **1** using **S4.05a** (69.5 mg, 0.200 mmol, 1.00 equiv.), 4-phenyl-1-butene (33.0 µL, 0.220 mmol, 1.10 equiv.) and paraformaldehyde (6.0 mg, 0.20 mmol, 1.0 equiv.). Purification by flash column chromatography (dichloromethane/DMA-mix 95:5 to 0:100) gave the title compound (61.4 mg, 0.130 mmol, 64%) as a yellow oil.

Rotameric effects are visible in the measured NMR spectra.

^{1}H NMR (600 MHz, CDCl$_3$) δ 7.32 – 7.26 (m, 4H), 7.25 – 7.07 (m, 6H), 4.84 – 4.71 (m, 1H), 4.57 (d, *J* = 7.4 Hz, 1H), 3.67 (s, 3H), 3.56 – 3.44 (m, 2H), 3.40 – 3.32 (m, 2H), 3.21 – 2.92 (m, 2H), 2.64 – 2.43 (m, 4H), 2.33 – 2.26 (m, 1H), 2.16 – 1.91 (m, 2H), 1.87 – 1.77 (m, 3H), 1.64 (dt, *J* = 15.4, 7.7 Hz, 2H), 1.58 – 1.45 (m, 2H), 1.42 – 1.33 (m, 2H).

^{13}C NMR (151 MHz, CDCl$_3$) δ 171.9, 171.8, 170.9, 142.8 (2C), 136.3, [130.4, 129.4, 129.0] (2C), [128.6, 128.5] (2C), 128.4 (2C), [127.5, 127.2, 127.1] (2C), [126.1, 125.8], 60.9, 60.7, 59.9, 59.8, 57.8, 53.7, 53.5, 52.9, 52.7, 52.4, 51.7, 51.6, 50.1, 50.0, 47.0, 46.4, 42.4, 39.2, 38.1, 38.0, 37.6, 36.0, 32.1, 31.6, 30.6, 30.2, 30.1, 27.4, 27.1, 27.1, 25.1, 24.9, 22.3.

HRMS (ESI$^+$): exact mass calculated for $[M+H]^+$ ($C_{28}H_{38}N_3O_4$) requires *m/z* 480.2857, found *m/z* 480.2850.

IR (neat) ν_{max}: 3283, 3053, 3033, 2967, 2855, 1732, 1659, 1322, 1188, 1052, 733, 722 cm^{-1}.

Dimethyl (5-phenylpentyl)glycylprolylphenylalanylglutamate (4.06)

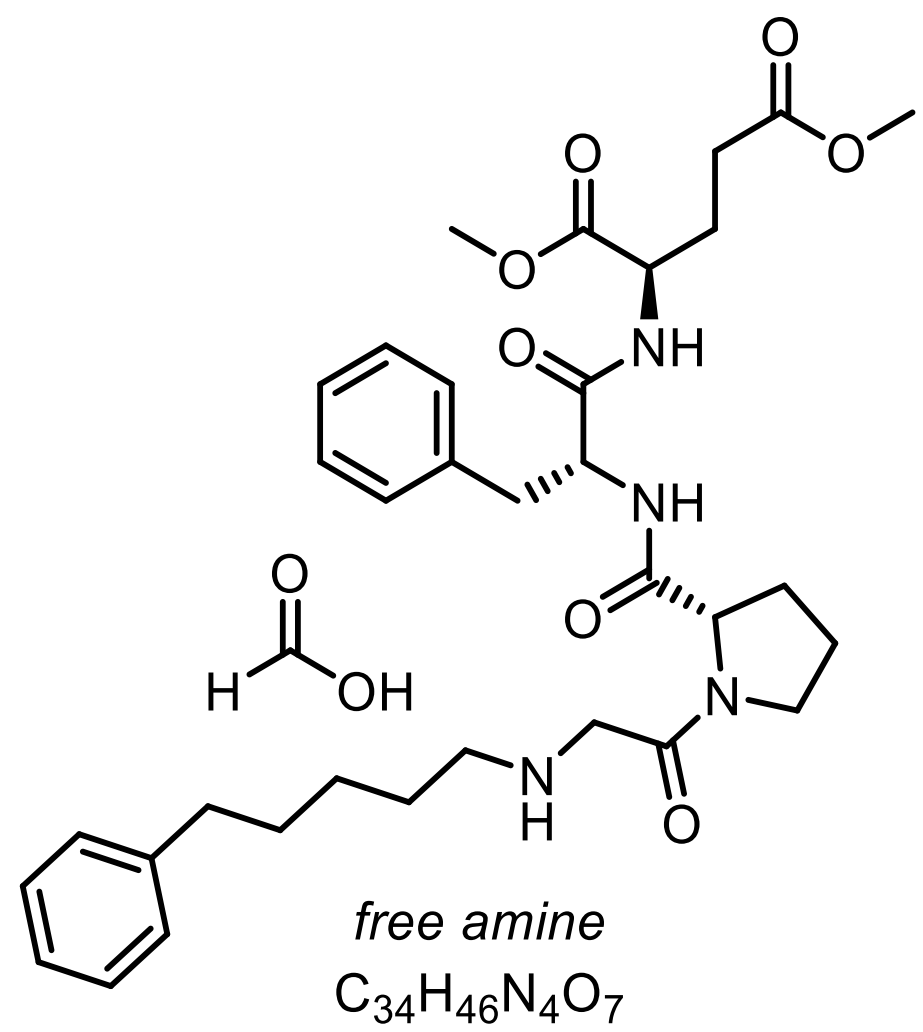

free amine
$C_{34}H_{46}N_4O_7$
MW: 622.7630

Prepared following general procedure **1** using **S4.06a** (176 mg, 0.300 mmol, 1.00 equiv.), 4-phenyl-1-butene (58.6 µL, 0.330 mmol, 1.10 equiv.), and paraformaldehyde (9.0 mg, 0.30 mmol, 1.0 equiv.). Purification by preparative high-performance liquid chromatography (water + 0.1% formic acid/acetonitrile 95:5 to 50:50) gave the title compound as formate salt (112 mg, 0.168 mmol, 56%) as a colourless oil.

Rotameric effects are visible in the measured NMR spectra.

^{1}H NMR (400 MHz, $CDCl_3$) δ 8.33 (s, 1.2H), 7.92 (d, *J* = 9.1 Hz, 0.6H), 7.37 – 7.11 (m, 10.6H), 7.05 (t, *J* = 10.8 Hz, 0.7H), 4.85 (ddd, *J* = 12.0, 9.1, 4.4 Hz, 1H), 4.53 (td, *J* = 7.5, 5.1 Hz, 0.8H), 4.43 – 4.30 (m, 1.2H), 4.05 – 3.90 (m, 5H), 3.75 – 3.72 (m, 3H), 3.68 – 3.66 (m, 3H), 3.53 – 3.44 (m, 1.7H), 3.40 – 3.25 (m, 1.6H), 3.05 – 2.89 (m, 1.6H), 2.69 – 2.59 (m, 0.7H), 2.56 – 2.45 (m, 1.2H), 2.45 – 2.30 (m, 1H), 2.25 – 1.95 (m, 3.6H), 1.88 – 1.62 (m, 6H), 1.59 – 1.37 (m, 2.5H).

^{13}C NMR (101 MHz, $CDCl_3$) δ 174.6, 172.1, 171.7, 170.8, 166.6, 142.1, 138.3, 129.3 (2C), 128.5 (2C), 128.5 (2C), 128.4 (2C), 126.6, 126.0, 61.6, 54.4, 52.5, 52.1, 51.9, 48.4, 48.1, 46.9, 36.5, 35.7, 30.9, 30.3, 29.6, 27.3, 26.3, 26.2, 23.9.

HRMS (ESI$^+$): exact mass calculated for $[M+H]^+$ ($C_{34}H_{47}N_4O_7$) requires *m/z* 623.3439, found *m/z* 623.3431.

IR (neat) ν_{max}: 3294, 3026, 2949, 2857, 1737, 1652, 1436, 1203, 1029, 749, 700, 487 cm^{-1}.

Dimethyl (10-(2-((*R*)-4-(4-chlorophenyl)-2,3,9-trimethyl-6*H*-thieno[3,2-*f*][1,2,4]triazolo[4,3-*a*][1,4]diazepin-6-yl)-*N*-methylacetamido)decyl)glycyl-*L*-prolyl-*L*-phenylalanyl-*L*-glutamate (4.07)

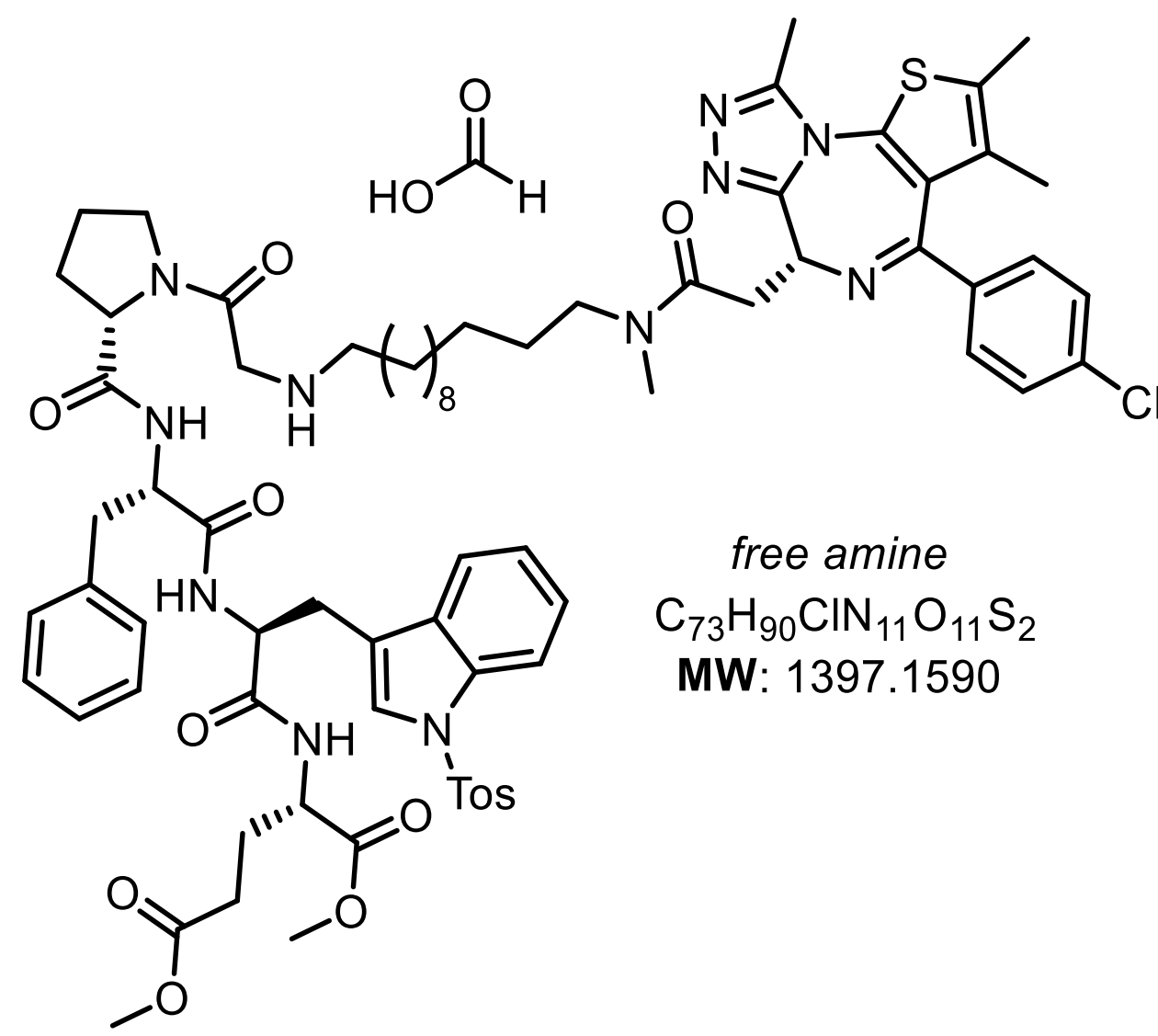

Prepared following general procedure **1** using **S4.04a** (83.1 mg, 0.100 mmol, 1.00 equiv.), **S1.19b** (62.3 mg, 0.110 mmol, 1.10 equiv.), and paraformaldehyde (3.0 mg, 0.10 mmol, 1.0 equiv.). Purification by preparative high-performance liquid chromatography (water + 0.1% formic acid/acetonitrile 95:5 to 60:40) gave the title compound as a formate salt (38.9 mg, 0.027 mmol, 27%) as a pale-yellow oil.

Rotameric effects are visible in the measured NMR spectra.

^{1}H NMR (600 MHz, CDCl$_3$) δ 8.01 – 7.86 (m, 1H), 7.80 – 7.67 (m, 4H), 7.45 – 7.30 (m, 9H), 7.25 – 7.08 (m, 8H), 6.67 – 6.46 (m, 1H), 4.92 – 4.85 (m, 1H), 4.85 – 4.77 (m, 1H), 4.59 (s, 1H), 4.44 – 4.36 (m, 1H), 4.25 – 4.07 (m, 2H), 3.80 – 3.55 (m, 10H), 3.52 – 3.28 (m, 5H), 3.20 (s, 1.5H), 3.15 – 3.02 (m, 4H), 2.98 (s, 1.5H), 2.90 (app t, *J* = 12.9 Hz, 1H), 2.69 – 2.64 (m, 3H), 2.44 – 2.36 (m, 4H), 2.35 – 2.23 (m, 4H), 2.13 – 1.92 (m, 4H), 1.92 – 1.83 (m, 2H), 1.78 – 1.73 (m, 1H), 1.72 – 1.60 (m, 4H), 1.54 (br s, 1H), 1.48 – 1.17 (m, 16H).

^{13}C NMR (151 MHz, CDCl$_3$) δ [173.9, 173.8], 171.8, [171.15, 171.14], [170.8, 170.7], 170.6, [170.4, 170.3], 166.4, 163.7, [163.1, 162.9], 156.2, [149.98, 149.91], [144.98, 144.97], 138.3, [137.07, 137.03], 136.7, 135.3, 135.2, [132.4, 132.3], [131.1, 131.0], [130.81, 130.79], [130.76, 130.74], [130.72, 130.71], 130.0 (4C), 129.3 (2C), [128.8, 128.7] (2C), 128.4 (2C), 126.9 (2C), 126.5, 124.9,

124.3, 123.4, 120.2, [118.3, 118.2], [113.5, 112.6], 62.1, 54.8, 54.6, [52.81, 52.80], 52.1, 51.9, 50.5, [48.93, 48.91], 48.6, 48.1, 47.2, 37.0, 35.9, 35.7, 35.3, 33.9, 29.9, 29.5 (3C), [29.3, 29.2], 28.77, [27.69, 27.65], 27.5, [27.14, 27.12], 27.0, 26.9, [26.87, 26.83], 24.4, 21.7, [14.55, 14.52], [13.22, 13.21], [12.01, 11.97].

HRMS (ESI$^+$): exact mass calculated for $[M+H]^+$ ($C_{73}H_{91}{}^{35}ClN_{11}O_{11}S_2$) requires *m/z* 1396.6024, found *m/z* 1396.6019.

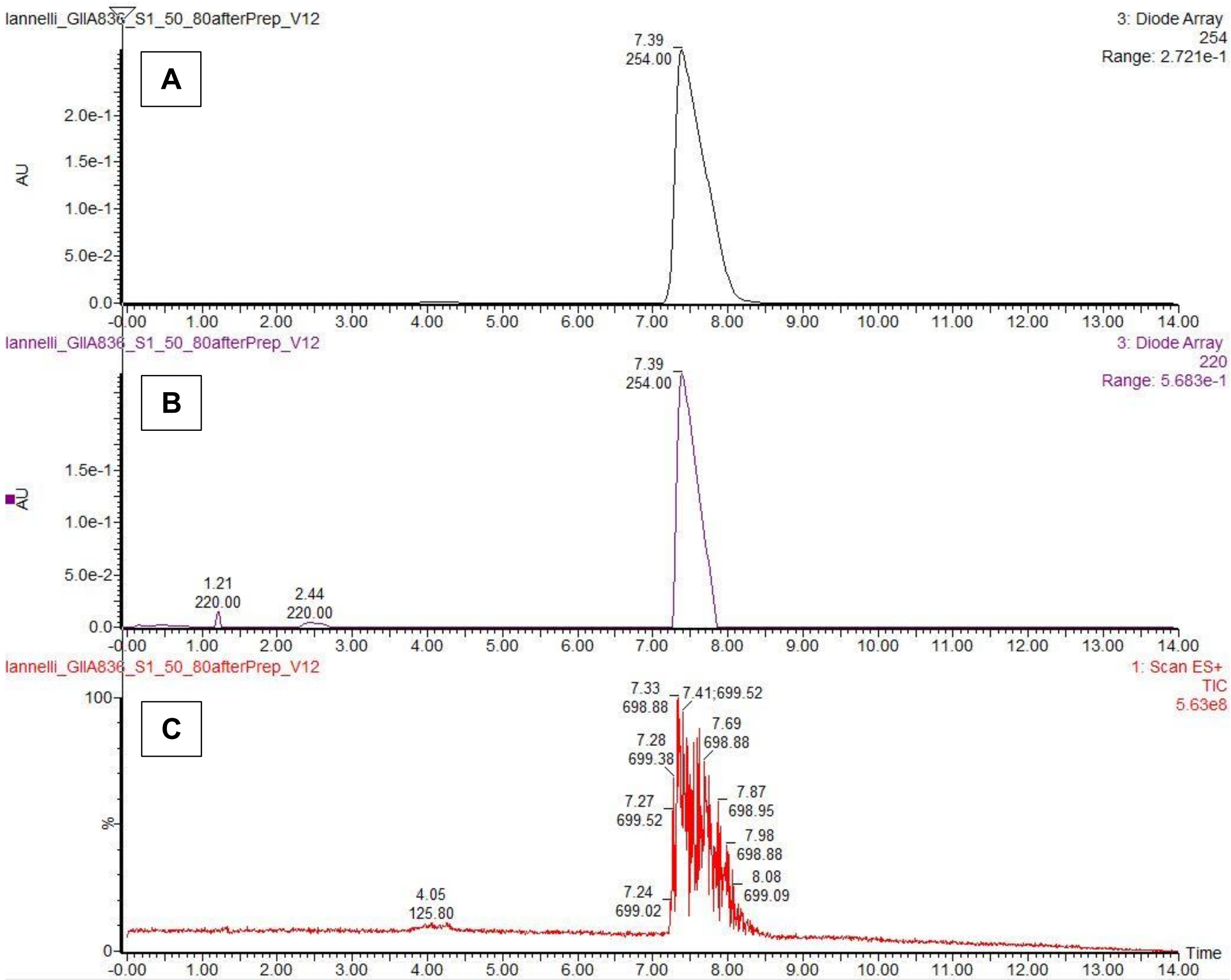

Supplementary Fig. S5. Purity of compound 3.07: A) LC trace of purified compound [water + 0.1% formic acid/acetonitrile 50:50 to 20:80, λ = 254 nm, XBridge^{TM} (5 µm, 4.6 × 10mm) – analytical column]. B) LC trace of purified compound [water + 0.1% formic acid/acetonitrile 50:50 to 20:80, λ = 220 nm, XBridge^{TM} (5 µm, 4.6 × 10mm) – analytical column]. C) MS trace of purified compound: $[M/2+H]^+$.

6. Mechanistic studies

Our proposed mechanistic picture is depicted in **Supplementary Fig. S6A**. Here, the nucleophilic addition of a π-system to an *in-situ* generated iminium ion (**5**) initiates an internal redox process through a 1,5-hydride transfer, forming a second intermediate iminium ion (**6**), which yields the desired secondary amine upon quenching and concomitant hydrolysis. The reaction mechanism was investigated through a series of experiments, beginning with isotopic labeling (**Supplementary Fig. S6B**). The use of deuterated formaldehyde provided clear evidence of this reagent's role as a methylene donor (**Supplementary Fig. S6B**, top). However, despite conducting the experiment with an excess of fully deuterated CD_2O, we only observed 85% deuterium incorporation in the final product (product **III**). This observation points towards a mechanism in which non-deuterated formaldehyde is formed *in situ* through hydrolysis of iminium ion **6**—a process facilitated by the water produced during the initial condensation to form **5**. As expected, the use of both deuterated formaldehyde and an amine perdeuterated on the methyl group led to the trideuterated product **IV** with 97% incorporation of deuterium in the expected positions (**Supplementary Fig. S6B**, bottom). Notably, the full incorporation of one deuterium atom at the β-position of the former alkene additionally unequivocally supports an intramolecular hydride/deuteride transfer as the formal reduction event. Key intermediates **6** and **6** were detected using 2D NMR experiments, which also revealed the presence of the hydrolyzed final product before work-up, thus substantiating the operation of *in-situ* hydrolytic recycling of formaldehyde (cf. labeling experiments, **Supplementary Fig. S6B**). Validation of the latter was also provided by the reactions, in which a sub-stoichiometric amount of paraformaldehyde enabled the formation of the desired product in modest to good yields (**Supplementary Fig. S6C**).

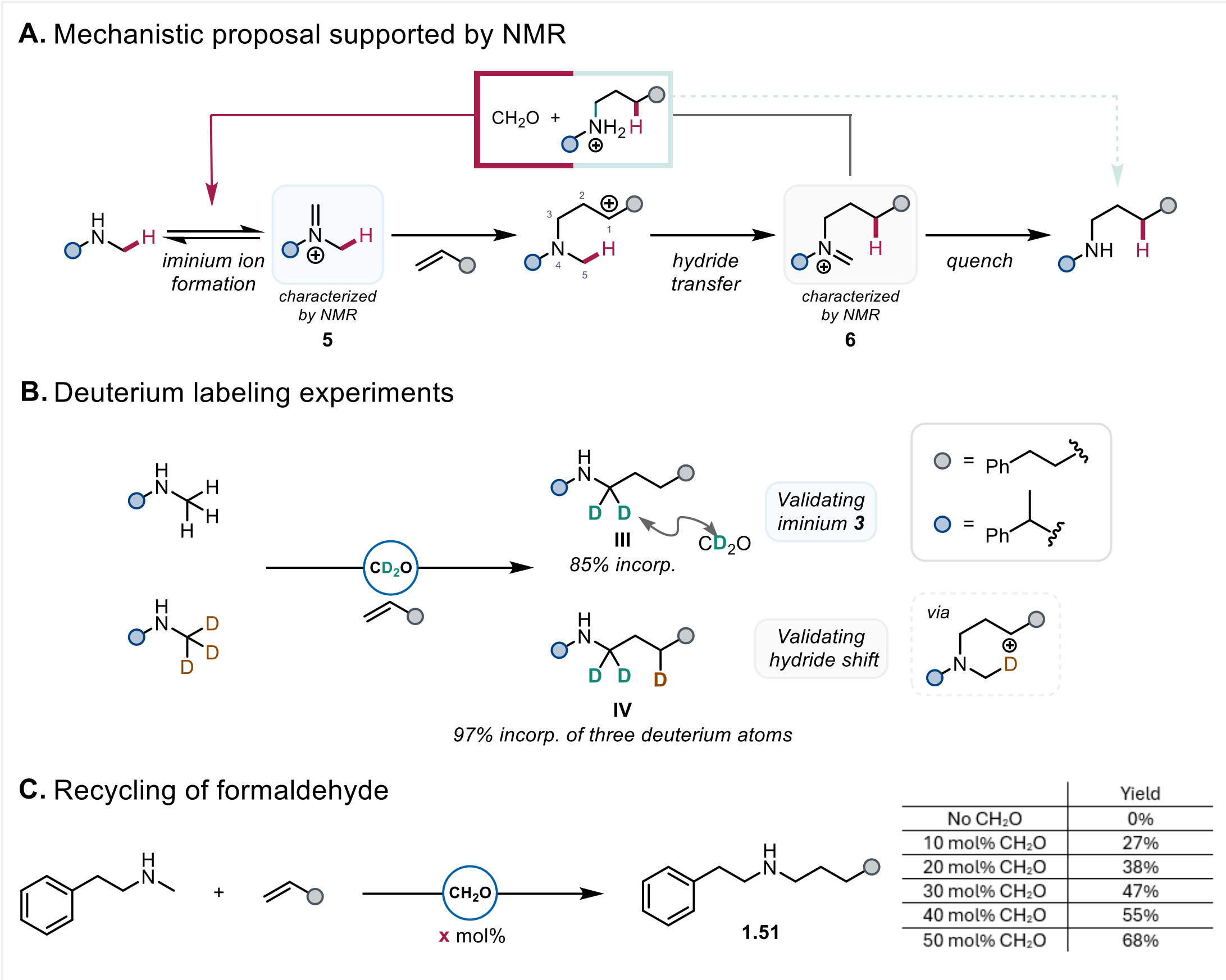

	Yield
No CH_2O	0%
10 mol% CH_2O	27%
20 mol% CH_2O	38%
30 mol% CH_2O	47%
40 mol% CH_2O	55%
50 mol% CH_2O	68%

Supplementary Fig. S6. **Mechanistic studies. A.** The proposed mechanistic picture is further corroborated by identification of intermediates **5** and **6** by 2D NMR. **B.** Deuterium labeling experiments support the formation of iminium **5** and the hydride transfer pathway. **C.** Owing to the *in-situ* hydrolysis of **6**, formaldehyde can be partially recycled.

6.1 Deuterium labeling

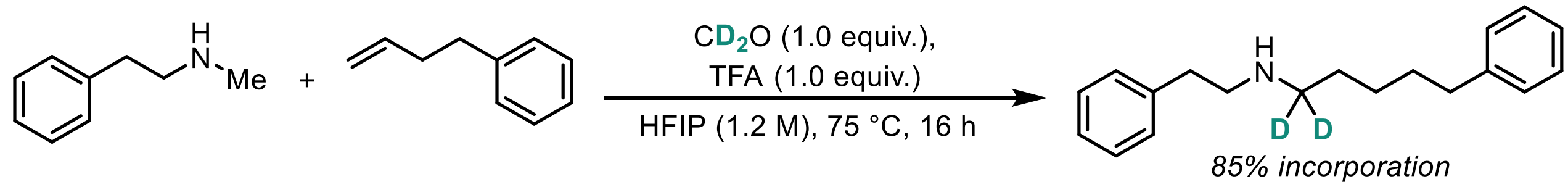

Prepared following a slightly modified general procedure **4**, using *N*-methyl-phenethylamine (43.6 µL, 0.30 mmol, 1.00 equiv.), 4-phenyl-1-butene (58.6 µL, 0.33 mmol, 1.10 equiv.), trifluoroacetic acid (22.3 µL, 0.30 mmol, 1.00 equiv.) and deuterated-paraformaldehyde (9.01 mg, 0.30 mmol, 1.0 equiv.). Purification by flash column chromatography (dichloromethane/DMA-mix 95:5 to 0:100) gave the title compound (60.0 mg, 0.224 mmol, 75%) as a colourless oil.

^{1}H NMR (400 MHz, $CDCl_3$) δ 7.32 – 7.27 (m, 3H), 7.26 – 7.25 (m, 1H), 7.24 – 7.13 (m, 6H), 2.95 – 2.78 (m, 4H), 2.62 – 2.55 (m, 2.30H)*, 2.21 (br s, 1H) 1.69 – 1.56 (m, 2H), 1.56 – 1.49 (m, 2H), 1.40 – 1.28 (m, 2H).

^{13}C NMR (101 MHz, $CDCl_3$) δ 142.7, 139.9, 128.8 (2C), 128.6 (2C), 128.5 (2C), 128.4 (2C), 126.3, 125.8, 51.2, 49.42 – 48.65 (m), 36.2, 35.9, 31.5, 29.5, 27.0.

HRMS (ESI$^+$): exact mass calculated for $[M+H]^+$ ($C_{19}H_{24}D_2N$) requires *m/z* 270.2185, found *m/z* 270.2187.

** The integral of 2.30 comprises the 2H benzylic indicated in "green" in the NMR spectrum, as well as 0.15 x 2H (resulting in an integral of 0.30H) of the non-deuterated α'-position of the product.*

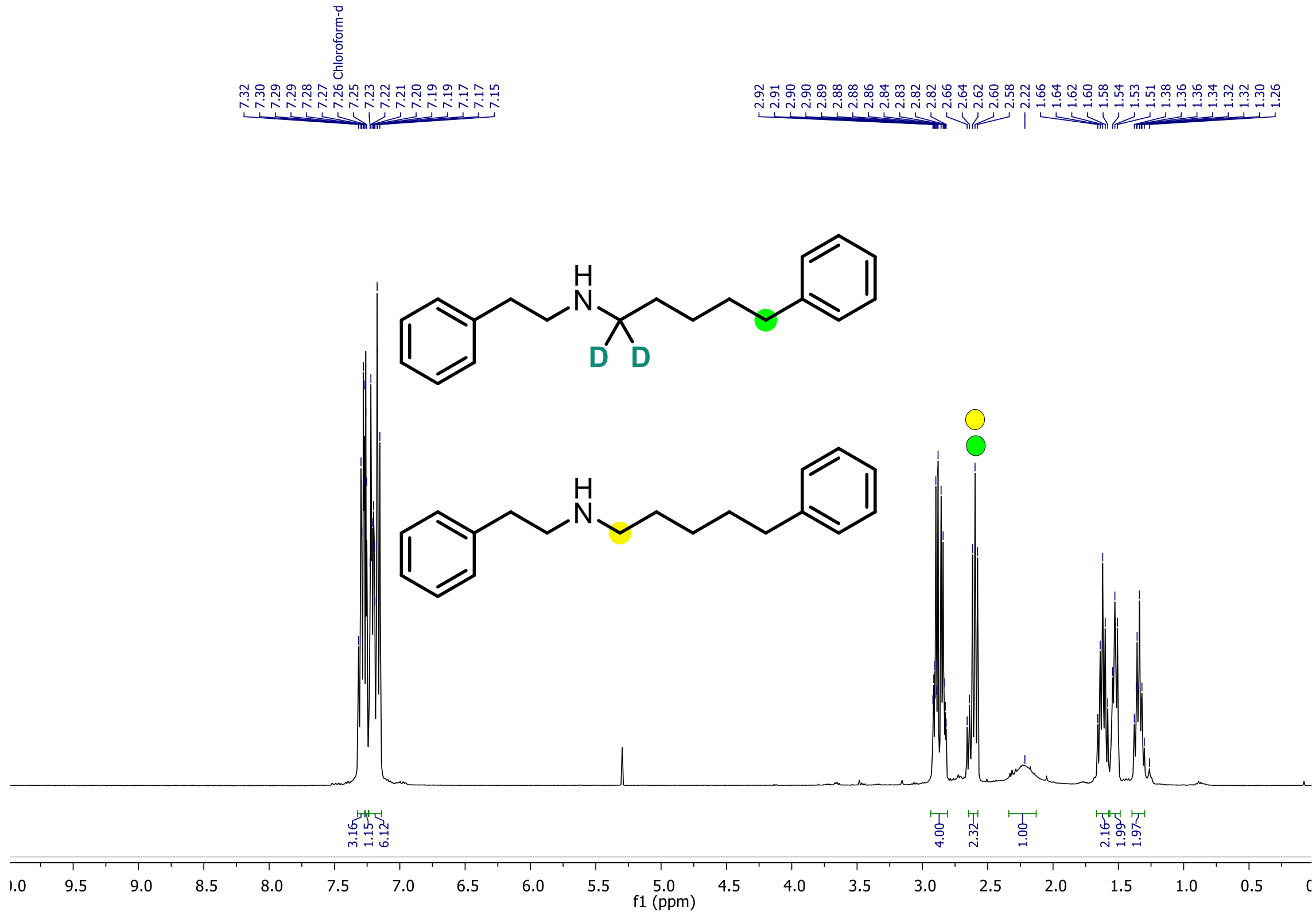
7.32
7.30
7.29
7.29
7.28
7.27
7.26 Chloroform-d
7.25
7.23
7.22
7.21
7.20
7.19
7.19
7.17
7.15
2.92
2.91
2.90
2.90
2.89
2.88
2.88
2.86
2.84
2.83
2.82
2.82
2.66
2.64
2.62
2.60
2.58
2.22
1.66
1.64
1.62
1.60
1.58
1.54
1.53
1.51
1.38
1.36
1.36
1.34
1.32
1.32
1.30
1.26
H
N
D
D
H
N
3.16
1.15
6.12
4.00
2.32
1.00
2.16
1.99
1.97
f1 (ppm)

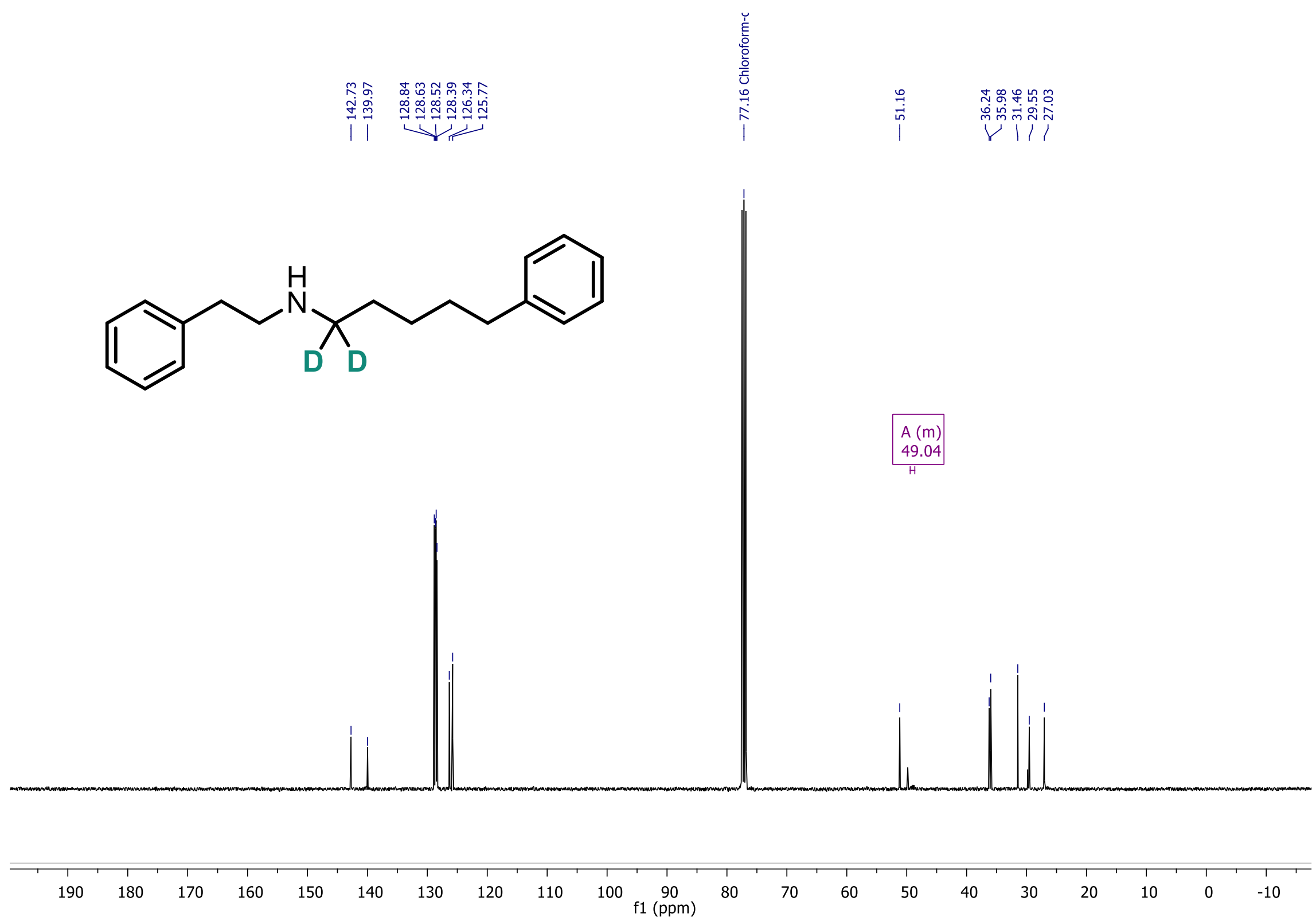
142.73
139.97
128.84
128.63
128.52
128.39
126.34
125.77
77.16 Chloroform-d
51.16
36.24
35.98
31.46
29.55
27.03
H
N
D
D
A (m)
49.04
f1 (ppm)

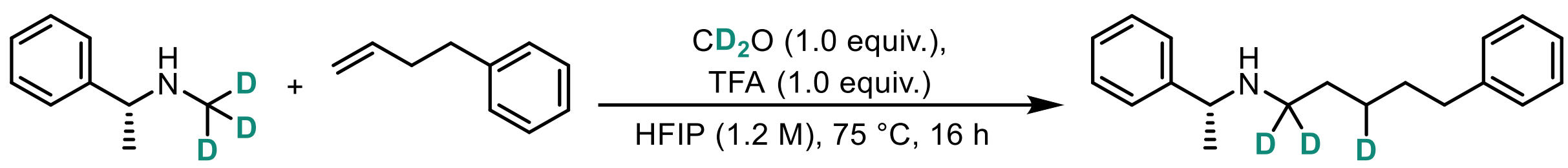

d.r 4:1
97% incorporation

Prepared following a slightly modified general procedure **4**, using (*R*)-*N*-(methyl-d3)-1-phenylethan-1-amine (43.6 µL, 0.30 mmol, 1.00 equiv.), 4-phenyl-1-butene (58.6 µL, 0.33 mmol, 1.10 equiv.), trifluoroacetic acid (22.3 µL, 0.30 mmol, 1.00 equiv.) and deuterated-paraformaldehyde (9.6 mg, 0.30 mmol, 1.0 equiv.).

Major isomer:

^{1}H NMR (600 MHz, $CDCl_3$) δ 7.35 – 7.27 (m, 5H), 7.24 – 7.16 (m, 5H), 3.60 – 3.51 (m, 1H), 3.06 (dd, *J* = 13.9, 4.7 Hz, 1H), 2.93 (dd, *J* = 13.7, 3.4 Hz, 1H), 2.62 (dd, *J* = 13.7, 9.1 Hz, 1H), 2.41 (dd, *J* = 13.9, 9.2 Hz, 1H), 1.91 (dt, *J* = 21.8, 10.9 Hz, 1H), 1.85 – 1.80 (m, 1H), 1.72 – 1.67 (m, 3H), 1.62 – 1.56 (m, 1H).

13C NMR (151 MHz, $CDCl_3$) δ 139.5, 137.9, 129.4 (2C), 128.9 (2C), 128.7 (2C), 128.5 (2C), 126.7, 126.2, 75.0, 45.7, 42.9, 35.1, 25.7, 29.1, 20.2.

#	m/z	I
1	123.0917	17292
2	129.0523	17417
3	130.0760	30297
4	131.0823	67434
5	132.0884	47809
6	134.1042	22226
7	157.0837	14685
8	159.1137	16795
9	160.1200	104319
10	161.1261	187367
11	162.1296	23477
12	167.1622	14754
13	179.1369	95019
14	183.0993	13955
15	185.1149	15492
16	197.0785	15340
17	197.1475	45034
18	216.1212	17826
19	219.1294	863559
20	220.1328	103219
21	235.1032	27147
22	237.1401	27074
23	270.2183	29187
24	271.2247	917242
25	272.2281	186047
26	273.2314	18345
27	314.1913	34003
28	365.1055	26008
29	415.2696	157563
30	416.2730	39661

#	m/z	I
1	271.2248	2000
2	272.2281	424
3	273.2314	43
4	274.2346	3

#	m/z	I
1	270.2185	2000
2	271.2218	424

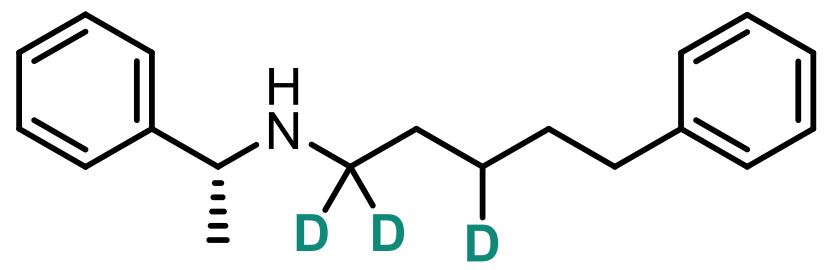

Exact Mass: 270.2175

[Exact Mass + H$^+$]: 271.2248

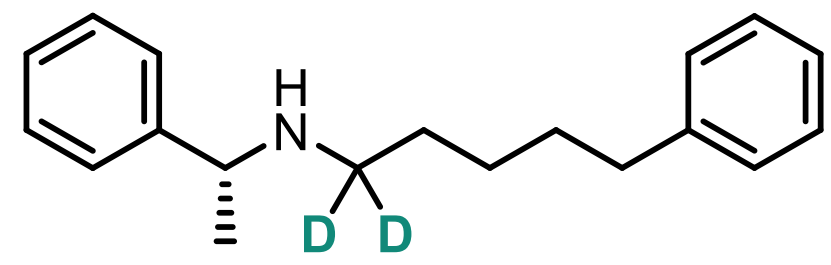

Exact Mass: 269.2113

[Exact Mass + H$^+$]: 270.2185

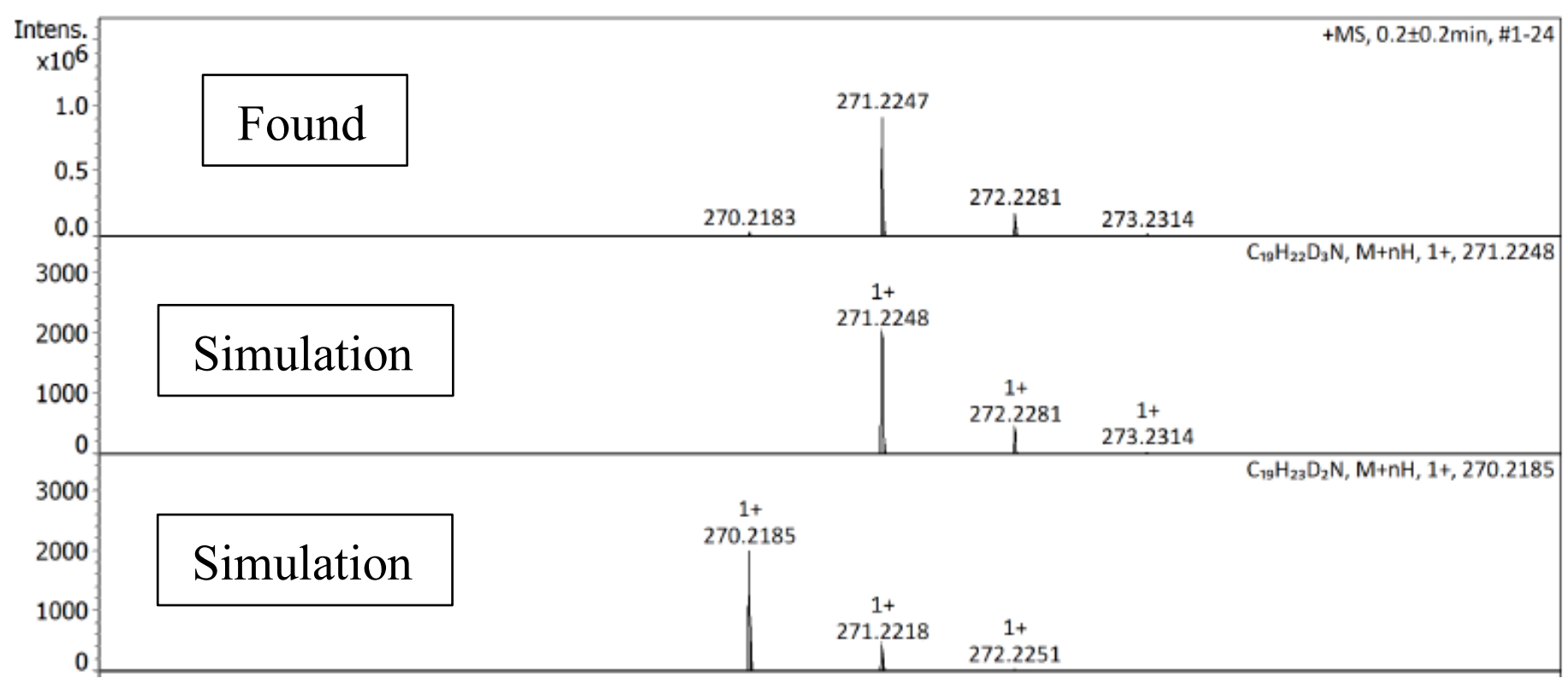

Mass Spectrum List Report

Analysis Info

Acquisition Date 22/07/2024 17:08:25

Analysis Name V:\Data\MSC service\106303000001.d
Method ESI_DI_standard_2023.m
Sample Name GIIA723
Comment Iannelli / Maulide
Ergebnis: +/- 5 ppm
ACN / MeOH + 1% H2O

Operator Demo User
Instrument timsTOF flex 1859745.00301

Acquisition Parameter

Source Type	ESI	Ion Polarity	Positive	Set Nebulizer	0.3 Bar
Focus	Not active	Set Capillary	3500 V	Set Dry Heater	200 Â°C
Scan Begin	75 m/z	Set End Plate Offset	-500 V	Set Dry Gas	3.0 l/min
Scan End	1600 m/z	Set Collision Cell RF	500.0 Vpp	Set Divert Valve	Source

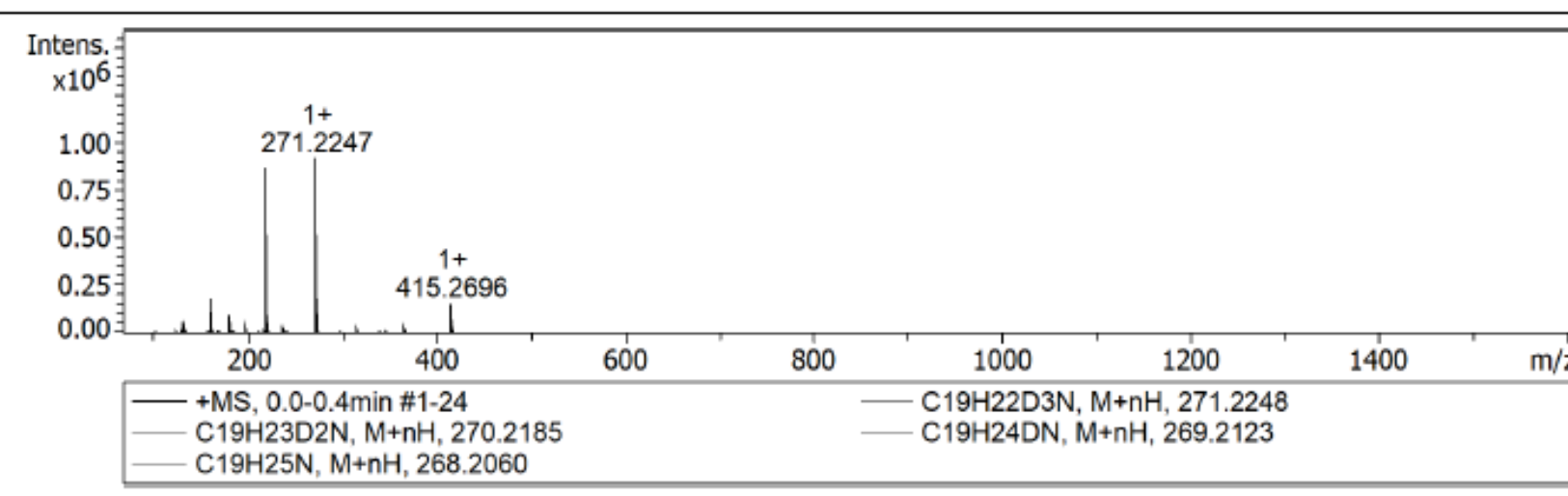

Supplementary Fig. S7. HRMS trace of the crude mixture.

6.2 NMR analysis

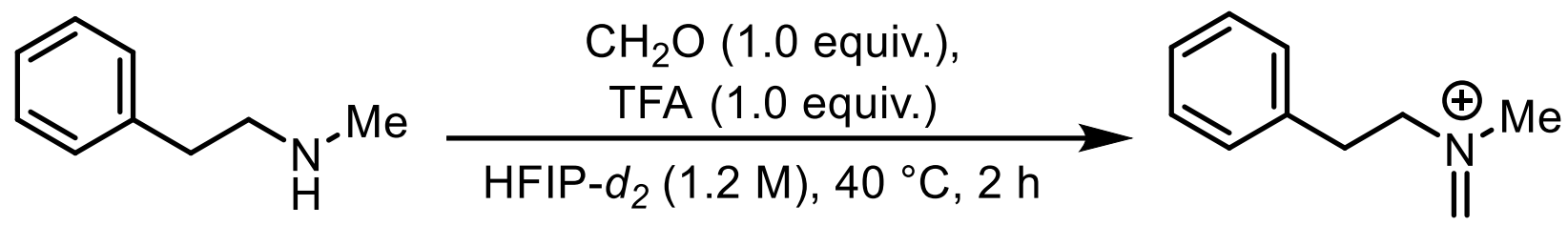

To an oven dried vial *N*-methyl-phenethylamine (43.6 µL, 0.30 mmol, 1.00 equiv.), paraformaldehyde (9.0 mg, 0.30 mmol, 1.0 equiv.) and deuterated hexafluoroisopropanol (1.2 M) were added at room temperature. The suspension was cooled to 0 °C and trifluoroacetic acid (22.3 µL, 0.30 mmol, 1.00 equiv.) was added dropwise. The resulting solution was heated at 40 °C for 2 h. Then, an aliquot was taken and used directly for NMR experiments.[24]

^{1}H NMR (700 MHz, HFIP-*d₂*) δ 7.56 (d, *J* = 7.1 Hz, 1H), 7.46 (dd, *J* = 11.0, 3.9 Hz, 2H), 7.43 – 7.38 S(m, 1H), 7.18 (d, *J* = 7.9 Hz, 2H), 7.13 (d, *J* = 7.0 Hz, 1H), 4.19 (t, *J* = 6.5 Hz, 2H), 3.73 (s, 3H), 3.19 (t, *J* = 5.9 Hz, 2H).

^{13}C NMR (176 MHz, HFIP-*d₂*) δ 165.3 – 165.1 (m), 132.3, 128.7 (2C), 127.6, 127.0 (2C), 63.3, 45.8, 31.8.

^{1}H NMR (700 MHz, HFIP-d_2)

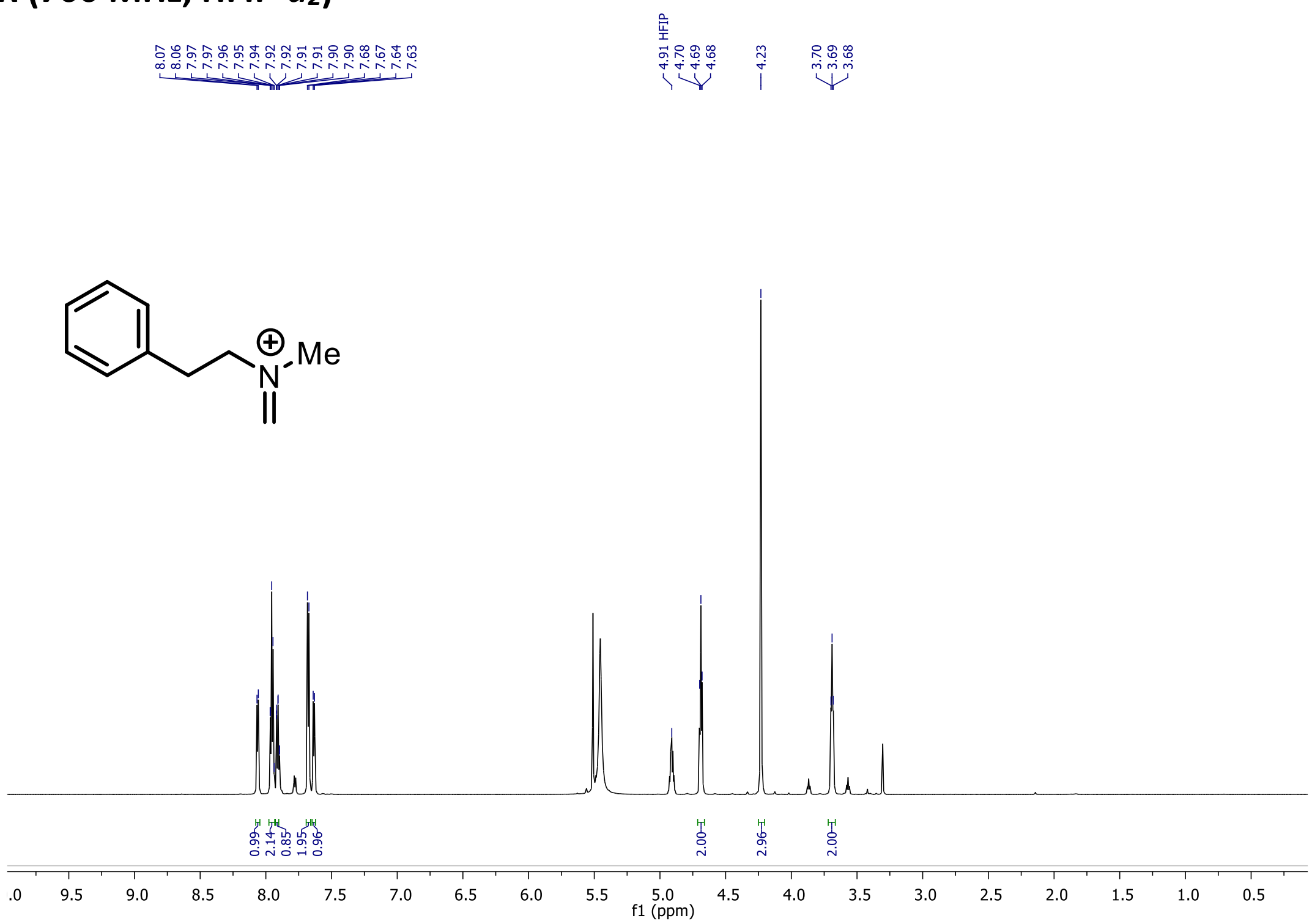

HSQC (^{1}H NMR (700 MHz, HFIP-d_2) ^{13}C NMR (176 MHz, HFIP-d_2))

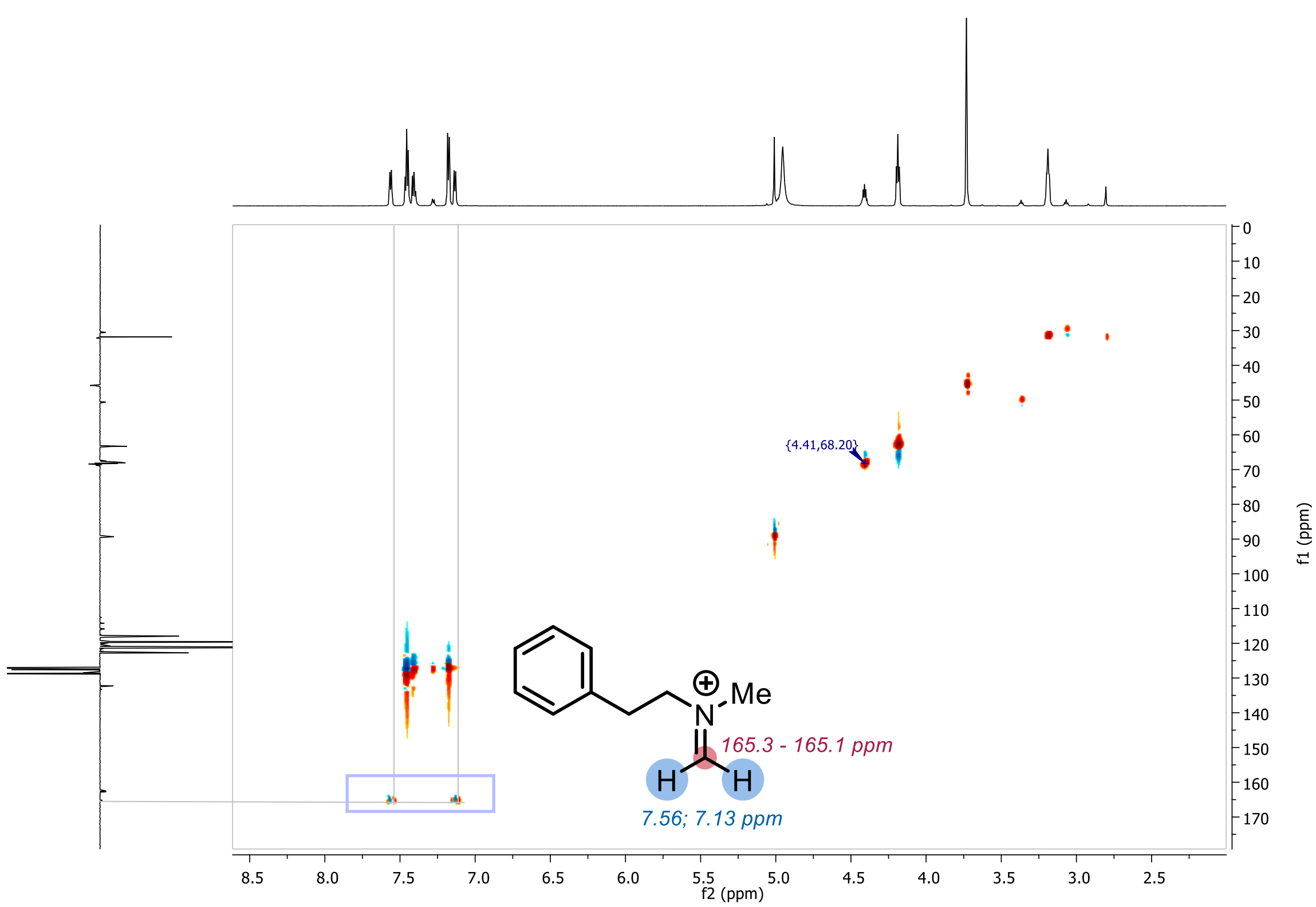

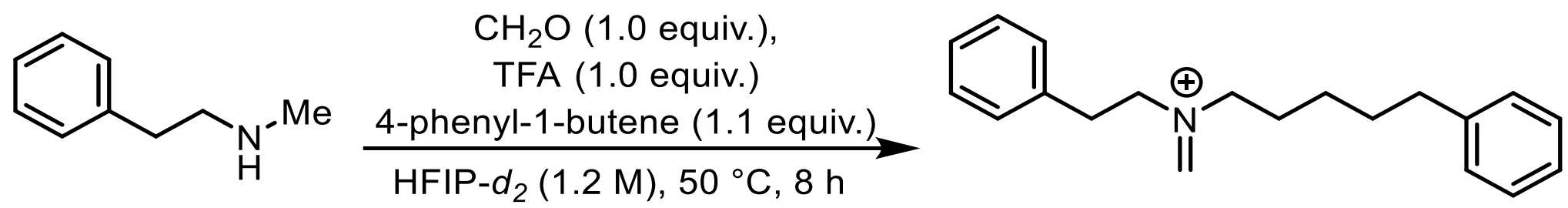

To an oven dried vial *N*-methyl-phenethylamine (43.6 µL, 0.30 mmol, 1.00 equiv.), paraformaldehyde (9.0 mg, 0.30 mmol, 1.0 equiv.) and deuterated hexafluoroisopropanol (1.2 M) were added at room temperature. The suspension was cooled to 0 °C and trifluoroacetic acid (22.3 µL, 0.30 mmol, 1.00 equiv.) was added dropwise. The resulting solution was heated at 40 °C for 2 h, allowed to cool to room temperature and then 4-phenyl-1-butene (58.6 µL, 0.33 mmol, 1.10 equiv.) was added to the reaction mixture. An aliquot was immediately taken, and the reaction was monitored by ^{1}H NMR at 50 °C for 8 h.

Note = Because of the solvent's boiling point, we were unable to conduct the monitoring at the standard temperature of 75 °C, resulting in only 40% conversion. The relative ratio of detected species after 8 h at 50 °C is reported below:

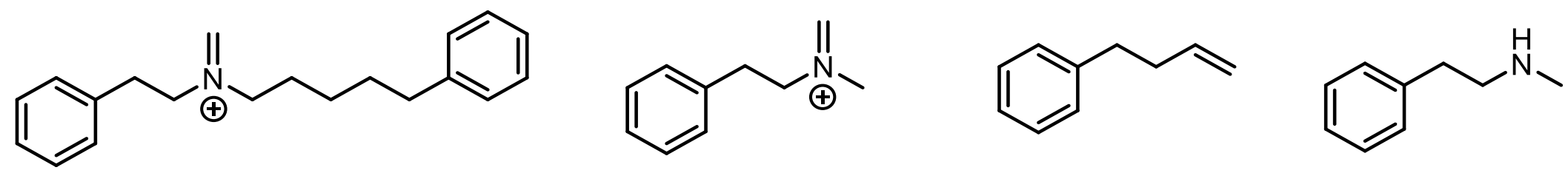

In ratio *1* *4.5* *7.5* *1.15*

^{1}H NMR (700 MHz, HFIP-*d*₂)

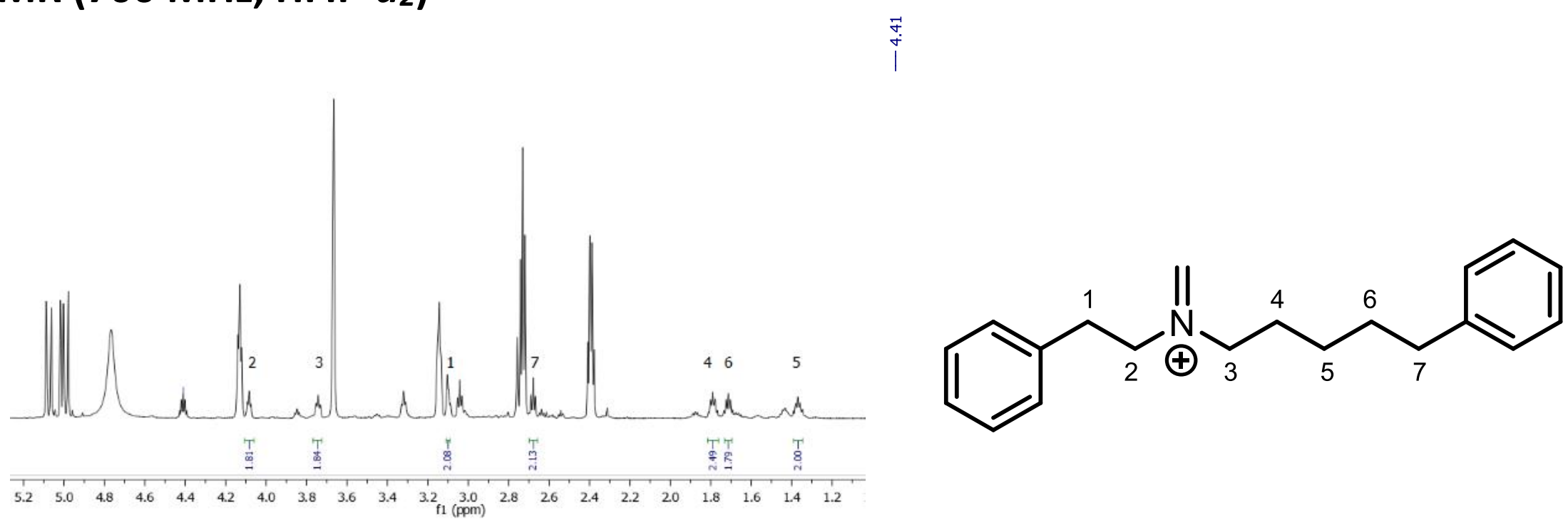

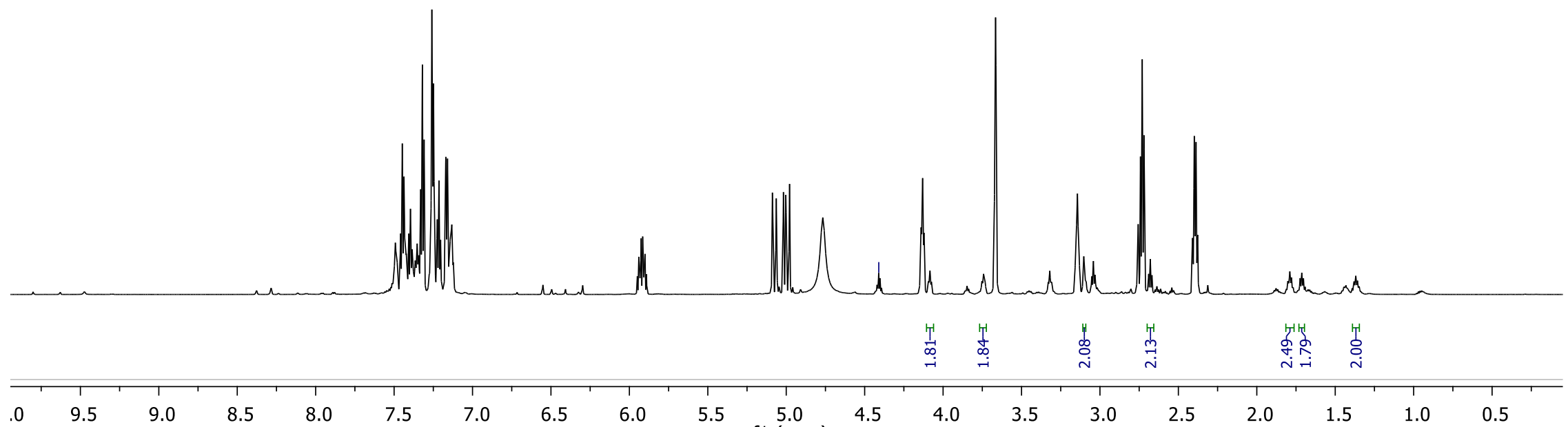

HSQC (^{1}H NMR (700 MHz, HFIP-*d*₂) ^{13}C NMR (176 MHz, HFIP-*d*₂))

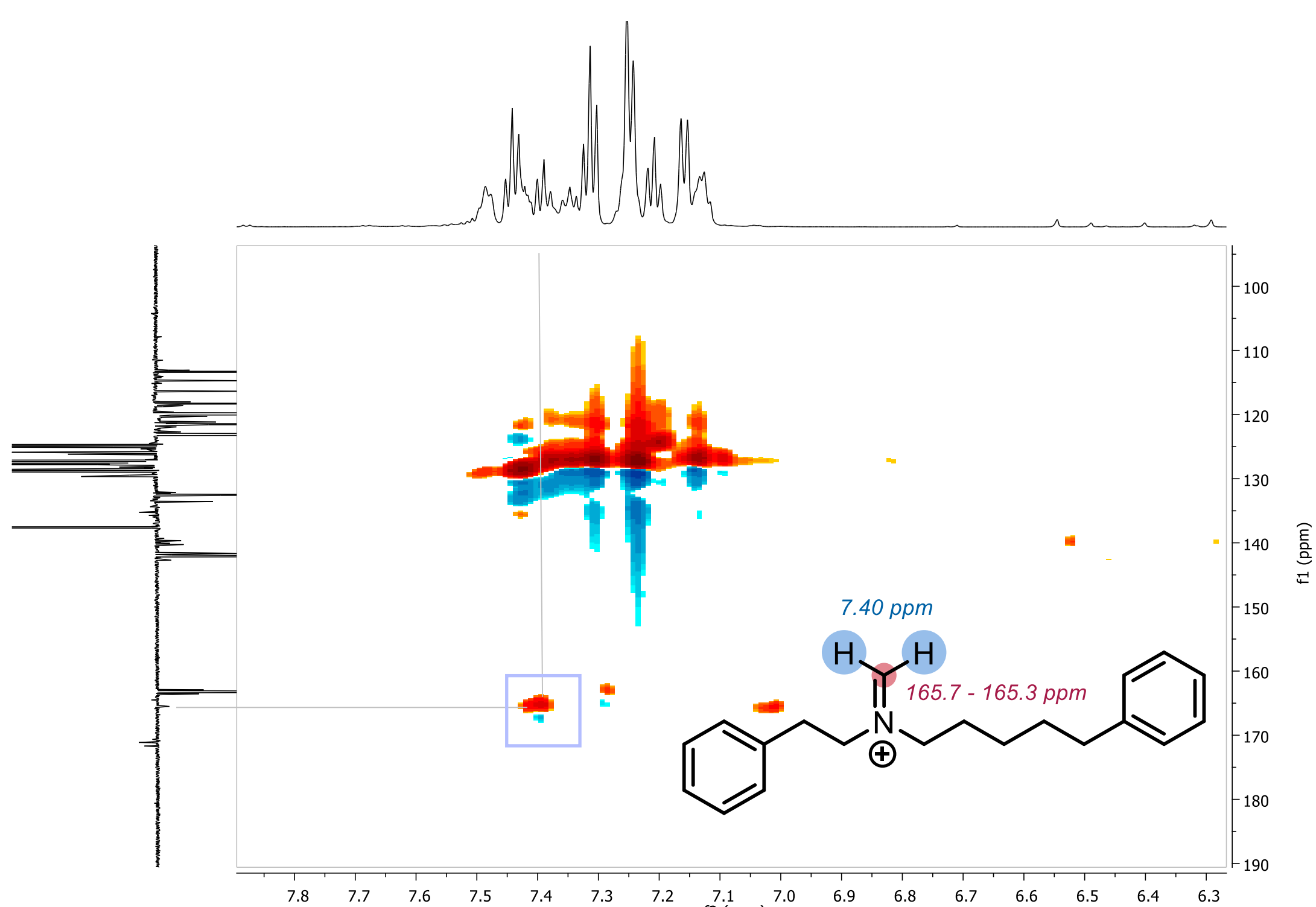

HMBC (^{1}H NMR (700 MHz, HFIP-d_2) ^{13}C NMR (176 MHz, HFIP-d_2))

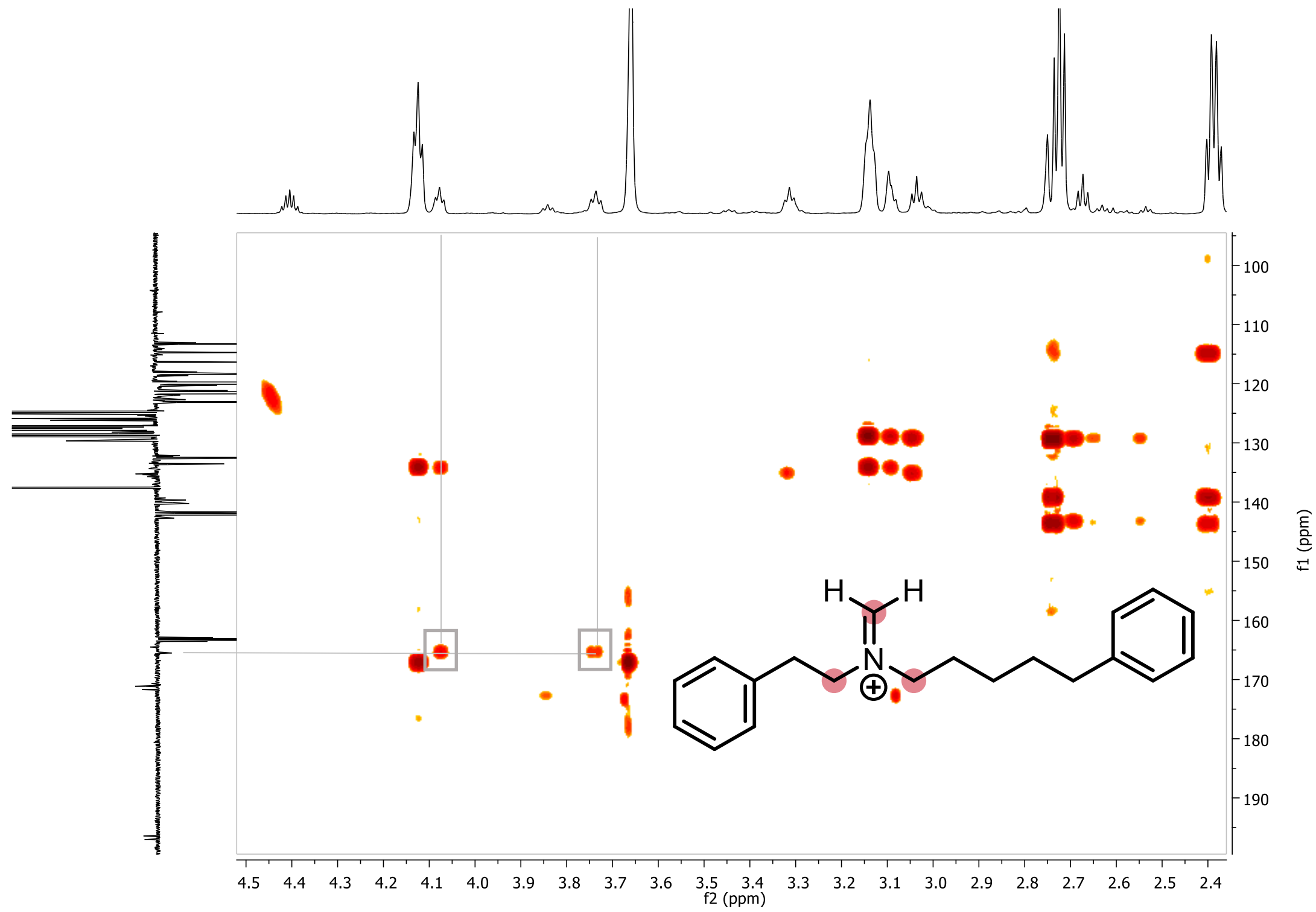

6.3 Formaldehyde recycling

Additionally, we performed a reaction in which a new combination of alkene and amine was added directly to the crude reaction mixture without adding further paraformaldehyde (**Supplementary Fig. S8**). The mixture was then subjected to the standard reaction conditions, and the formation of both desired products was observed.

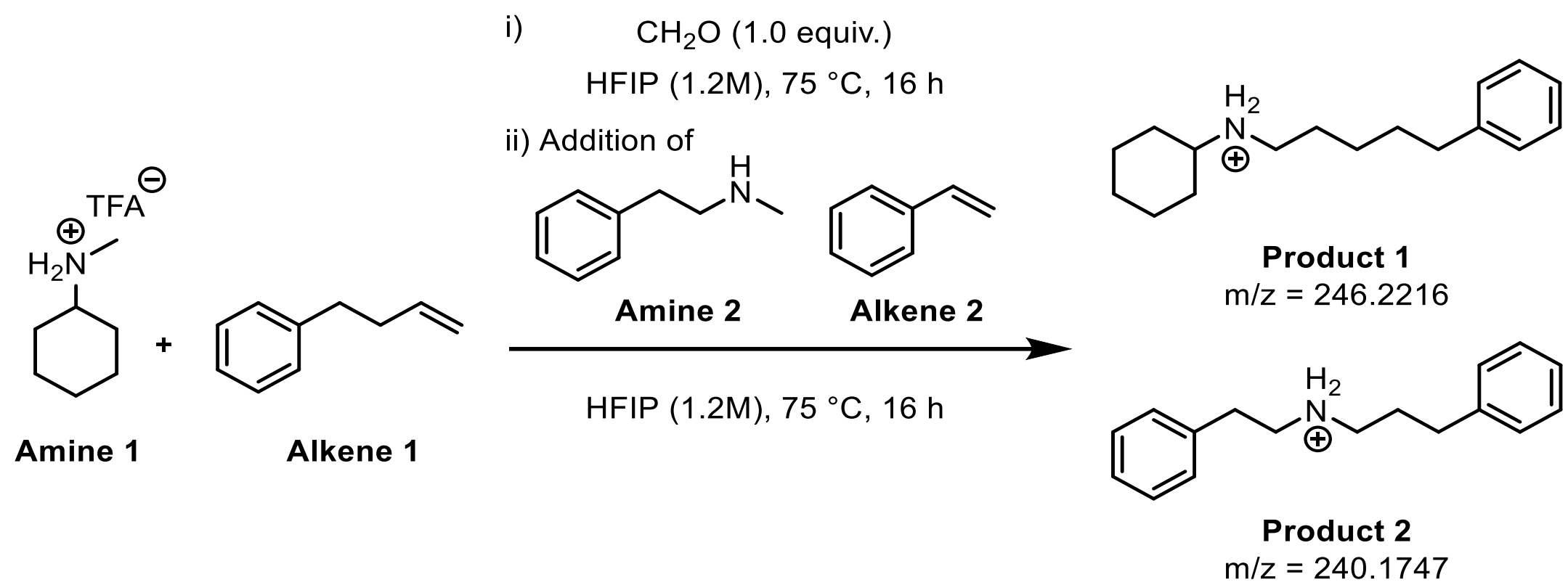

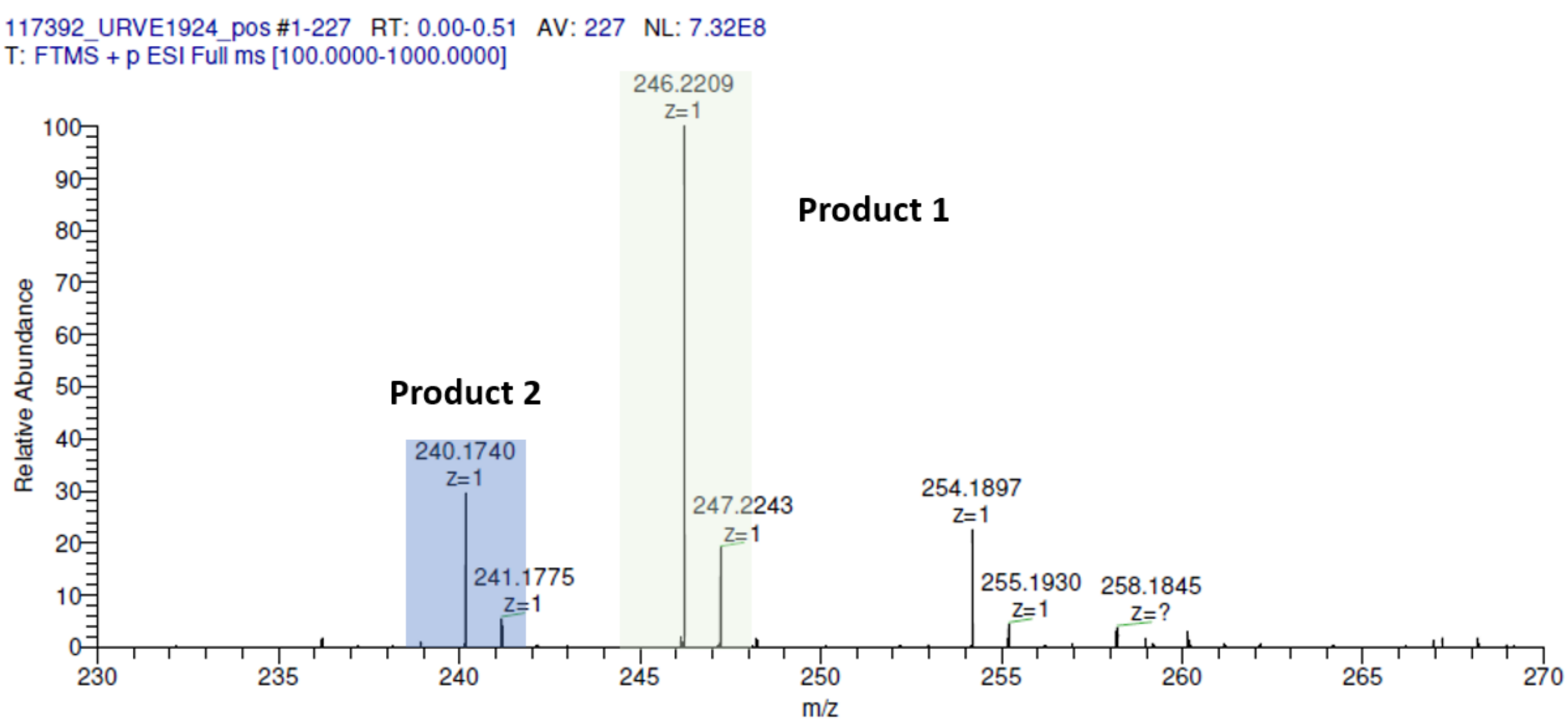

Supplementary Fig. S8. **Additional experiment supporting formaldehyde recovery.**

7. Comparison with the state-of-the-art approach

To demonstrate the significance of our transformation in functionalizing native amines, we compared it with the state-of-the-art approach for α-alkylation of amines developed by the MacMillan group.[25]

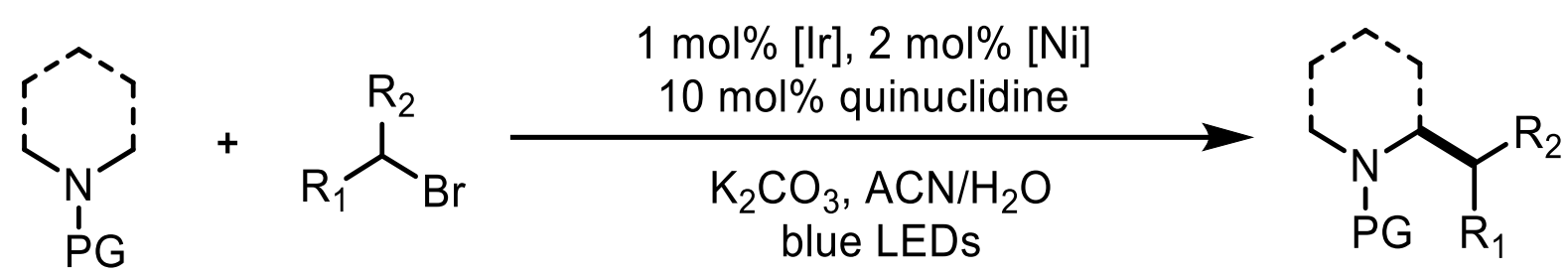

Supplementary Fig. S9. State-of-the-art method for peripheral modification.

To establish the reproducibility of their protocol in our laboratory, we repeated a literature-reported example and successfully prepared compound **C1**, see below.

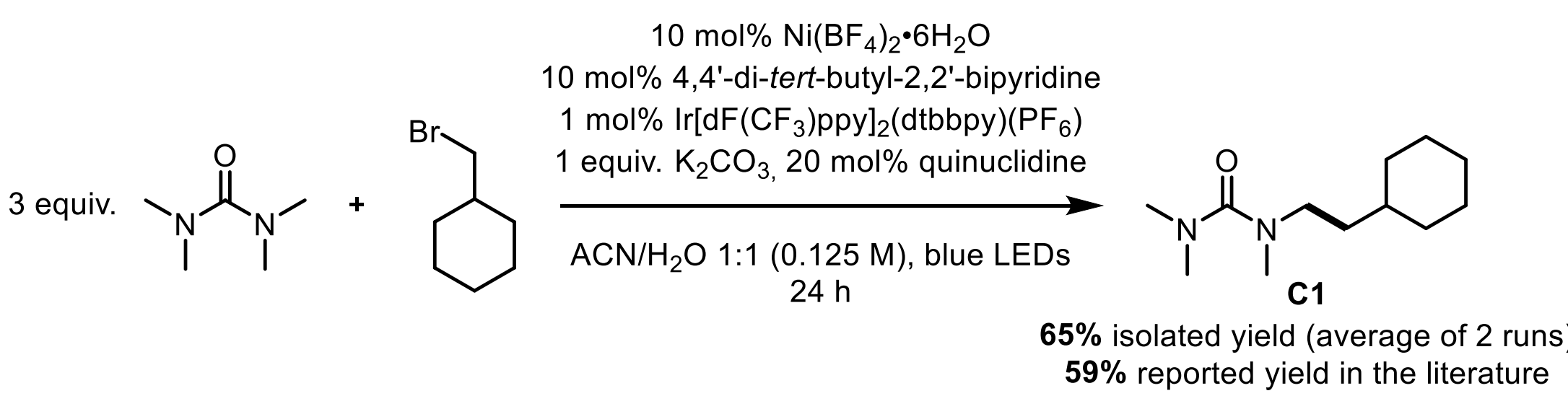

Supplementary Fig. S10. Literature-reported reaction reproduced in our laboratory to confirm reproducibility.

Having established the general reproducibility of the reaction in our laboratory, we subjected unprotected Fluoxetine to the reported conditions (the authors had previously reported a successful reaction with Boc-protected Fluoxetine). As shown below, the desired product was not detected under these conditions and both starting materials were recovered in substantial quantities.

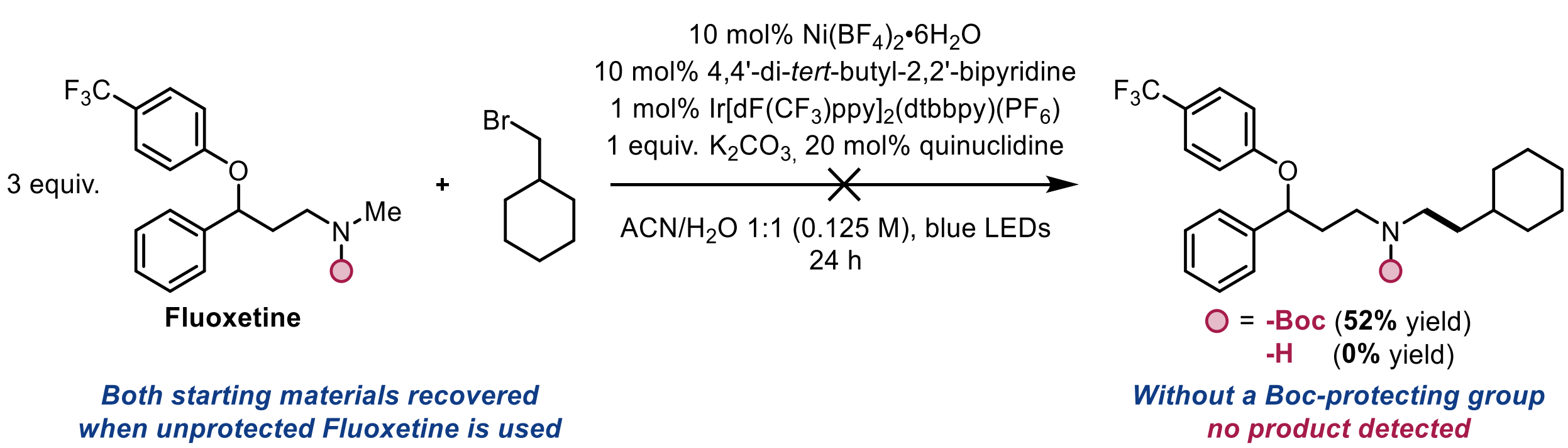

Supplementary Fig. S11. Unsuccessful reaction under literature conditions when applied to the native, unprotected amine.

Hence, it appears that the functionalization of native, unprotected amines is indeed a very challenging endeavor.

1-(2-Cyclohexylethyl)-1,3,3-trimethylurea (C1)

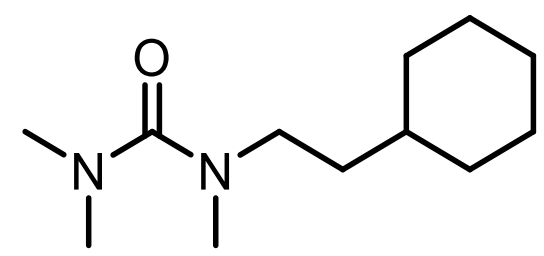

$C_{12}H_{24}N_2O$
MW: 212.3370

^{1}H NMR (400 MHz, $CDCl_3$): δ 3.15 – 3.10 (m, 2H), 2.75 (s, 6H), 2.74 (s, 3H), 1.72 – 1.57 (m, 5H), 1.45 – 1.37 (m, 2H), 1.26 – 1.05 (m, 4H), 0.96 – 0.80 (m, 2H).

The spectroscopic properties are consistent with the data available in the literature.[25]

8. References

1. Lebleu, T., Ma, X., Maddaluno, J. & Legros, J. Selective monomethylation of primary amines with simple electrophiles. *ChemComm* **50**, 1836–1838 (2013).
2. Porte, V. *et al.* Chemical and redox noninnocence of pentane-2,4-dione Bis(S-methylisothiosemicarbazone) in cobalt complexes and their application in Wacker-Type oxidation. *JACS Au* **4**, 1166–1183 (2024).
3. Chen, Y., Ma, Y., Li, L., Jiang, H. & Li, Z. Nitration–Peroxidation of Alkenes: A Selective Approach to β-Peroxyl Nitroalkanes. *Org. Lett.* **21**, 1480–1483 (2019).
4. Kaiser, D. *et al.* A General Acid-Mediated Hydroaminomethylation of Unactivated Alkenes and Alkynes. *Angew. Chem. Int. Ed.* **58**, 14639–14643 (2019).
5. Hsu, C. *et al.* Leveraging Electron-Deficient iminium intermediates in a general synthesis of valuable amines. *Angew. Chem. Int. Ed.* **61**, e202115435 (2022).
6. Porte, V., Di Mauro, G., Schupp, M. Kaiser, D. & Maulide, N. Chemoselective Alpha-Deuteration of amides via retro-ene reaction. *Chem. Eur. J.* **26**, 15509–15512 (2020).
7. Jin, R.-X., Dai, J.-C., Li, Y. & Wang, X.-S. Copper-Catalyzed Intramolecular Amination of C(sp^3)–H Bond of Secondary Amines to Access Azacycles. *Org. Lett.* **23**, 421–426 (2021).
8. Zhang, Y., Li, S., Chen, H., Song, W., Ning, Y., Liu, Z., Wu, Y., Murali, K., Sivaguru, P., & Zhang, X. Dimethoxyacetaldehyde-N-triftosylhydrazone: preparation and Carbene reactivity in cyclopropanation and Doyle–Kirmse reactions. *Org. Lett.*, **27**, 1941–1948 (2025).
9. Zamboni, R. *et al.* Development of a novel series of styrylquinoline compounds as high-affinity leukotriene D4 receptor antagonists: synthetic and structure-activity studies leading to the discovery of (.+-.)-3-[[[3-[2-(7-chloro-2-quinolinyl)-(E)-ethenyl]phenyl][[3-(dimethylamino)-3-oxopropyl]thio]methyl]thio]propionic acid. *J. Med. Chem.* **35**, 3832–3844 (1992).
10. Schleicher, K. D., & Jamison, T. F. Nickel-Catalyzed Synthesis of Acrylamides from α-Olefins and Isocyanates. *Org. Lett.*, **9**, 875–878 (2007).
11. Fabre, B. *et al.* Progress towards water-soluble triazole-based selective MMP-2 inhibitors. *Org. Biomol. Chem.* **11**, 6623 (2013).
12. Bolchi, C., Pallavicini, M., Binda, M., Fumagalli, L. & Valoti, E. From carnitinamide to 5-aminomethyl-2-oxazolidinones. *Tetrahedron Asymmetry.* **3**, 217–220 (2012).

13. Hansen, E. B., Cho, B. P., Korfmacher, W. A. & Cerniglia, C. A. Fungal transformations of antihistamines: metabolism of brompheniramine, chlorpheniramine, and pheniramine to N-oxide and N-demethylated metabolites by the fungus Cunninghamella elegans. *Xenobiotica* **25**, 1081–1092 (1995).
14. Jin, C., Boldt, K. G., Rehder, K. S. & Brine, G. A. Improved syntheses of N-Desmethylcitalopram and N,N-Didesmethylcitalopram. *Synth. Commun.* **37**, 901–908 (2007).
15. Spieß, P. *et al*. Nms-amides: An Amine protecting group with unique stability and selectivity. *Chem. Eur. J.* **29**, e202301312 (2023).
16. Luo, T. *et al.* STK33 kinase inhibitor BRD-8899 has no effect on KRAS-dependent cancer cell viability. *Proc. Natl. Acad. Sci. USA* **109**, 2860–2865 (2012).
17. Yu, H. *et al.* Conformational control of a Metallo-Supramolecular cage via the dissymmetrical modulation of ligands. *Angew. Chem. Int. Ed.* **60**, 26523–26527 (2021).
18. Wu, Z. et al. Iridium-catalyzed direct asymmetric reductive amination utilizing primary alkyl amines as the N-sources. *Nat. Commun.* **13**, 3344 (2022).
19. Manickam, M. *et al*. Exploration of flexible phenylpropylurea scaffold as novel cardiac myosin activators for the treatment of systolic heart failure. *Eur. J. Med. Chem.* **134**, 379–391 (2017).
20. Morimoto, Y., Kochi, T., & Kakiuchi, F. Rhodium-Catalyzed Anti-Markovnikov Hydroamination of Aliphatic and Aromatic Terminal Alkynes with Aliphatic Primary Amines. *J. Org. Chem.*, **86**, 13143–13152 (2021).
21. Karageorge, G. N. & Macor, J. E. Synthesis of novel serotonergics and other N-alkylamines using simple reductive amination using catalytic hydrogenation with Pd/C. *Tetrahedron Lett.* **52**, 5117–5119 (2011).
22. Simmons, B. J., Hoffmann, M., Hwang, J., Jackl, M. K. & Garg, N. K. Nickel-Catalyzed reduction of secondary and tertiary amides. *Org. Lett.* **19,** 1910–1913 (2017).
23. Zheng, J., Roisnel, T., Darcel, C. & Sortais, J. Nickel-Catalysed Reductive Amination with Hydrosilanes. *ChemCatChem* **5**, 2861–2864 (2013).
24. Lemmerer, M. *et al.* HFIP Mediates a Direct C−C Coupling between Michael Acceptors and Eschenmoser's salt. *Angew. Chem. Int. Ed.* **61**, e202109933 (2021).

25. Le, C., Liang, Y., Evans, R. W., Li, X. & MacMillan, D. W. C. Selective sp^3 C–H alkylation via polarity-match-based cross-coupling. *Nature* **547**, 79–83 (2017).

9. NMR spectra

3-(4-Chlorophenyl)-*N*-methyl-3-(pyridin-2-yl)propan-1-amine (S1.09a)
^{1}H NMR (400 MHz, $CDCl_3$)

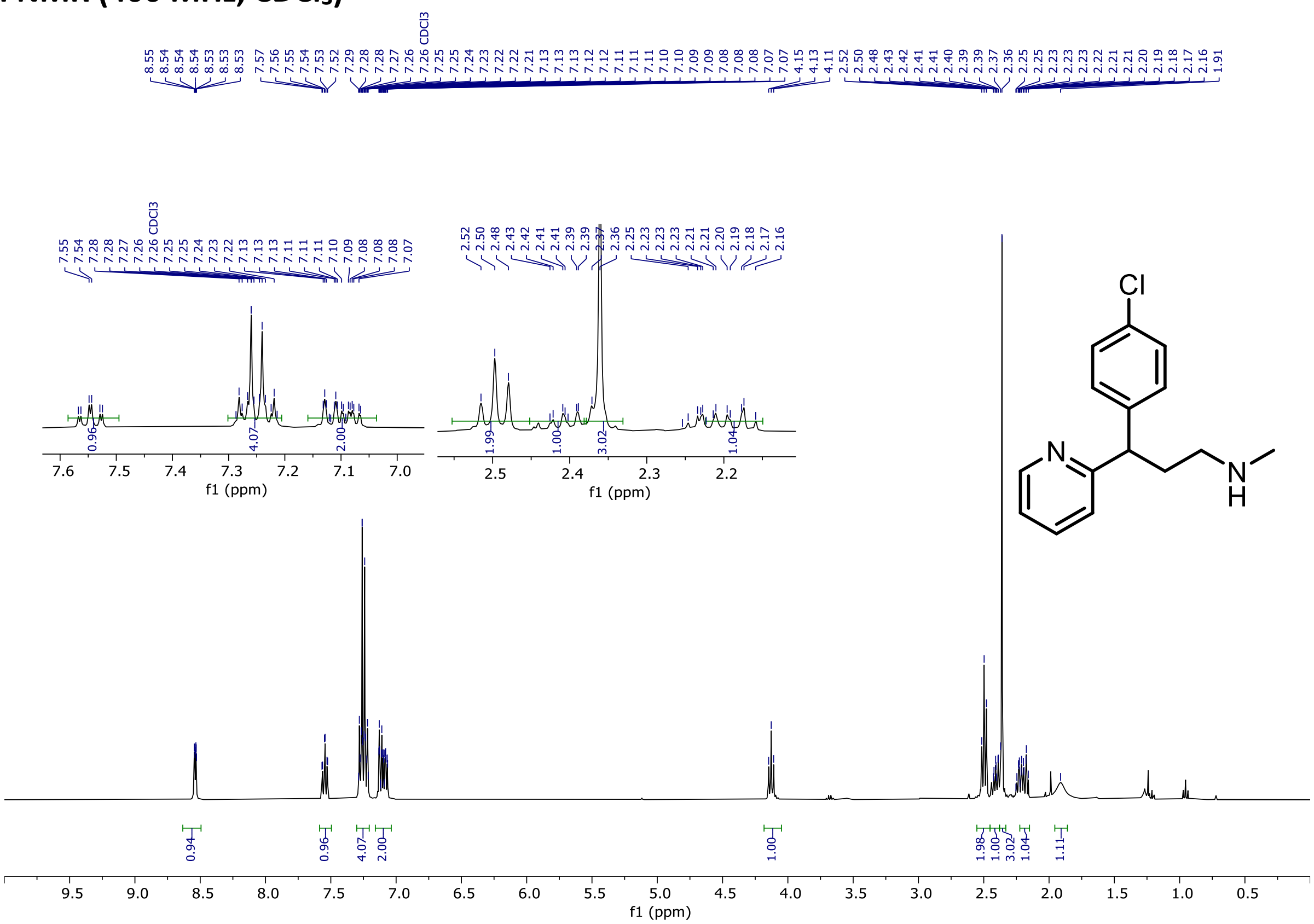

1-(4-Fluorophenyl)-1-(3-(methylamino)propyl)-1,3-dihydroisobenzofuran-5-carbonitrile (S1.10a)

^{1}H NMR (400 MHz, $CDCl_3$)

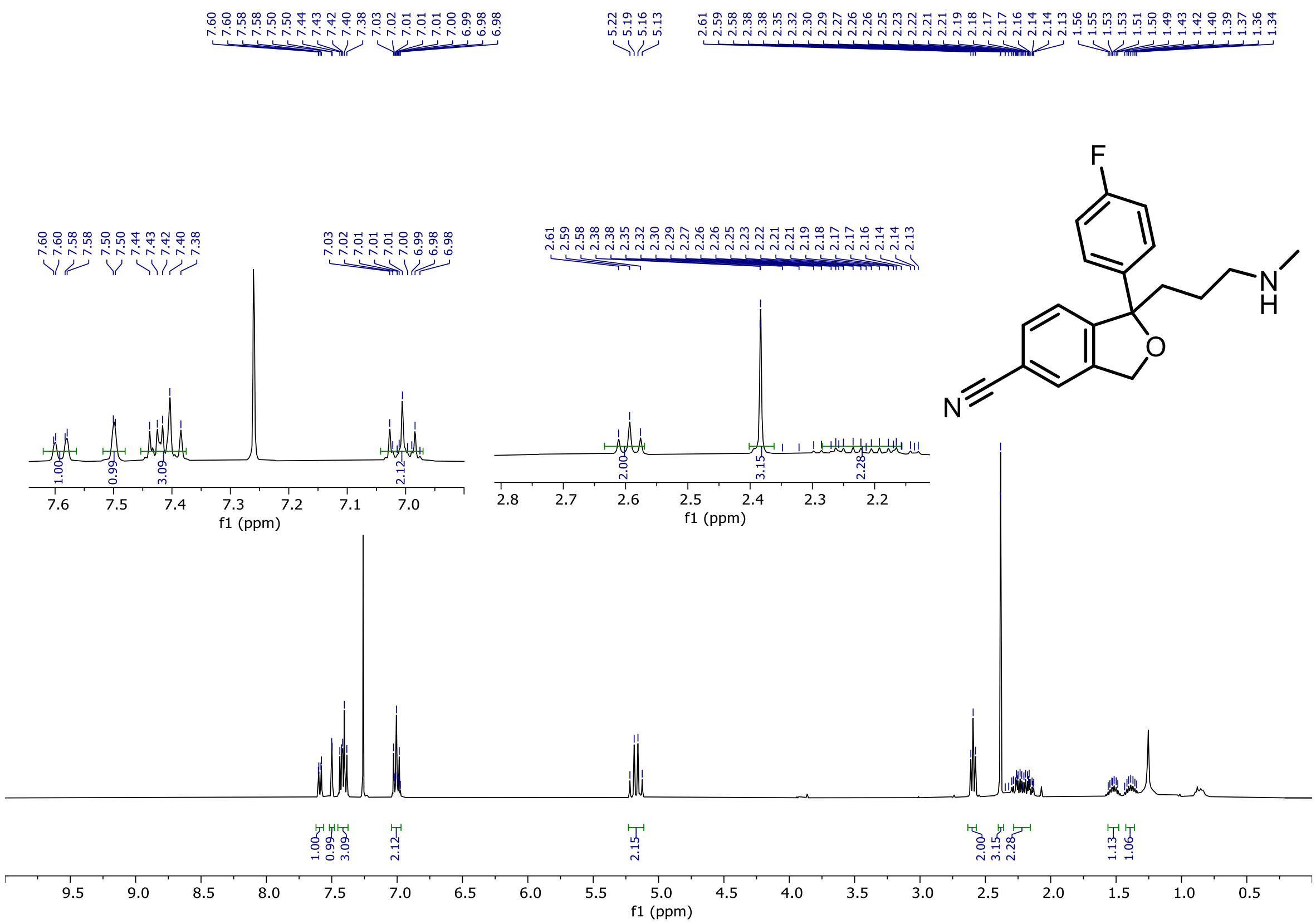

3-((1-Benzyl-1*H*-indazol-3-yl)oxy)-*N*-methylpropan-1-amine (S1.11a)

^{1}H NMR (400 MHz, $CDCl_3$)

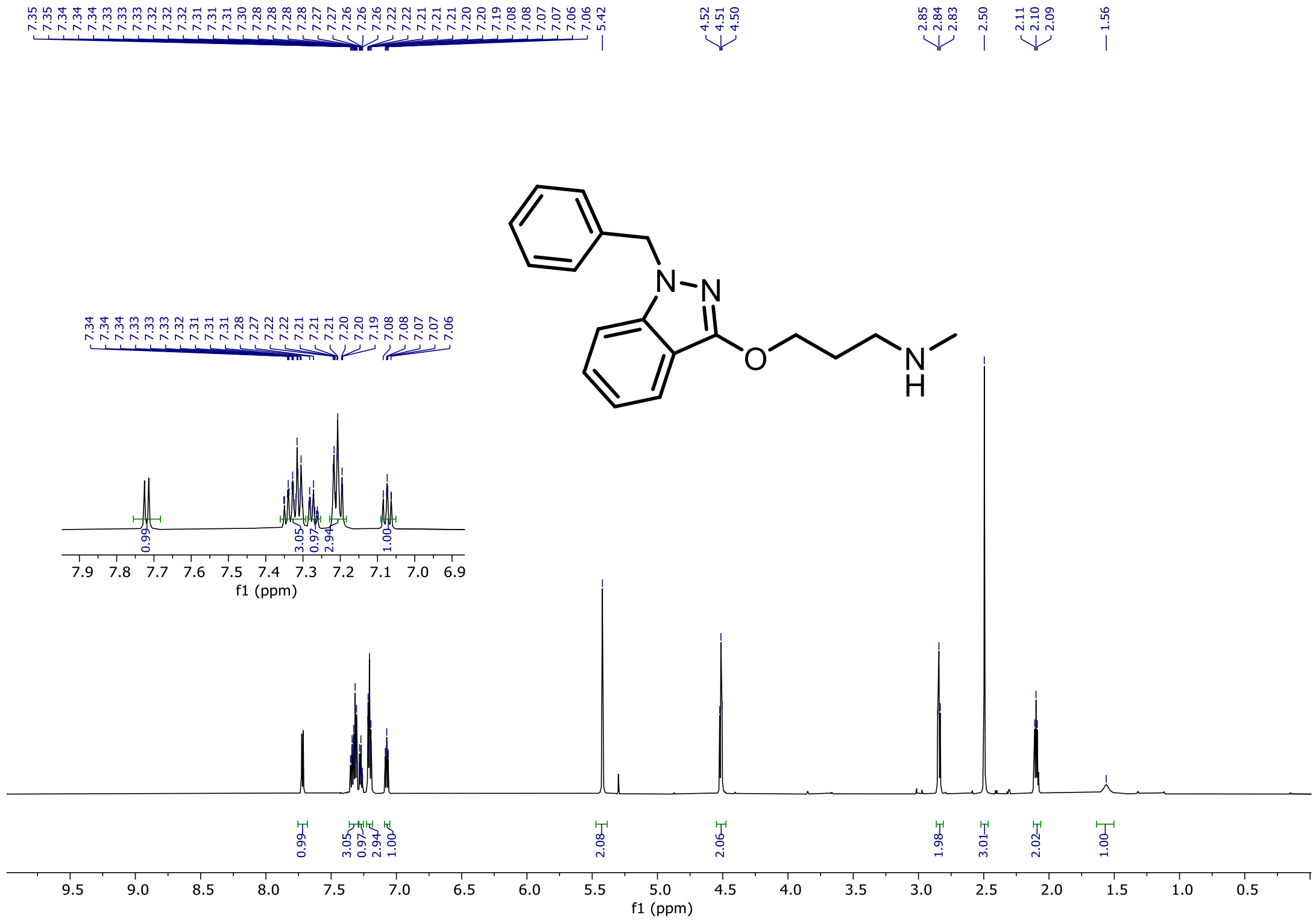

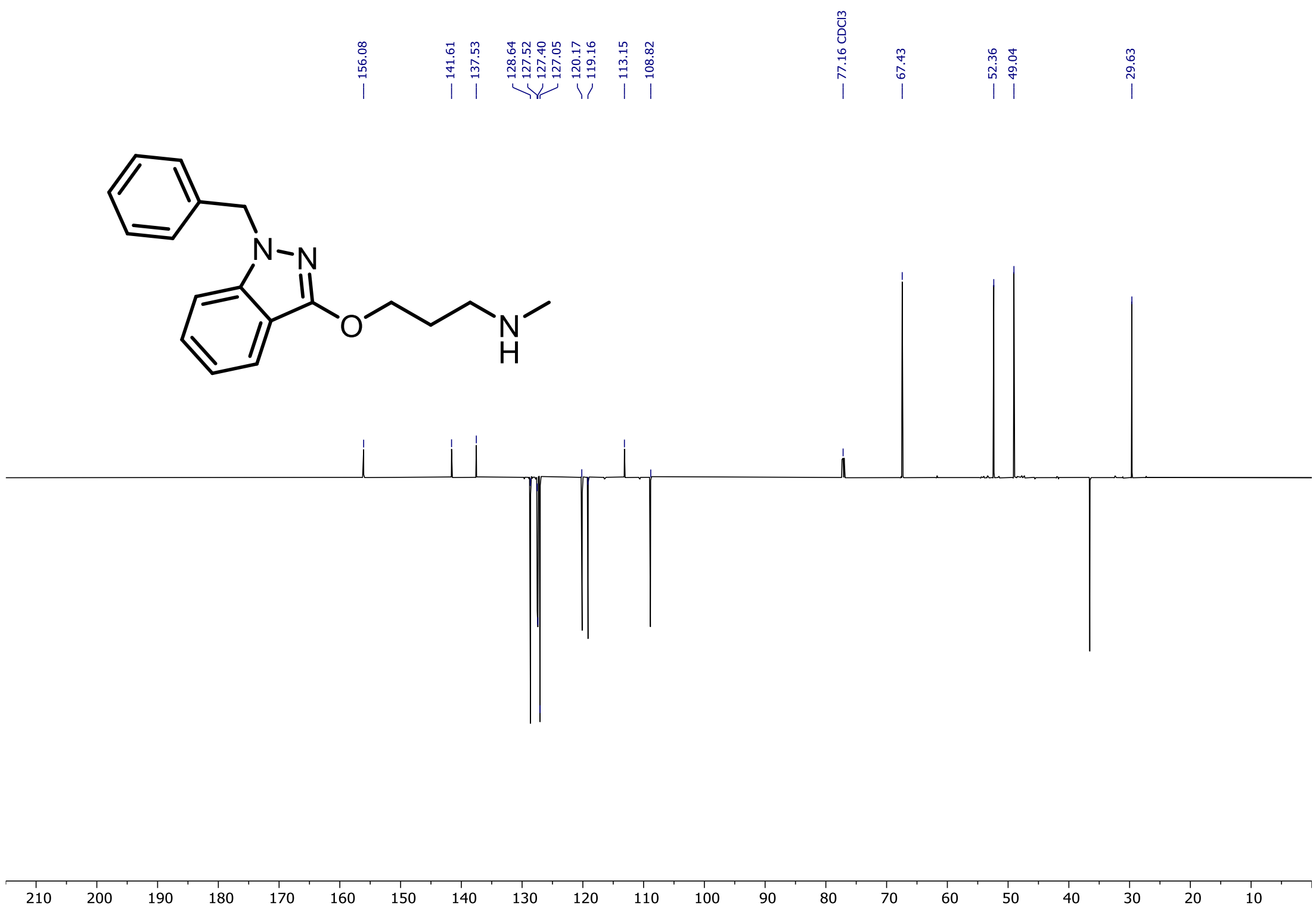

2-(1-(4-Chlorobenzoyl)-5-methoxy-2-methyl-1*H*-indol-3-yl)-*N*-methyl-*N*-(undec-10-en-1-yl)acetamide (S1.16b)

^{1}H NMR (600 MHz, $CDCl_3$)

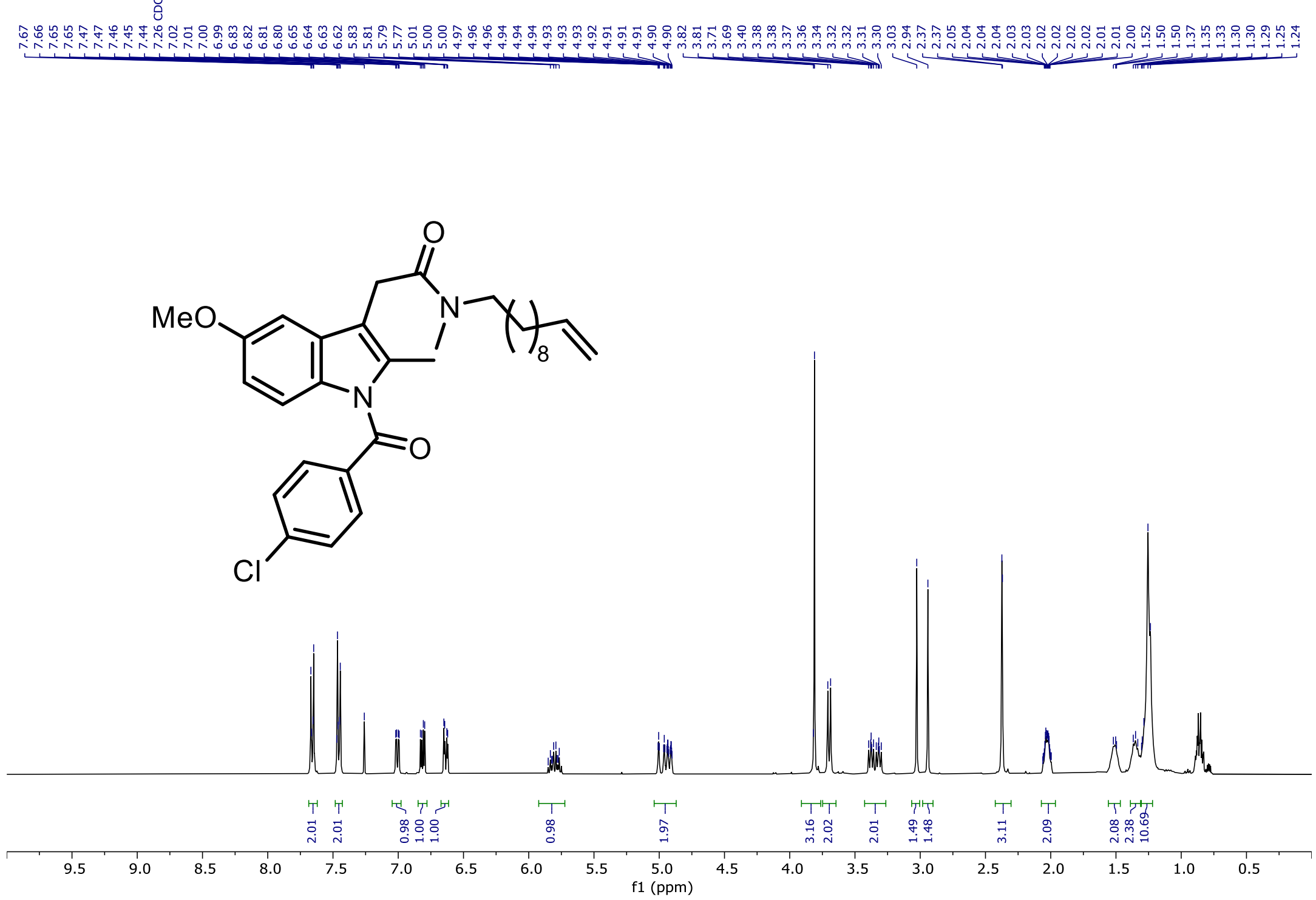

^{13}C NMR (151 MHz, $CDCl_3$)

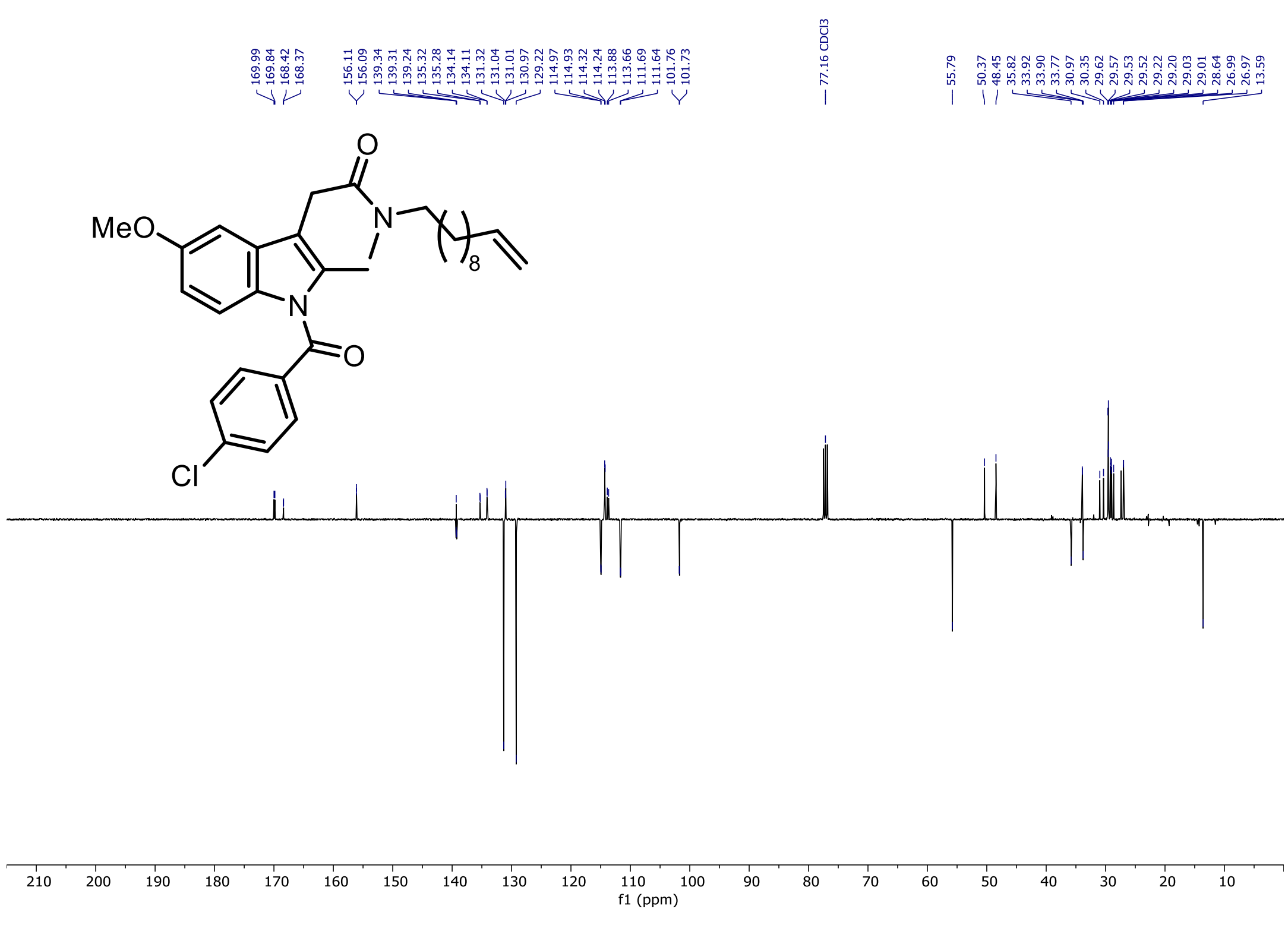

(*S*)-2-(4-(4-Chlorophenyl)-2,3,9-trimethyl-6*H*-thieno[3,2-f][1,2,4]triazolo[4,3-a][1,4]diazepin-6-yl)-*N*-methyl-*N*-(undec-10-en-1-yl)acetamide (S1.19b)

^{1}H NMR (600 MHz, CDCl$_3$)

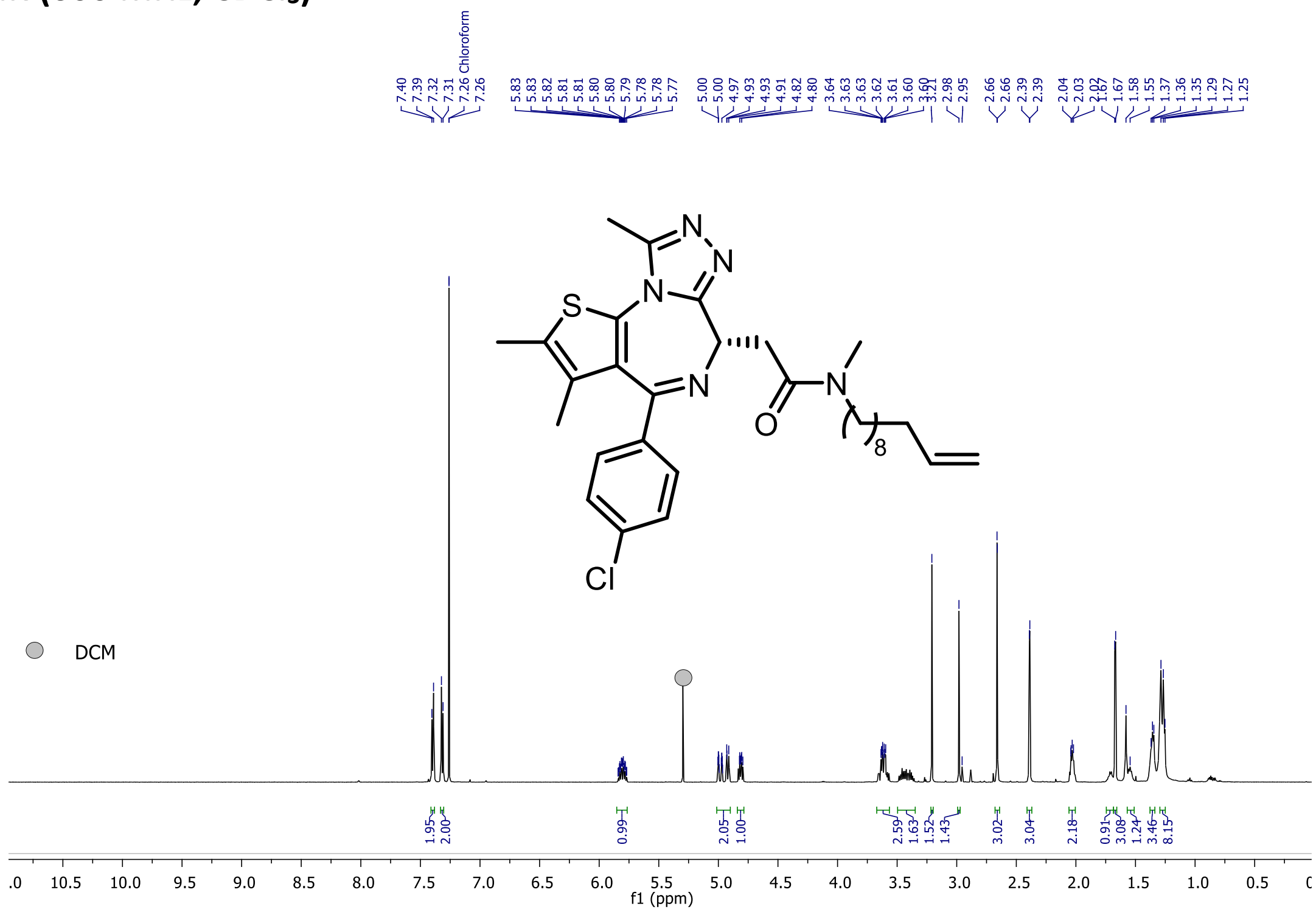

^{13}C NMR (151 MHz, CDCl$_3$)

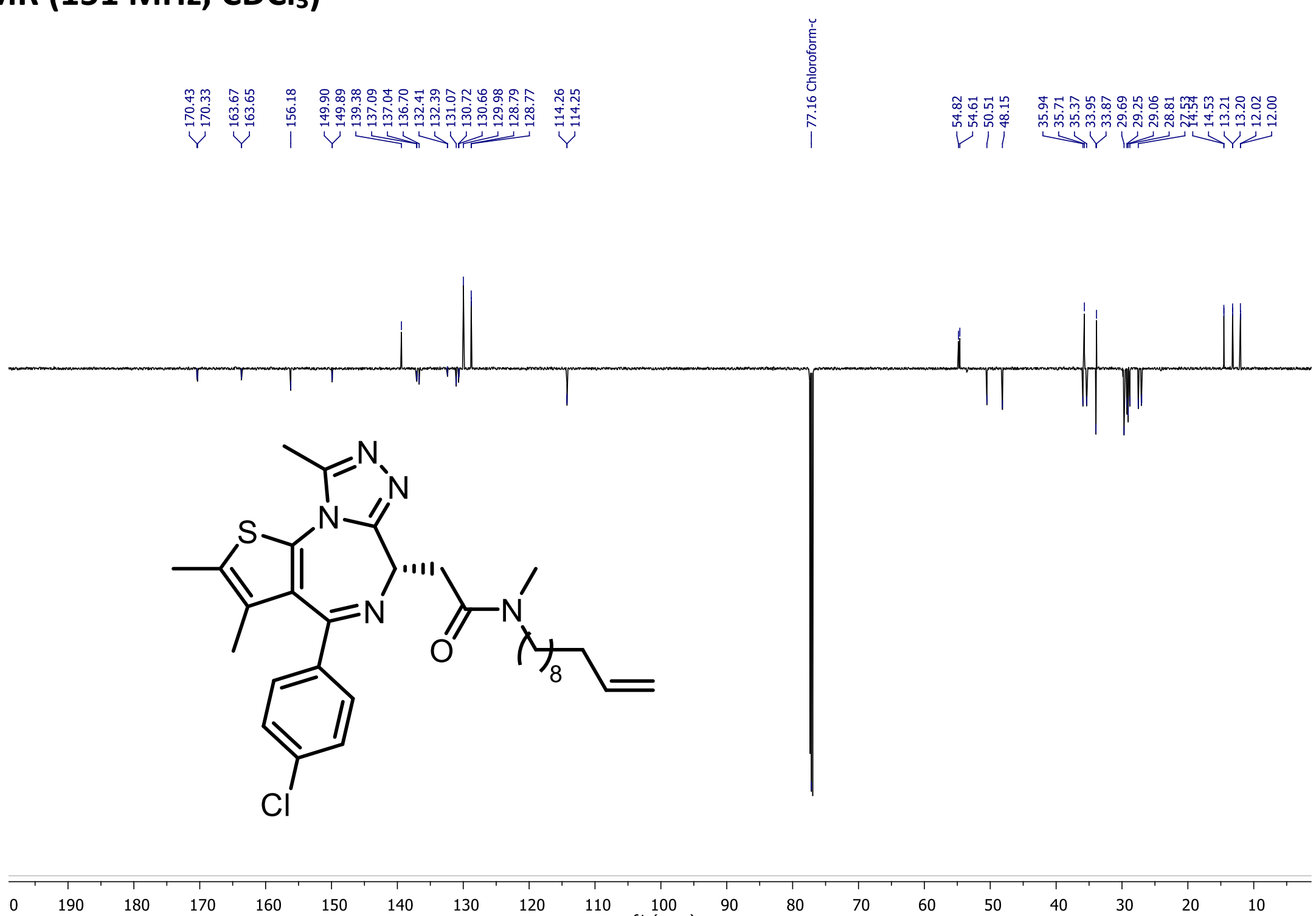

(*4R*)-4-((*8R,9S,10S,13R,14S,17R*)-10,13-Dimethyl-3,7,12-trioxohexadecahydro-1*H*-cyclopenta[*a*]phenanthren-17-yl)-*N*-methyl-*N*-(undec-10-en-1-yl)pentanamide (S1.20b)

^{1}H NMR (700 MHz, $CDCl_3$)

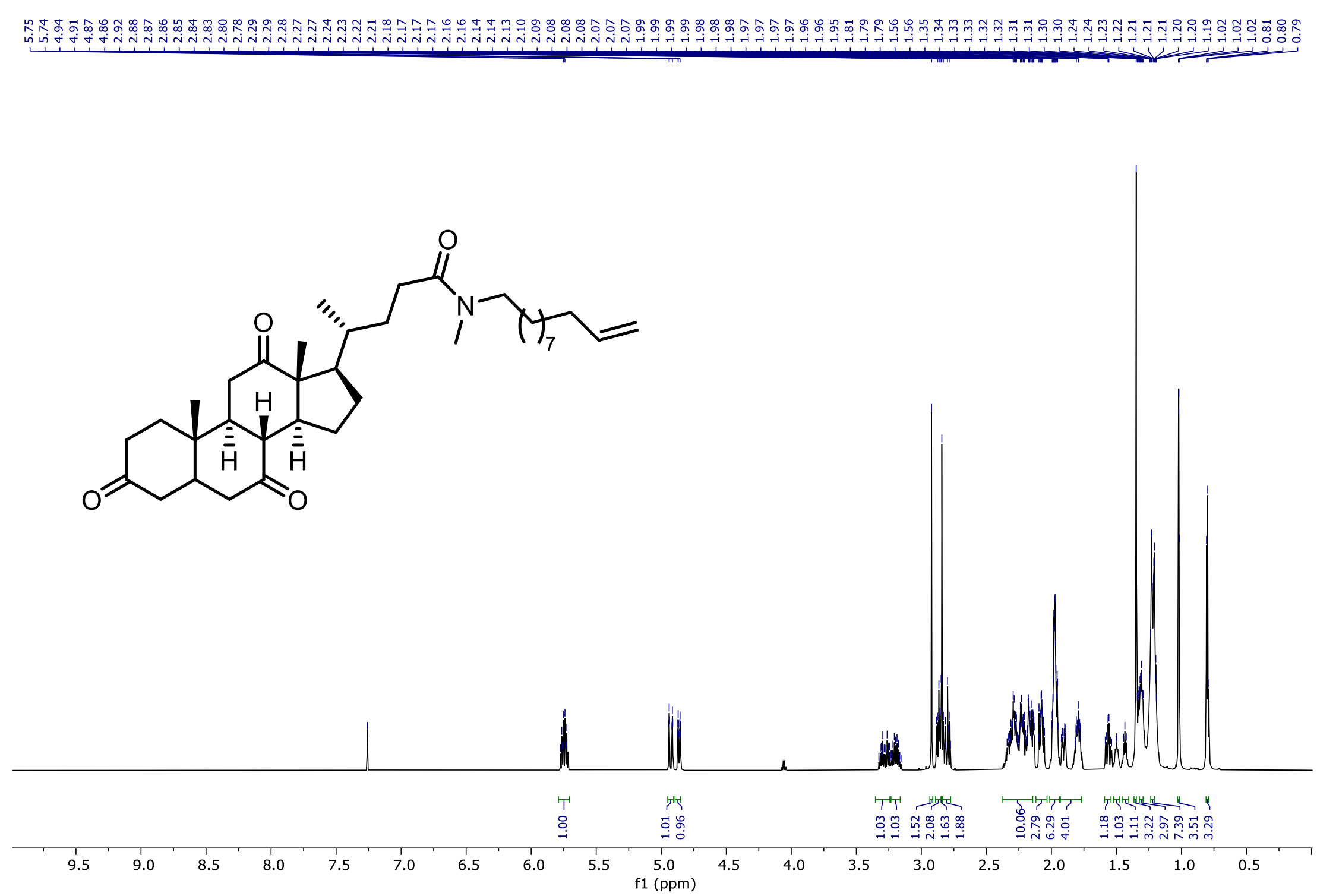

^{13}C NMR (176 MHz, $CDCl_3$)

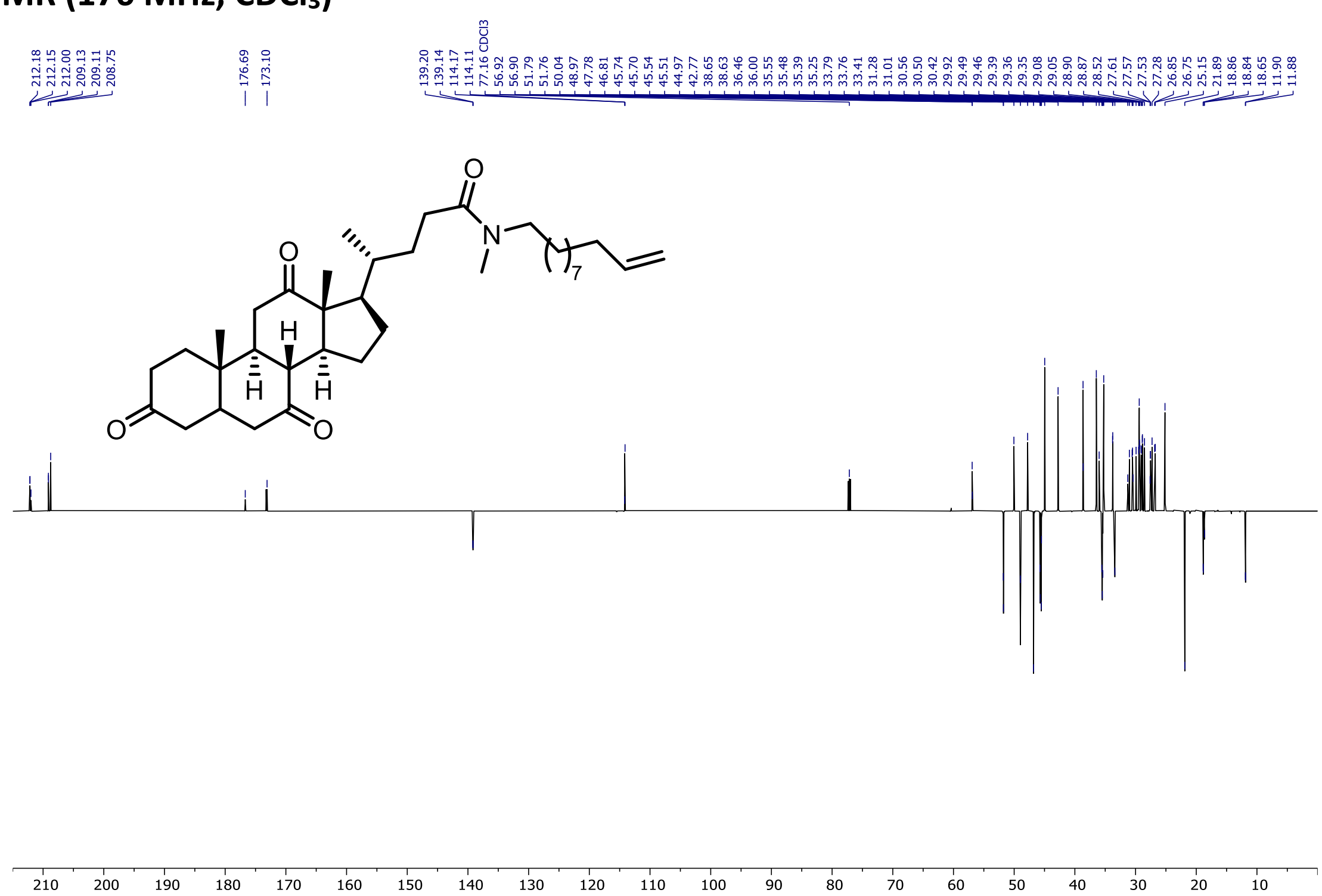

2-(3-Cyano-4-isobutoxyphenyl)-*N*,4-dimethyl-*N*-(undec-10-en-1-yl)thiazole-5-carboxamide (S1.22b)

^{1}H NMR (400 MHz, CDCl$_3$)

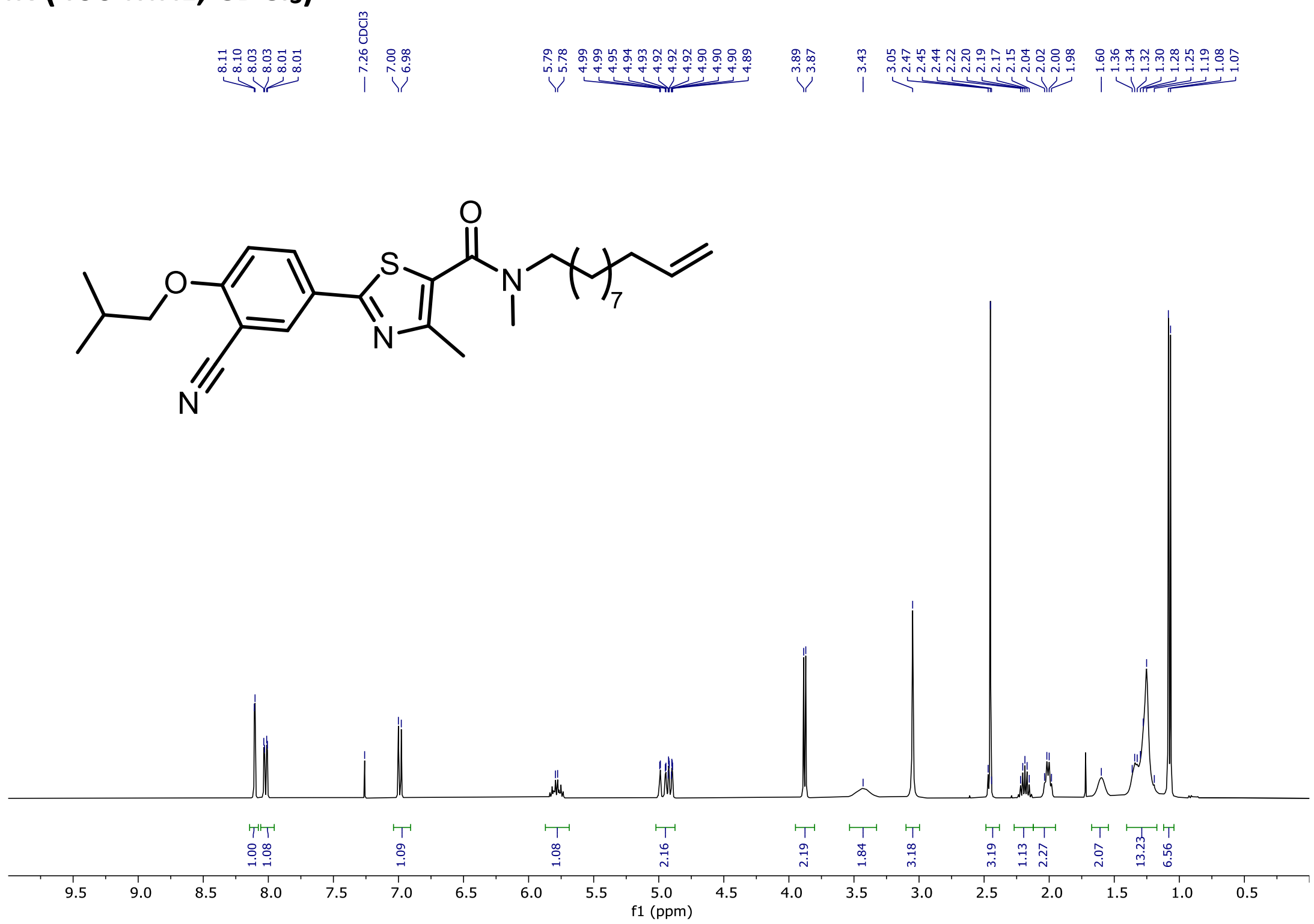

^{13}C NMR (101 MHz, CDCl$_3$)

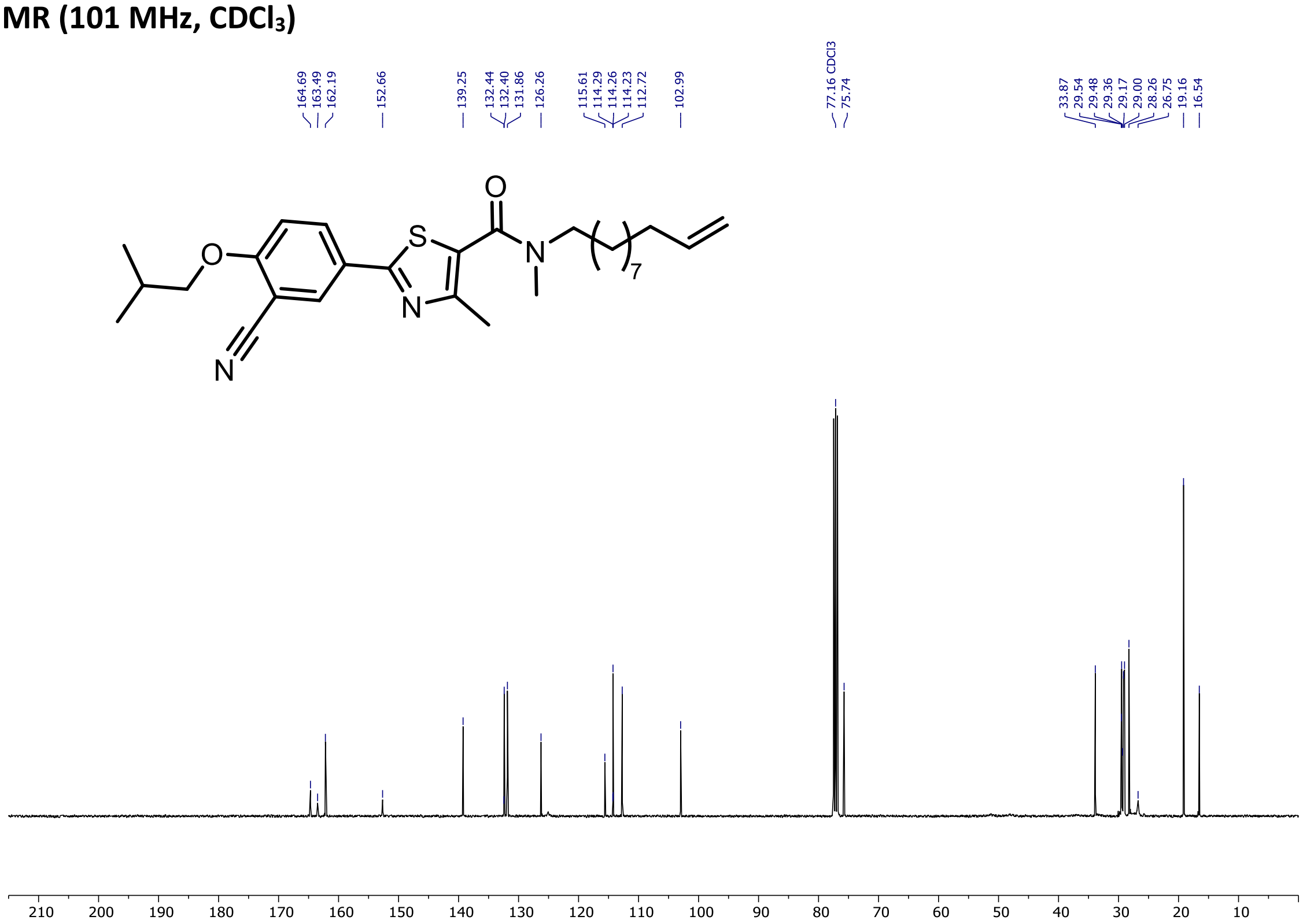

N-Methyl-2,4,6-tris(trifluoromethyl)-*N*-(undec-10-en-1-yl)benzenesulfonamide (1.44b)

^{1}H NMR (400 MHz, CDCl$_3$)

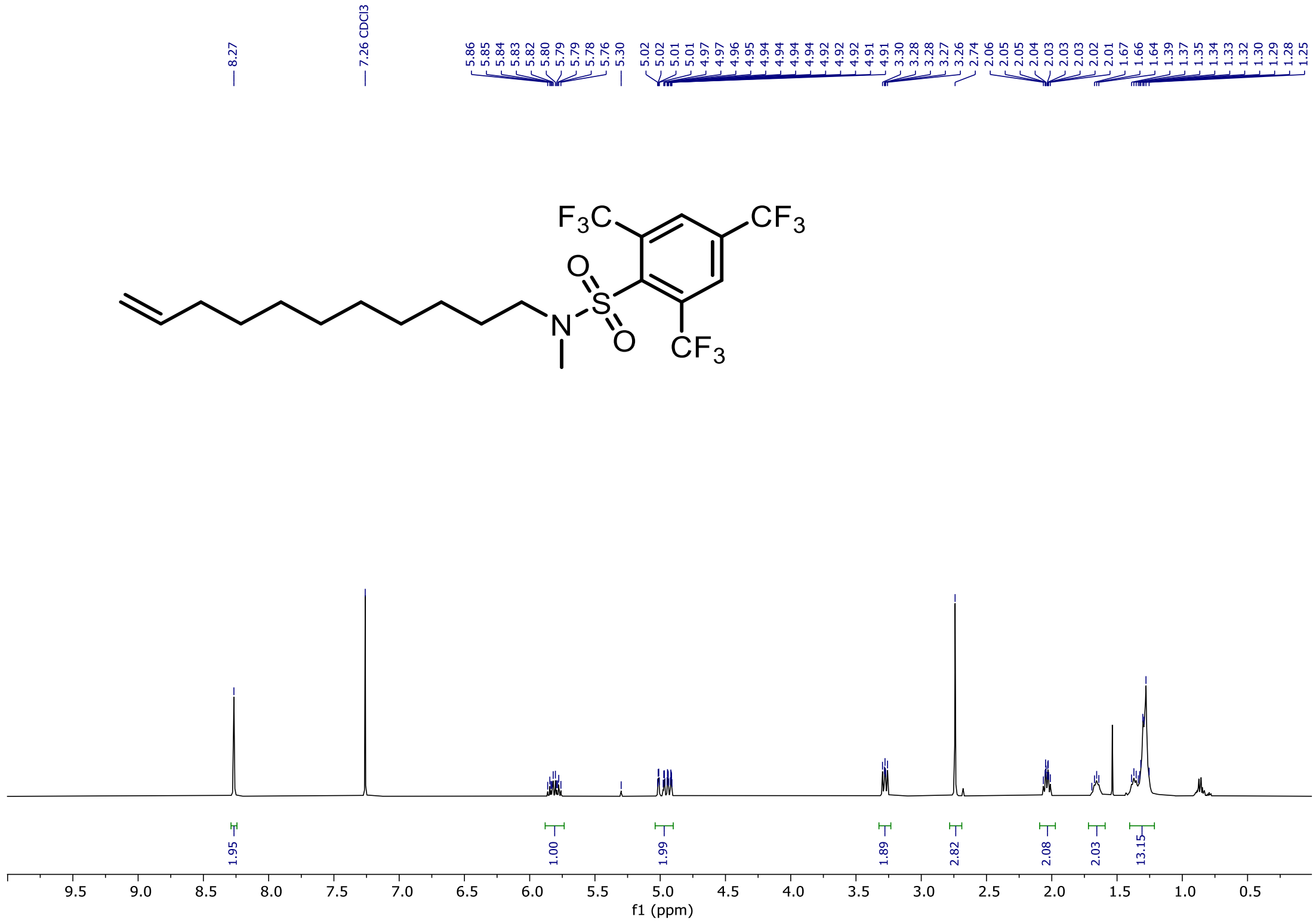

^{13}C NMR (101 MHz, CDCl$_3$)

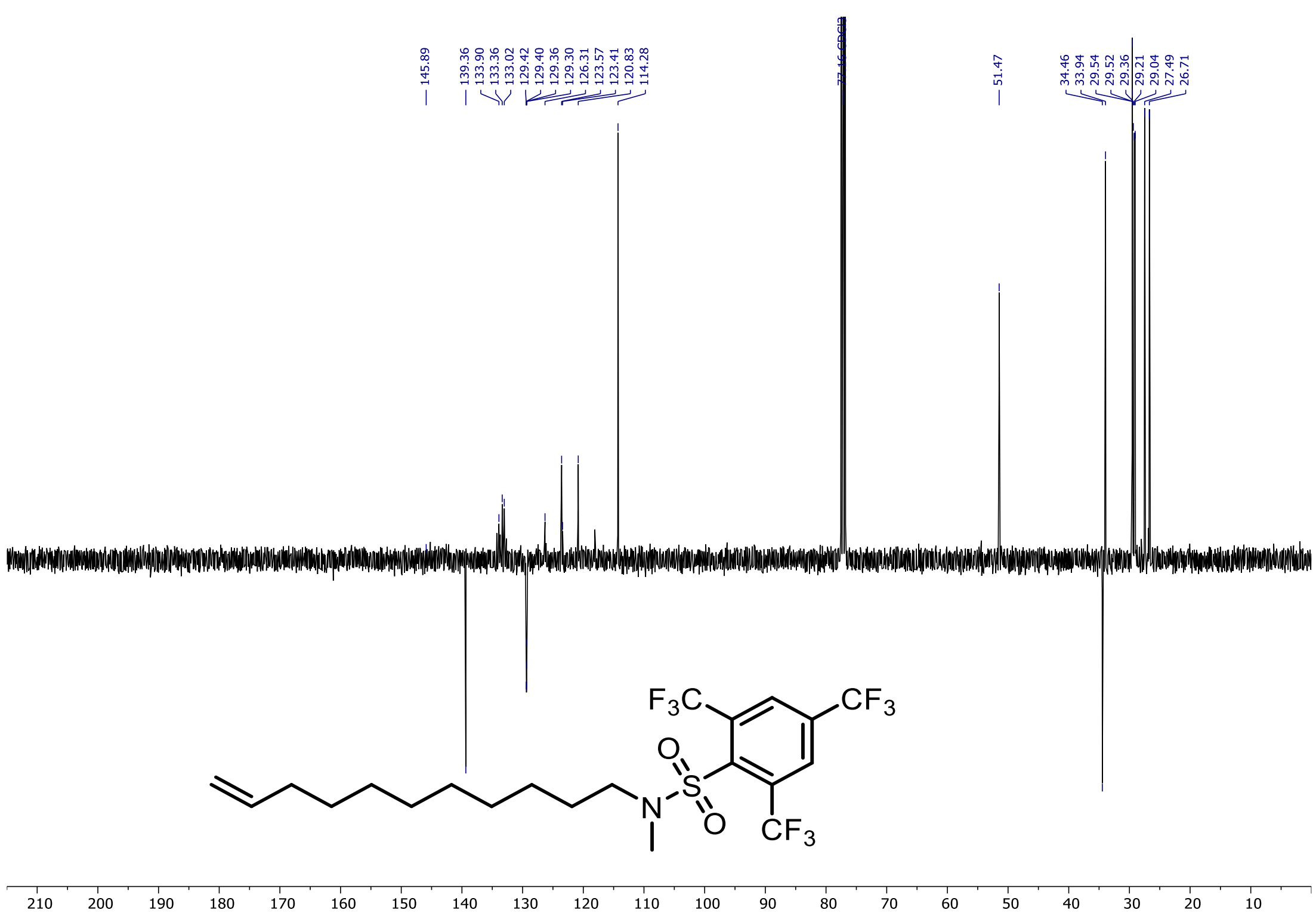

^{19}F NMR (376 MHz, CDCl$_3$)

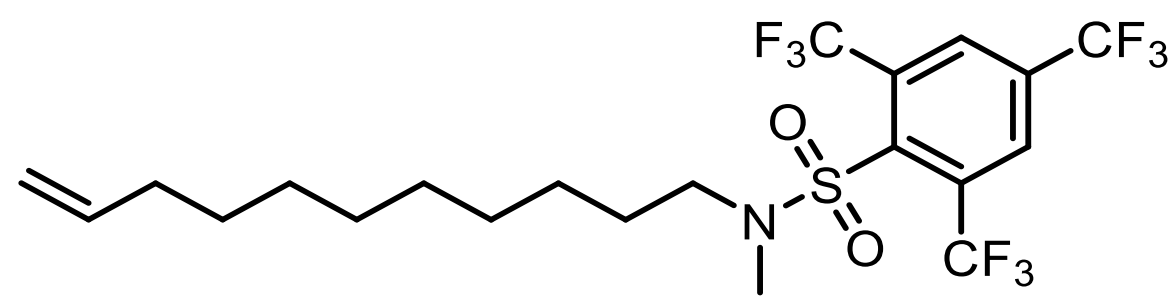

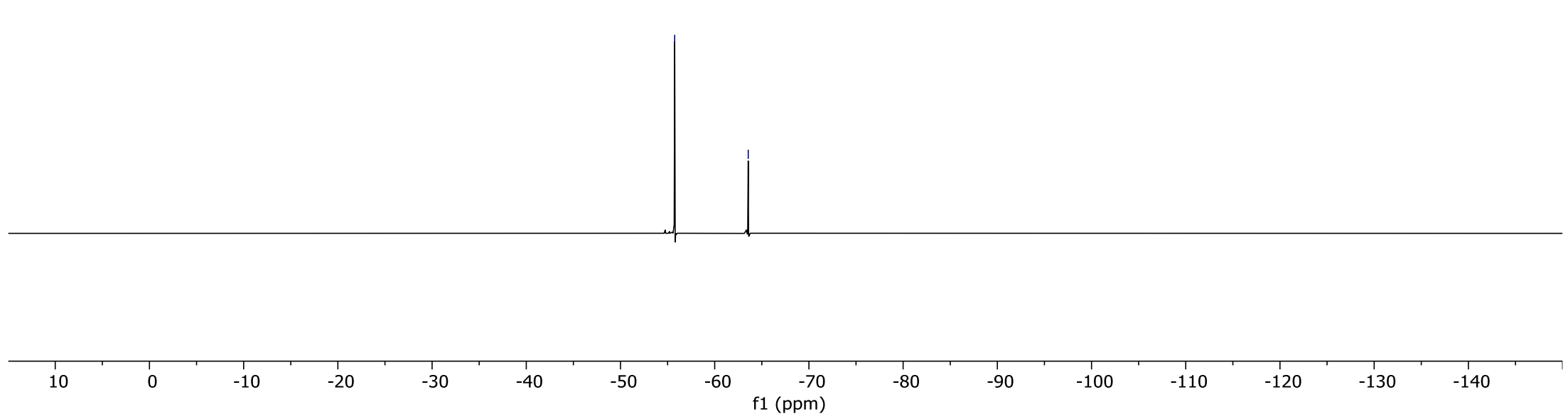

***N*-Methyl-N-(2-(methylamino)ethyl)isoquinoline-5-sulfonamide (S3.01a)**

^{1}H NMR (400 MHz, CDCl$_3$)

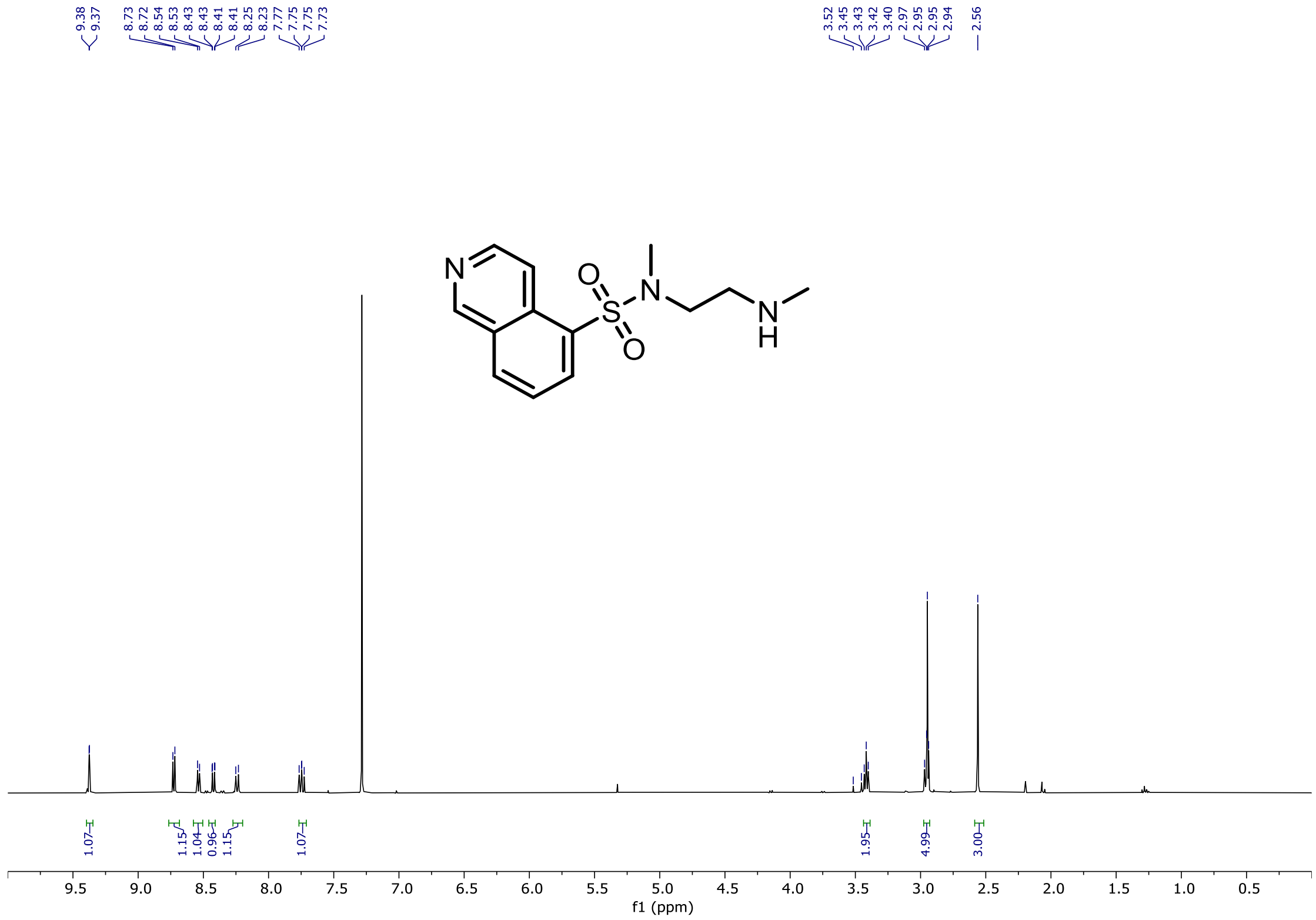

3-(4-Ethynylphenyl)pyridine (S3.05b)

^{1}H NMR (400 MHz, $CDCl_3$)

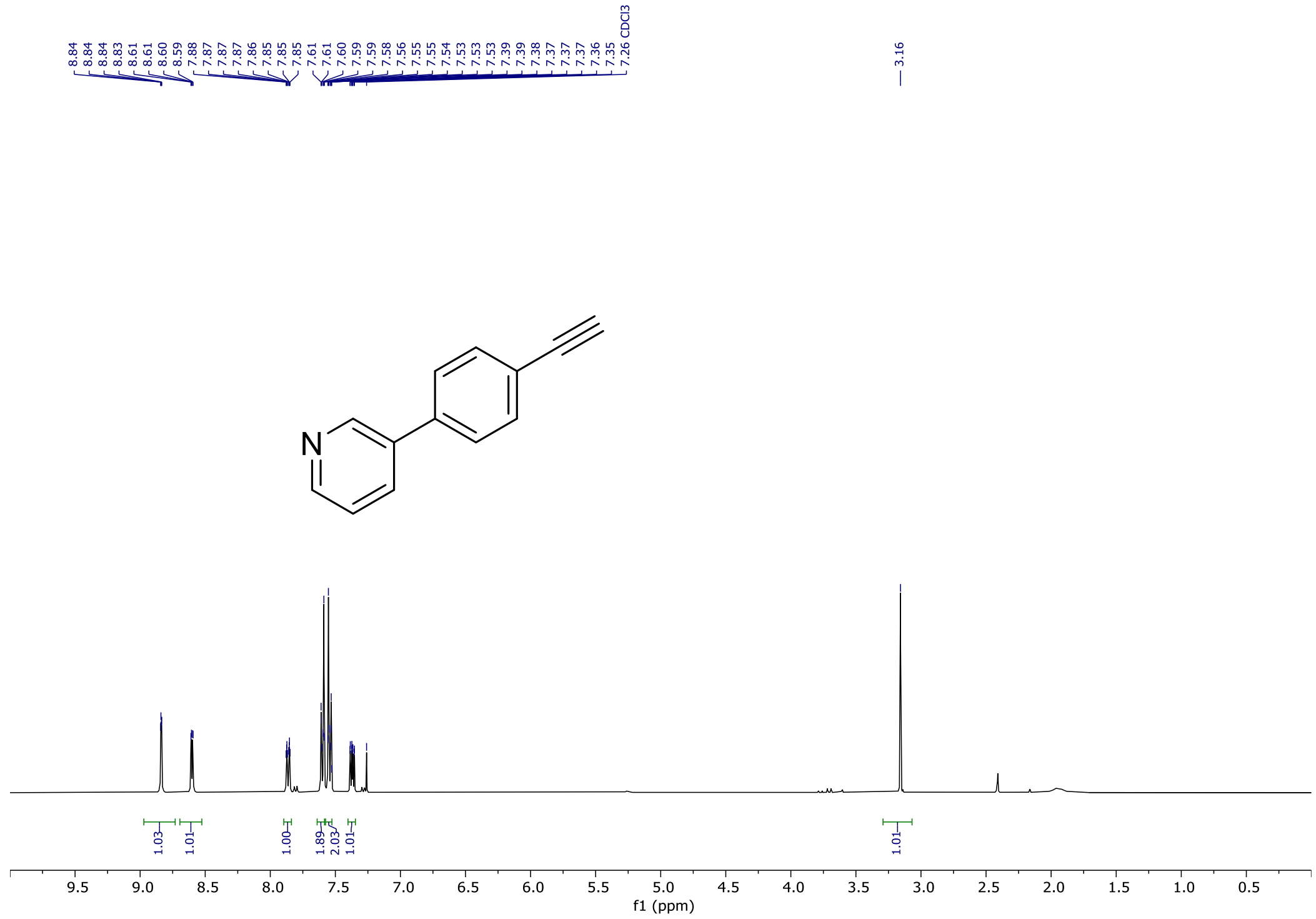

1-((*S*)-2-(Dodec-11-enamido)-3,3-dimethylbutanoyl)-4-hydroxy-*N*-(4-(4-methylthiazol-5 yl)benzyl)pyrrolidine-2-carboxamide (4.01b)

^{1}H NMR (600 MHz, $CDCl_3$)

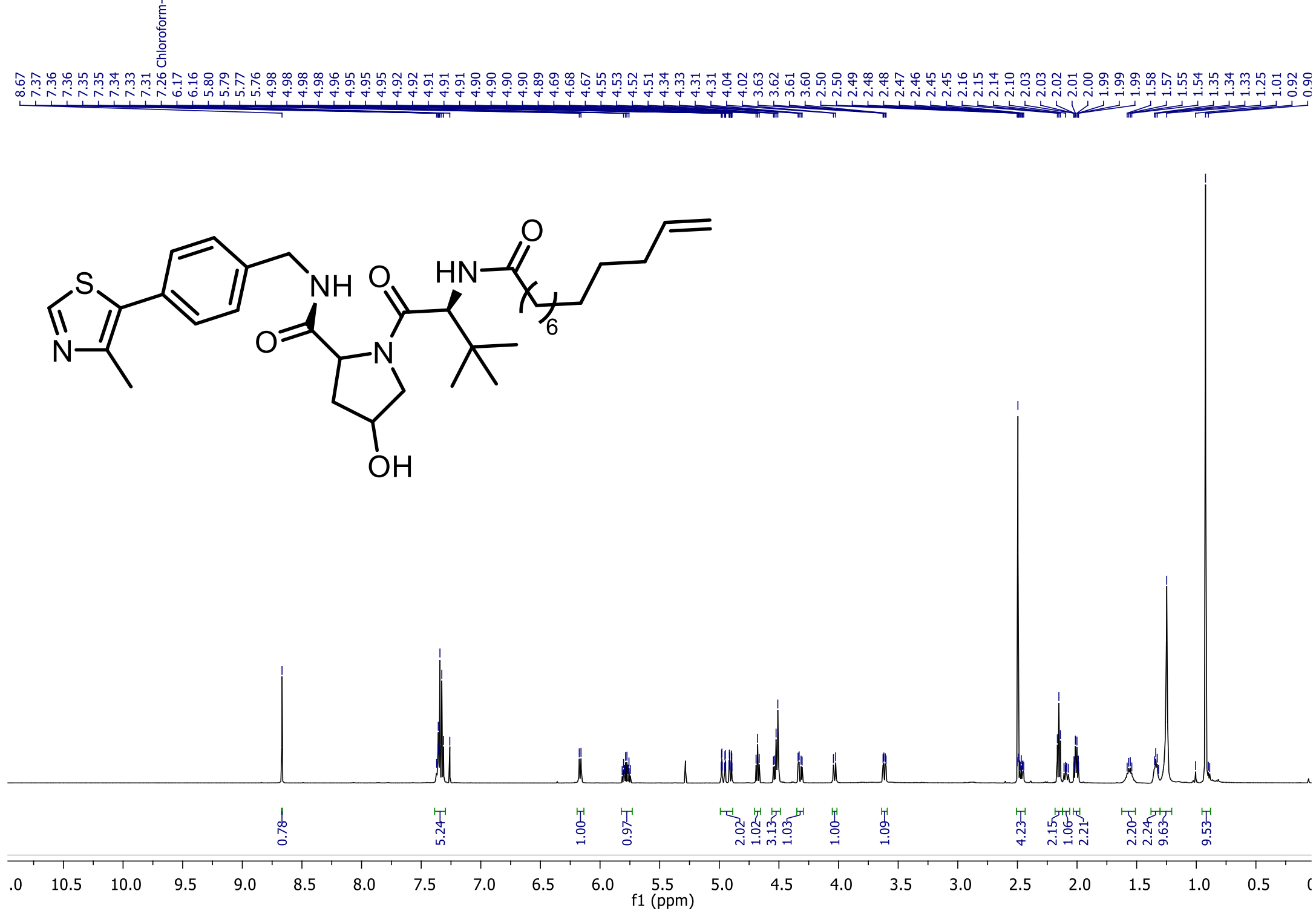

^{13}C NMR (151 MHz, $CDCl_3$)

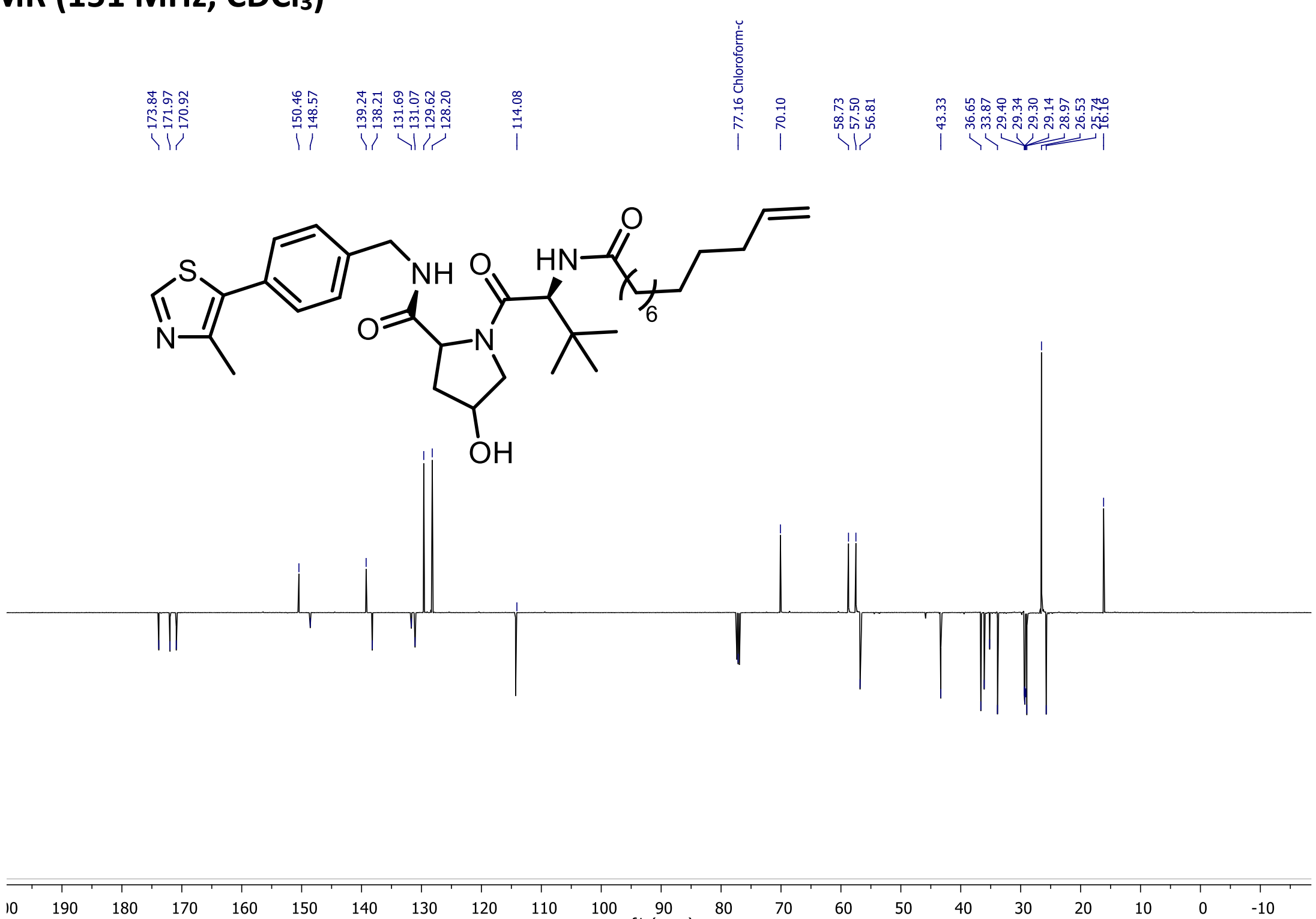

N-(2-(2,6-Dioxopiperidin-3-yl)-1,3-dioxoisoindolin-5-yl)dodec-11-enamide (4.02b)
^{1}H NMR (400 MHz, DMSO-*d6*)

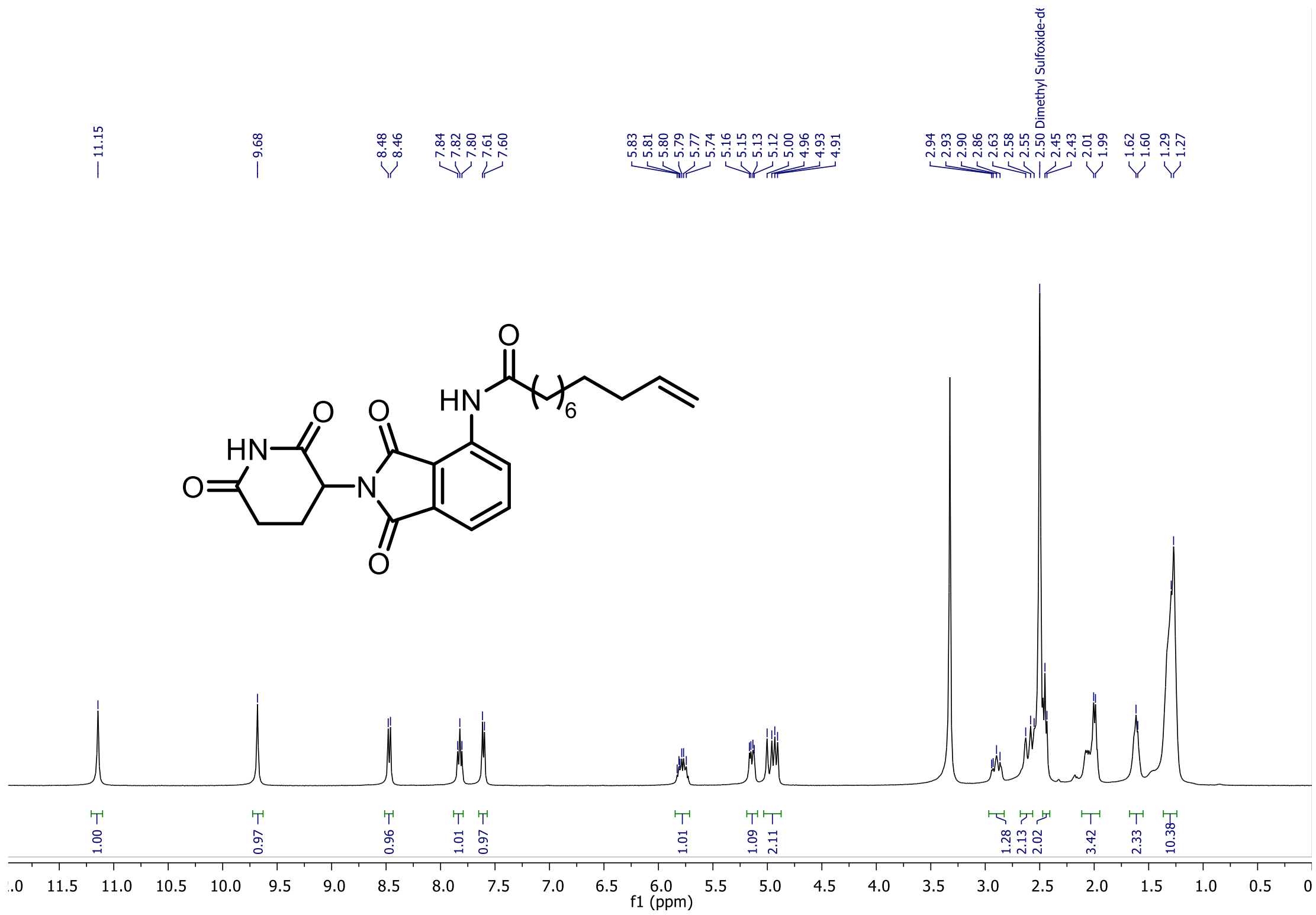

^{13}C NMR (101 MHz, DMSO-*d6*)

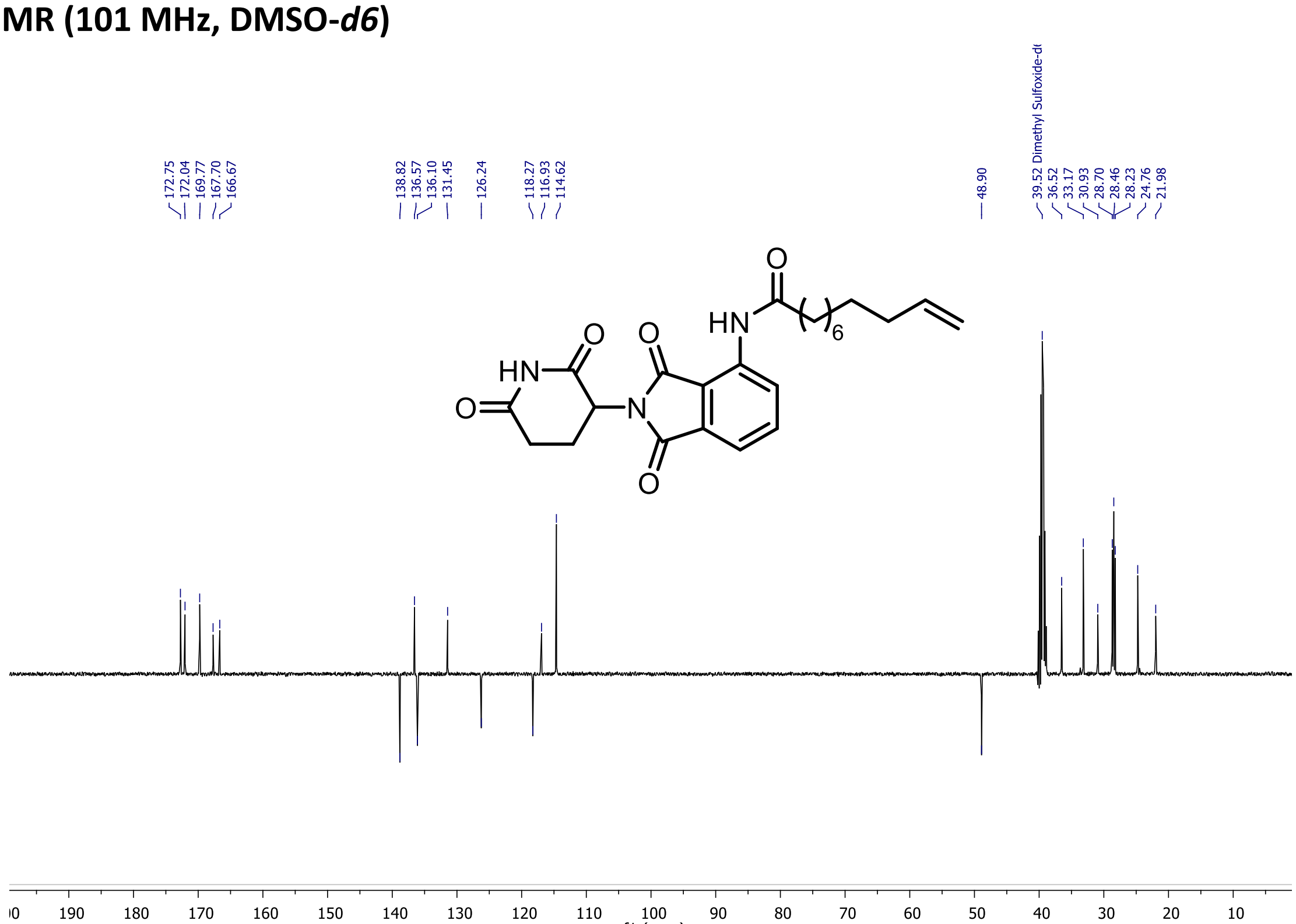

Me-Gly-Pro-Phe-Trp(Tos)-Glu(OMe) (S4.04a)

^{1}H NMR (400 MHz, MeOD)

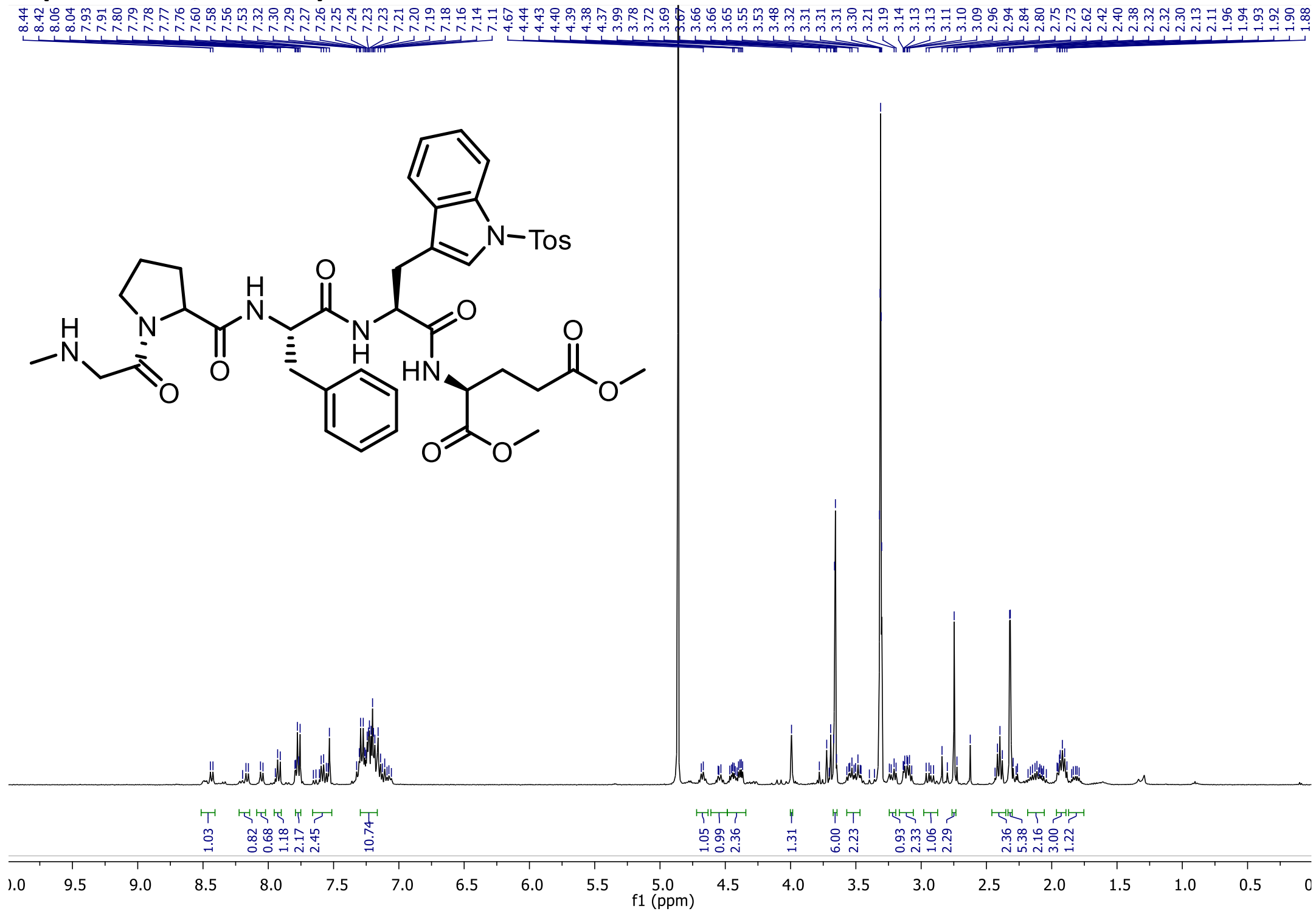

^{13}C NMR (101 MHz, MeOD)

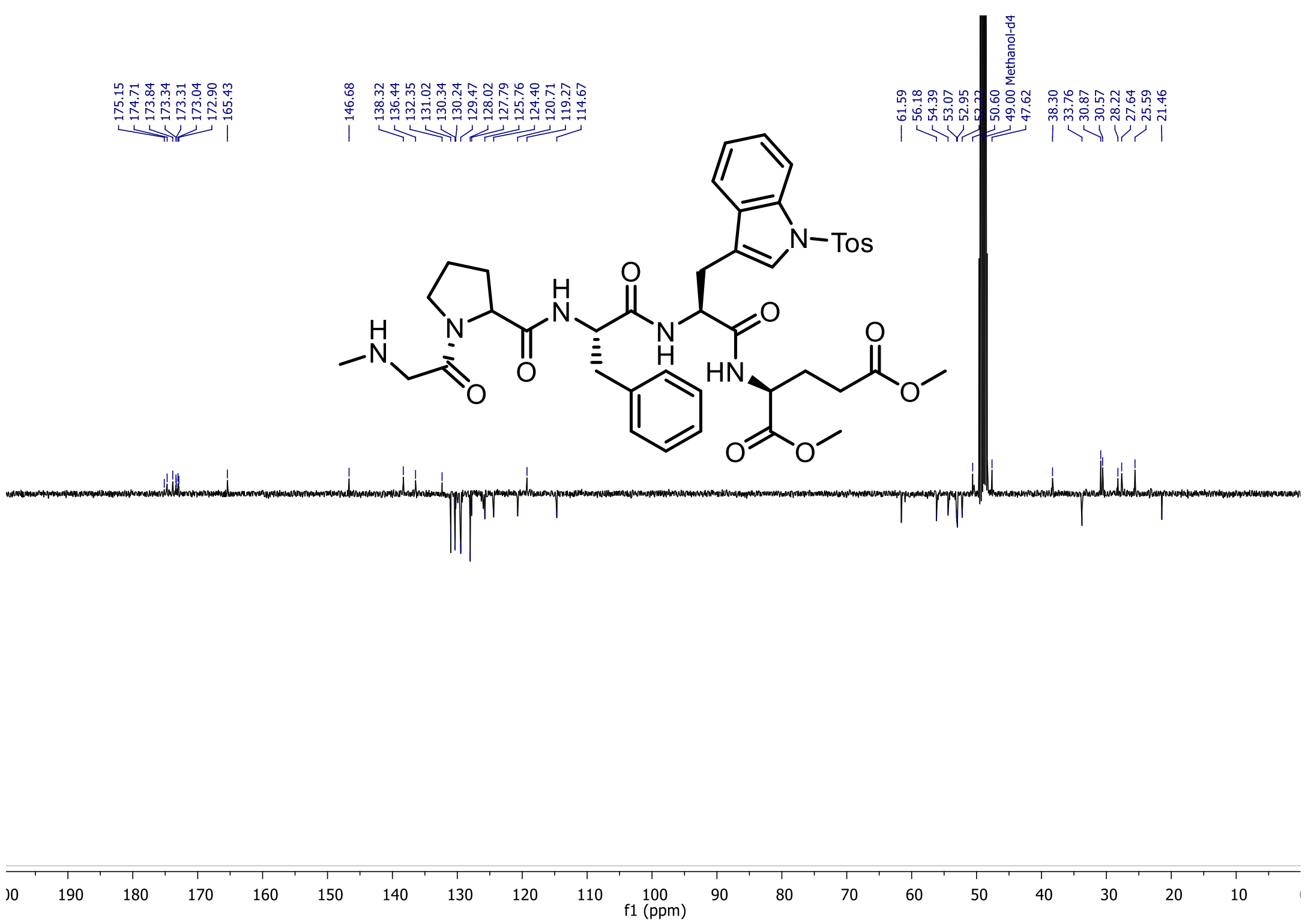

Me-Gly-Pro-Phe (S4.05a)

^{1}H NMR (400 MHz, DMSO-*d6*)

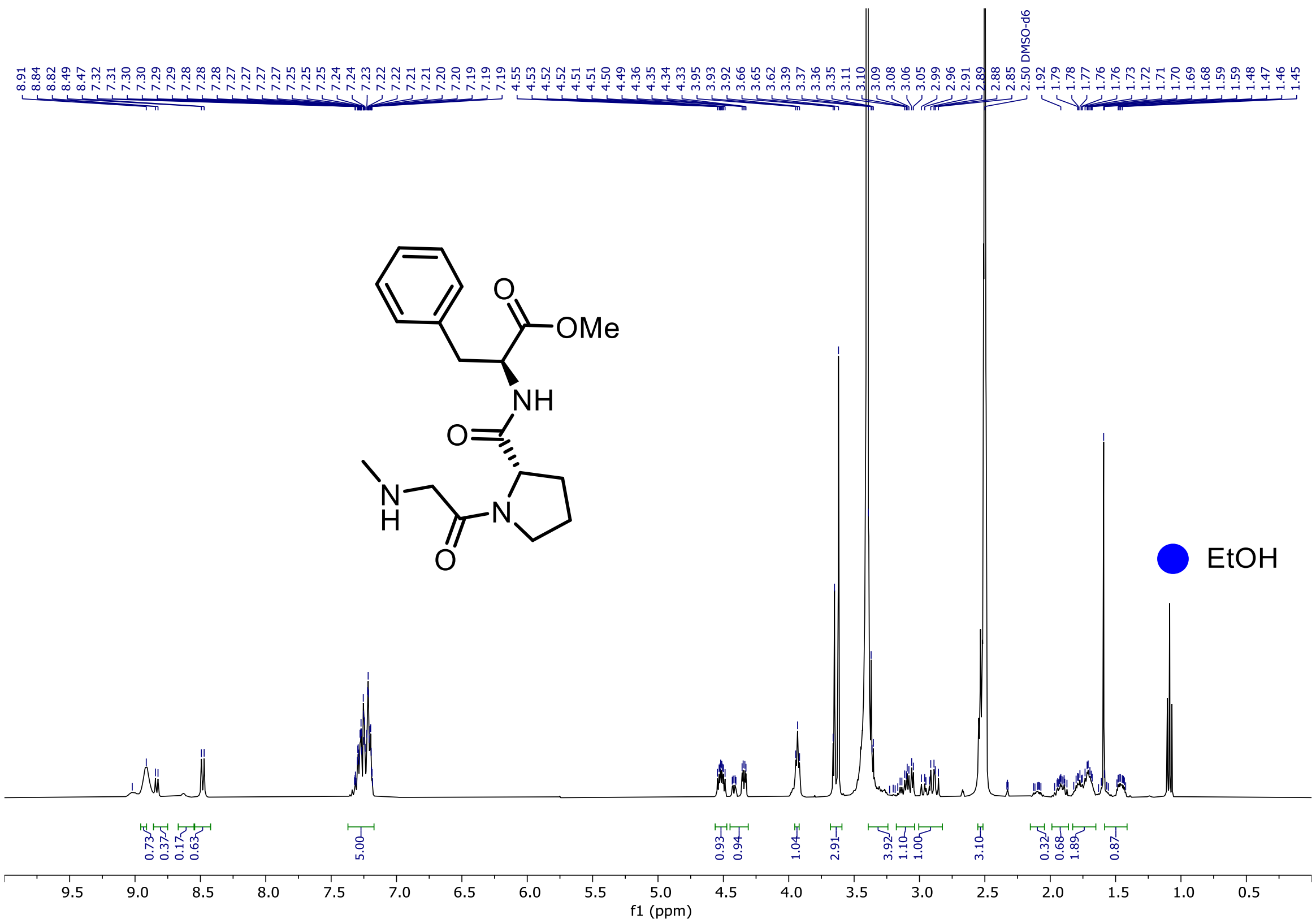

^{13}C NMR (101 MHz, DMSO-*d6*)

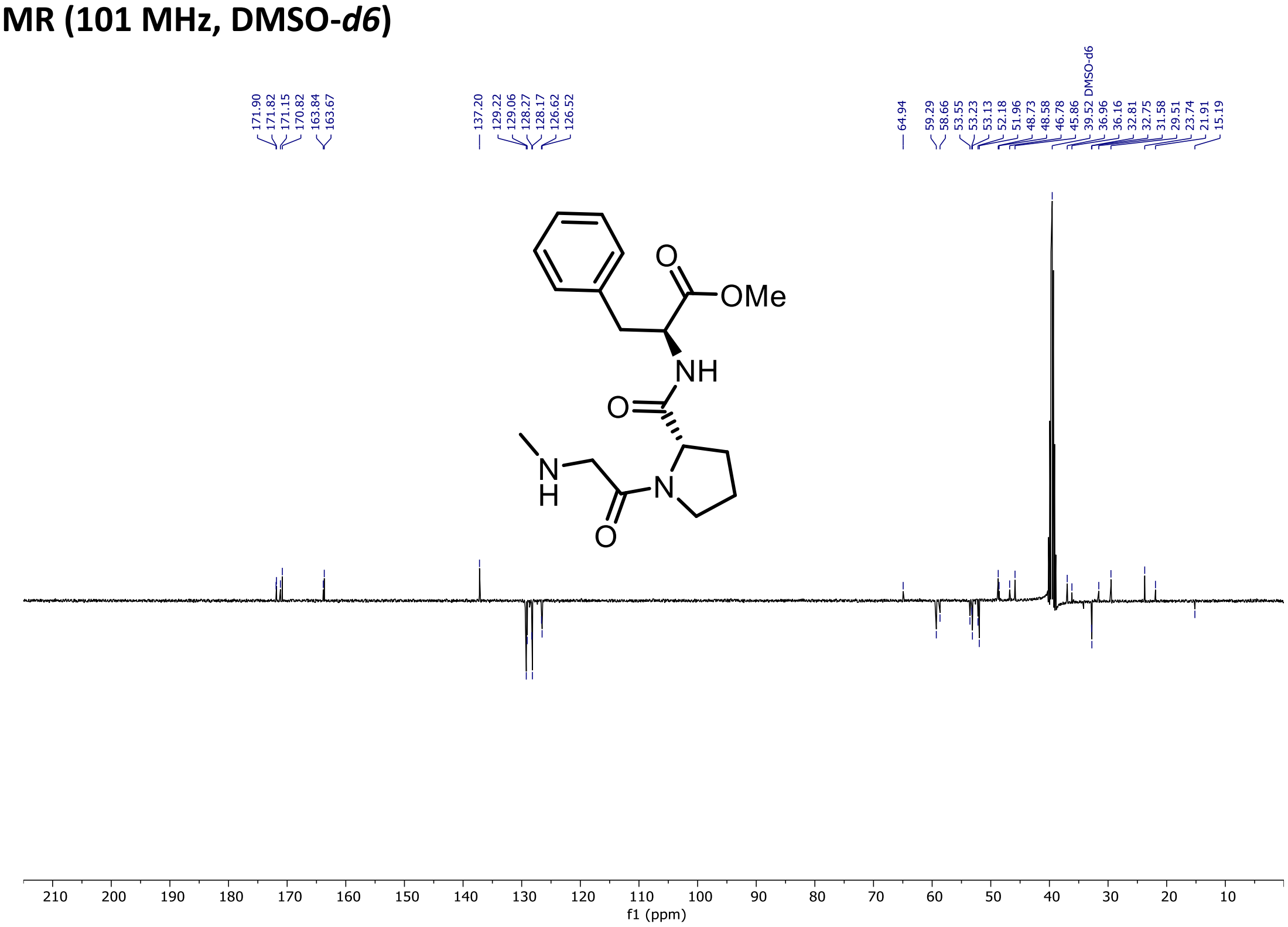

Me-Gly-Pro-Phe-Glu(OMe) (S4.06a)

^{1}H NMR (600 MHz, MeOD)

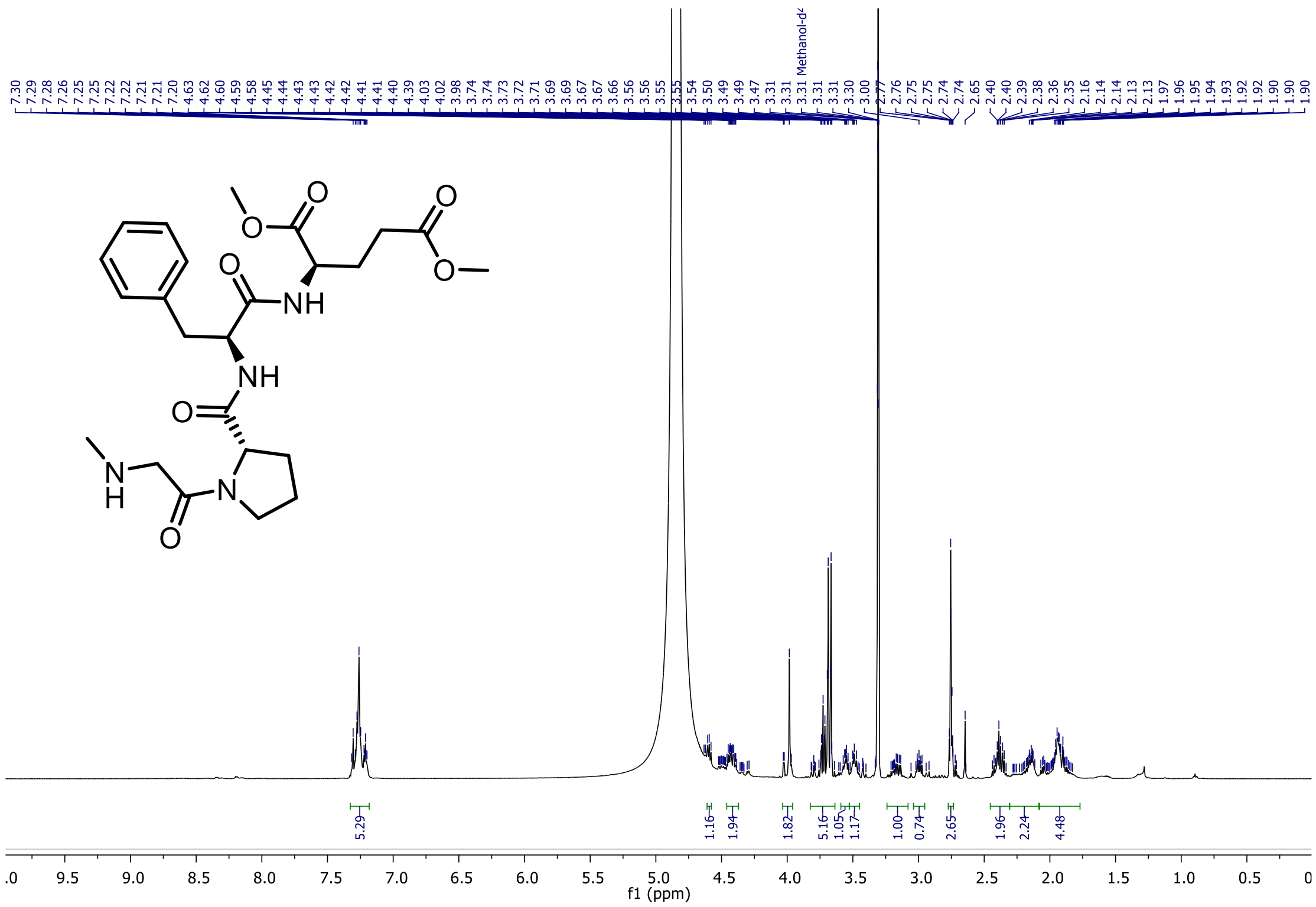

^{13}C NMR (151 MHz, MeOD)

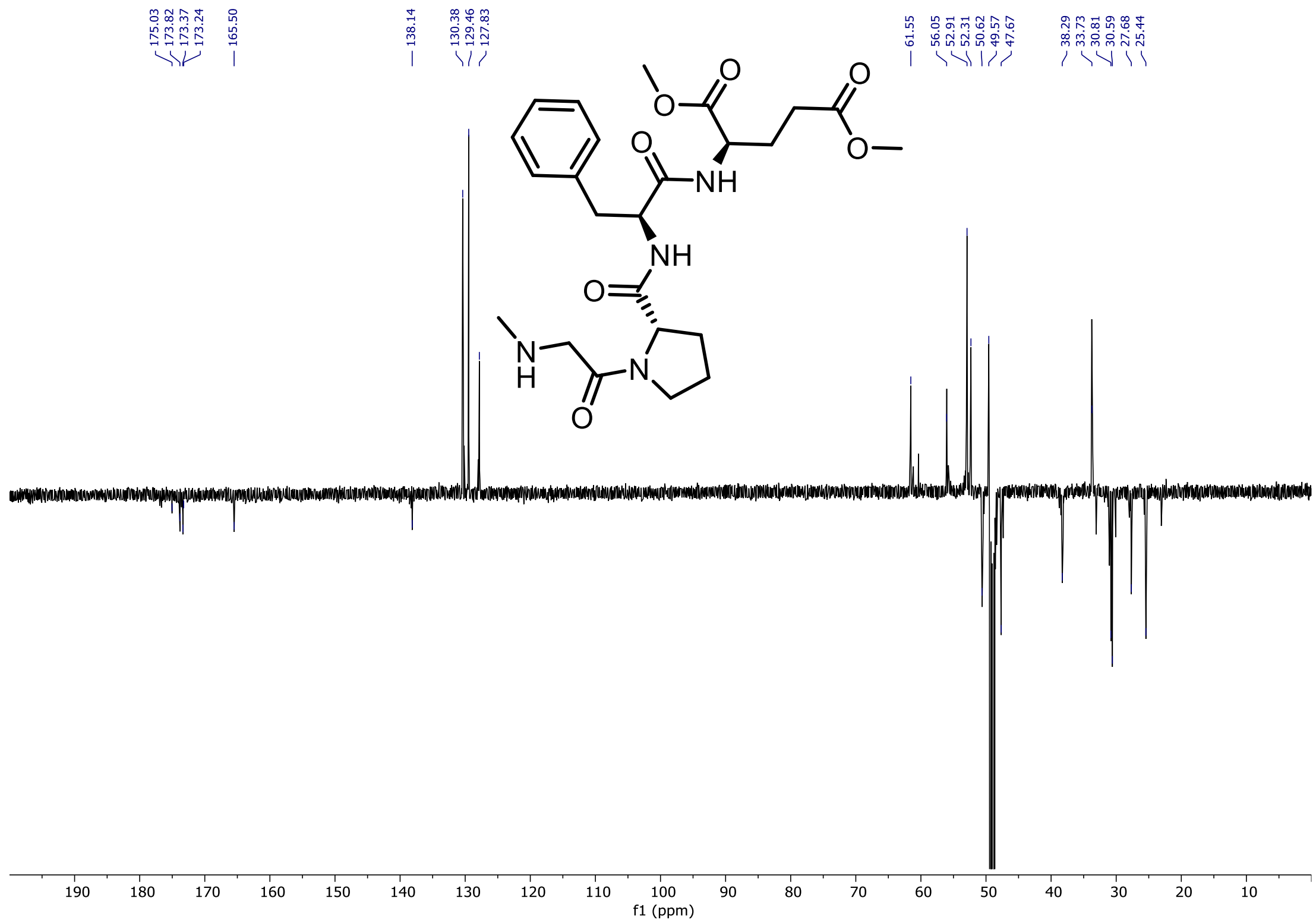

3-(9,10-Ethanoanthracen-9(10*H*)-yl)-*N*-(3-cyclohexylpropyl)propan-1-amine (1.01)

^{1}H NMR (400 MHz, $CDCl_3$)

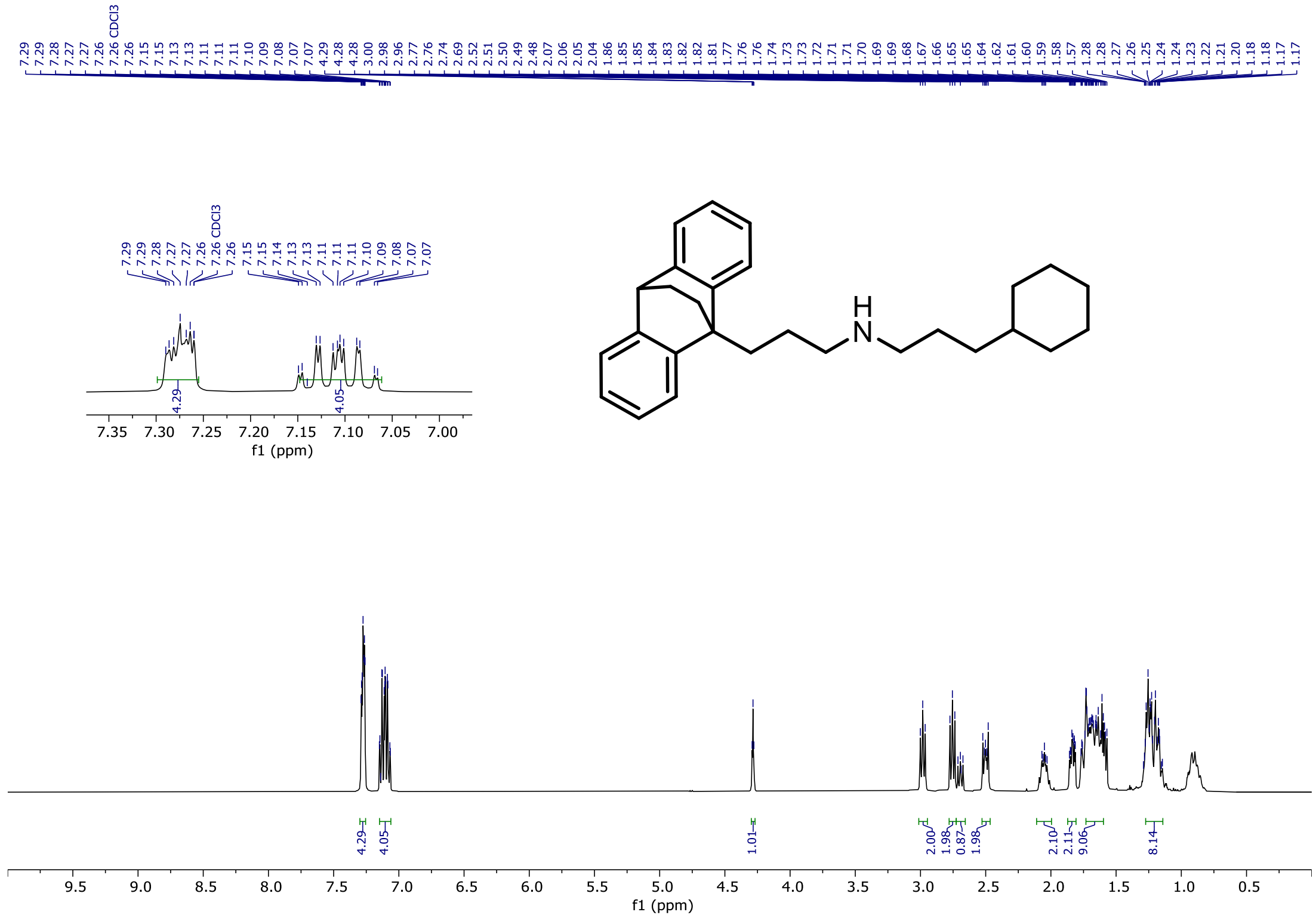

^{13}C NMR (101 MHz, $CDCl_3$)

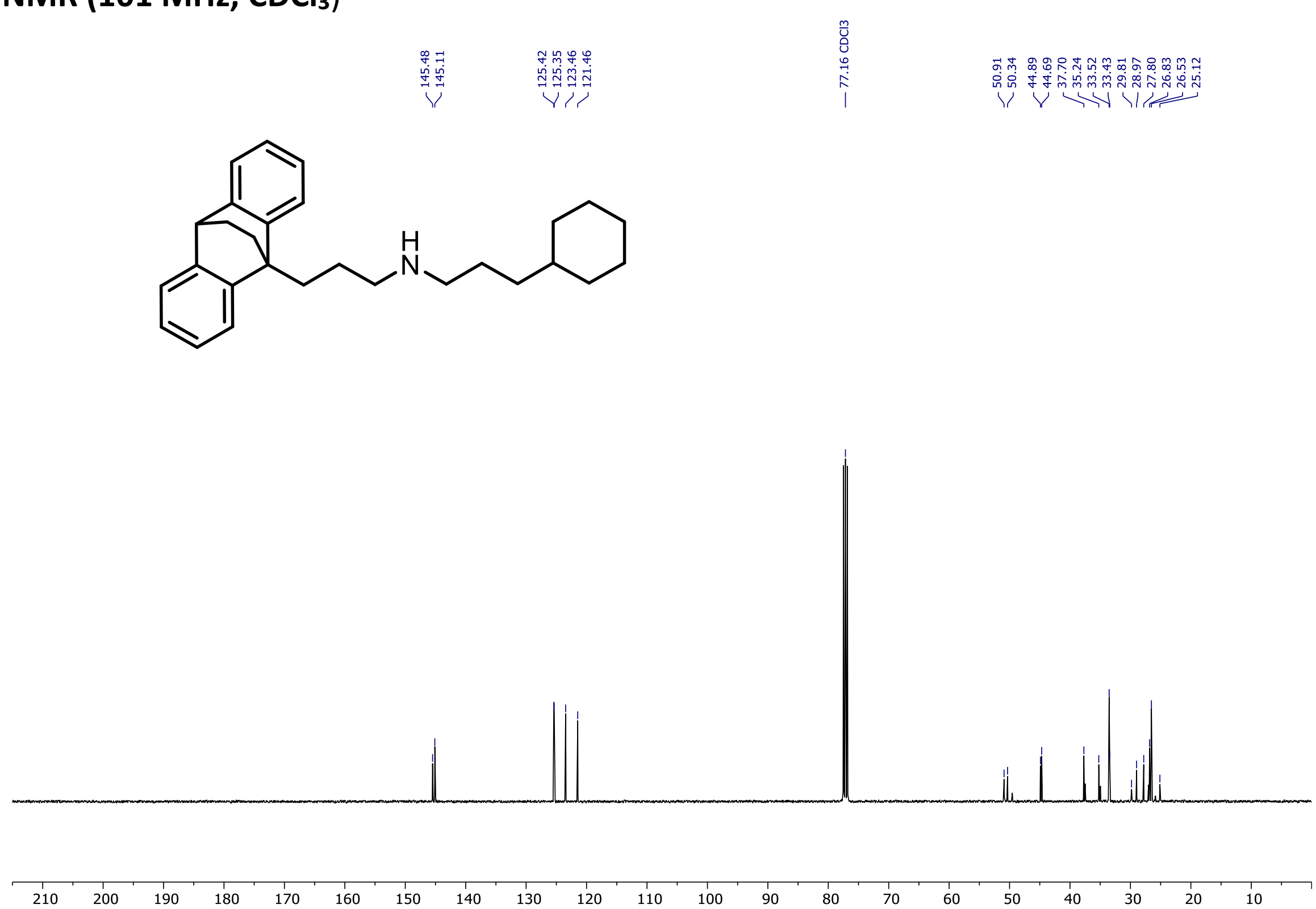

(*S*)-3-(Naphthalen-1-yloxy)-*N*-(3-phenylpropyl)-3-(thiophen-2-yl)propan-1-amine (1.02)

^{1}H NMR (600 MHz, CD_2Cl_2)

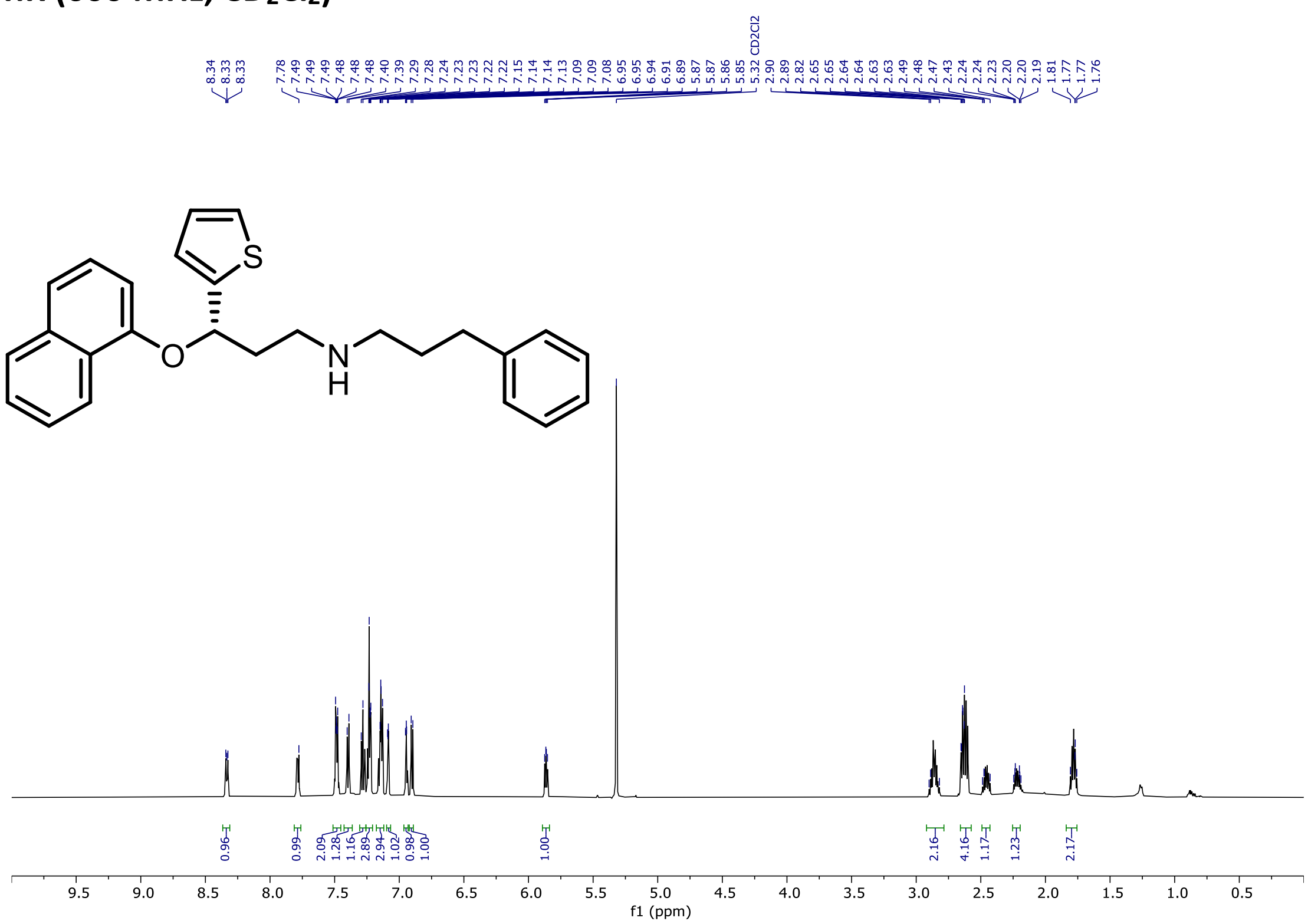

^{13}C NMR (151 MHz, CD_2Cl_2)

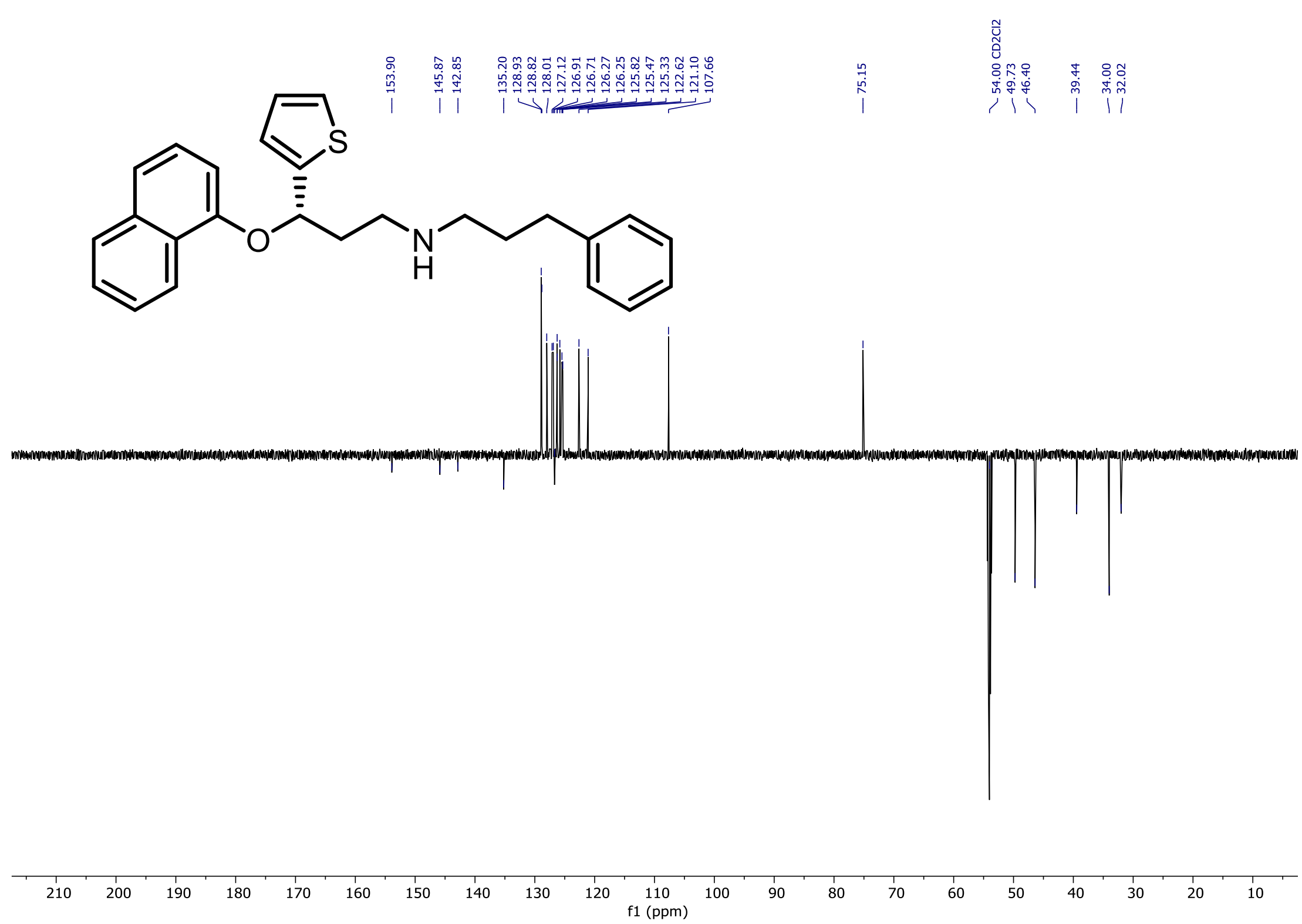

5-Phenyl-*N*-(2-(pyridin-2-yl)ethyl)pentan-1-amine (1.03)
^{1}H NMR (600 MHz, $CDCl_3$)

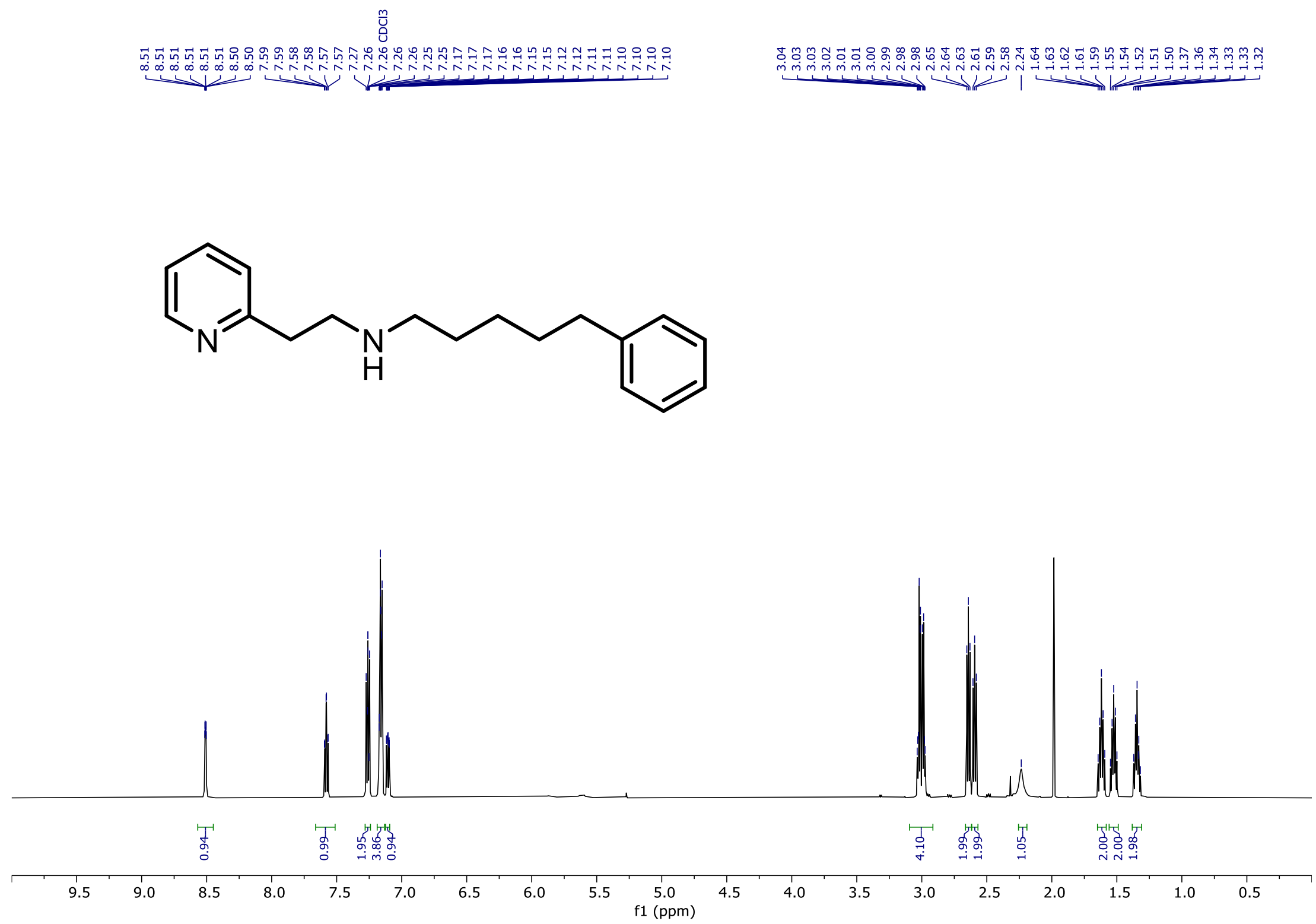

^{13}C NMR (151 MHz, $CDCl_3$)

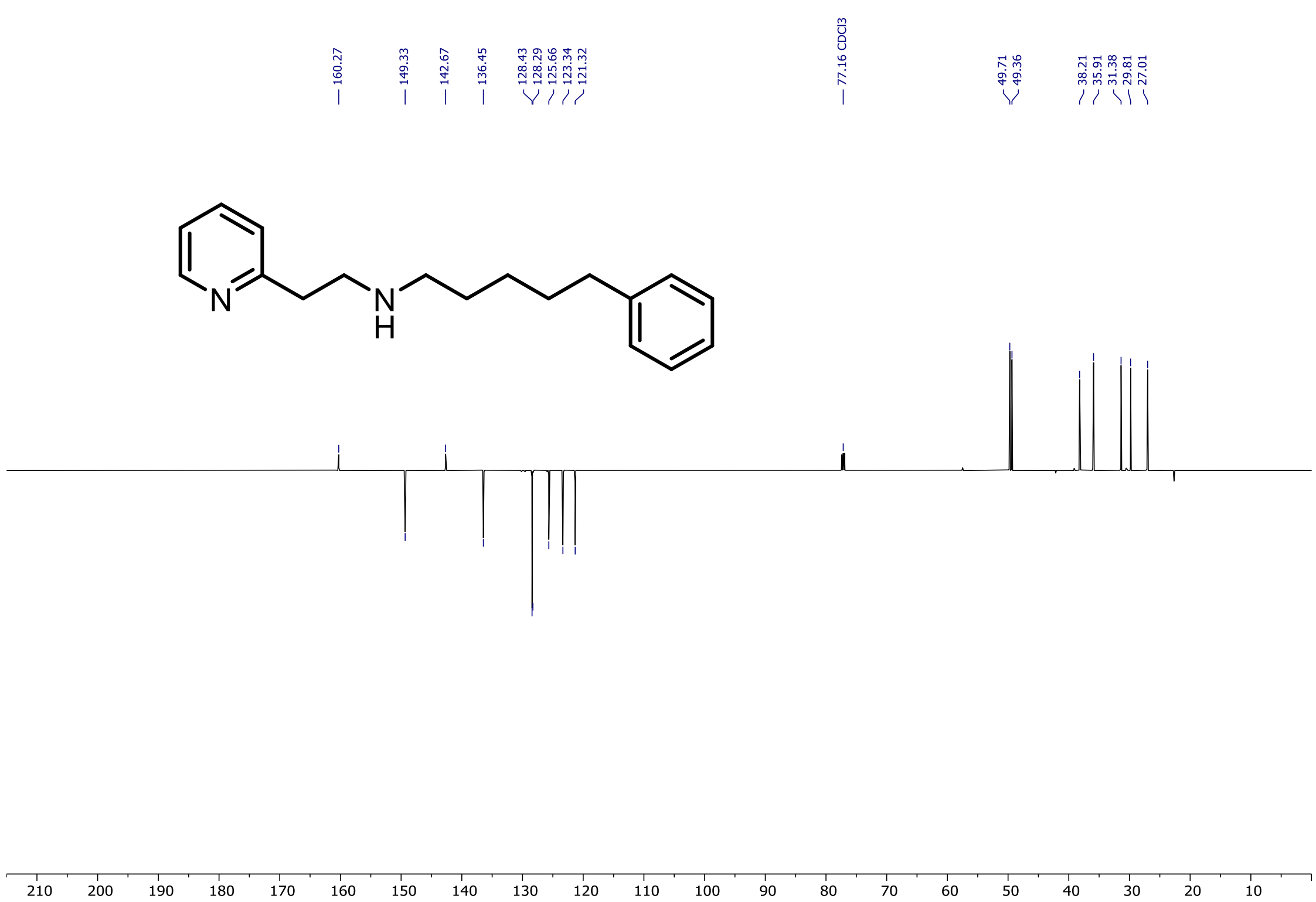

(1*S*,2*S*)-2-(Cinnamylamino)-1-phenylpropan-1-ol (1.04)

^{1}H NMR (400 MHz, $CDCl_3$)

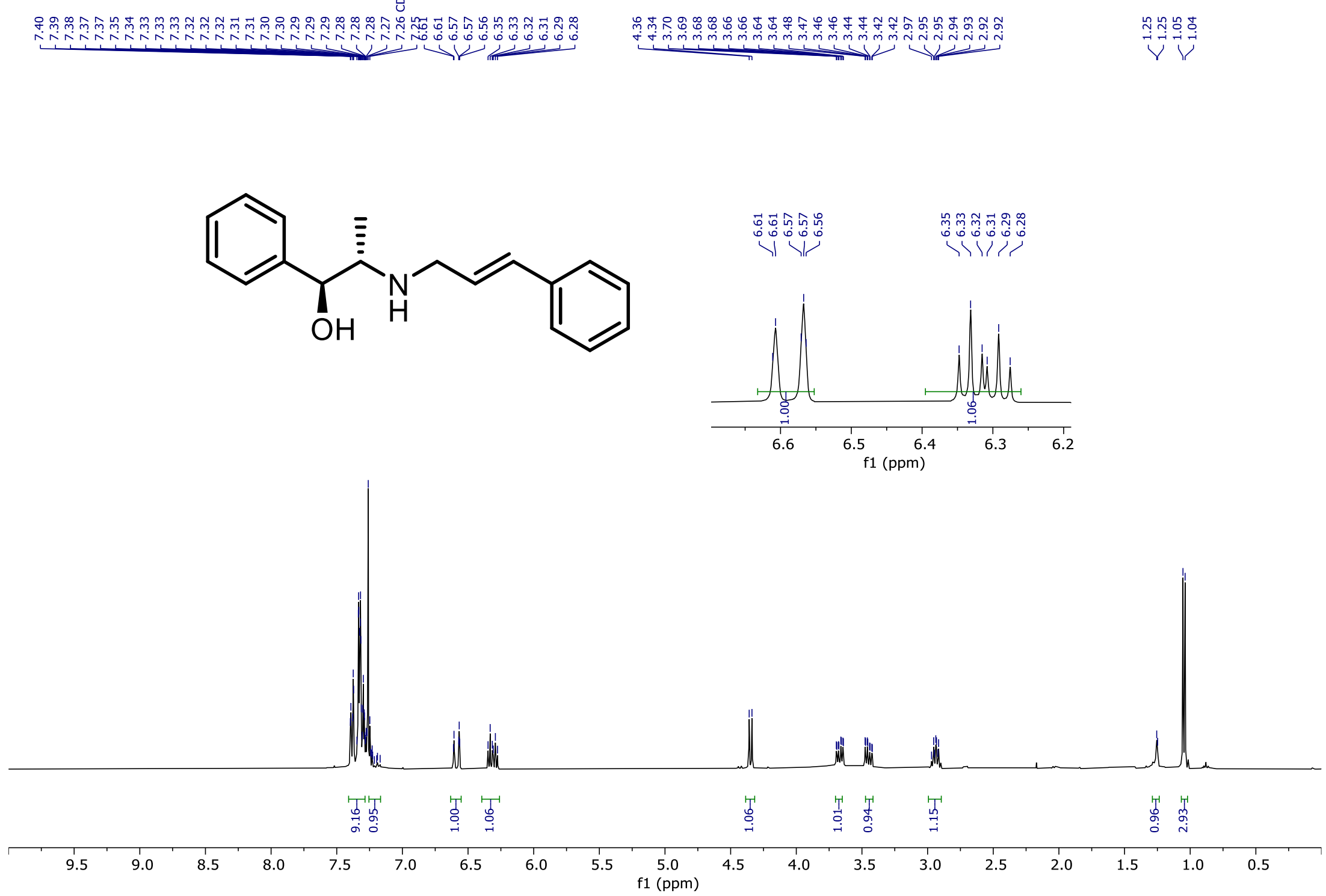

^{13}C NMR (151 MHz, $CDCl_3$)

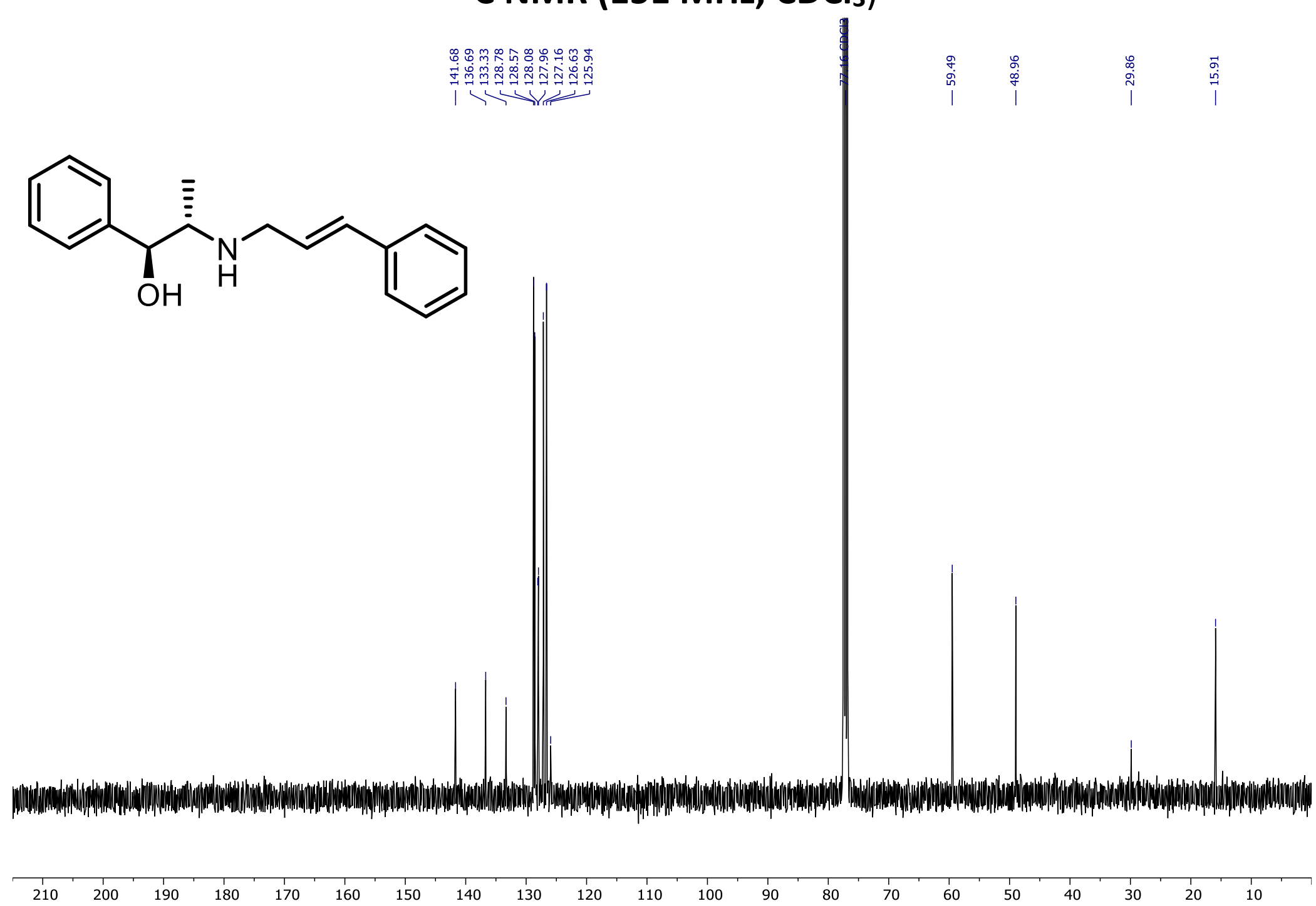

(*R*)-5-Phenyl-*N*-(3-phenyl-3-(*o*-tolyloxy)propyl)pentan-1-amine (1.05)

^{1}H NMR (400 MHz, $CDCl_3$)

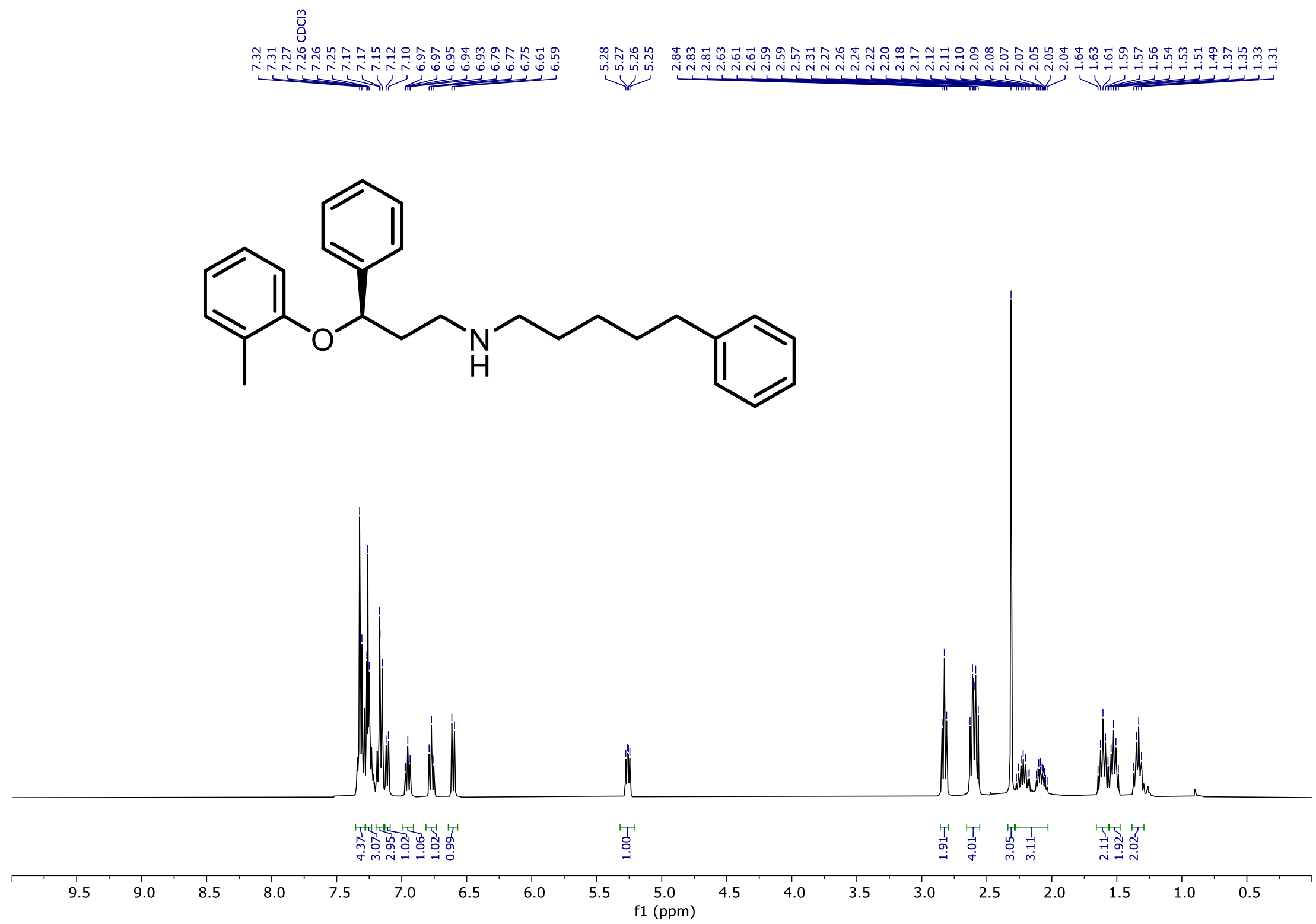

^{13}C NMR (151 MHz, $CDCl_3$)

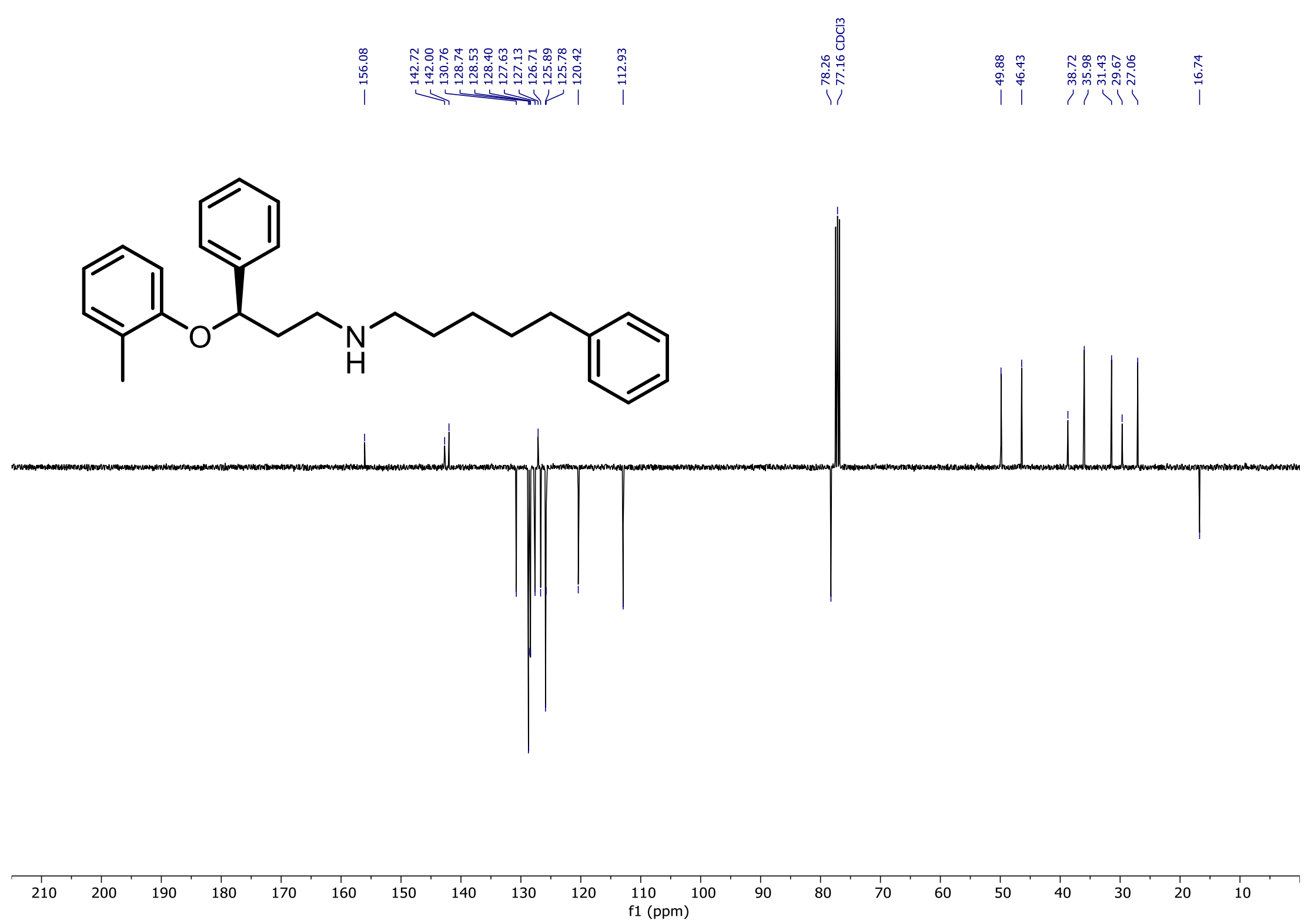

(*E*)-*N*-(3-(2-methoxyphenoxy)-3-phenylpropyl)-3-(4-(trifluoromethyl)phenyl)prop-2-en-1-amine (1.06)

^{1}H NMR (600 MHz, $CDCl_3$)

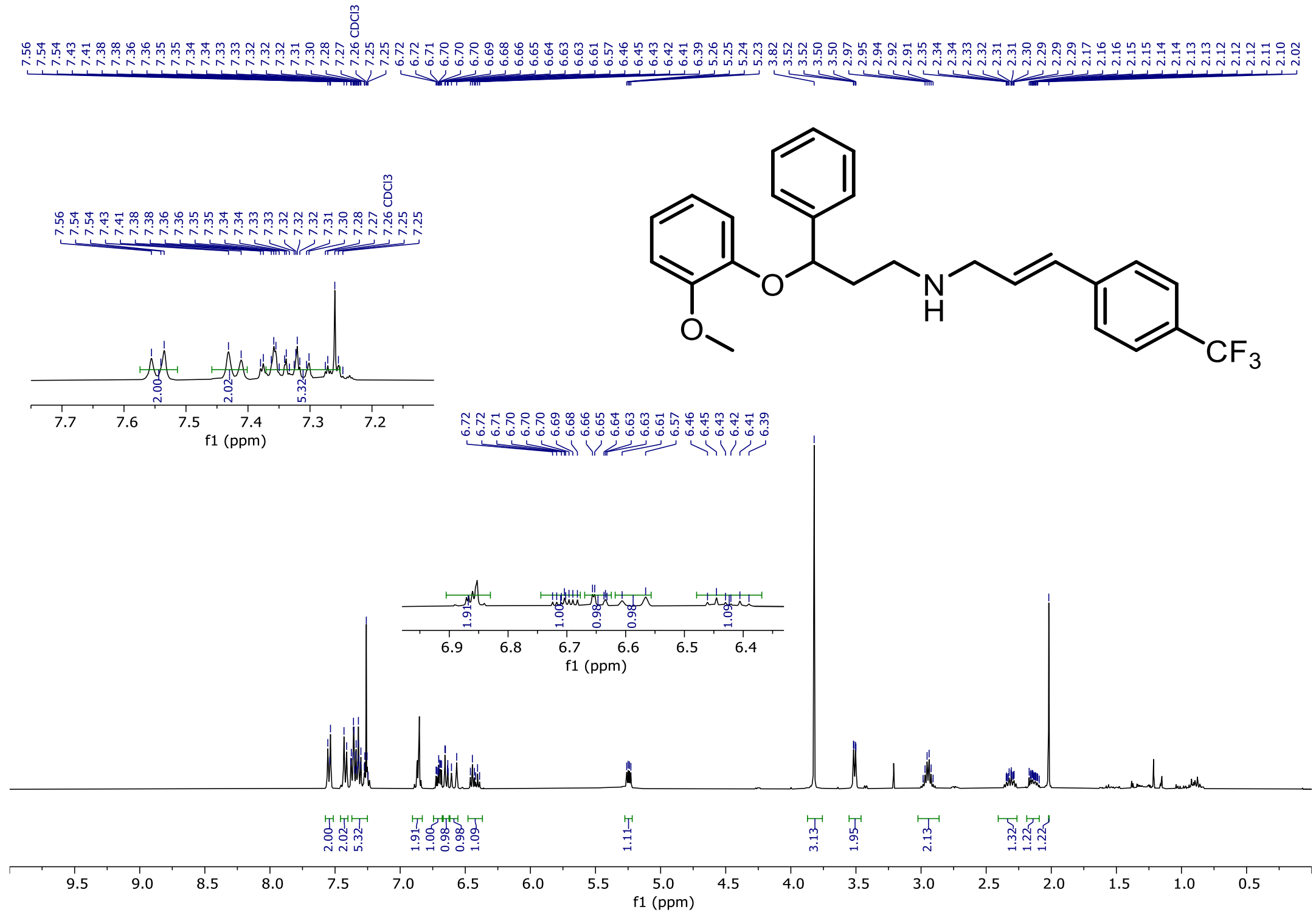

^{13}C NMR (151 MHz, $CDCl_3$)

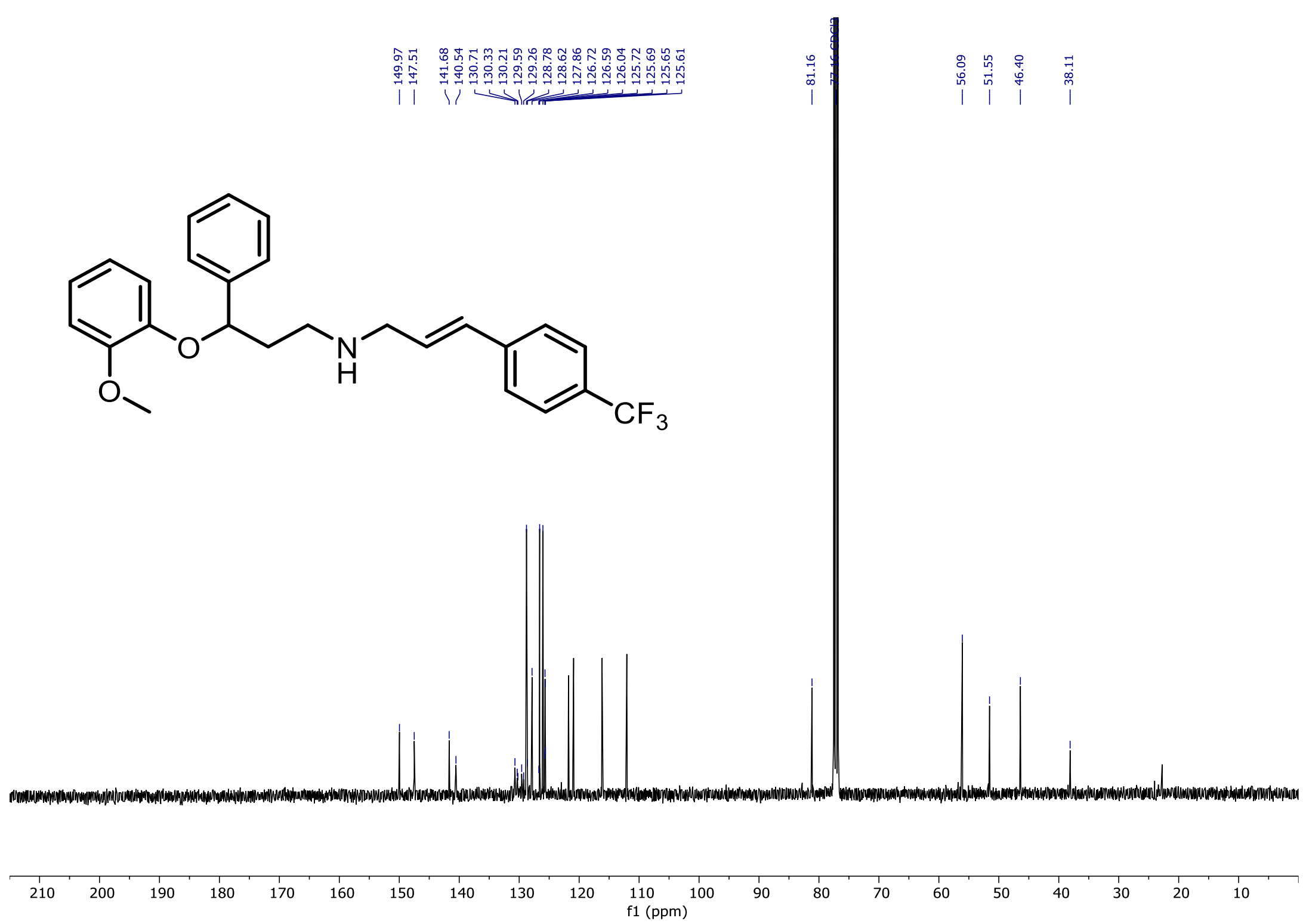

^{19}F NMR (376 MHz, CDCl$_3$)

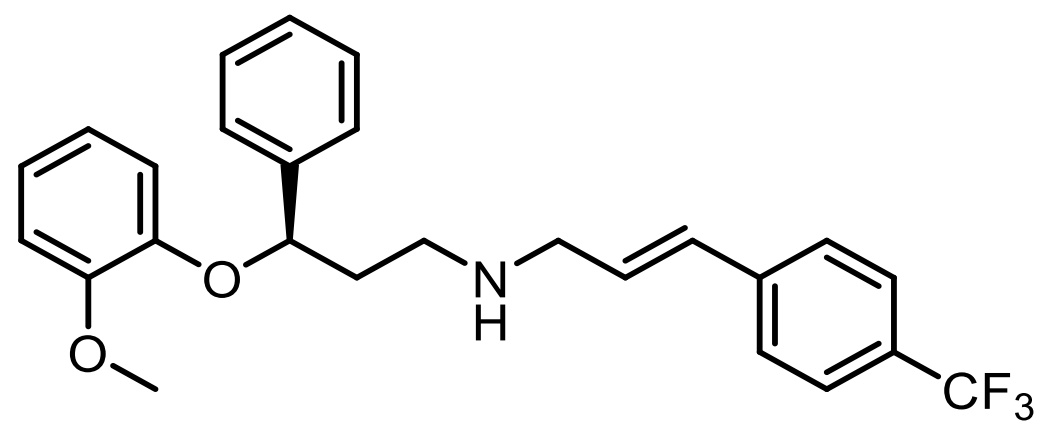

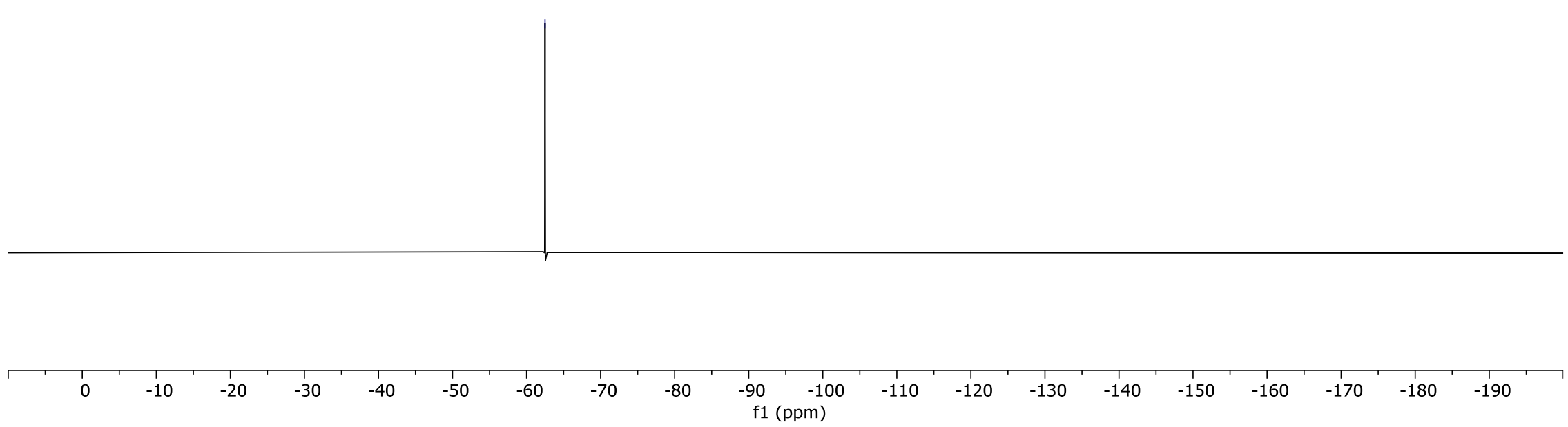

_N_-Cinnamyl-4-(3,4-dichlorophenyl)-1,2,3,4-tetrahydronaphthalen-1-amine (1.07)

^{1}H NMR (400 MHz, $CDCl_3$)

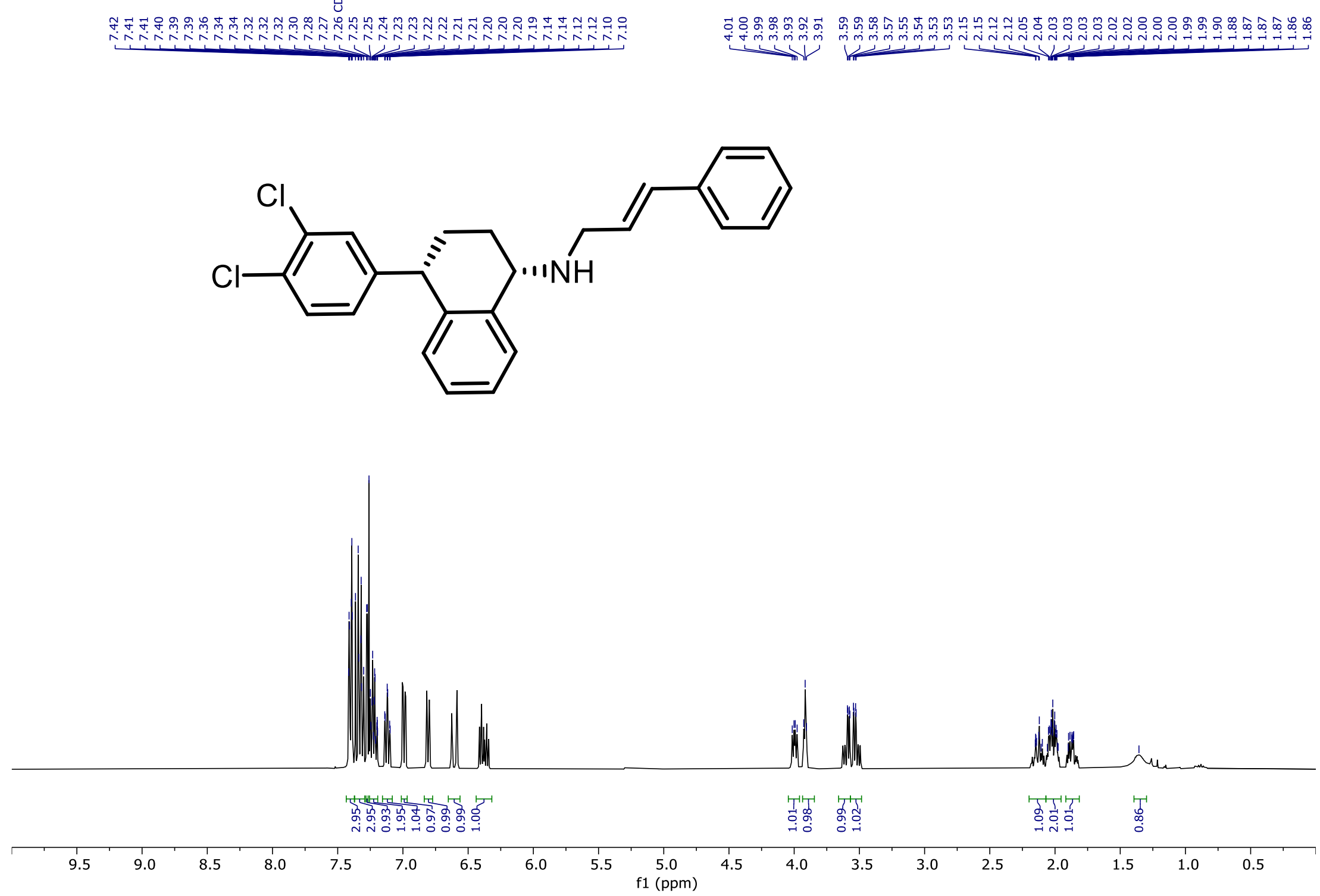

^{13}C NMR (101 MHz, $CDCl_3$)

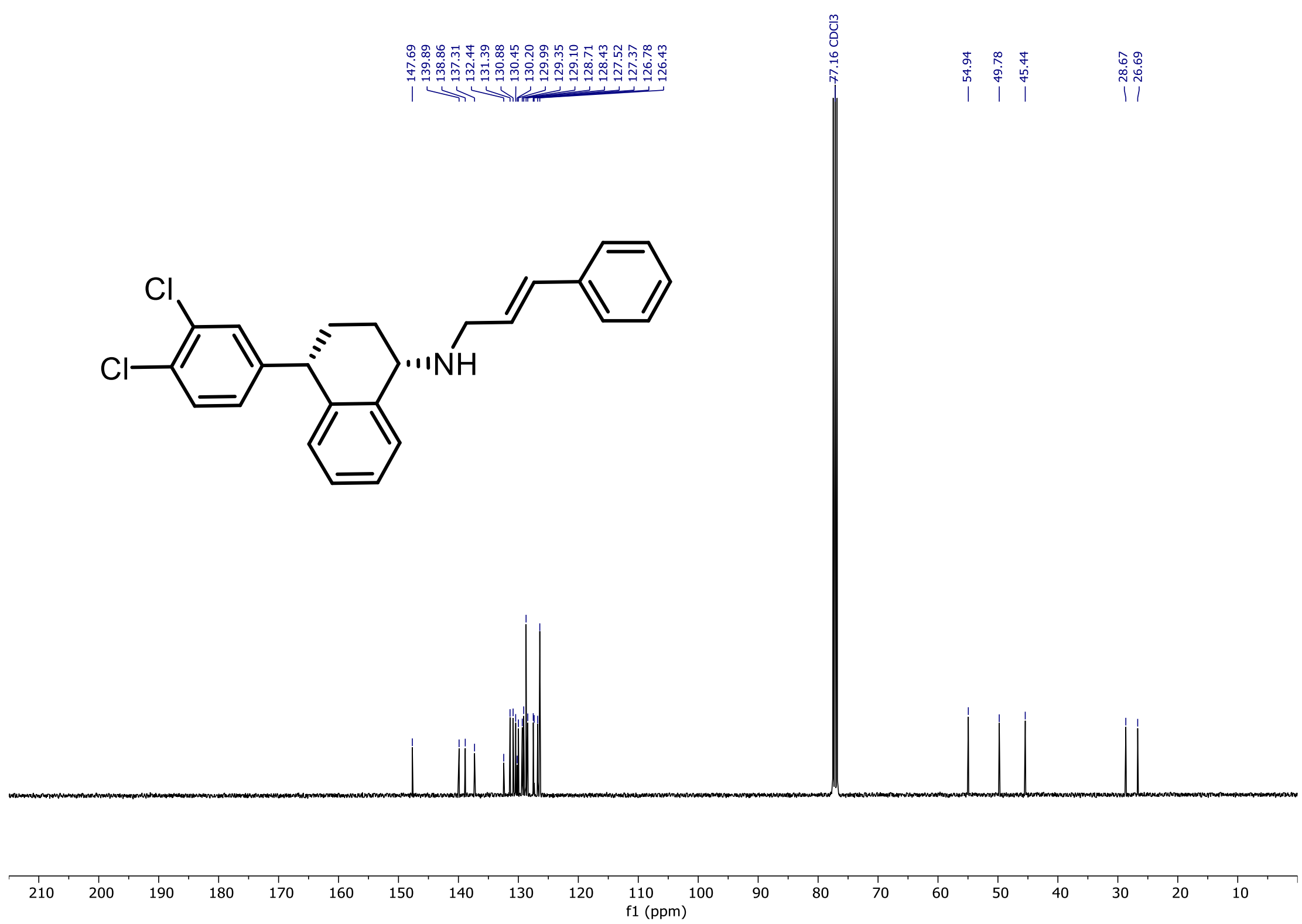

_N_-((5-(2-Fluorophenyl)-1-(pyridin-3-ylsulfonyl)-1_H_-pyrrol-3-yl)methyl)-3-phenylpropan-1-amin (1.08)

^{1}H NMR (600 MHz, $CDCl_3$)

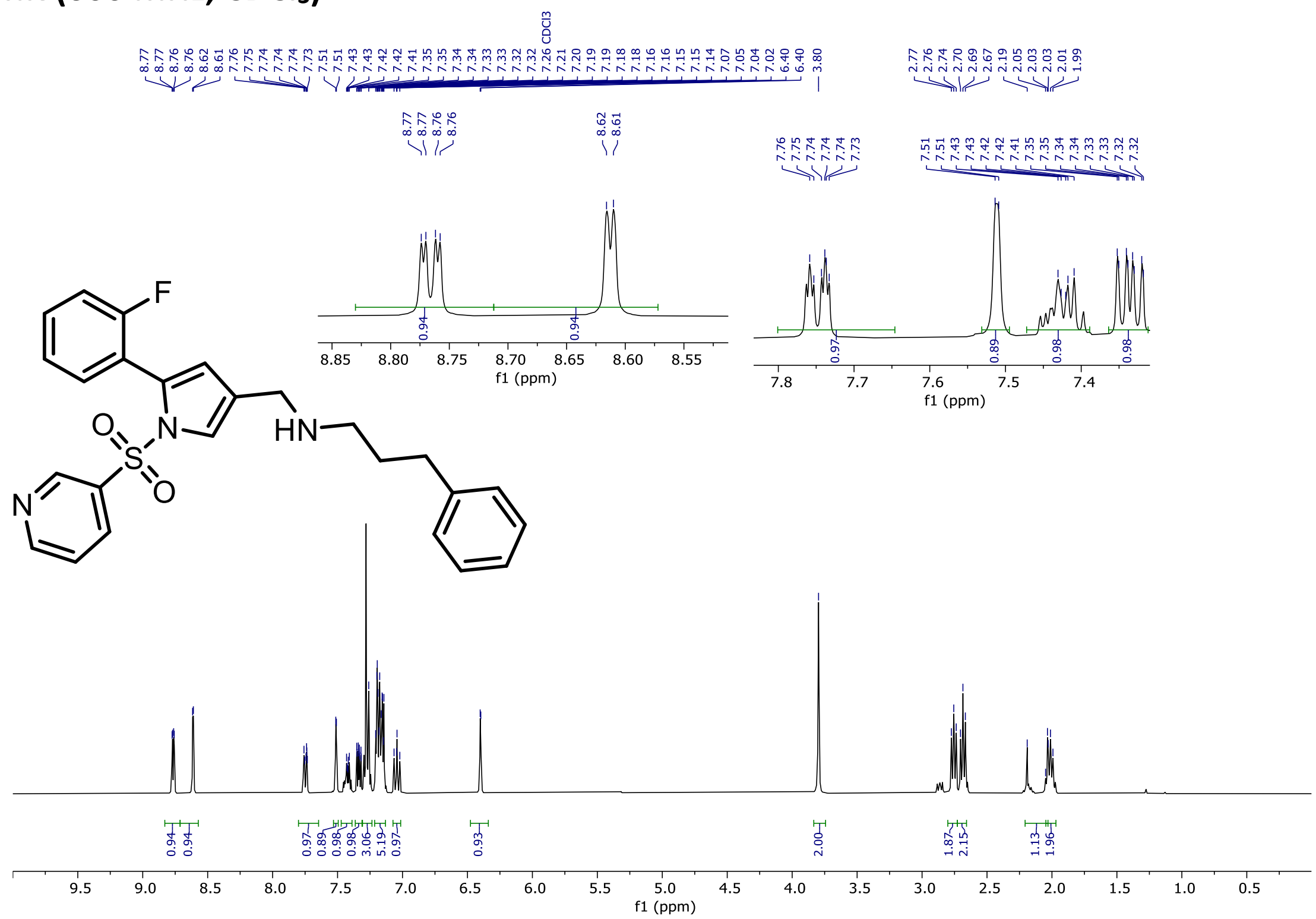

^{13}C NMR (151 MHz, $CDCl_3$)

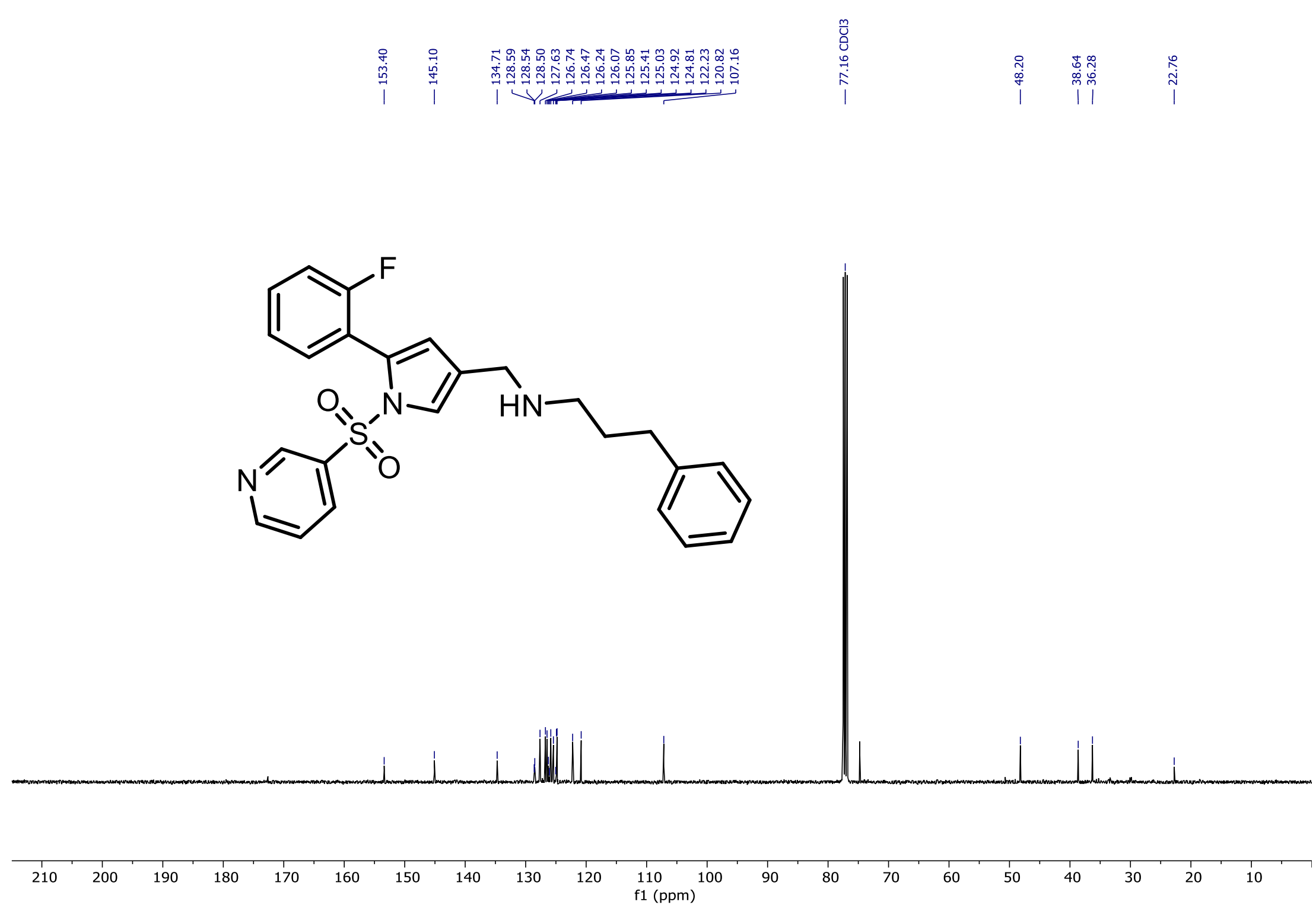

^{19}F NMR (659 MHz, $CDCl_3$)

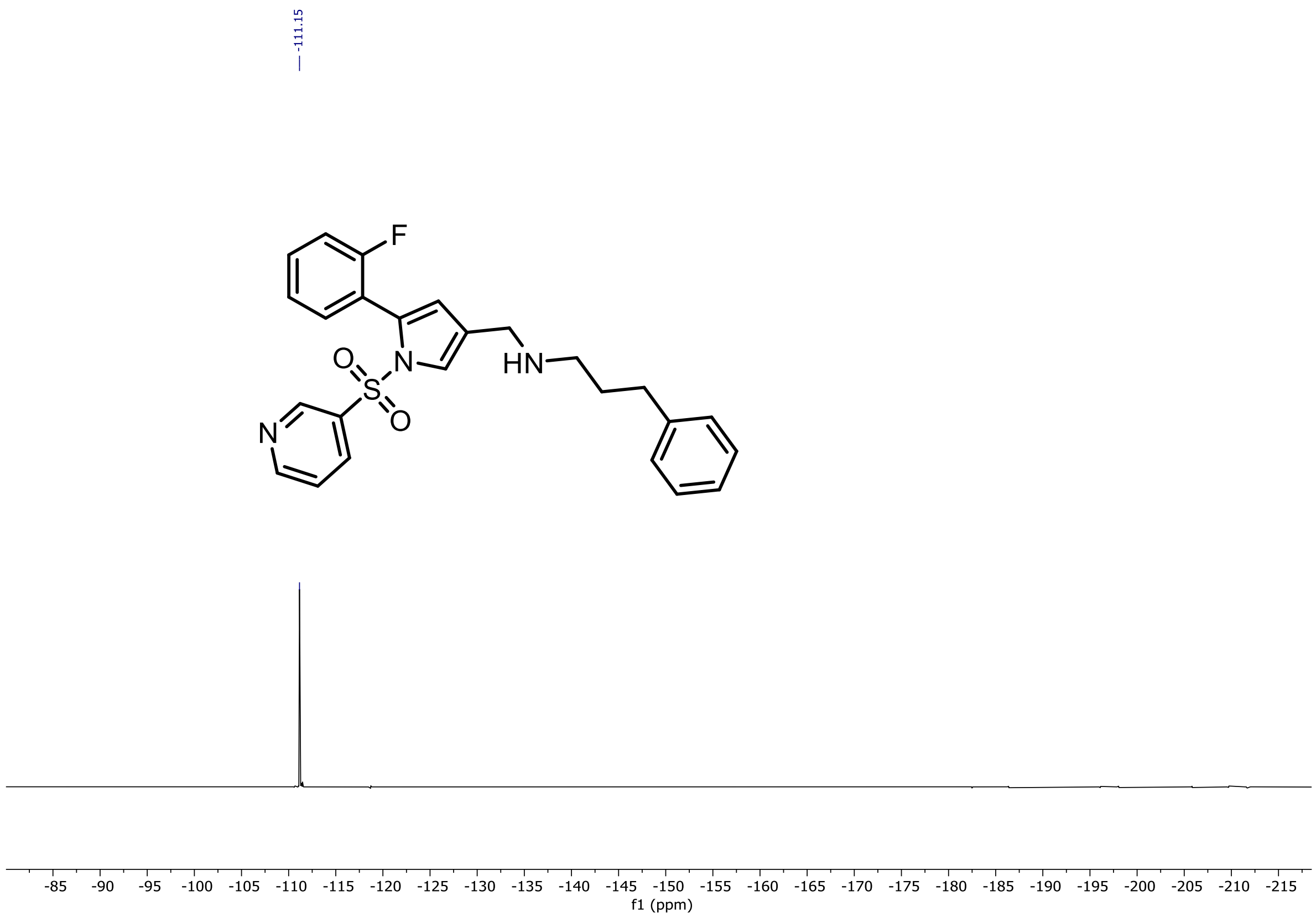

N-(3-(4-Chlorophenyl)-3-(pyridin-2-yl)propyl)-5-phenylpentan-1-amine (1.09)

^{13}C NMR (151 MHz, CDCl$_3$)

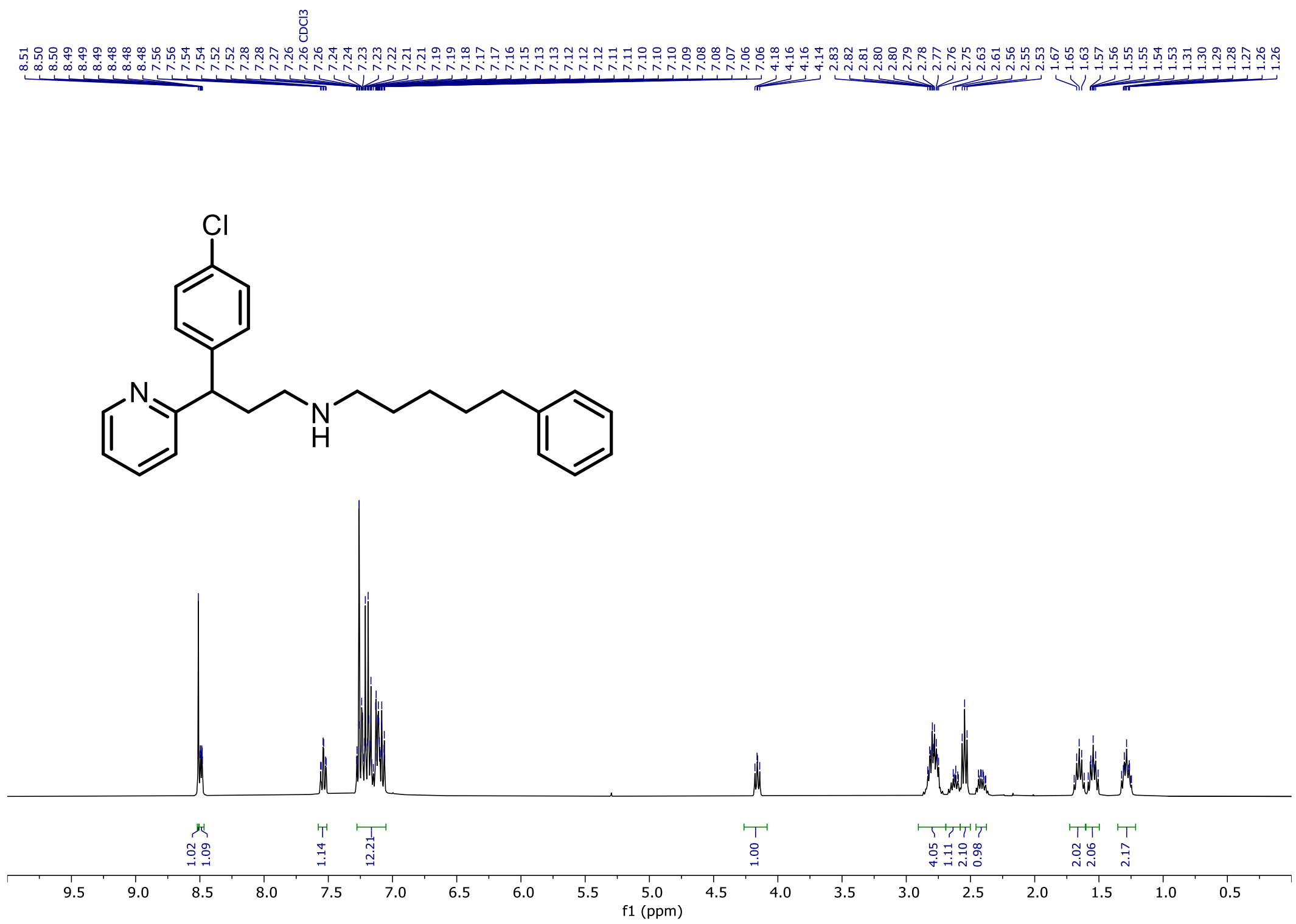

^{13}C NMR (126 MHz, CDCl$_3$)

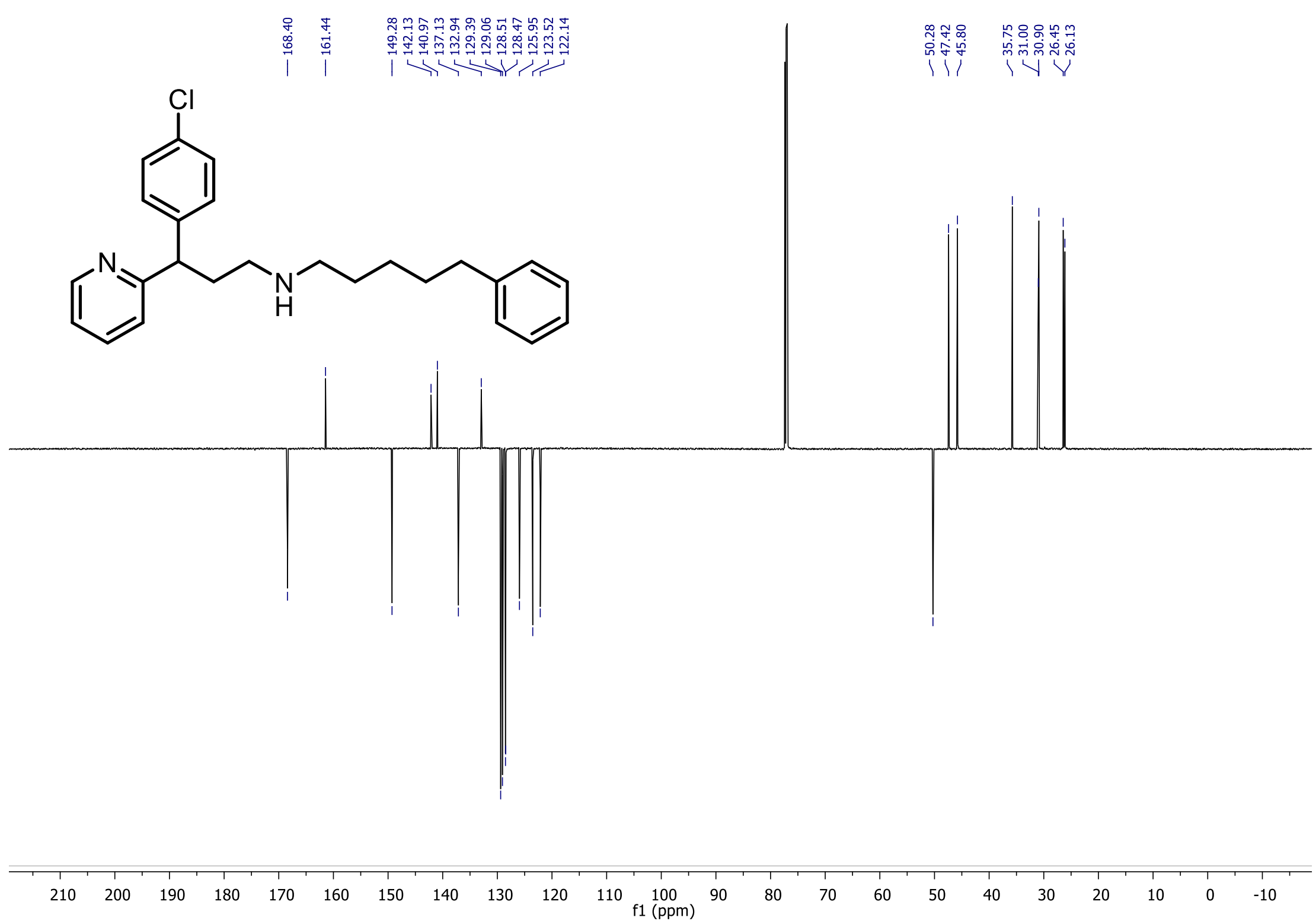

1-(4-Fluorophenyl)-1-(3-((5-phenylpentyl)amino)propyl)-1,3-dihydroisobenzofuran-5-carbonitrile (1.10)

^{1}H NMR (600 MHz, $CDCl_3$)

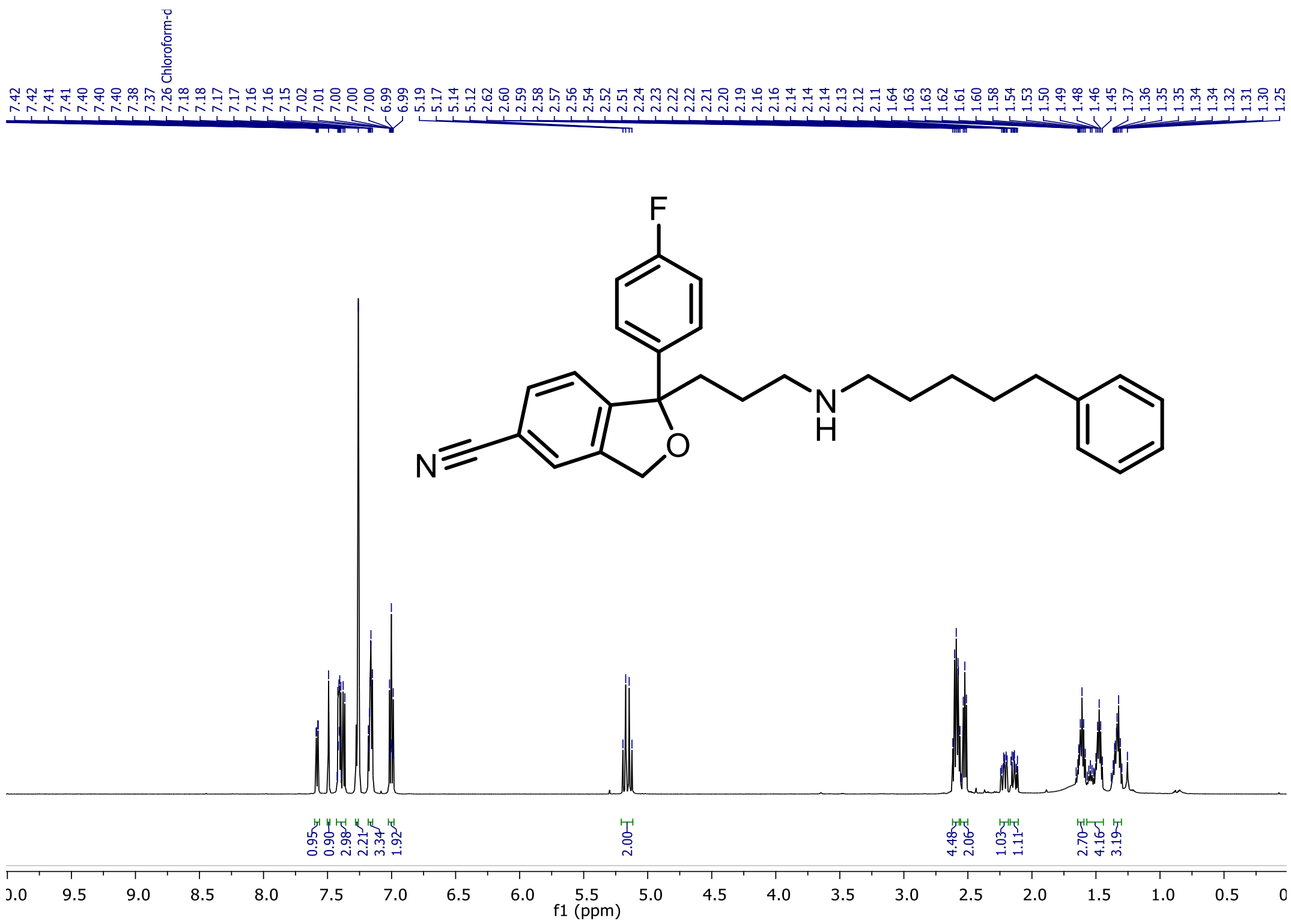

^{13}C NMR (151 MHz, $CDCl_3$)

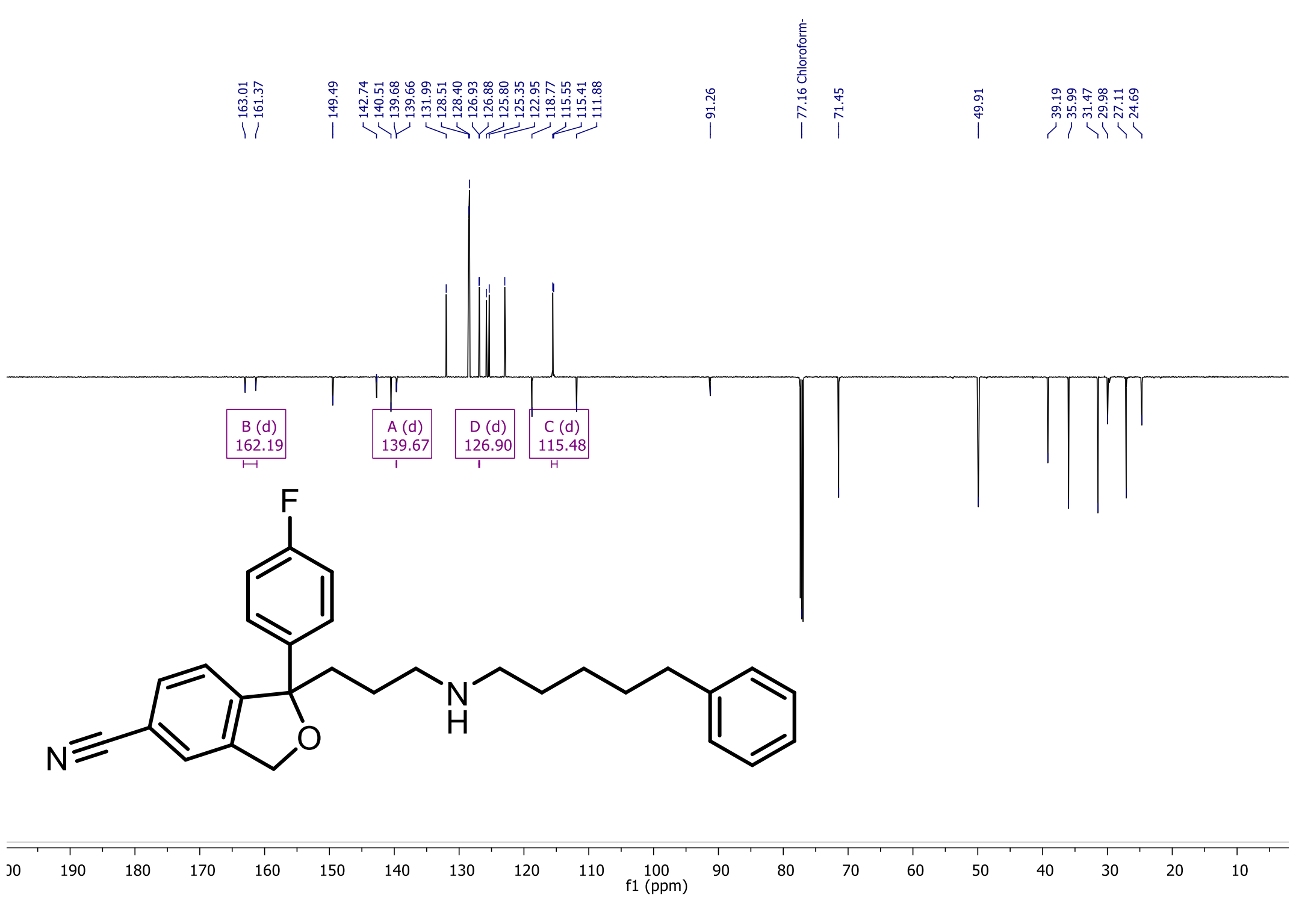

^{19}F NMR (377 MHz, $CDCl_3$)

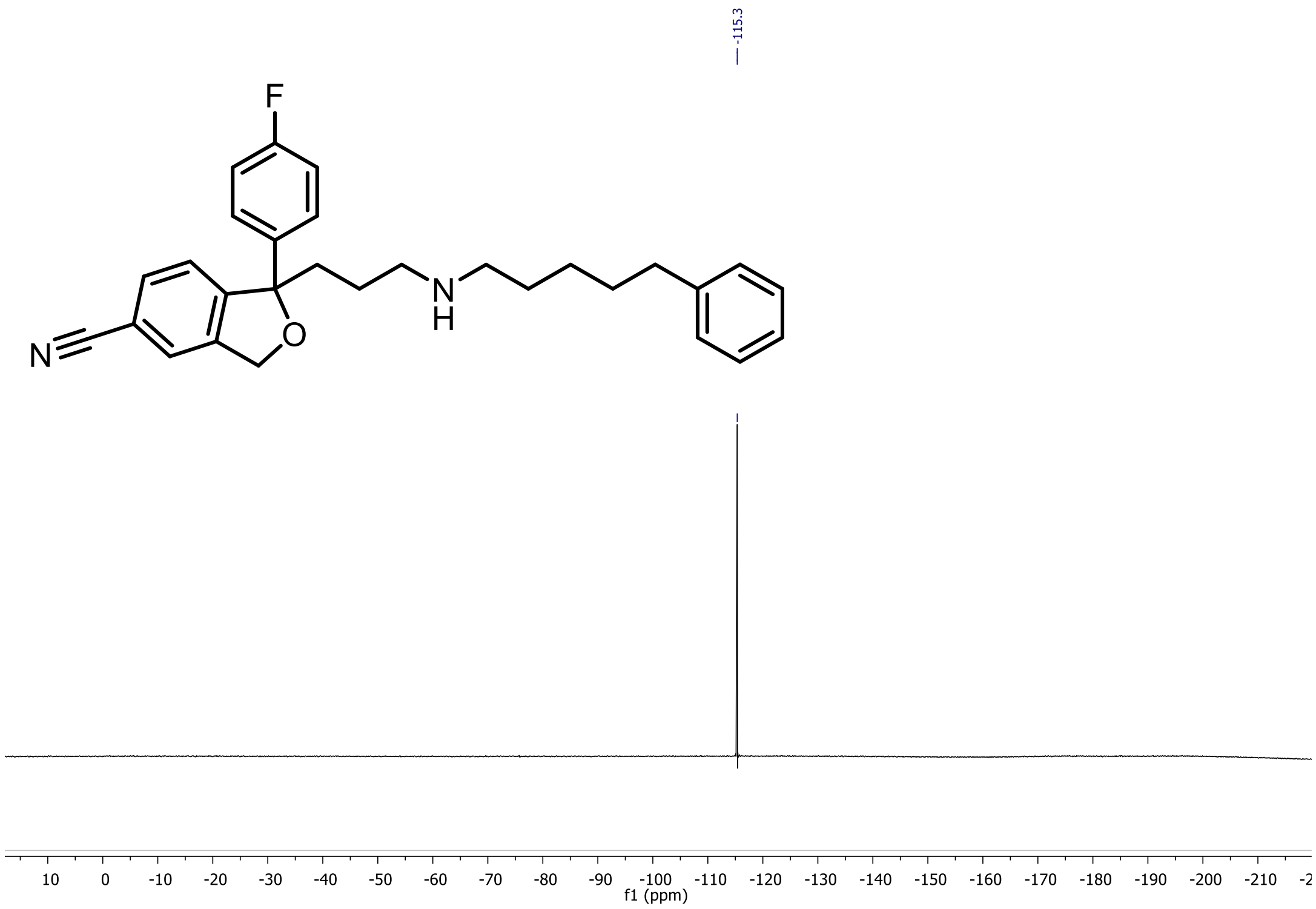

***N*-(3-((1-Benzyl-1H-indazol-3-yl)oxy)propyl)-5-phenylpentan-1-amine (1.11)**

^{1}H NMR (600 MHz, $CDCl_3$)

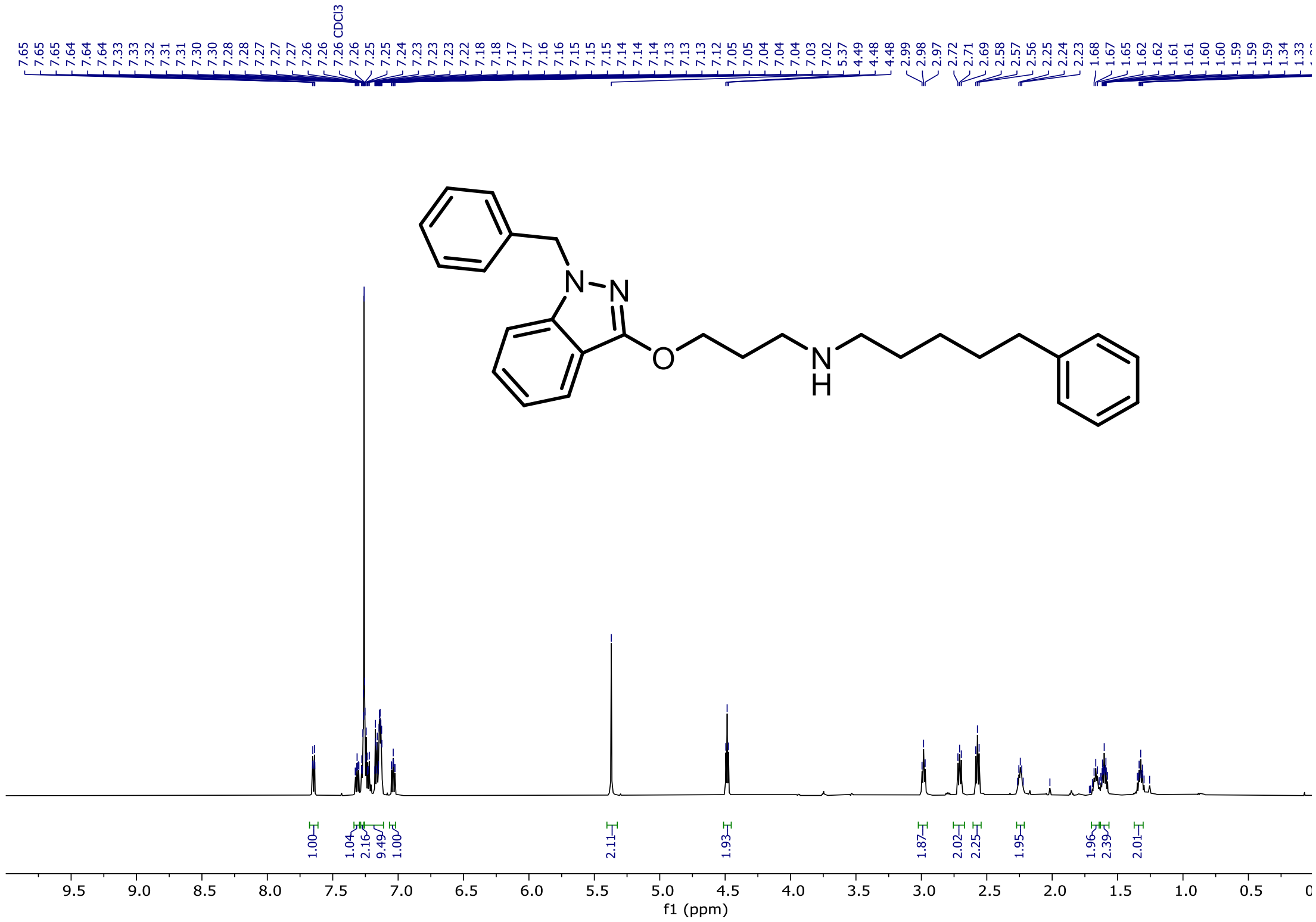

^{13}C NMR (151 MHz, $CDCl_3$)

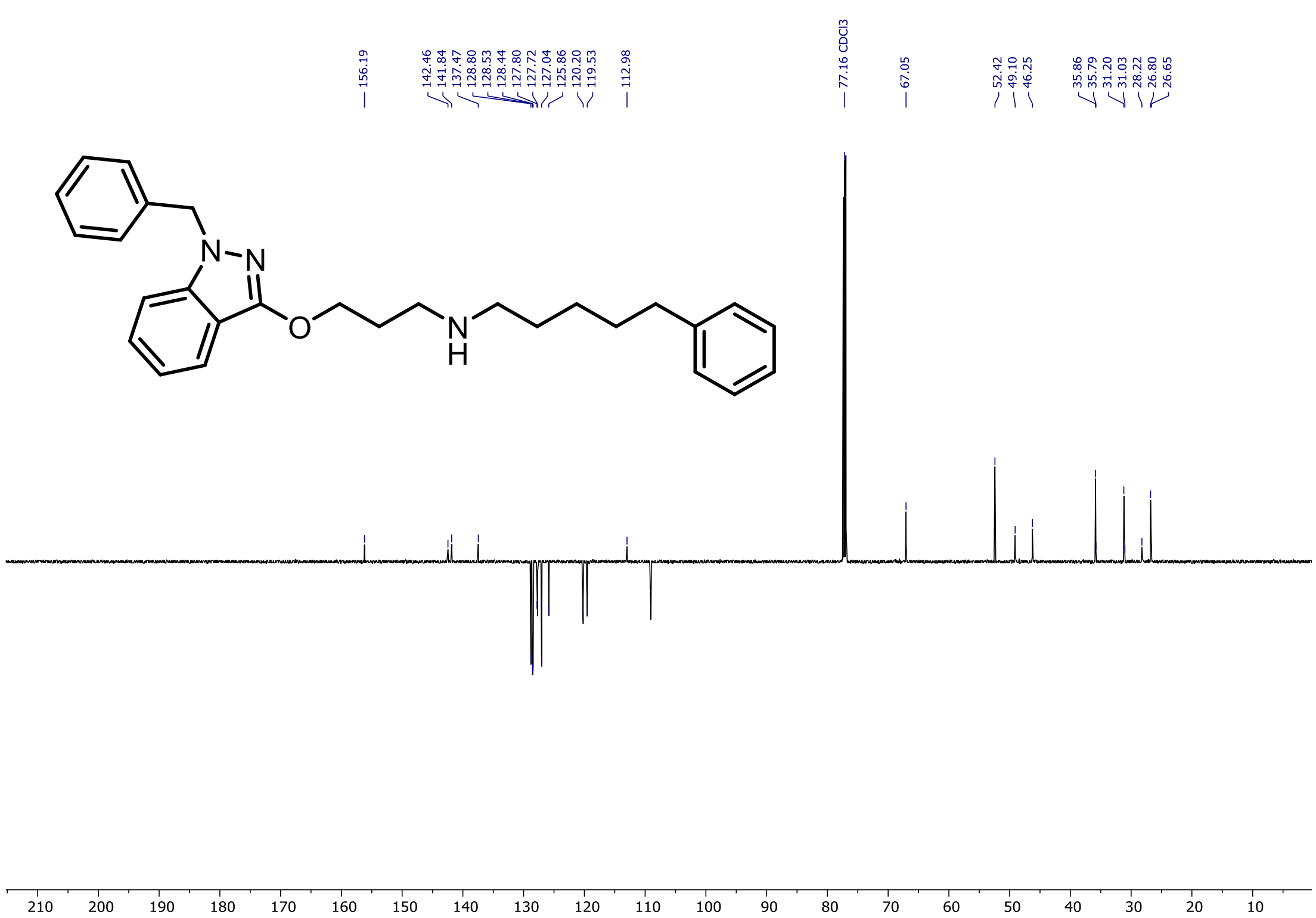

2,6-Dimethyl-*N*-(5-phenylpentyl)aniline (1.12)

^{1}H NMR (600 MHz, $CDCl_3$)

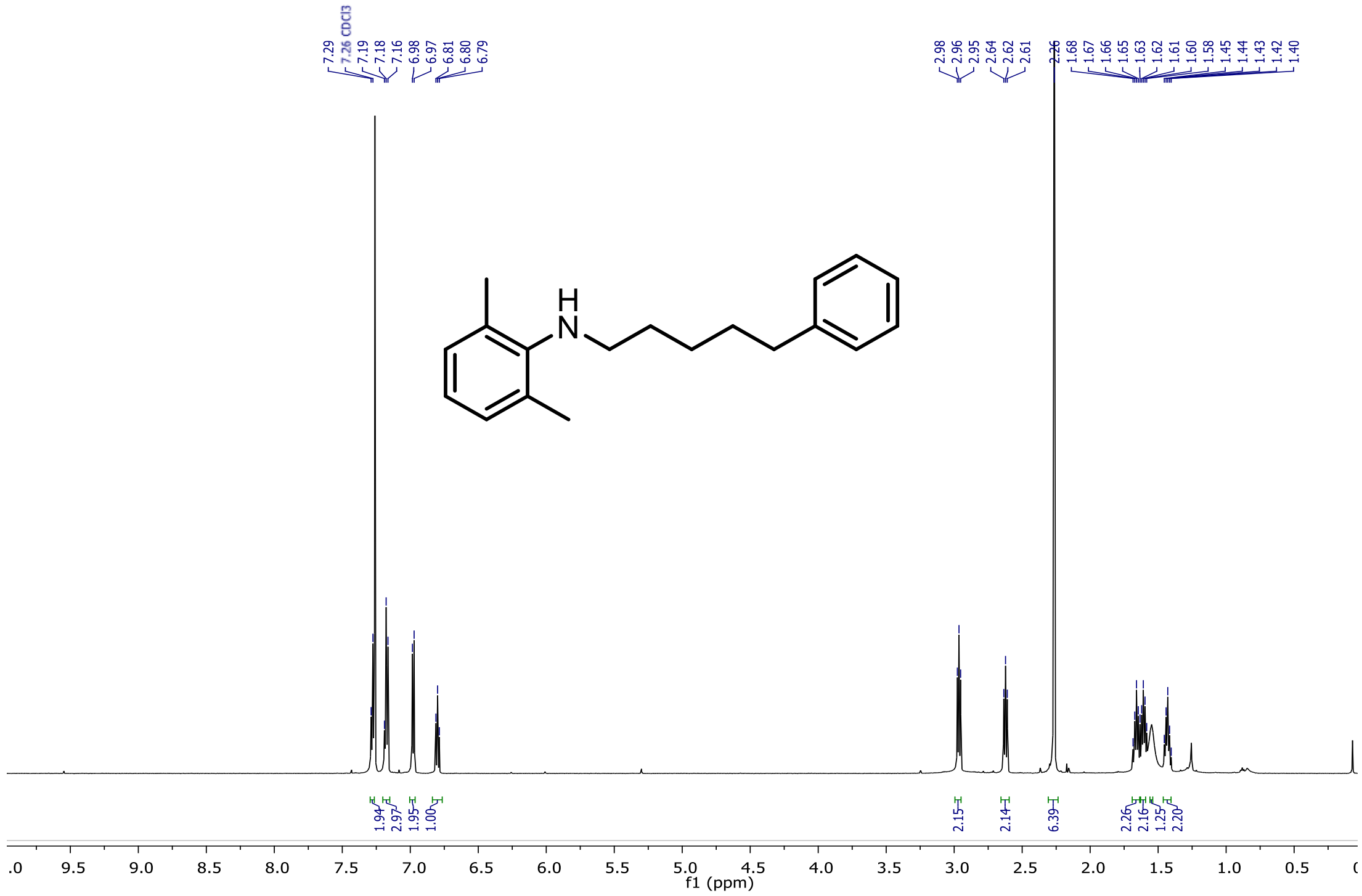

^{13}C NMR (151 MHz, $CDCl_3$)

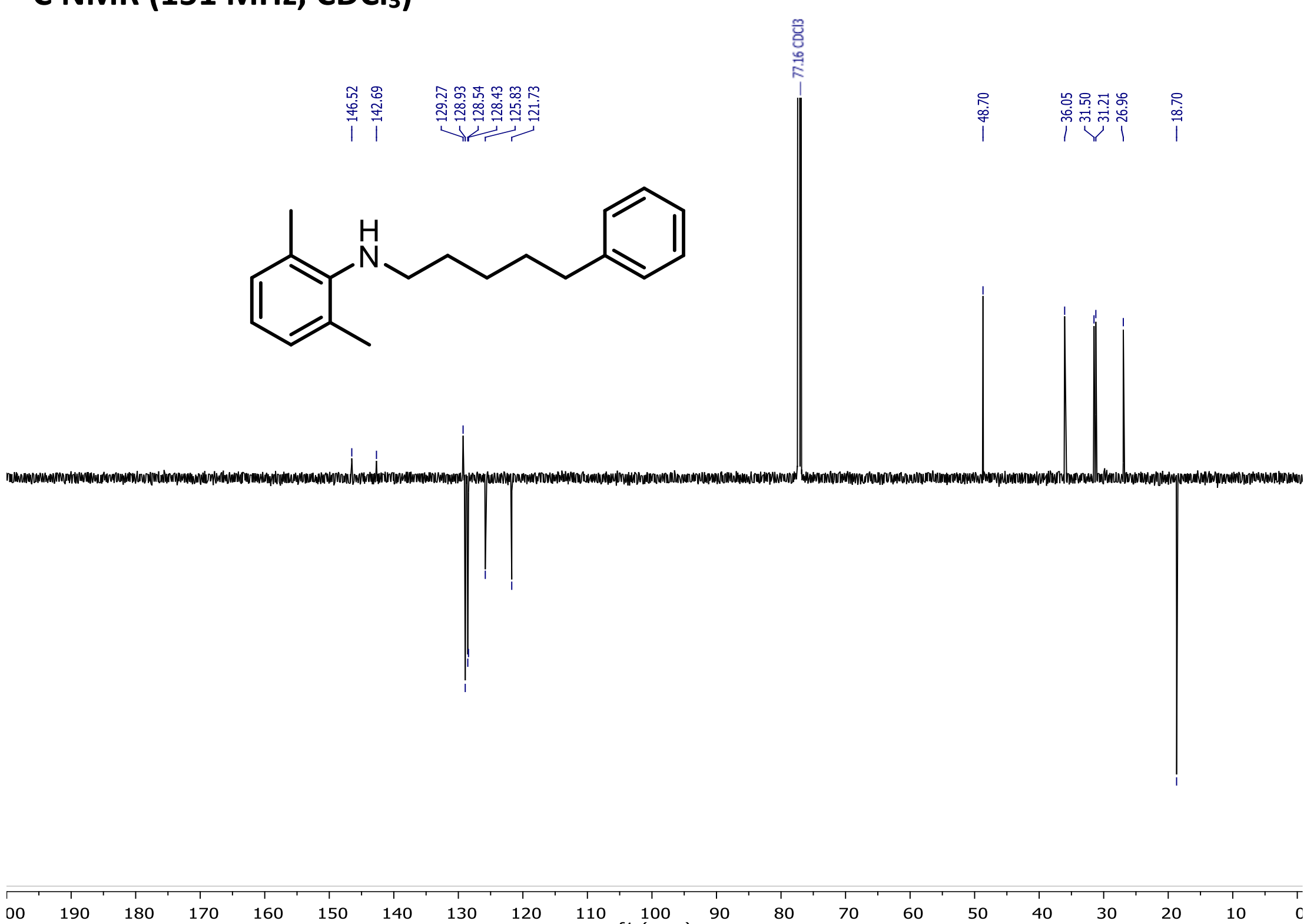

5-Phenyl-*N*-(1-phenylethyl)pentan-1-amine (1.13)

^{1}H NMR (400 MHz, $CDCl_3$)

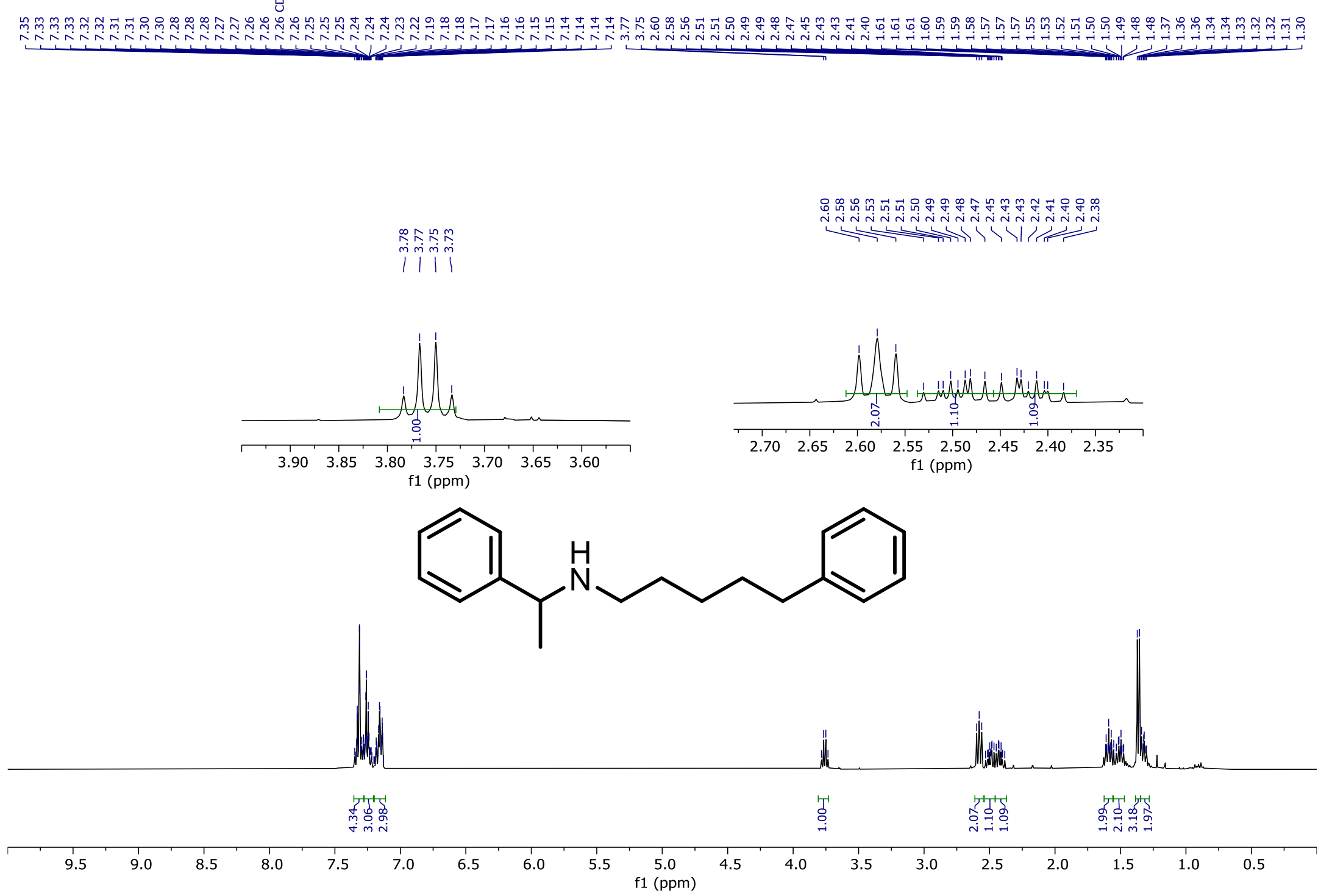

^{13}C NMR (101 MHz, $CDCl_3$)

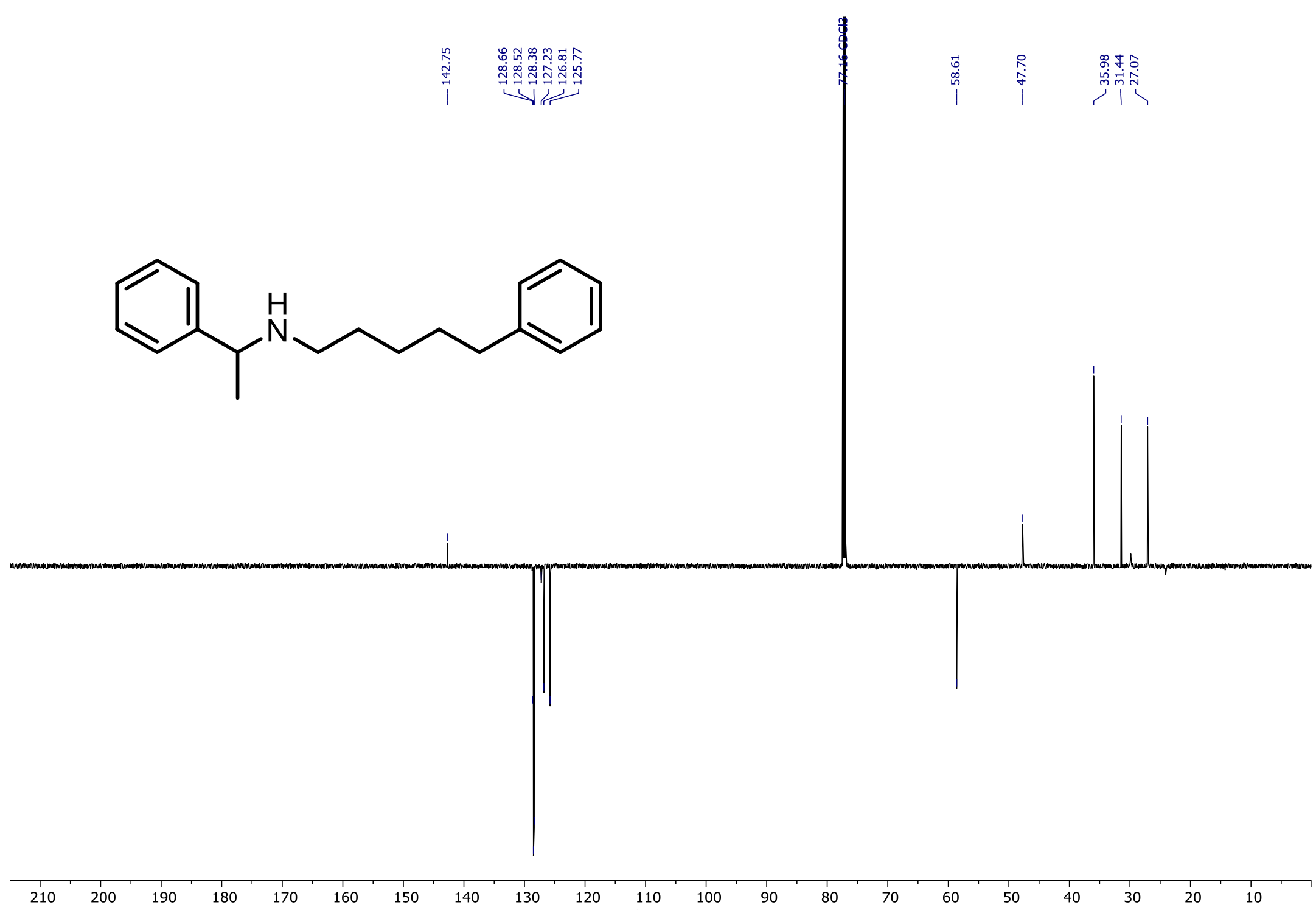

5-Phenyl-*N*-(1-phenylethyl)pentan-1-amine (1.14)

^{1}H NMR (400 MHz, $CDCl_3$)

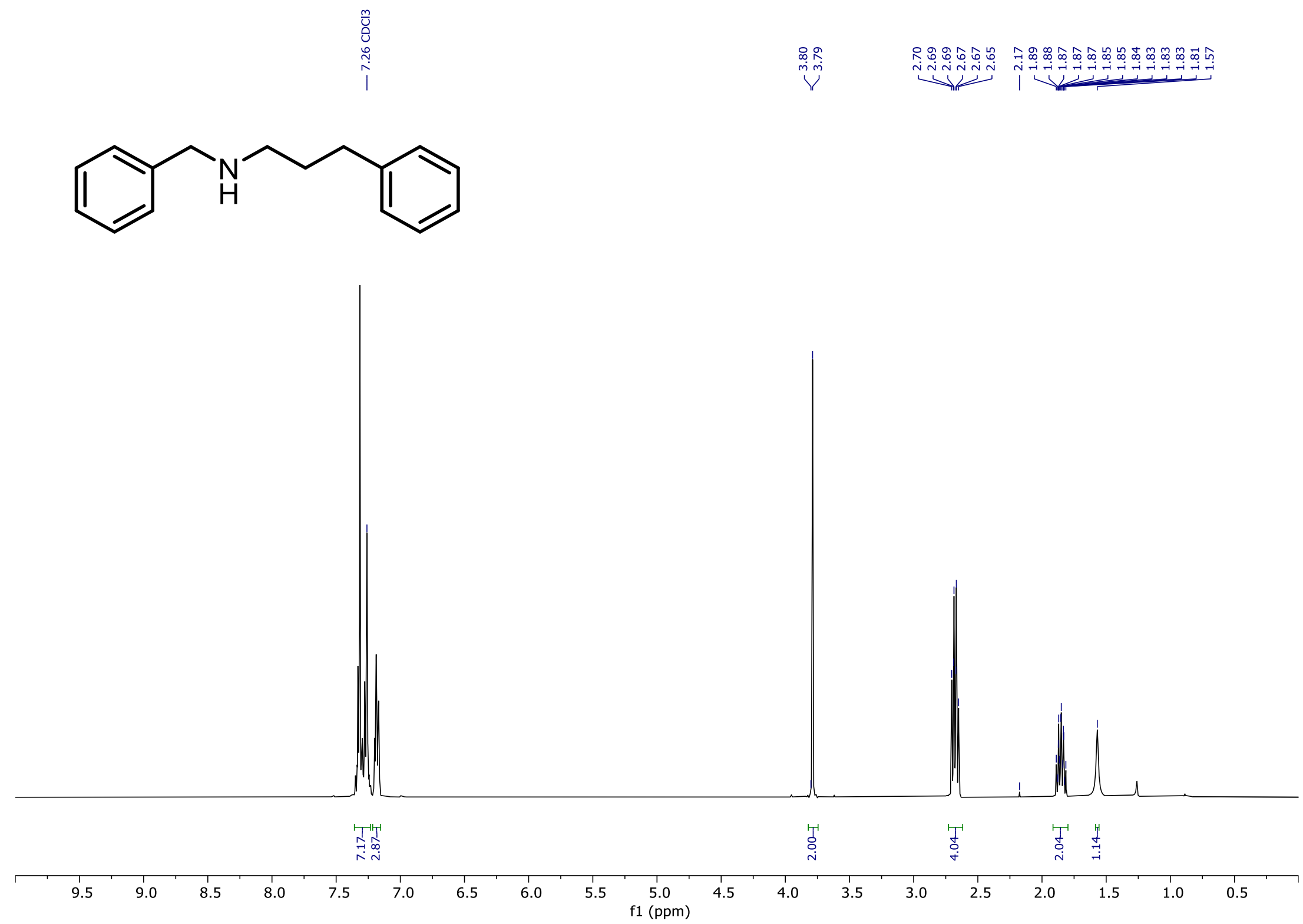

^{13}C NMR (101 MHz, $CDCl_3$)

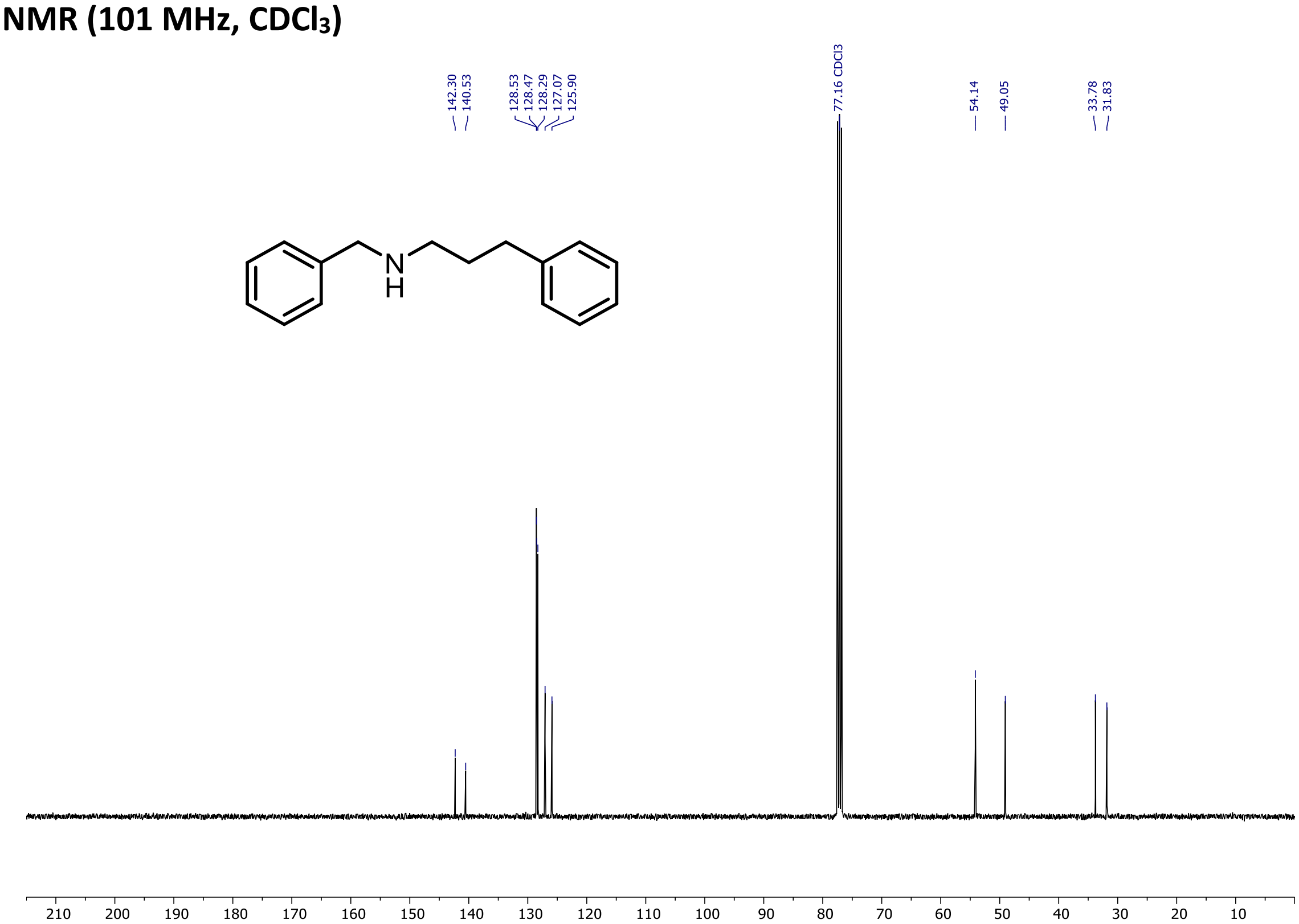

4-Phenyl-*N*-(3-phenyl-3-(4-(trifluoromethyl)phenoxy)propyl)butan-1-amine (1.15a)

^{1}H NMR (600 MHz, $CDCl_3$)

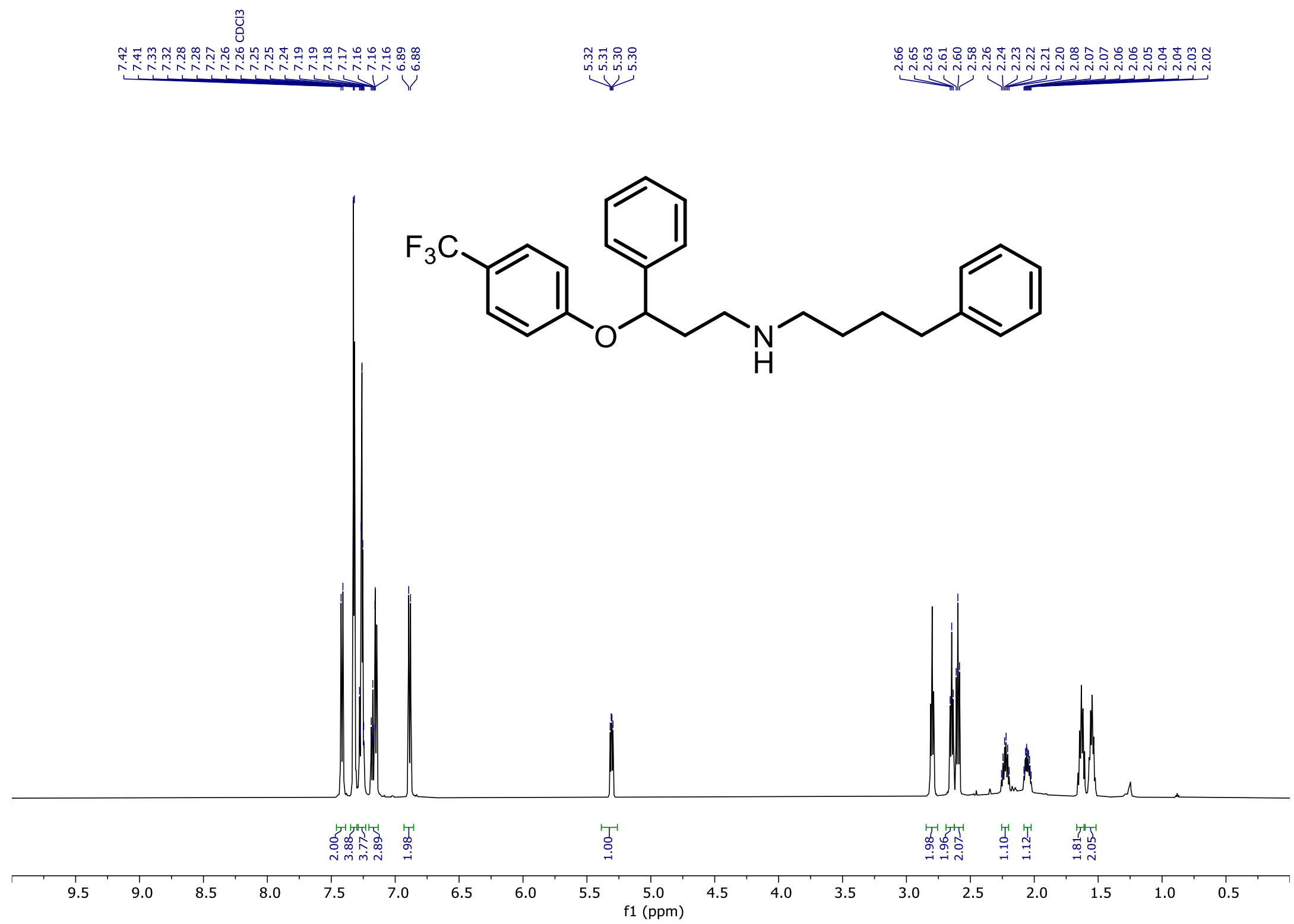

^{13}C NMR (151 MHz, $CDCl_3$)

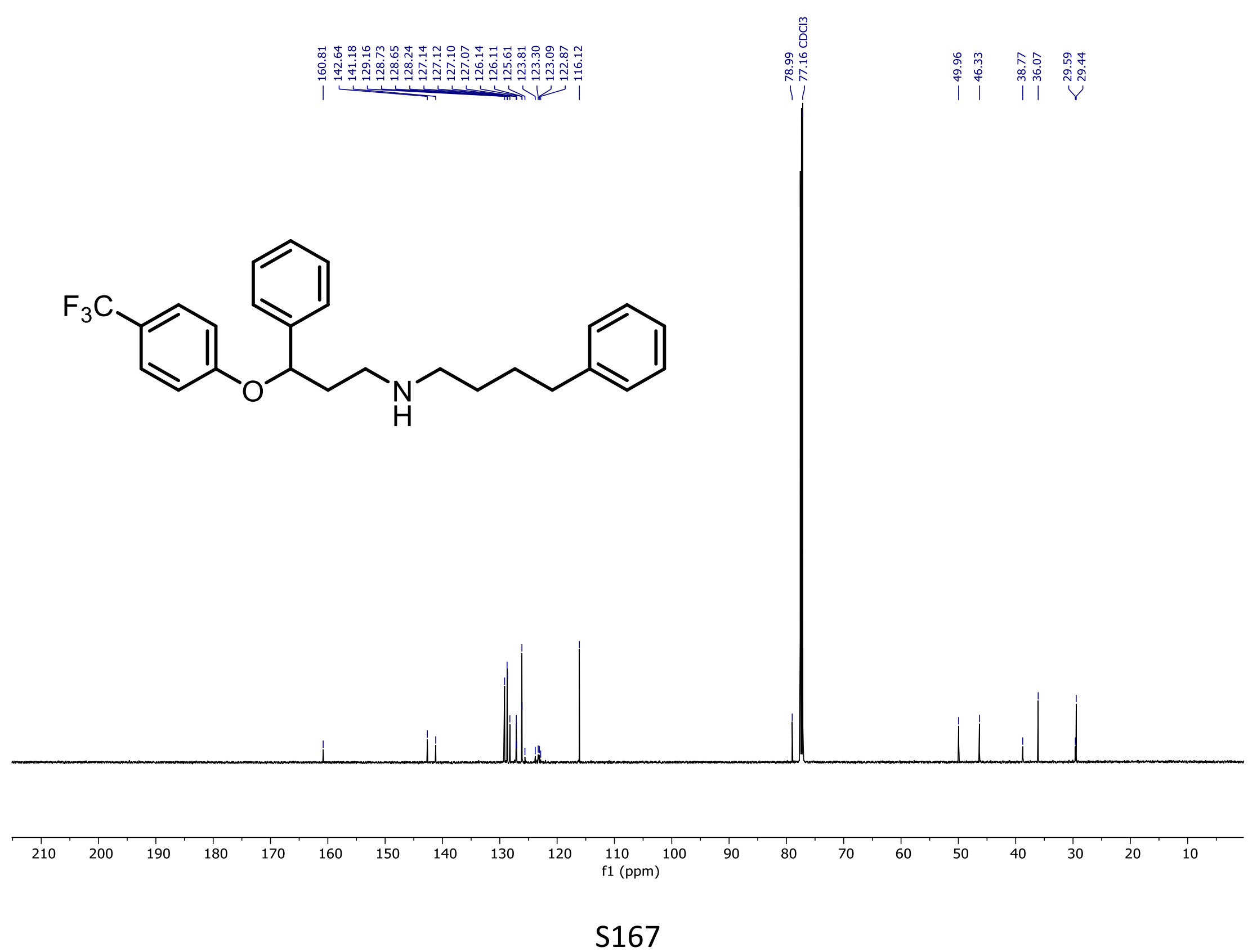

^{19}F NMR (565 MHz, $CDCl_3$)

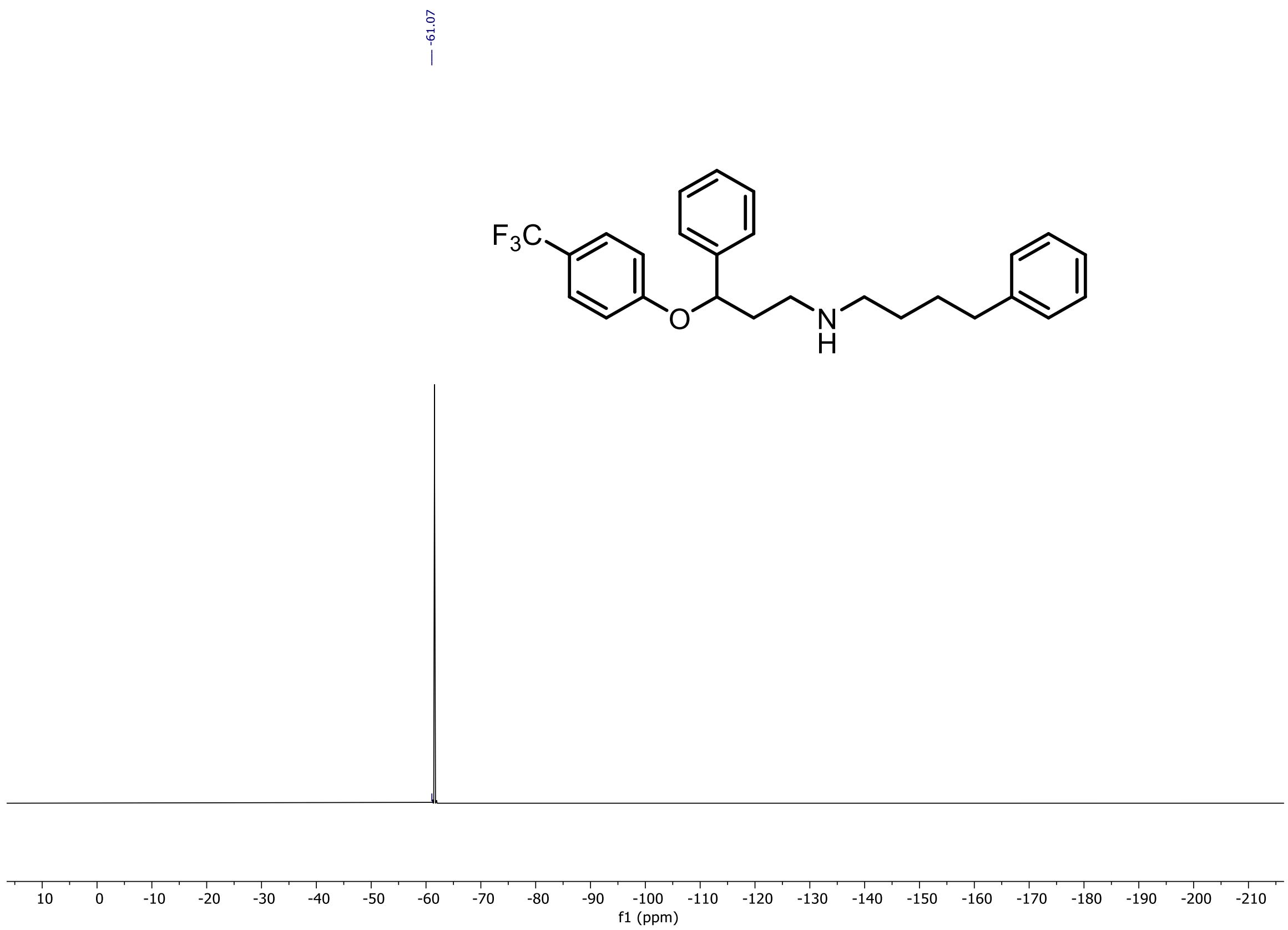

5-Phenyl-*N*-(3-phenyl-3-(4-(trifluoromethyl)phenoxy)propyl)pentan-1-amine (1.15b)

^{1}H NMR (400 MHz, $CDCl_3$)

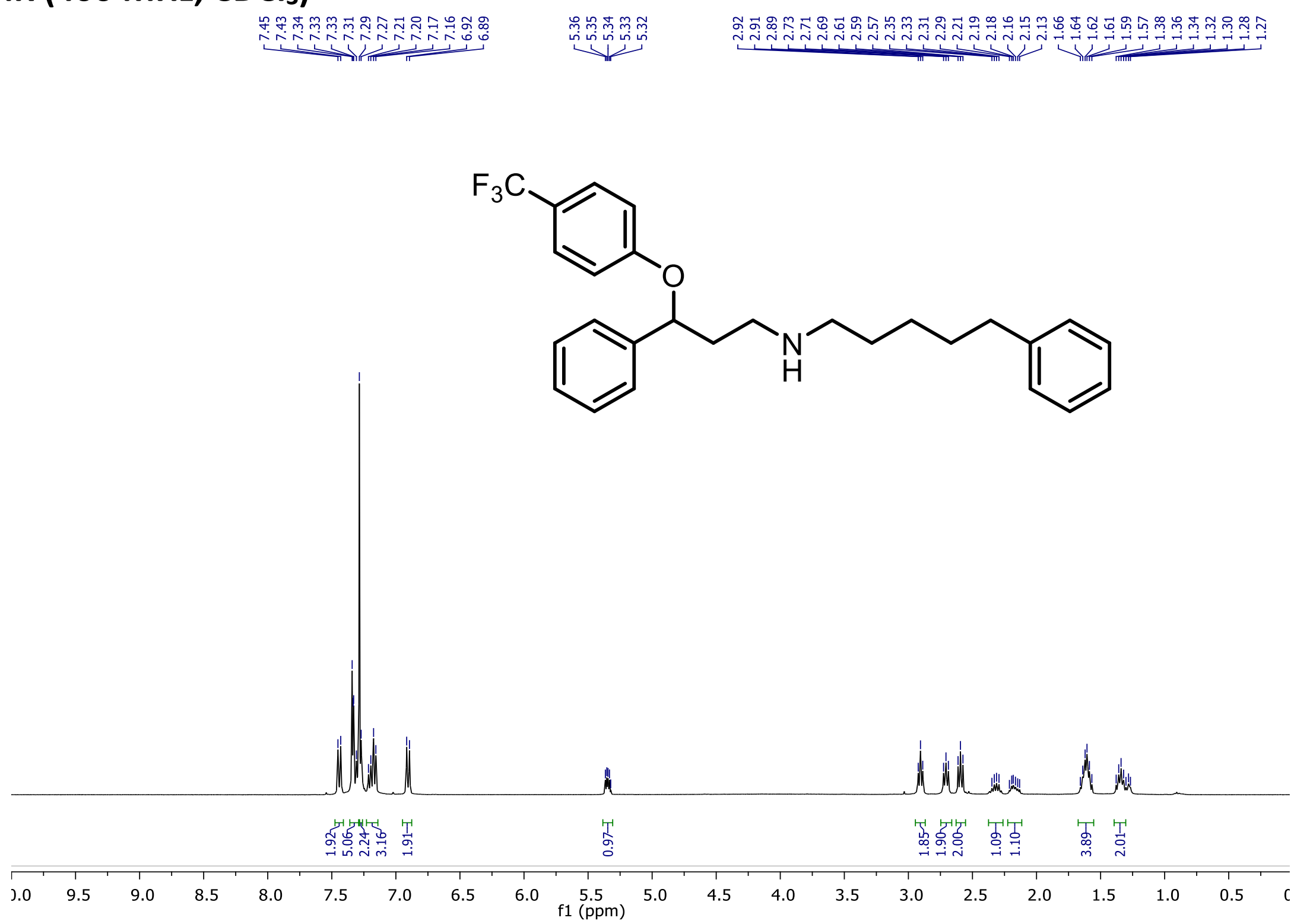

^{13}C NMR (151 MHz, $CDCl_3$)

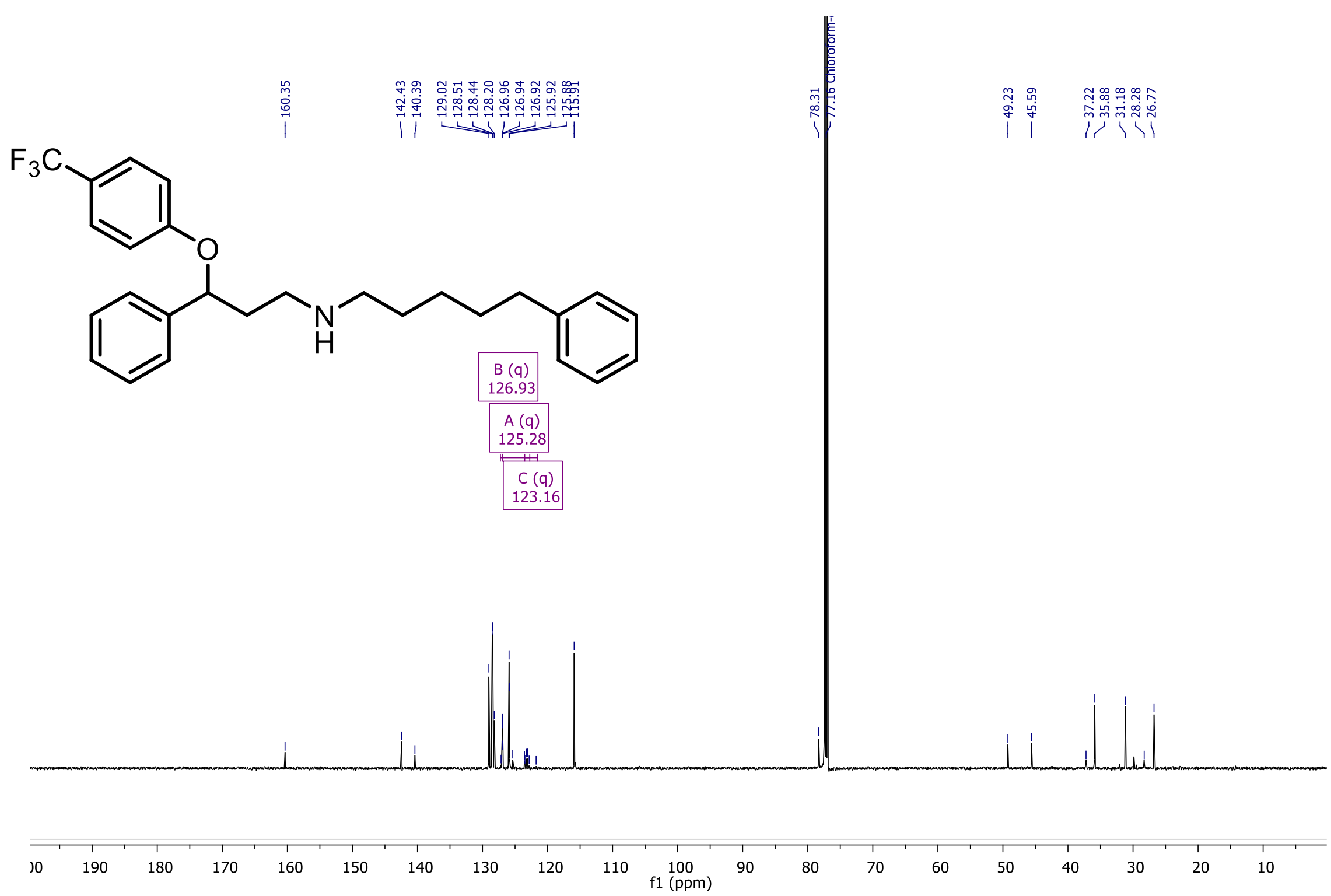

^{19}F NMR (377 MHz, $CDCl_3$)

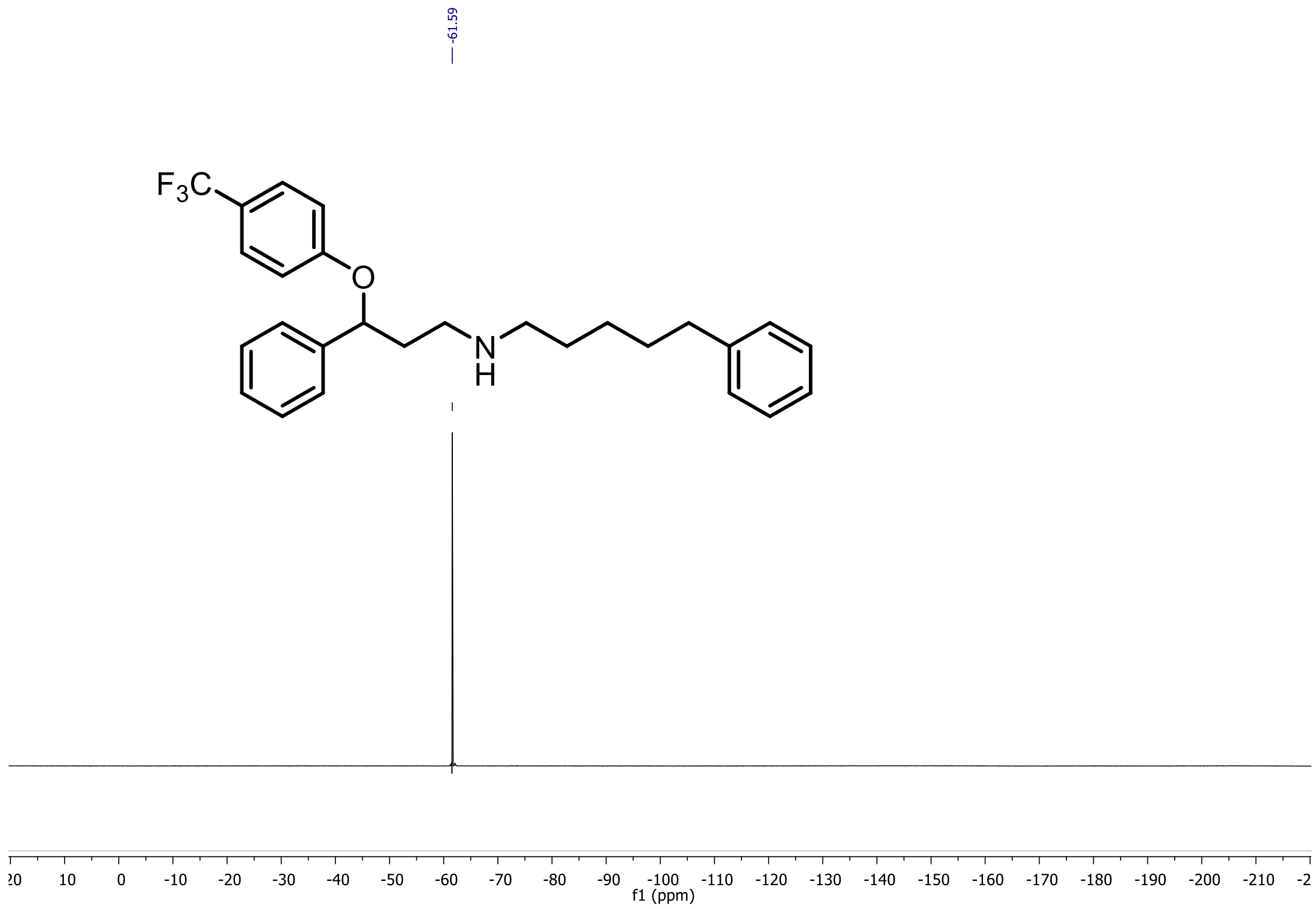

***N*-(2-Cyclohexylethyl)-3-phenyl-3-(4-(trifluoromethyl)phenoxy)propan-1-amine (1.15c)**

^{1}H NMR (600 MHz, $CDCl_3$)

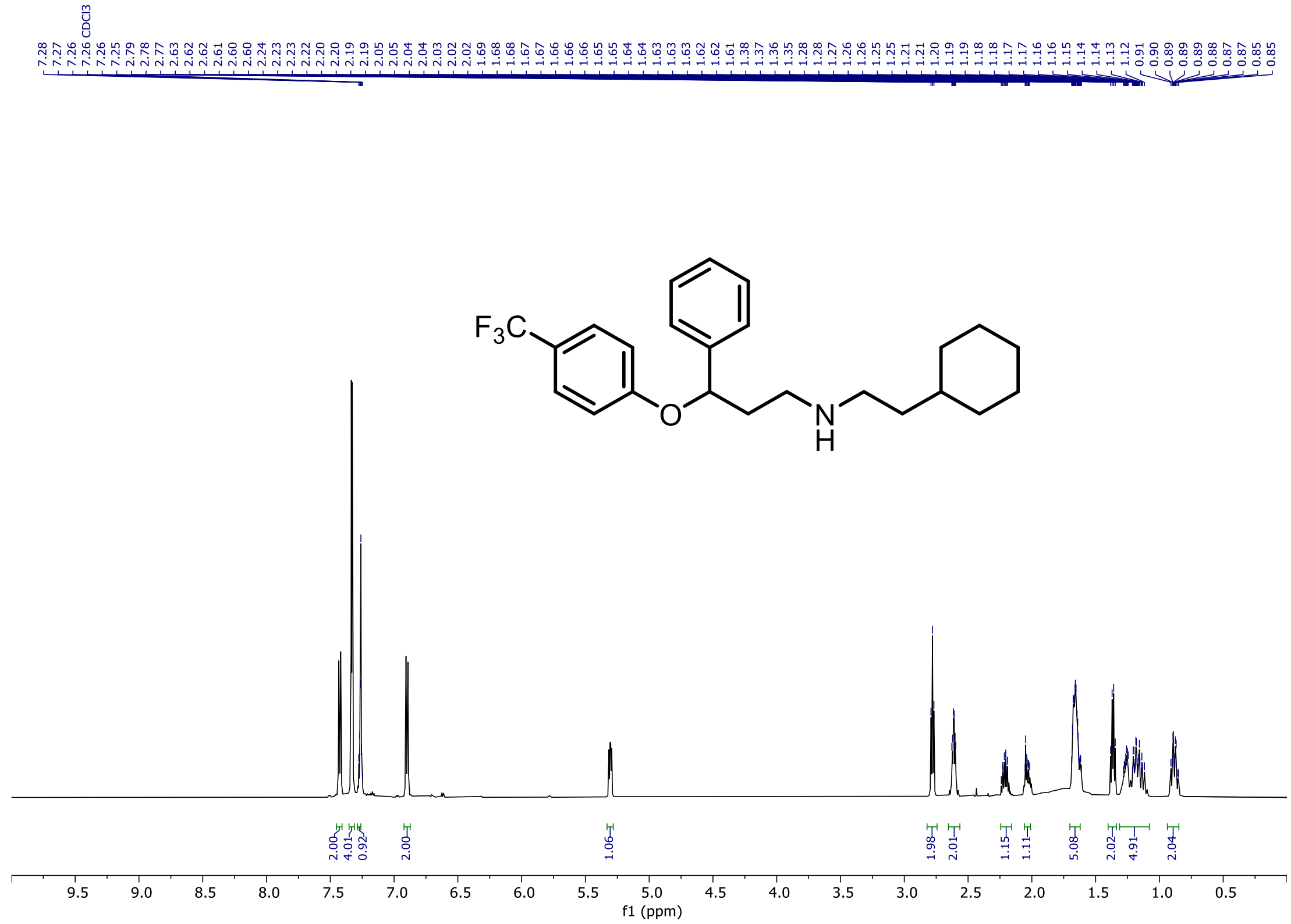

^{13}C NMR (151 MHz, $CDCl_3$)

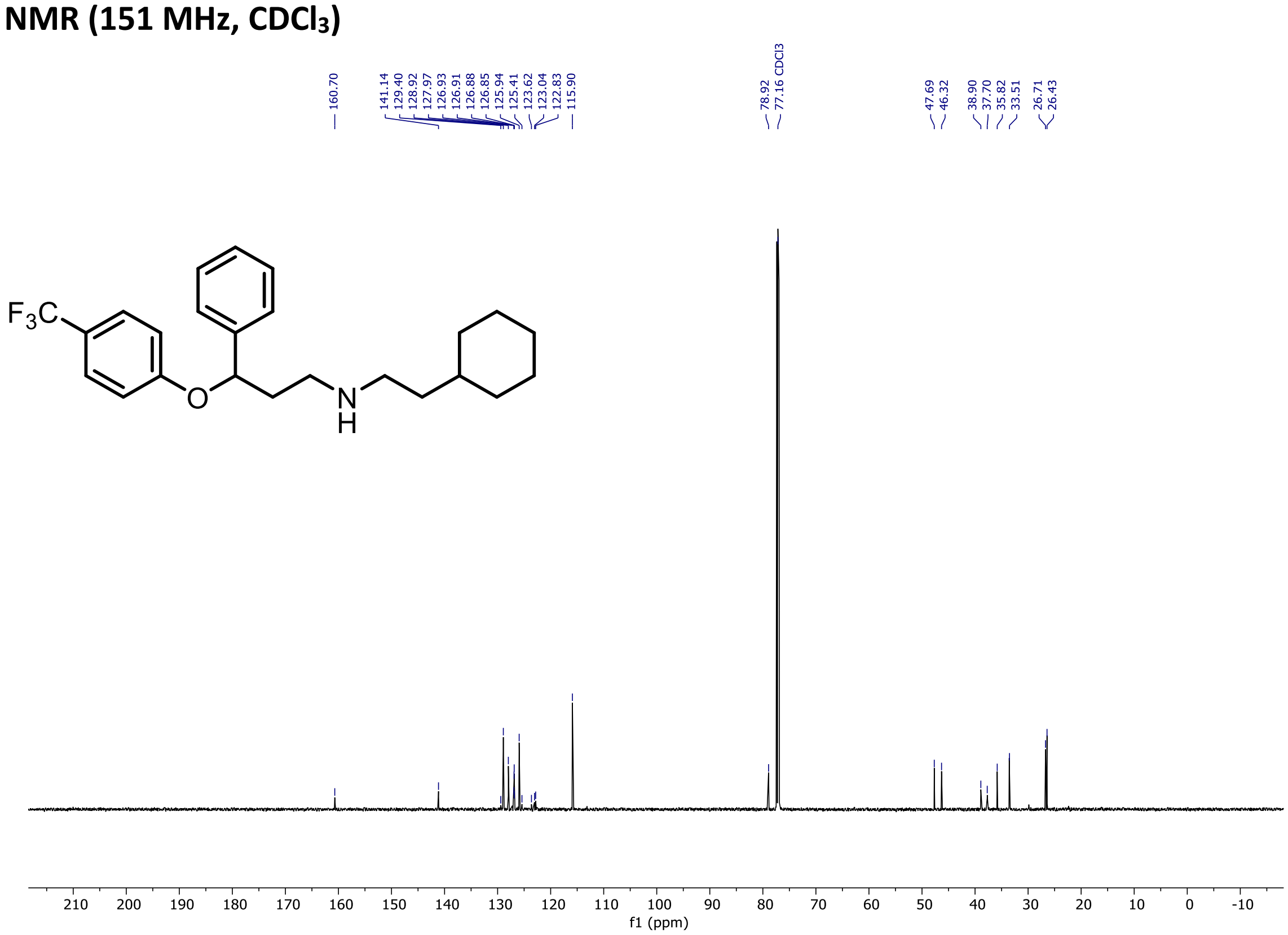

^{19}F NMR (565 MHz, $CDCl_3$)

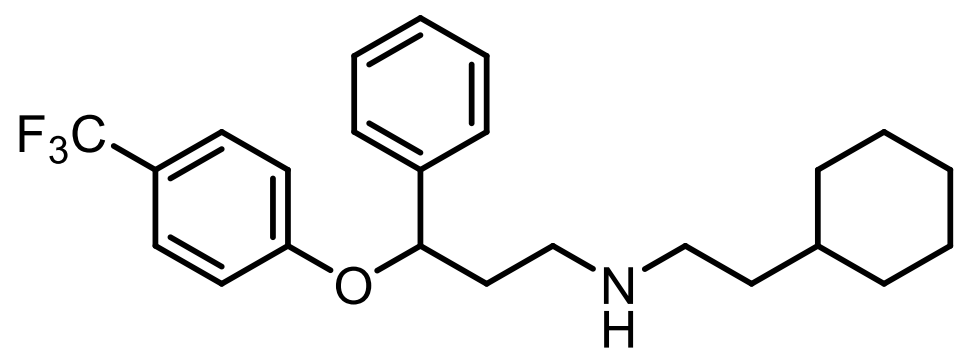

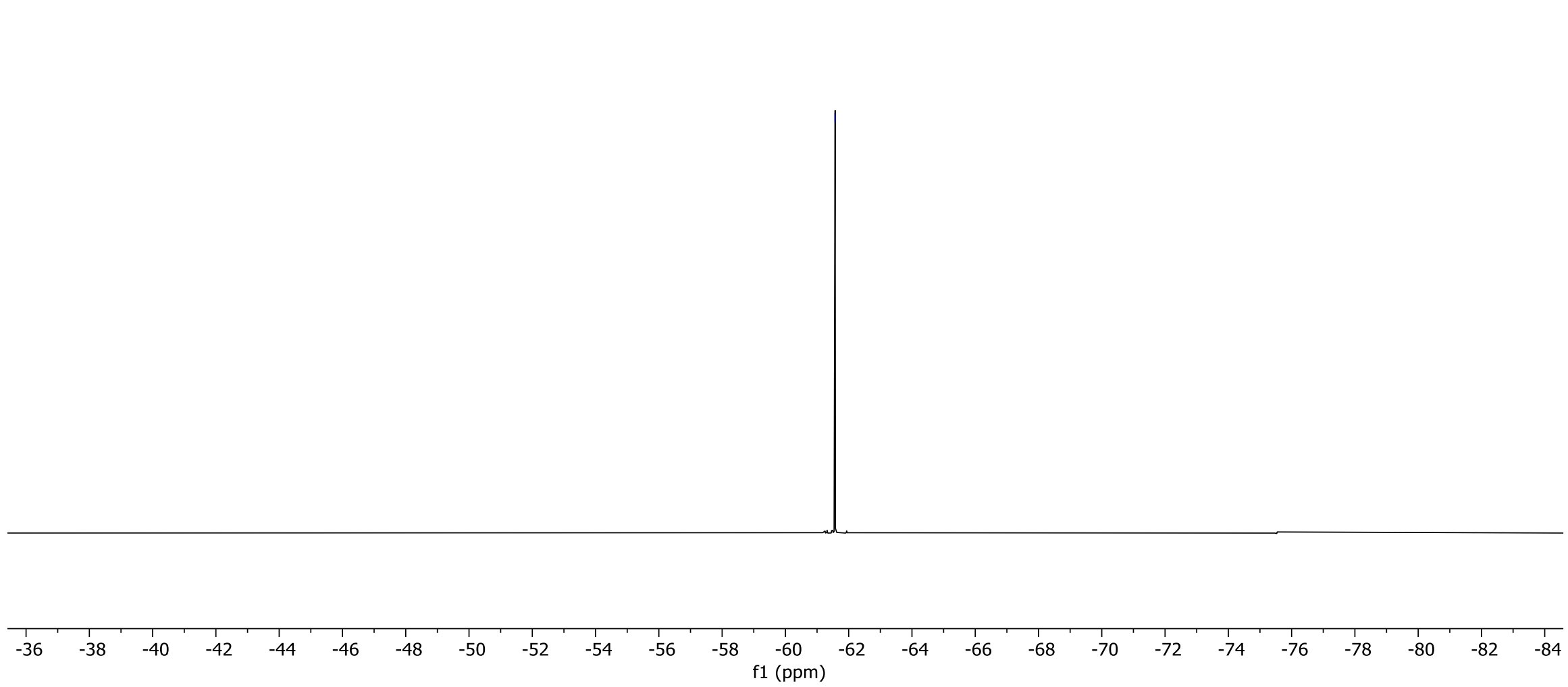

2-(1-(4-Chlorobenzoyl)-5-methoxy-2-methyl-1*H*-indol-3-yl)-*N*-methyl-*N*-(12-(phenethylamino)dodecyl)acetamide (1.16)

^{1}H NMR (600 MHz, $CDCl_3$)

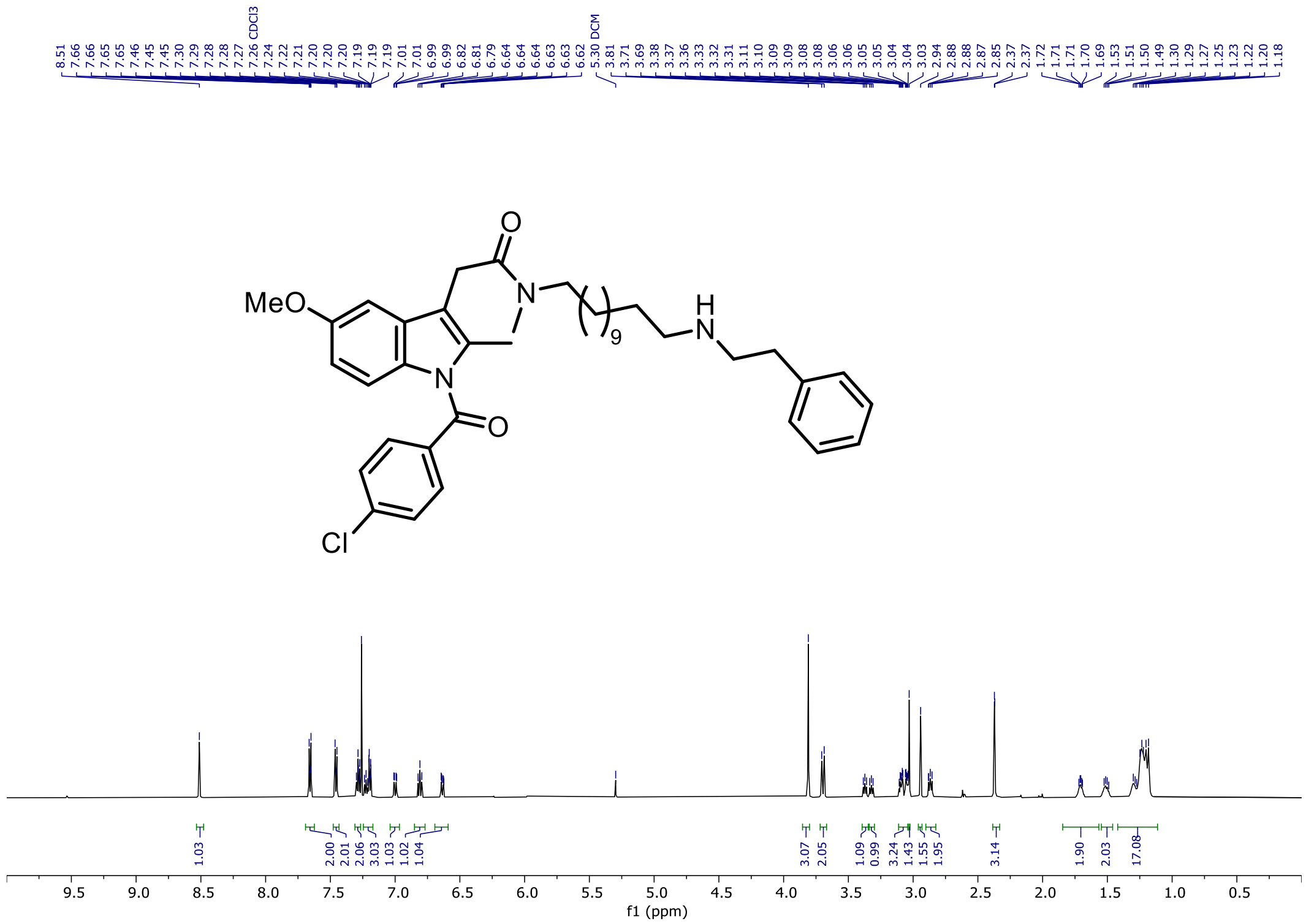

^{13}C NMR (151 MHz, $CDCl_3$)

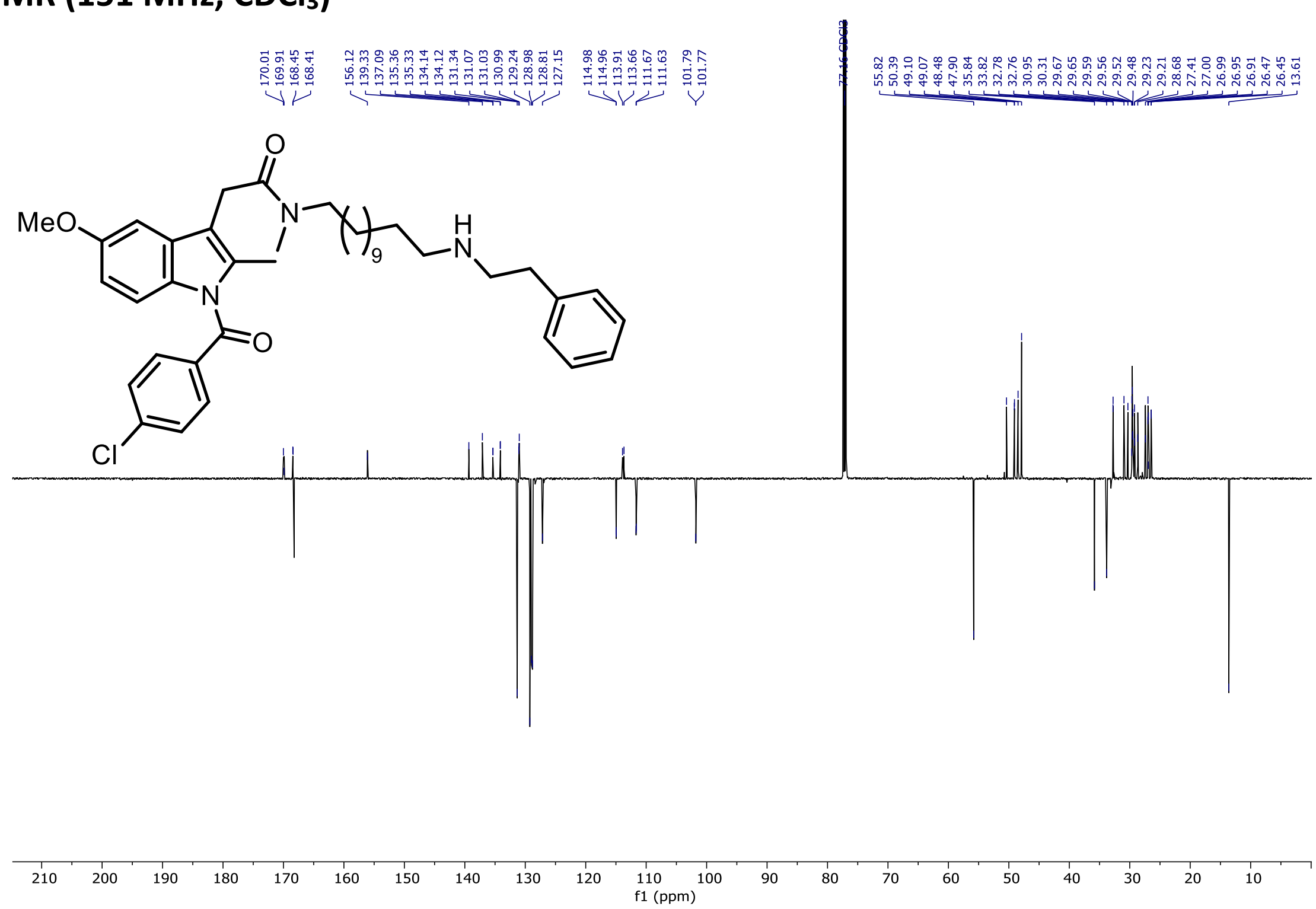

4-(3-(Phenethylamino)propyl)benzyl 3-(4,5-diphenyloxazol-2-yl)propanoate (1.17)
^{1}H NMR (400 MHz, CDCl$_3$)

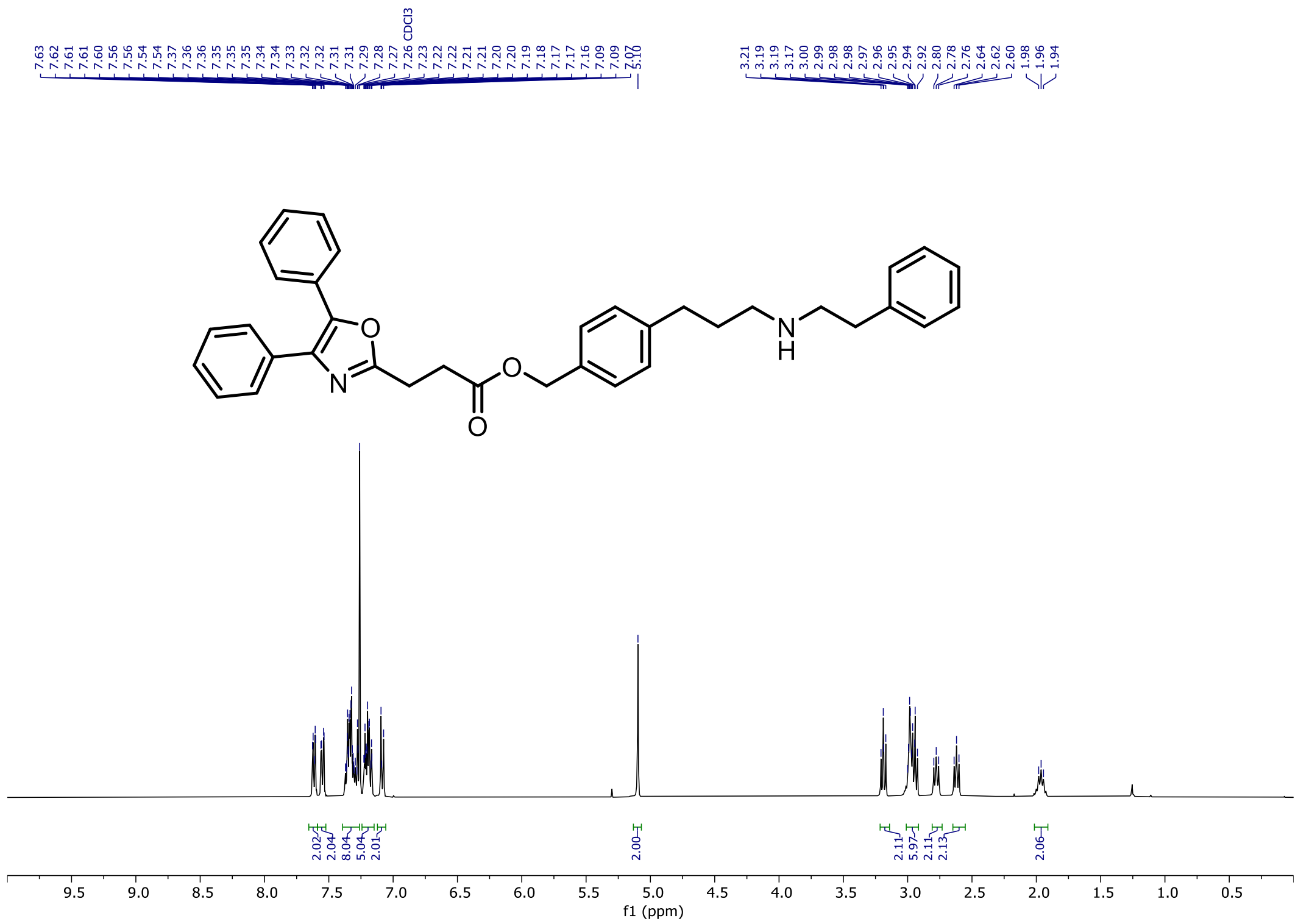

^{13}C NMR (101 MHz, CDCl$_3$)

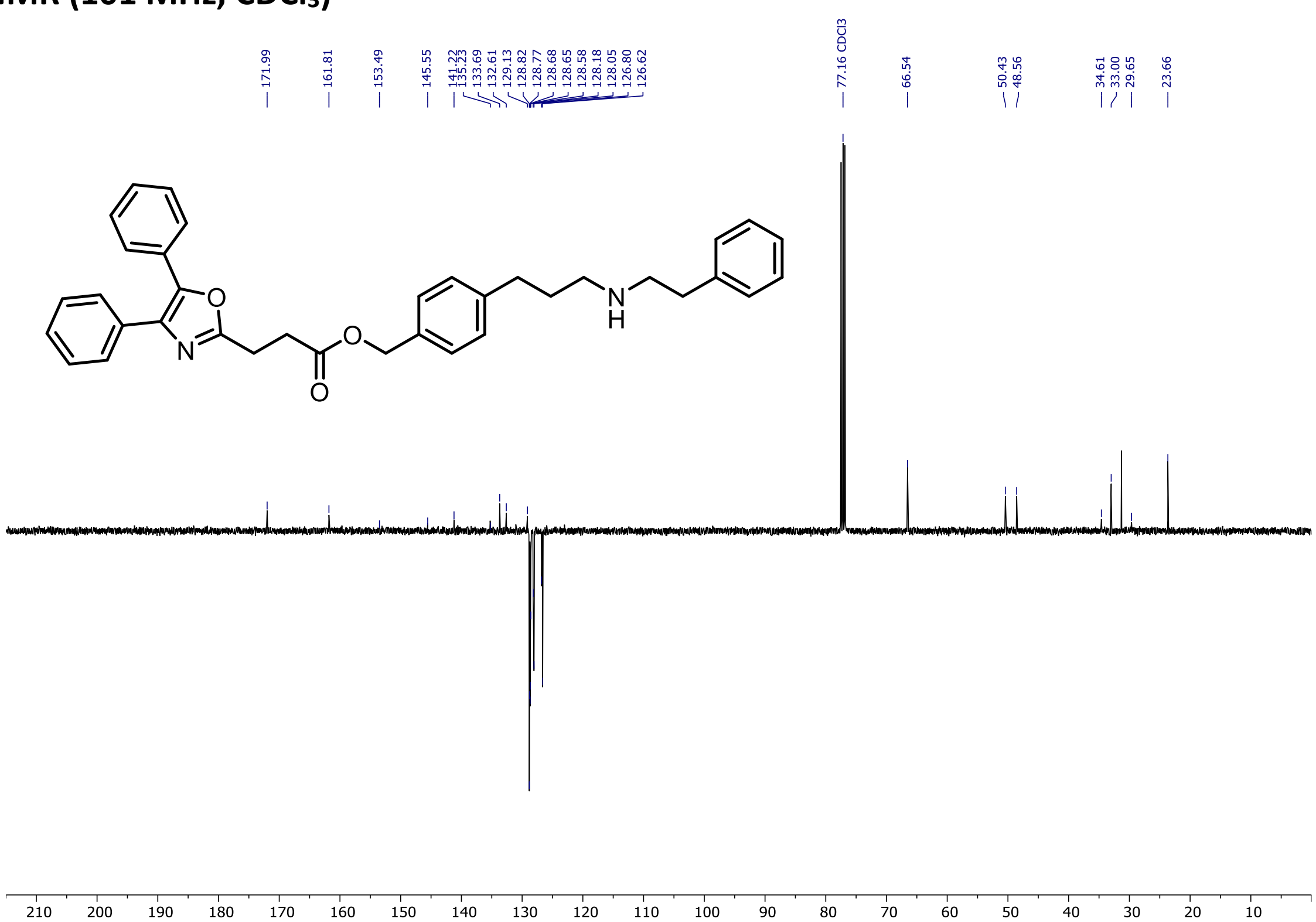

4-(3-(Phenethylamino)propyl)benzyl 4-(*N*,*N*-dipropylsulfamoyl)benzoate (1.18)

^{1}H NMR (400 MHz, CDCl$_3$)

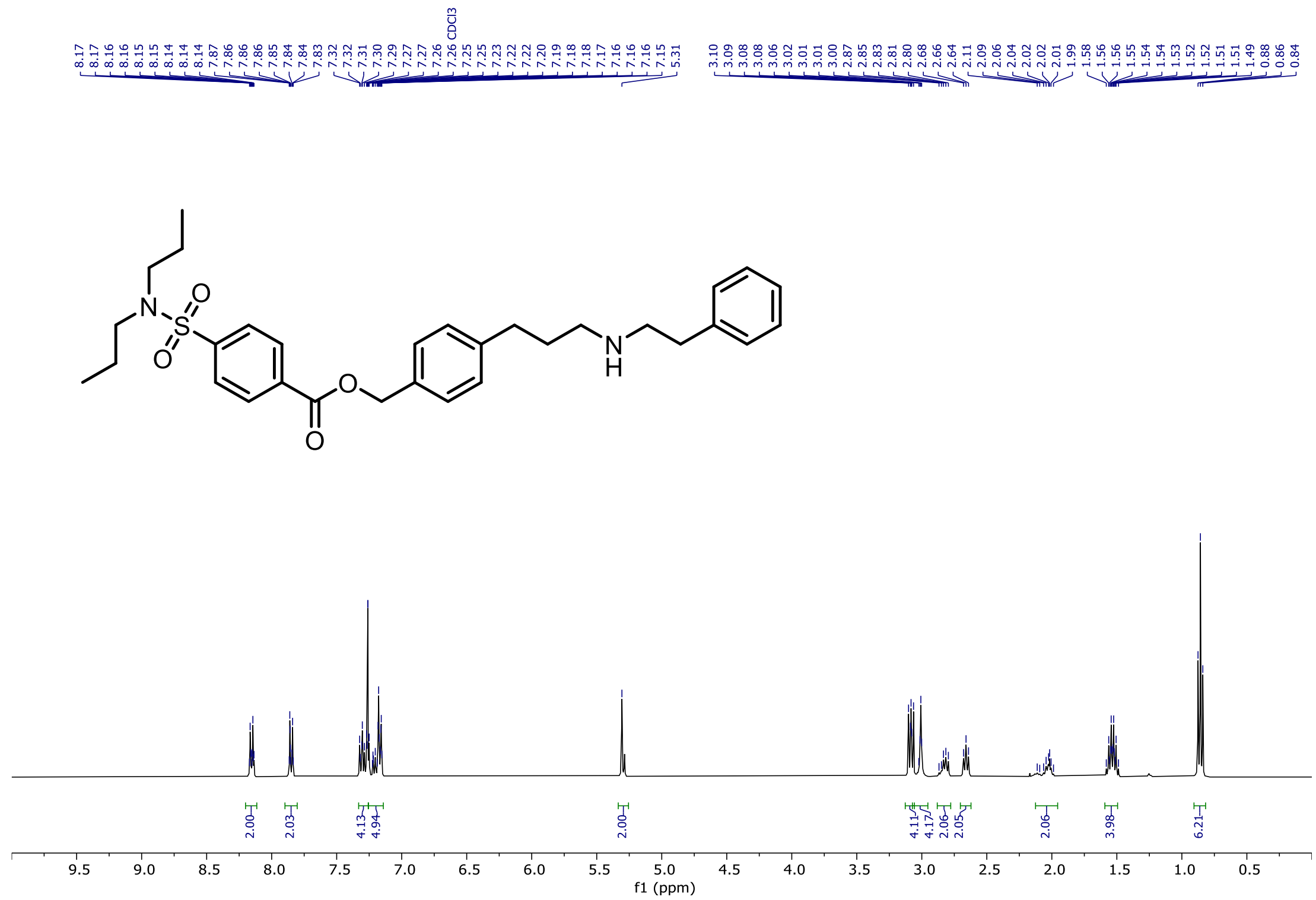

^{13}C NMR (101 MHz, CDCl$_3$)

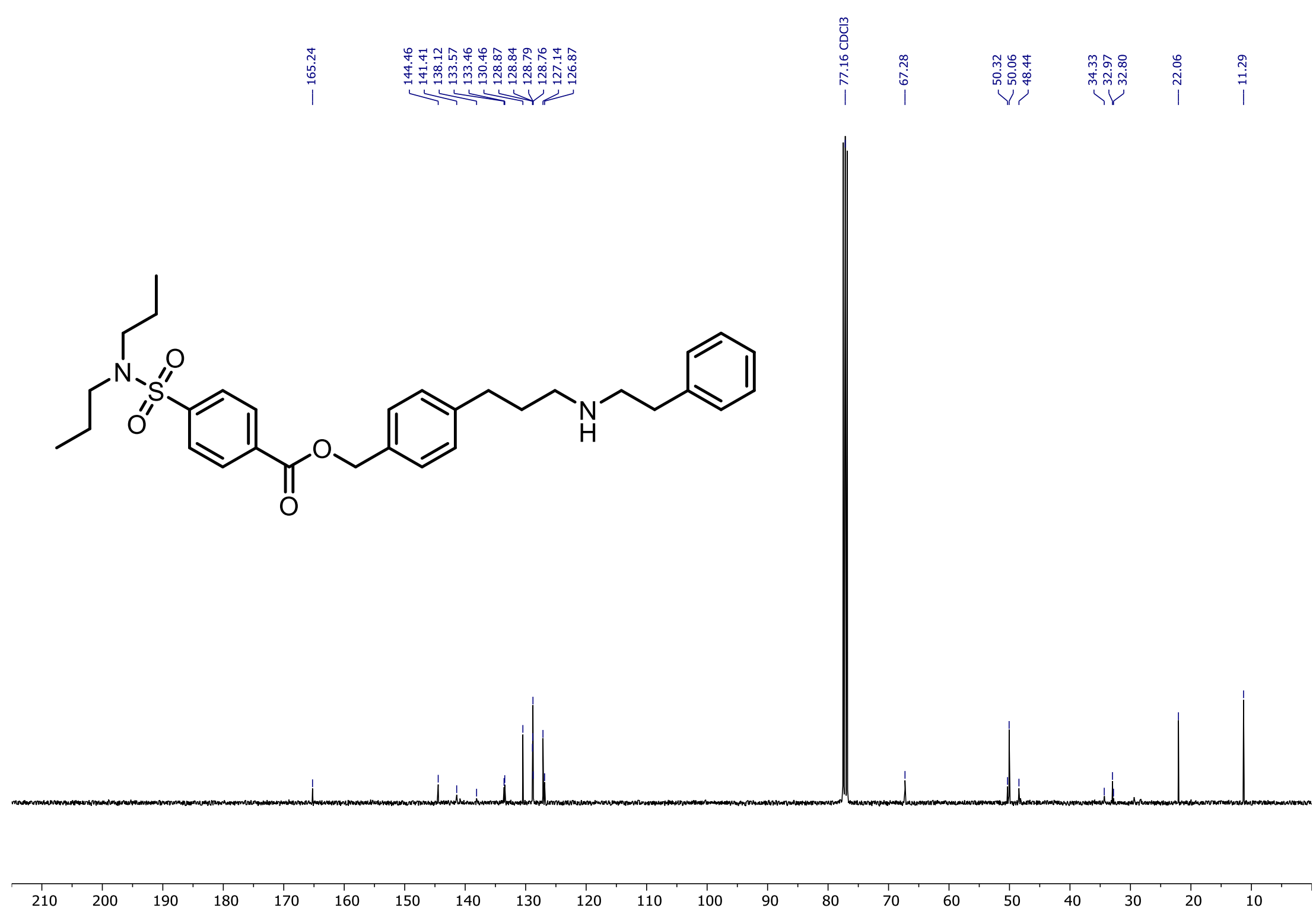

(*S*)-2-(4-(4-Chlorophenyl)-2,3,9-trimethyl-6*H*-thieno[3,2-f][1,2,4]triazolo[4,3-a][1,4]diazepin-6-yl)-*N*-methyl-*N*-(6-(phenethylamino)hexyl)acetamide (1.19)

^{1}H NMR (400 MHz, $CDCl_3$)

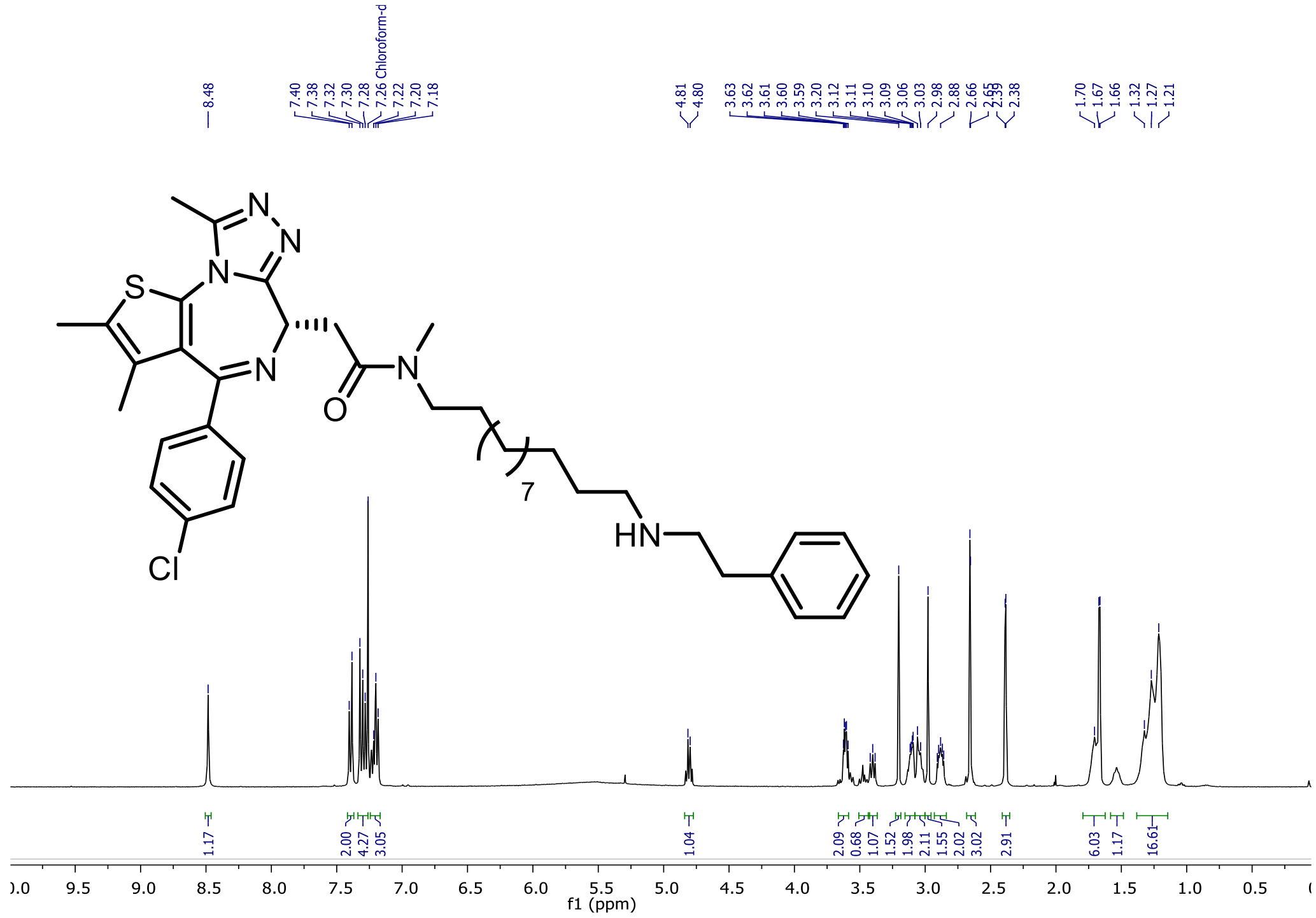

^{13}C NMR (151 MHz, $CDCl_3$)

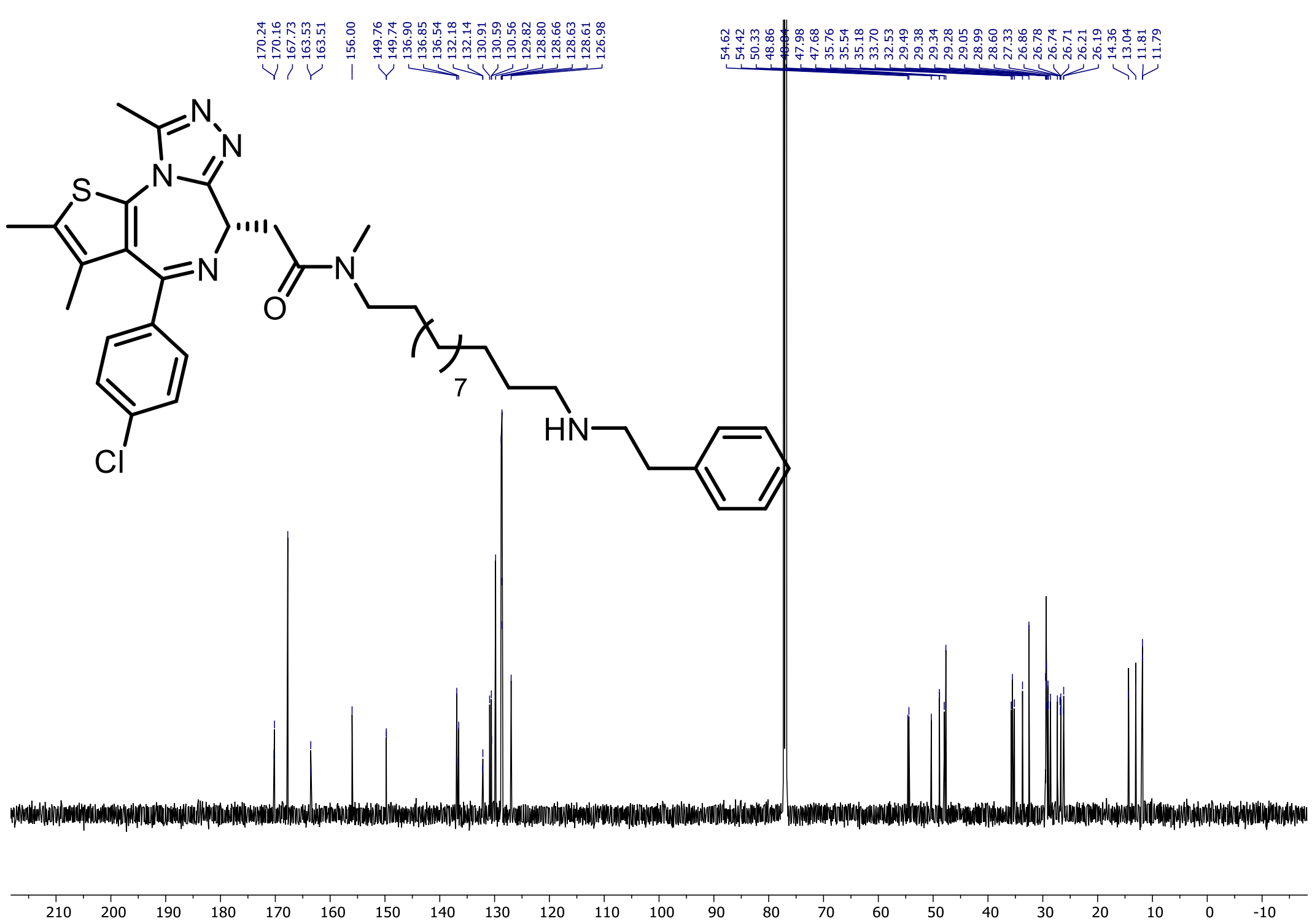

(4*R*)-4-((8*R*,9*S*,10*S*,13*R*,14*S*,17*R*)-10,13-Dimethyl-3,7,12-trioxohexadecahydro-1*H*-cyclopenta[*a*]phenanthren-17-yl)-*N*-methyl-*N*-(12-(phenethylamino)dodecyl)pentanamide (1.20)

^{1}H NMR (600 MHz, CDCl$_3$)

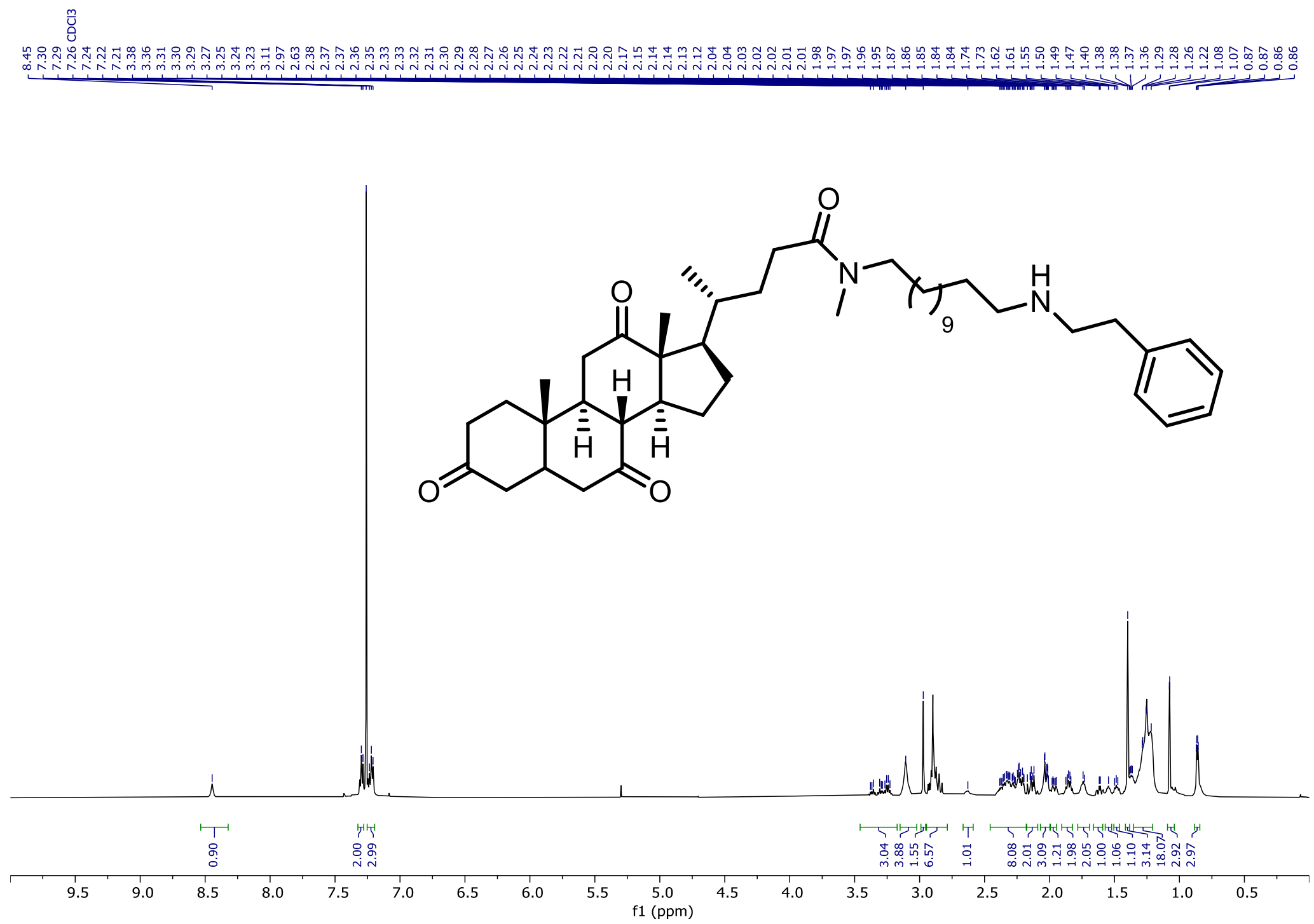

^{13}C NMR (151 MHz, CDCl$_3$)

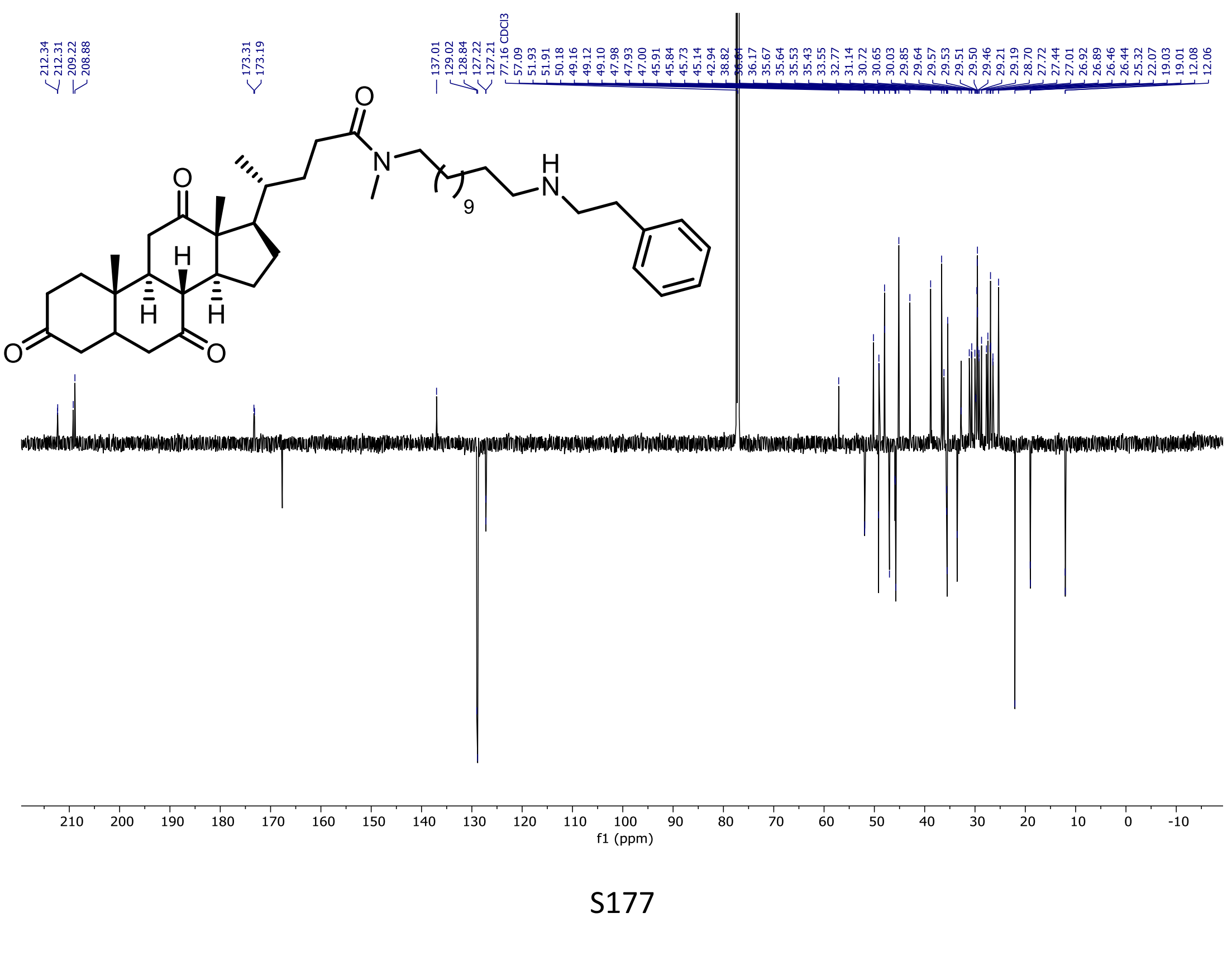

(*8R,9S,13S,14S*)-13-Methyl-3-((4-(3-(phenethylamino)propyl)benzyl)oxy)-6,7,8,9,11,12,13,14,15,16-decahydro-17*H*-cyclopenta[*a*]phenanthren-17-one (1.21)

^{1}H NMR (400 MHz, $CDCl_3$)

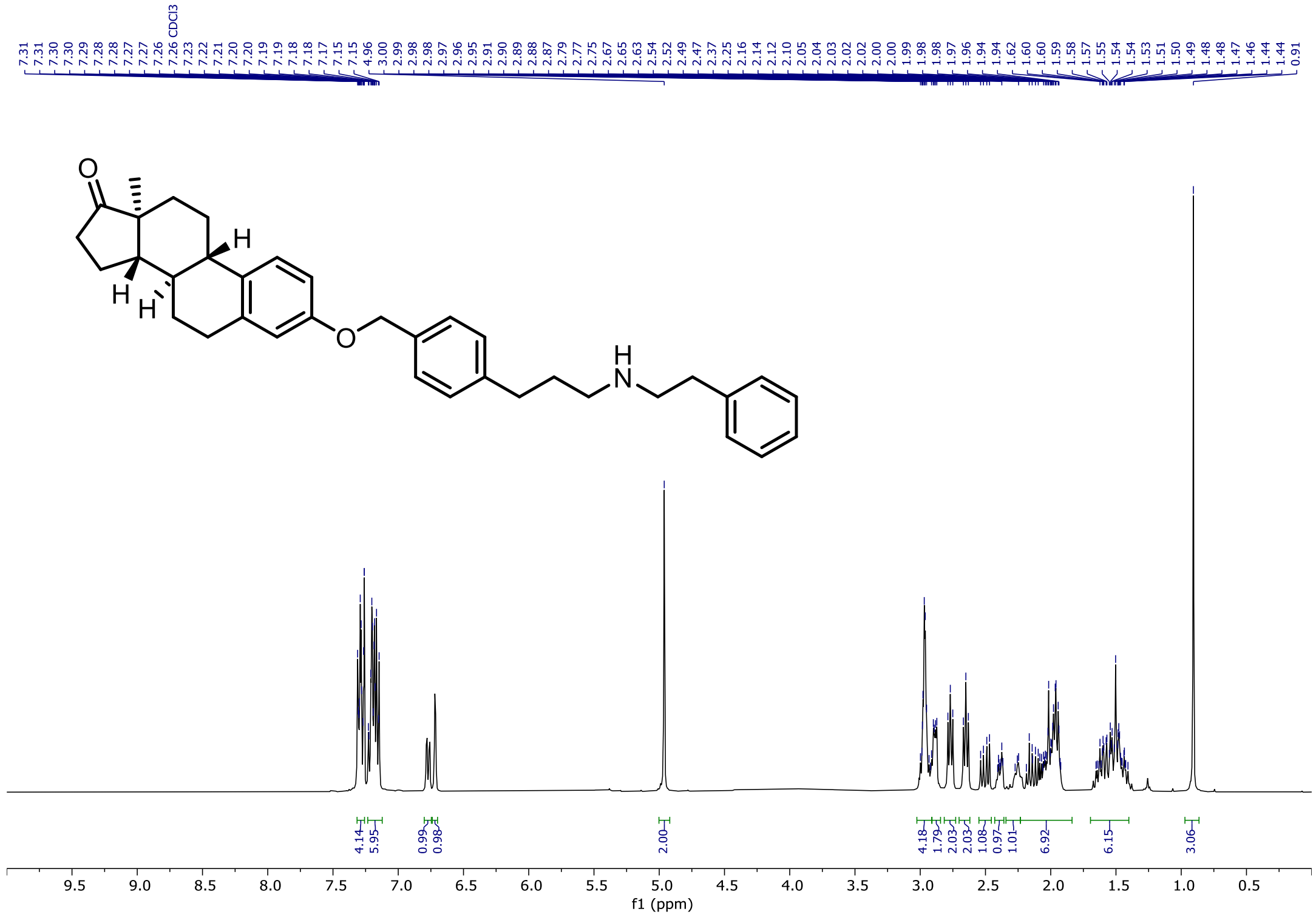

^{13}C NMR (101 MHz, $CDCl_3$)

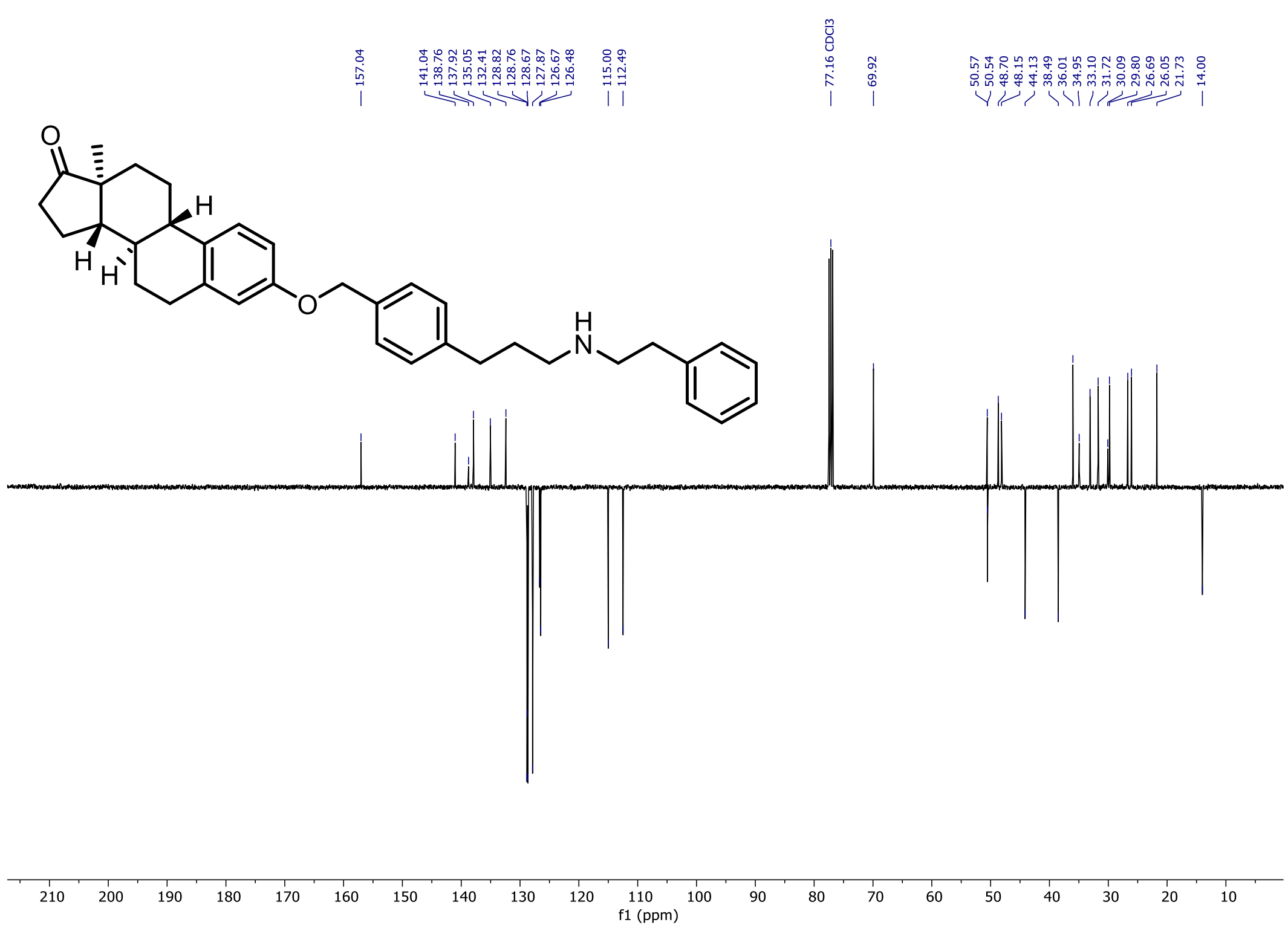

(*4R*)-4-((*8R,9S,10S,13R,14S,17R*)-10,13-Dimethyl-3,7,12-trioxohexadecahydro-1*H*-cyclopenta[*a*]phenanthren-17-yl)-*N*-methyl-*N*-(12-(phenethylamino)dodecyl)pentanamide (1.22)

^{1}H NMR (600 MHz, CDCl$_3$)

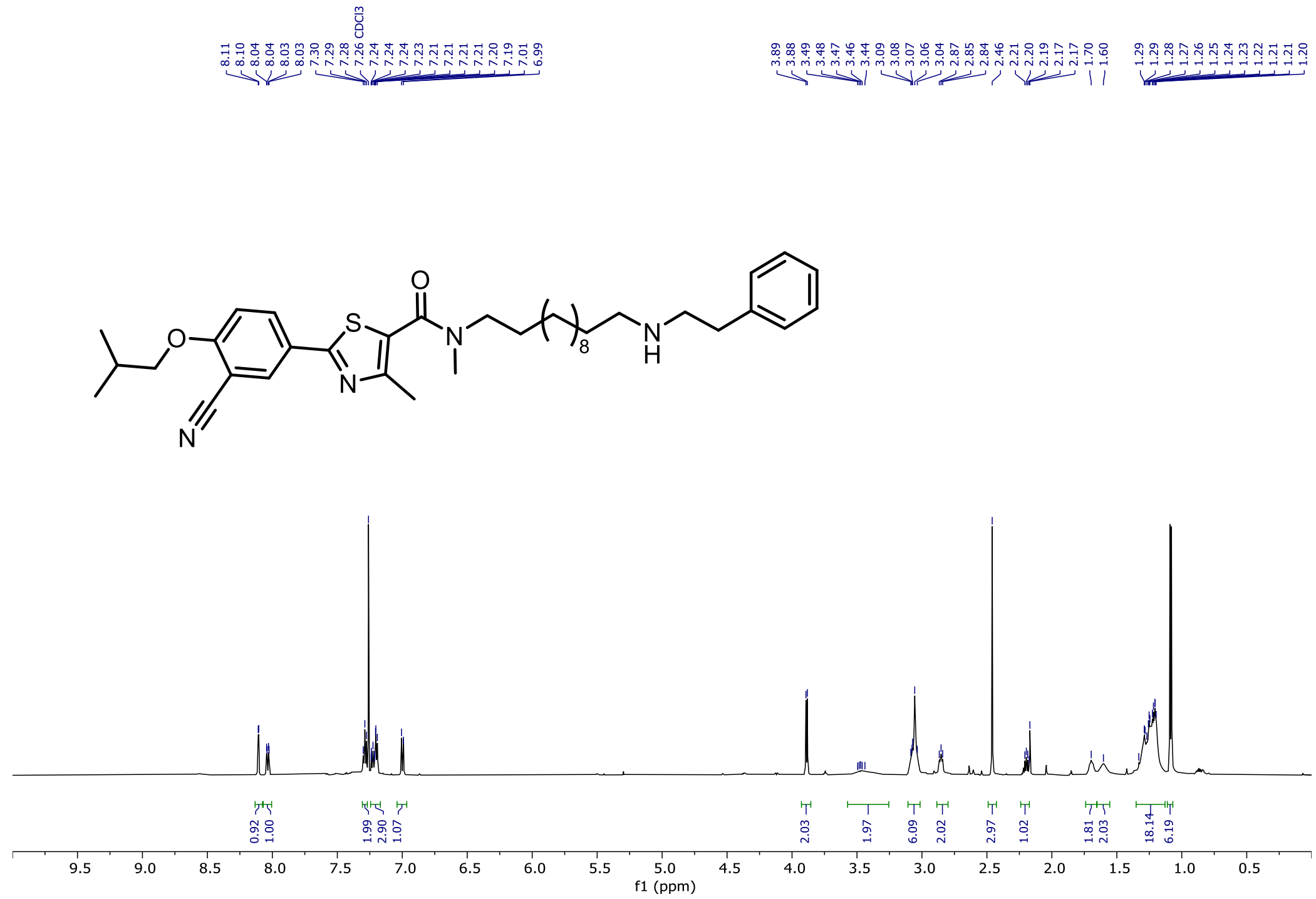

^{13}C NMR (151 MHz, CDCl$_3$)

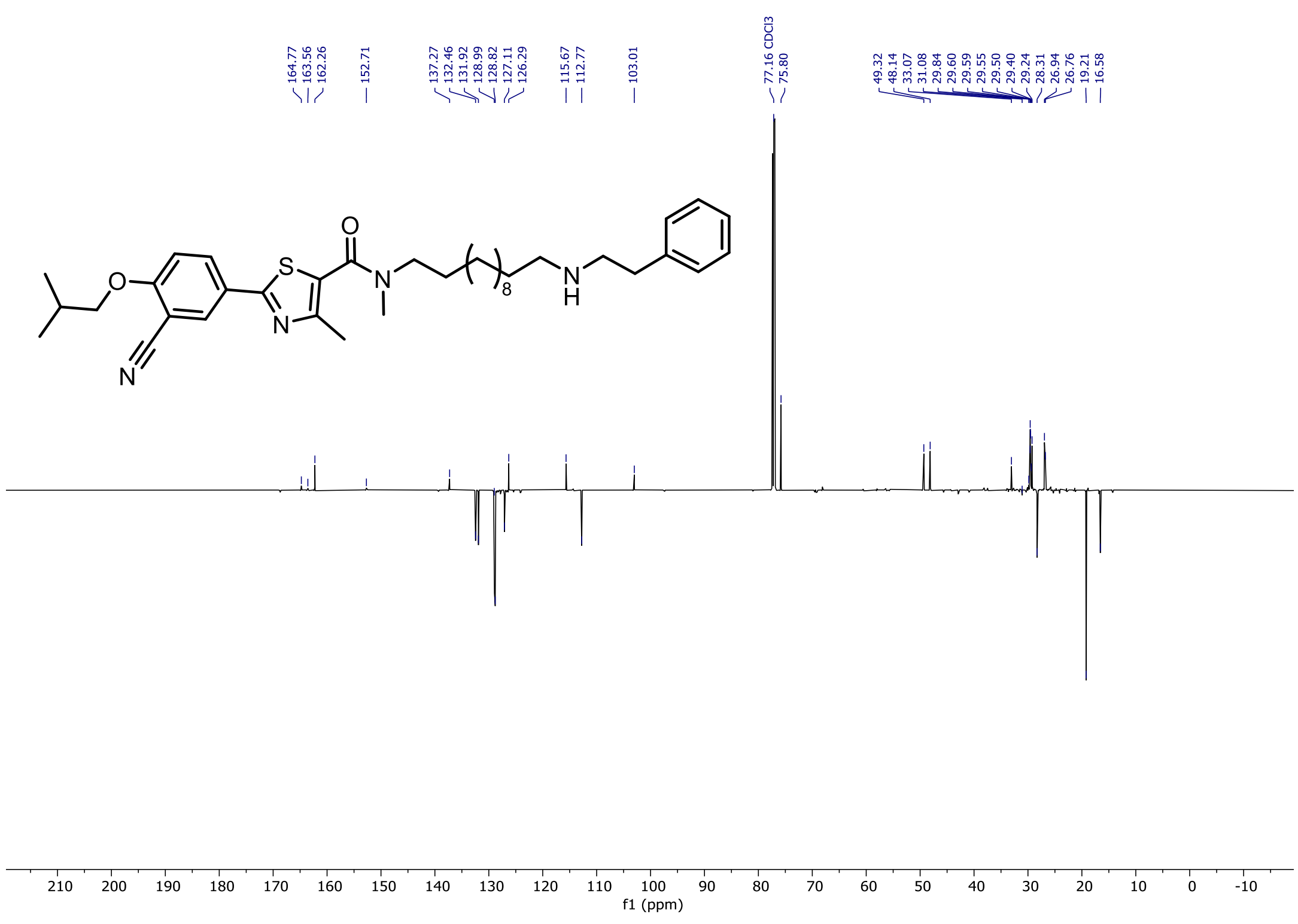

8-Bromo-*N*-phenethyloctan-1-amine (1.23)

^{1}H NMR (400 MHz, CDCl$_3$)

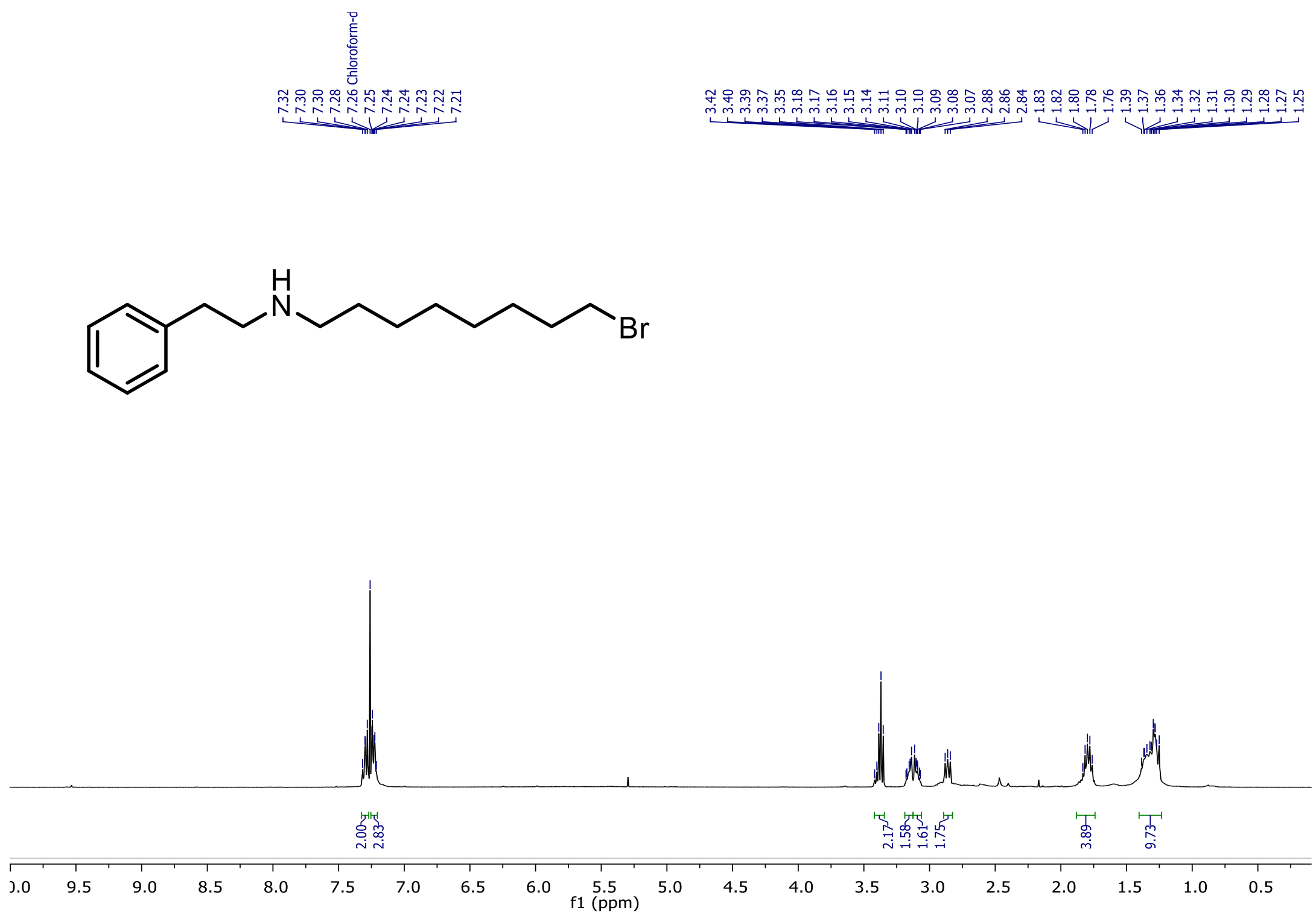

^{13}C NMR (101 MHz, CDCl$_3$)

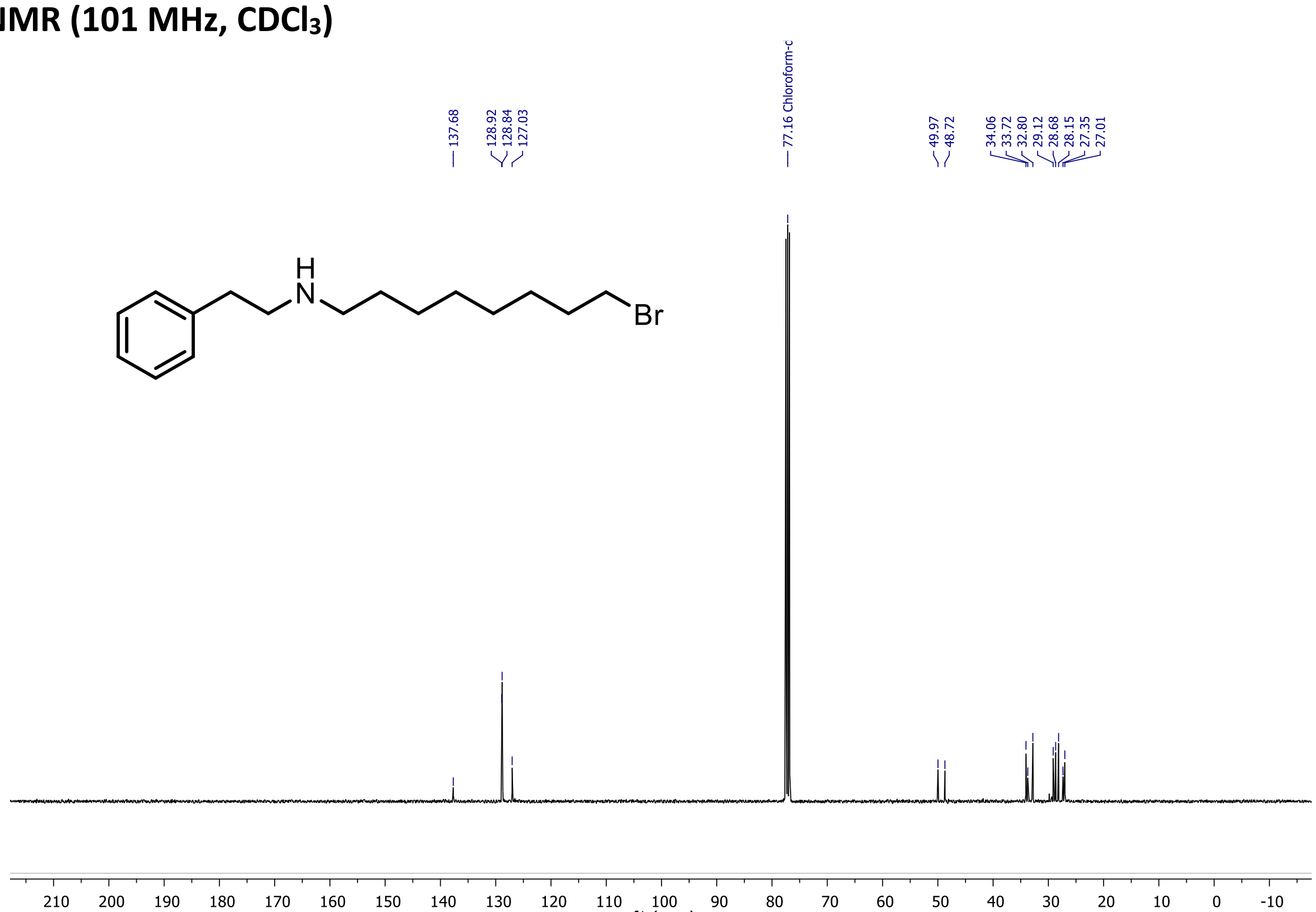

5-(Phenethylamino)pentyl acetate (1.24)

^{1}H NMR (400 MHz, CDCl$_3$)

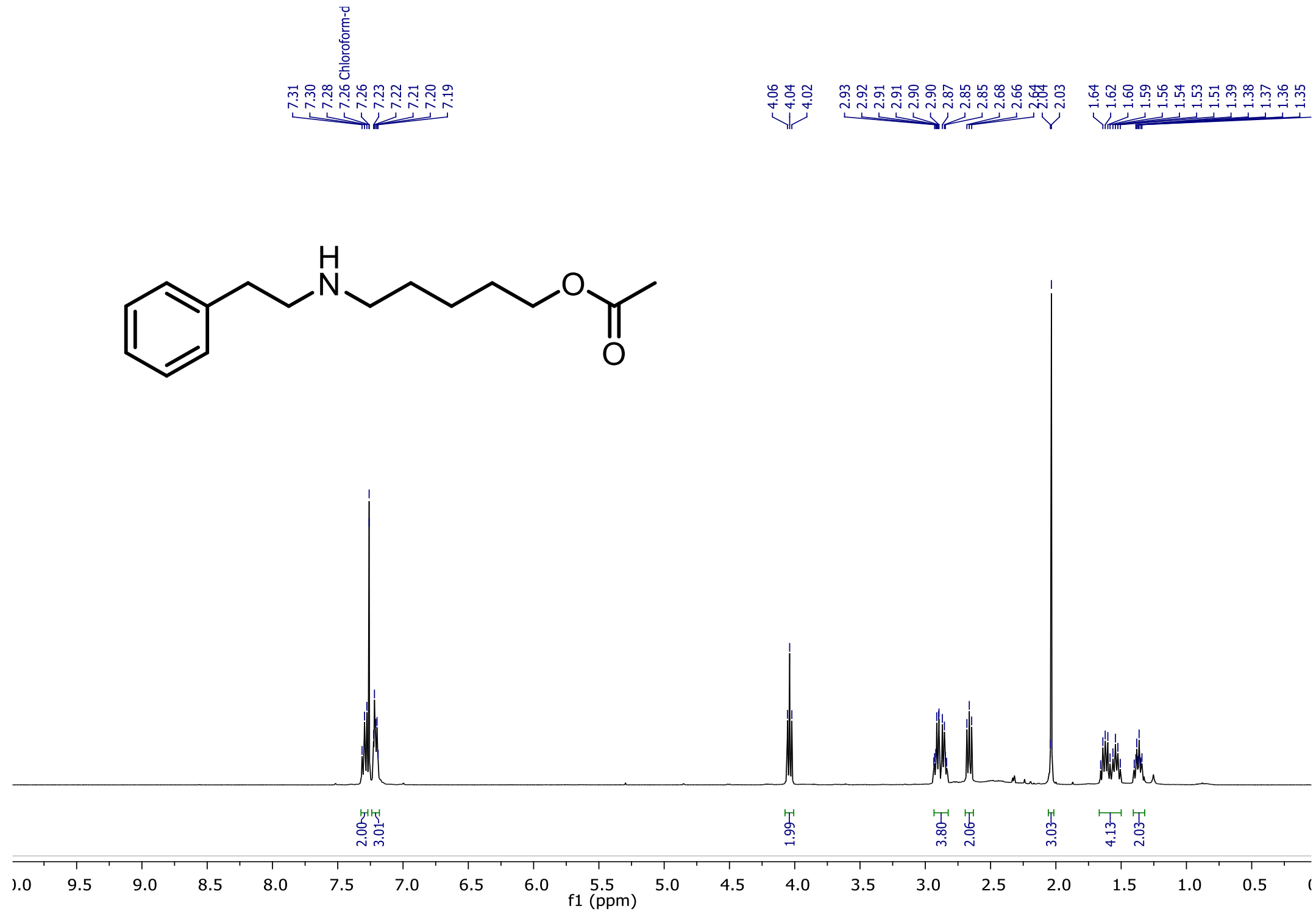

^{13}C NMR (101 MHz, CDCl$_3$)

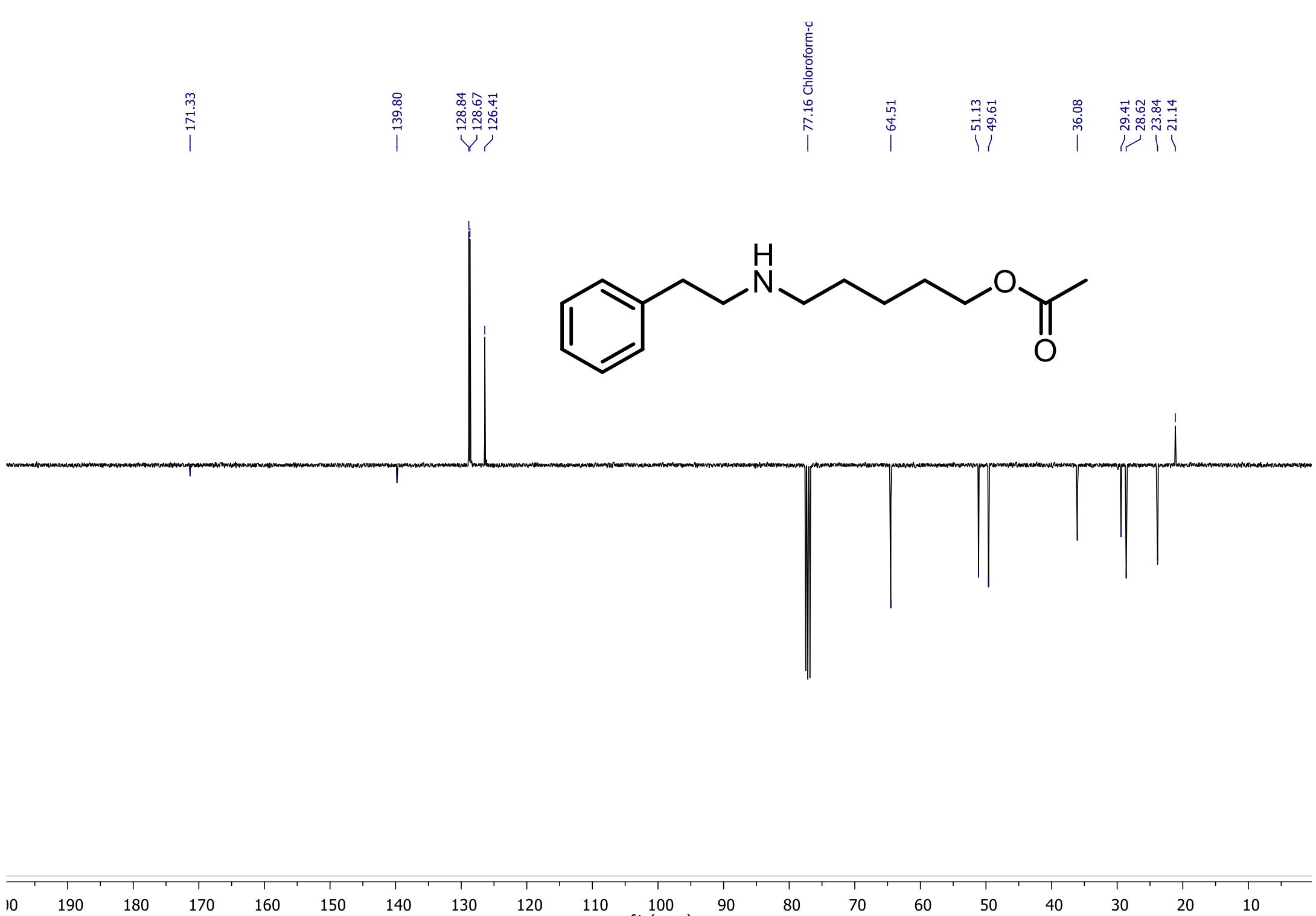

Methyl 7-(phenethylamino)heptanoate (1.25)
^{1}H NMR (400 MHz, $CDCl_3$)

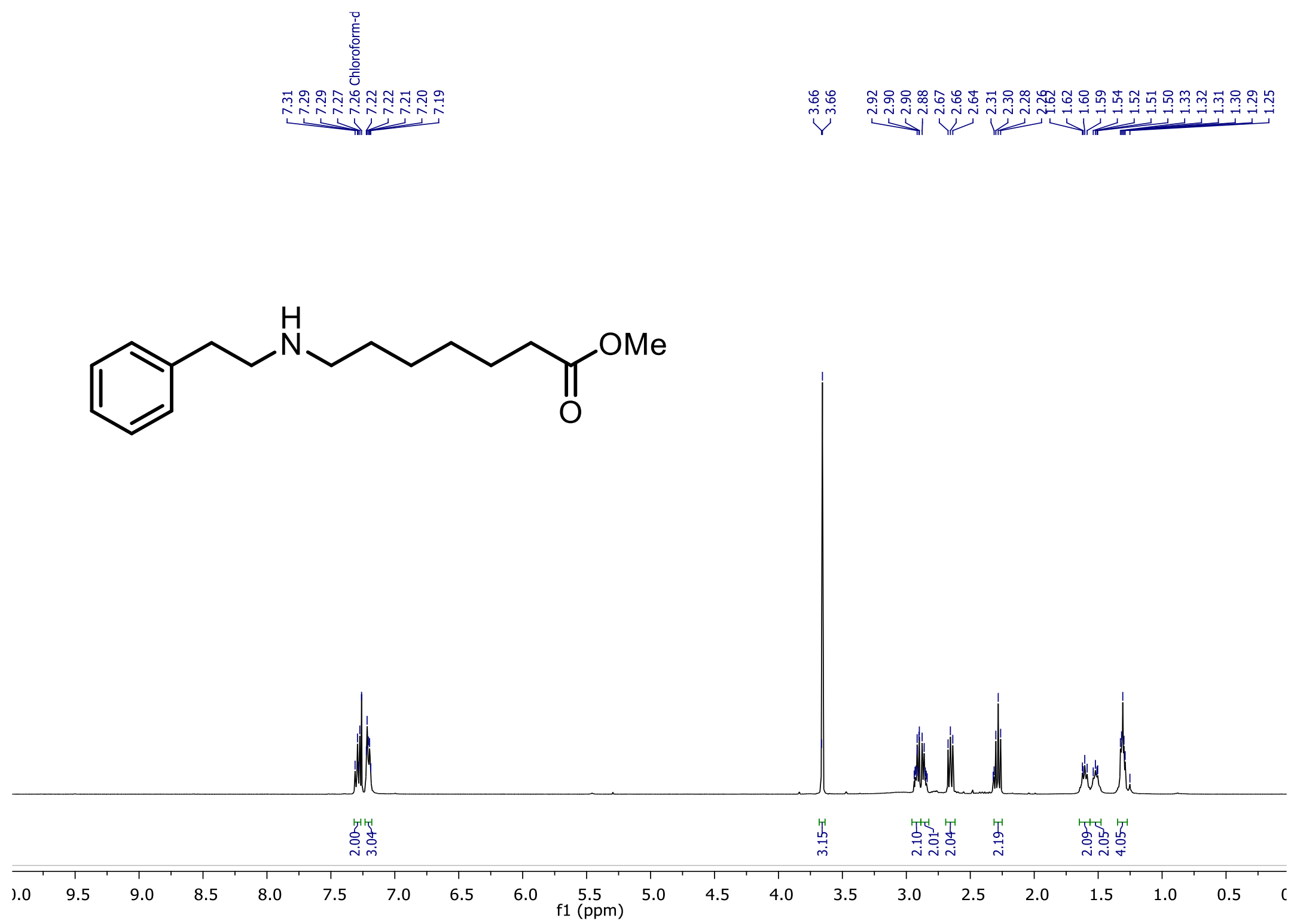

^{13}C NMR (101 MHz, $CDCl_3$)

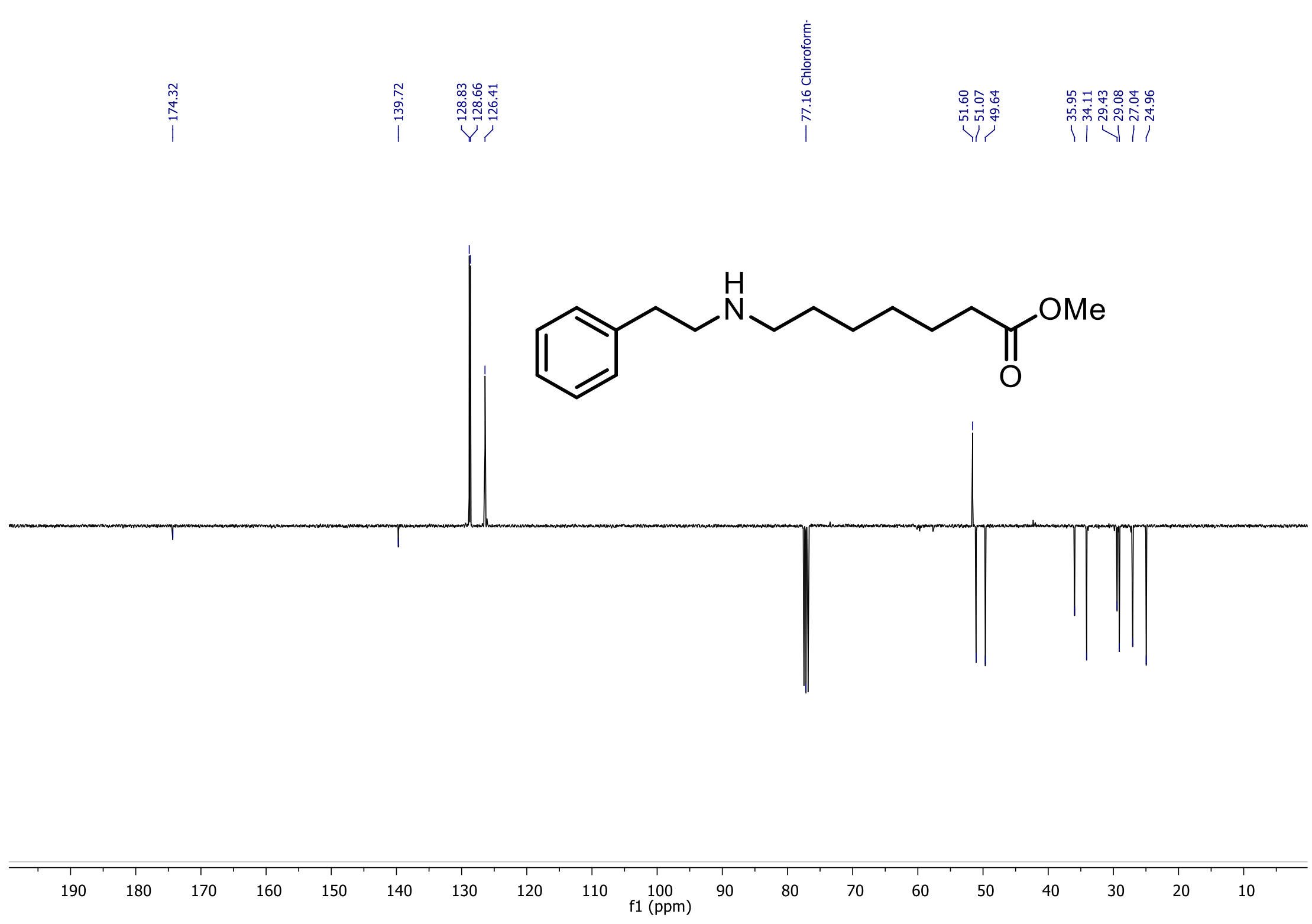

2-(5-(Phenethylamino)pentyl)isoindoline-1,3-dione (1.26)

^{1}H NMR (400 MHz, $CDCl_3$) ^{13}C NMR (101 MHz, $CDCl_3$)

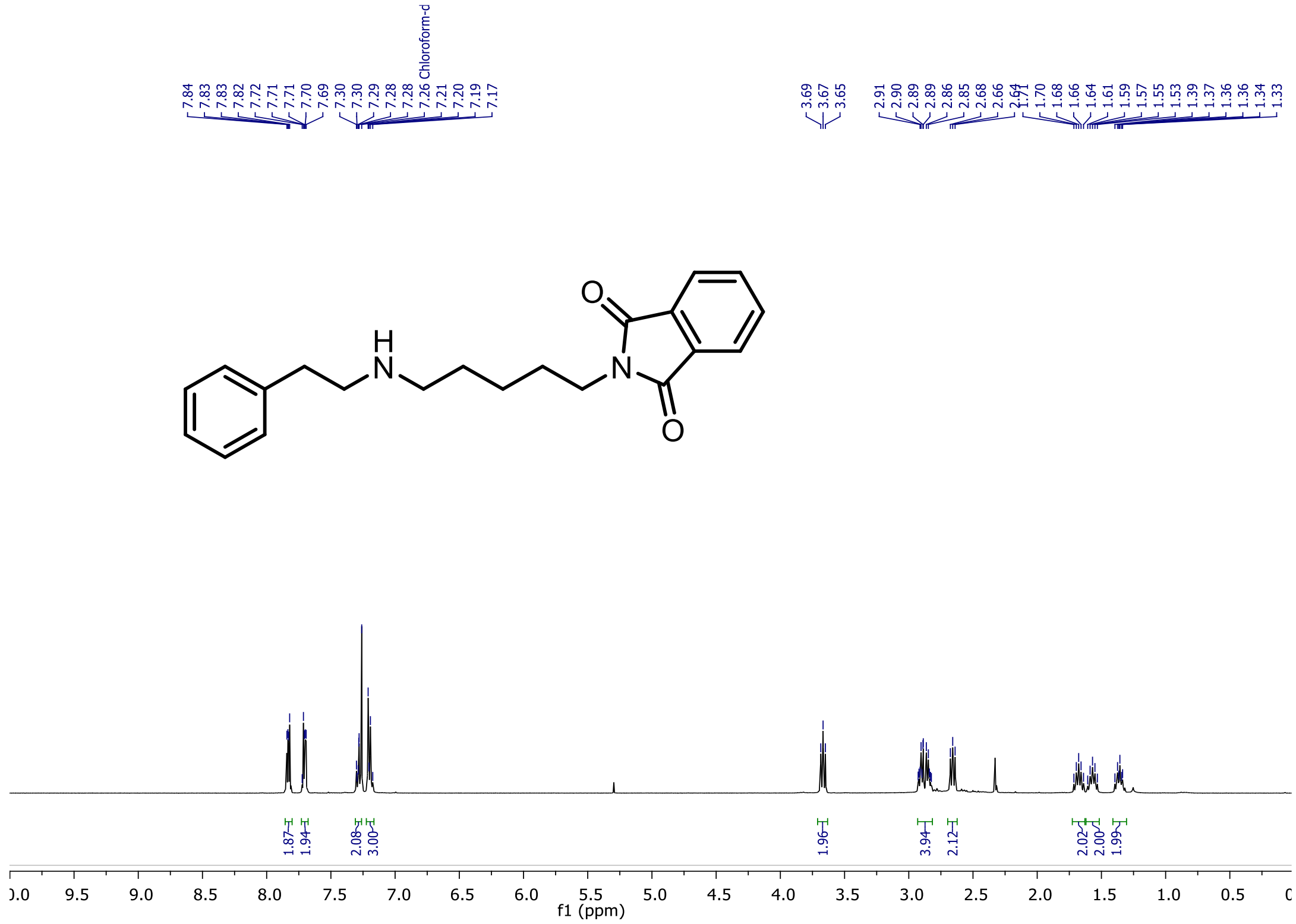

^{13}C NMR (101 MHz, $CDCl_3$)

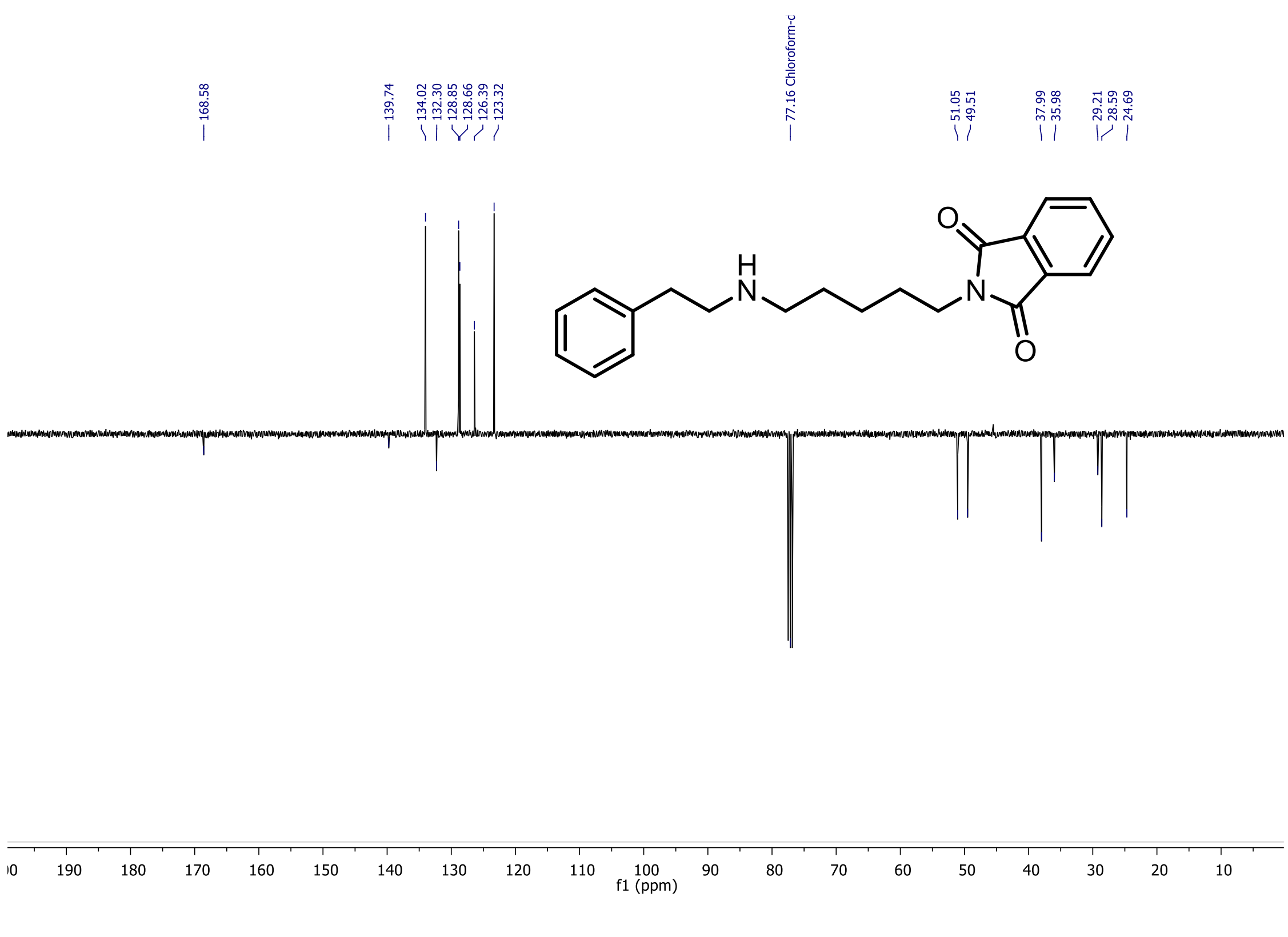

Diethyl (9-(phenethylamino)nonyl)phosphonate (1.27)

^{1}H NMR (400 MHz, $CDCl_3$)

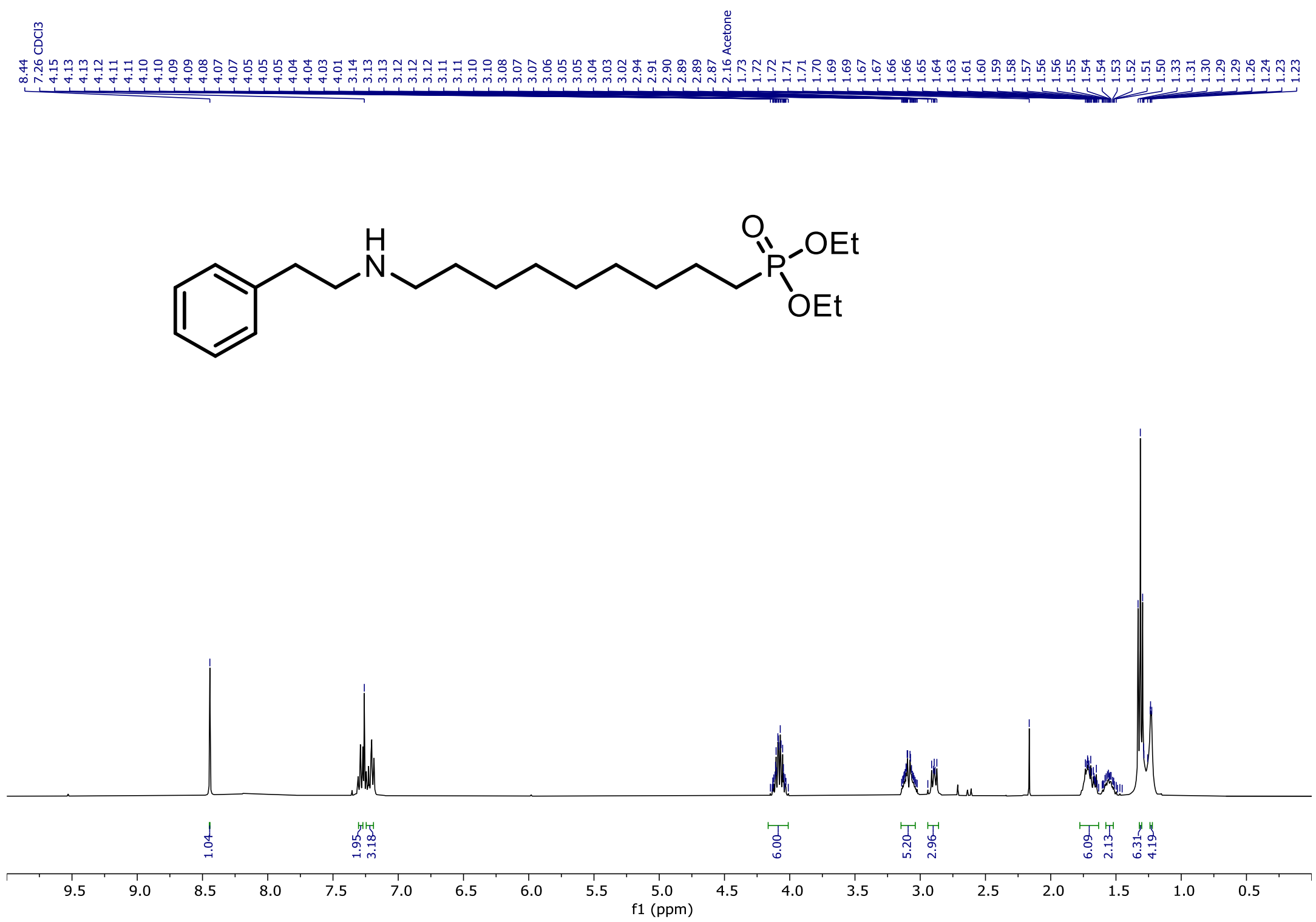

^{13}C NMR (101 MHz, $CDCl_3$)

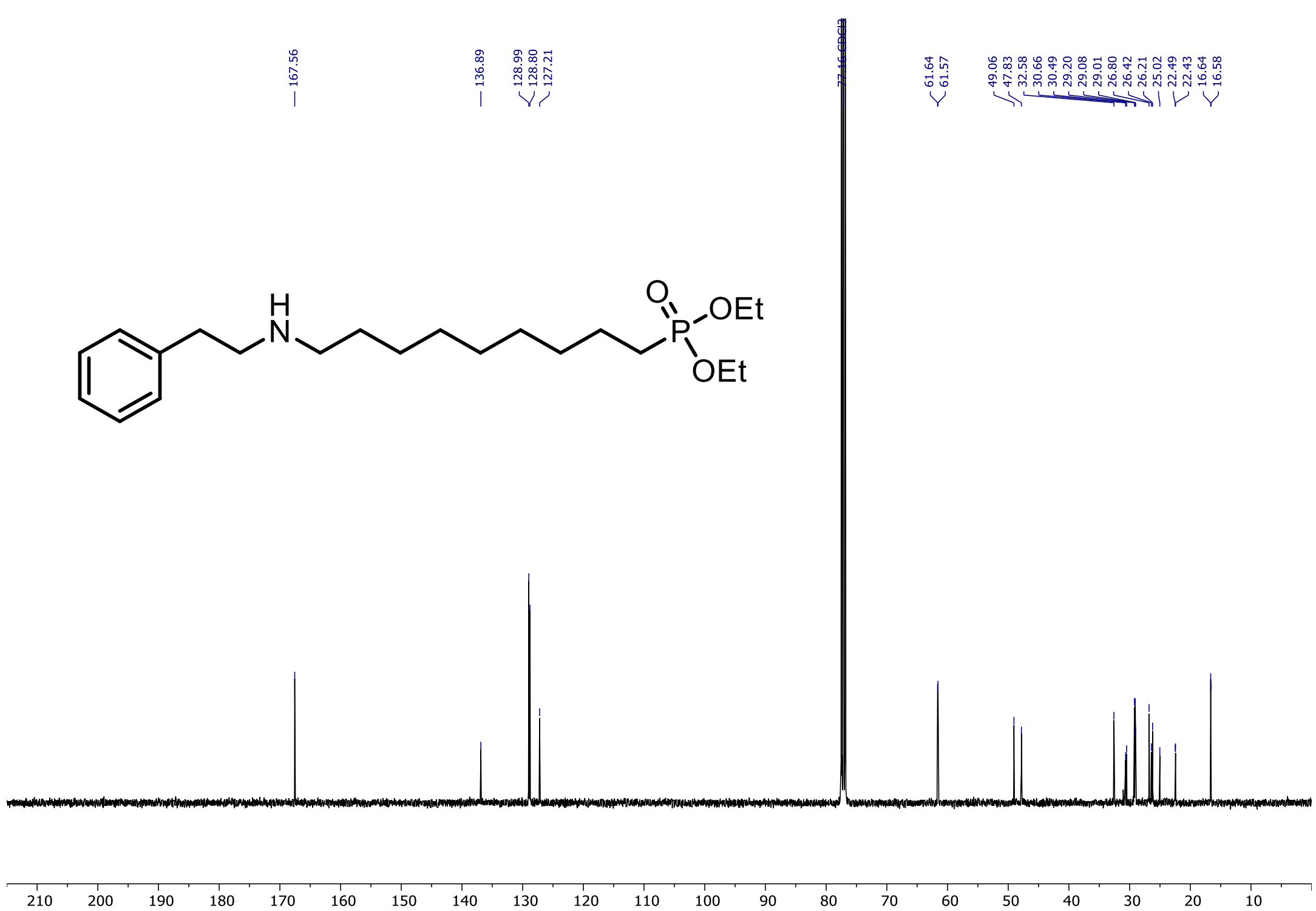

***N*-Phenethyl-9-(phenylsulfonyl)nonan-1-amine(1.28)**

^{1}H NMR (400 MHz, CDCl$_3$)

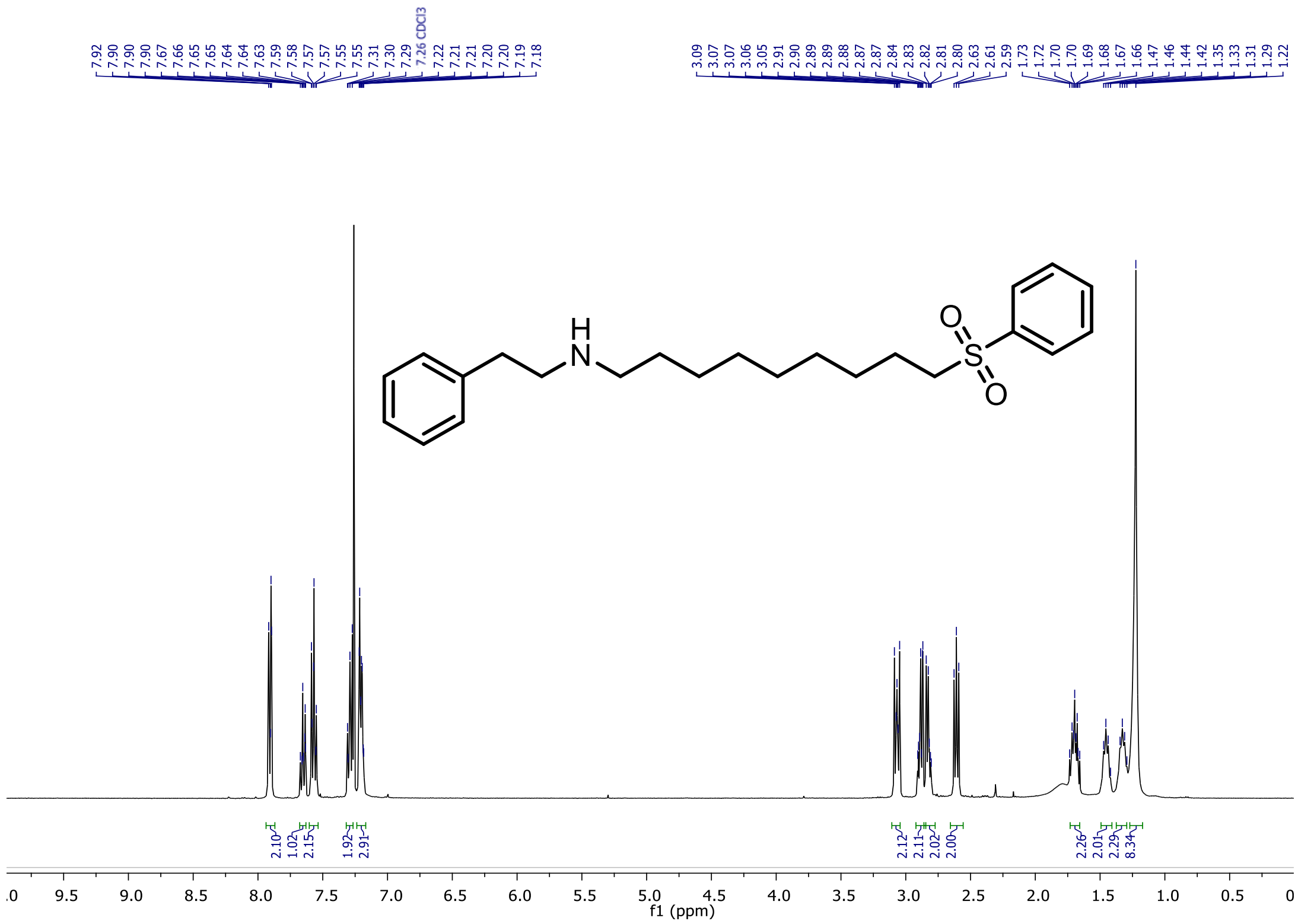

^{13}C NMR (151 MHz, CDCl$_3$)

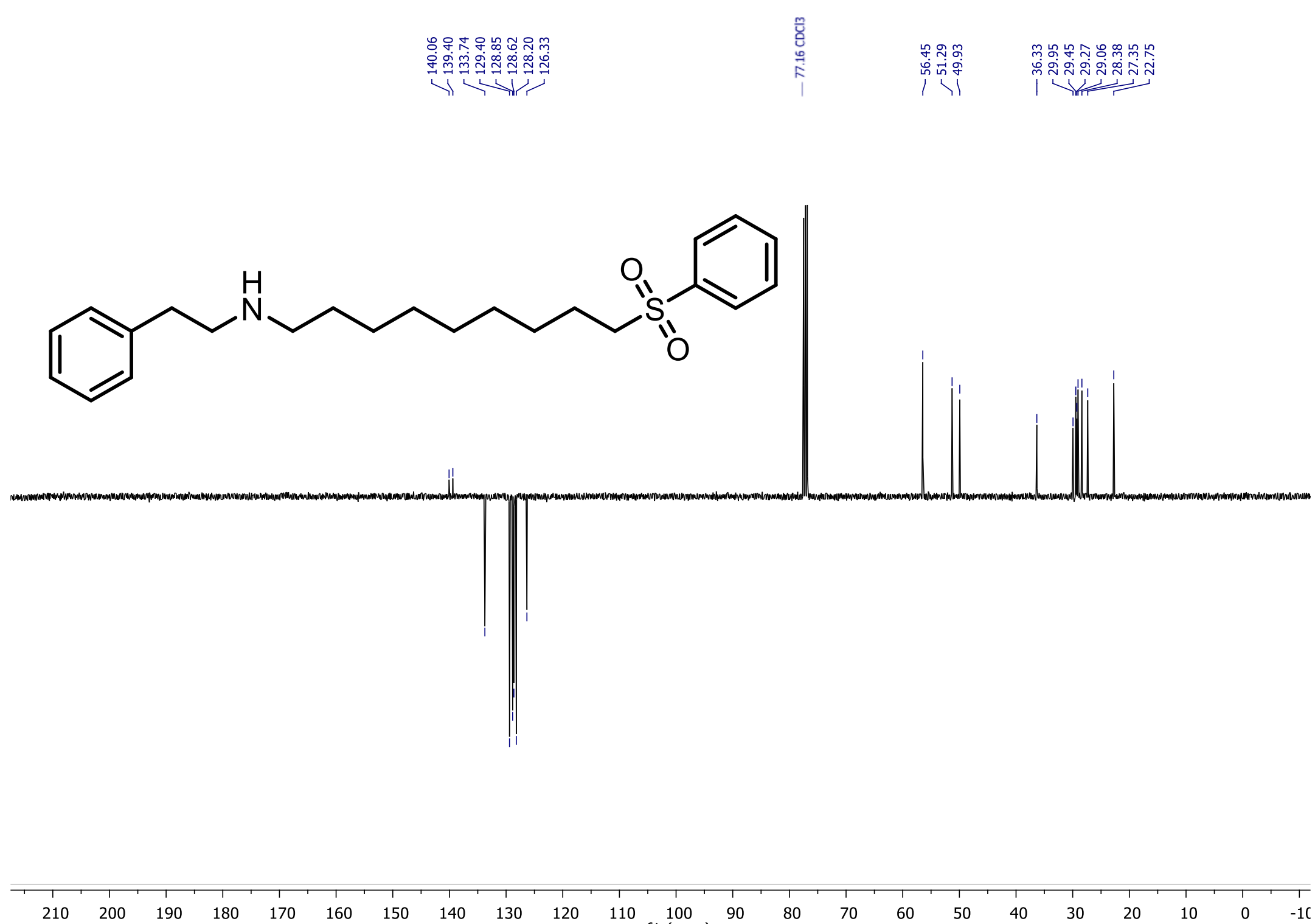

***N*,*N*-Diethyl-*N*[11]-phenethylundecane-1,11-diamine (1.29)**

^{1}H NMR (400 MHz, $CDCl_3$)

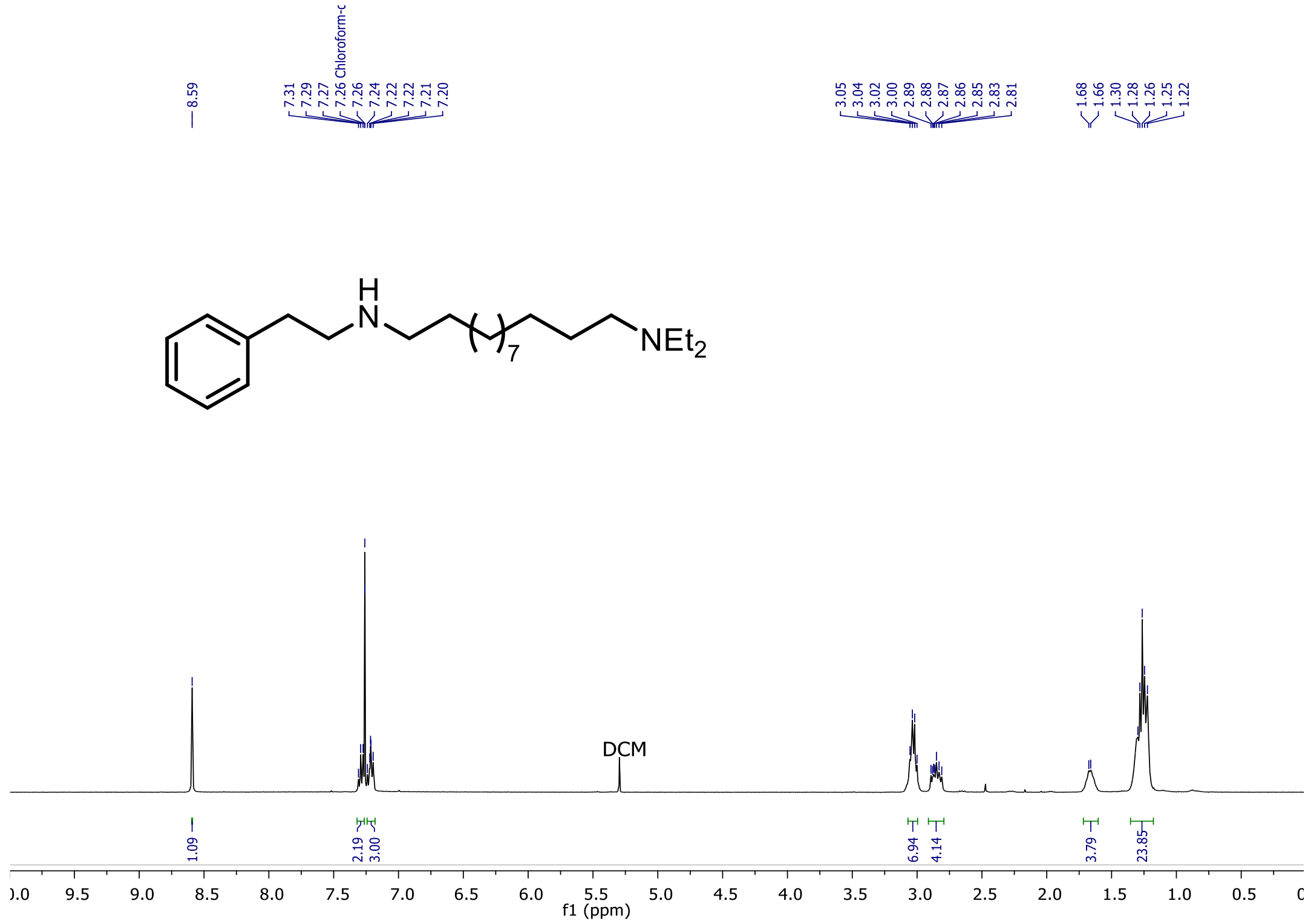

^{13}C NMR (101 MHz, $CDCl_3$)

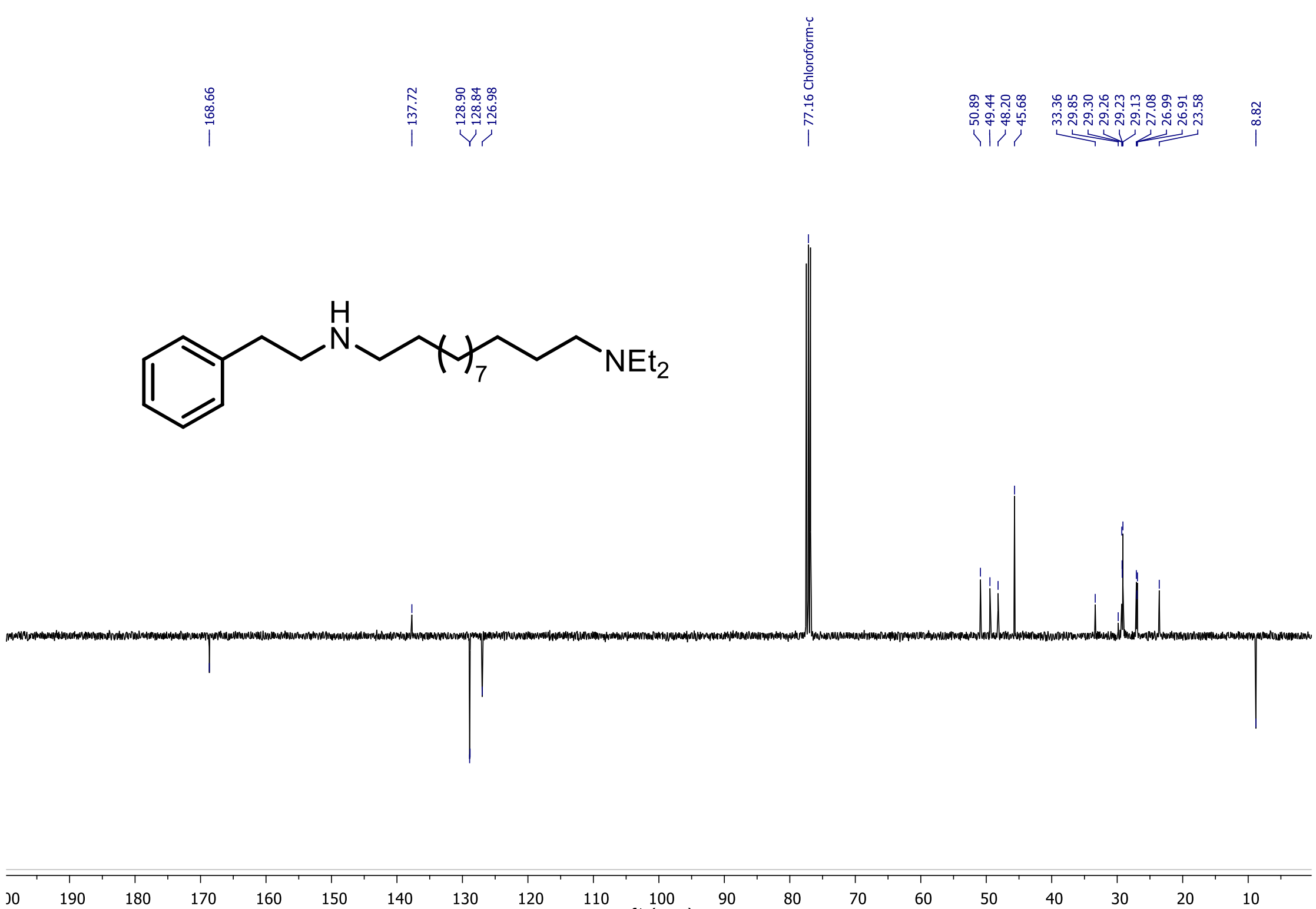

7-(Phenethylamino)heptan-1-ol (1.30)
^{1}H NMR (400 MHz, CDCl$_3$)

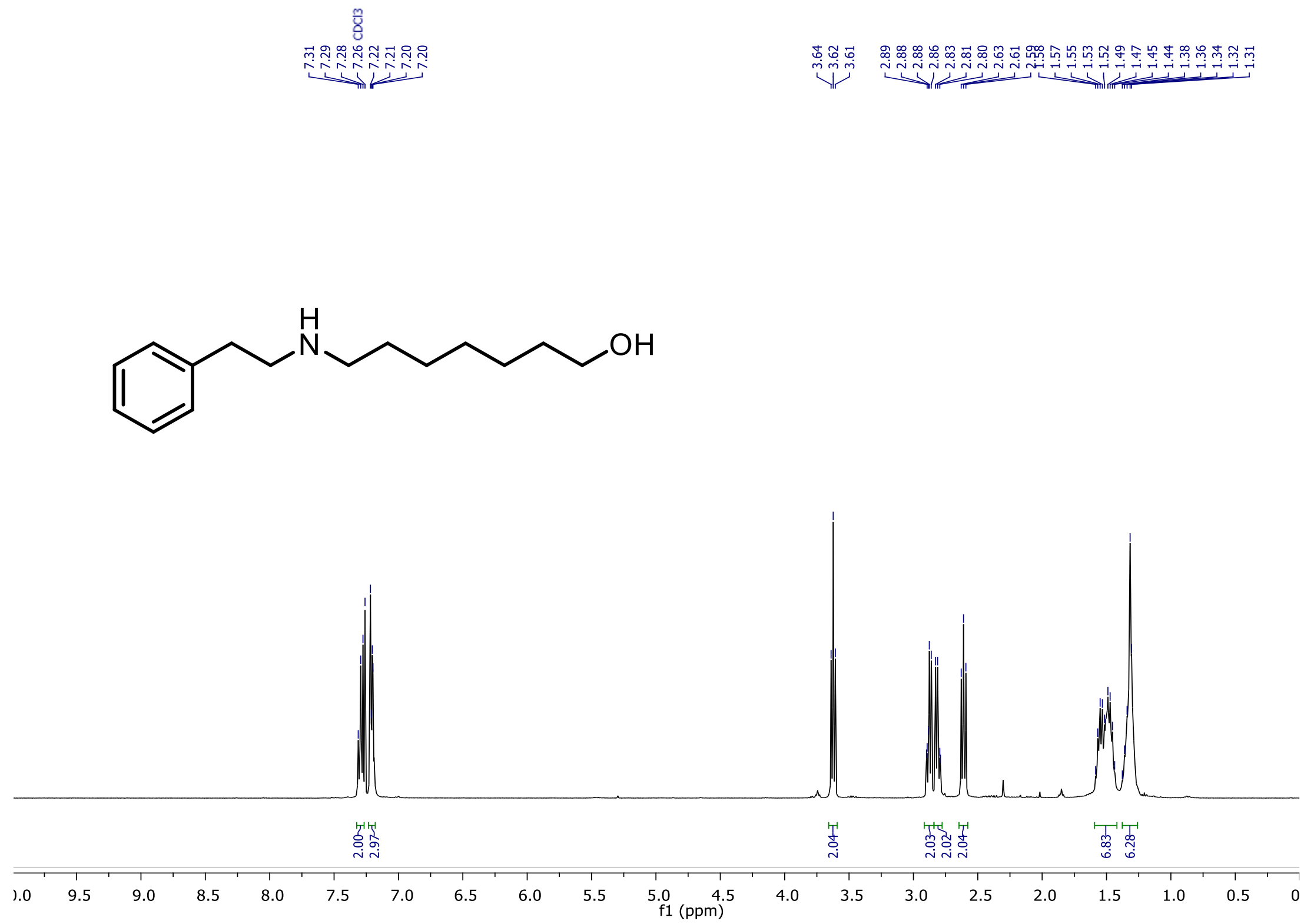

^{13}C NMR (101 MHz, CDCl$_3$)

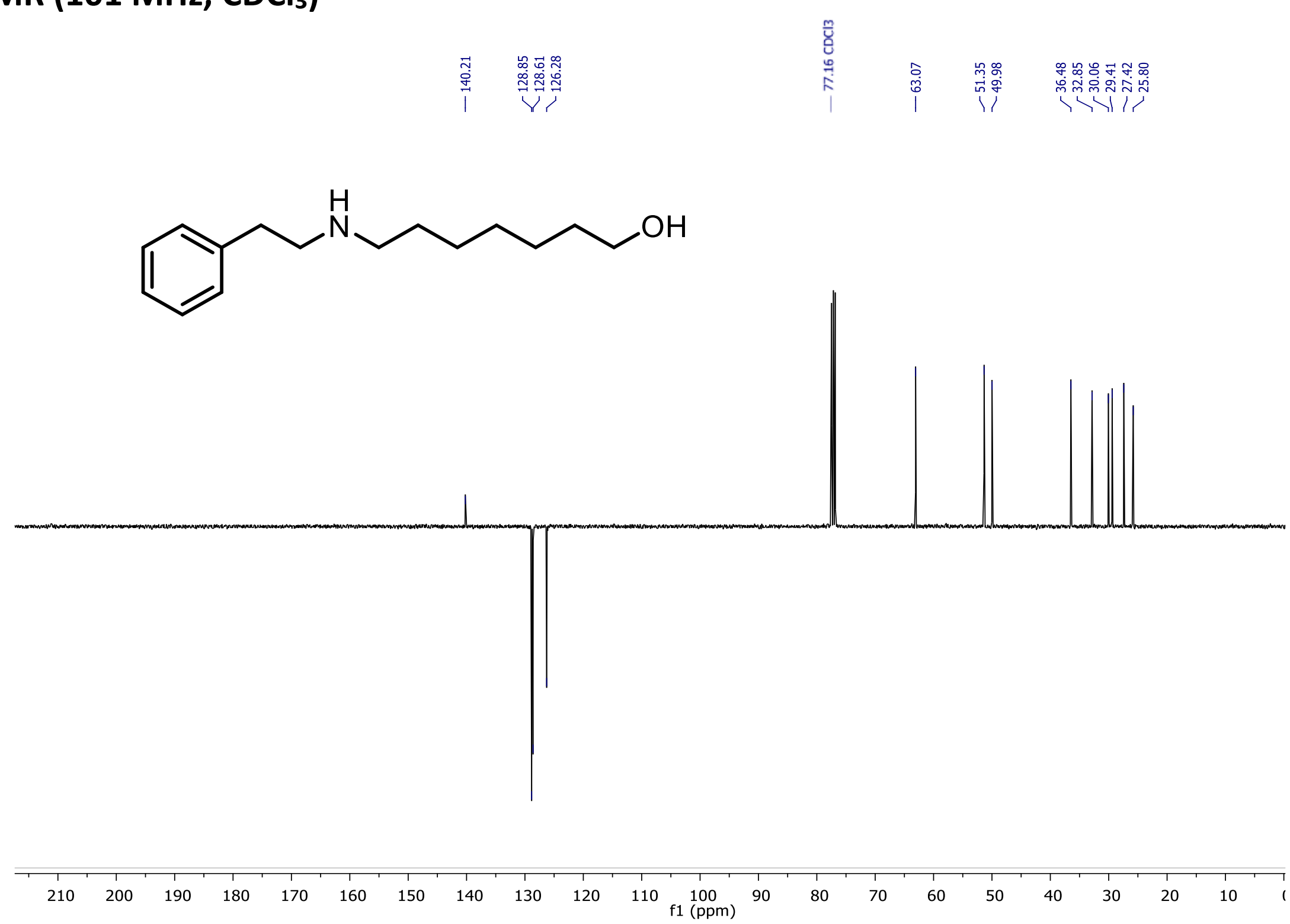

***N,N*-Dimethyl-12-(phenethylamino)dodecanamide (1.31)**

^{1}H NMR (400 MHz, $CDCl_3$)

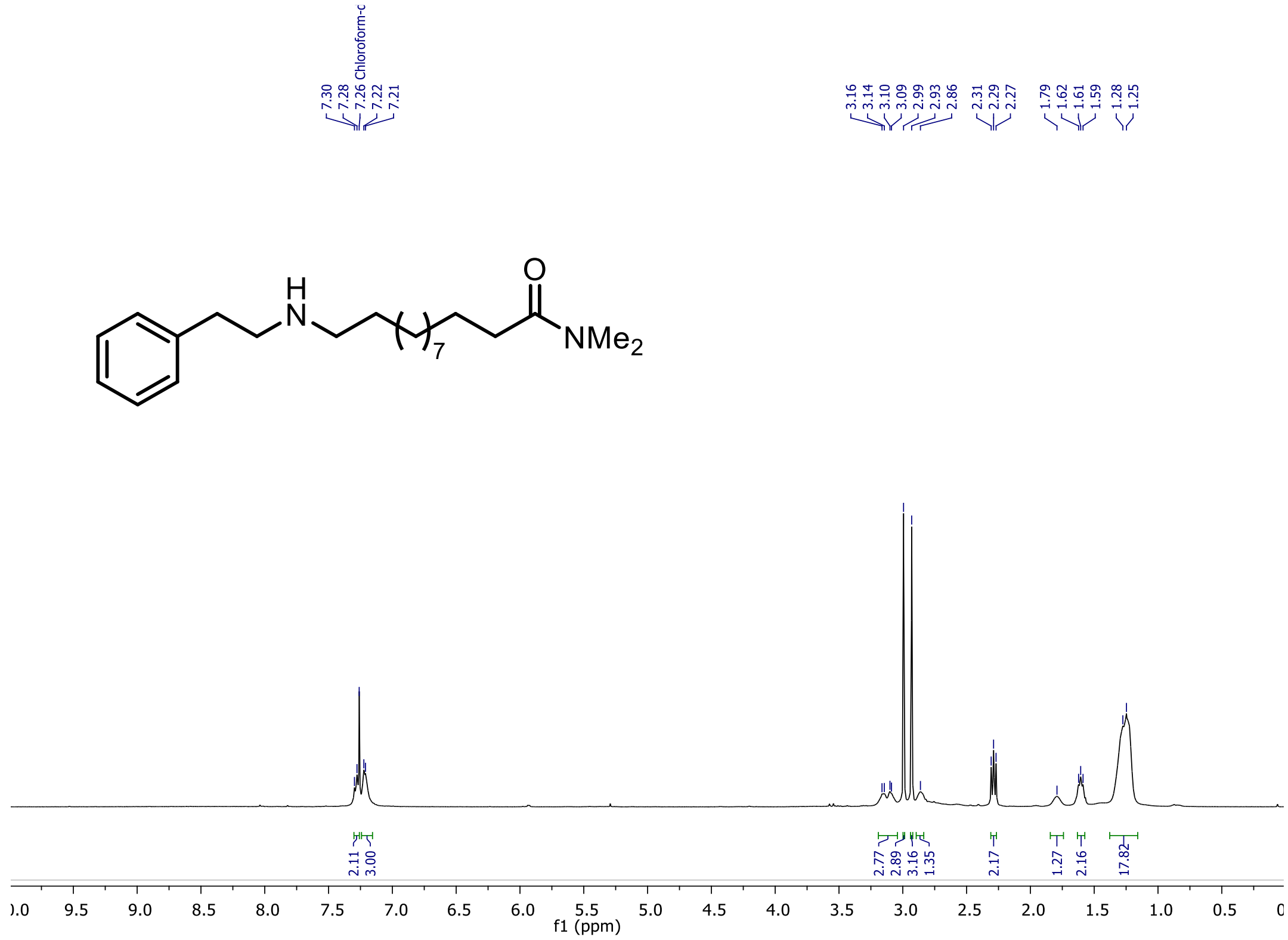

^{13}C NMR (101 MHz, $CDCl_3$)

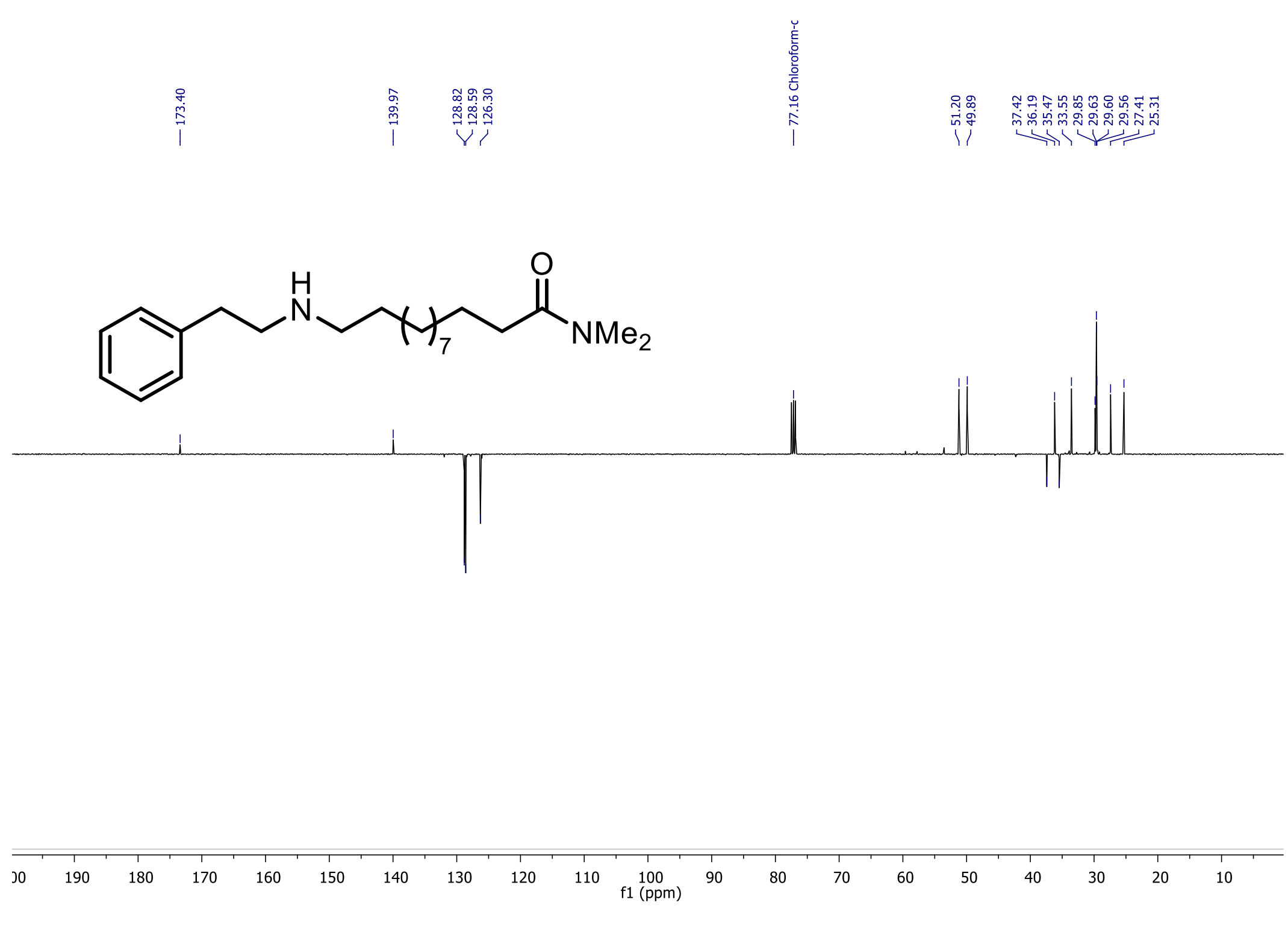

8-(Phenethylamino)-1-phenyloctan-1-one (1.32)

^{1}H NMR (600 MHz, $CDCl_3$)

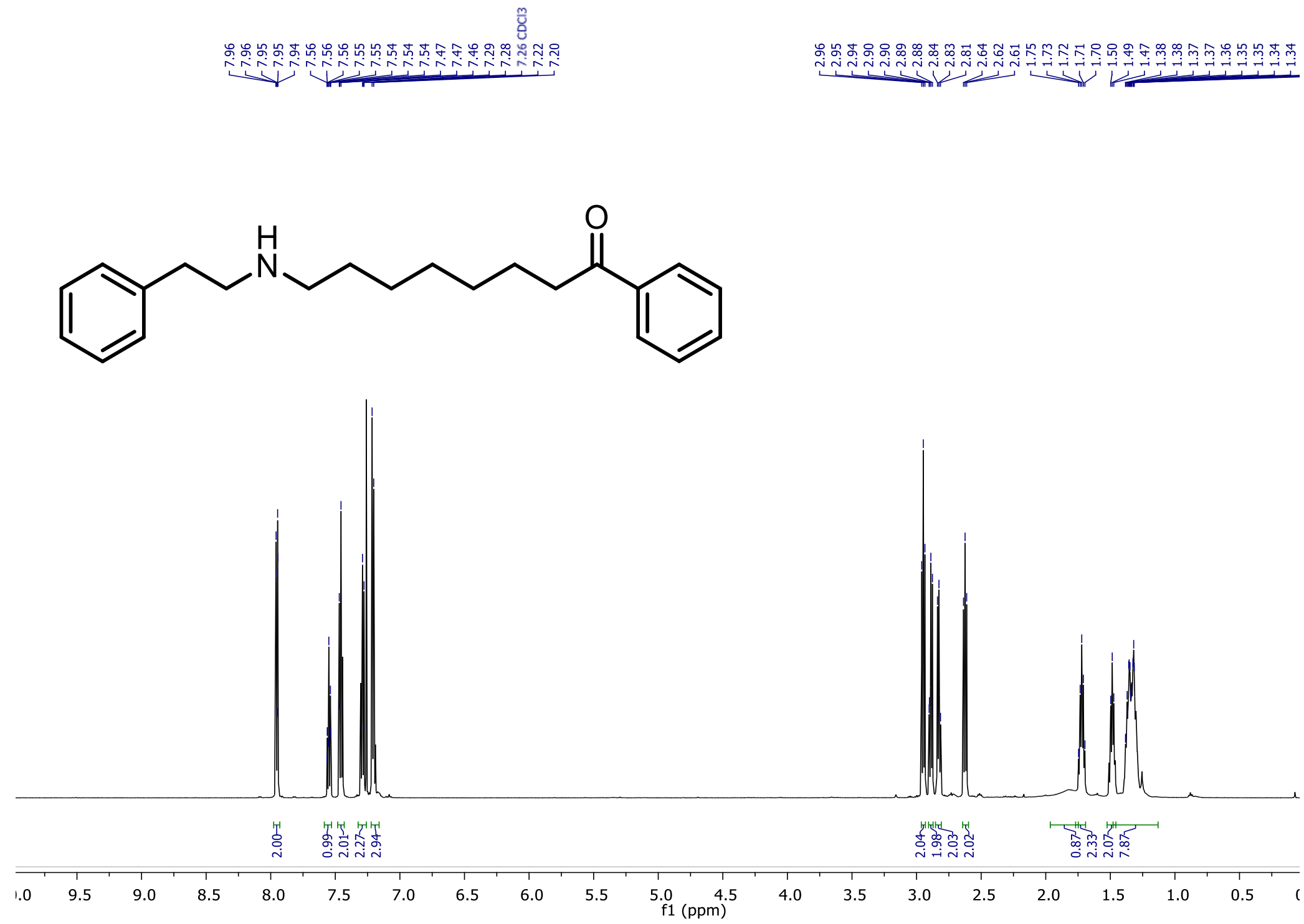

^{13}C NMR (151 MHz, $CDCl_3$)

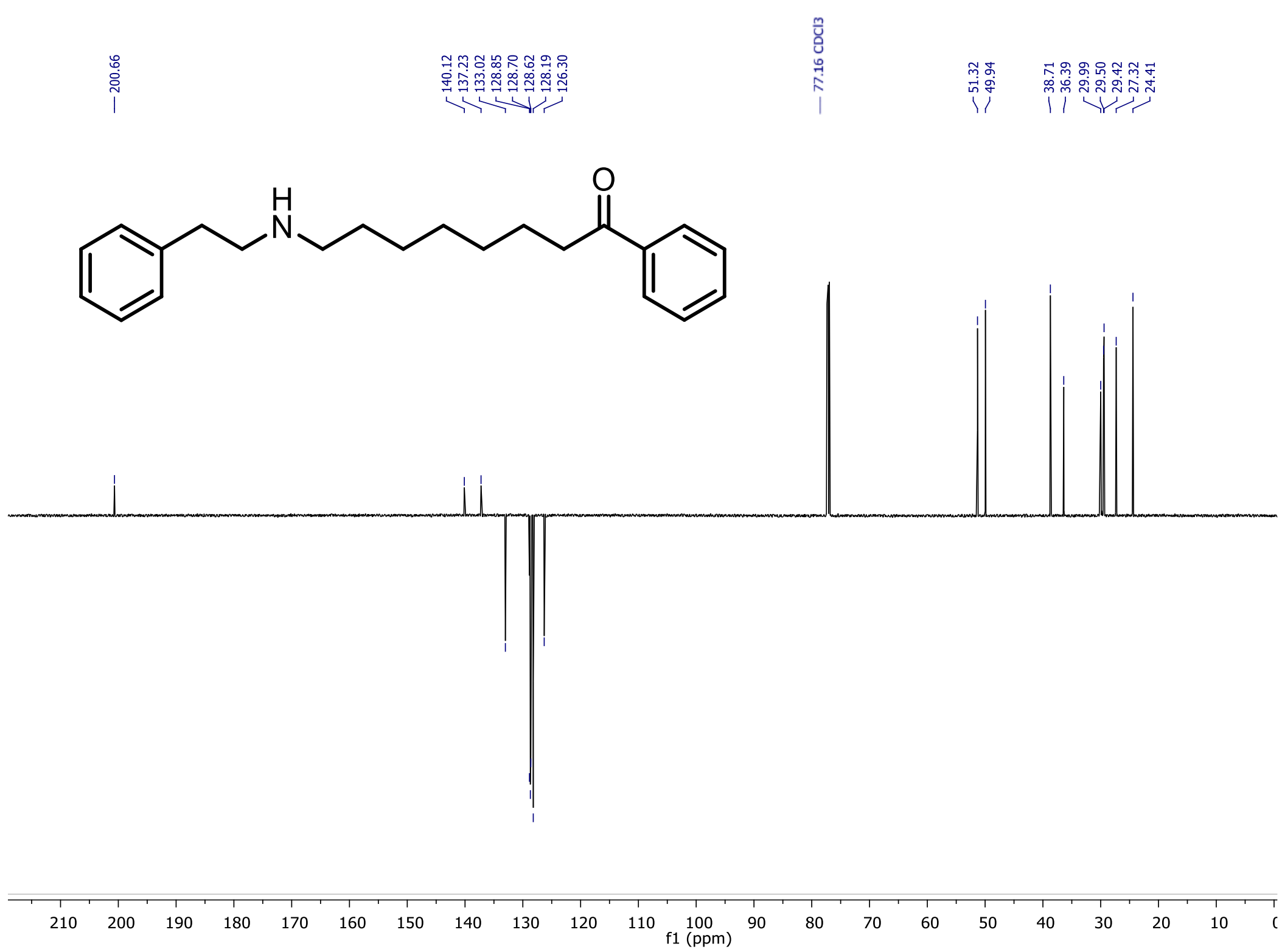

3-(3-(Phenethylamino)propyl)benzaldehyde(1.33)

^{1}H NMR (600 MHz, CDCl$_3$)

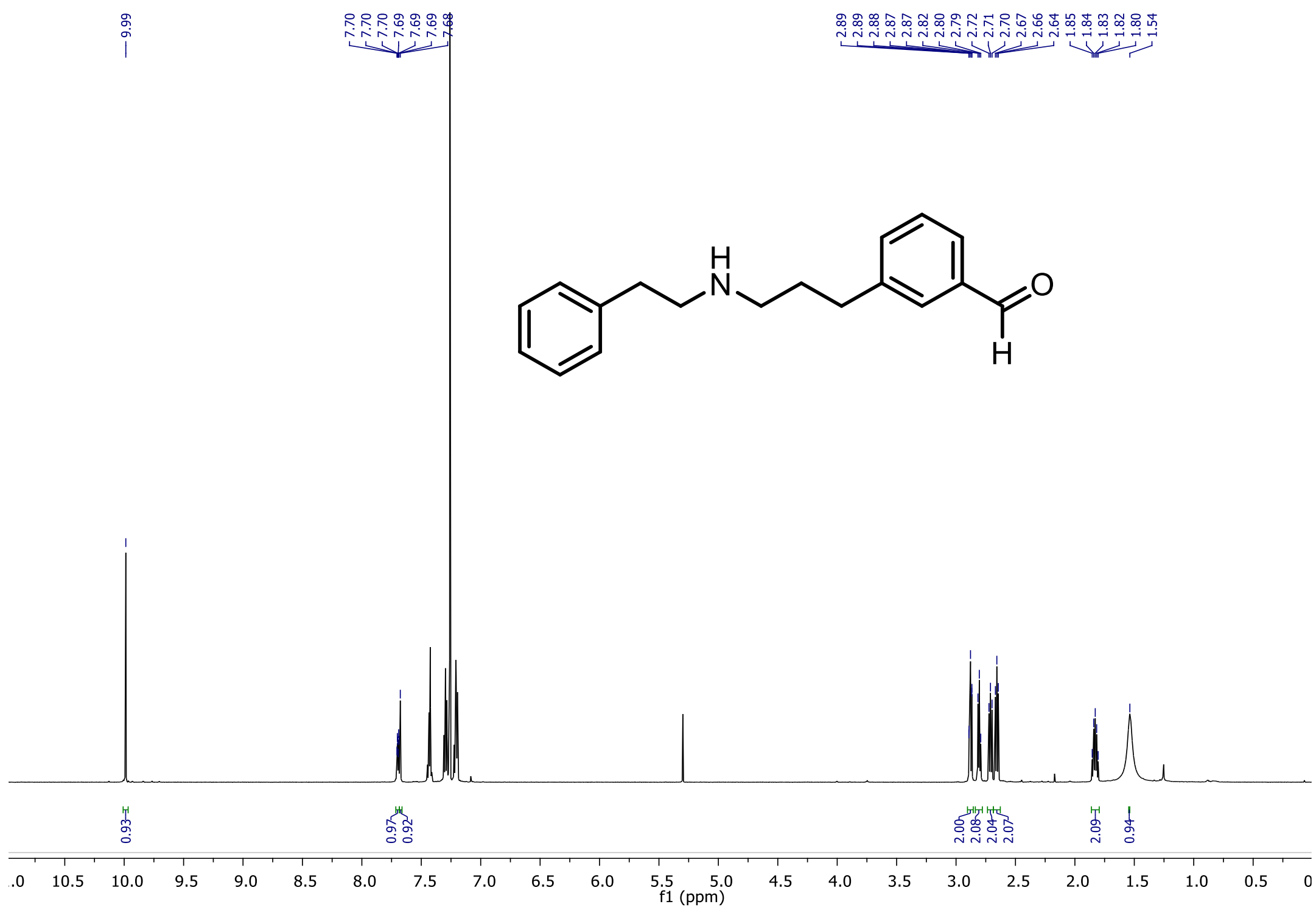

^{13}C NMR (151 MHz, CDCl$_3$)

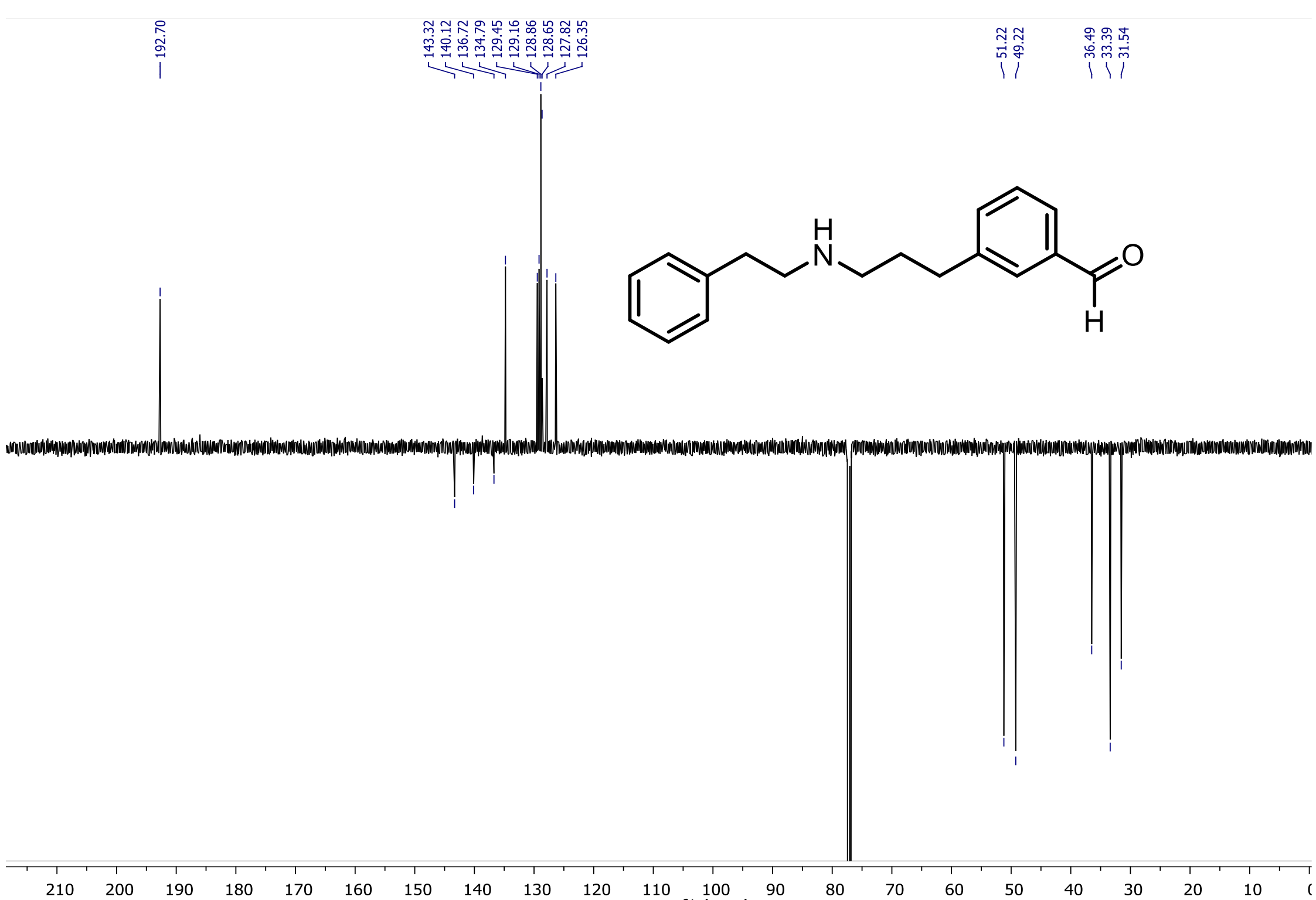

3-(Benzo[*b*]thiophen-3-yl)-*N*-phenethylpropan-1-amine(1.34)

^{1}H NMR (600 MHz, CDCl$_3$)

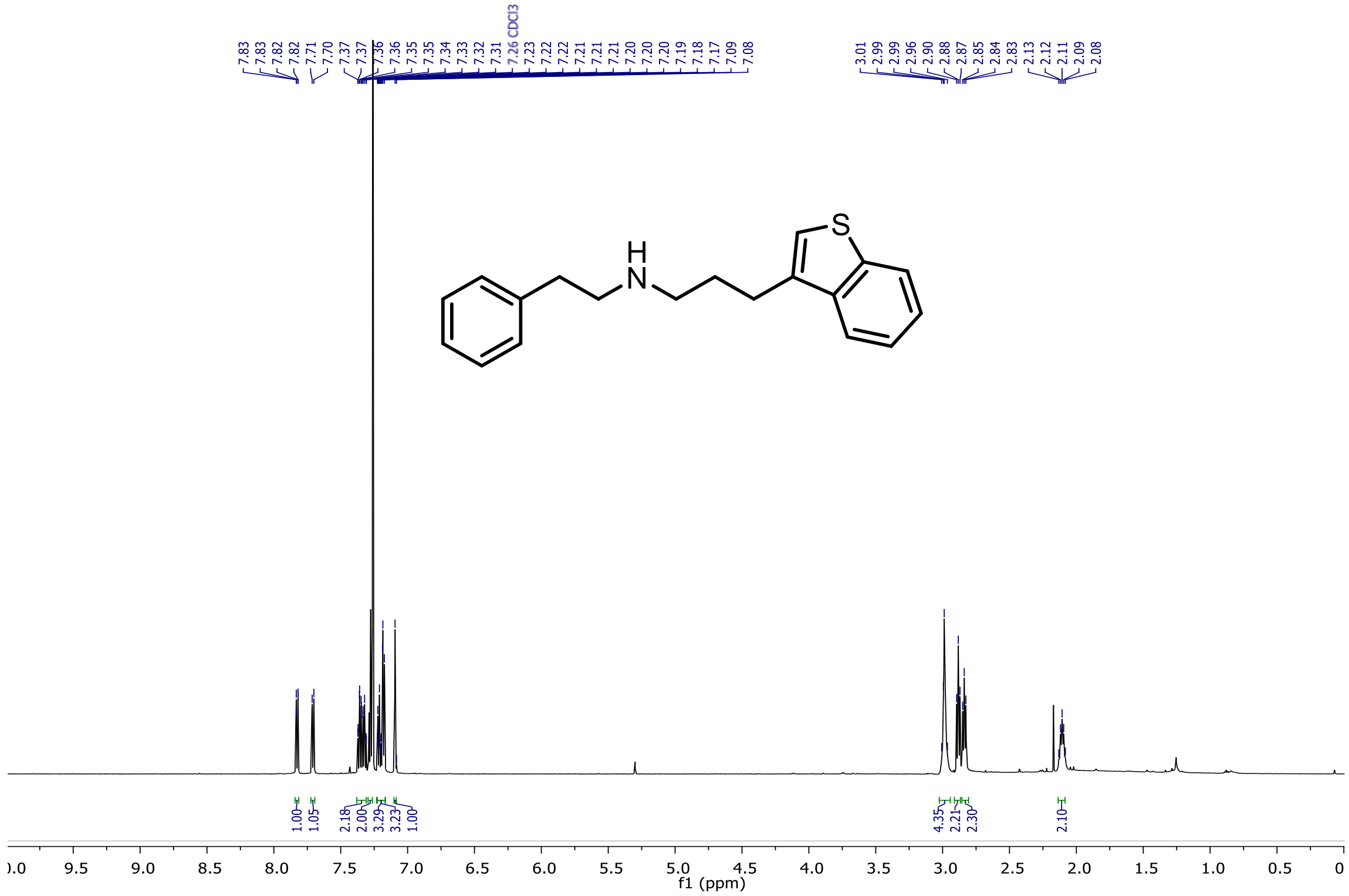

^{13}C NMR (151 MHz, CDCl$_3$)

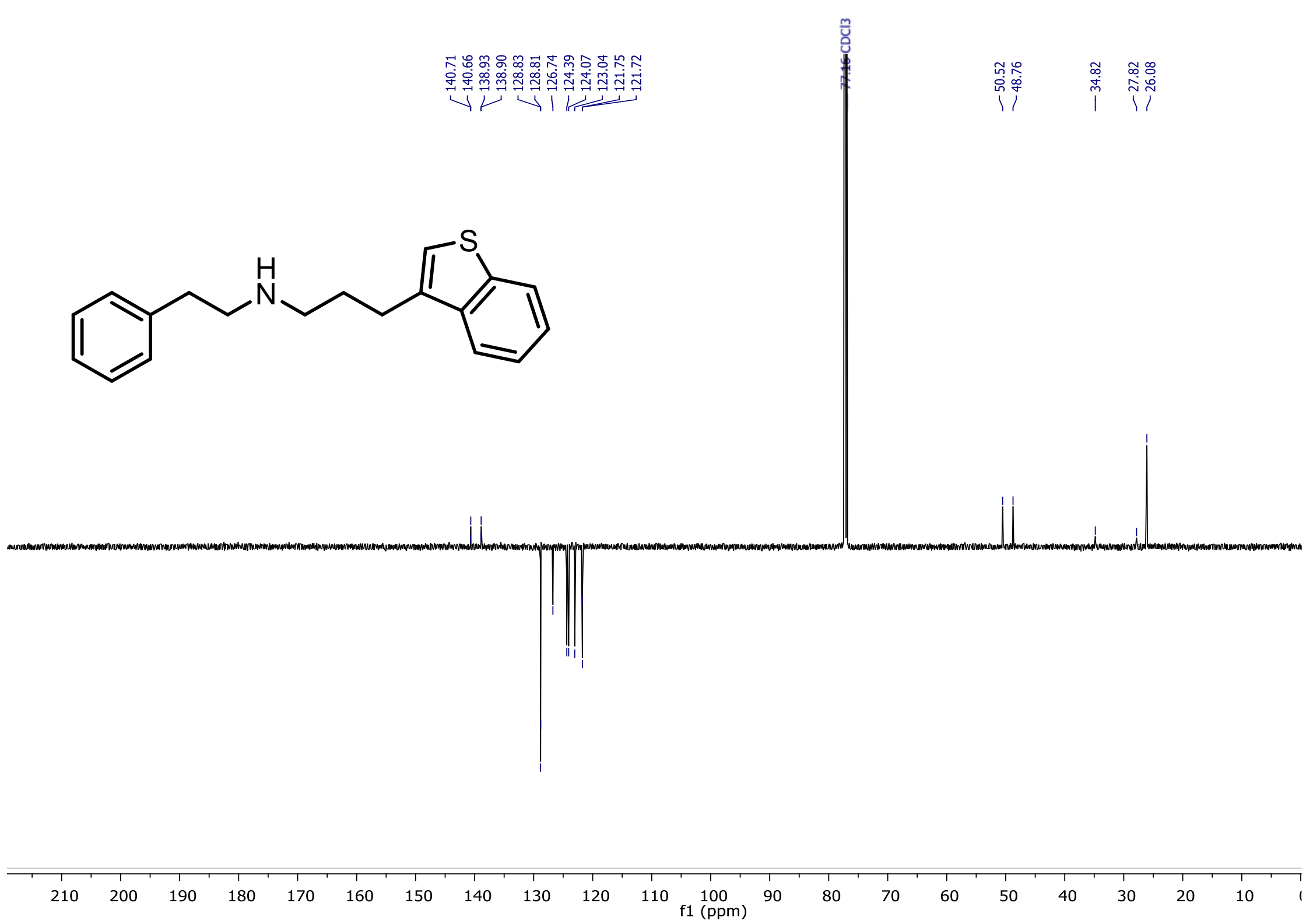

3-(Benzofuran-3-yl)-*N*-phenethylpropan-1-amine (1.35)

^{1}H NMR (600 MHz, $CDCl_3$)

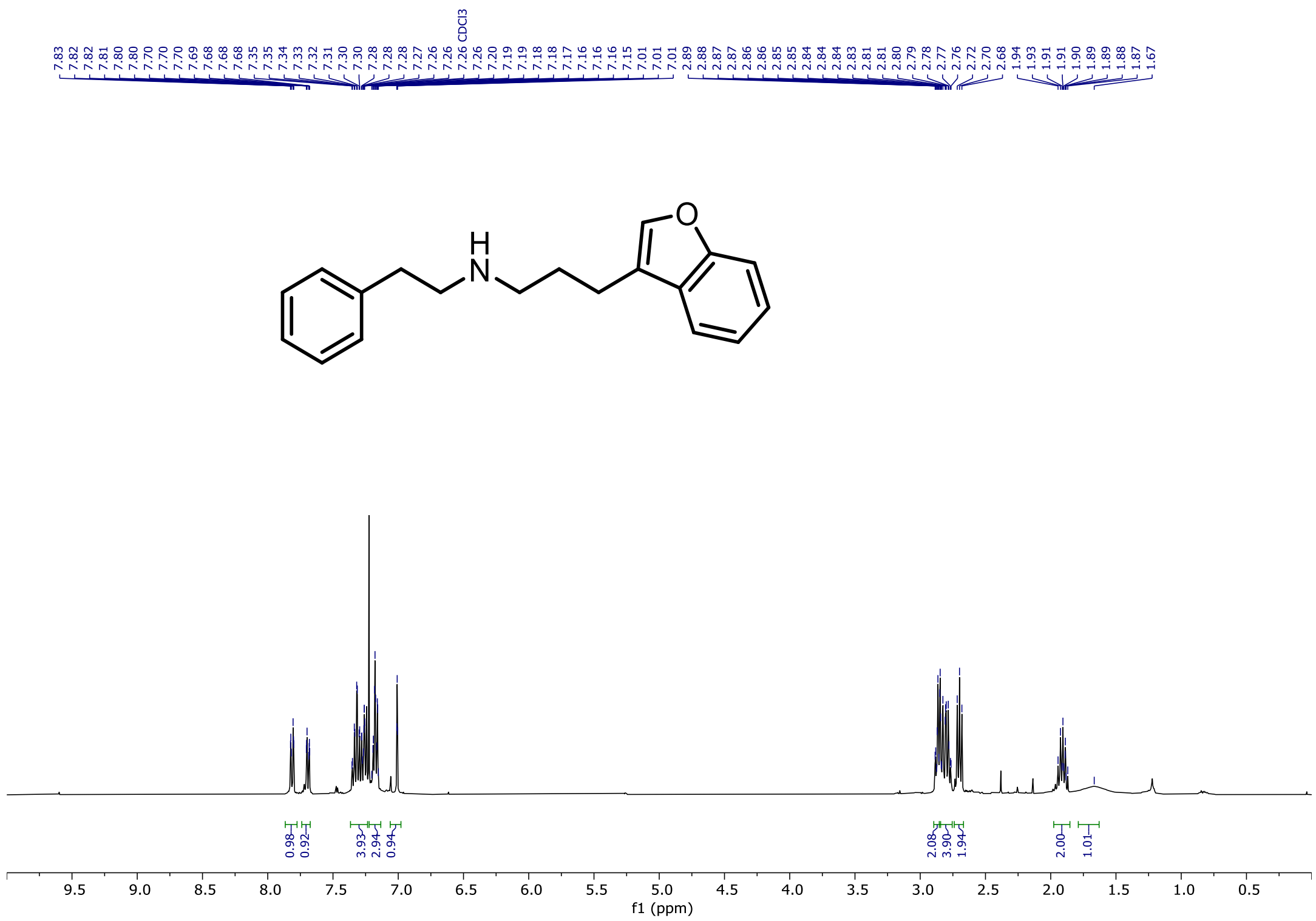

^{13}C NMR (151 MHz, $CDCl_3$)

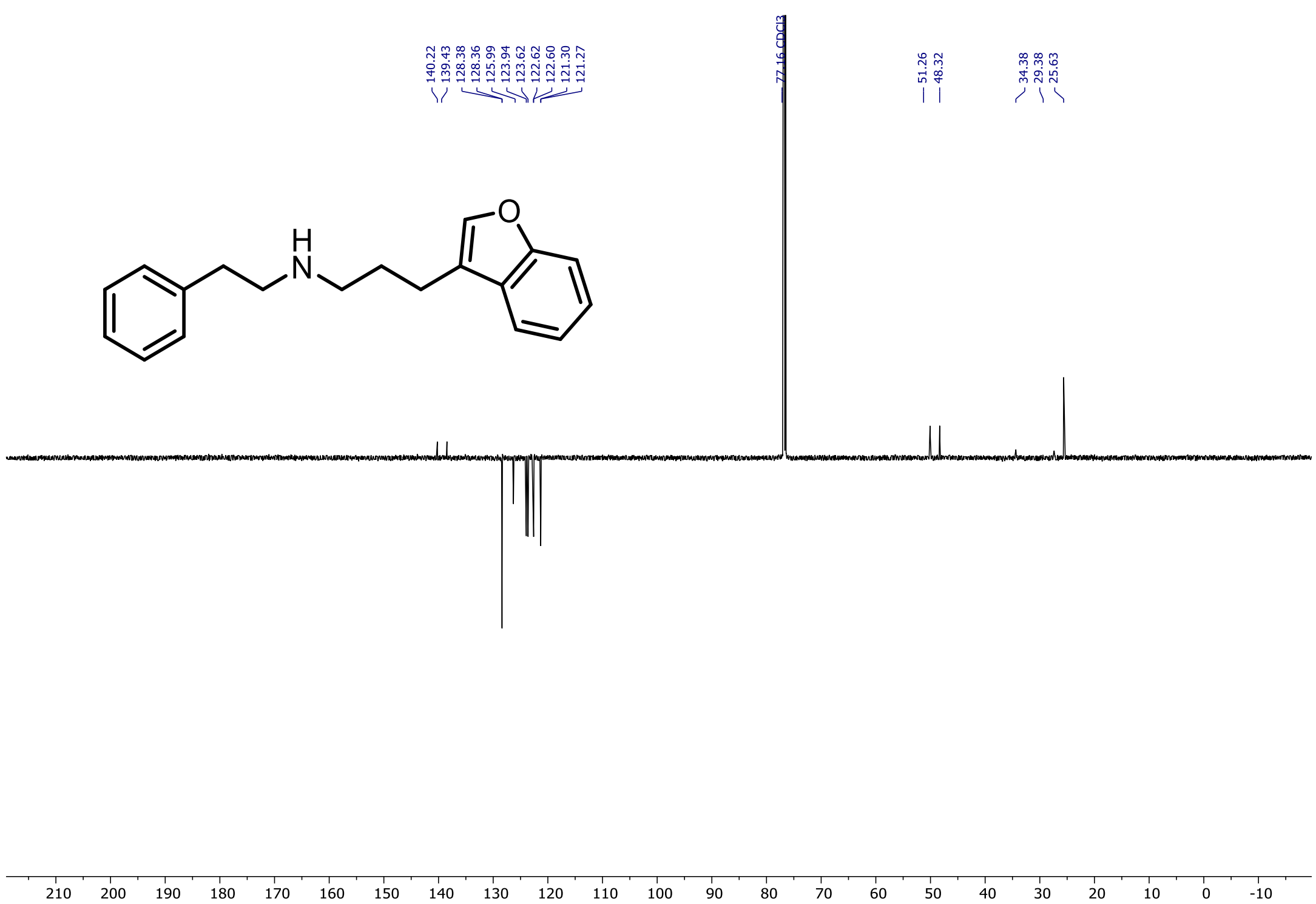

3-Phenyl-*N*-(1-phenylethyl)propan-1-amine (1.36)

^{1}H NMR (700 MHz, $CDCl_3$)

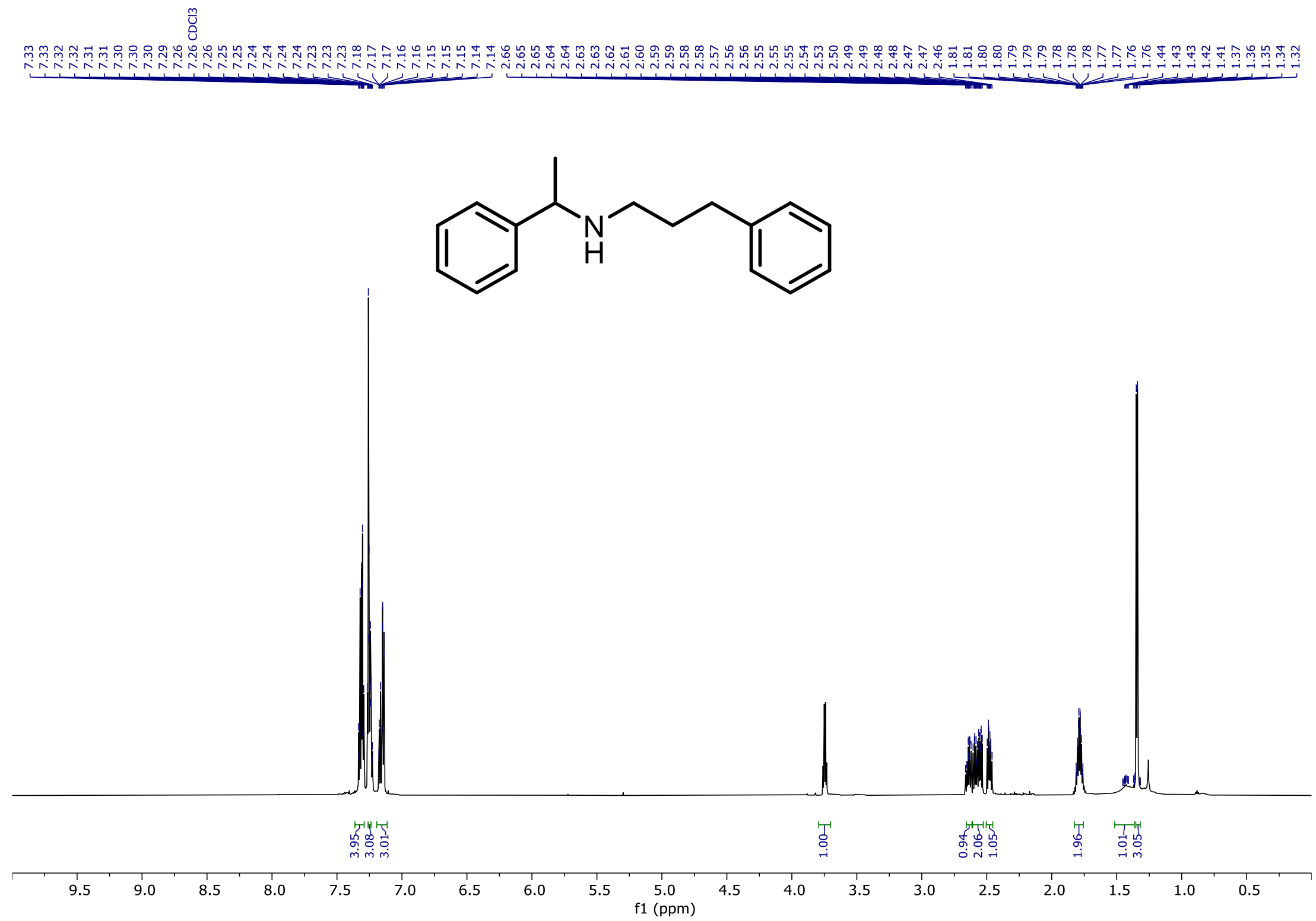

^{13}C NMR (176 MHz, $CDCl_3$)

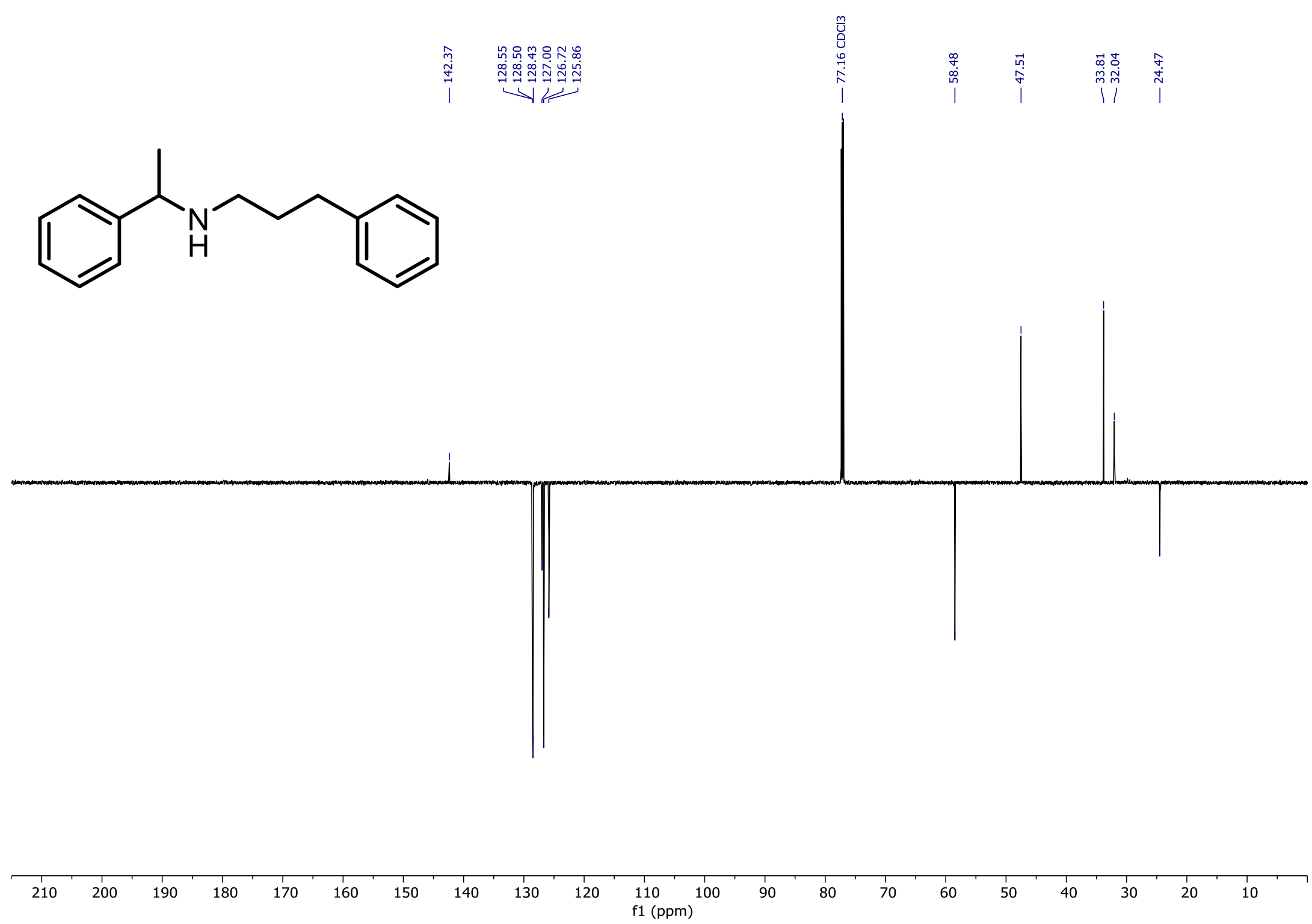

3-(4-Bromophenyl)-*N*-phenethylpropan-1-amine (1.37)

^{1}H NMR (600 MHz, $CDCl_3$)

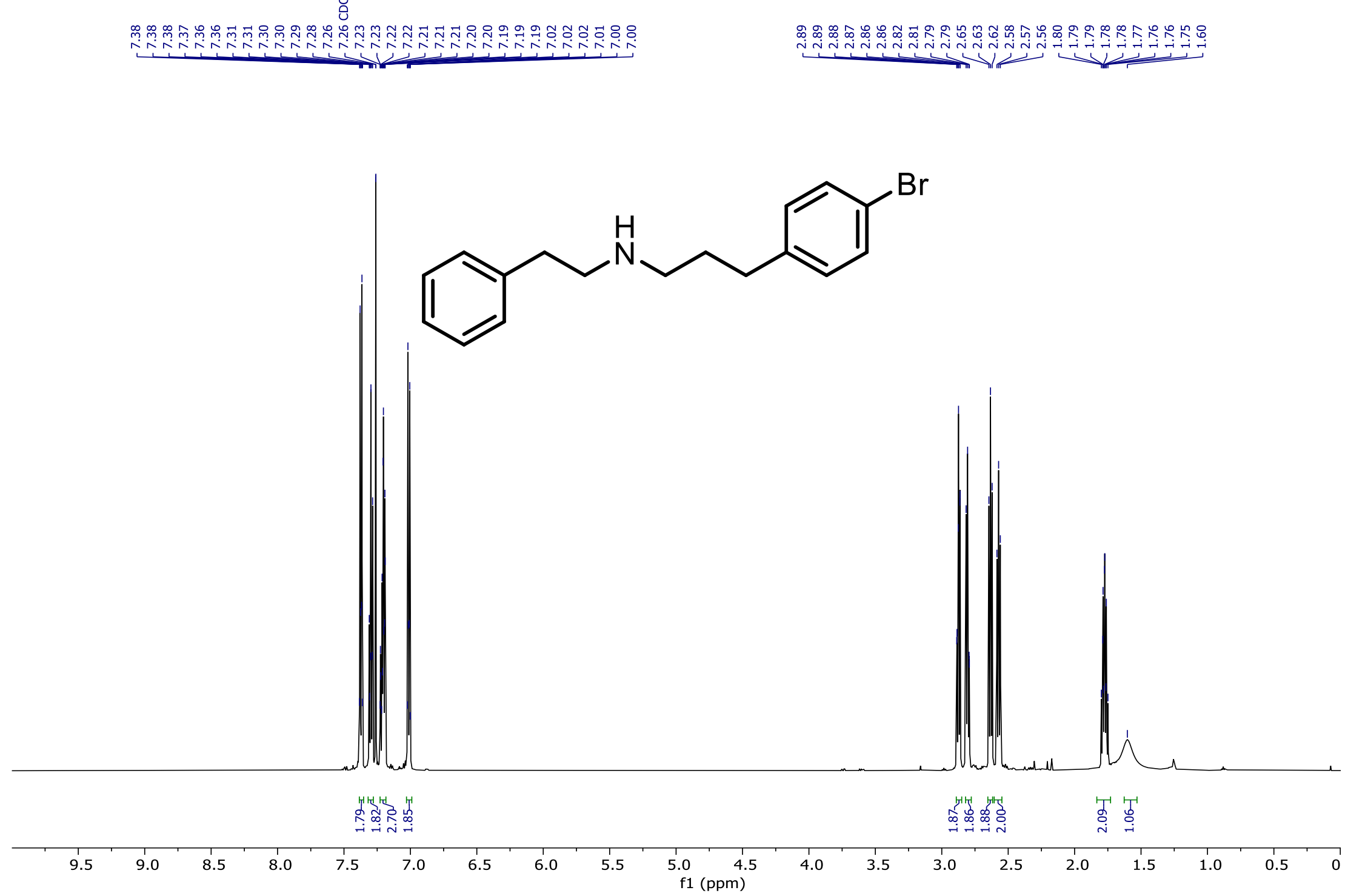

^{13}C NMR (151 MHz, $CDCl_3$)

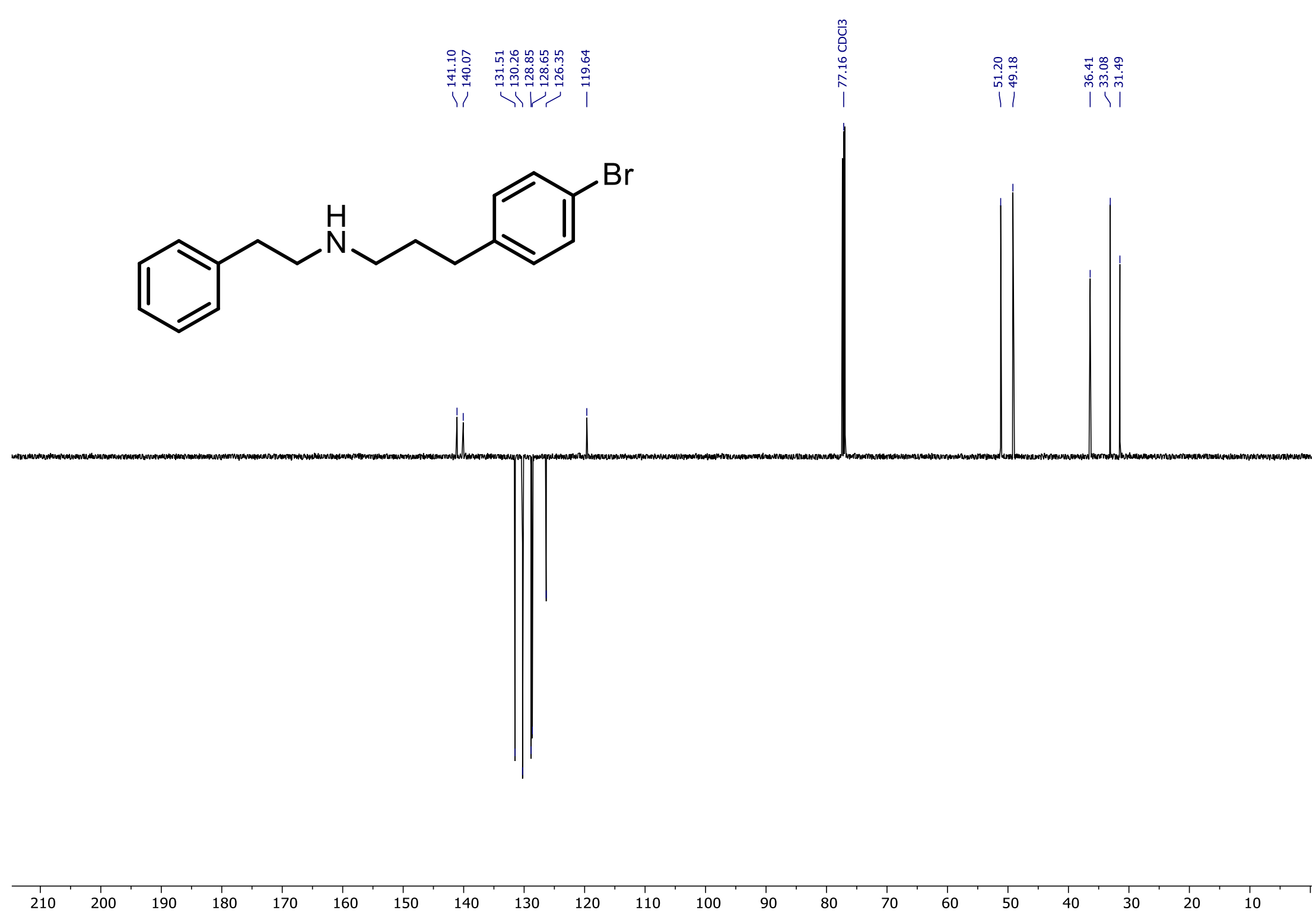

3-(4-Chlorophenyl)-*N*-phenethylpropan-1-amine (1.38)

^{1}H NMR (600 MHz, CDCl$_3$)

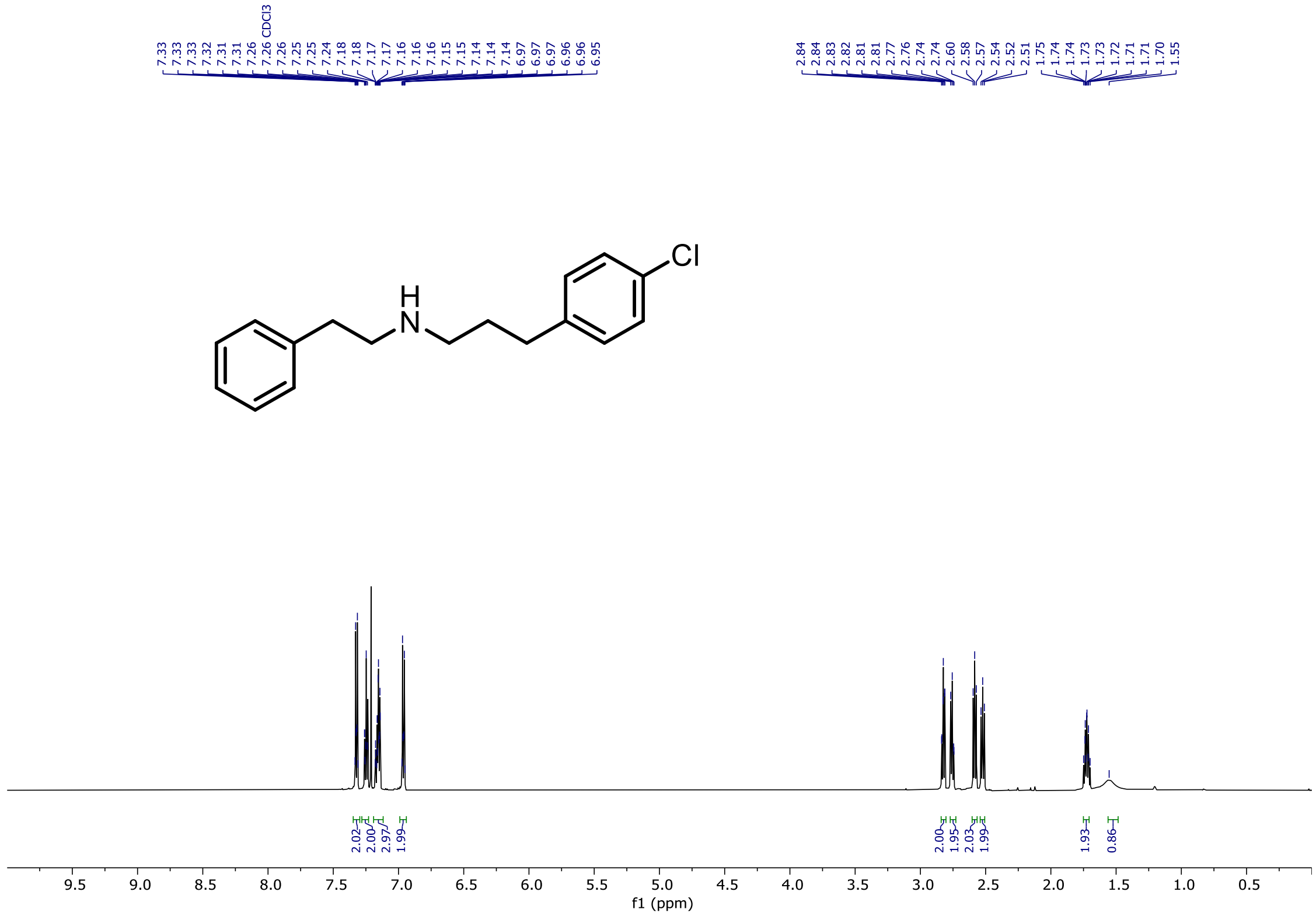

^{13}C NMR (151 MHz, CDCl$_3$)

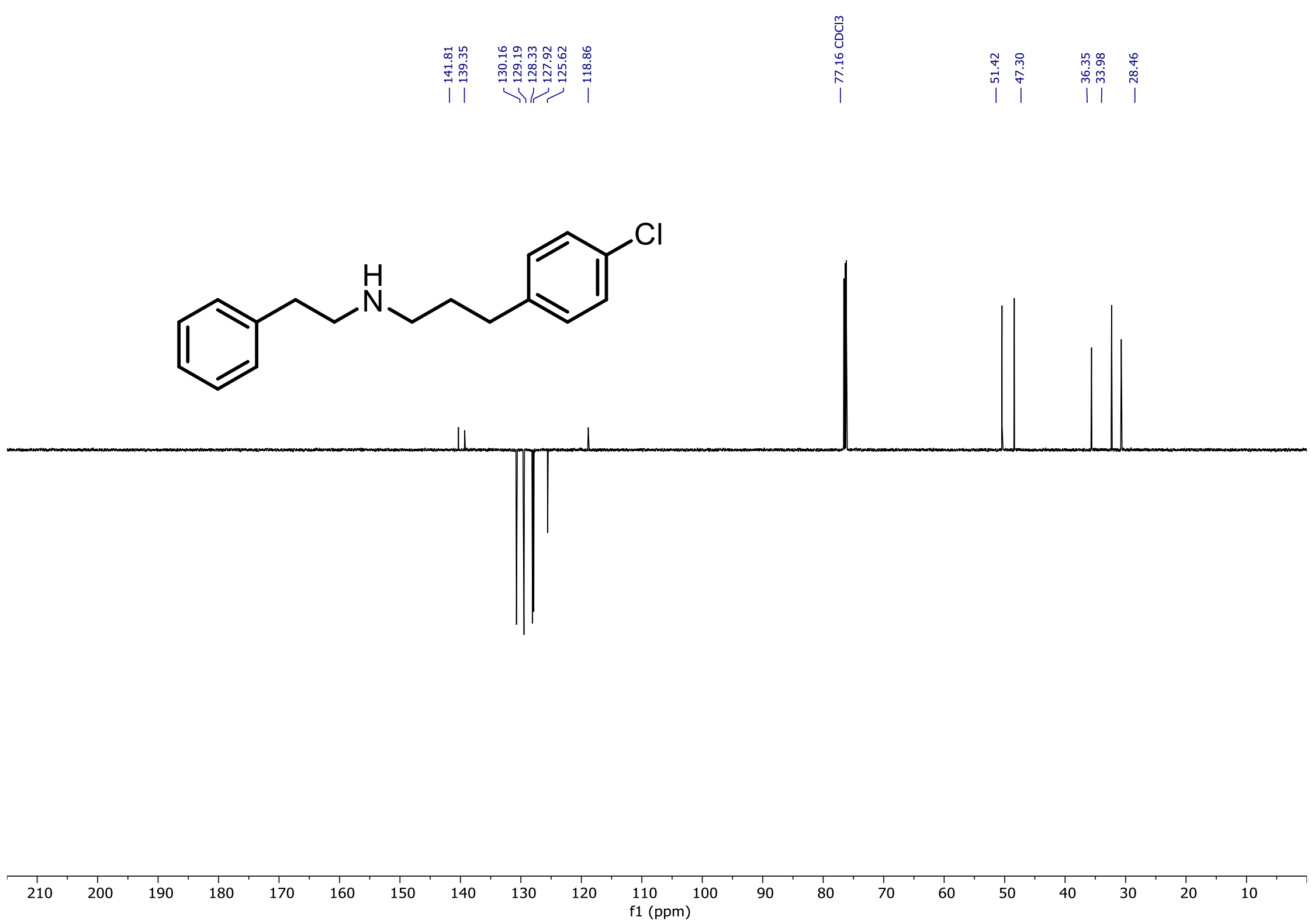

2-(3-((*tert*-Butoxycarbonyl)(phenethyl)amino)propyl) benzoic acid(1.39)

^{1}H NMR (400 MHz, $CDCl_3$)

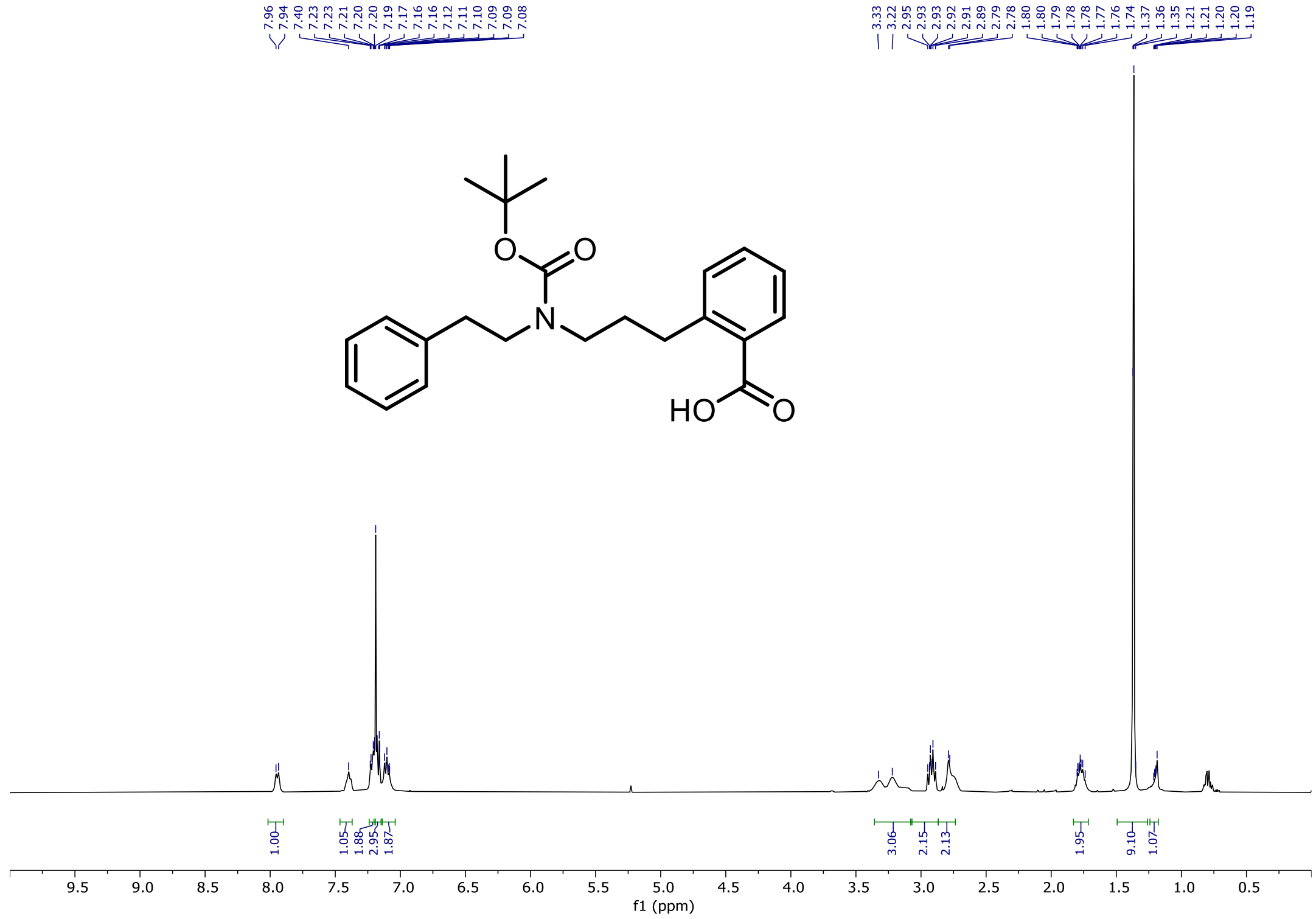

^{13}C NMR (101 MHz, $CDCl_3$)

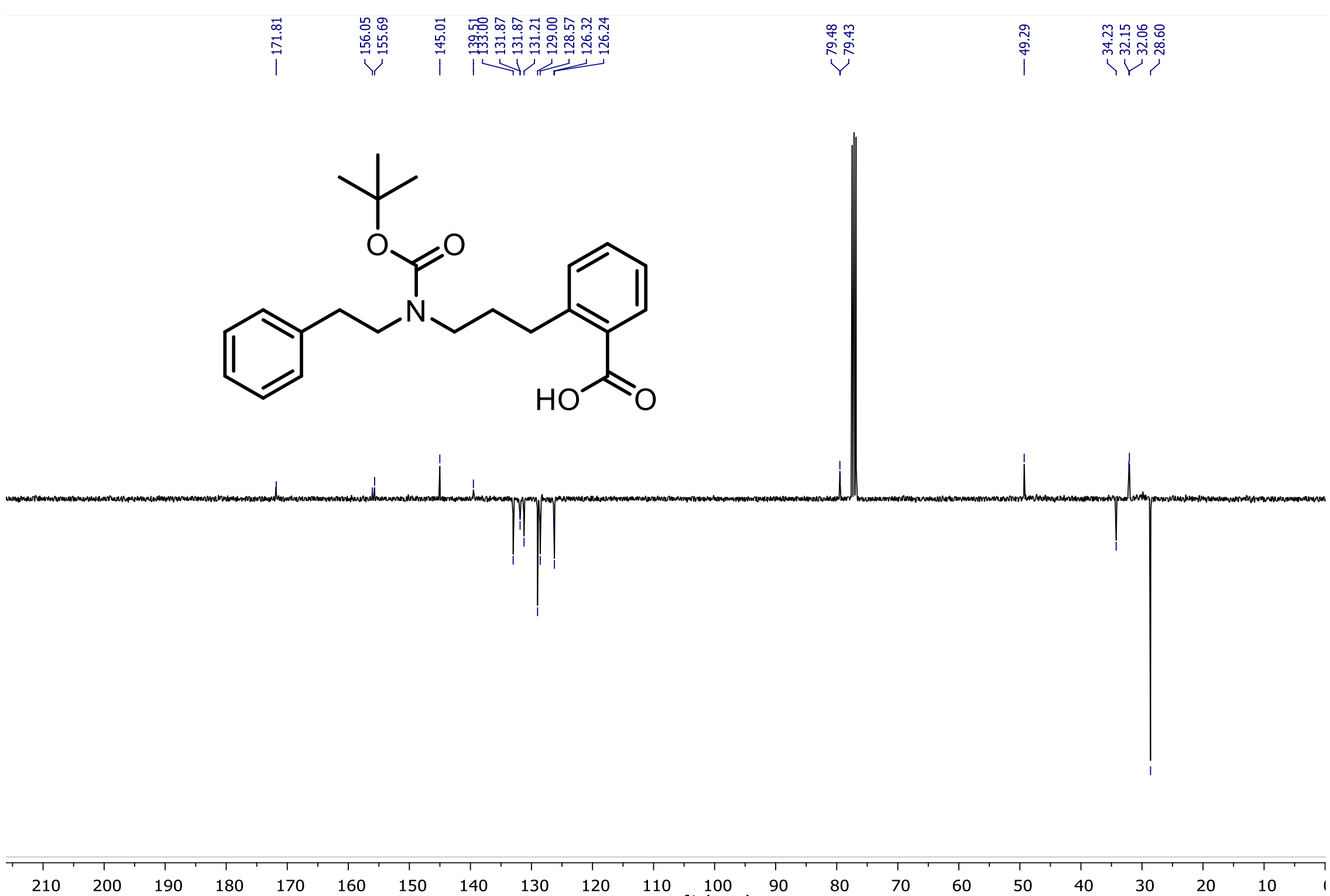

2-Methyl-*N*-phenethyl-3-phenylpropan-1-amine (1.40)
^{1}H NMR (600 MHz, $CDCl_3$)

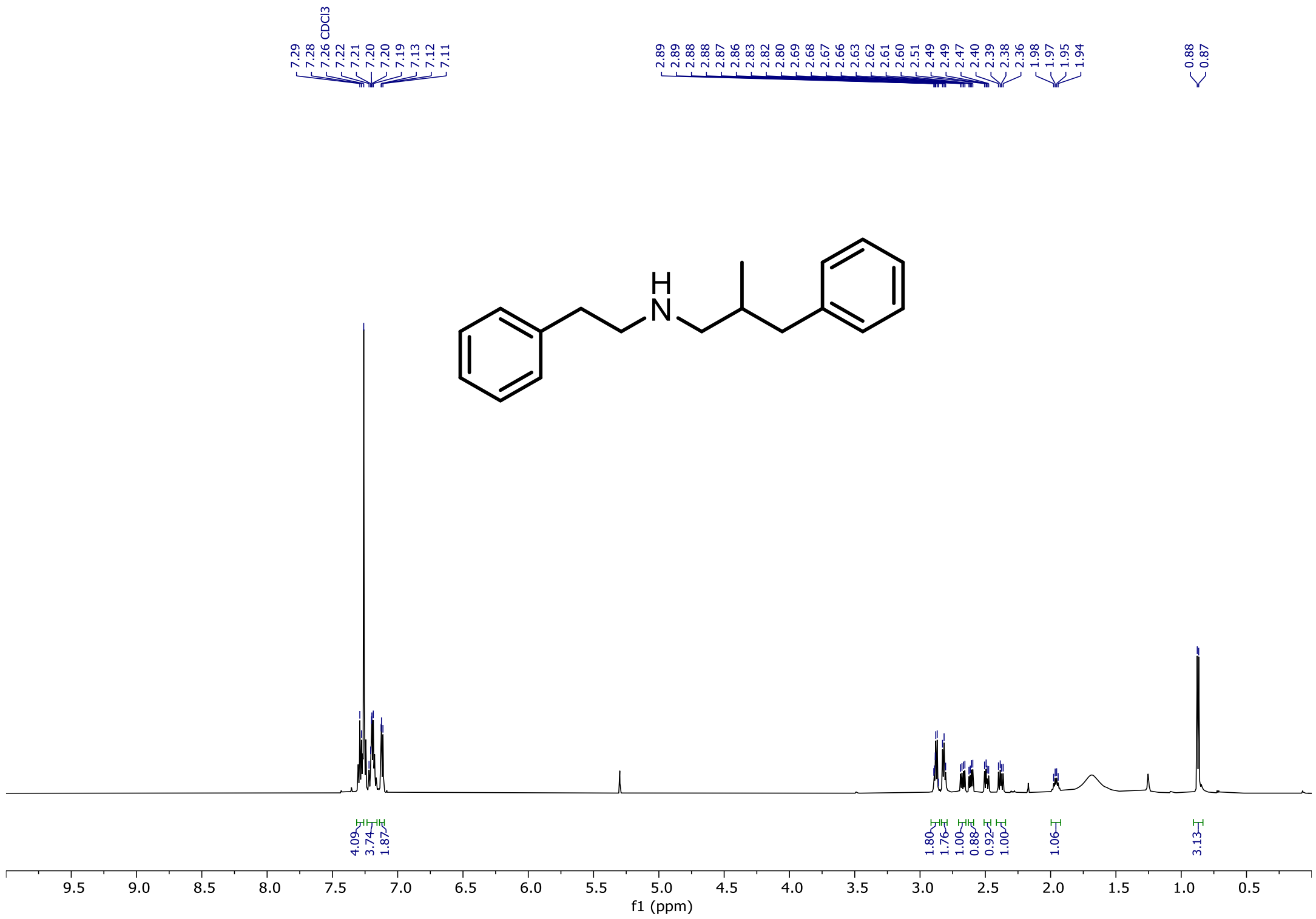

^{13}C NMR (151 MHz, $CDCl_3$)

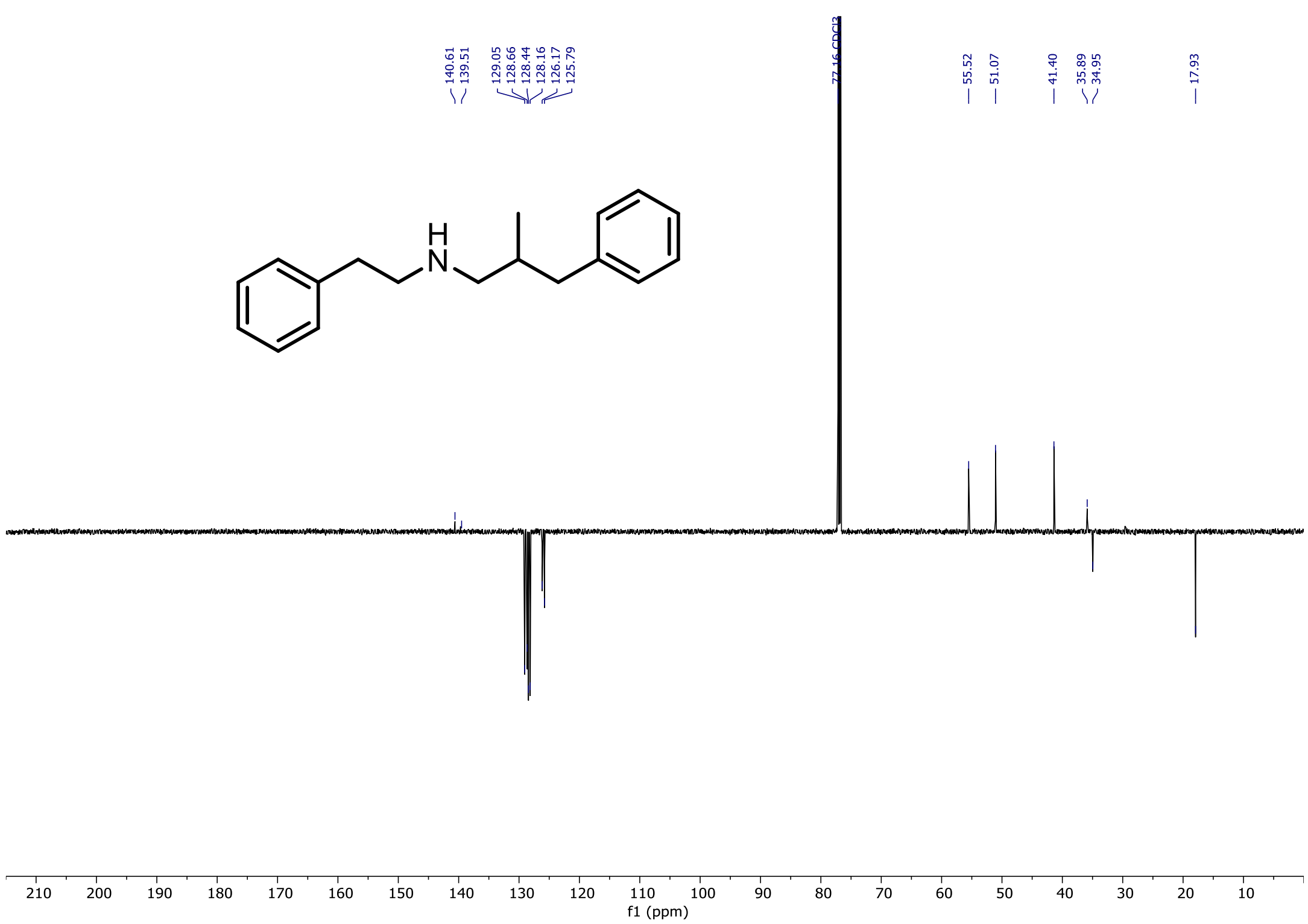

3-(4-Methoxyphenyl)-*N*-phenethylpropan-1-amine (1.41)

^{1}H NMR (700 MHz, CDCl$_3$)

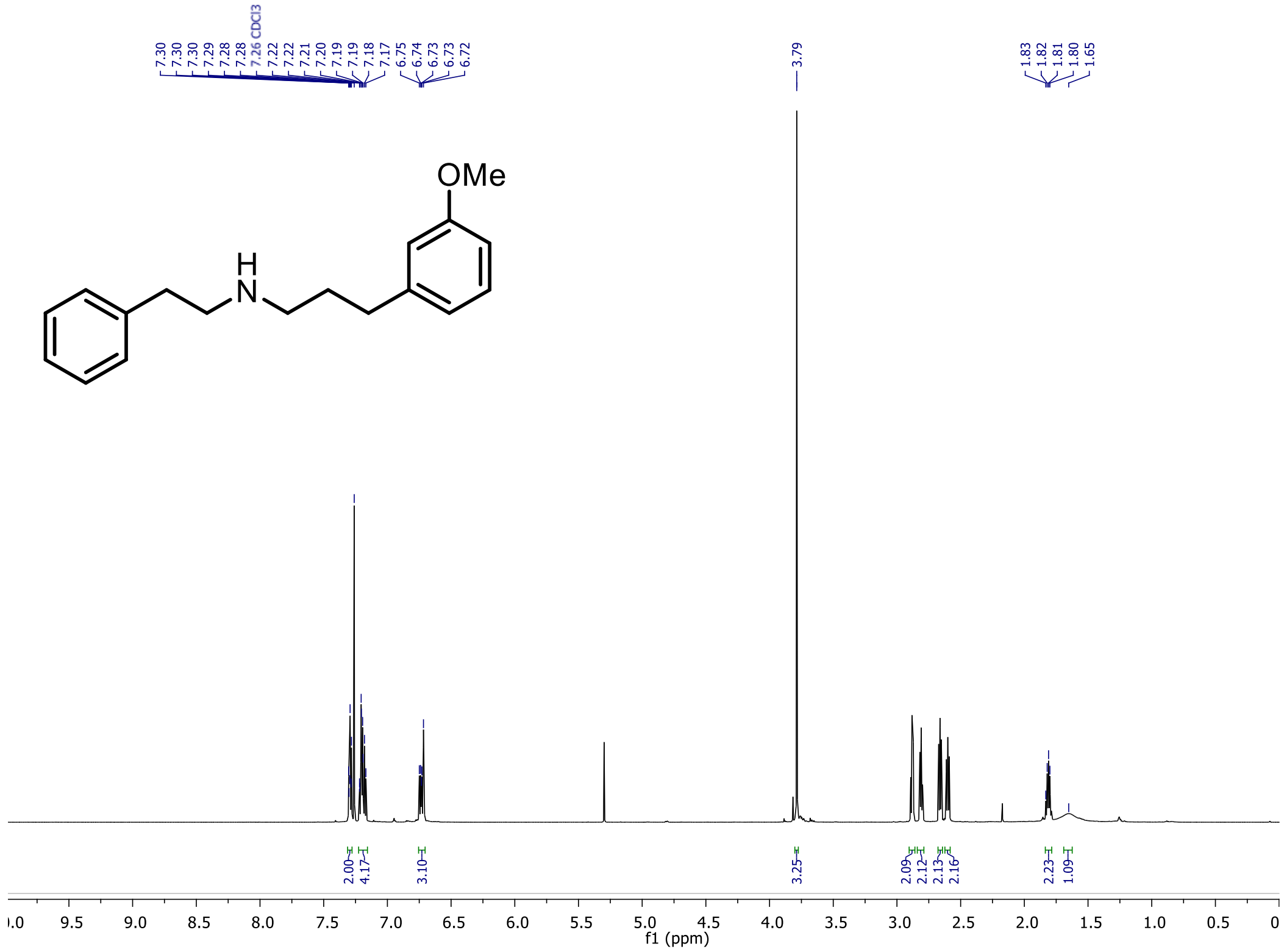

^{13}C NMR (176 MHz, CDCl$_3$)

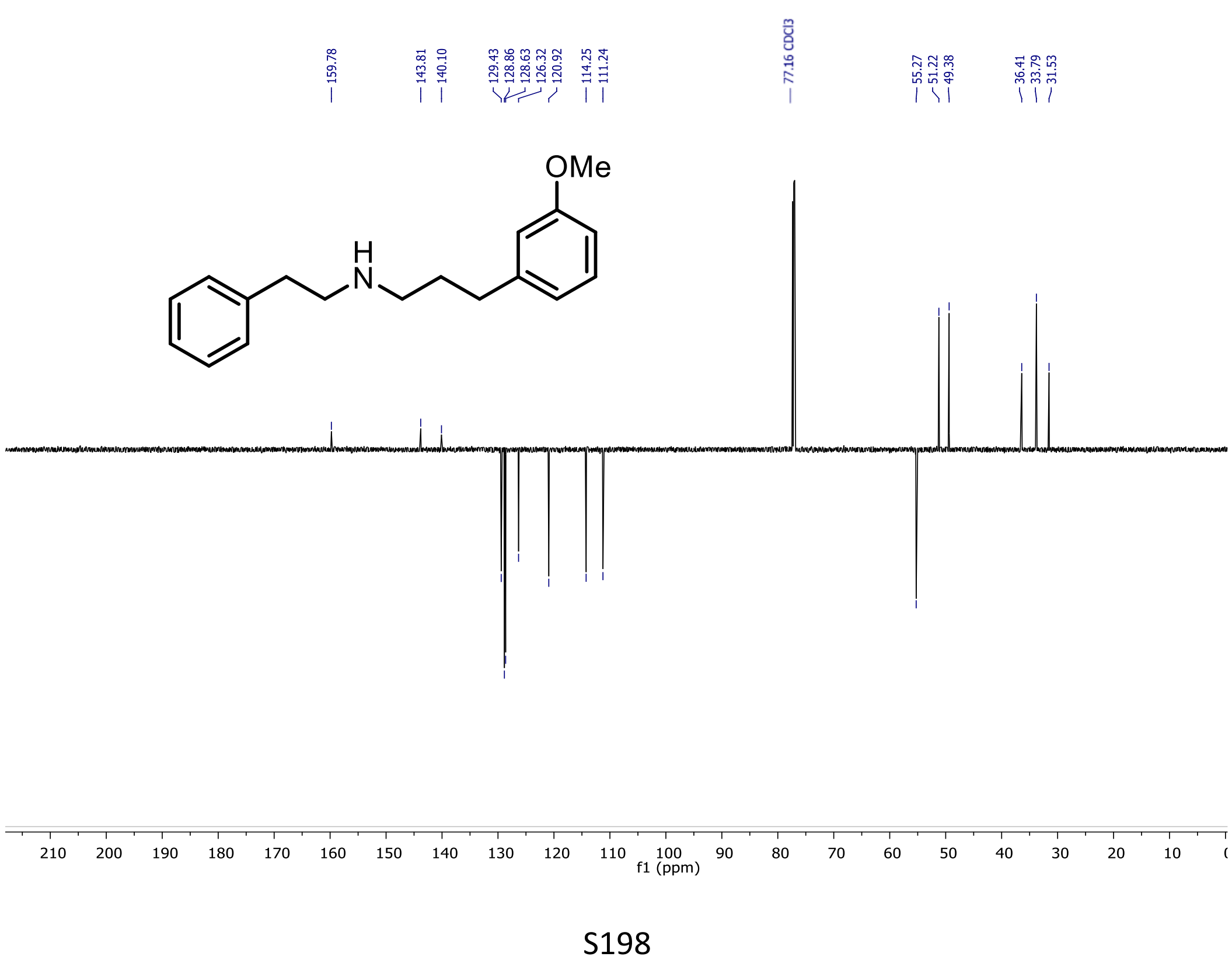

3-(3-(Phenethylamino)propyl)benzaldehyde (1.42)

^1H NMR (600 MHz, $CDCl_3$)

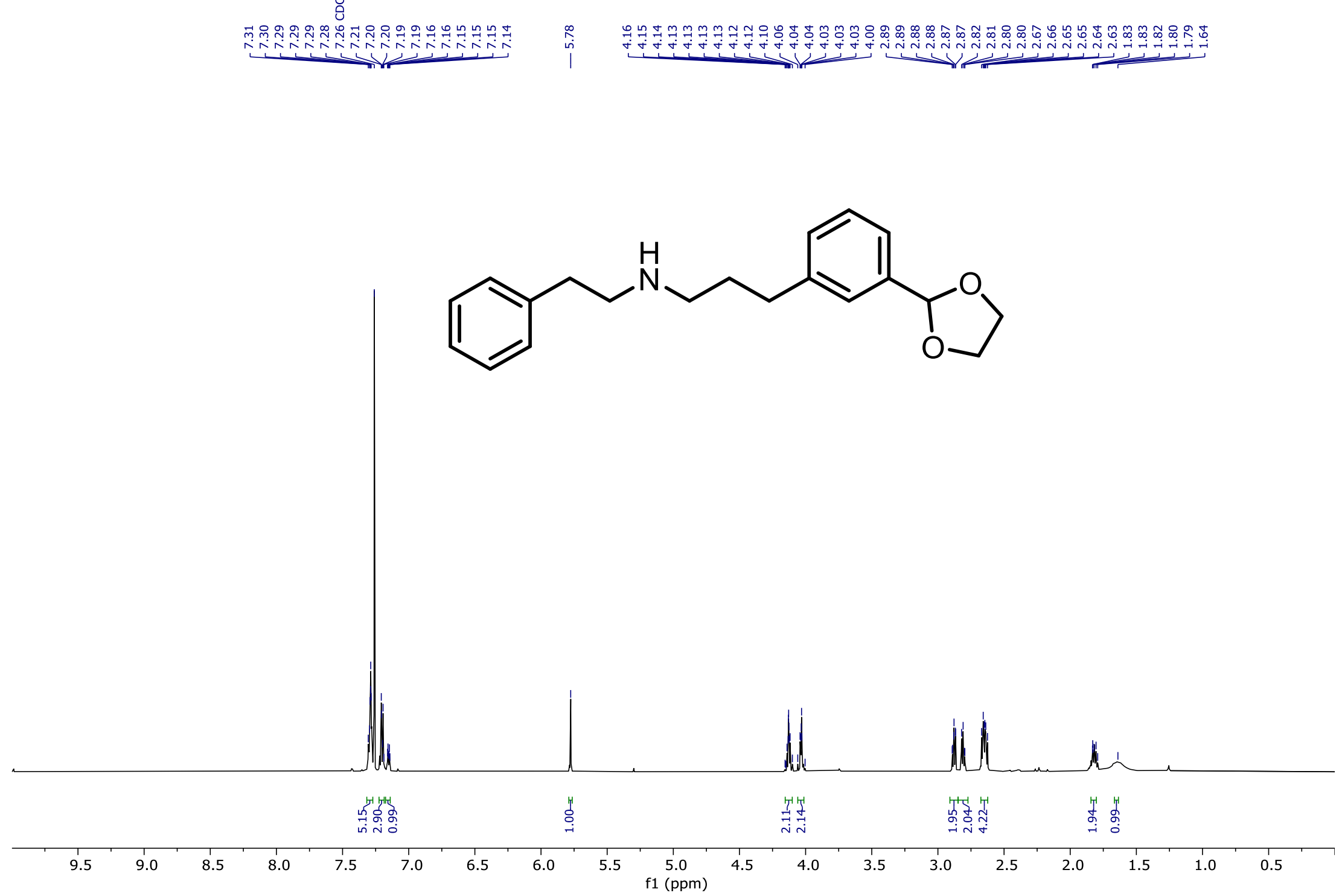

^{13}C NMR (151 MHz, $CDCl_3$)

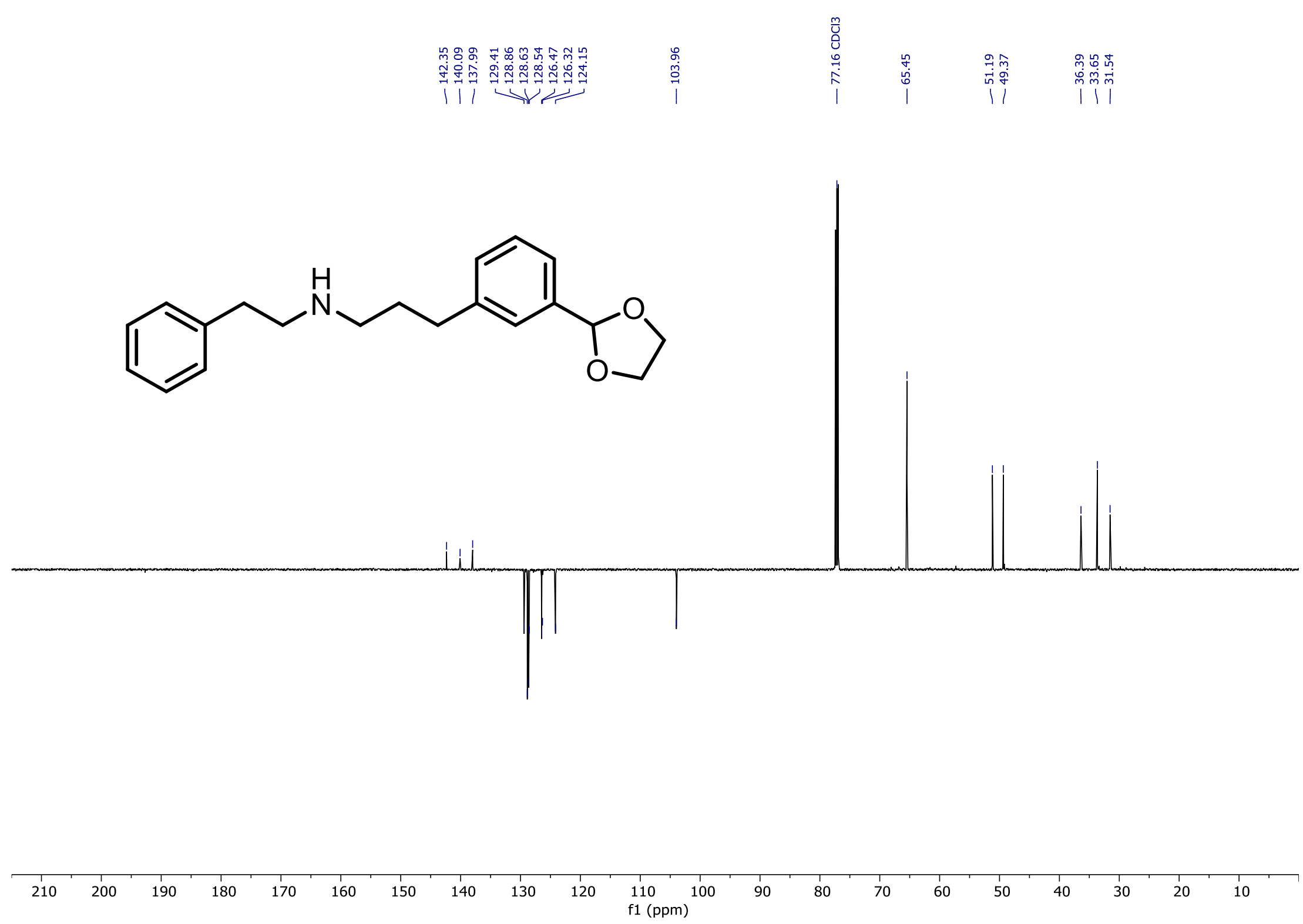

7-((*tert*-Butyldimethylsilyl)oxy)-*N*-phenethylheptan-1-amine (1.43)

^{1}H NMR (400 MHz, $CDCl_3$)

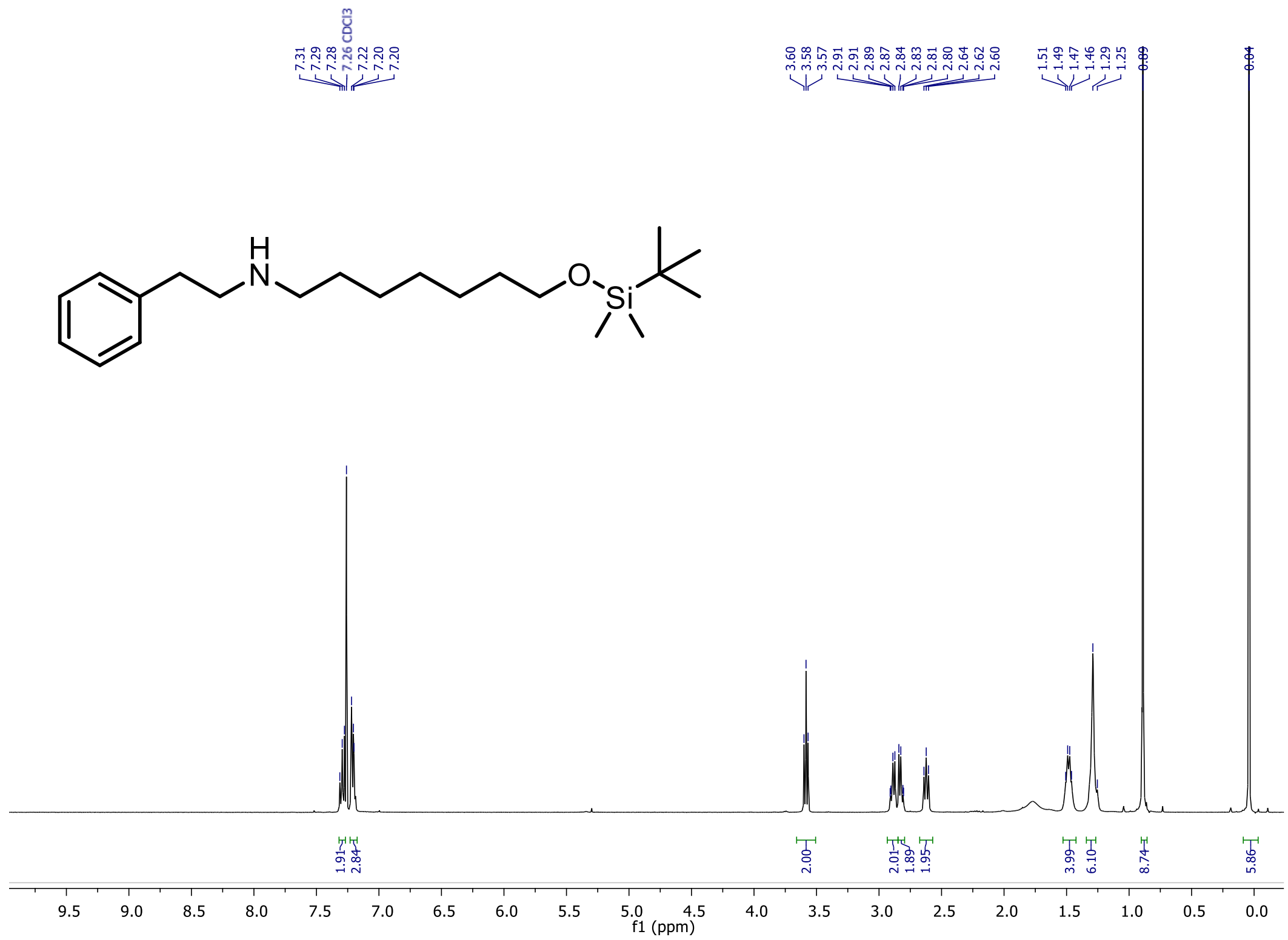

^{13}C NMR (101 MHz, $CDCl_3$)

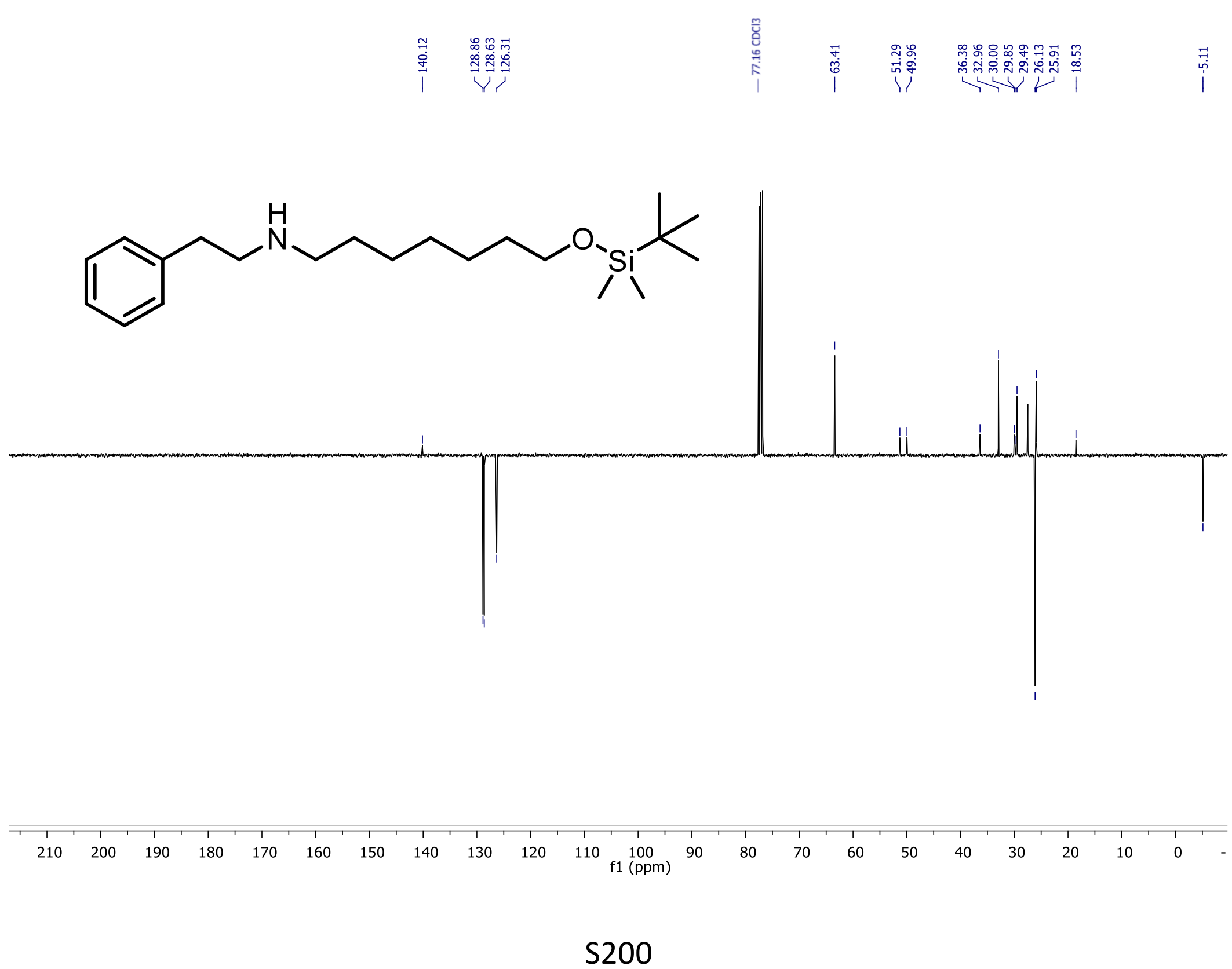

_N_-Methyl-_N_-(12-(phenethylamino)dodecyl)-2,4,6-tris(trifluoromethyl)benzenesulfonamide (1.44)

^{1}H NMR (400 MHz, $CDCl_3$)

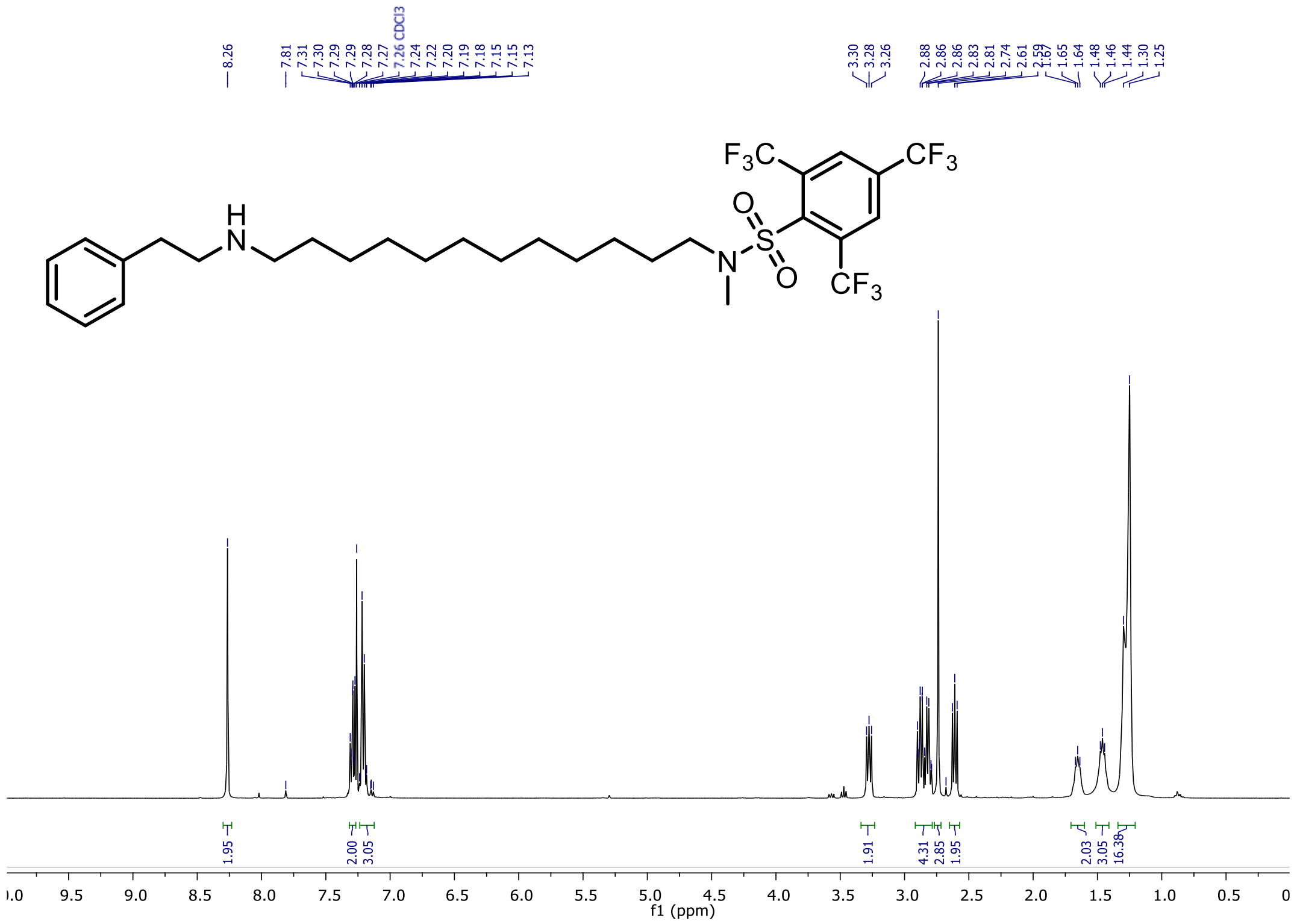

^{13}C NMR (101 MHz, $CDCl_3$)

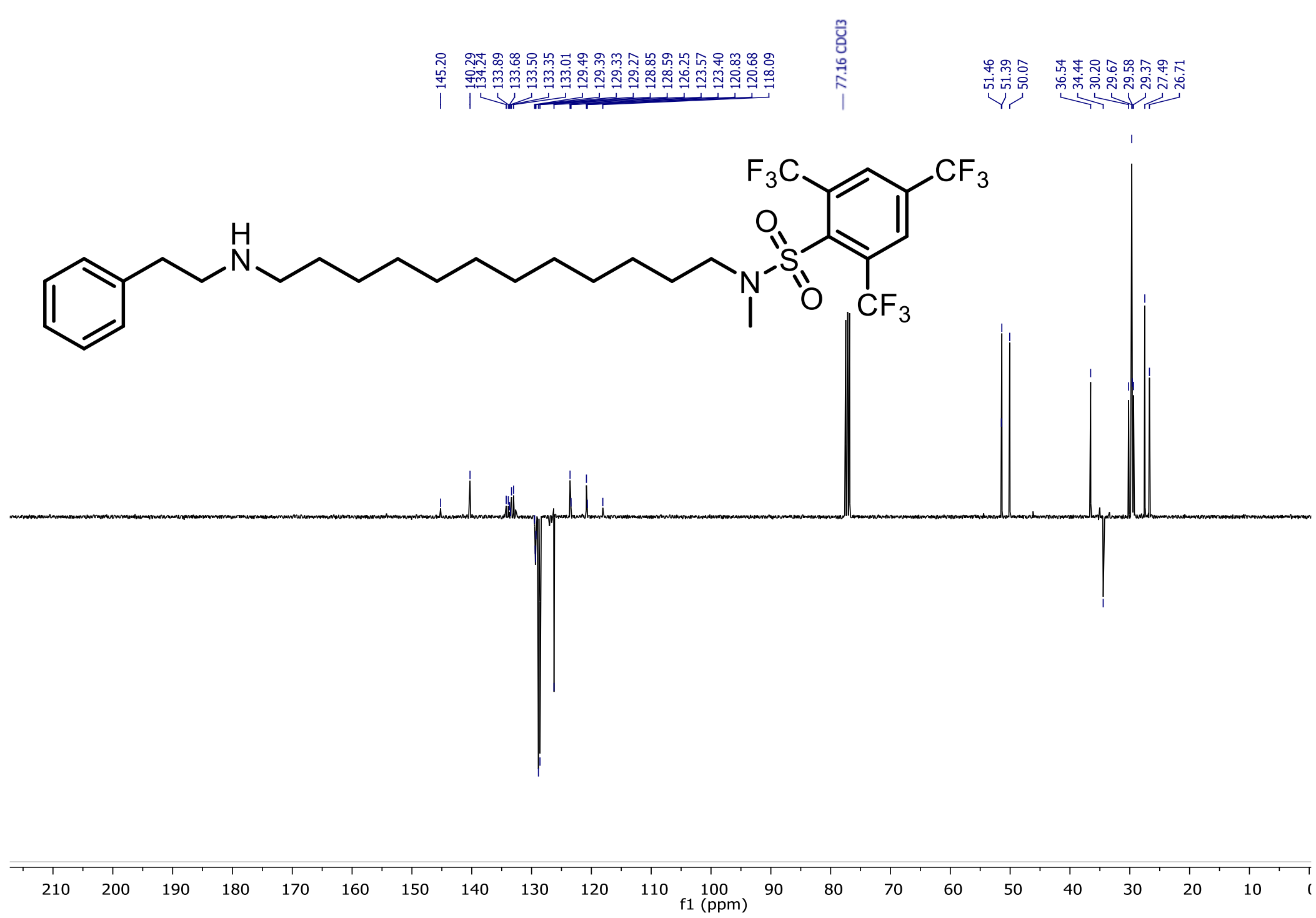

^{19}F NMR (376 MHz, $CDCl_3$)

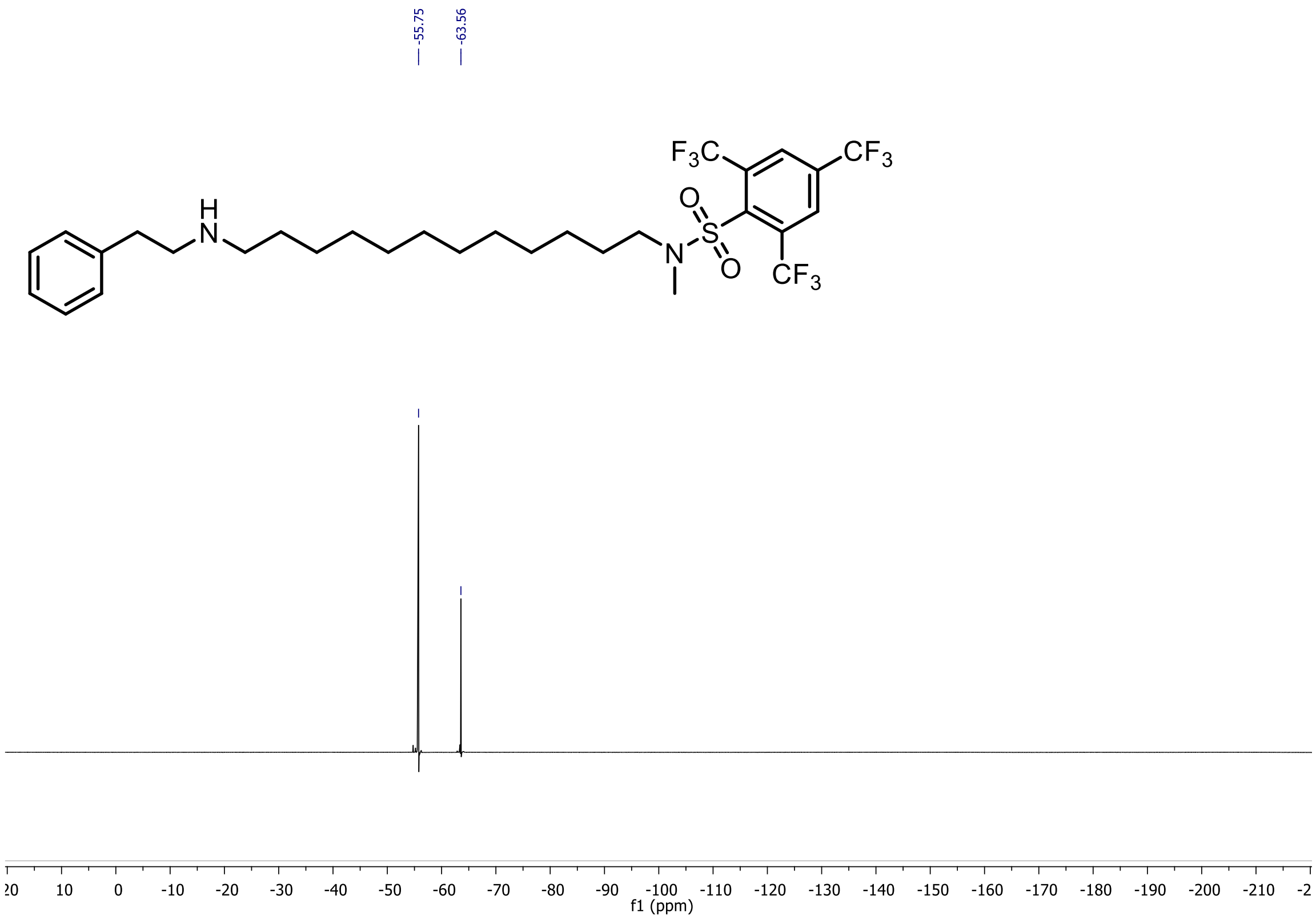

(*E*)-3-Cyclohexyl-*N*-phenethylprop-2-en-1-amine (1.45)
^{1}H NMR (600 MHz, $CDCl_3$)

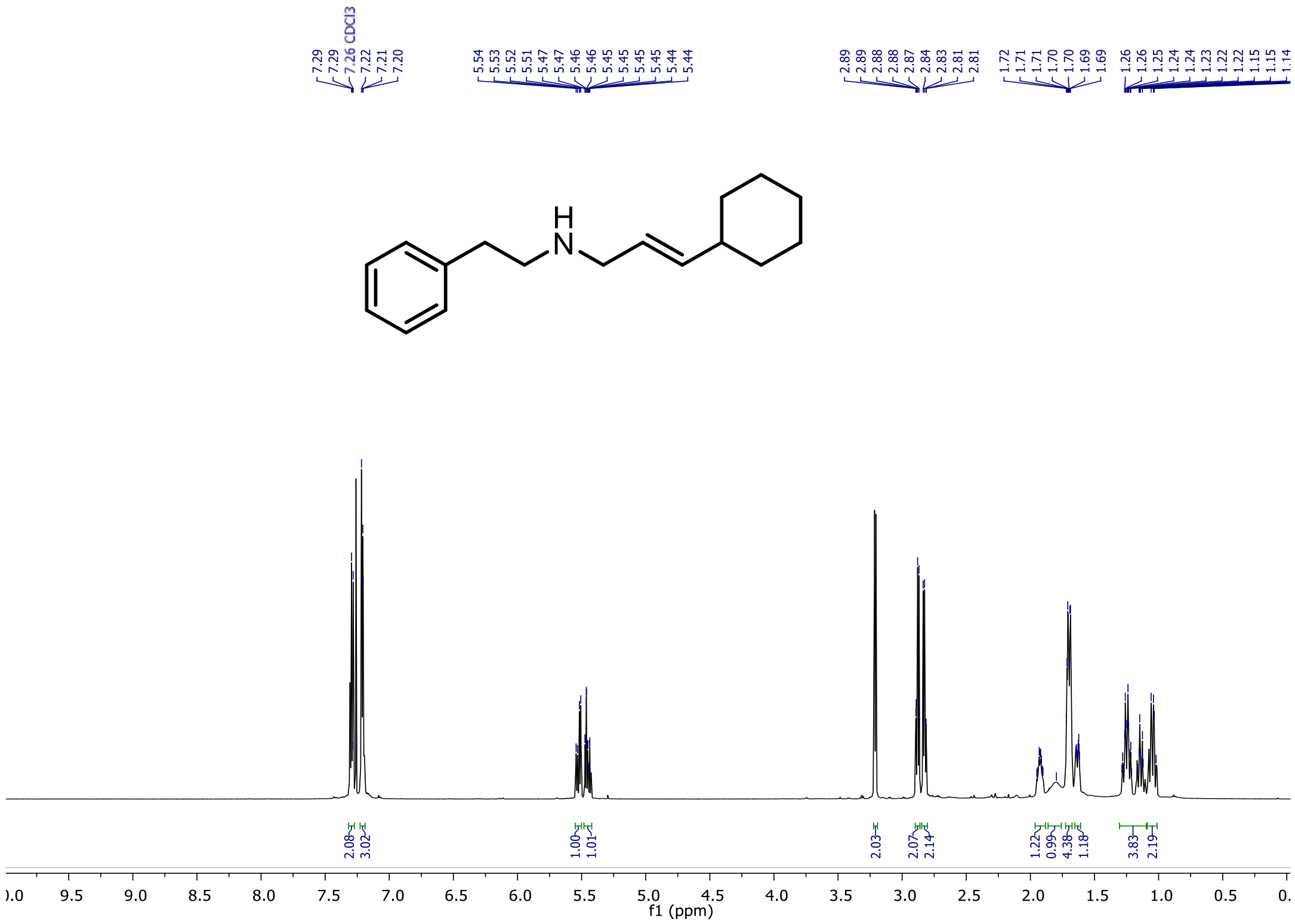

^{13}C NMR (151 MHz, $CDCl_3$)

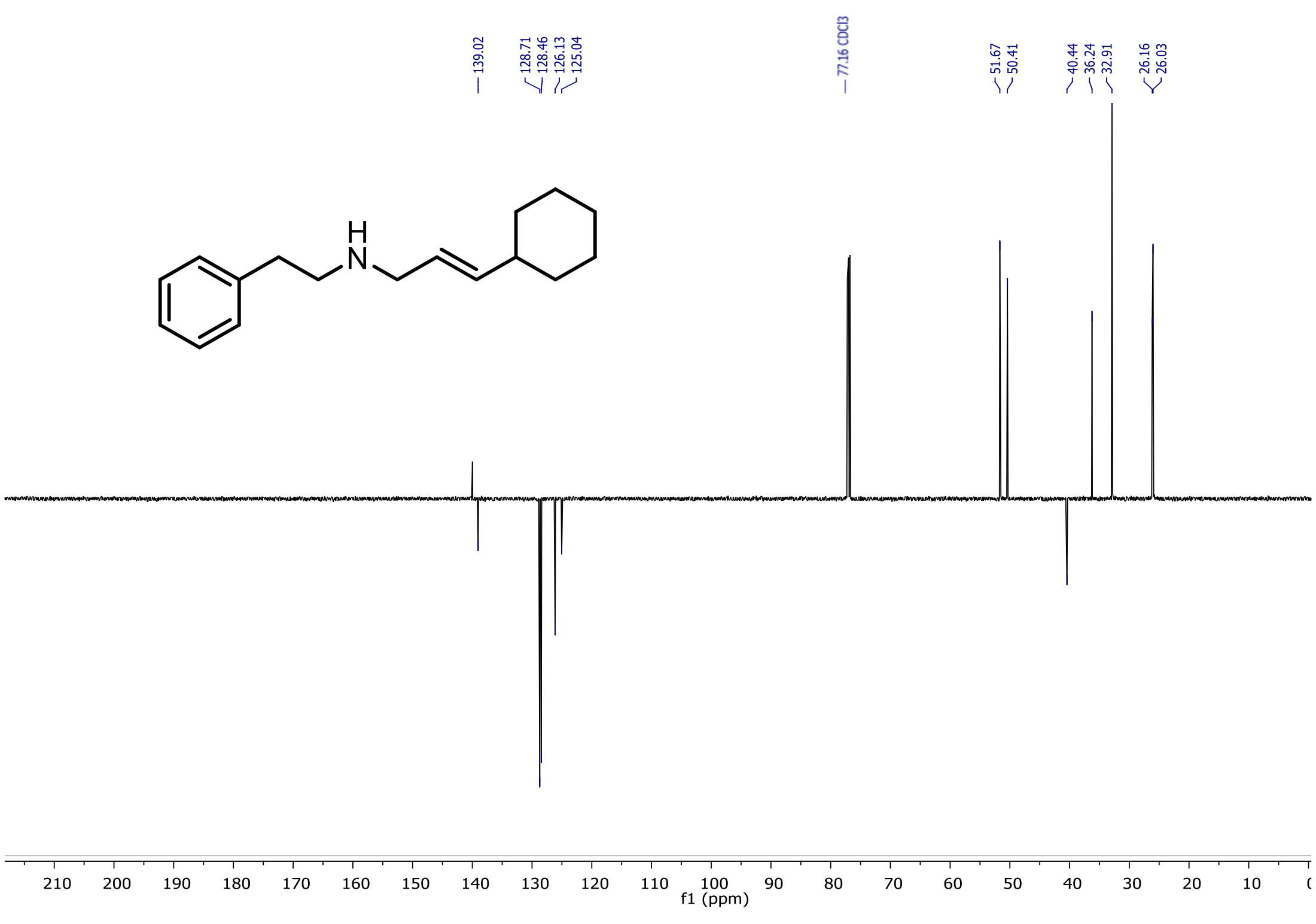

(*E*)-*N*-(2-Phenylpropyl)-3-(*p*-tolyl)prop-2-en-1-amine (1.46)

^{1}H NMR (600 MHz, $CDCl_3$)

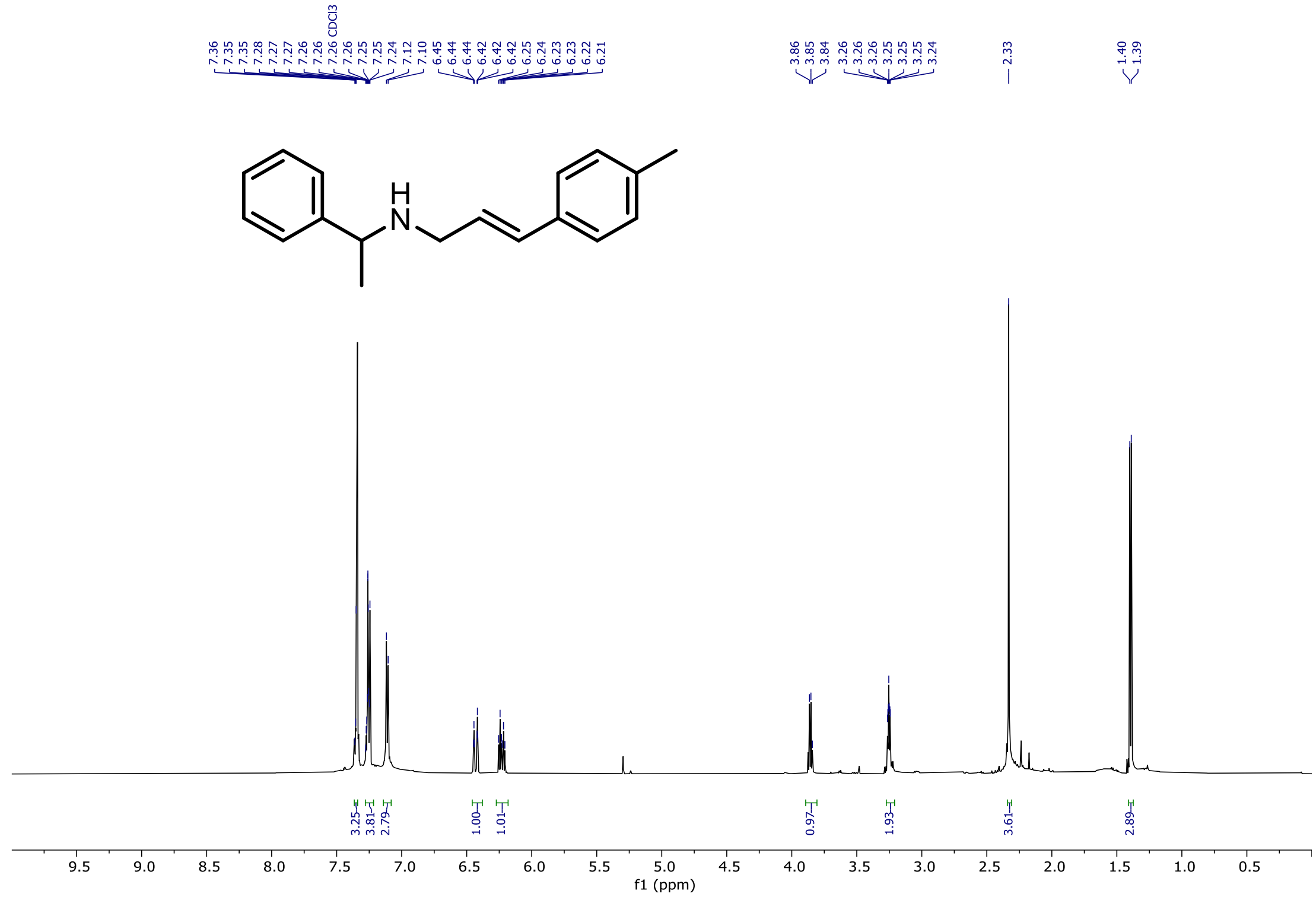

^{13}C NMR (151 MHz, $CDCl_3$)

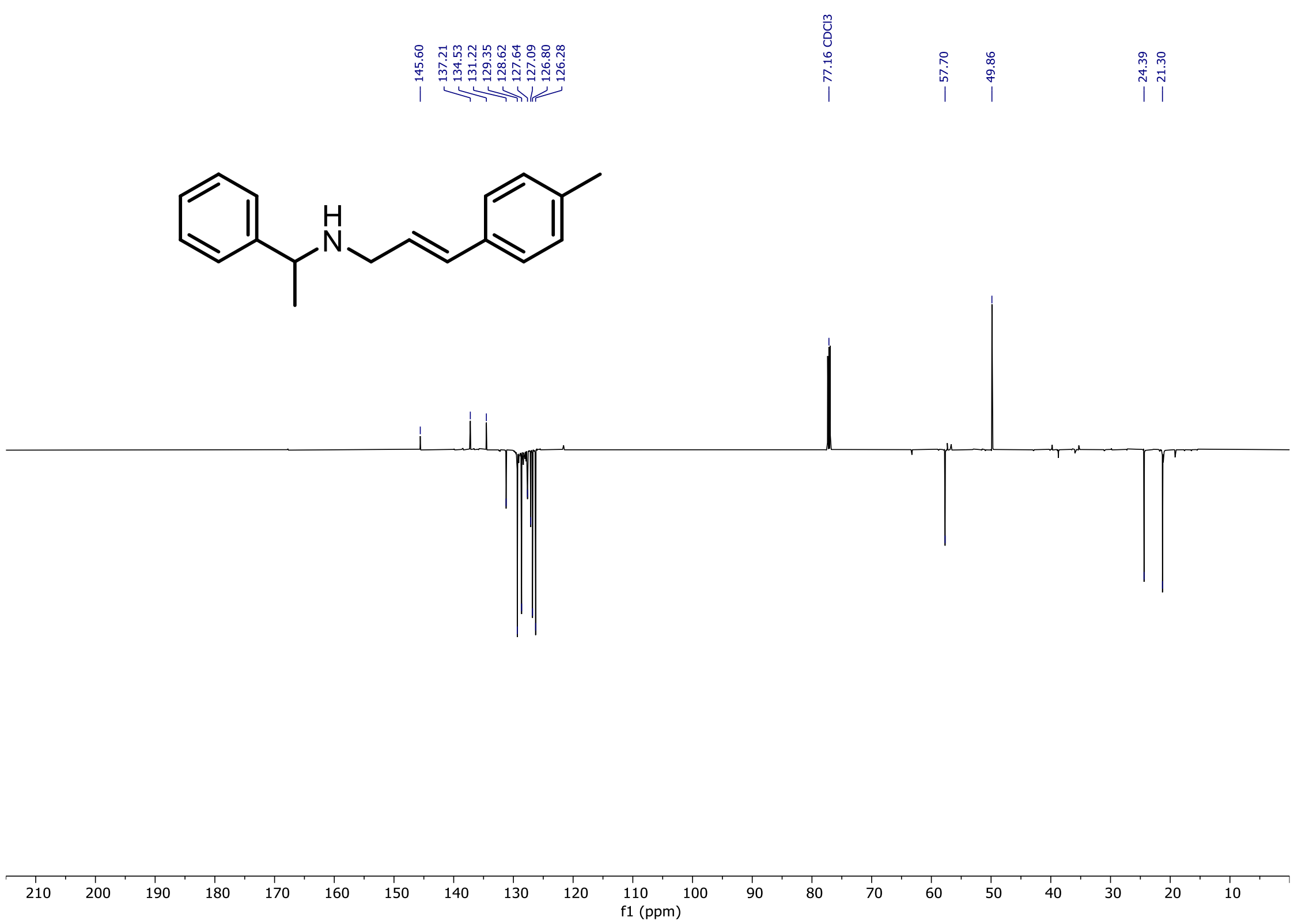

(*E*)-*N*-(1-Phenylethyl)hept-2-en-1-amine (1.47)

^{1}H NMR (400 MHz, CDCl$_3$)

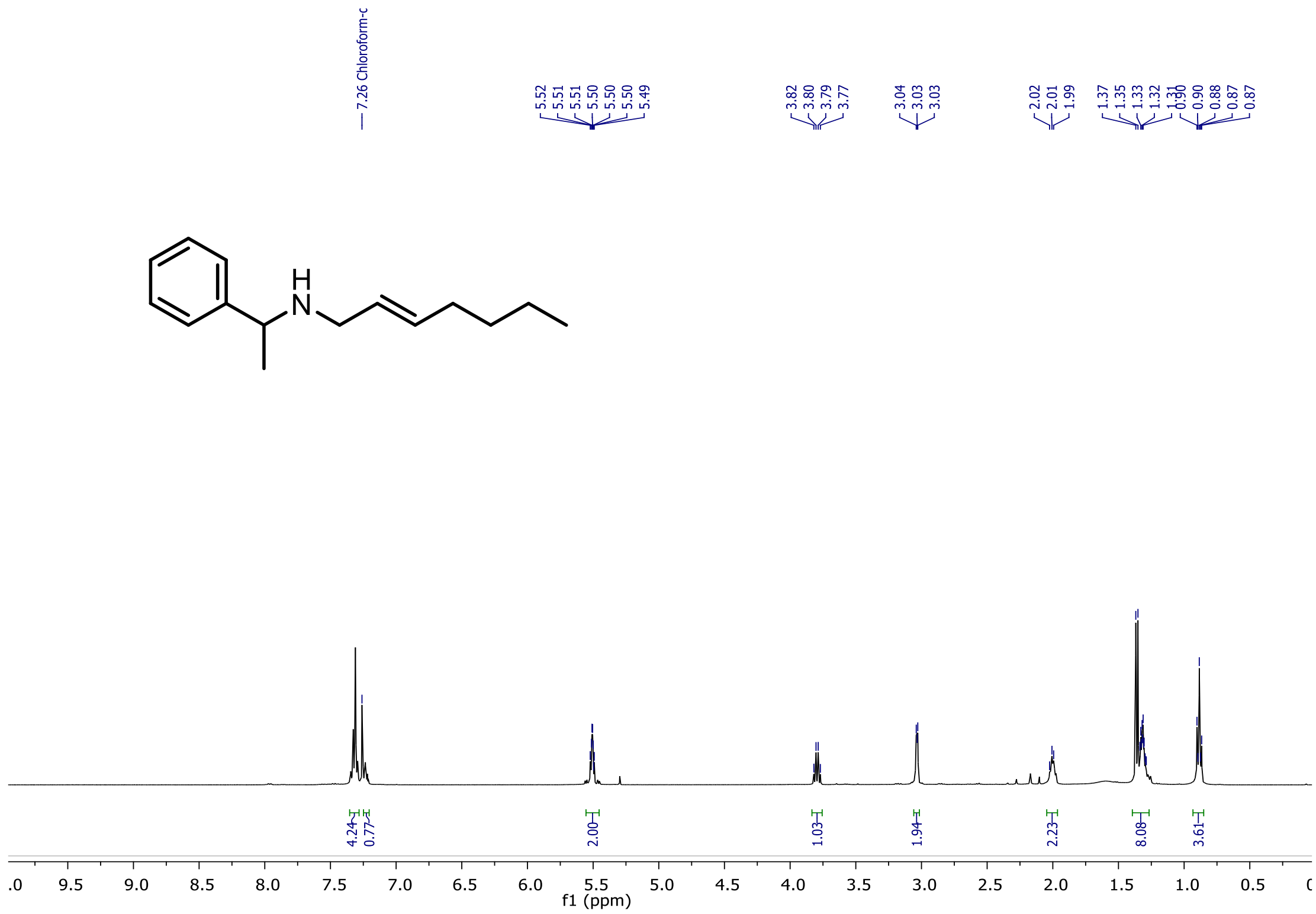

^{13}C NMR (101 MHz, CDCl$_3$)

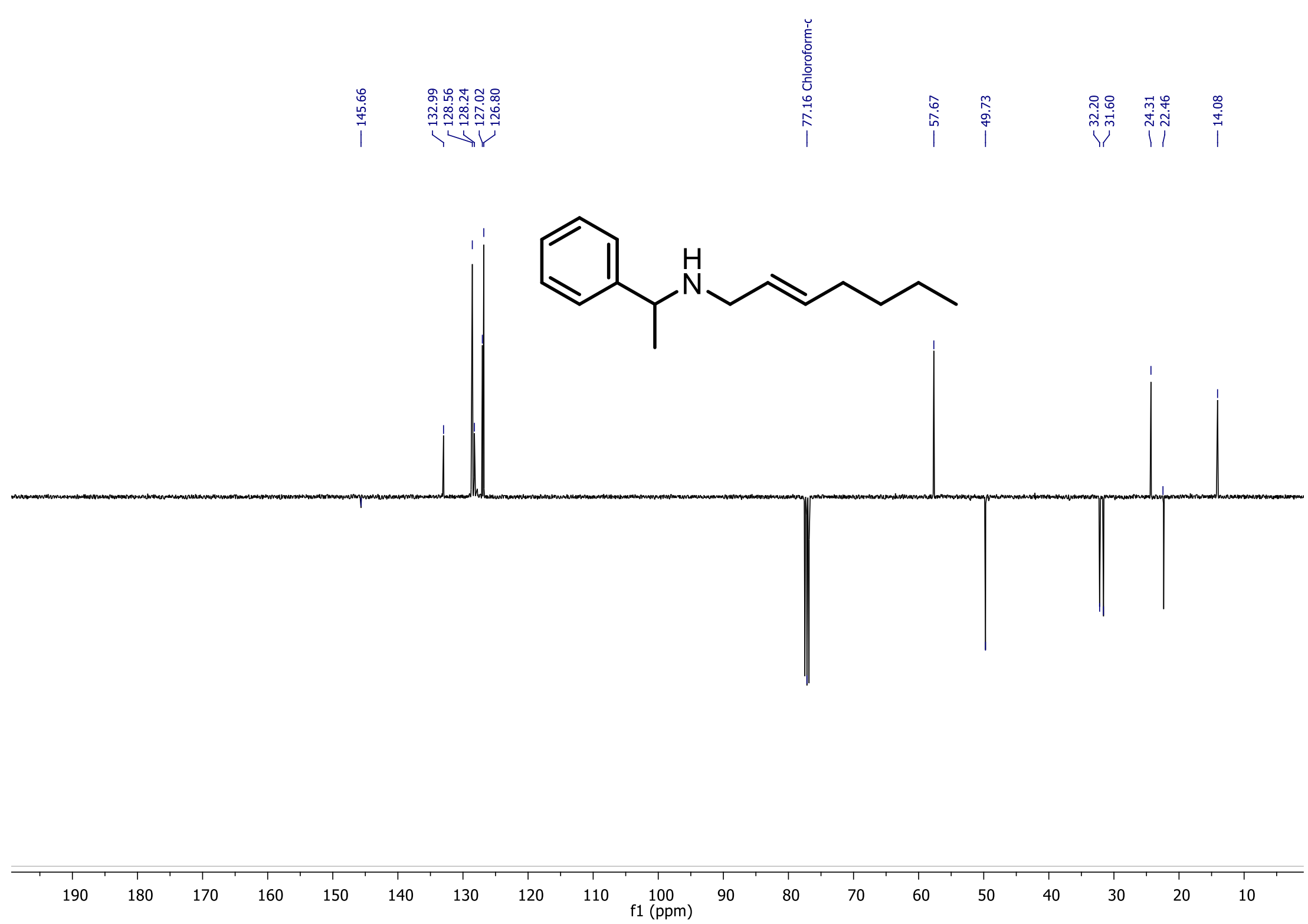

(*E*)-*N*-(1-Phenylethyl)undec-2-en-1-amine (1.48)

^{1}H NMR (400 MHz, CDCl$_3$)

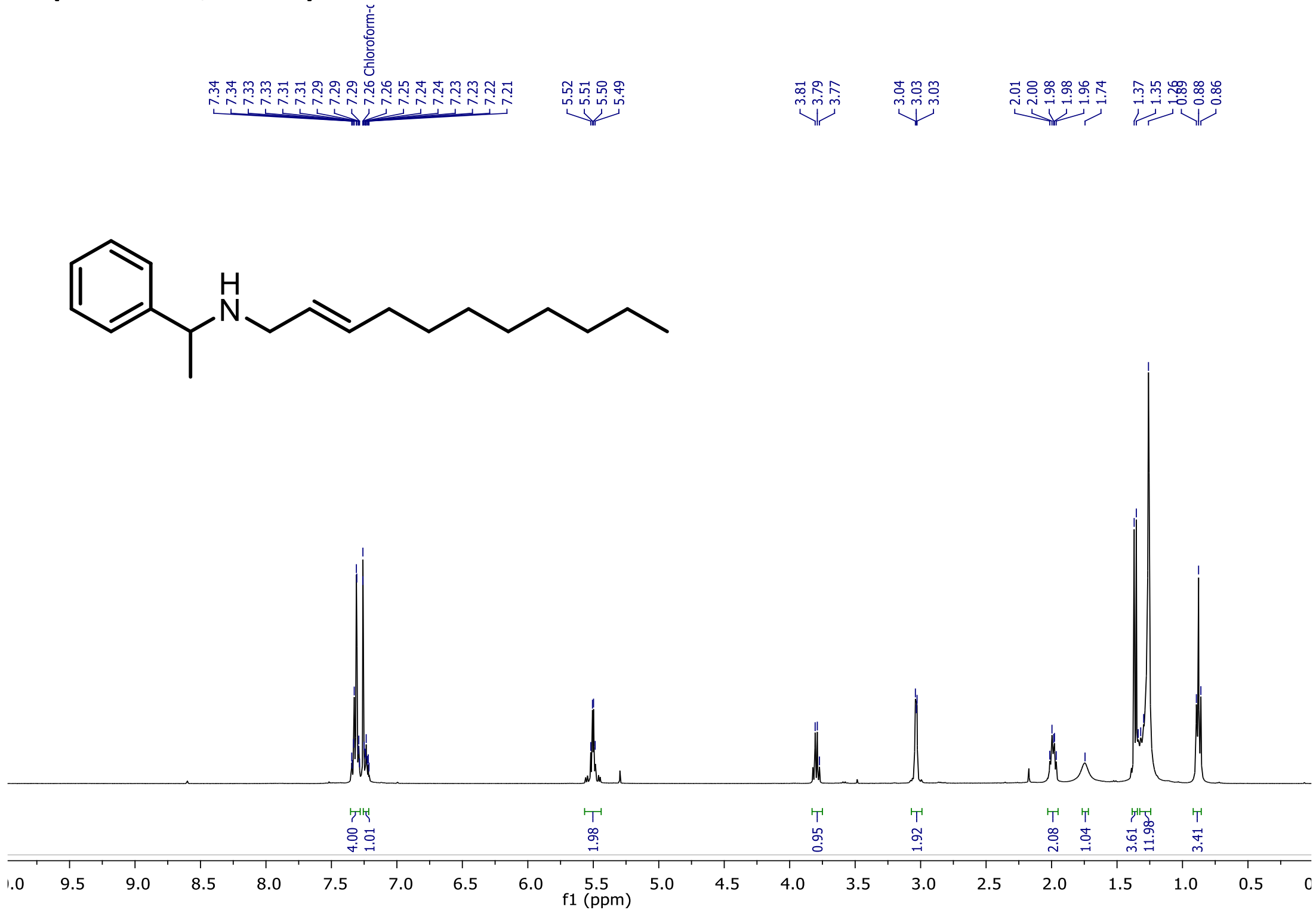

^{13}C NMR (101 MHz, CDCl$_3$)

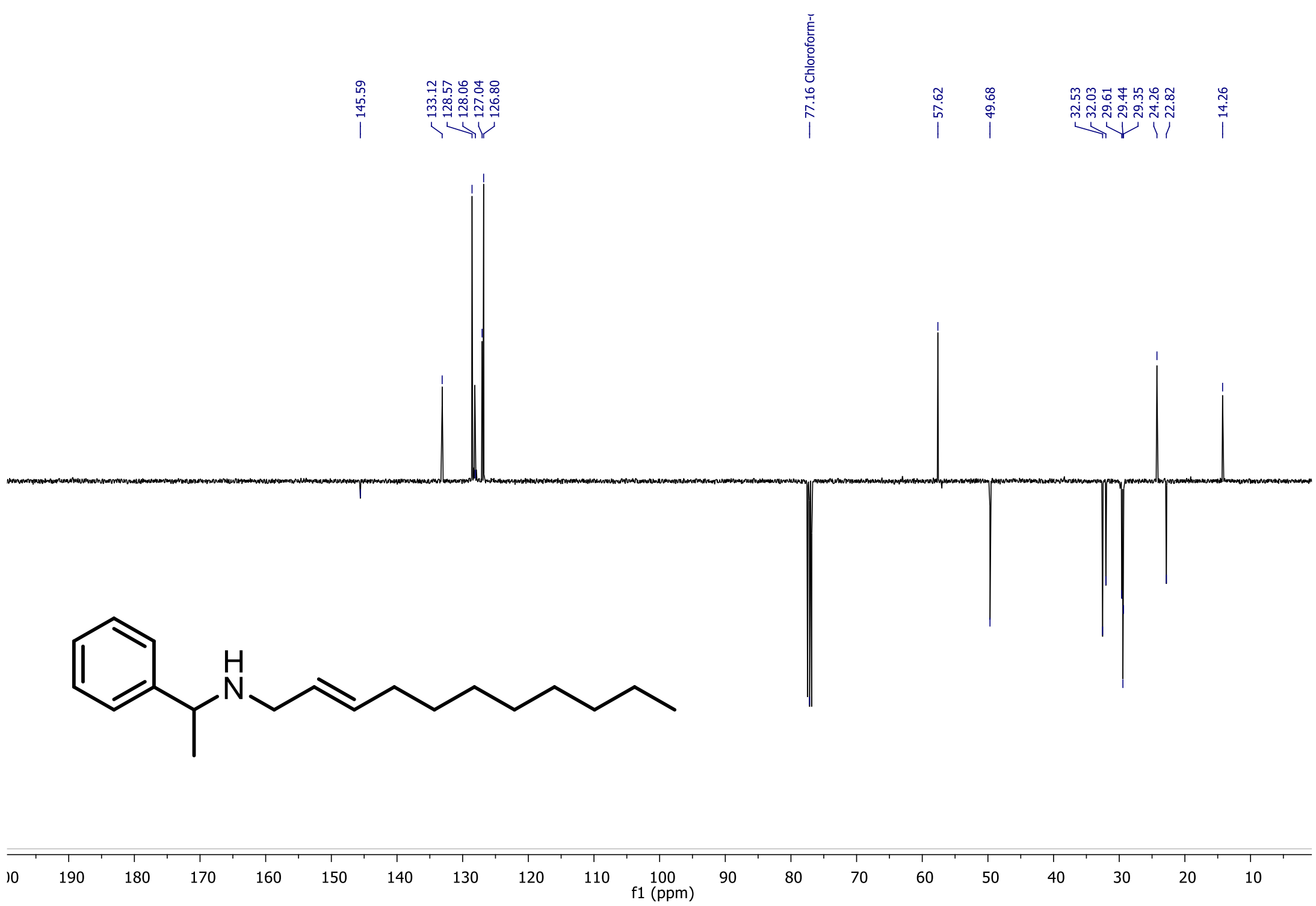

(*E*)-3-Cyclopropyl-N-(1-phenylethyl)prop-2-en-1-amine (1.49)

^{1}H NMR (400 MHz, $CDCl_3$)

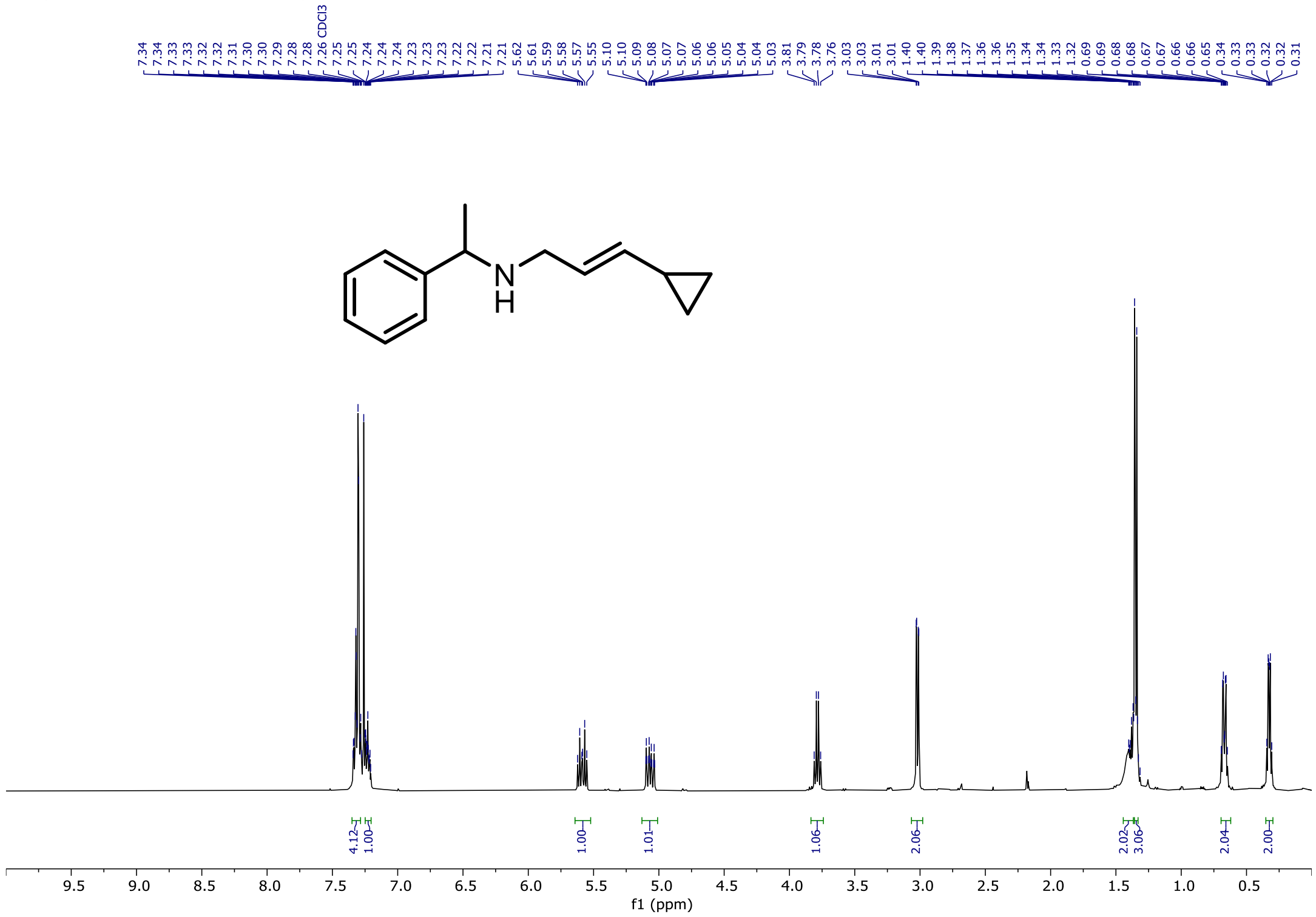

^{13}C NMR (101 MHz, $CDCl_3$)

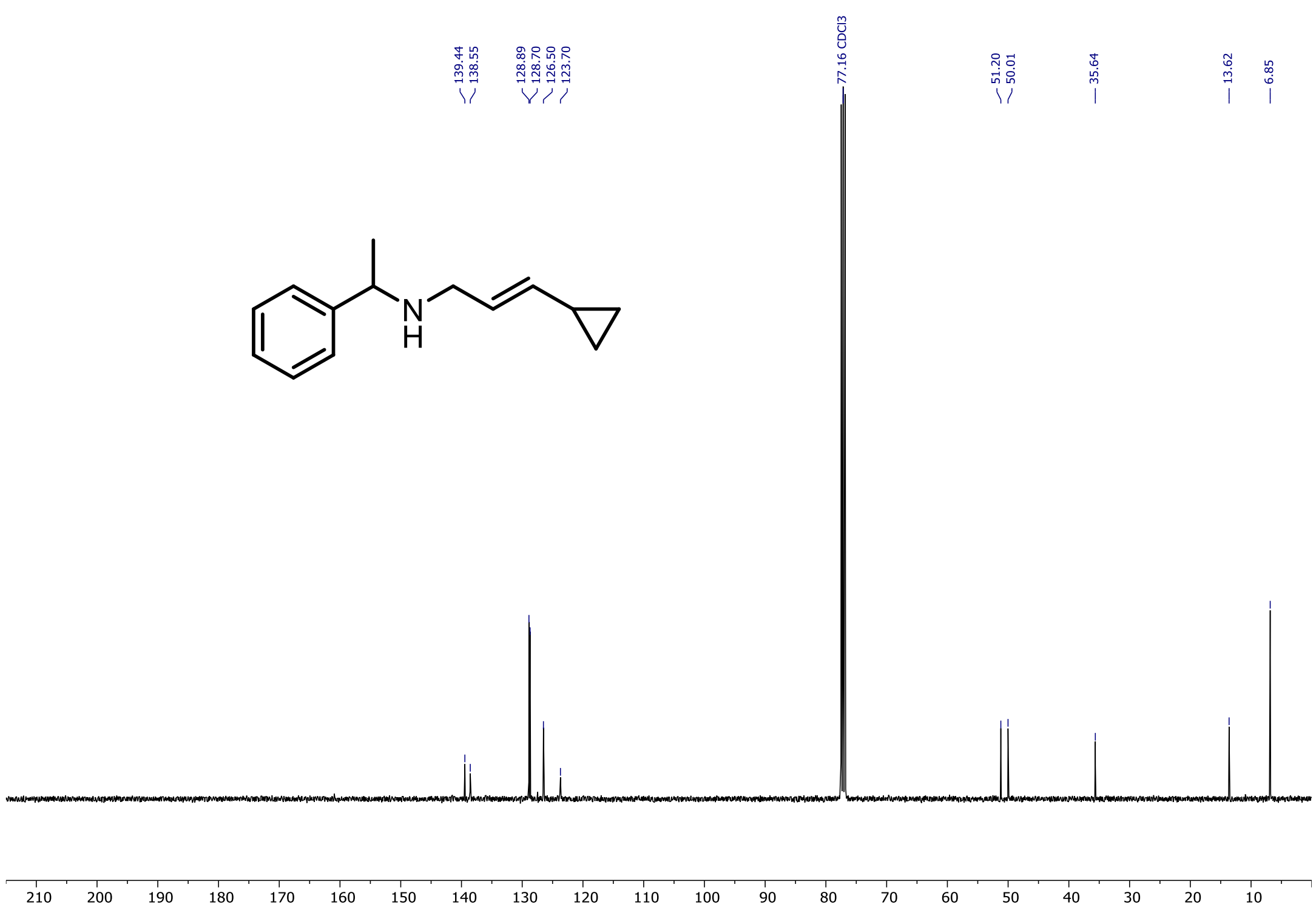

(*E*)-3-Cyclopentyl-*N*-(1-phenylethyl)prop-2-en-1-amine (1.50)
^{1}H NMR (400 MHz, $CDCl_3$)

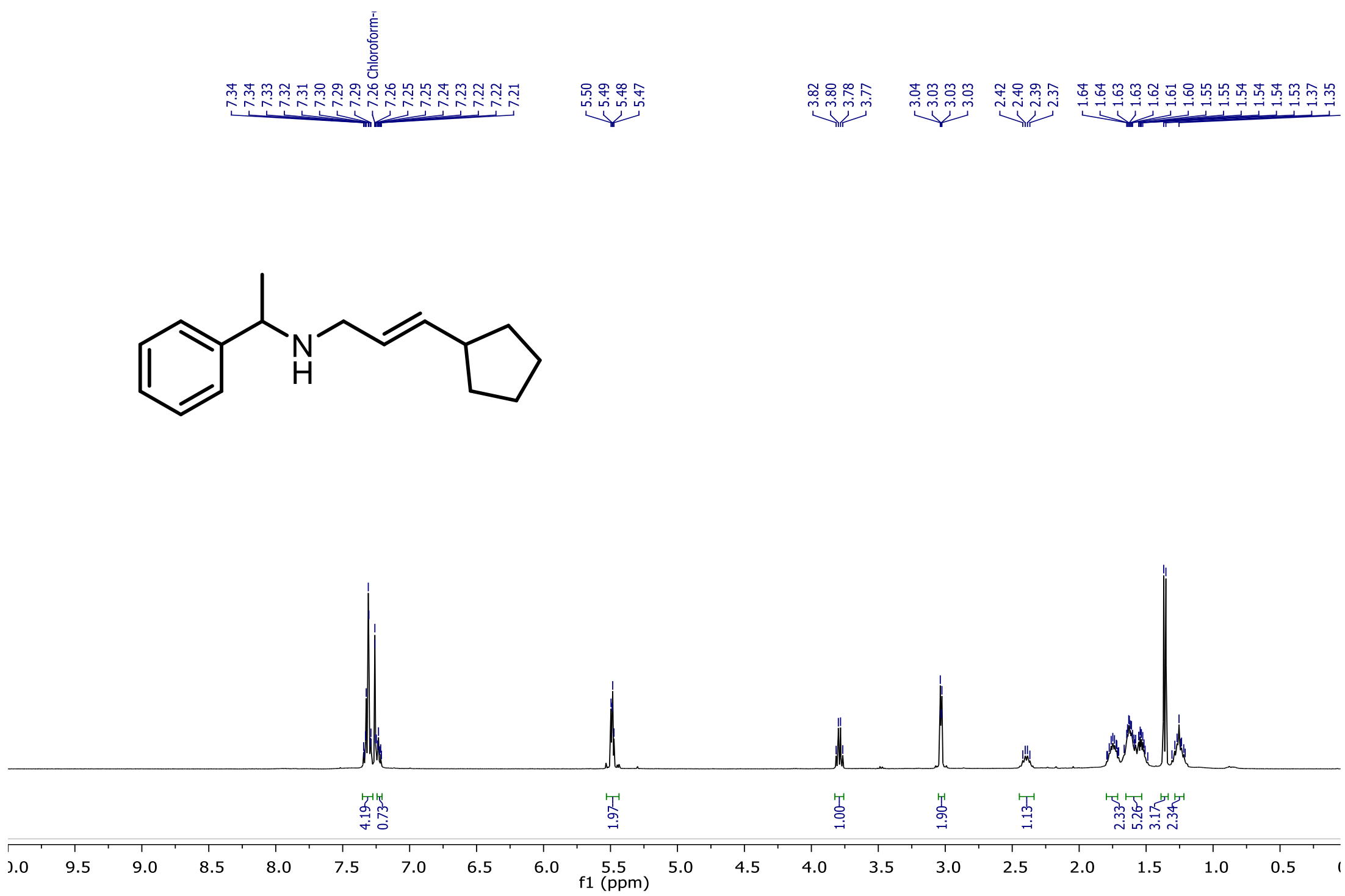

^{13}C NMR (101 MHz, $CDCl_3$)

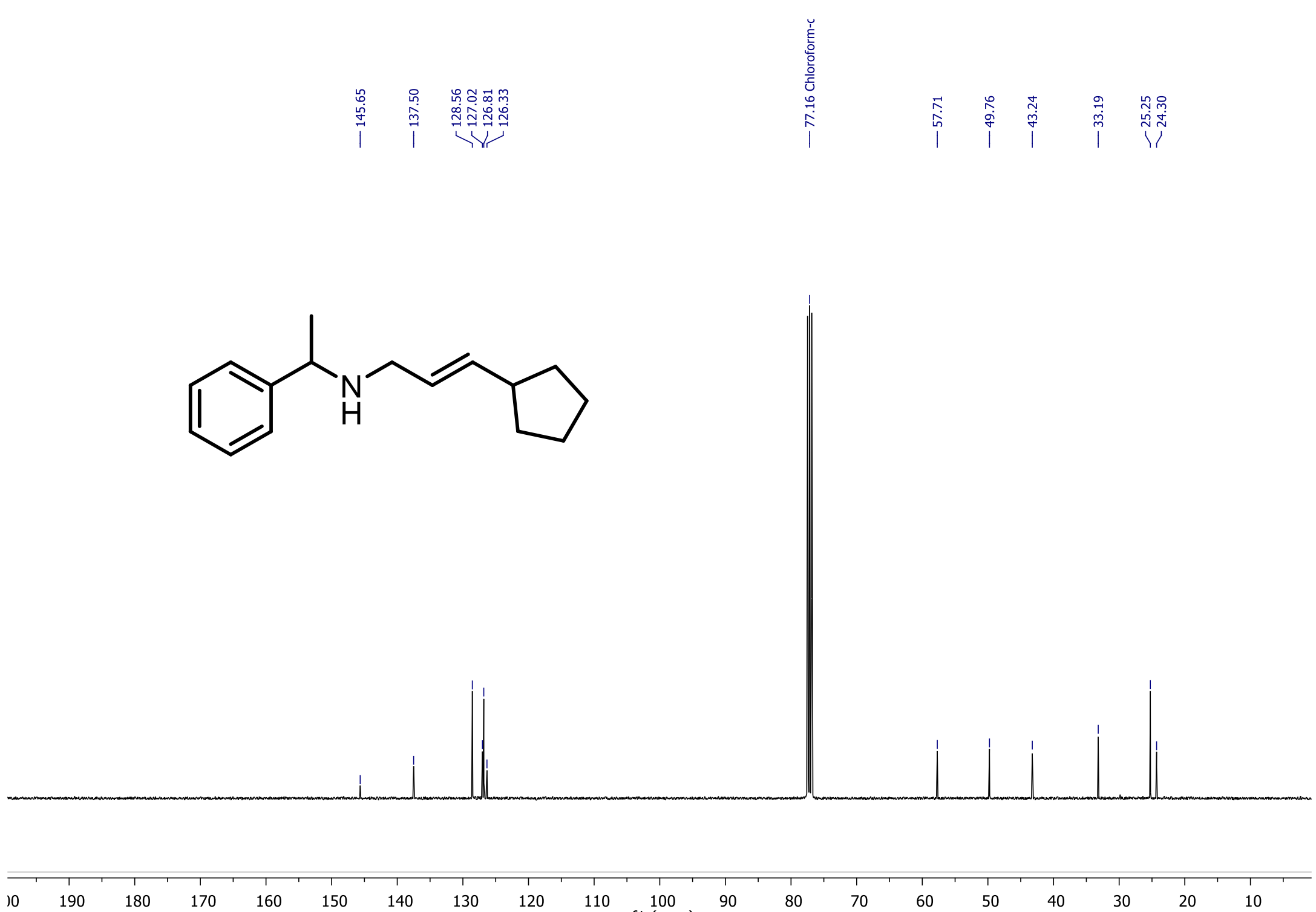

***N*-Phenethyl-5-phenylpentan-1-amine (1.51)**

^{1}H NMR (600 MHz, CDCl$_3$)

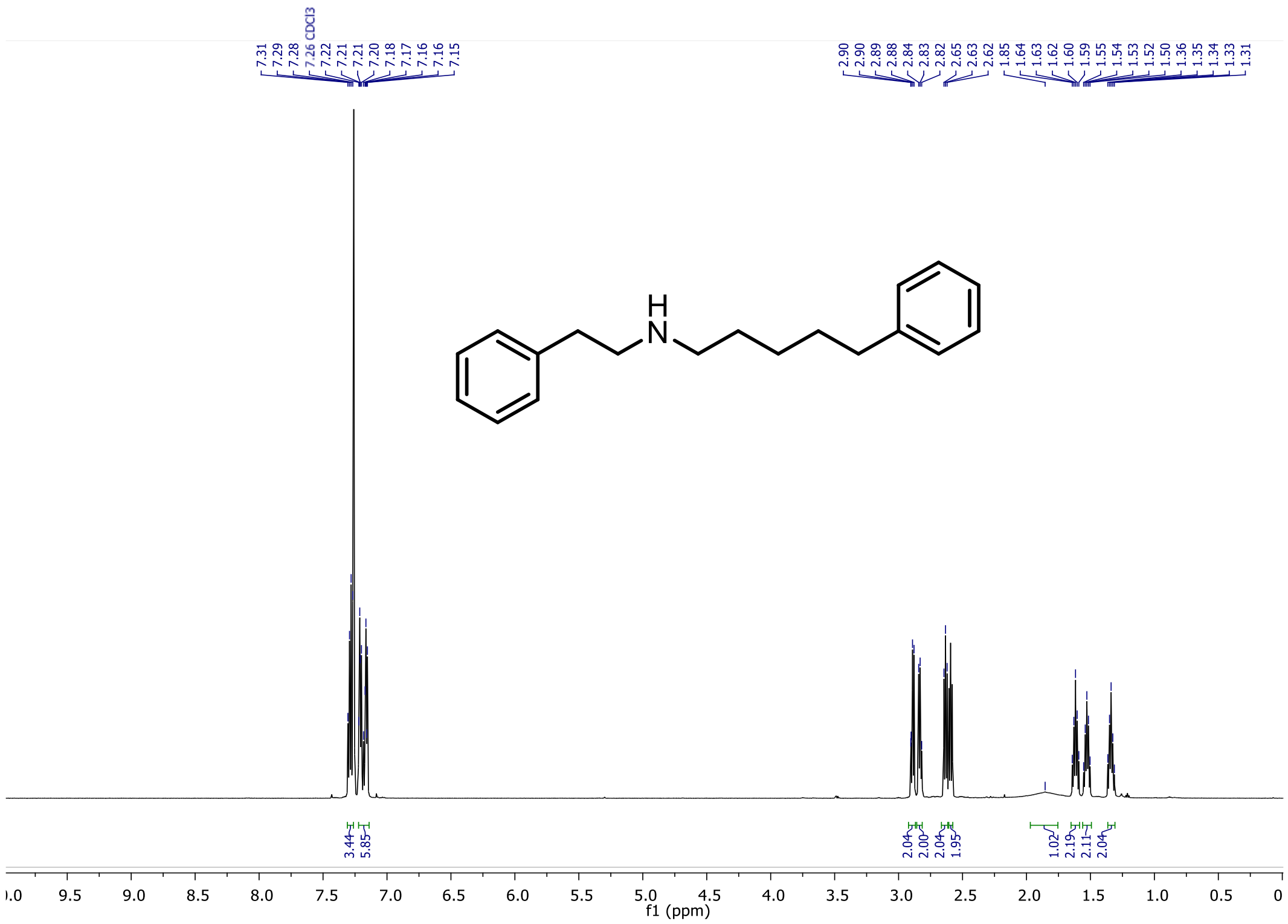

^{13}C NMR (151 MHz, CDCl$_3$)

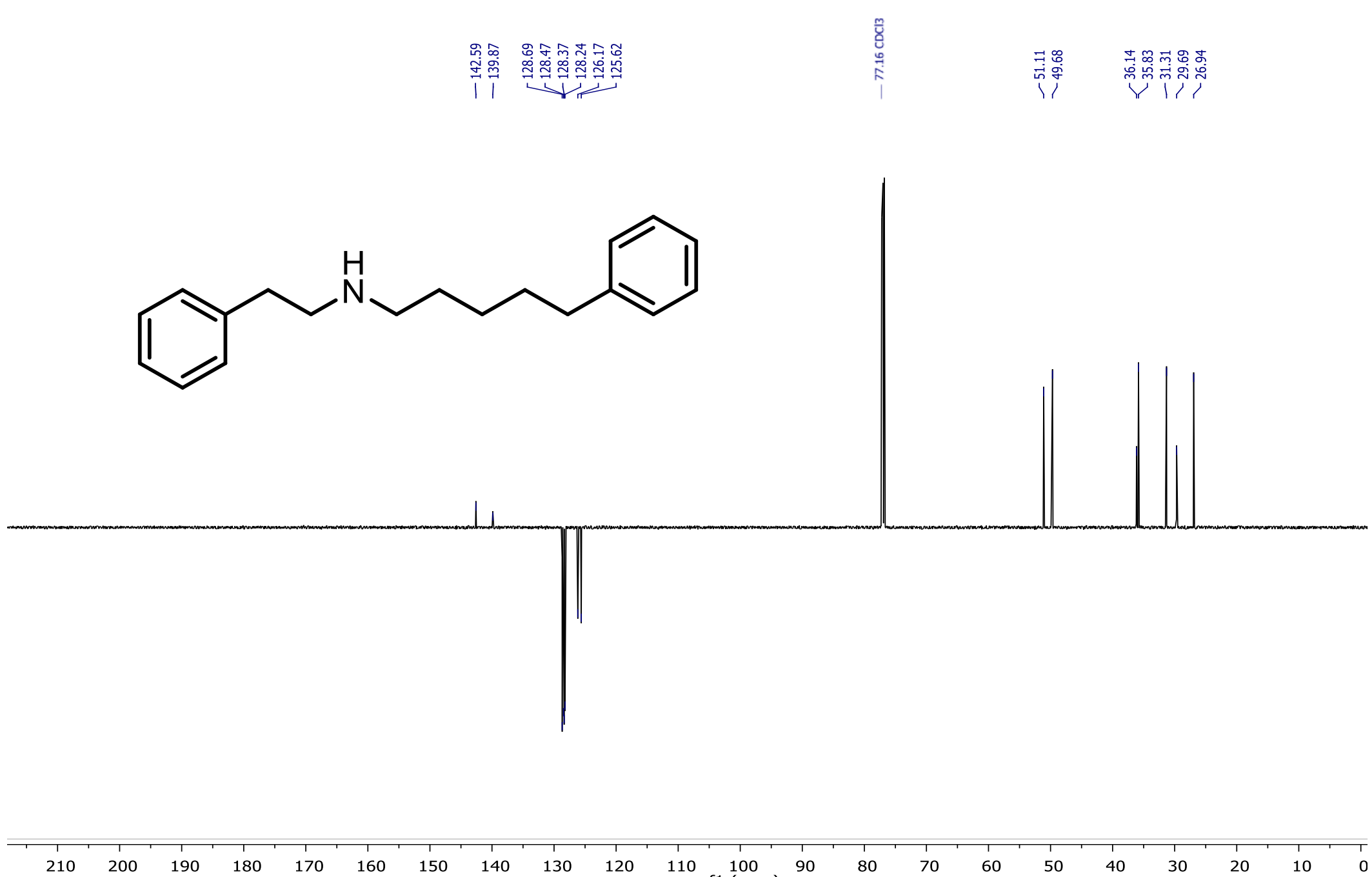

3-((3-Phenylpropyl)amino)propanenitrile (1.52)

^{1}H NMR (600 MHz, CDCl$_3$)

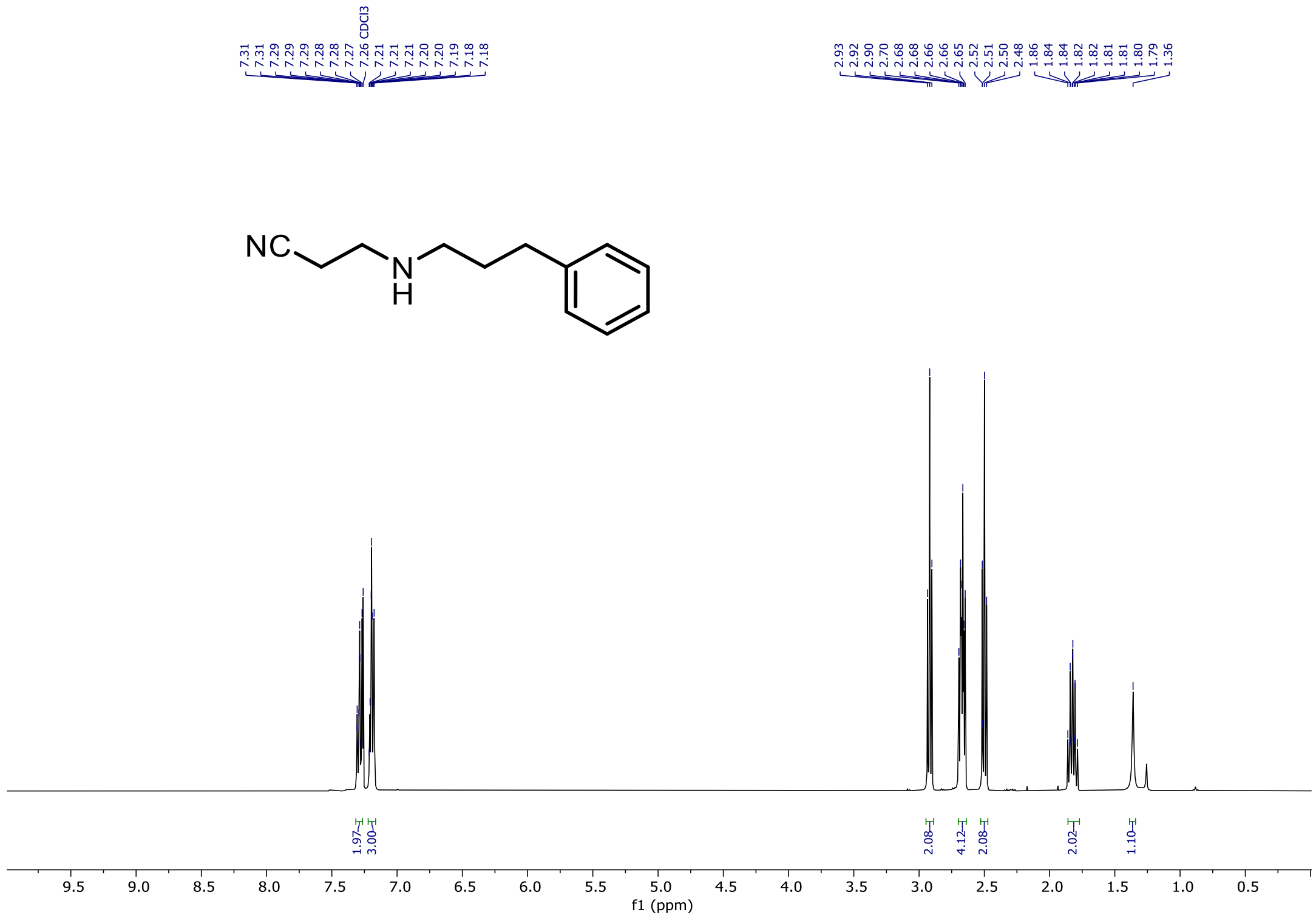

^{13}C NMR (151 MHz, CDCl$_3$)

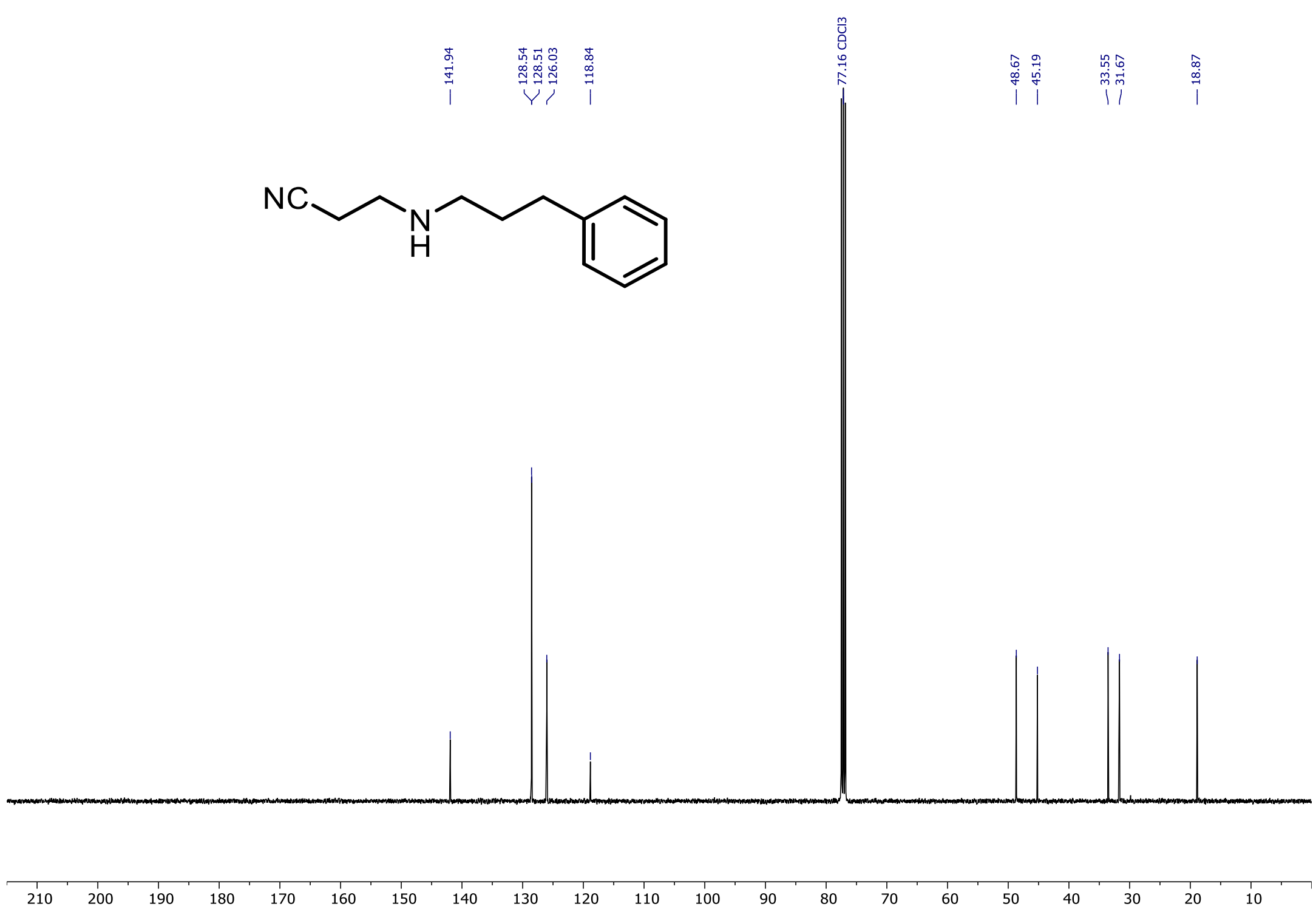

Ethyl (5-phenylpentyl)glycinate (1.53)

^{1}H NMR (400 MHz, $CDCl_3$)

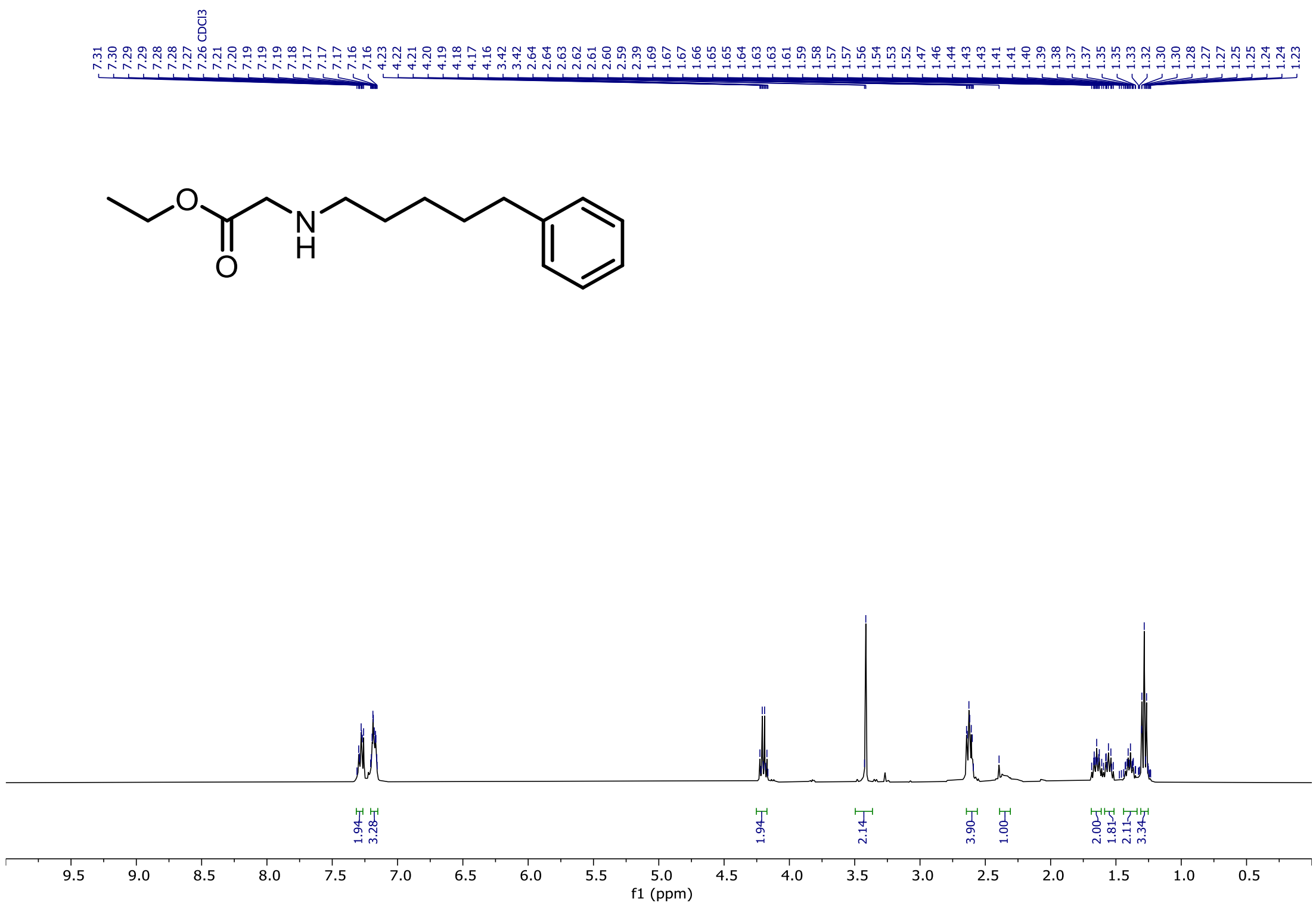

^{13}C NMR (101 MHz, $CDCl_3$)

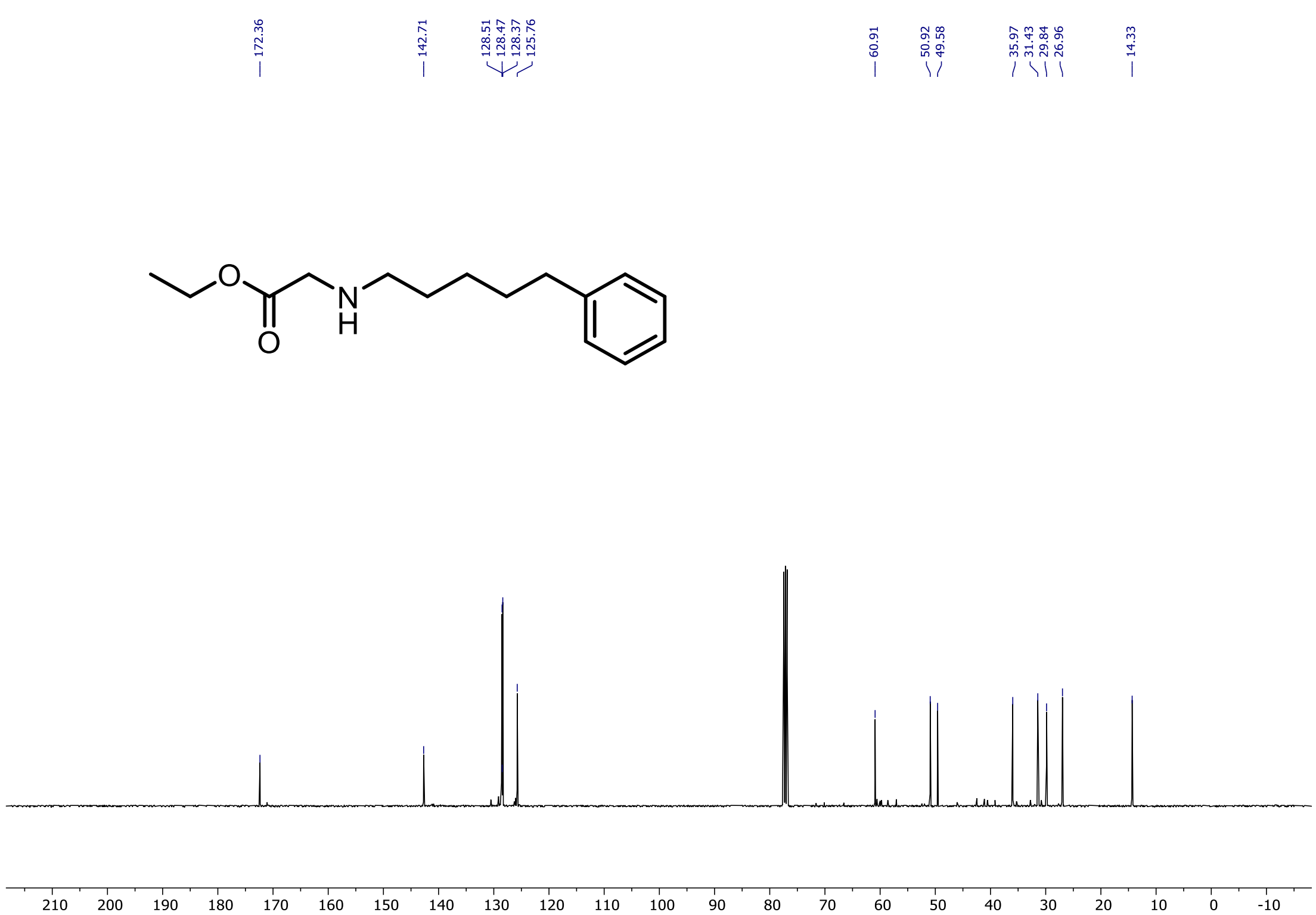

***N,N*-Dimethyl-3-((3-phenylpropyl)amino)propanamide (1.54)**
^{1}H NMR (600 MHz, CDCl$_3$)

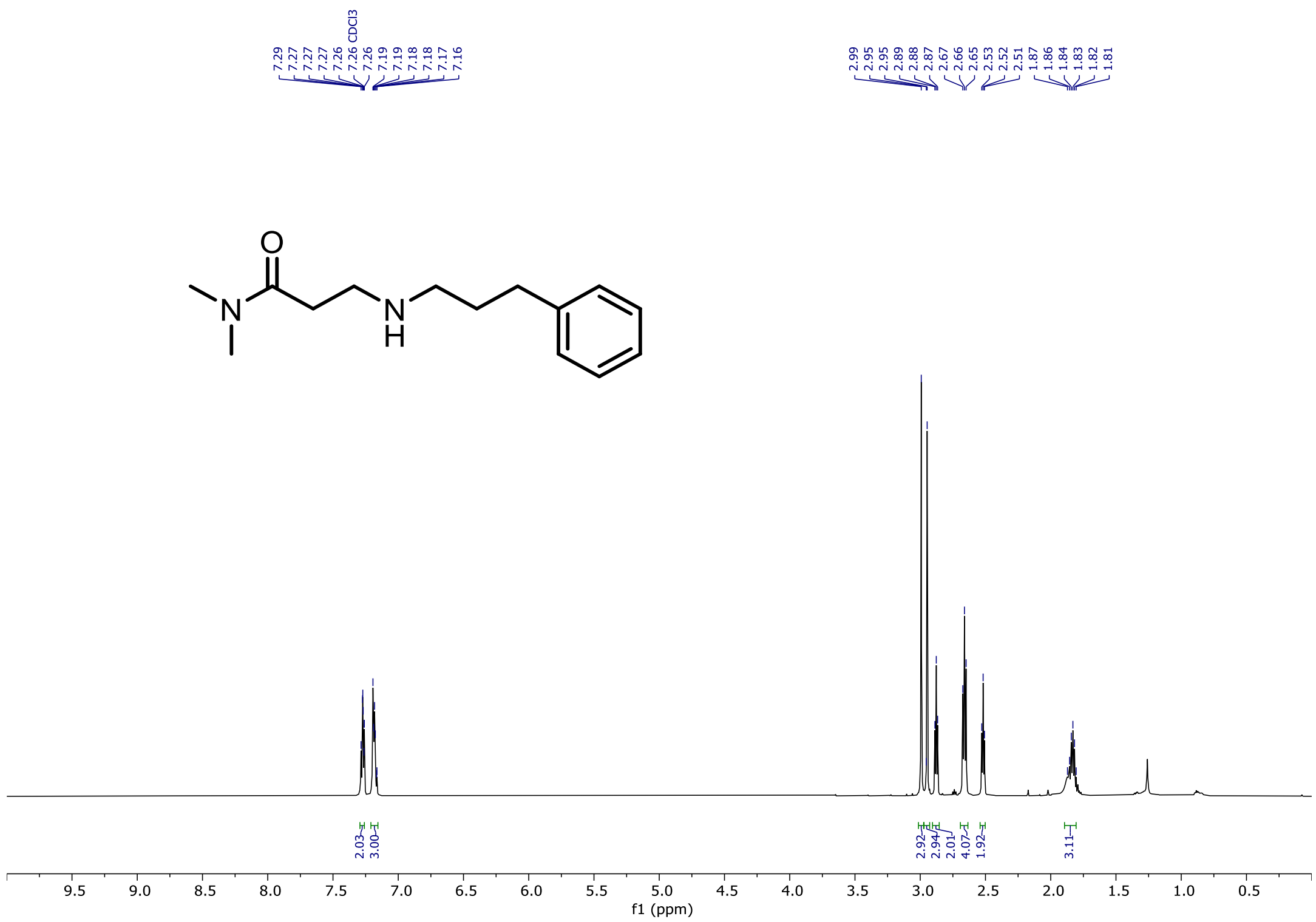

^{13}C NMR (151 MHz, CDCl$_3$)

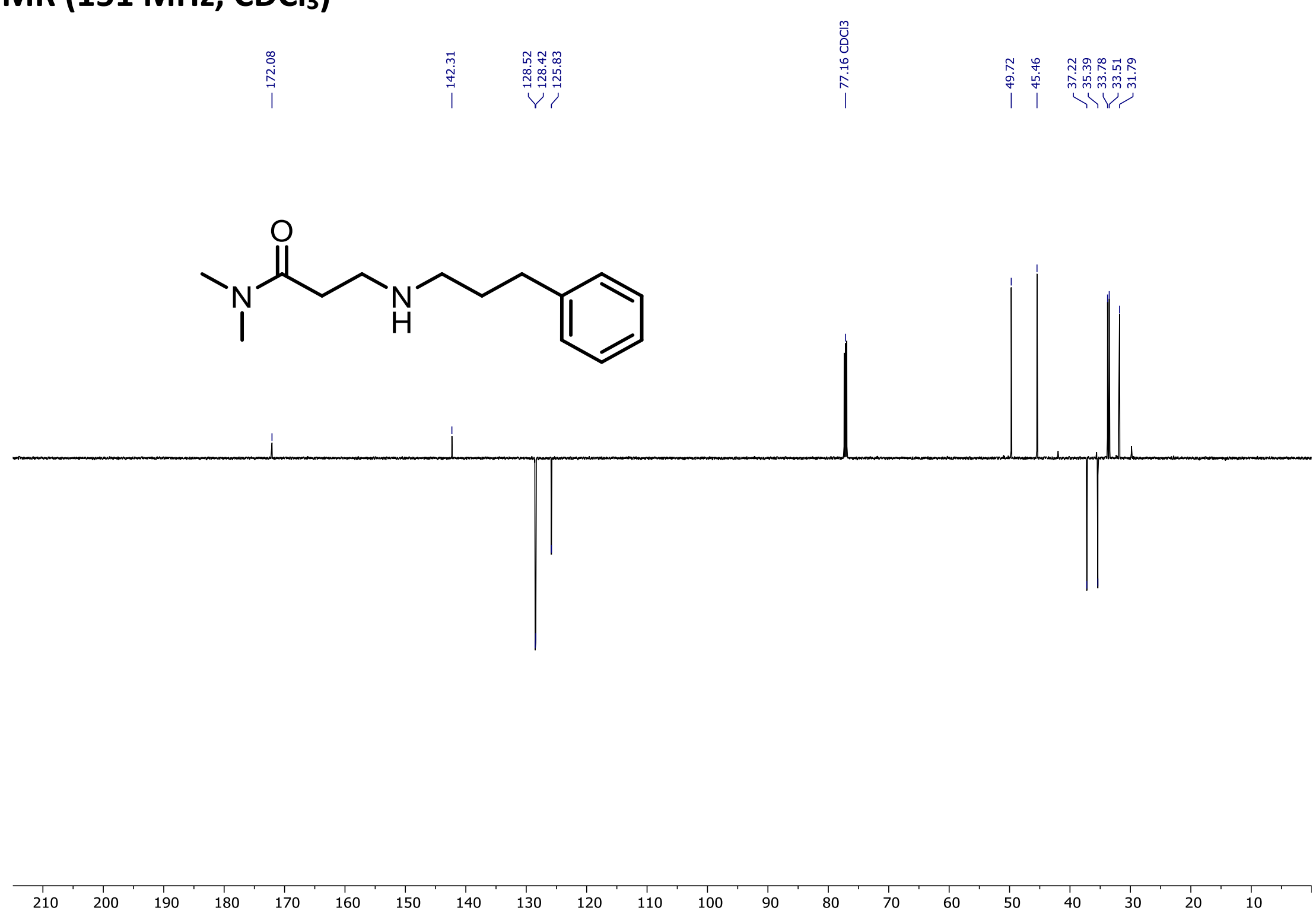

N-Pentyl-5-phenylpentan-1-amine (1.55)

^{1}H NMR (600 MHz, $CDCl_3$)

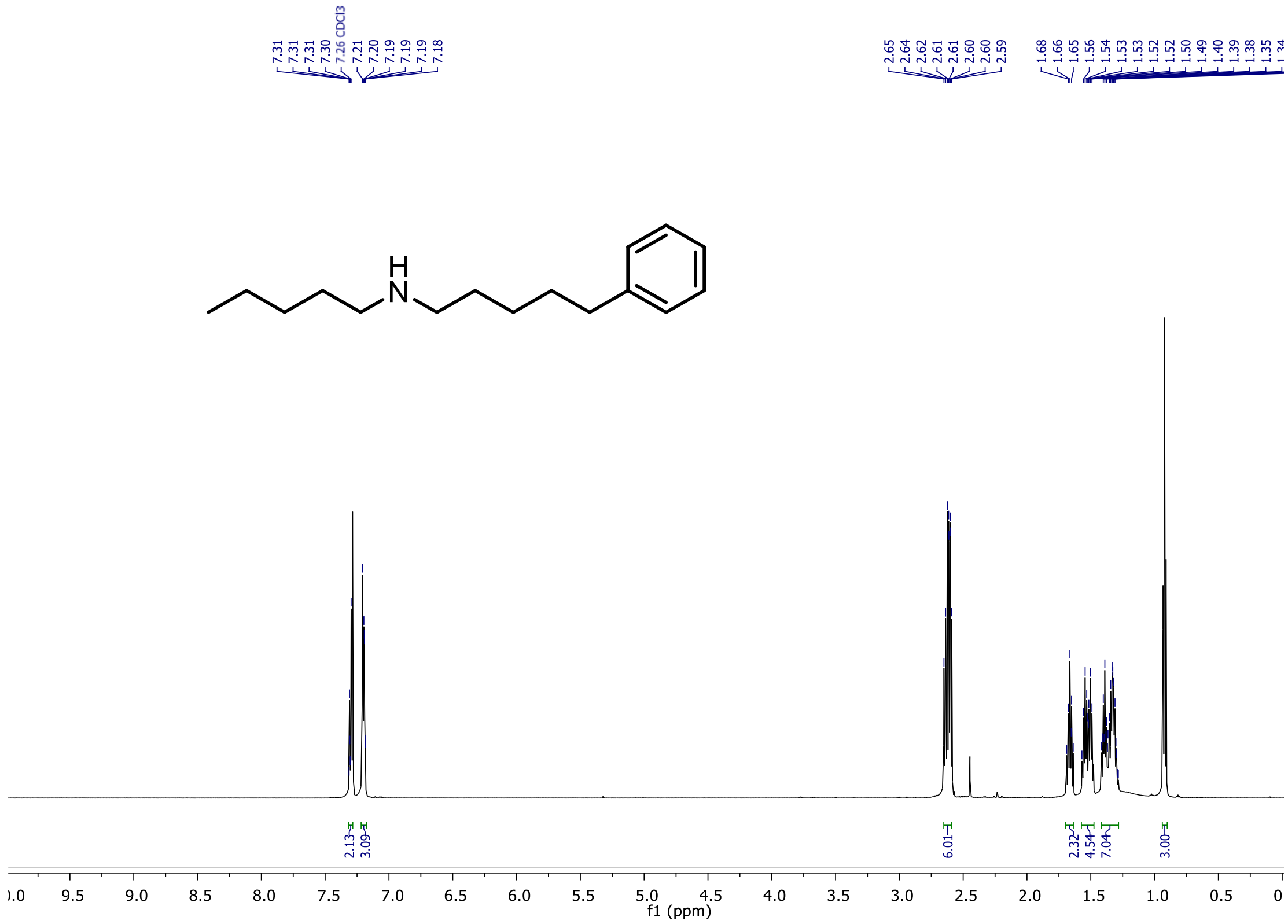

^{13}C NMR (151 MHz, $CDCl_3$)

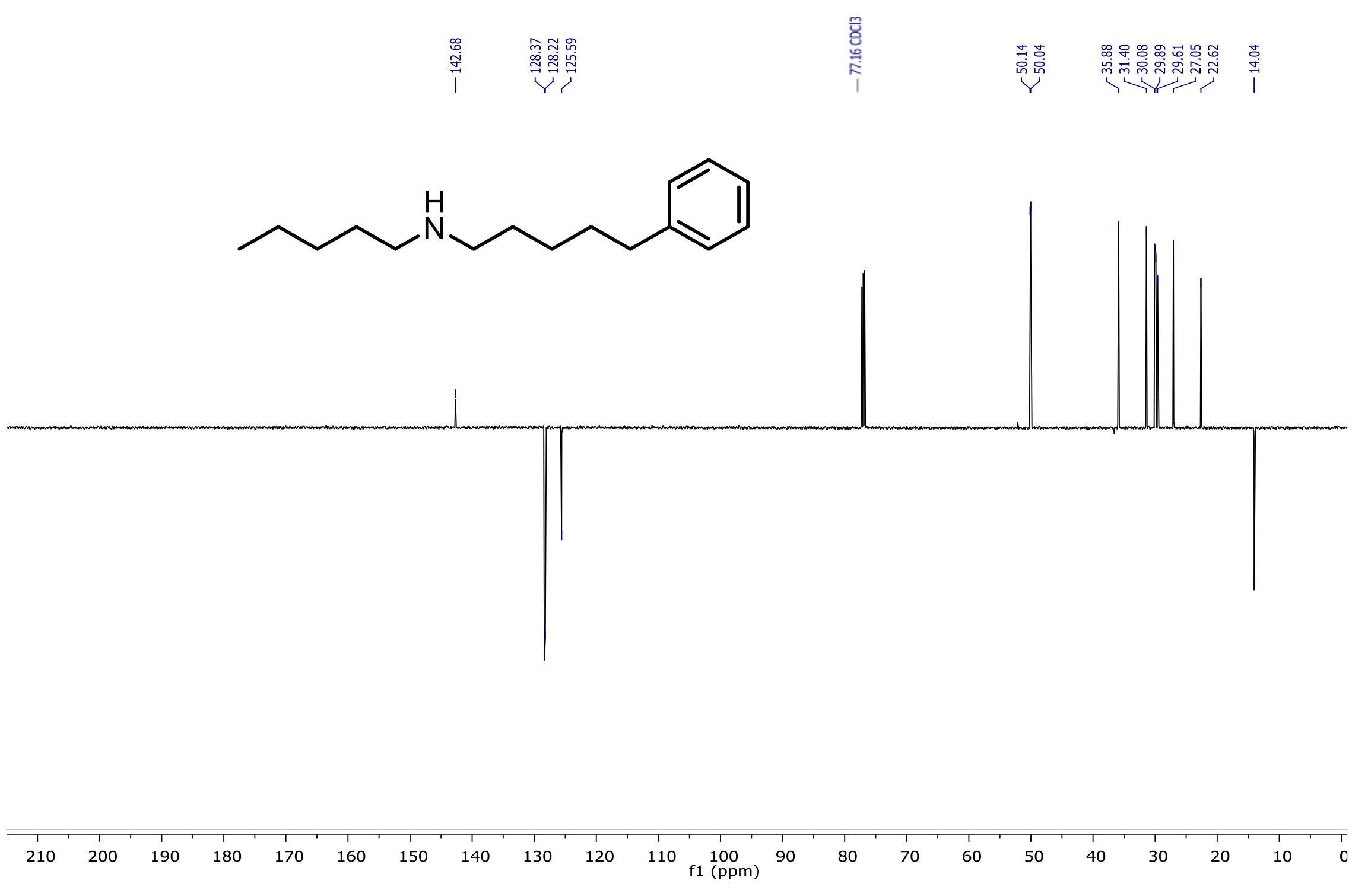

***N*-Phenethylpentan-1-amine (1.56)**

^{1}H NMR (400 MHz, $CDCl_3$)

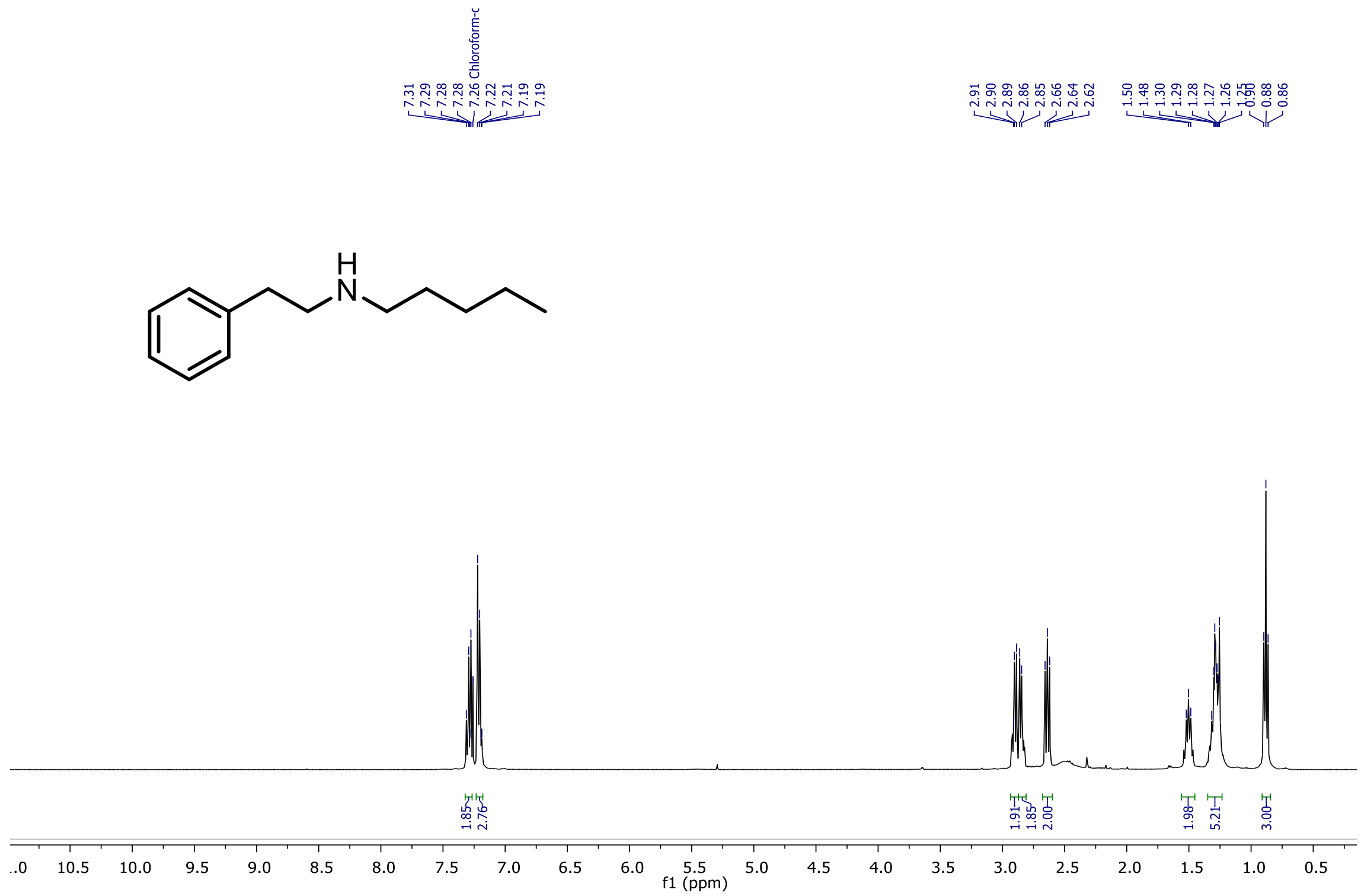

^{13}C NMR (101 MHz, $CDCl_3$)

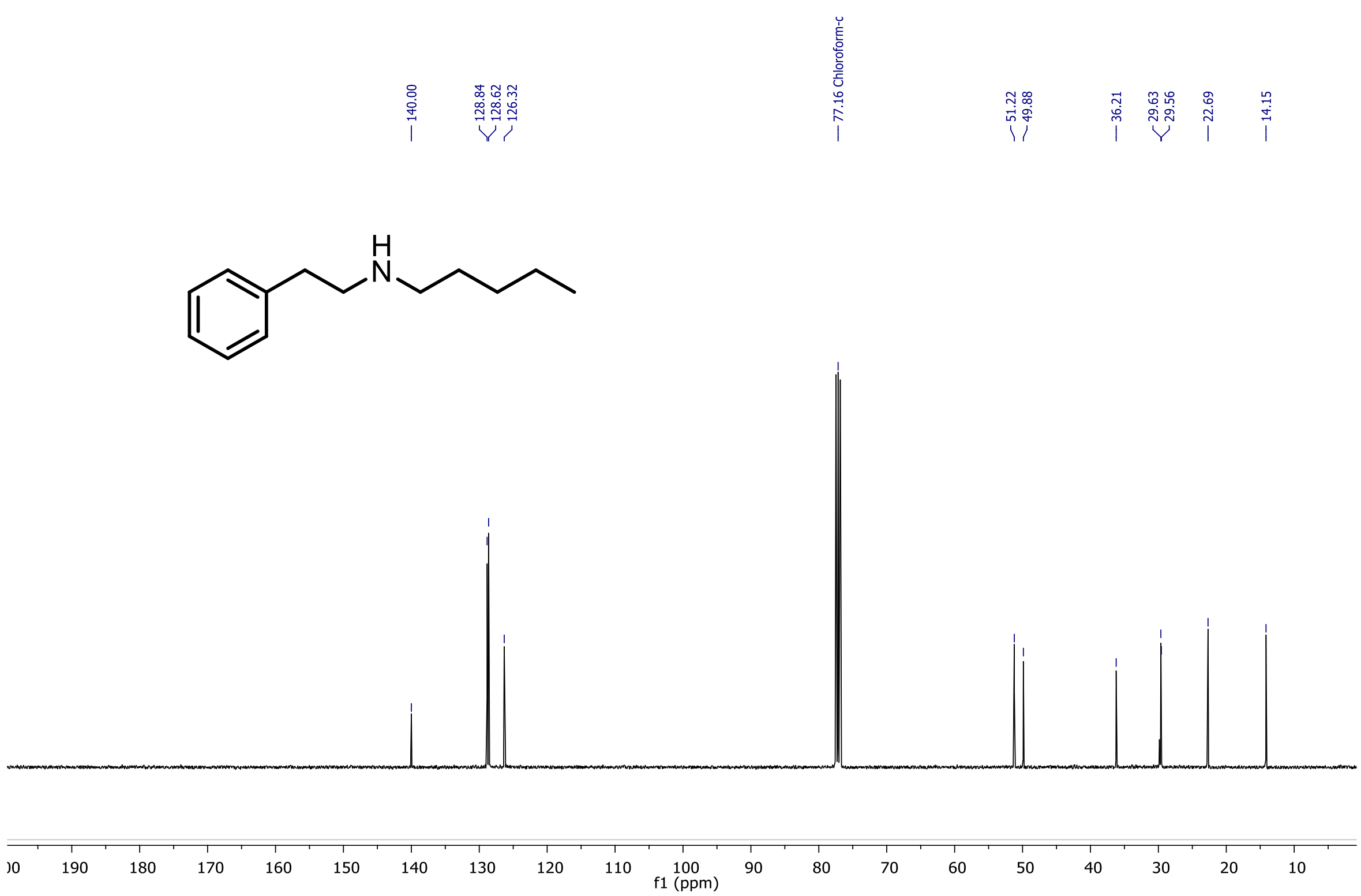

3-(Naphthalen-2-yl)-*N*-phenethylpropan-1-amine (1.57)
^{1}H NMR (600 MHz, CDCl$_3$)

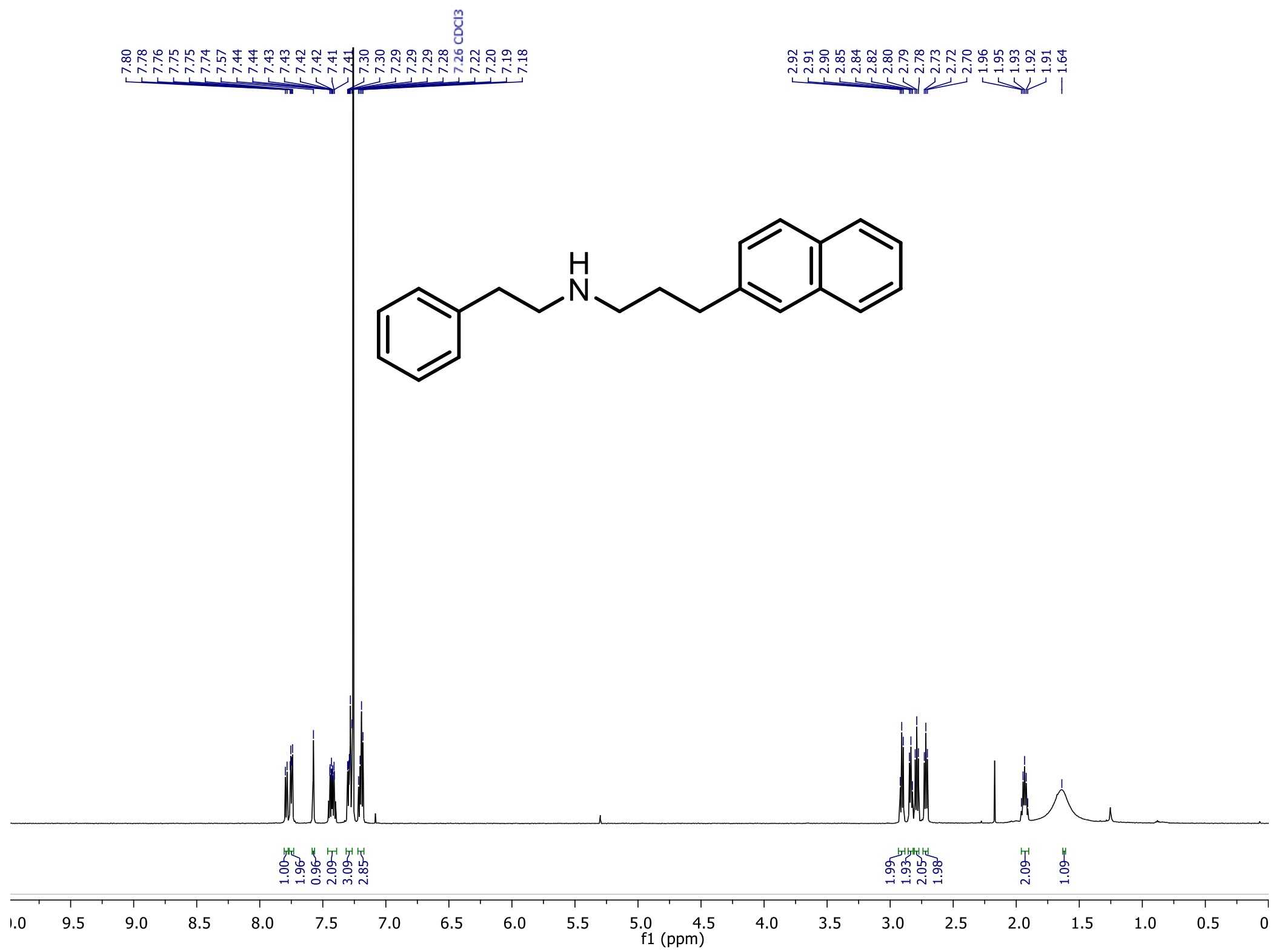

^{13}C NMR (151 MHz, CDCl$_3$)

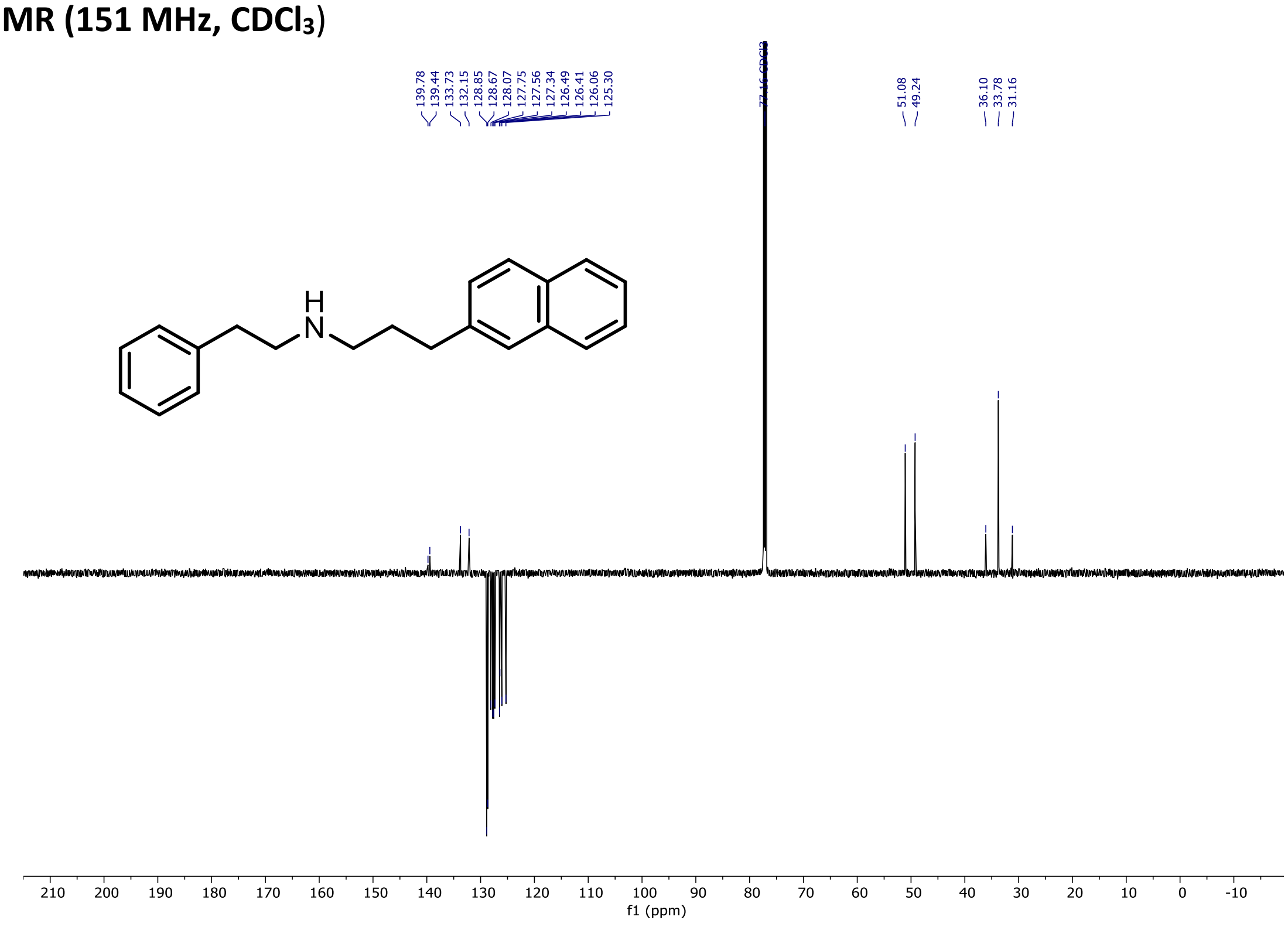

***N*-Phenethylheptan-1-amine (1.58)**

^{1}H NMR (600 MHz, $CDCl_3$)

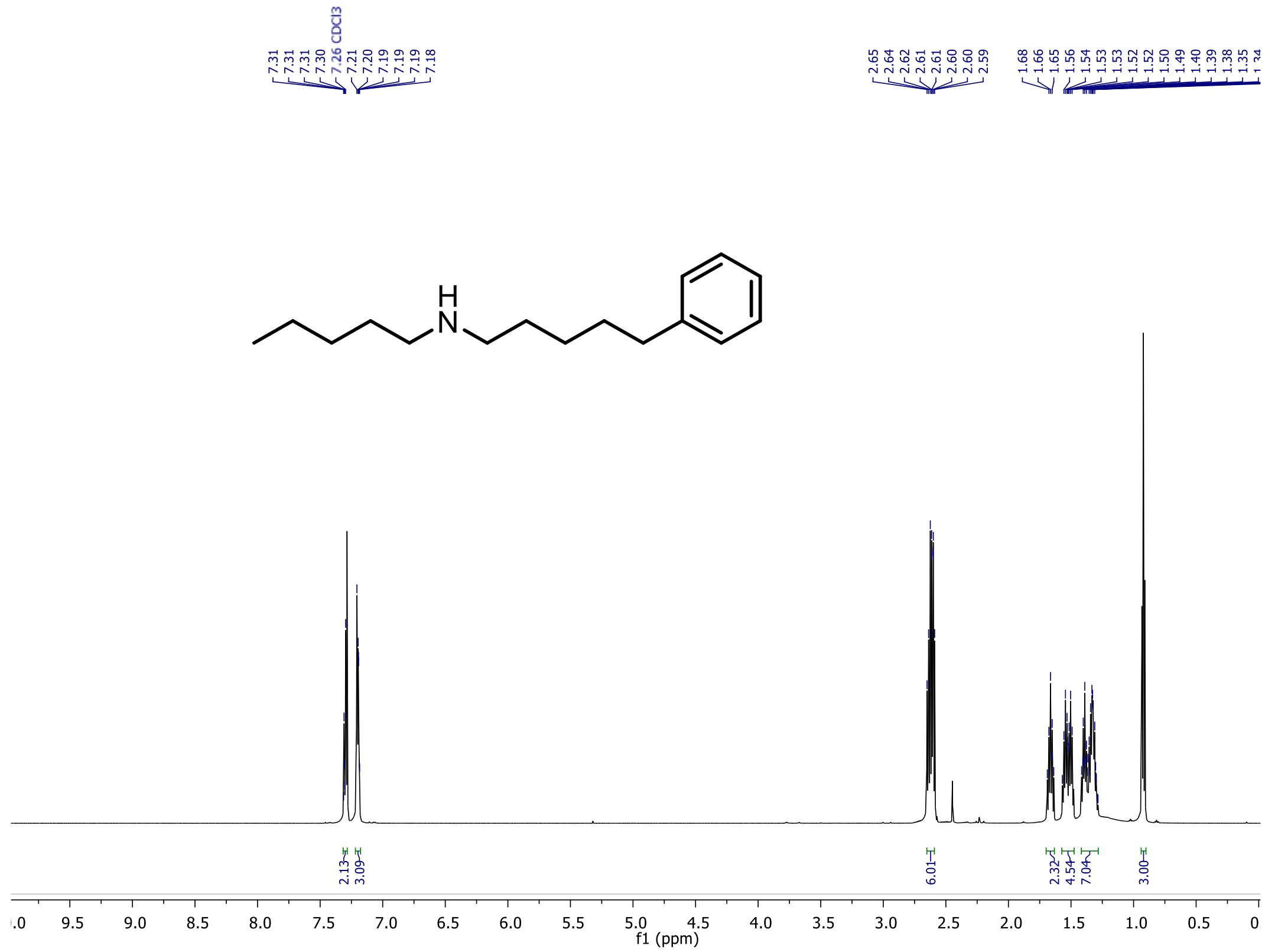

^{13}C NMR (151 MHz, $CDCl_3$)

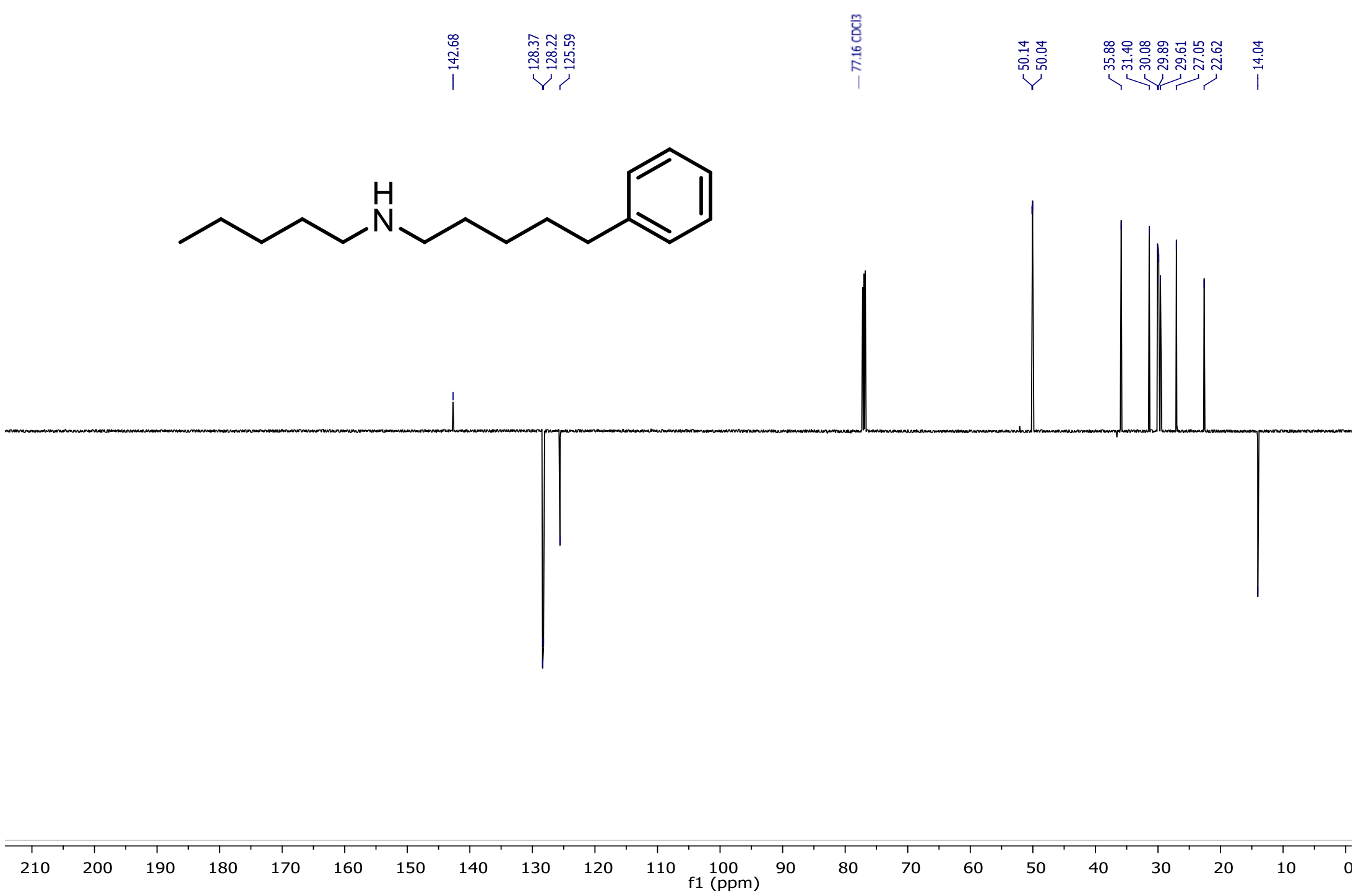

***N*-(Cyclohexylmethyl)-2-phenylethan-1-amine (1.59)**
^{1}H NMR (600 MHz, CDCl$_3$)

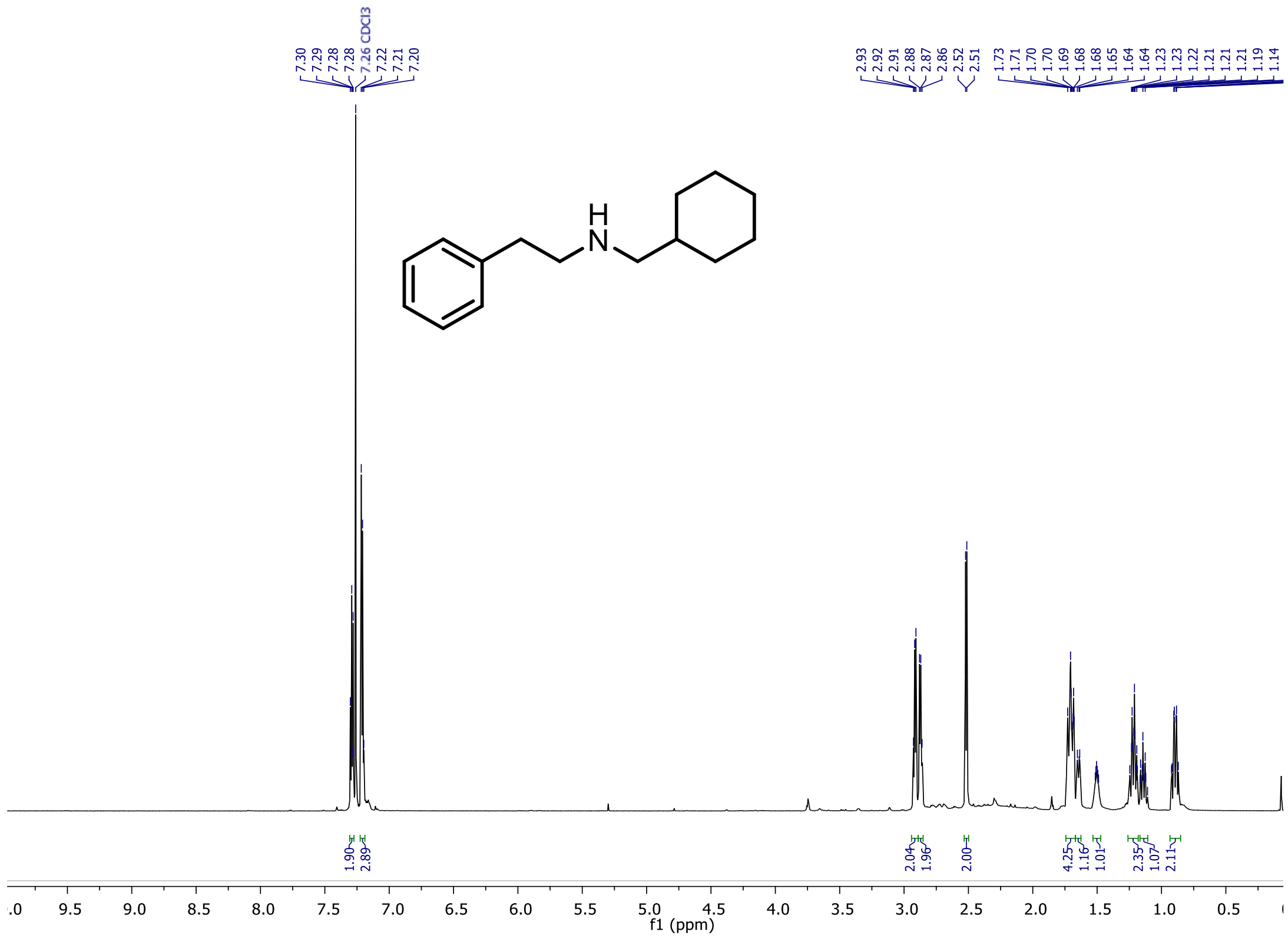

^{13}C NMR (151 MHz, CDCl$_3$)

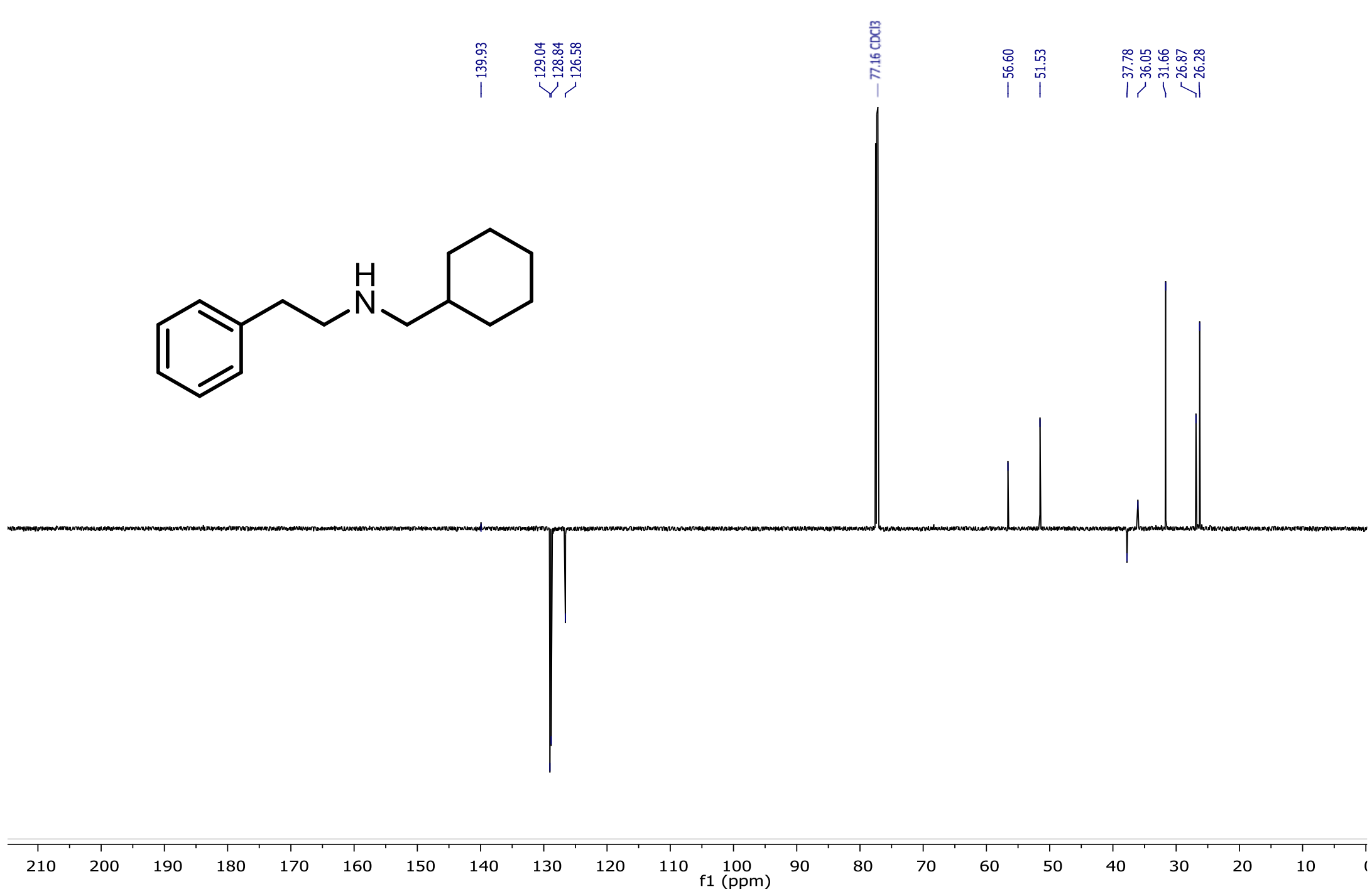

_N_-(5-Phenylpentyl)cyclohexanamine (1.60)

^{1}H NMR (600 MHz, $CDCl_3$)

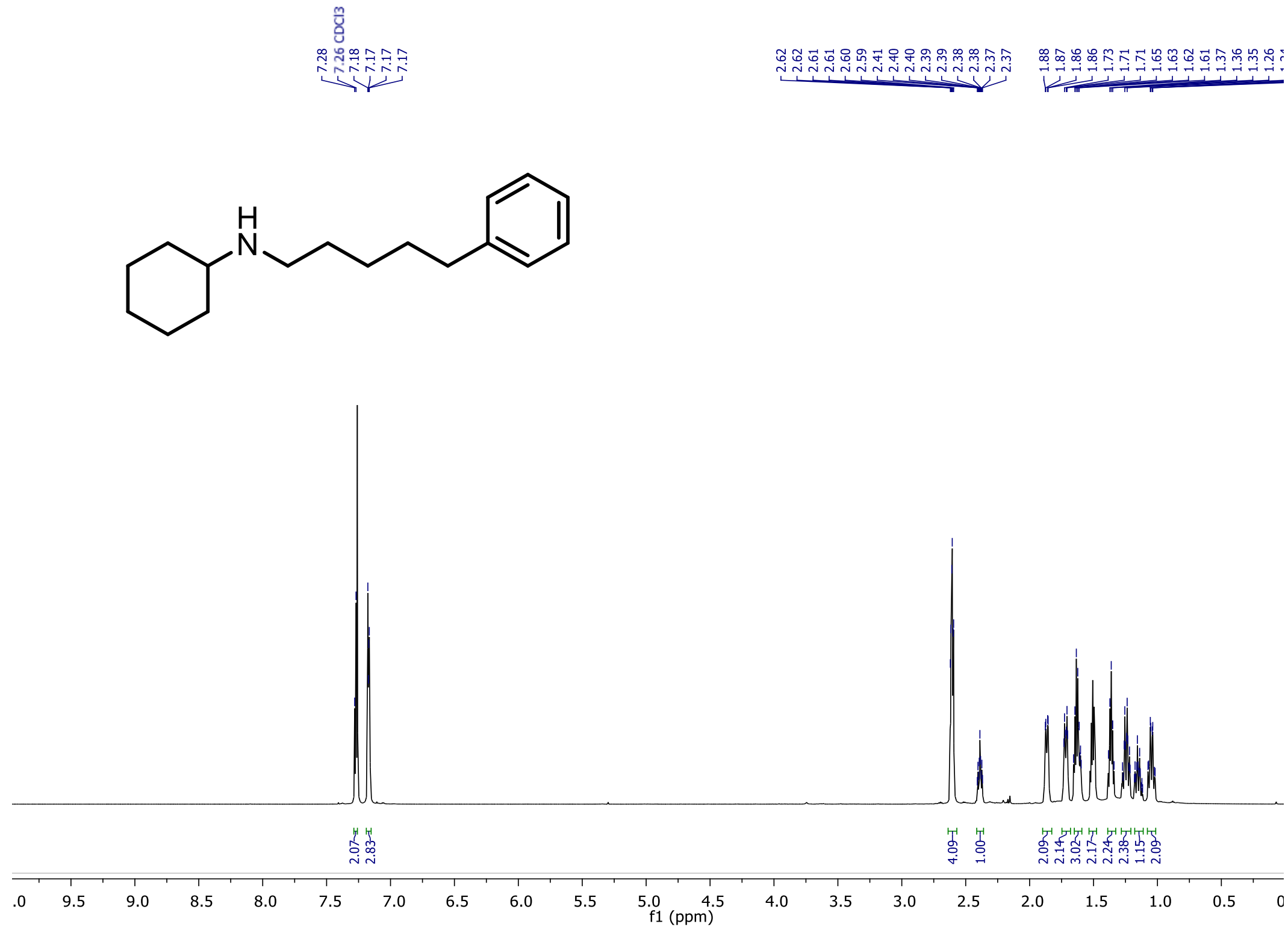

^{13}C NMR (151 MHz, $CDCl_3$)

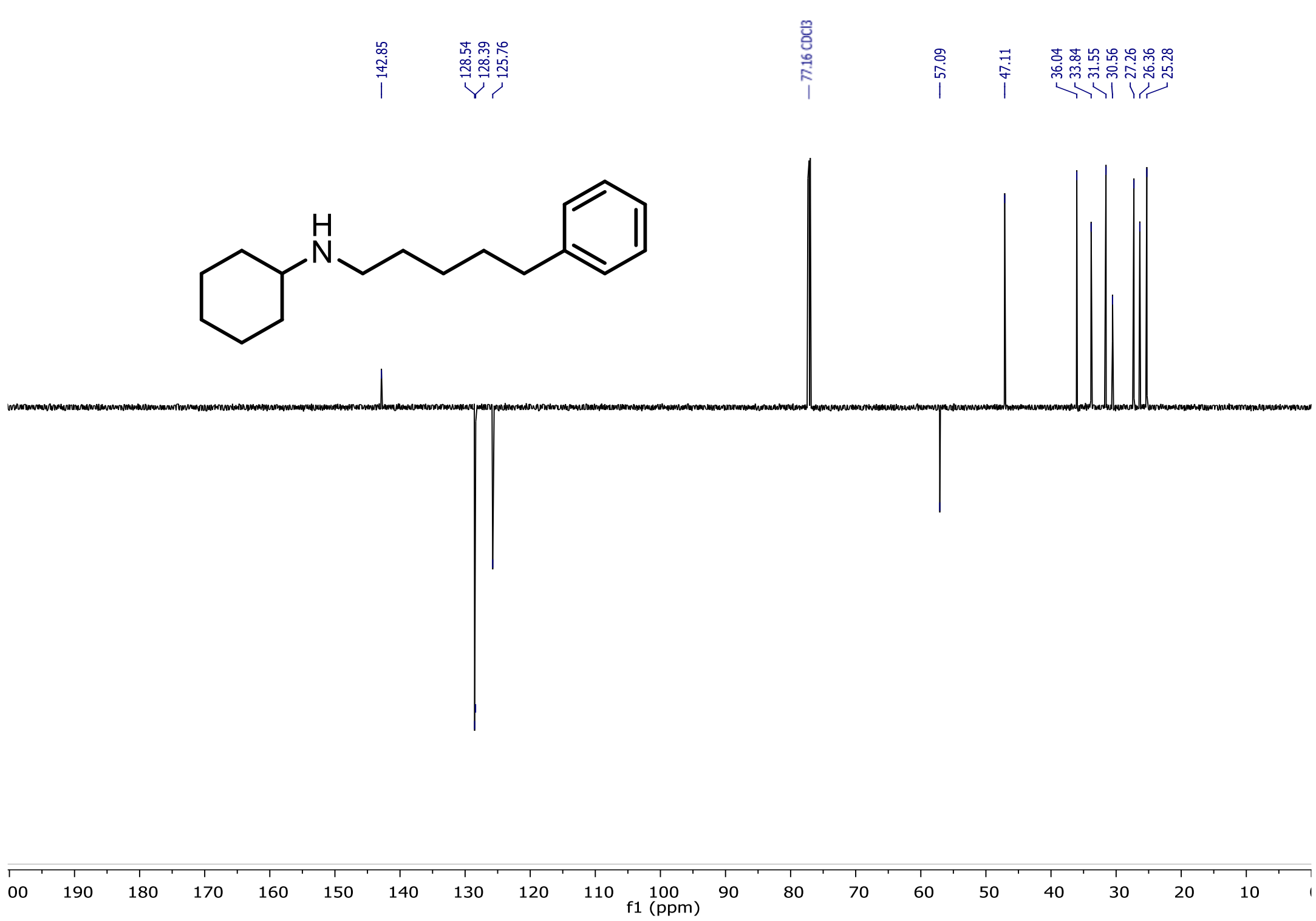

_N_-Phenethyldodecan-1-amine (1.61)
^{1}H NMR (600 MHz, $CDCl_3$)

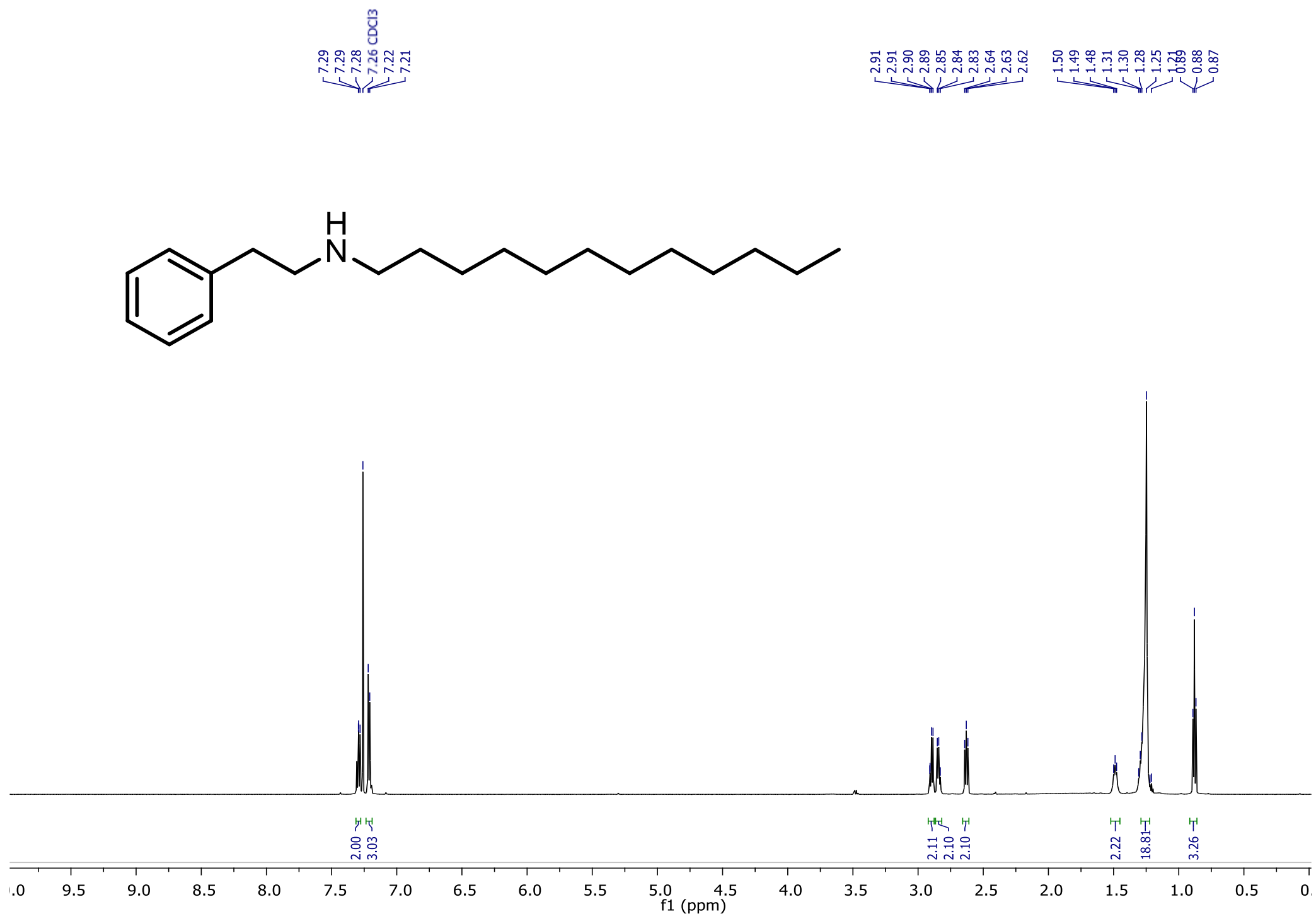

^{13}C NMR (151 MHz, $CDCl_3$)

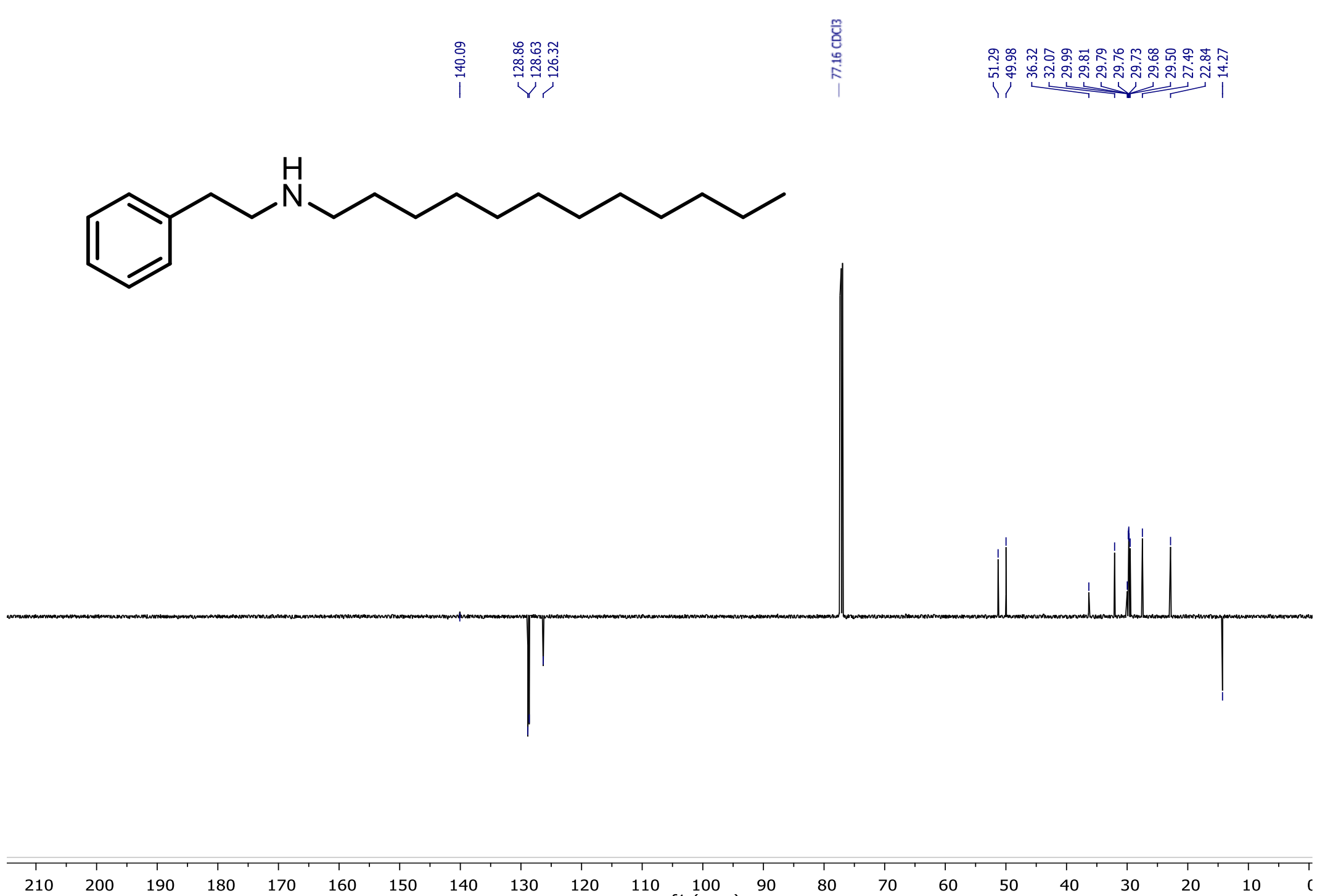

***N*-Phenethyl-3-(thiophen-3-yl)propan-1-amine (1.62)**

^{1}H NMR (600 MHz, $CDCl_3$)

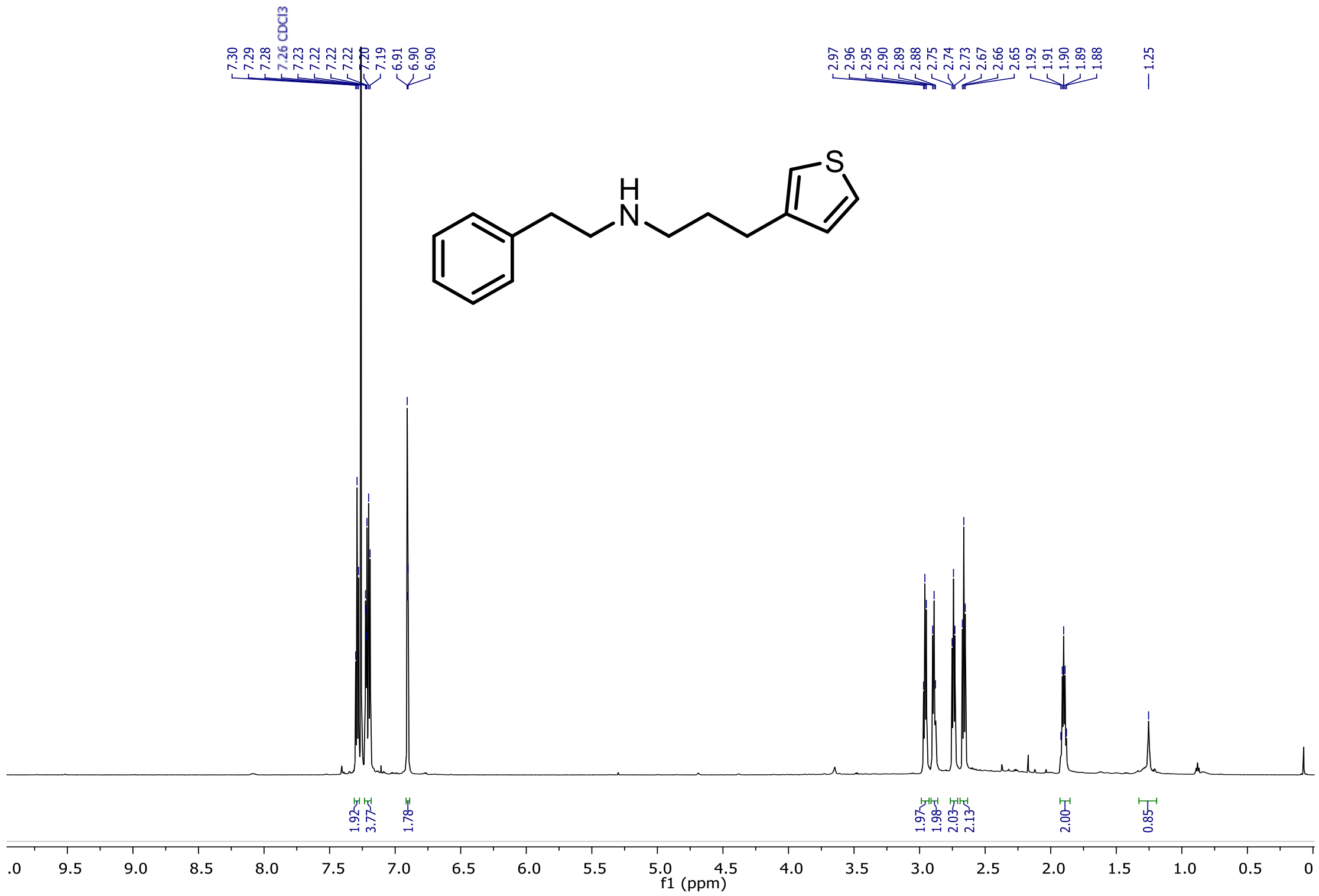

^{13}C NMR (151 MHz, $CDCl_3$)

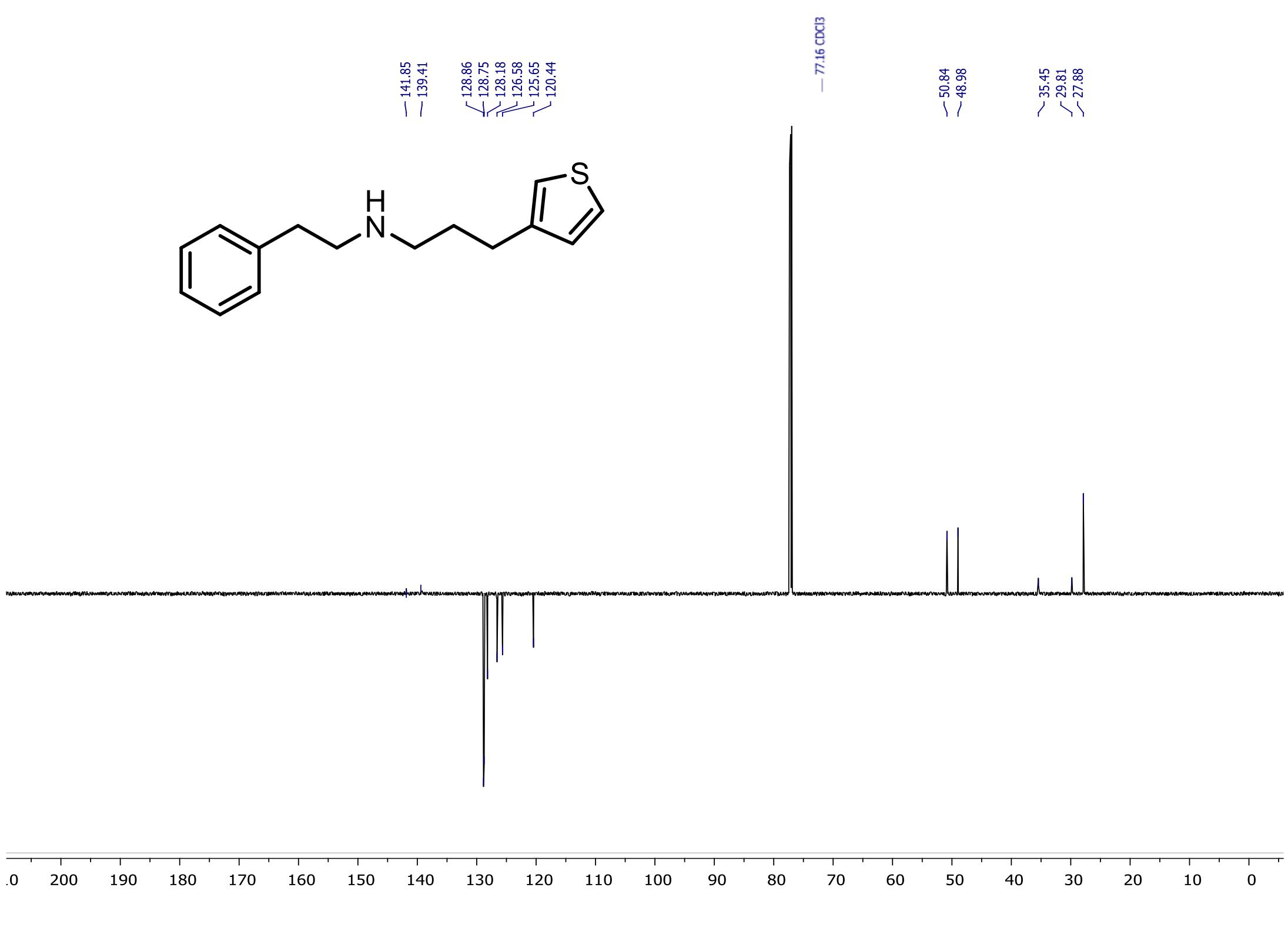

N-Phenethyl-3-(thiophen-3-yl)propan-1-amine (1.63)

^{1}H NMR (600 MHz, $CDCl_3$)

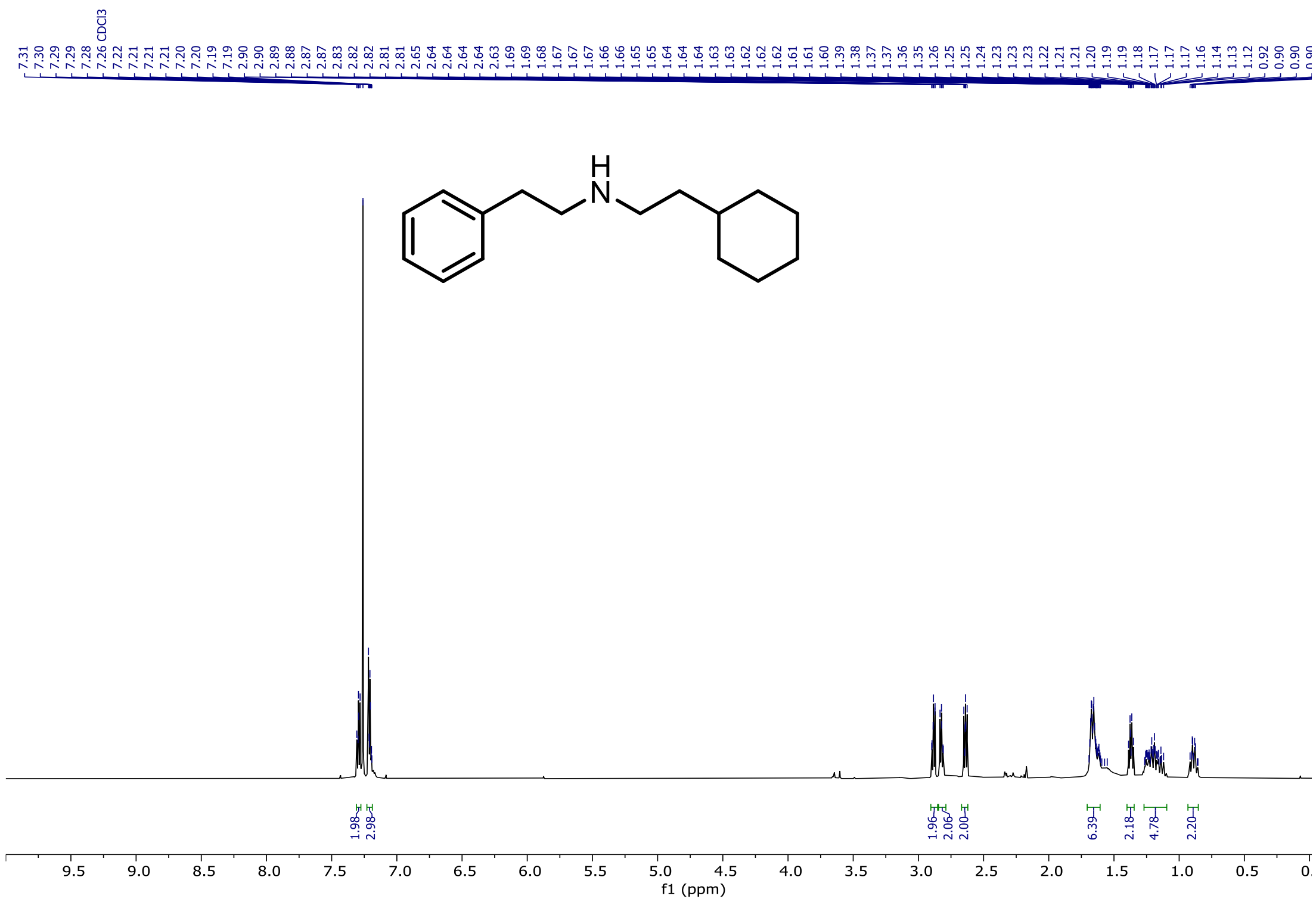

^{13}C NMR (151 MHz, $CDCl_3$)

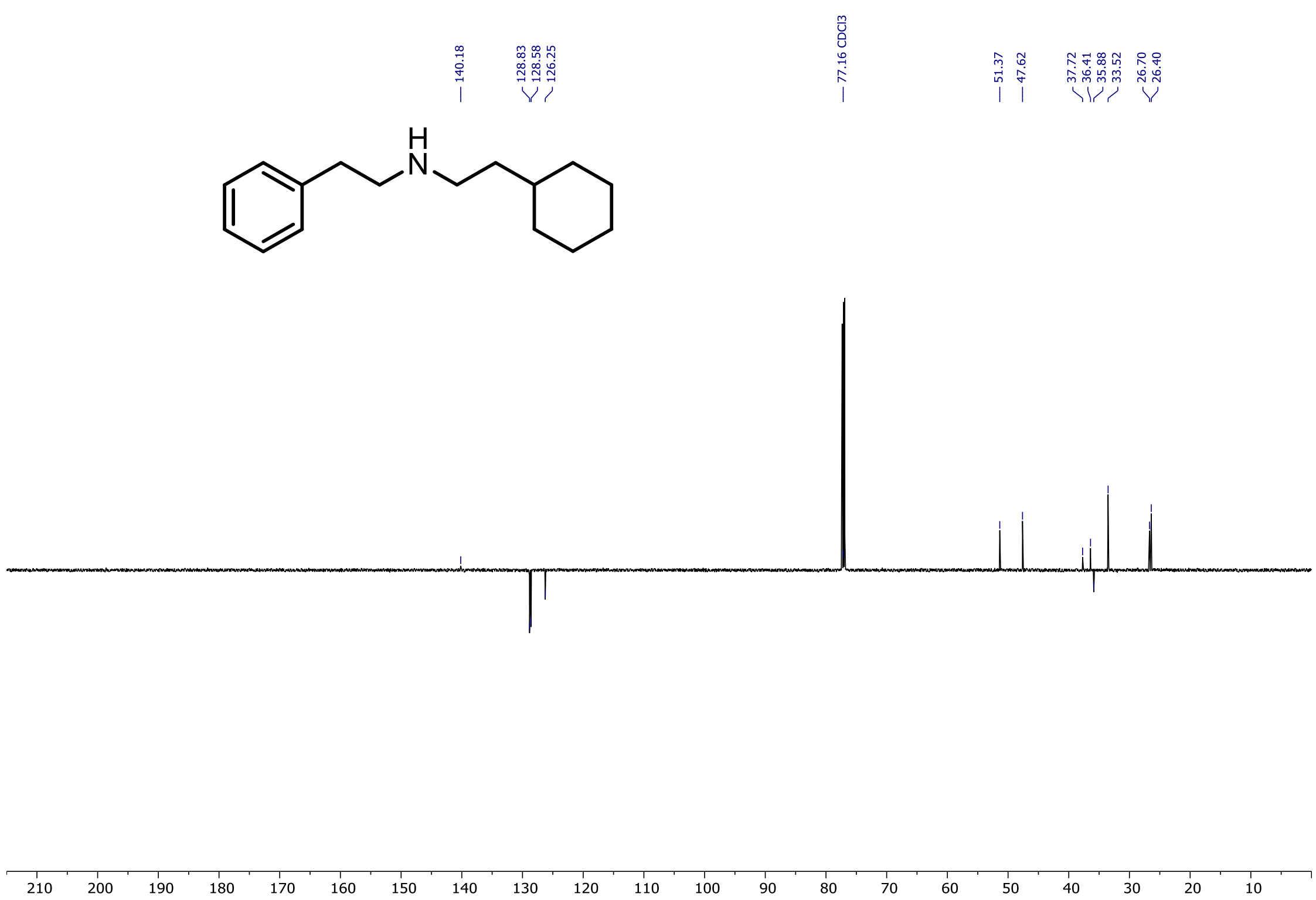

3-(4-Methylcyclohex-3-en-1-yl)-*N*-phenethylbutan-1-amine(1.64)

^{1}H NMR (700 MHz, $CDCl_3$)

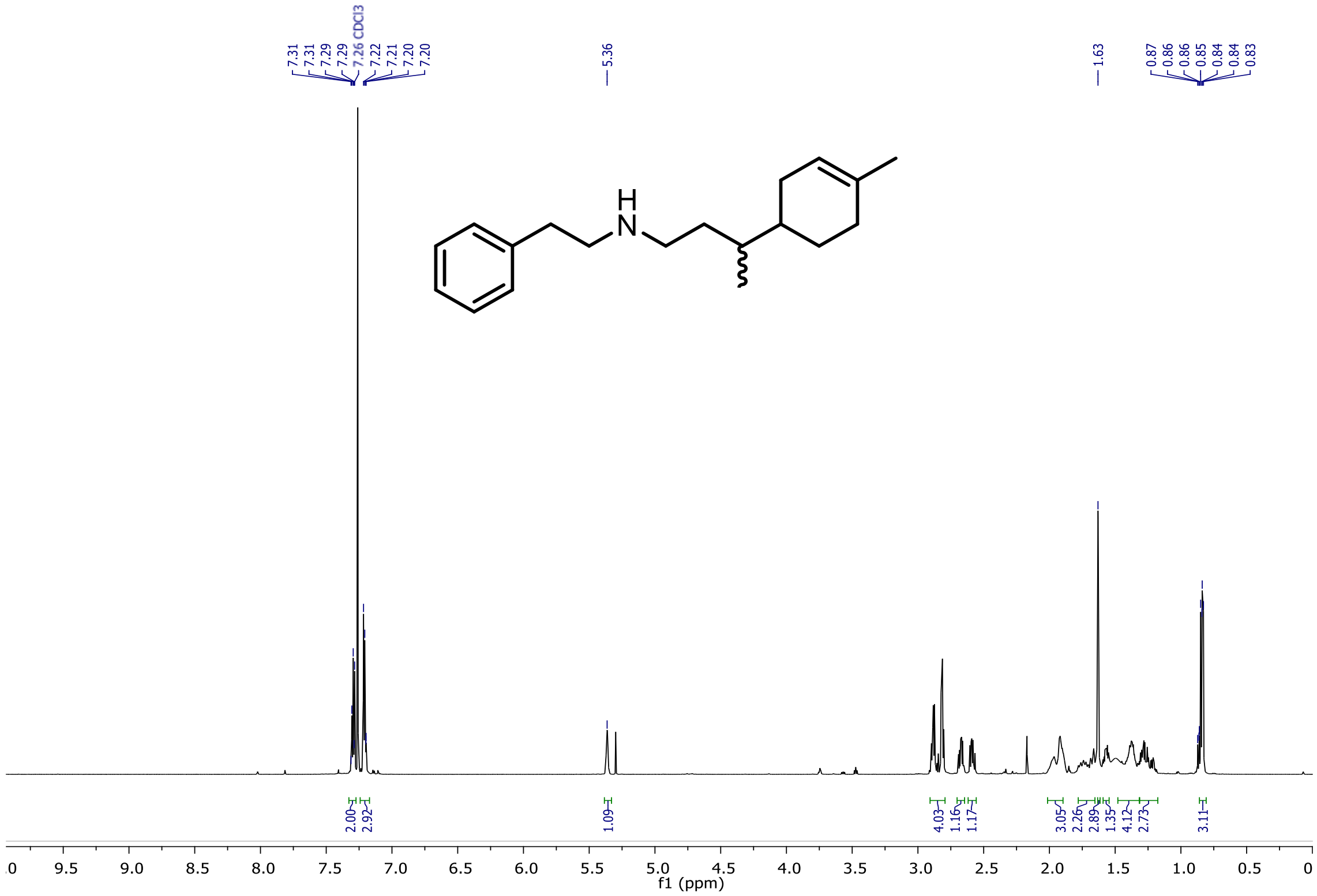

^{13}C NMR (176 MHz, $CDCl_3$)

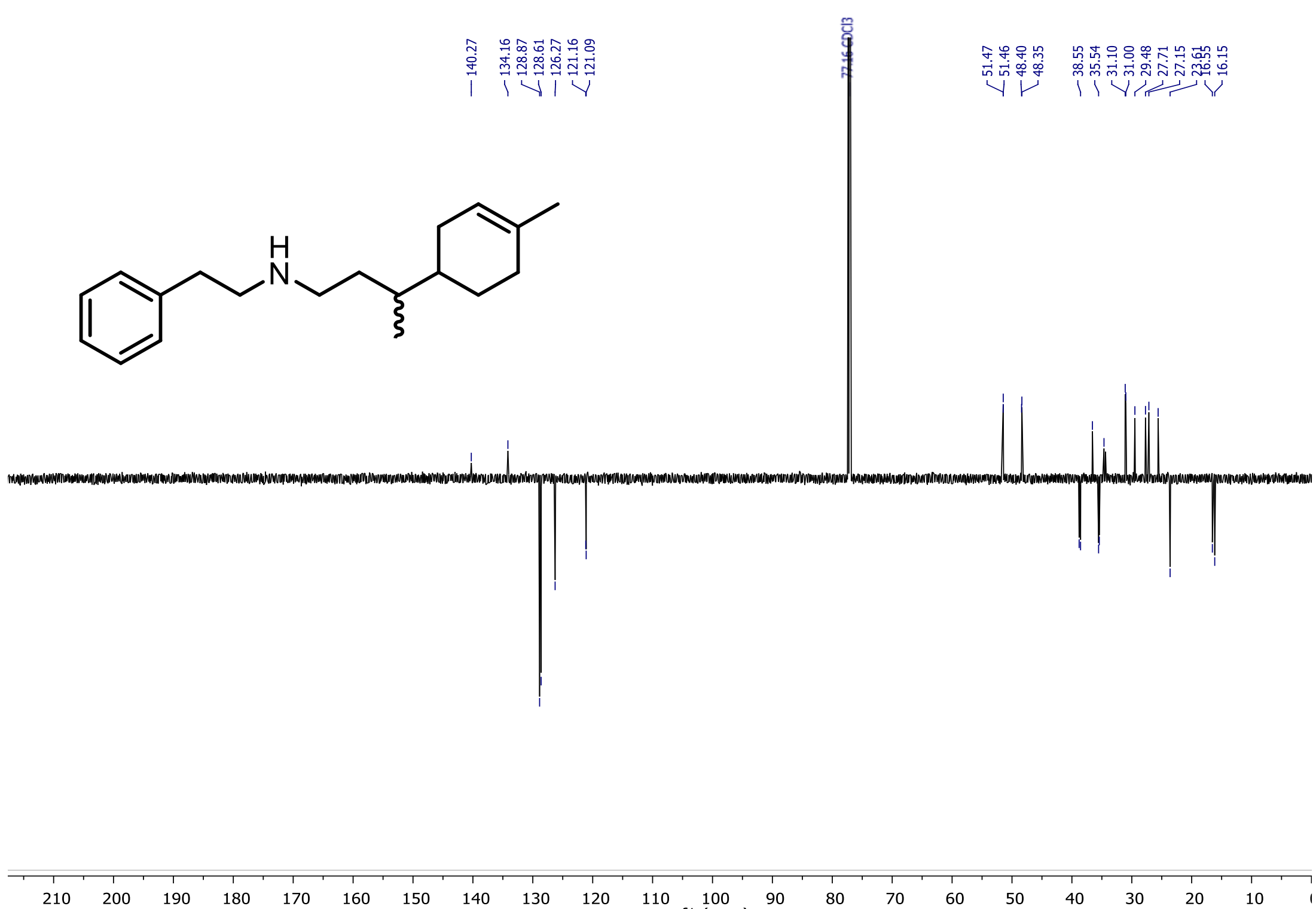

2-Butyl-*N*-phenethylheptan-1-amine (1.65)
^{1}H NMR (600 MHz, $CDCl_3$)

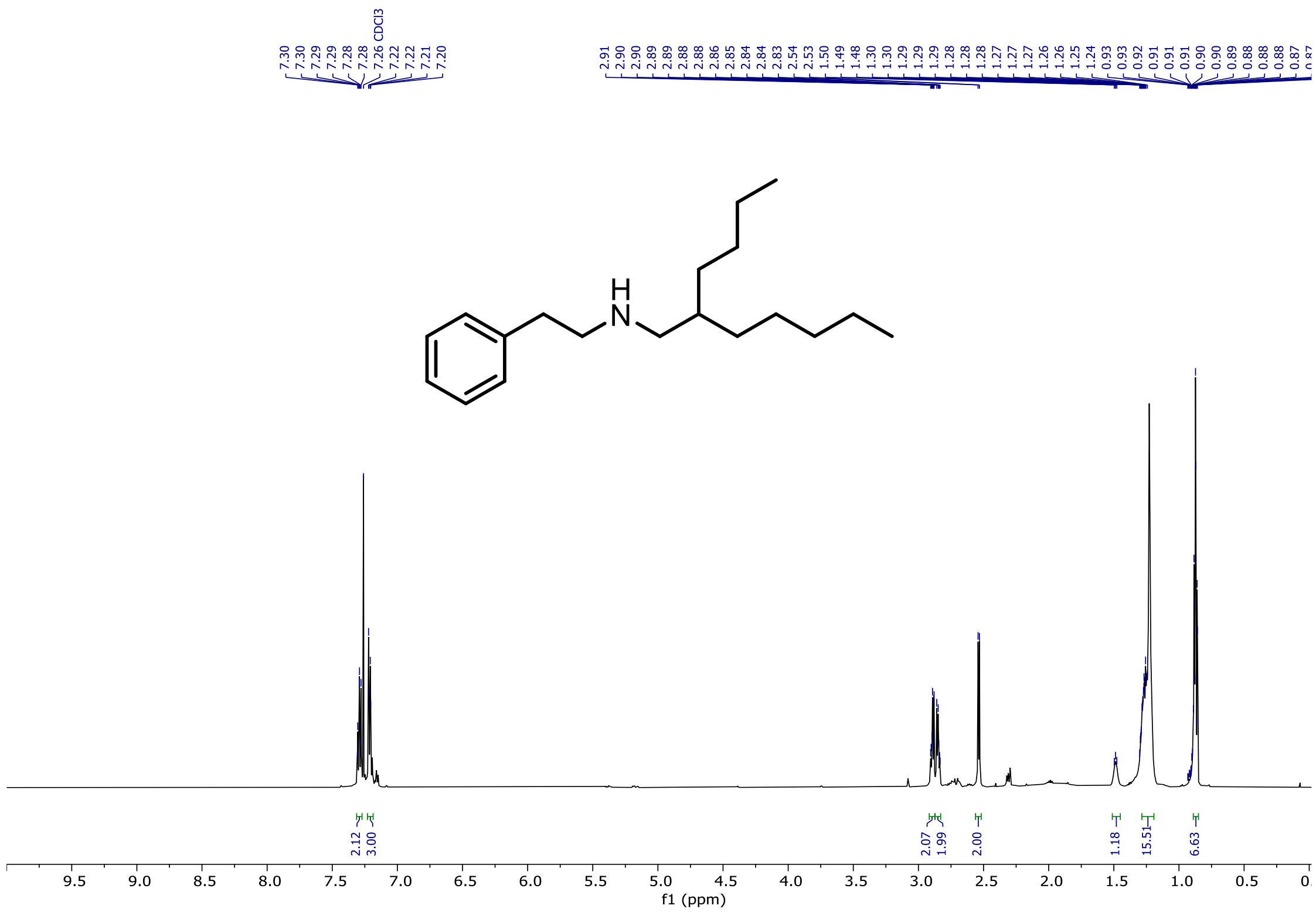

^{13}C NMR (151 MHz, $CDCl_3$)

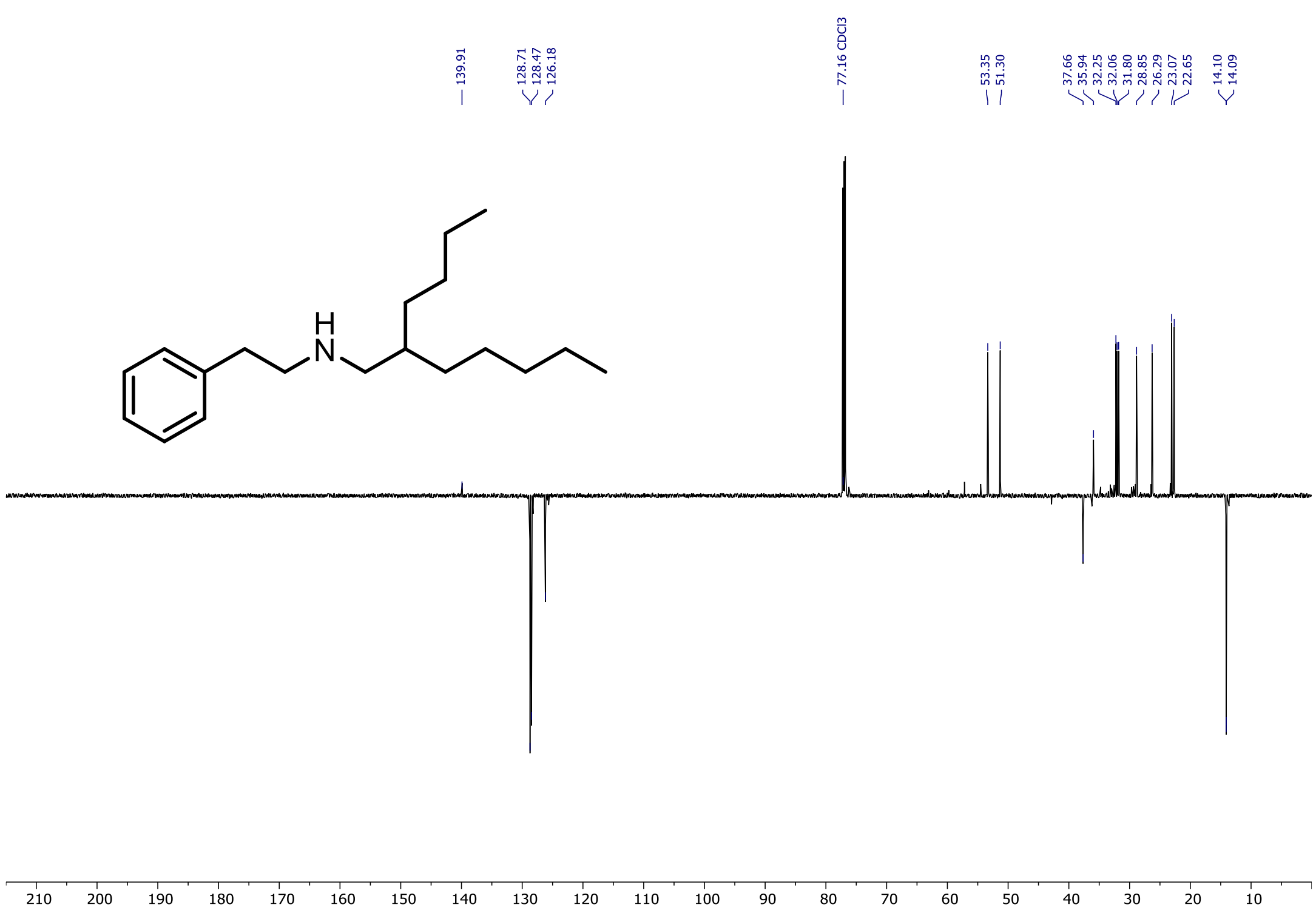

***N*-(Cyclooct-4-en-1-ylmethyl)-2-phenylethan-1-amine (1.66)**
^{1}H NMR (600 MHz, $CDCl_3$)

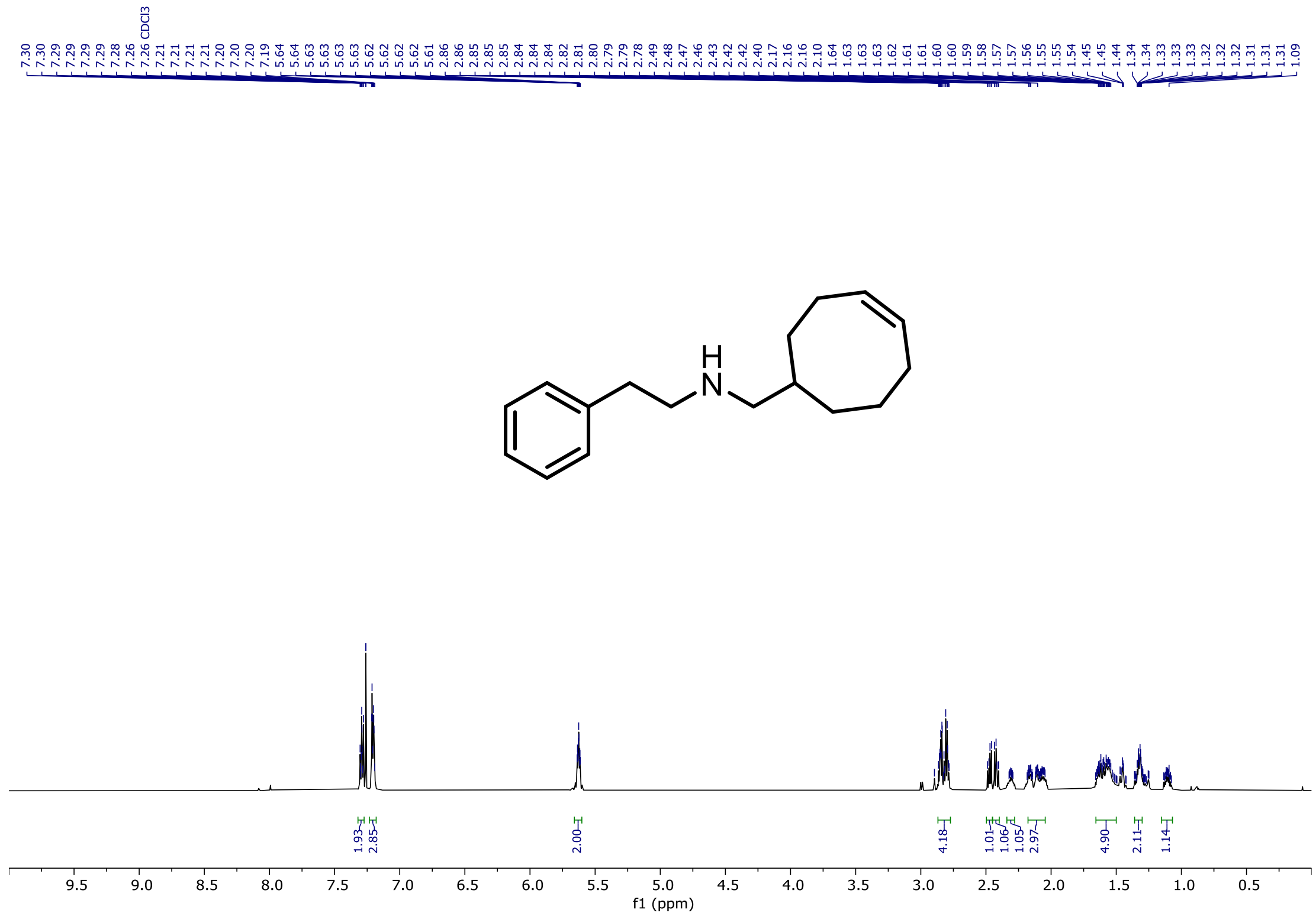

^{13}C NMR (151 MHz, $CDCl_3$)

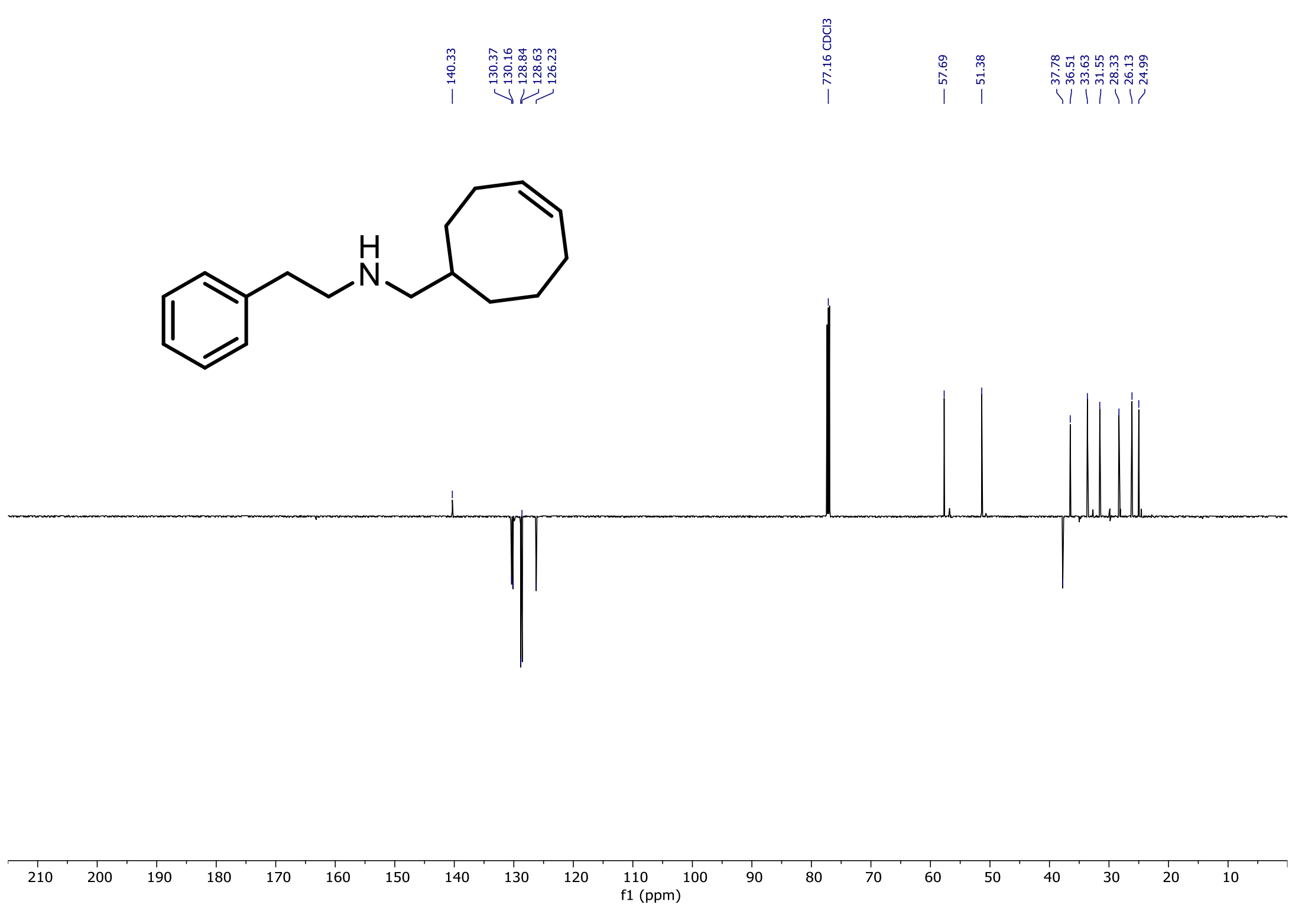

Cinacalcet (2.01)

^{1}H NMR (600 MHz, CDCl$_3$)

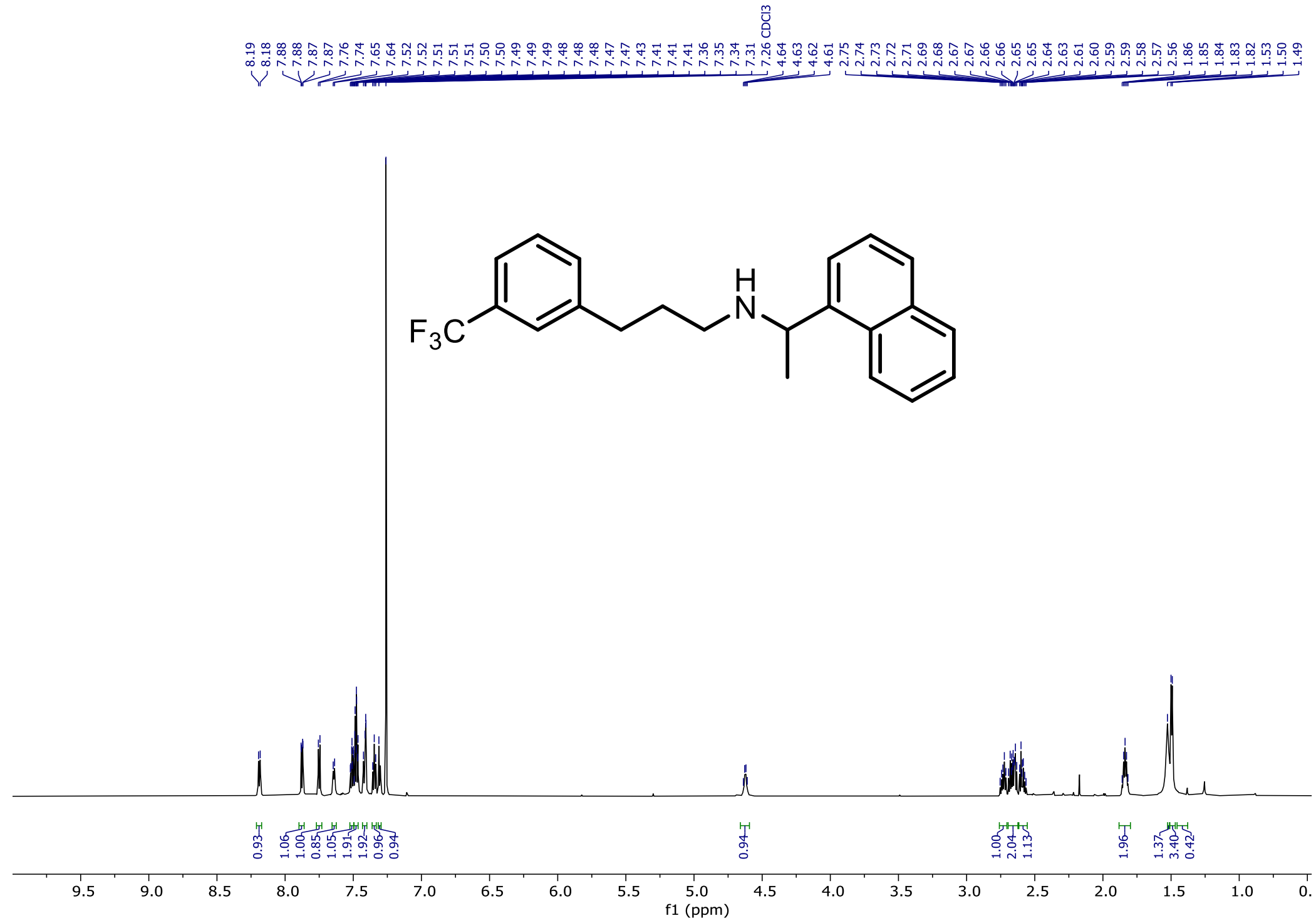

^{13}C NMR (151 MHz, CDCl$_3$)

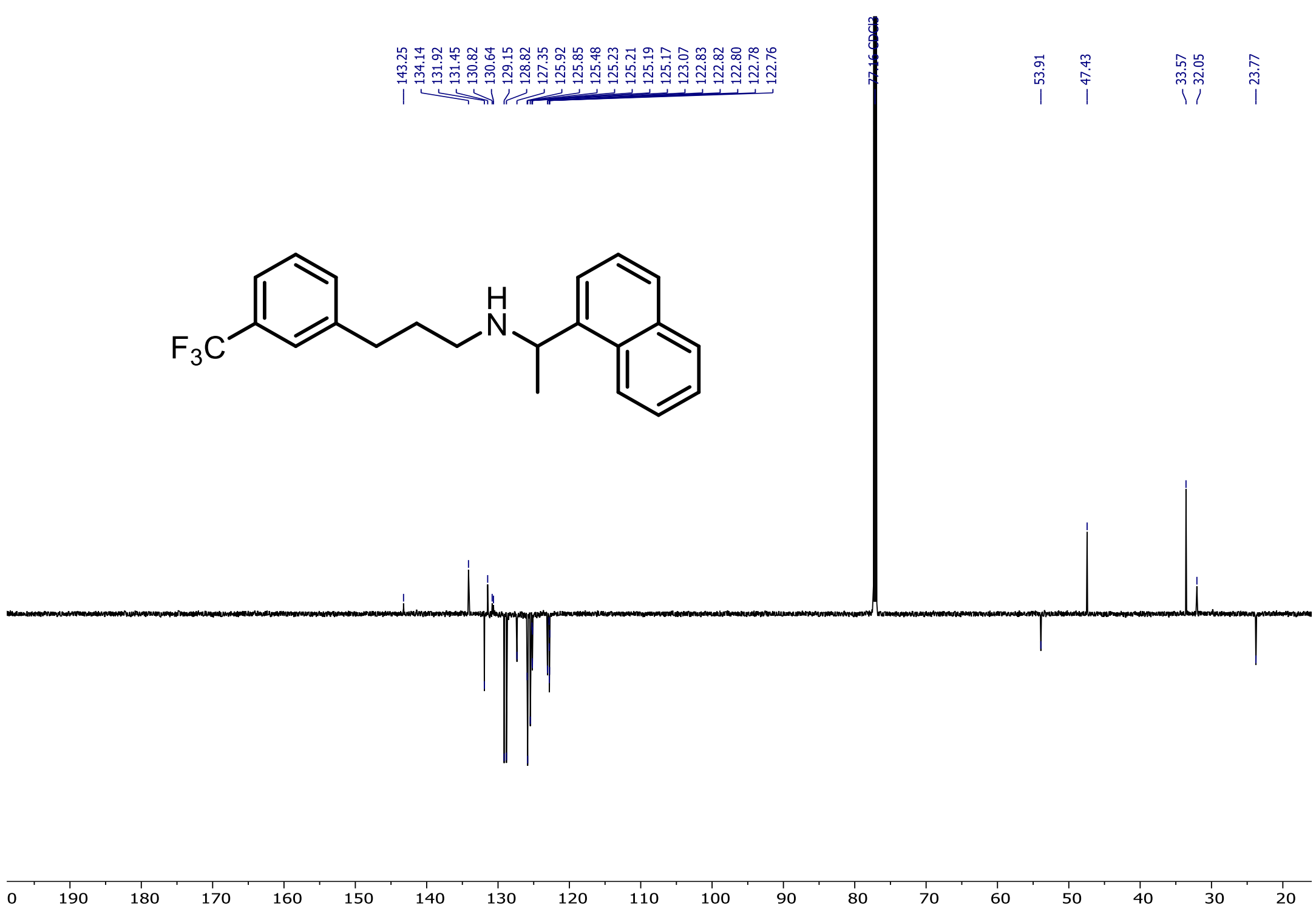

^{19}F (376 MHz, $CDCl_3$)

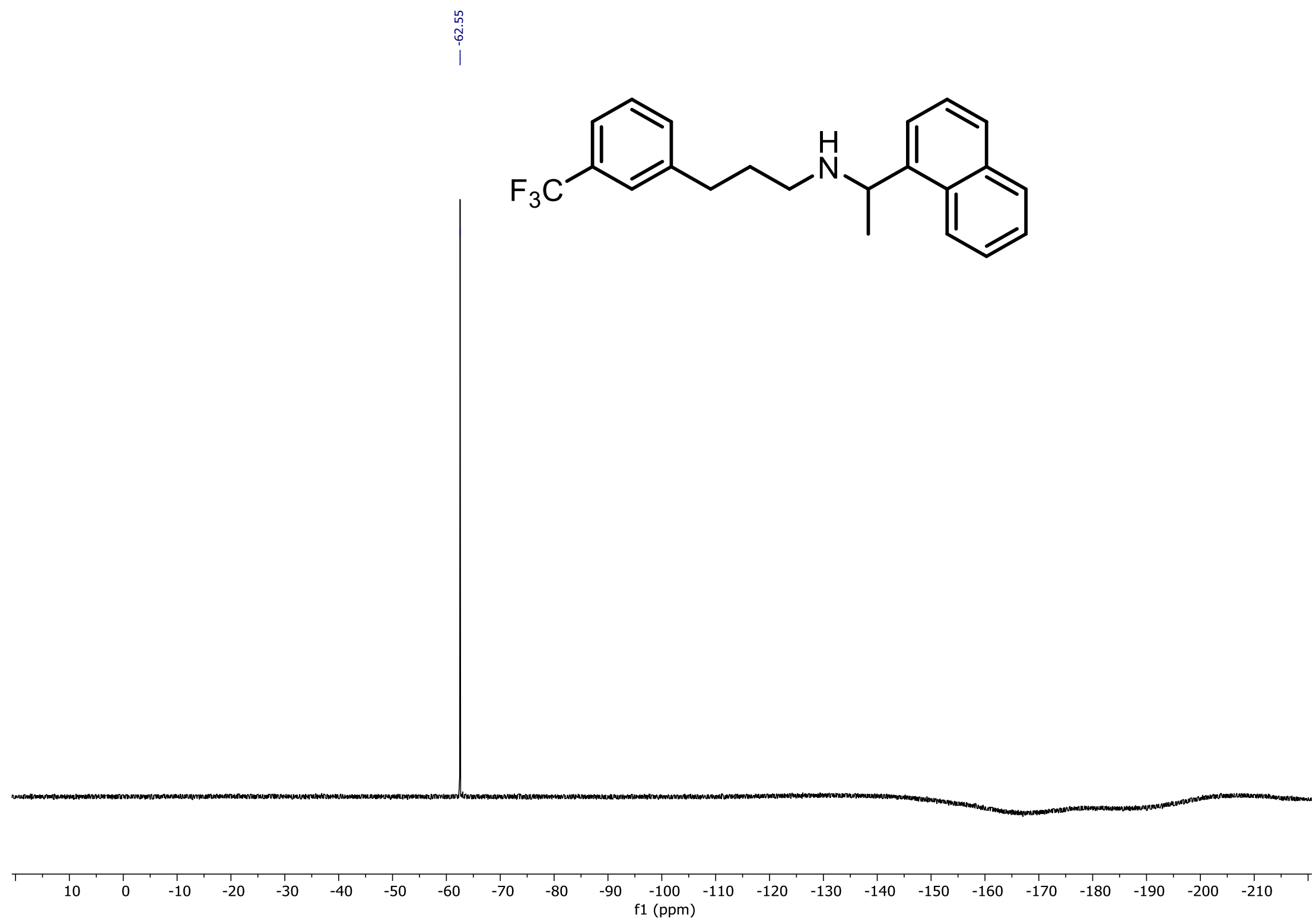

Tecalcet (2.02)
^{1}H NMR (600 MHz, $CDCl_3$)

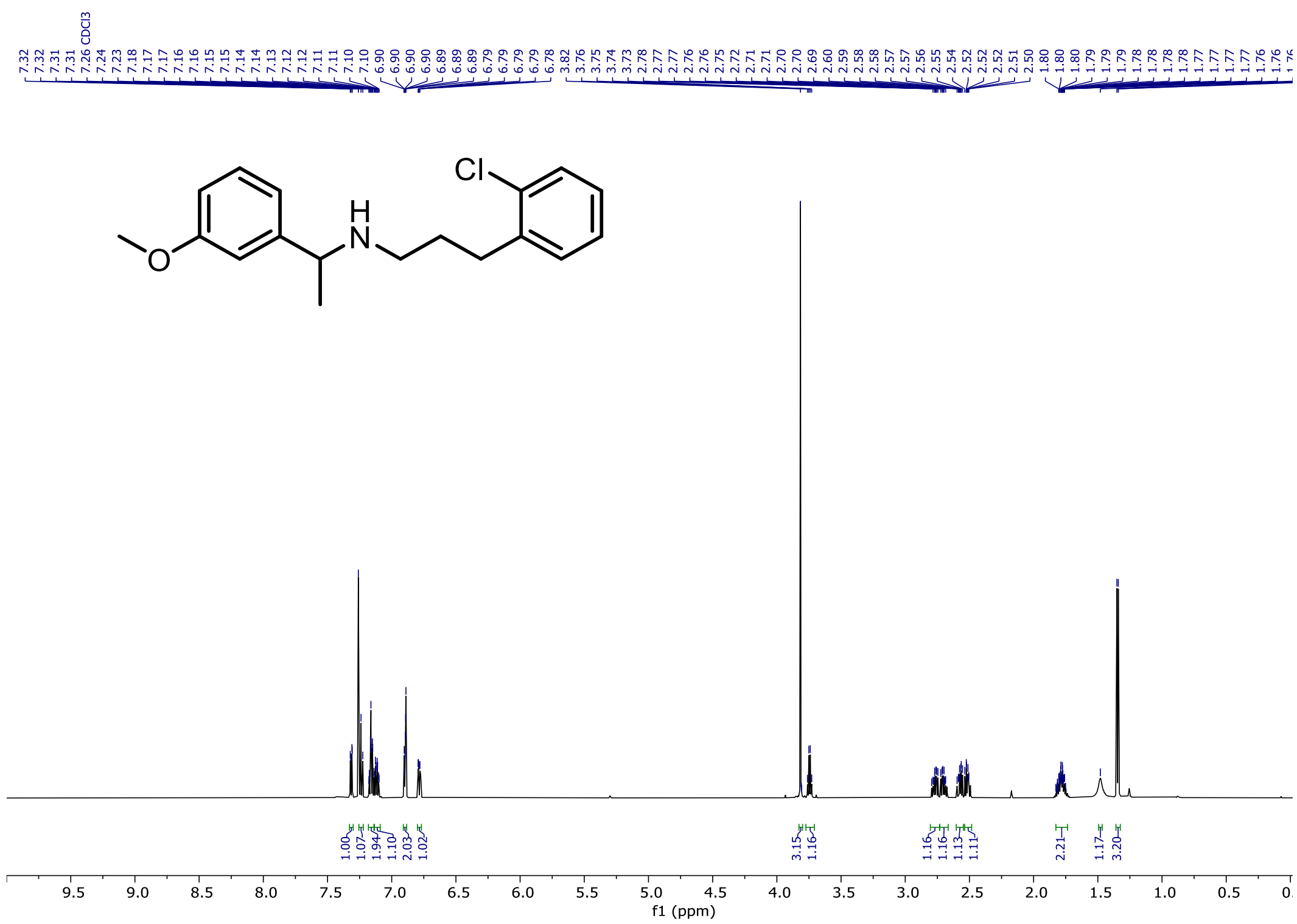

^{13}C NMR (151 MHz, $CDCl_3$)

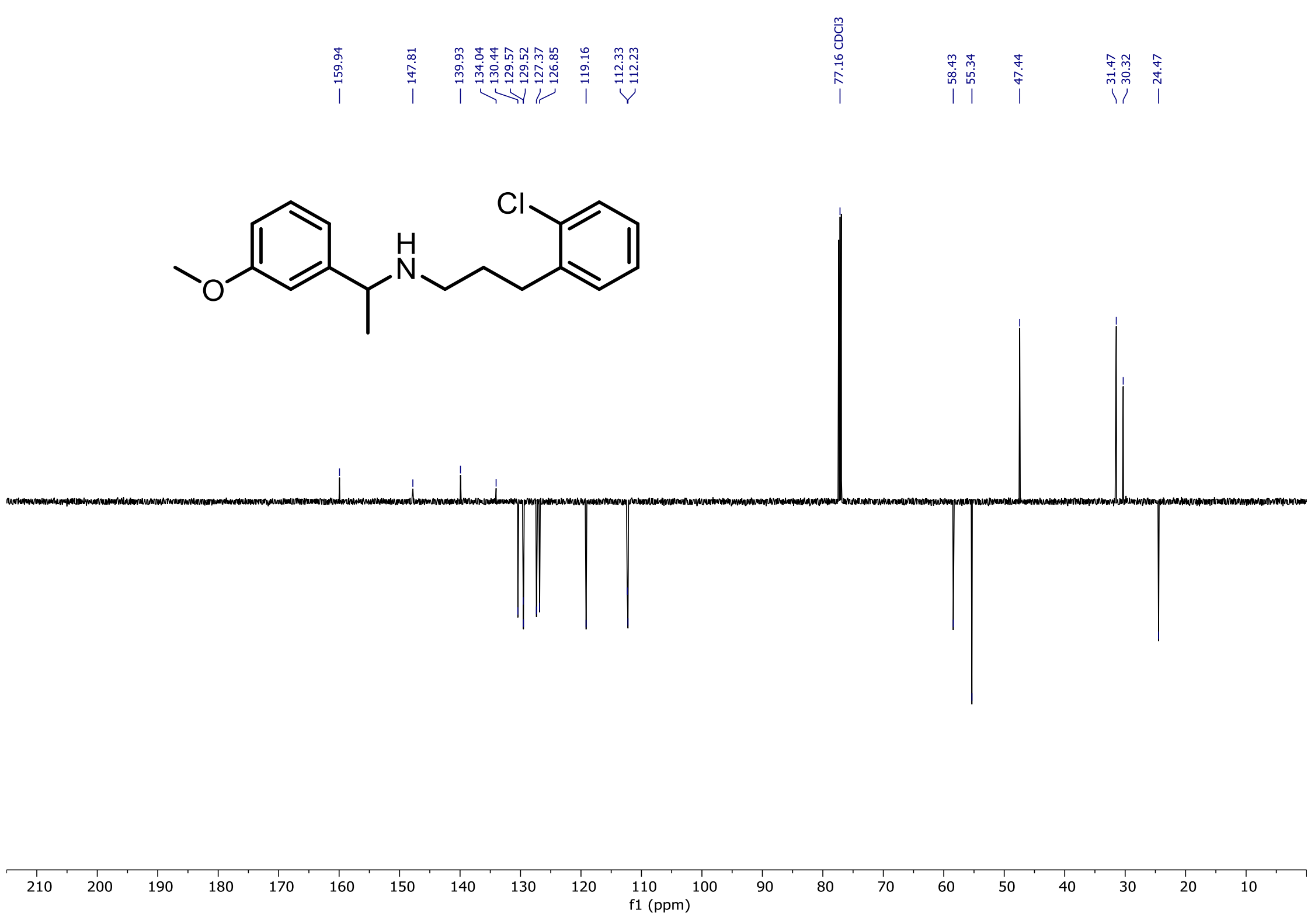

Naftifine (2.03)

^{1}H NMR (600 MHz, $CDCl_3$)

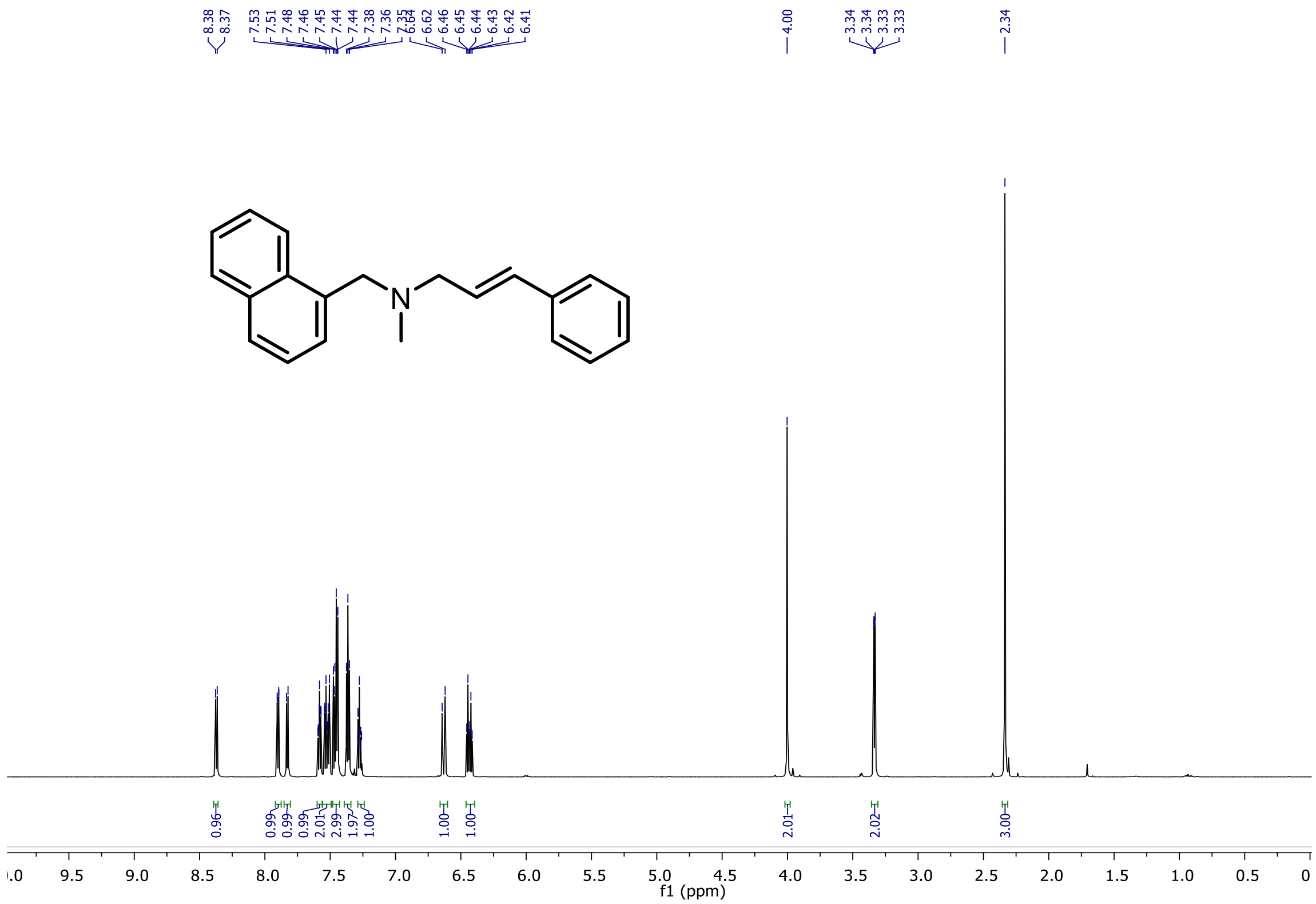

^{13}C NMR (151 MHz, $CDCl_3$)

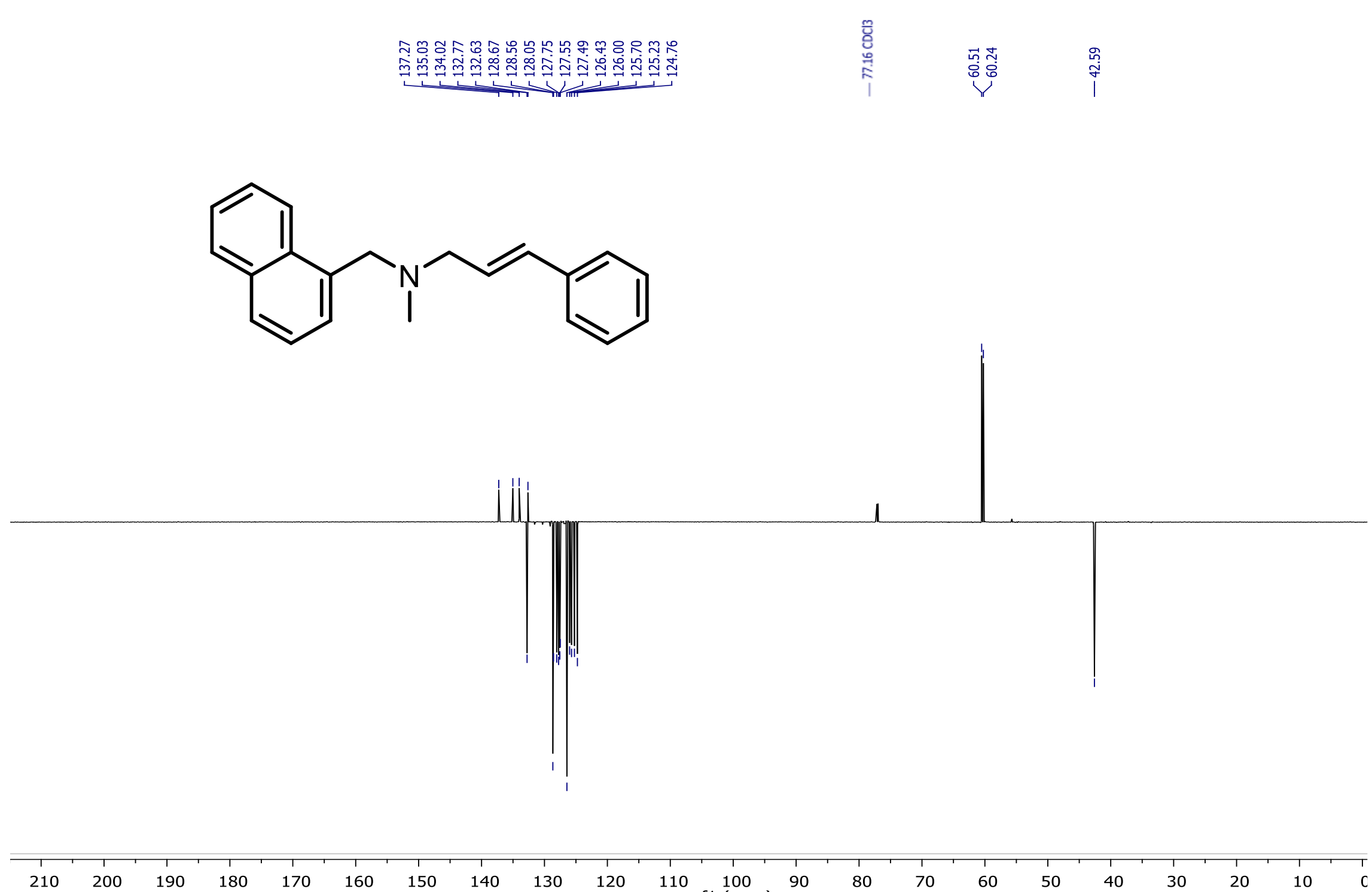

Fendiline (2.04)

^{1}H NMR (400 MHz, CDCl$_3$)

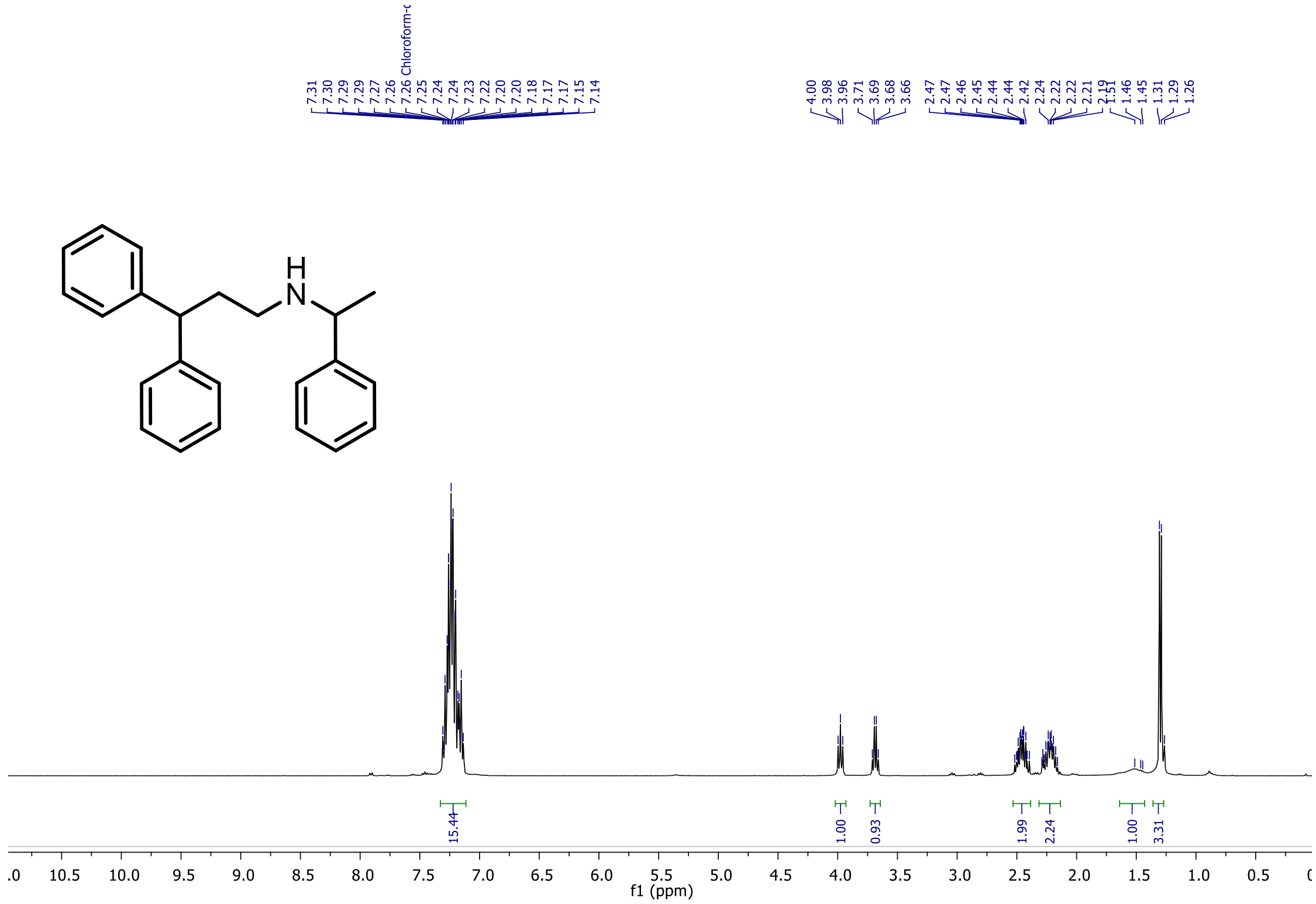

^{13}C NMR (101 MHz, CDCl$_3$)

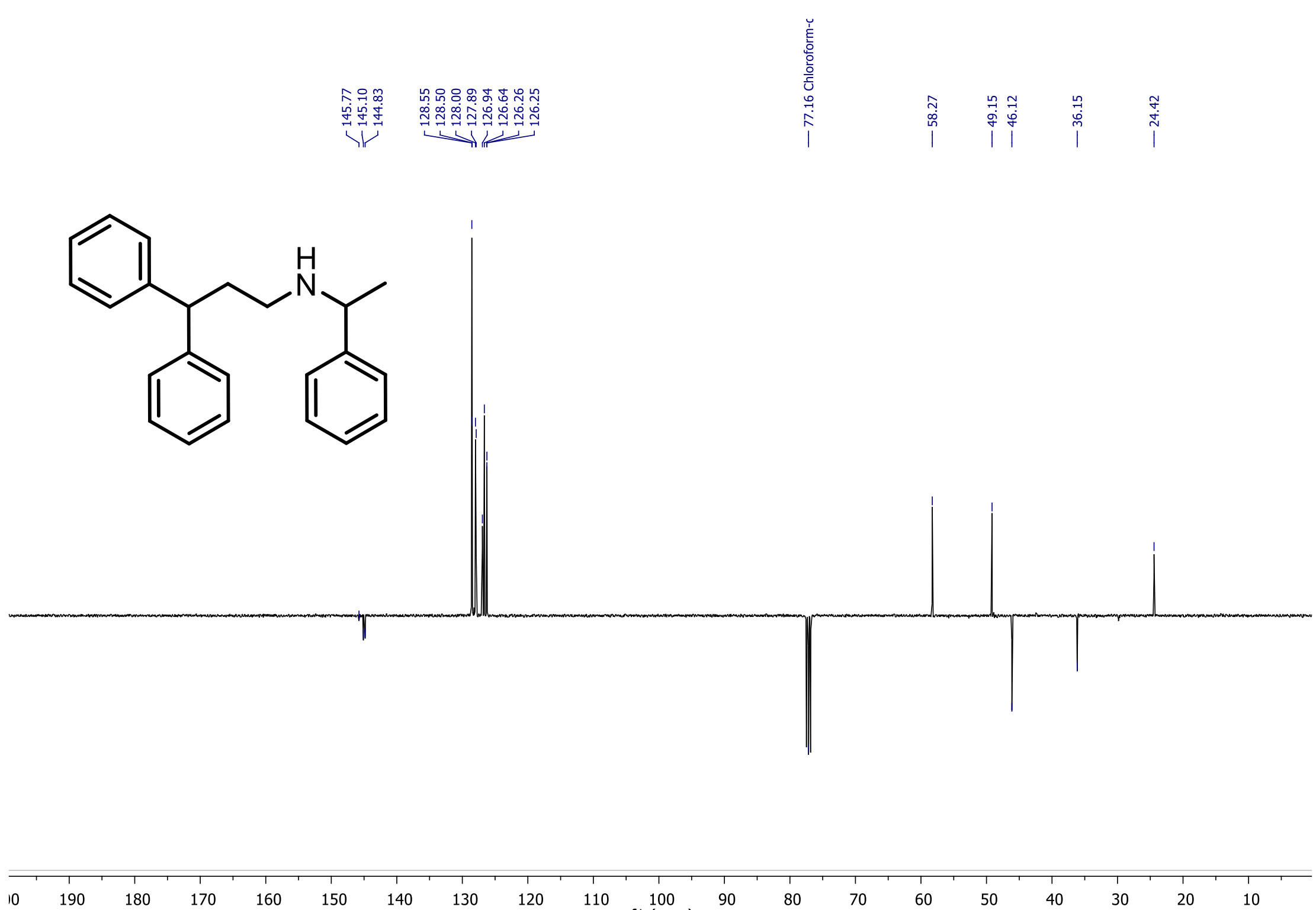

_N_-Methyl-_N_-(2-((3-phenylpropyl)amino)ethyl)isoquinoline-5-sulfonamide (3.01)

^{1}H NMR (400 MHz, CDCl$_3$)

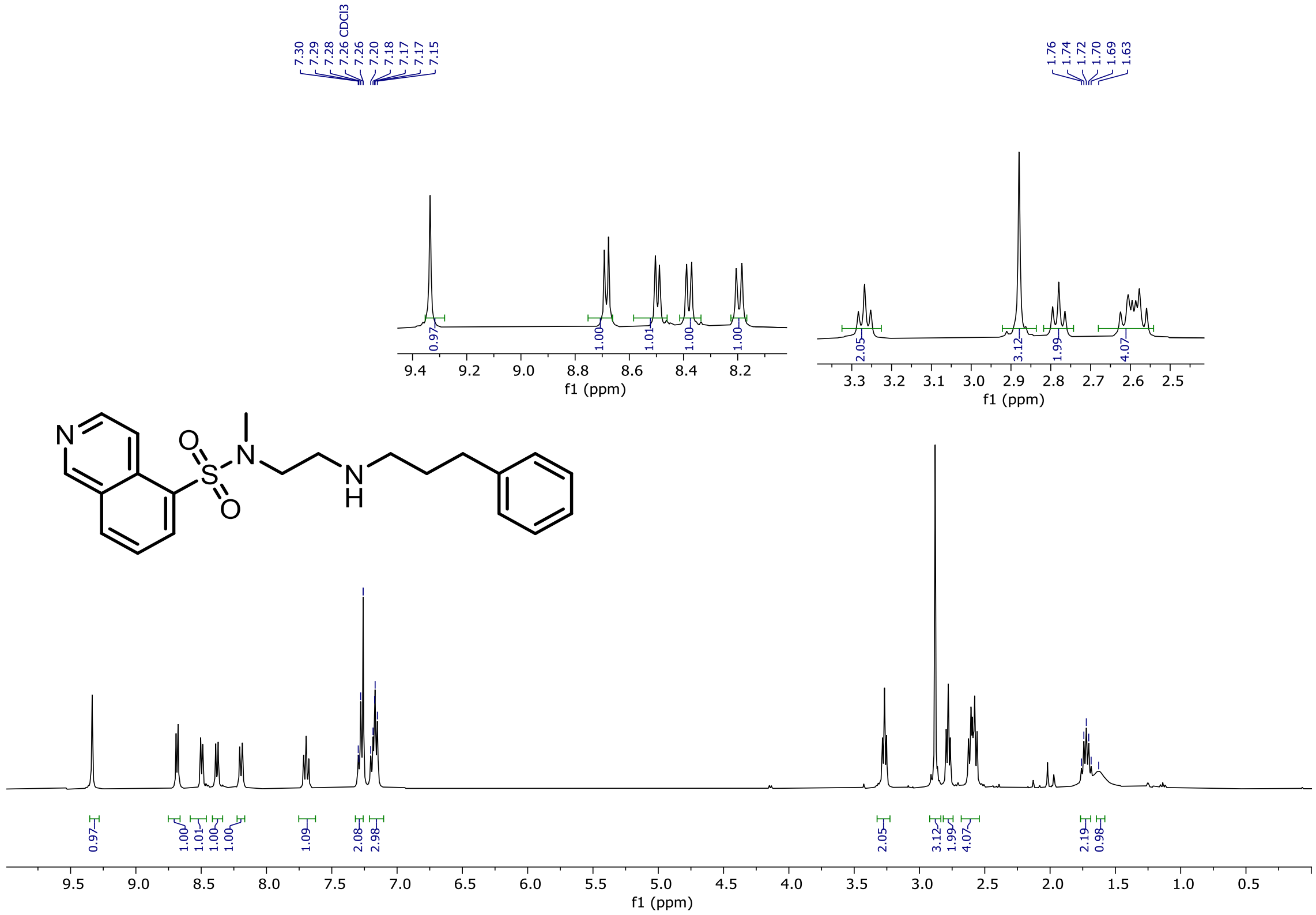

^{13}C NMR (101 MHz, CDCl$_3$)

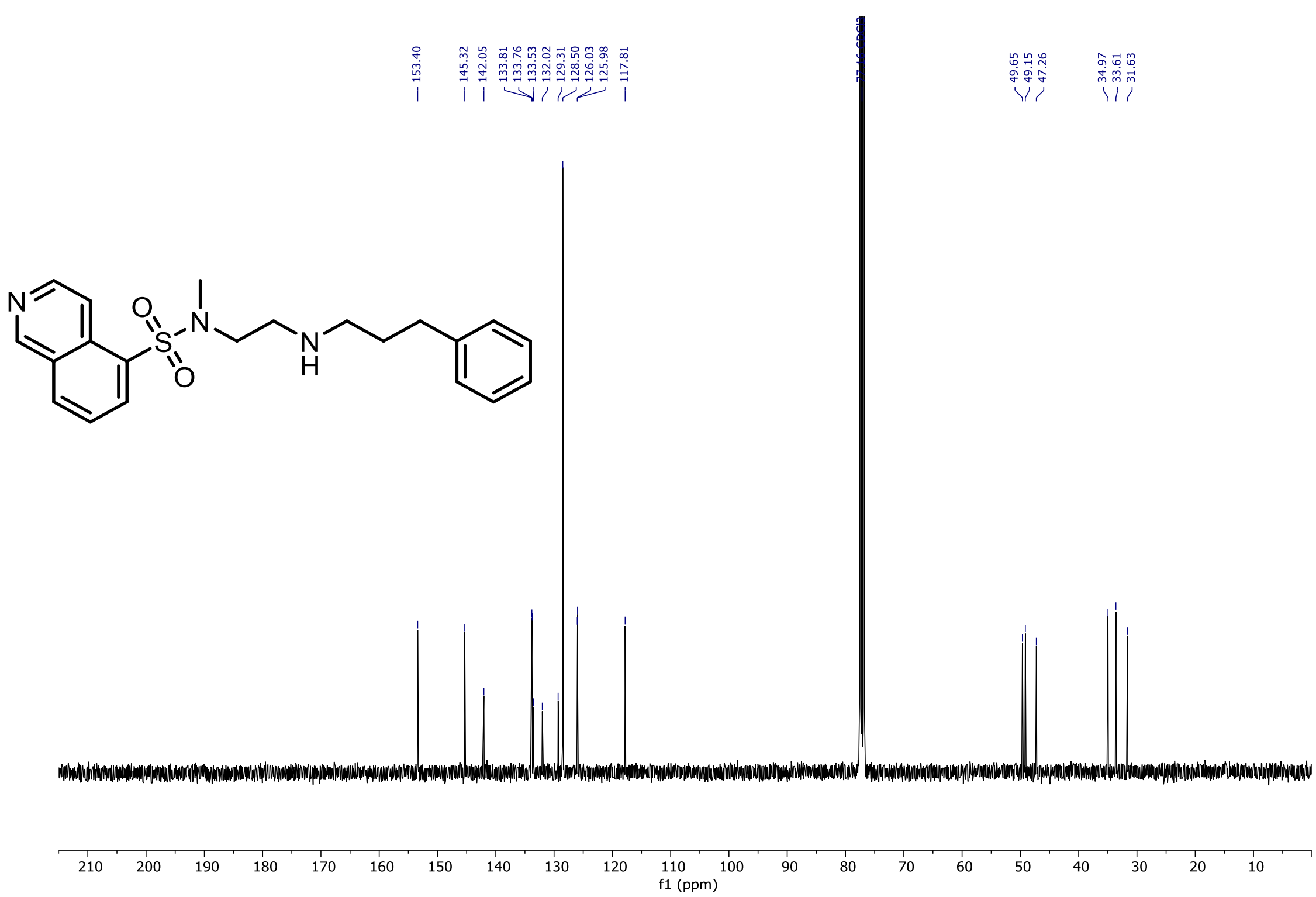

_N_-(2-((3-(4-Fluorophenyl)propyl)amino)ethyl)-_N_-methylisoquinoline-5-sulfonamide (3.02)
^{1}H NMR (400 MHz, $CDCl_3$)

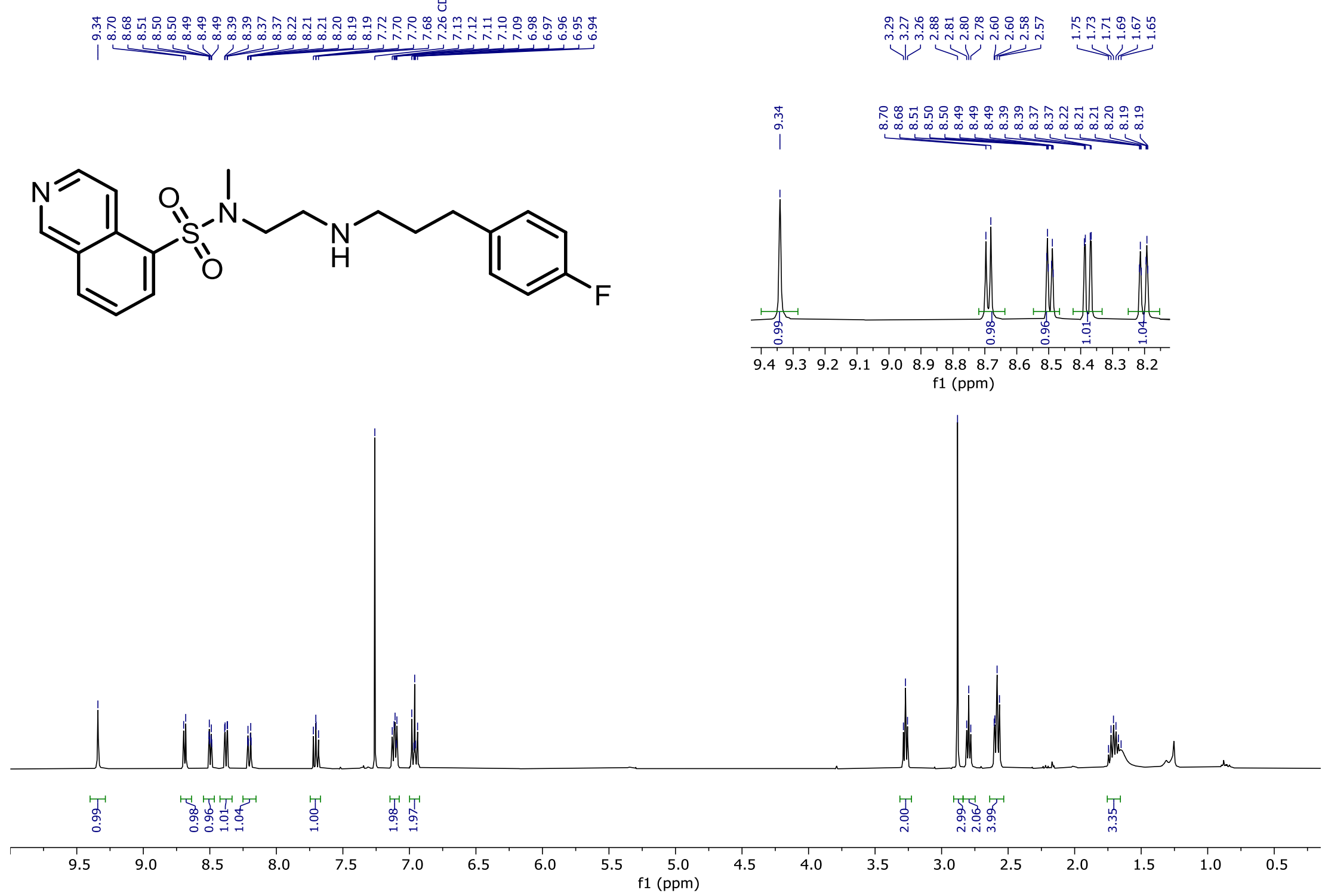

^{13}C NMR (101 MHz, $CDCl_3$)

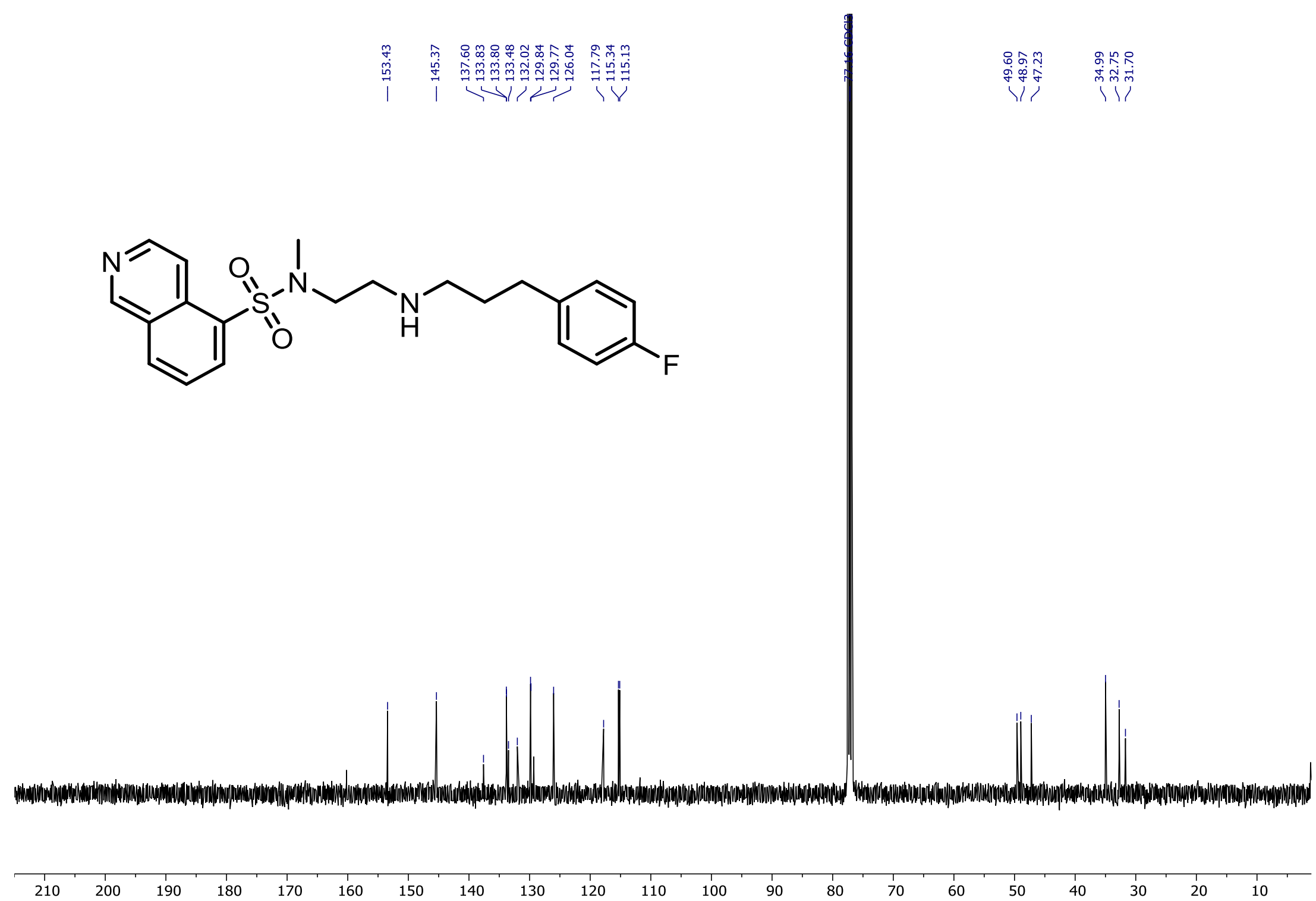

^{19}F NMR (376 MHz, $CDCl_3$)

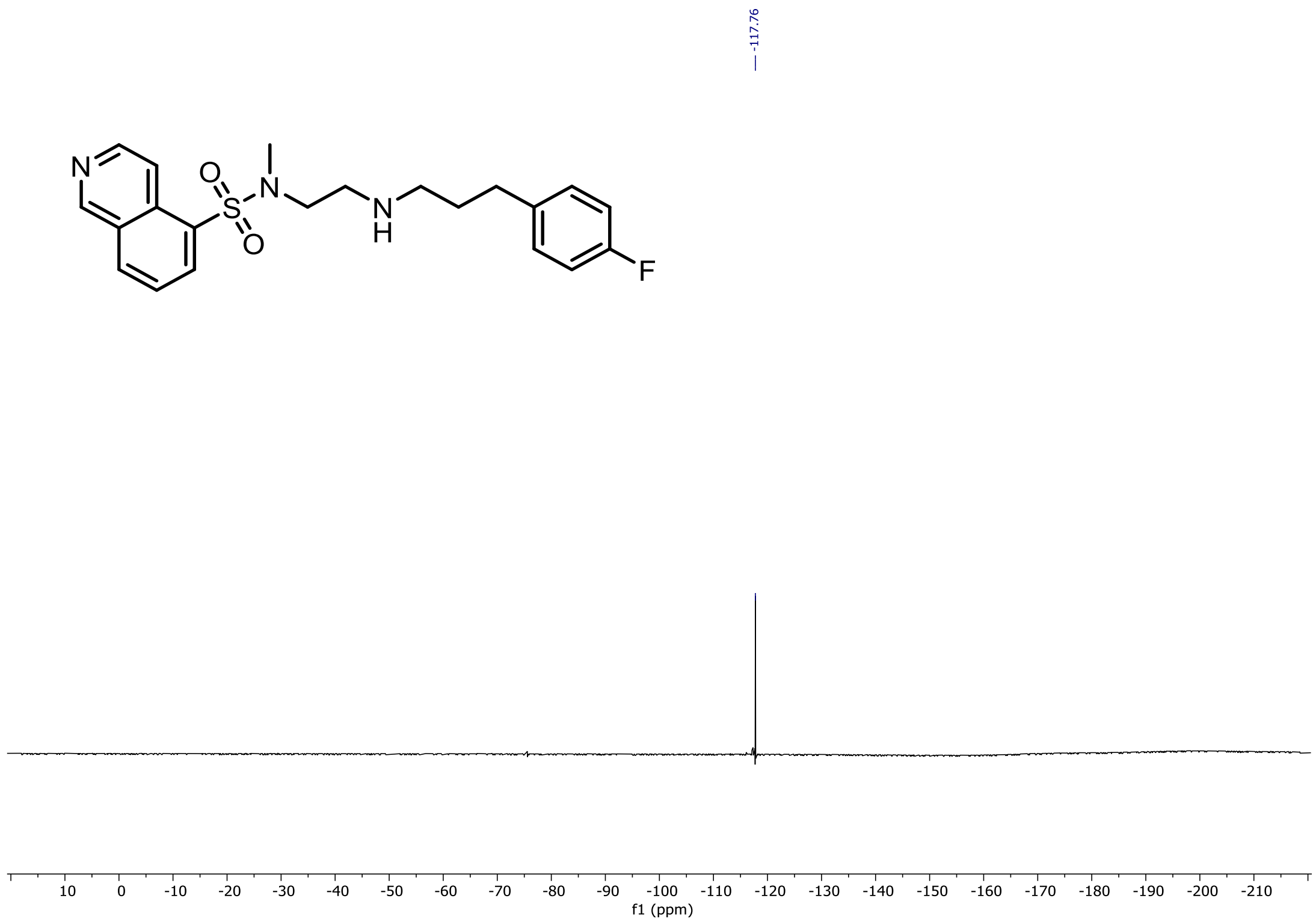

***N*-(2-((3-(4-Bromophenyl)propyl)amino)ethyl)-*N*-methylisoquinoline-5-sulfonamide (3.03)**

^{1}H NMR (400 MHz, $CDCl_3$)

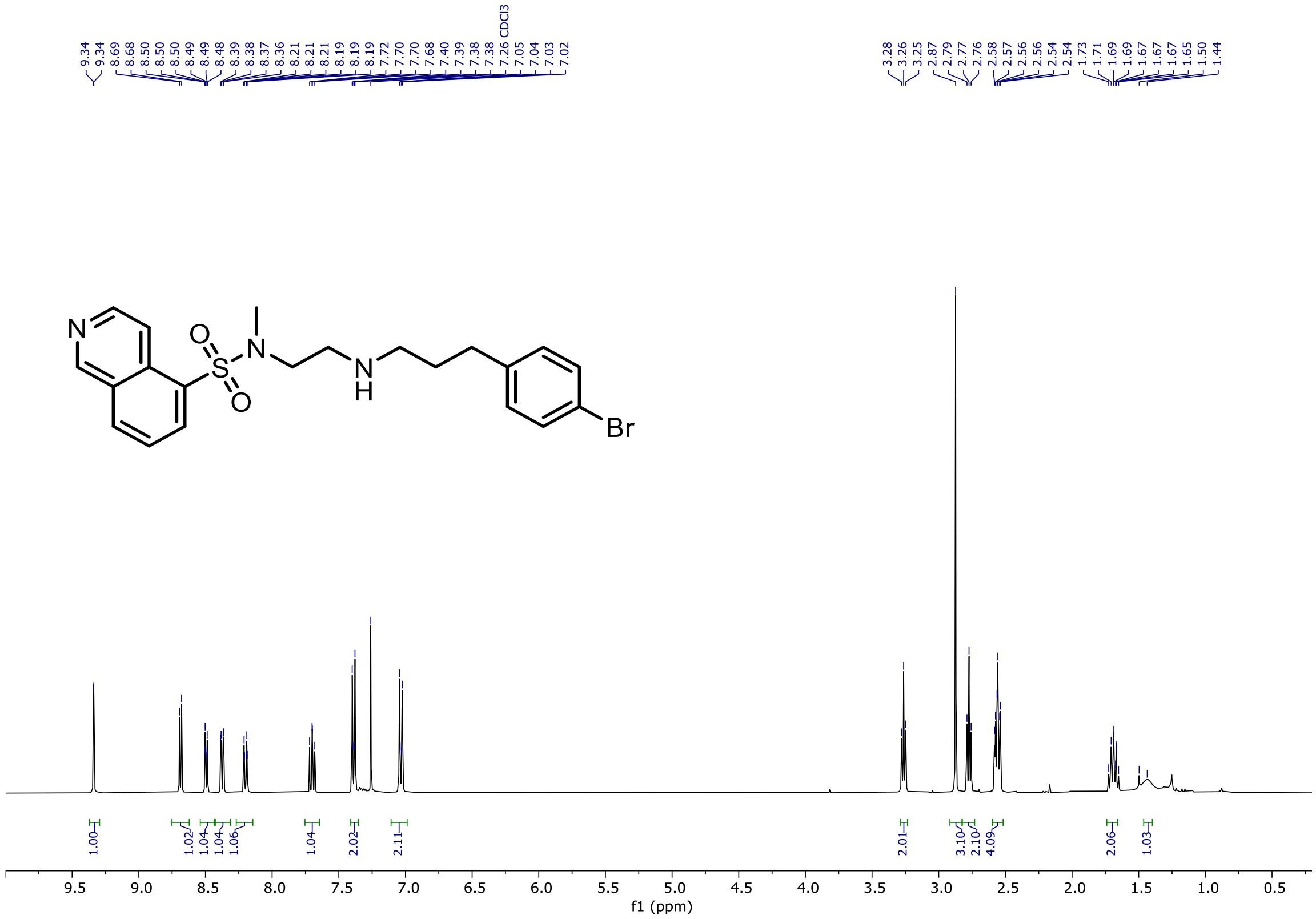

^{13}C NMR (101 MHz, $CDCl_3$)

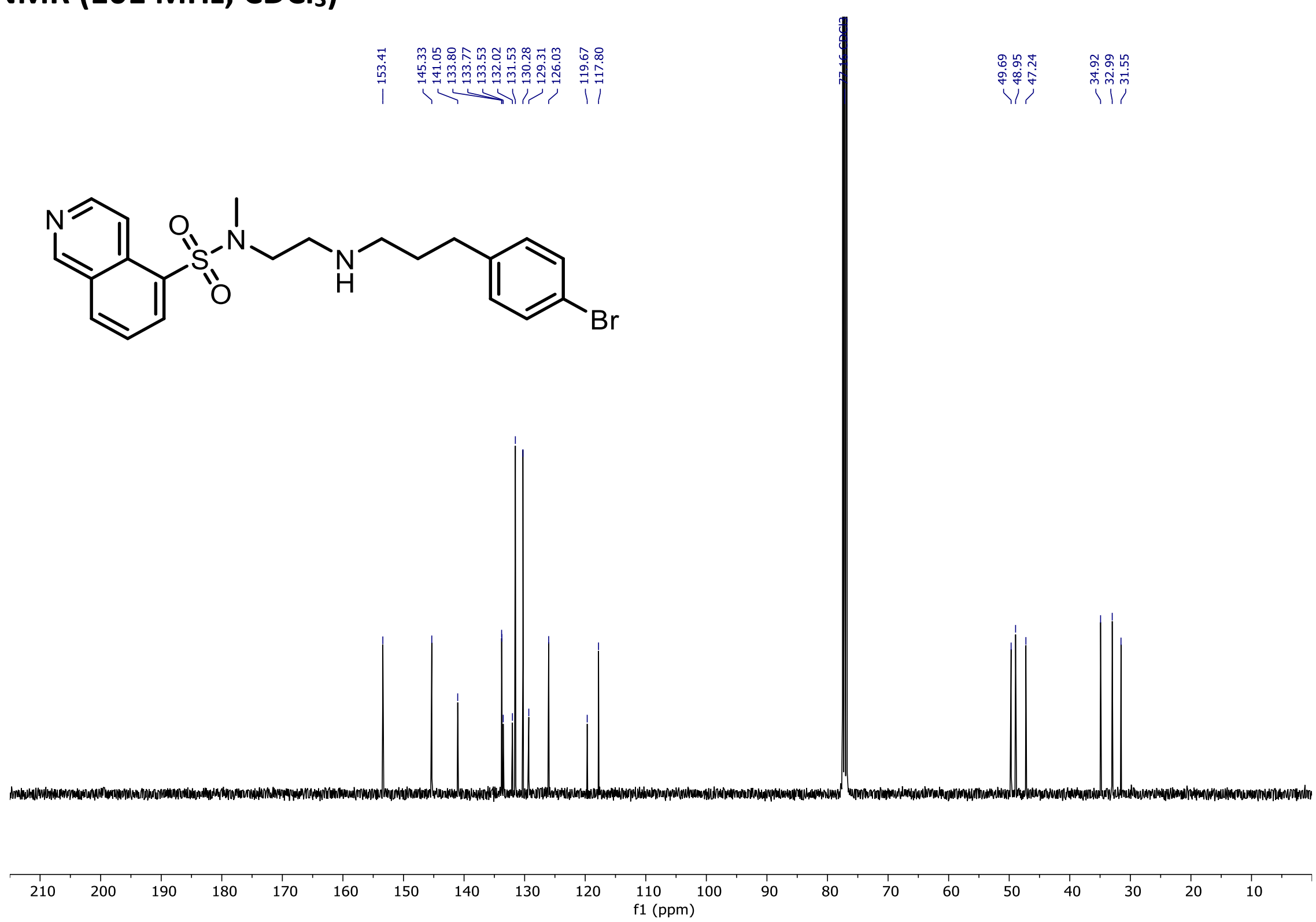

(*E*)-*N*-Methyl-*N*-(2-((3-(4-(pyrimidin-5-yl)phenyl)allyl)amino)ethyl)isoquinoline-5-sulfonamide (3.04)

^{1}H NMR (400 MHz, $CDCl_3$)

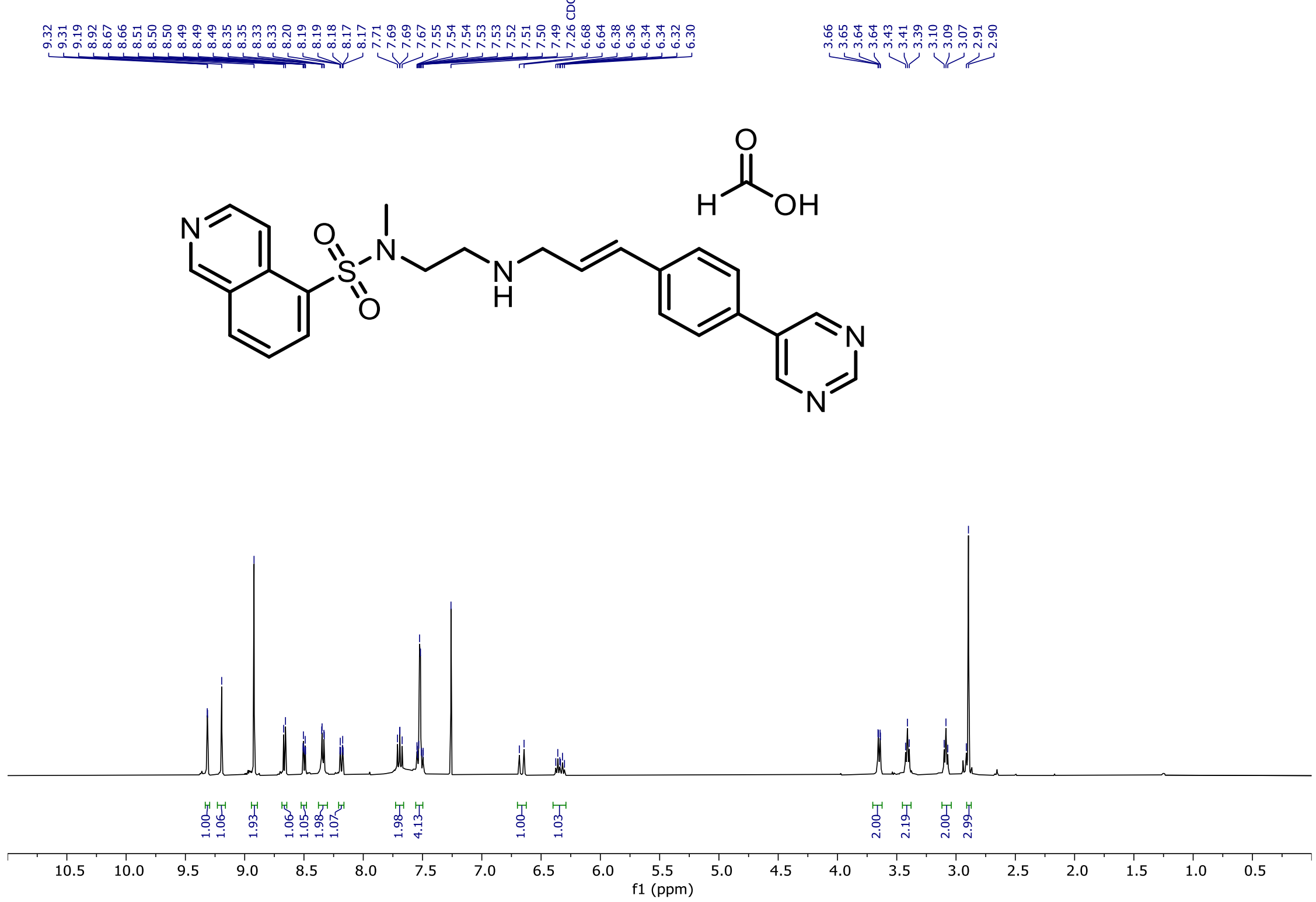

^{13}C NMR (101 MHz, $CDCl_3$)

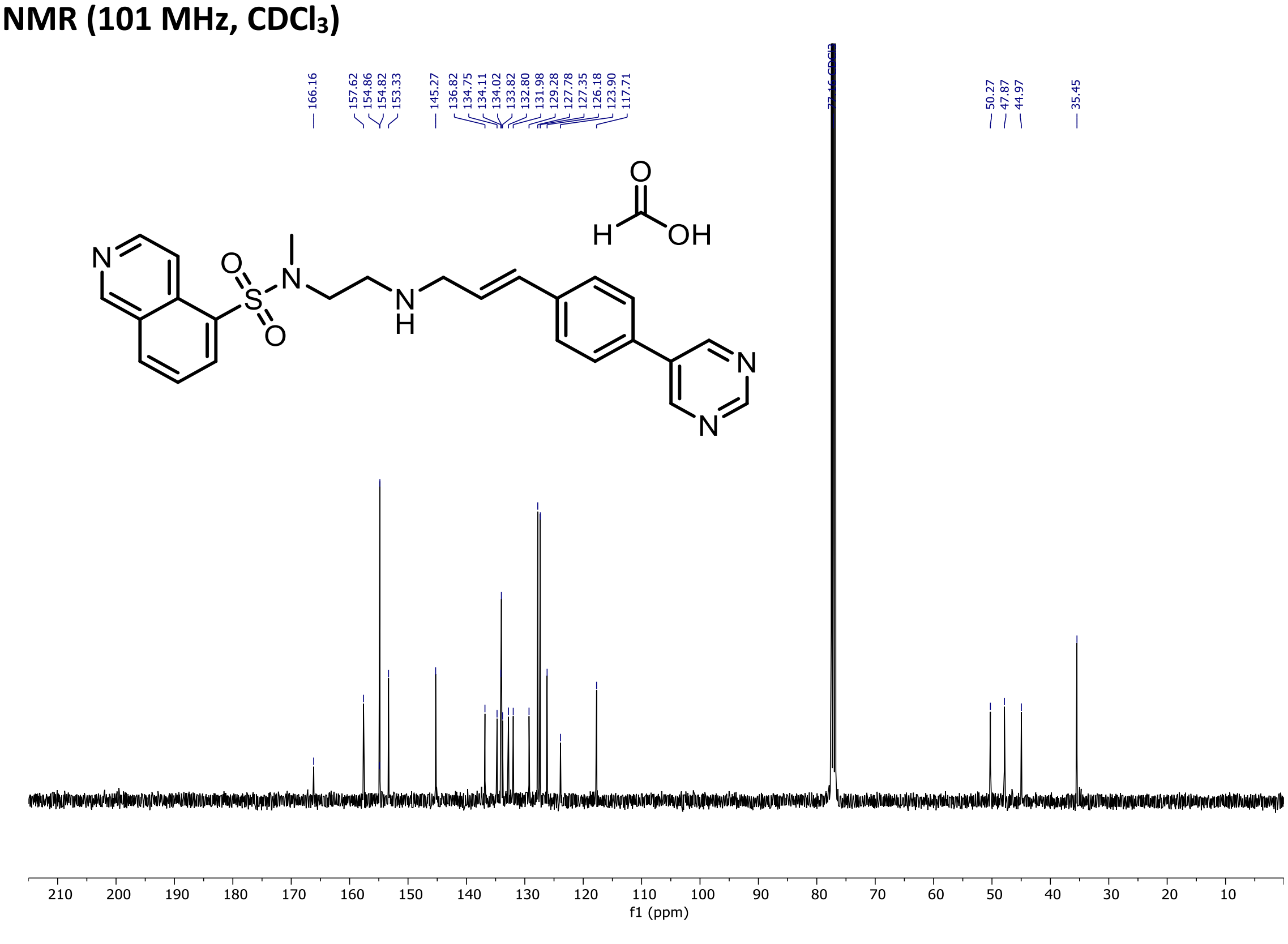

(*E*)-*N*-Methyl-*N*-(2-((3-(4-(pyridin-3-yl)phenyl)allyl)amino)ethyl)isoquinoline-5-sulfonamide (3.05)

^{1}H NMR (400 MHz, $CDCl_3$)

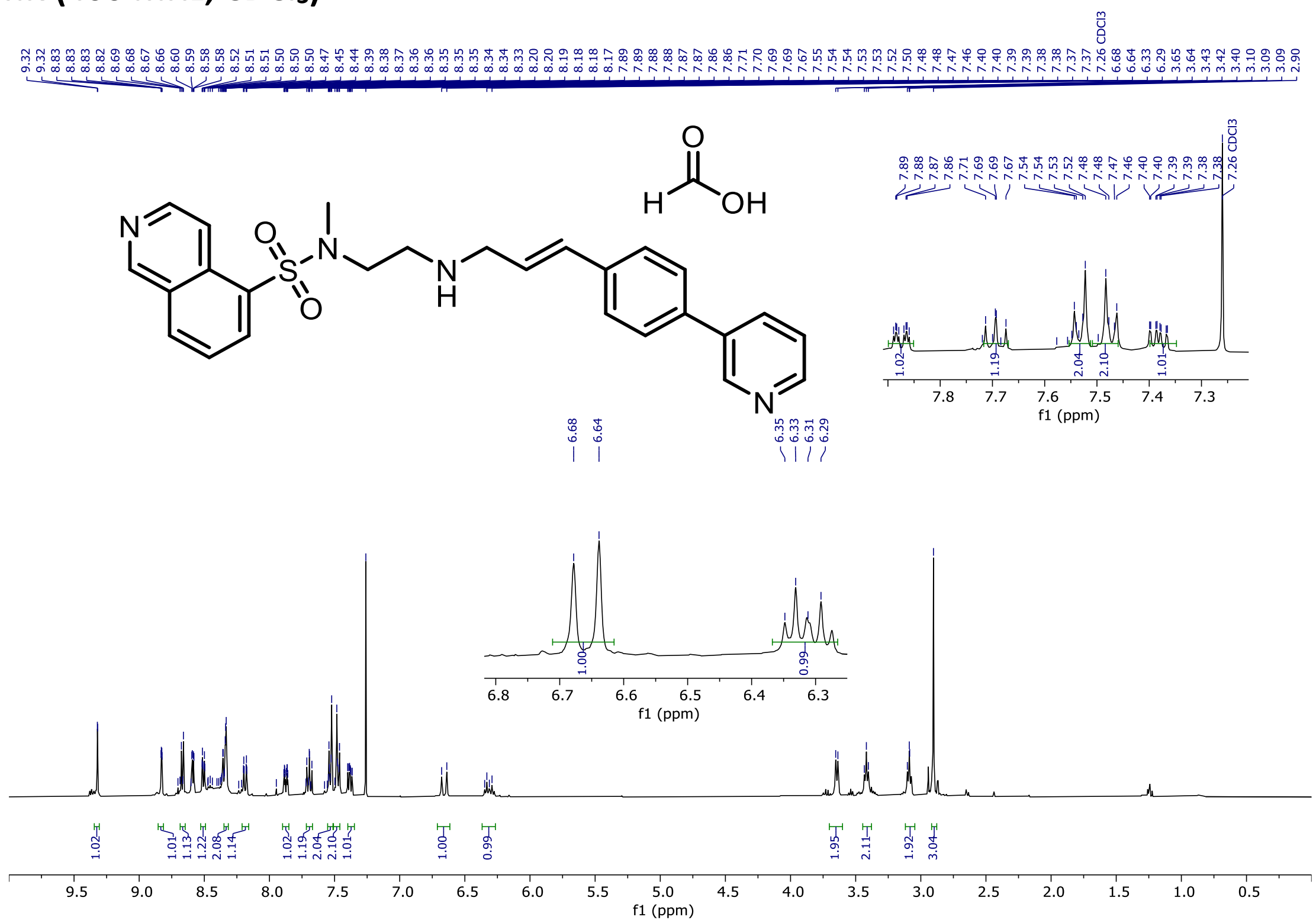

^{13}C NMR (101 MHz, $CDCl_3$)

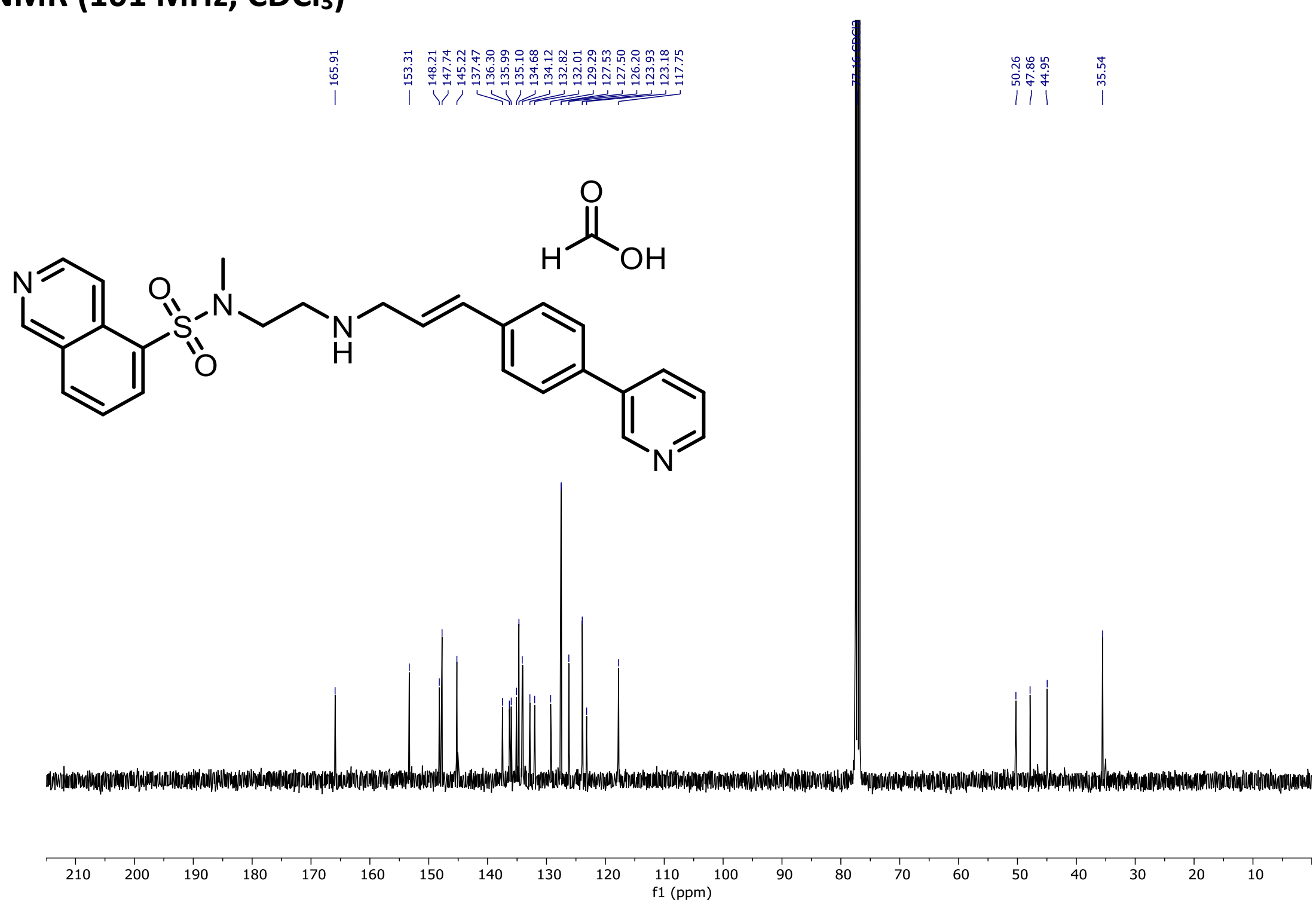

1-((*S*)-3,3-Dimethyl-2-(12-(phenethylamino)dodecanamido)butanoyl)-4-hydroxy-*N*-(4-(4-methylthiazol-5-yl)benzyl)pyrrolidine-2-carboxamide (4.01)

^{1}H NMR (600 MHz, MeOD)

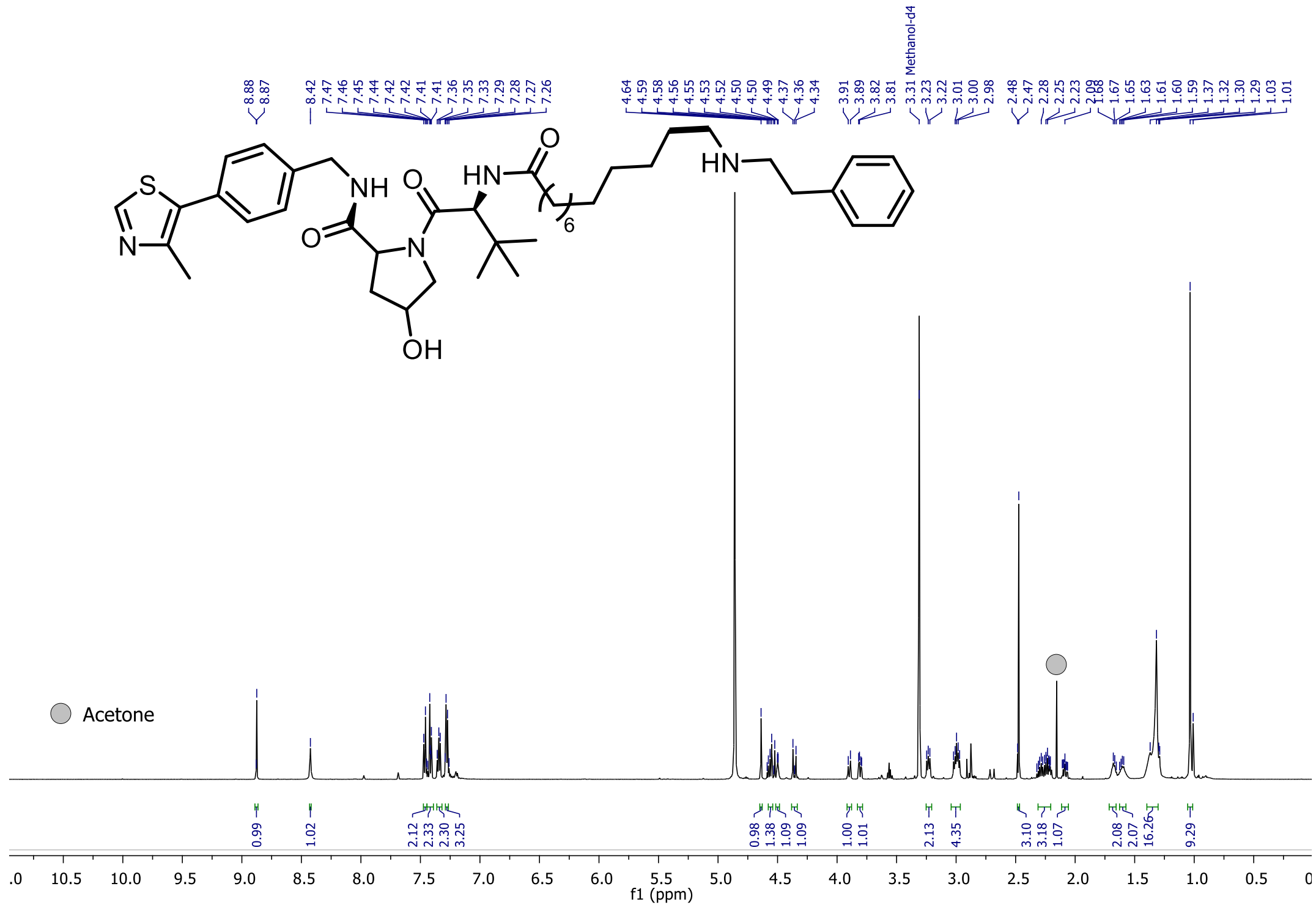

^{13}C NMR (151 MHz, MeOD)

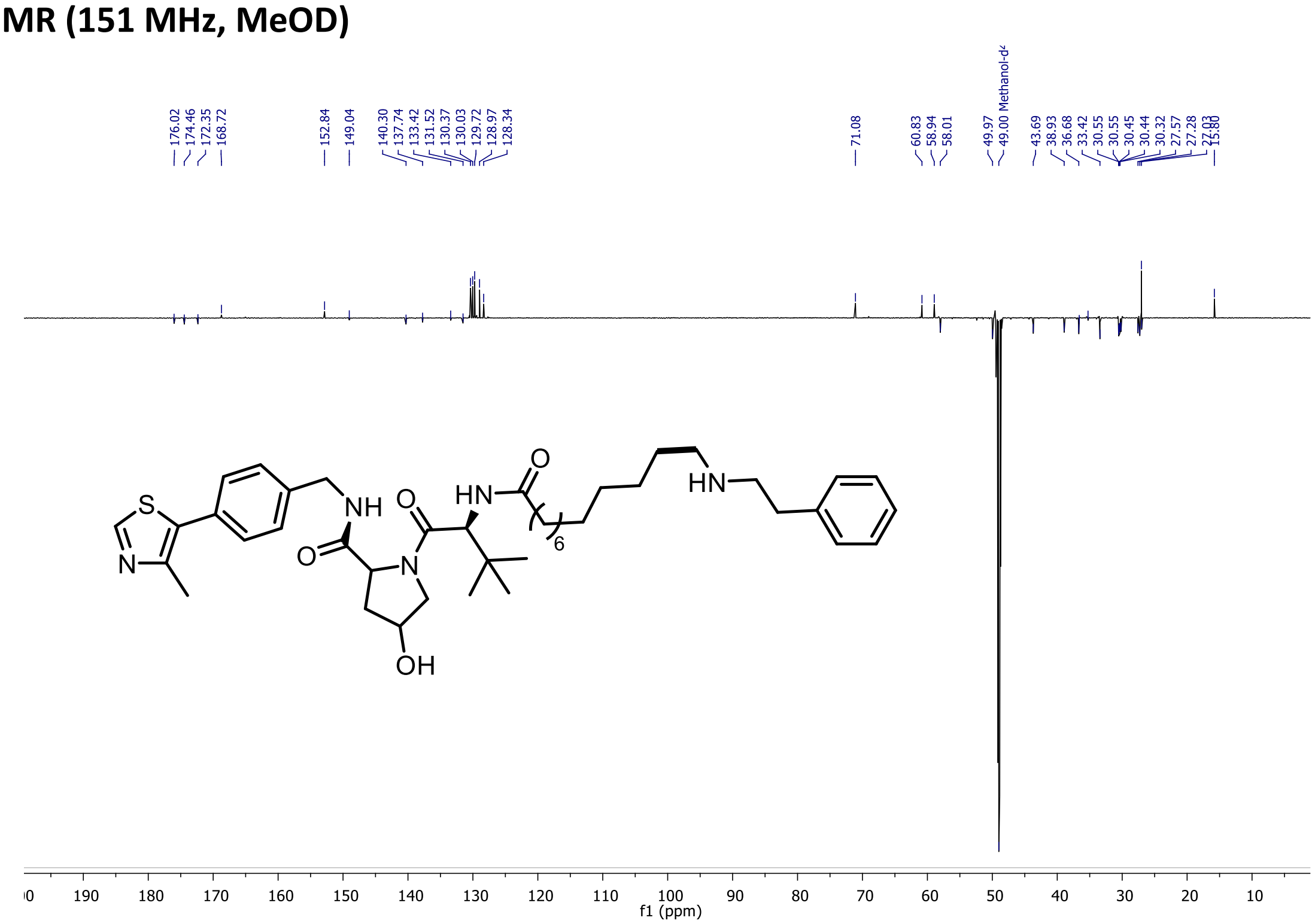

12-((3-((1-Benzyl-1*H*-indazol-3-yl)oxy)propyl)amino)-*N*-(2-(2,6-dioxopiperidin-3-yl)-1,3-dioxoisoindolin-5-yl)dodecanamide (4.02)

^{1}H NMR (400 MHz, $CDCl_3$)

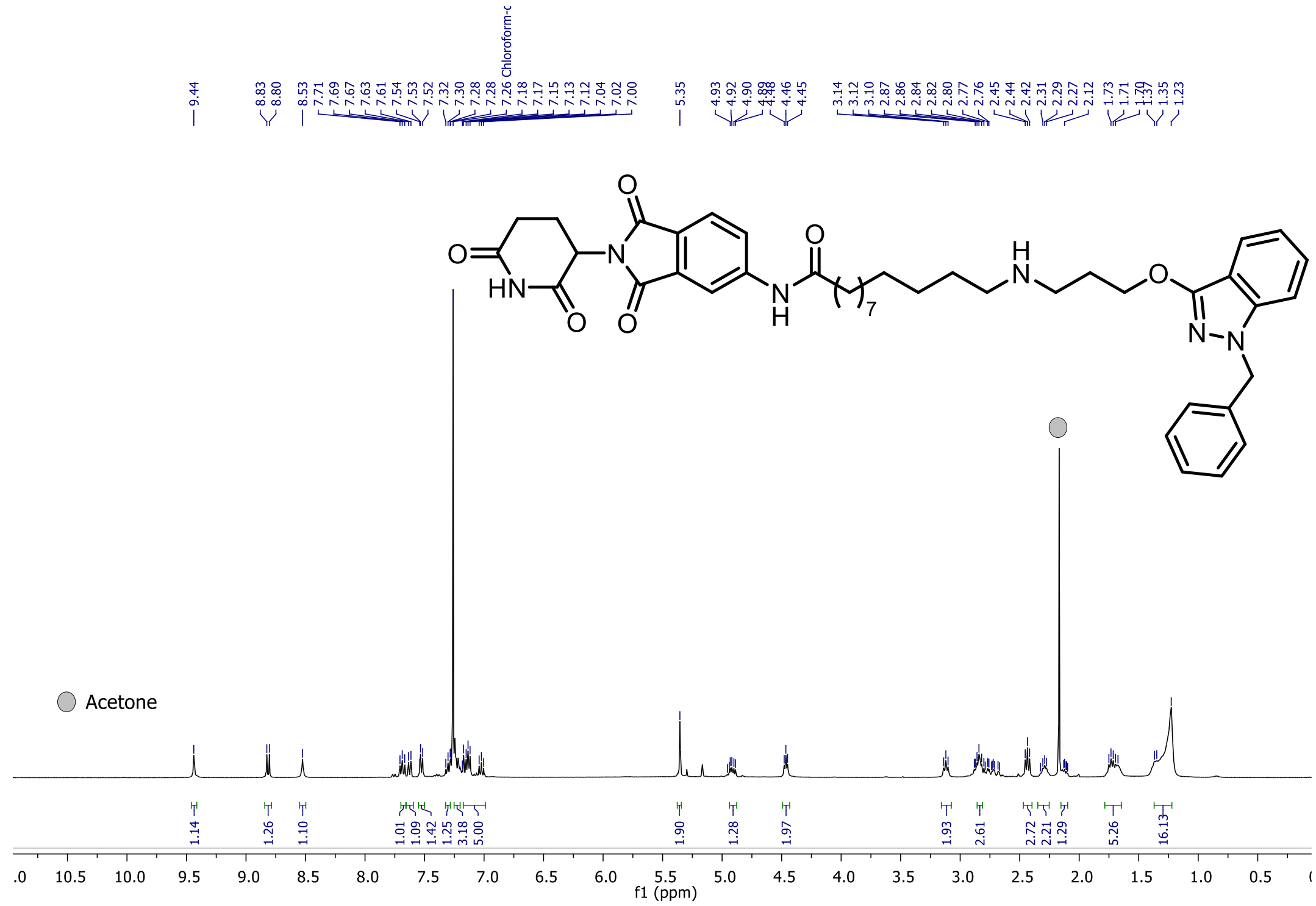

^{13}C NMR (101 MHz, $CDCl_3$)

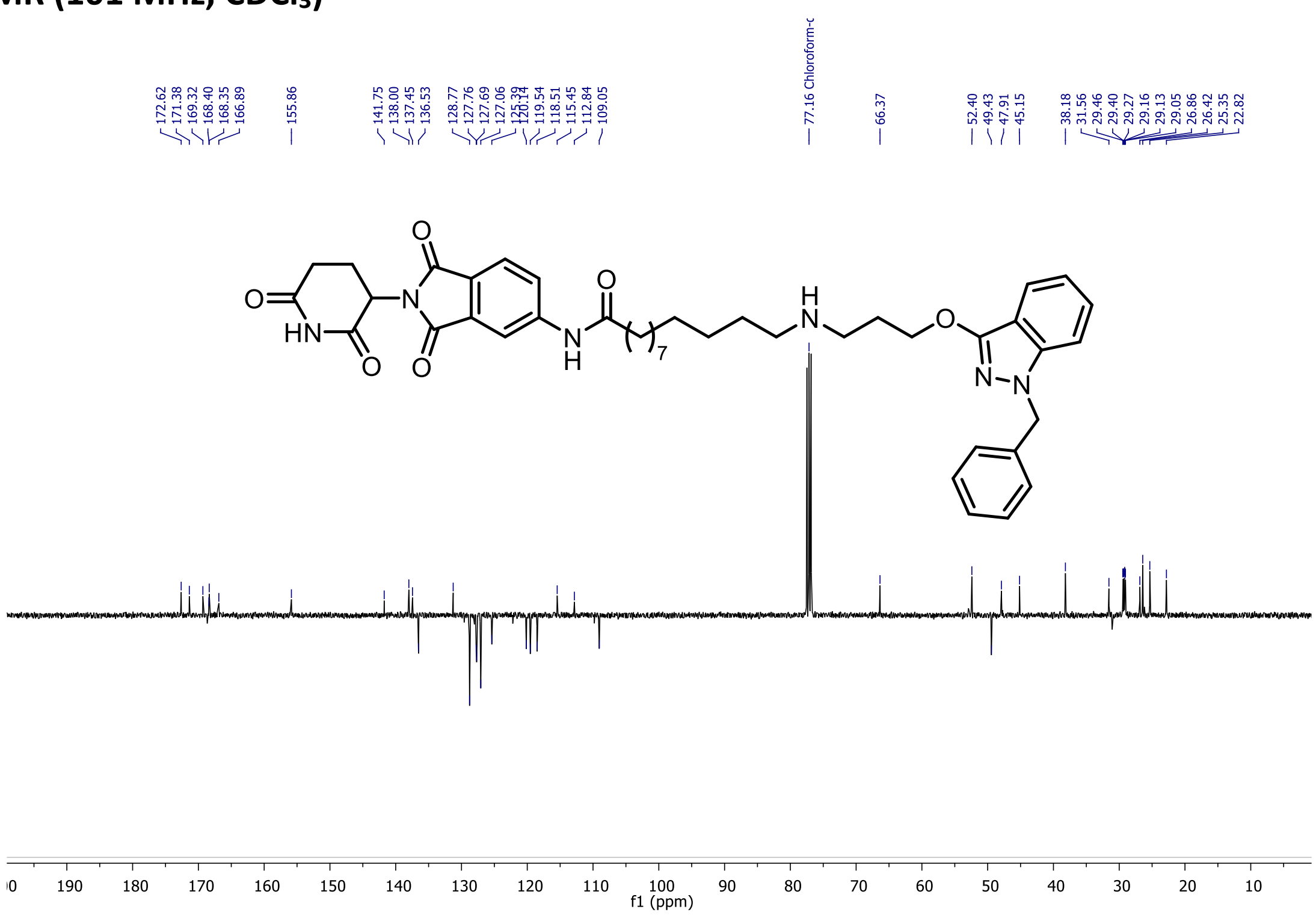

11-((3-(9,10-Ethanoanthracen-9(10*H*)-yl)propyl)amino)-*N*-(2-(2,6-dioxopiperidin-3-yl)-1,3-dioxoisoindolin-5-yl)undecanamide (4.03)

^{1}H NMR (400 MHz, $CDCl_3$)

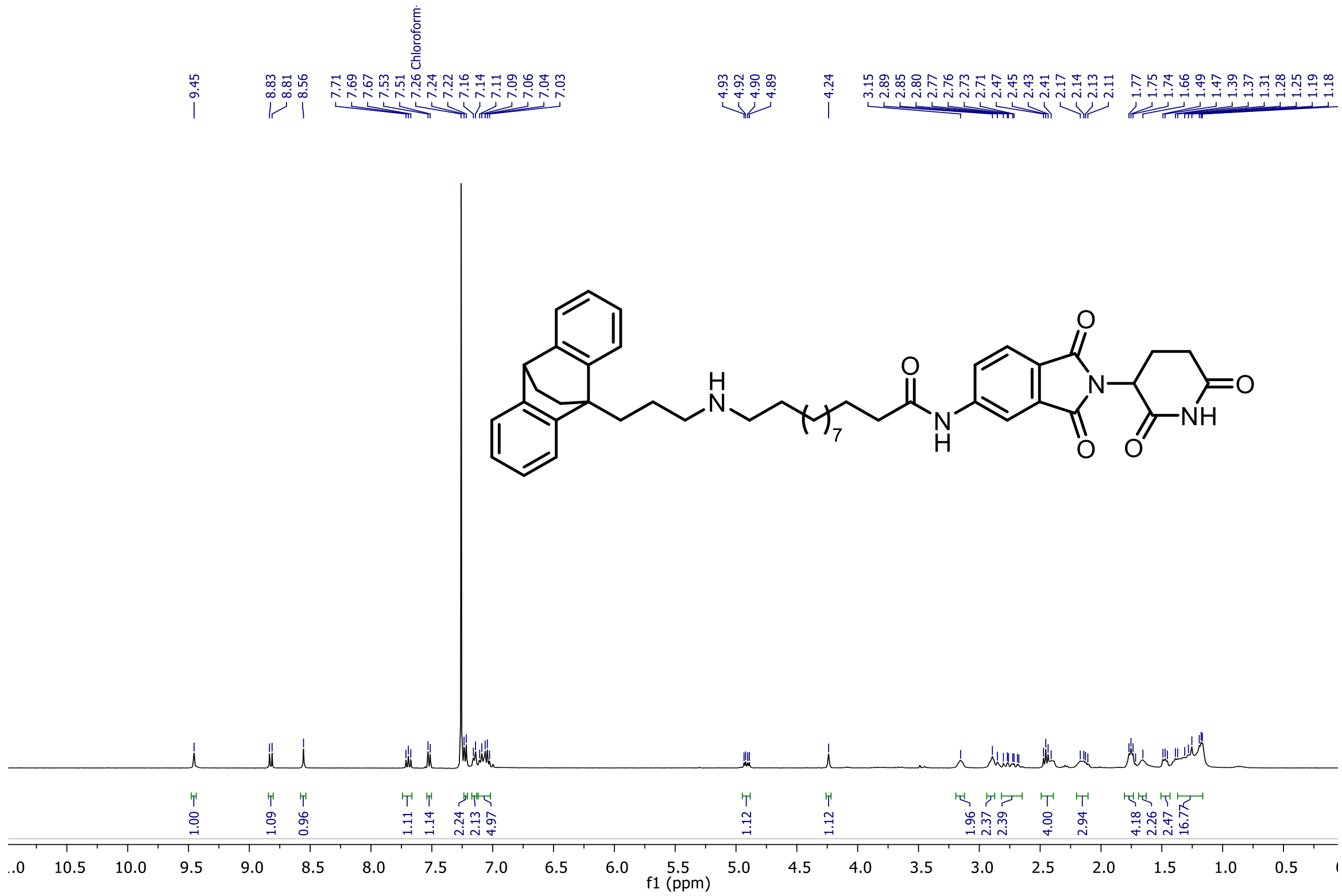

^{13}C NMR (176 MHz, $CDCl_3$)

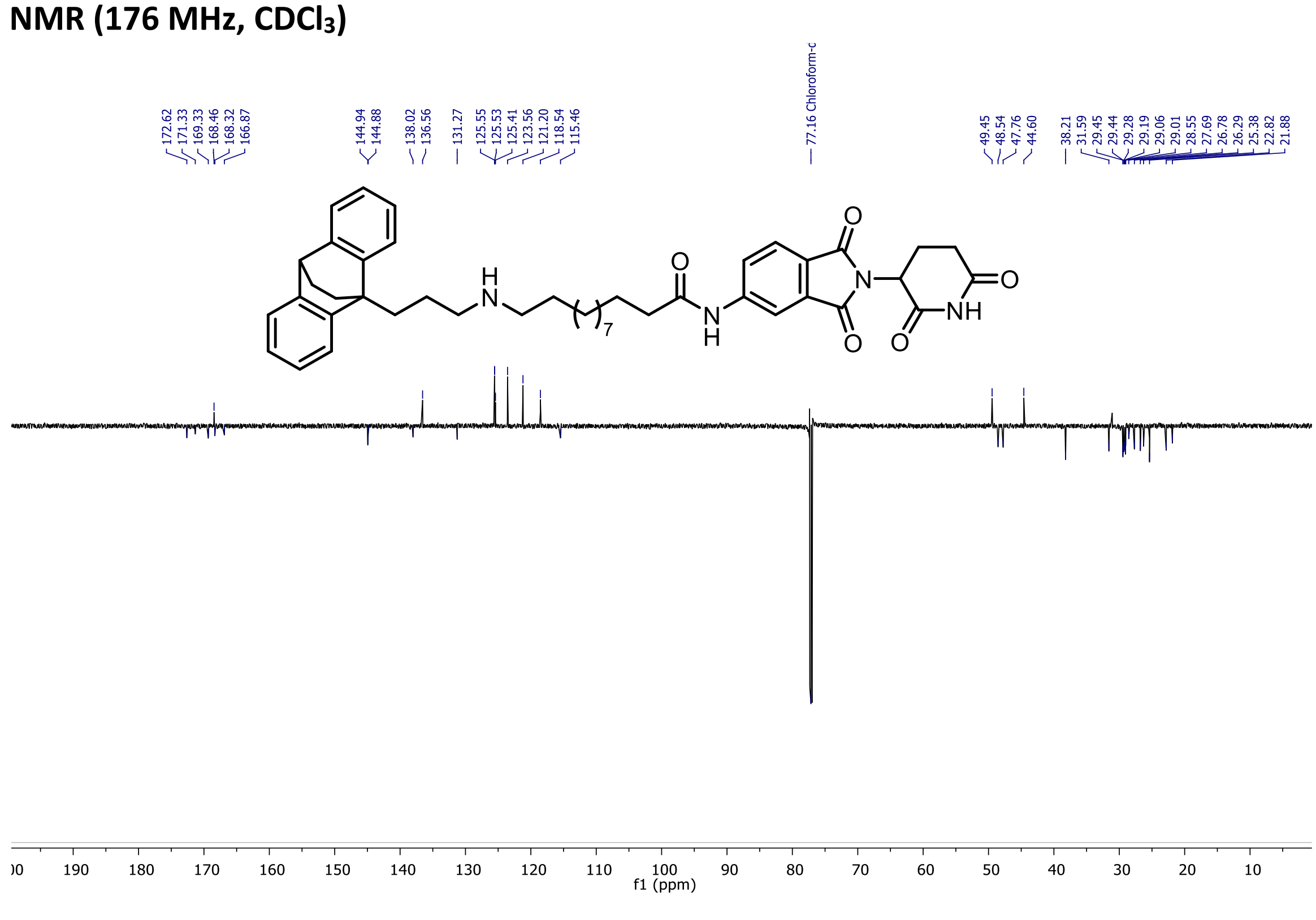

Dimethyl *N*-(5-phenylpentyl)glycylprolylphenylalanyl-1-tosyltryptophylglutamate (4.04)
^{1}H NMR (400 MHz, $CDCl_3$)

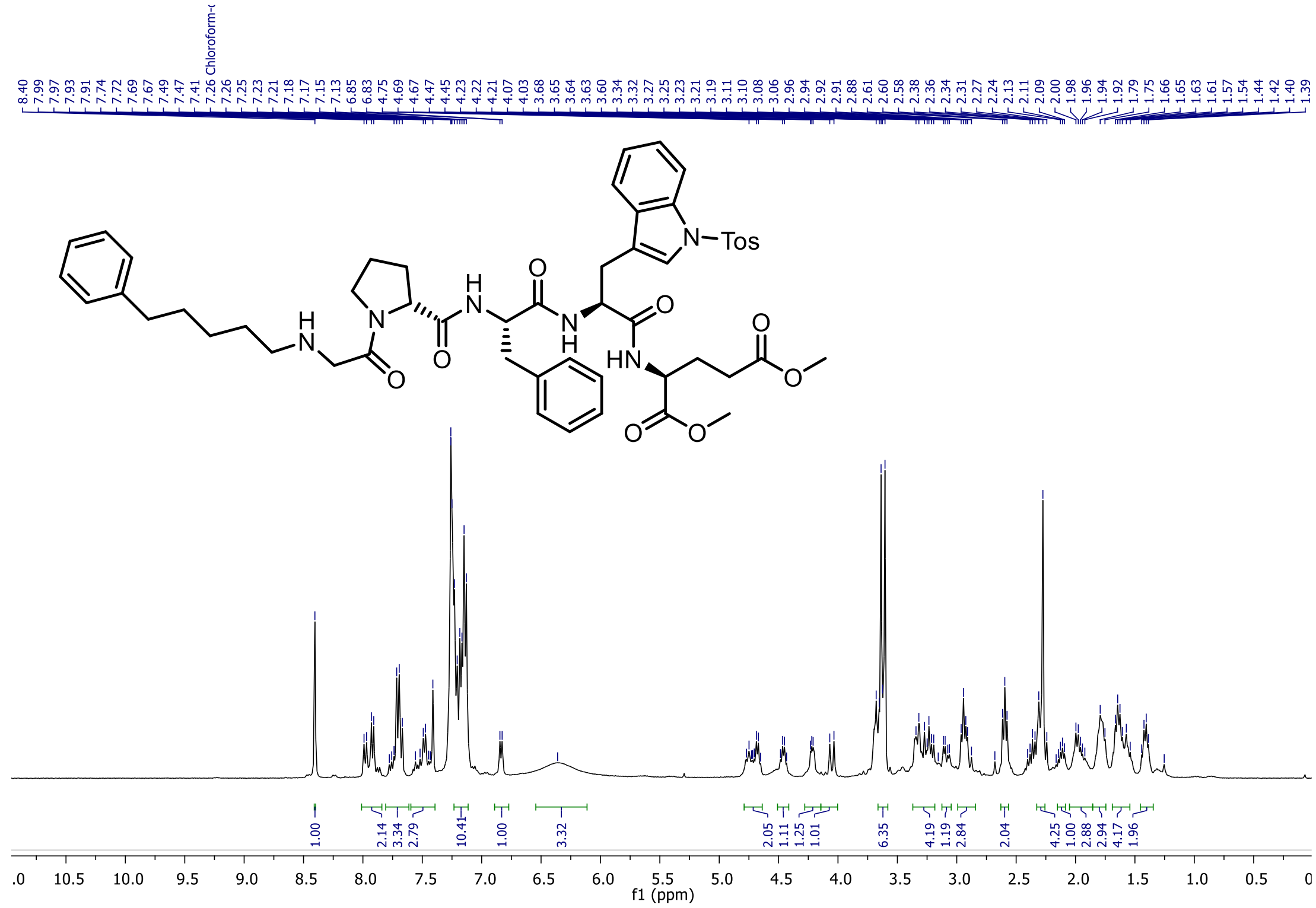

^{13}C NMR (101 MHz, $CDCl_3$)

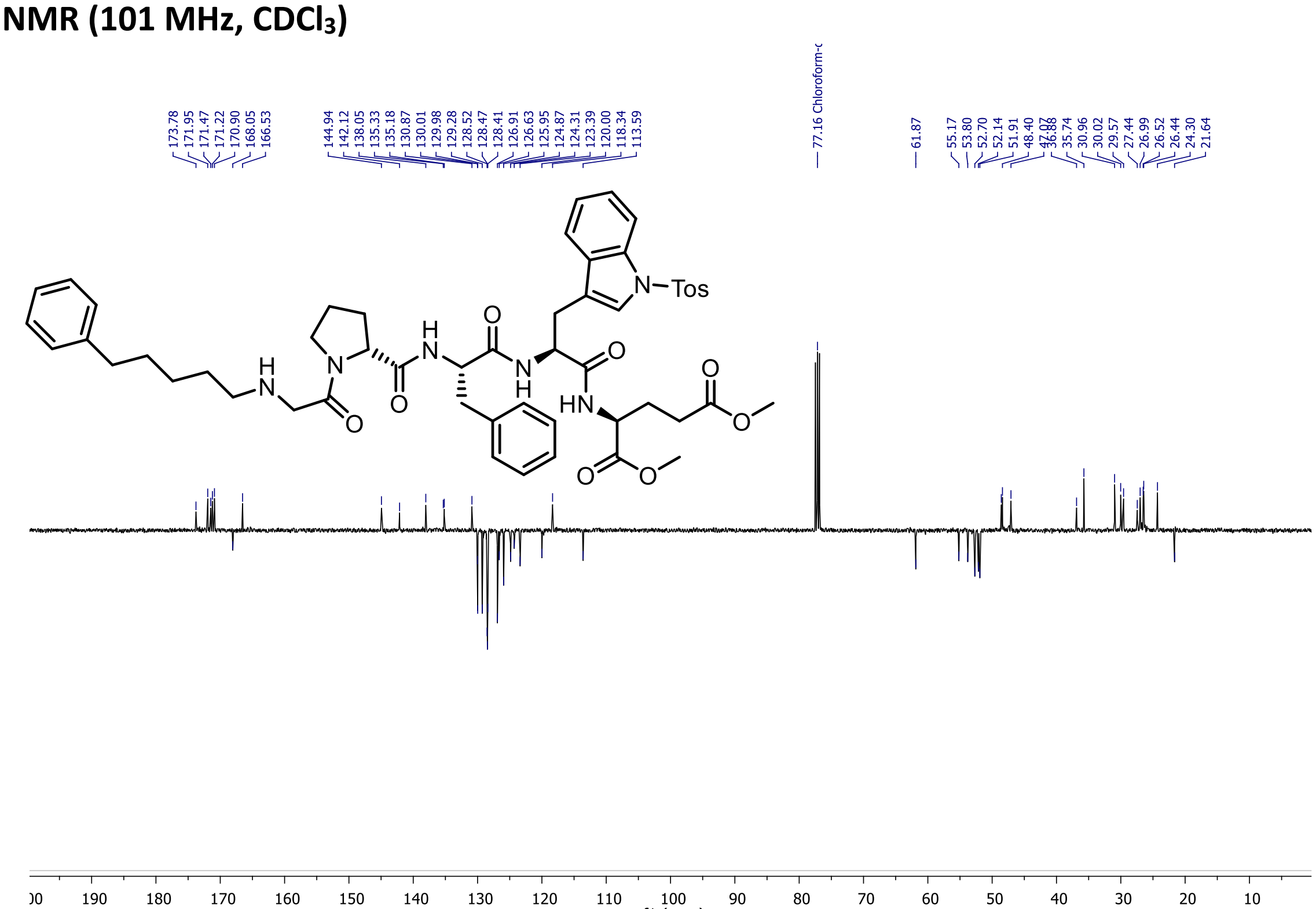

Methyl (*S*)-2-phenyl-3-((*R*)-1-((5-phenylpentyl)glycyl)pyrrolidine-3-carboxamido)propanoate (4.05)

^{1}H NMR (600 MHz, $CDCl_3$)

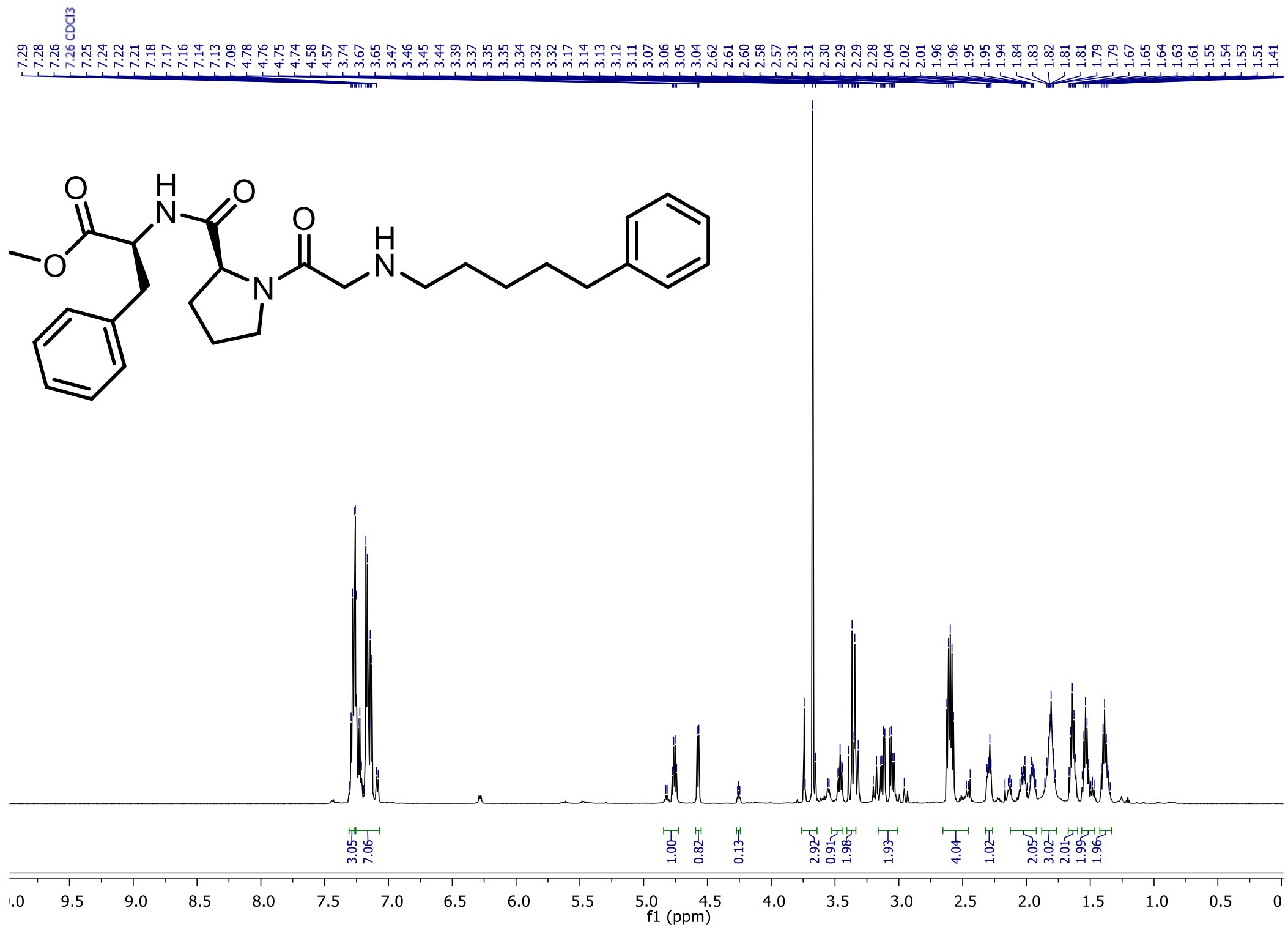

^{13}C NMR (151 MHz, $CDCl_3$)

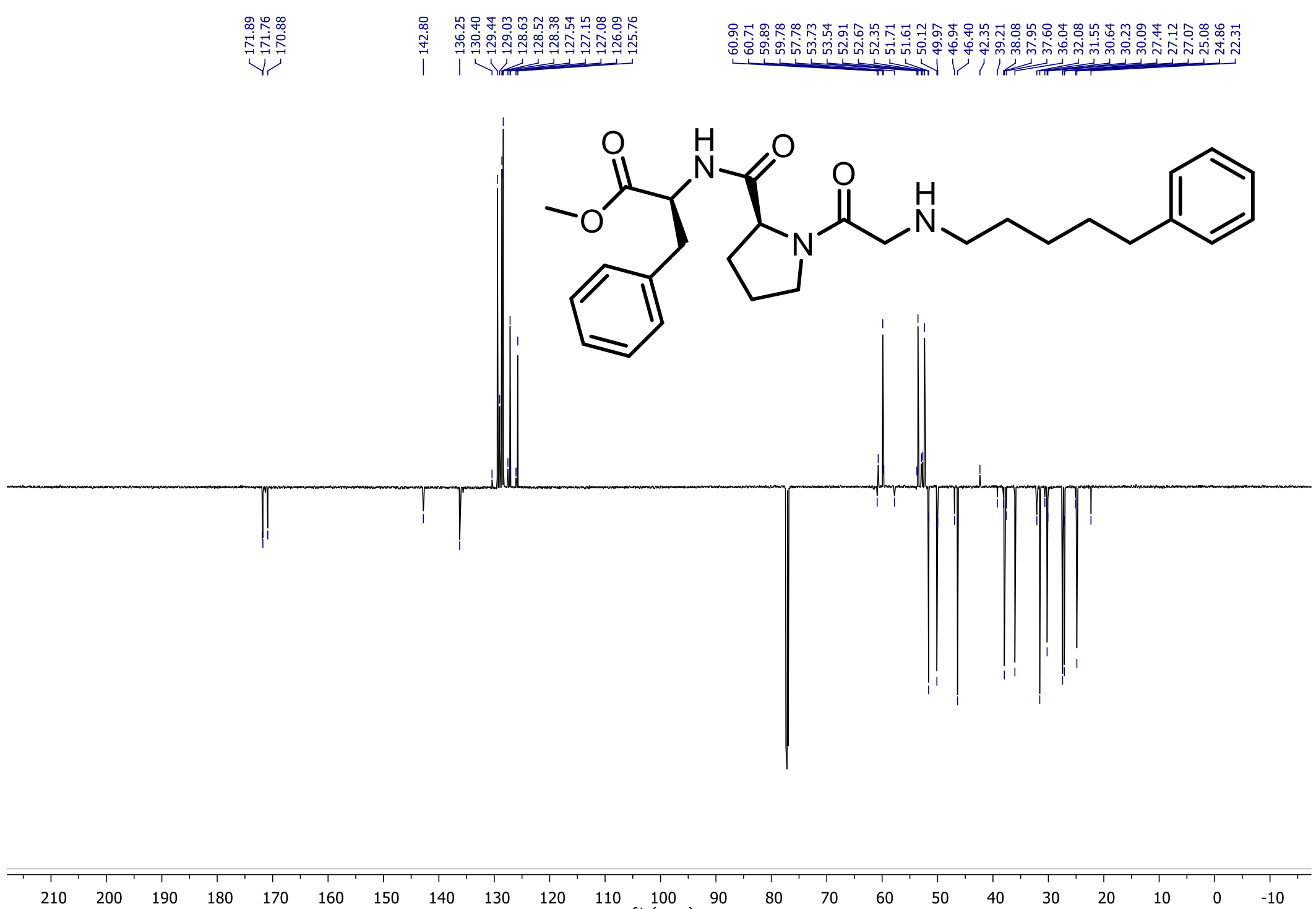

Dimethyl (5-phenylpentyl)glycylprolylphenylalanylglutamate 4.06)

^{1}H NMR (400 MHz, CDCl$_3$)

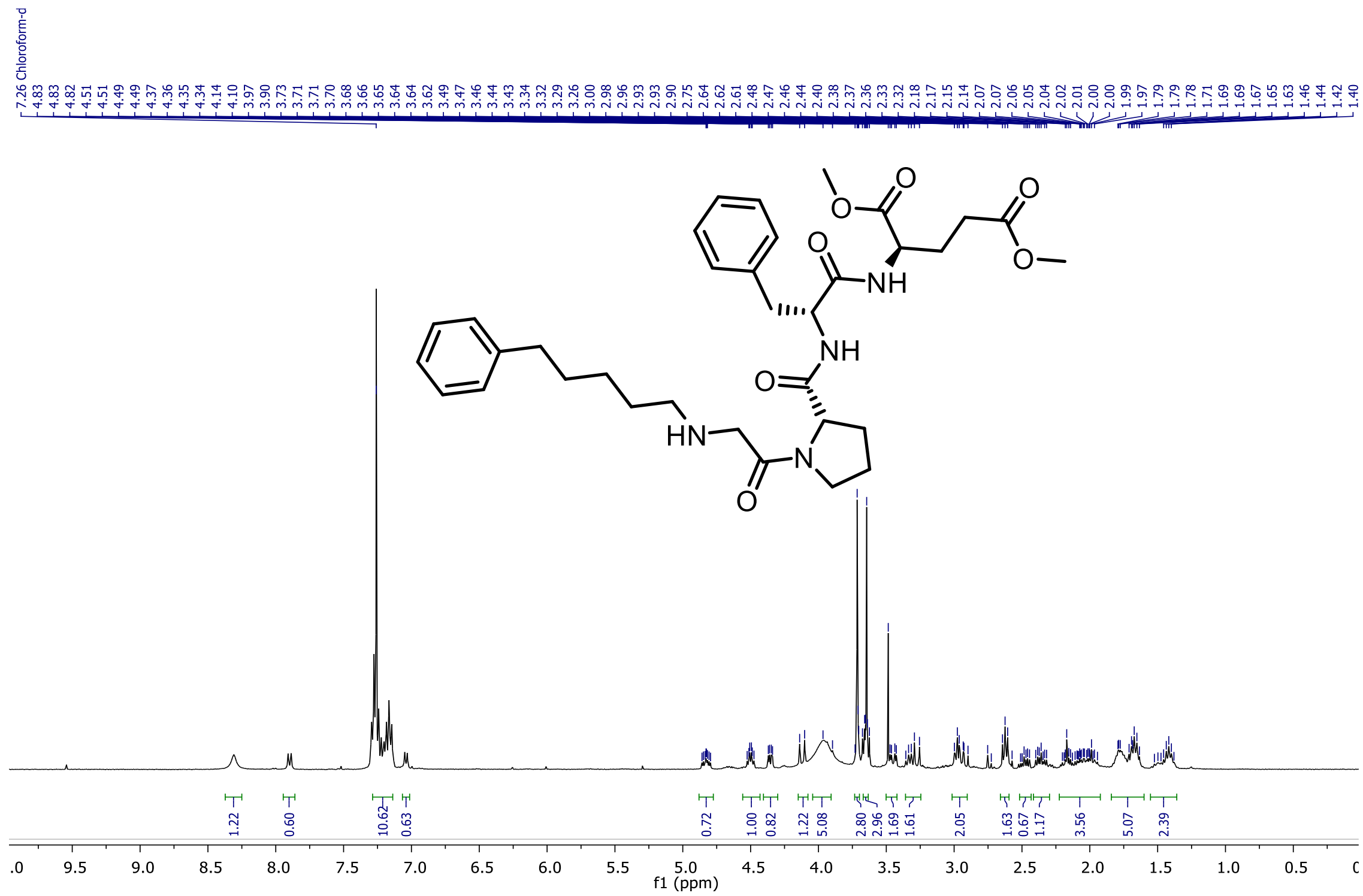

^{13}C NMR (101 MHz, CDCl$_3$)

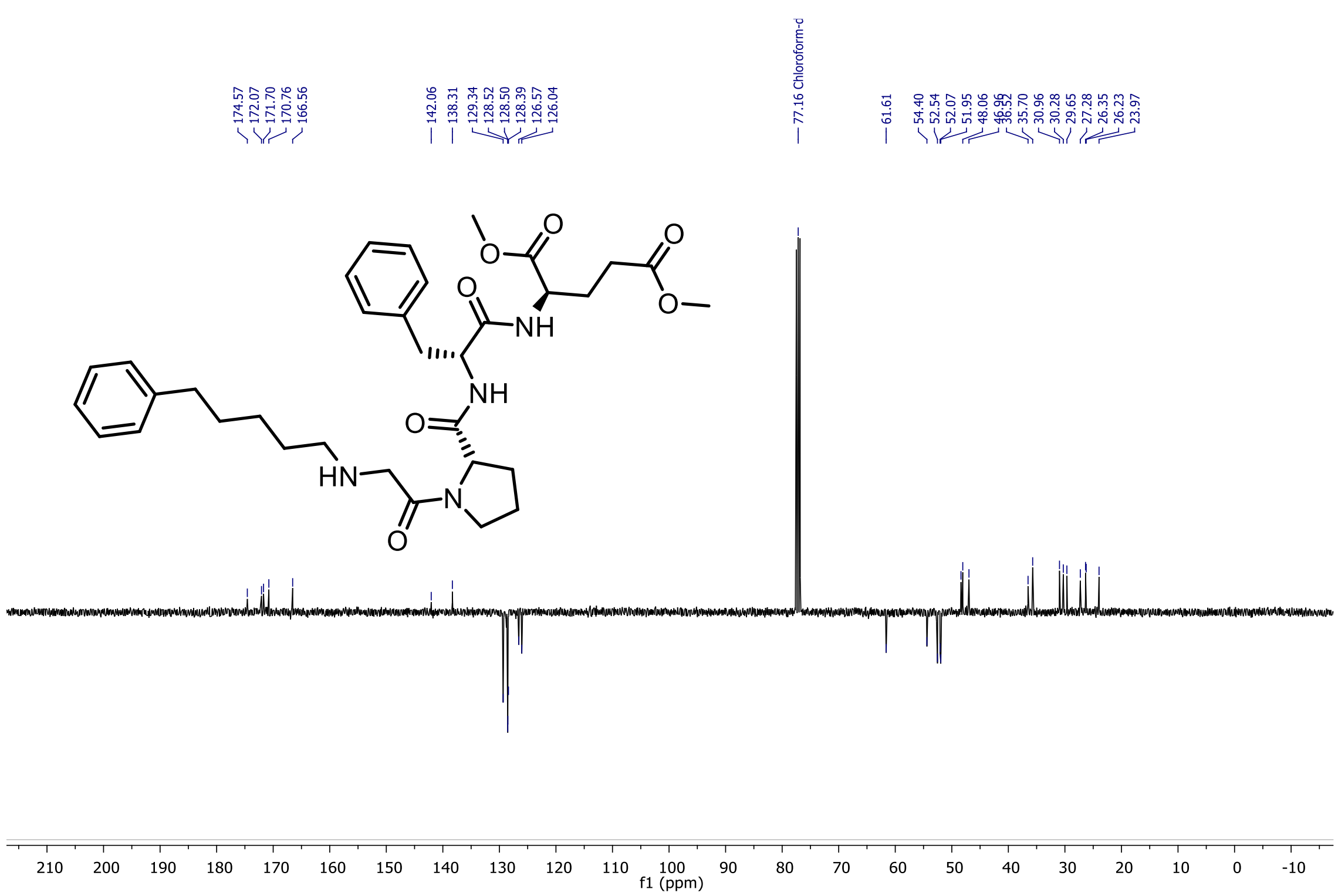

Dimethyl (10-(2-((*R*)-4-(4-chlorophenyl)-2,3,9-trimethyl-6*H*-thieno[3,2-*f*][1,2,4]triazolo[4,3-*a*][1,4]diazepin-6-yl)-*N*-methylacetamido)decyl)glycyl-*L*-prolyl-*L*-phenylalanyl-*L*-glutamate (4.07)

^{1}H NMR (600 MHz, CDCl$_3$)

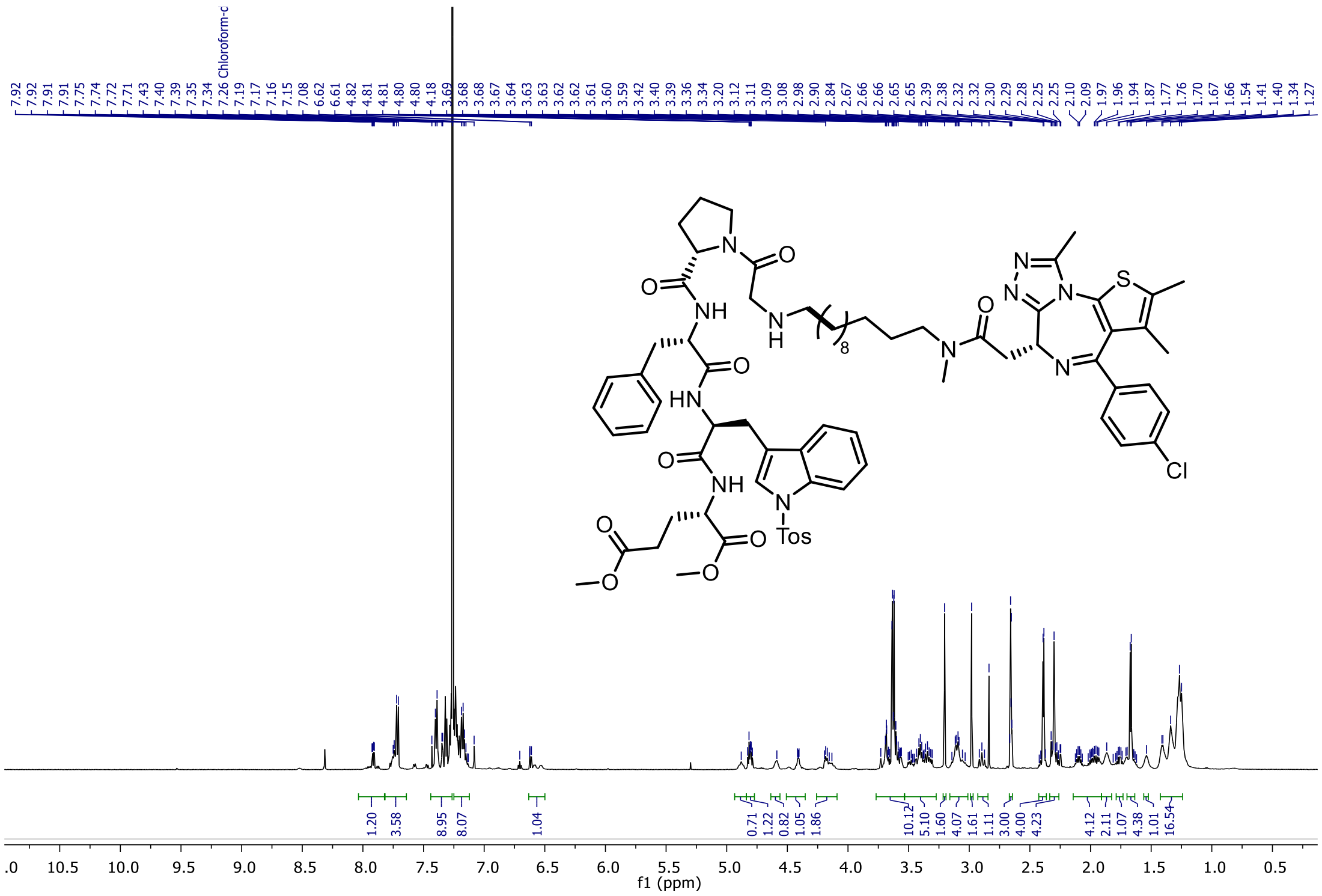

^{13}C NMR (151 MHz, CDCl$_3$)

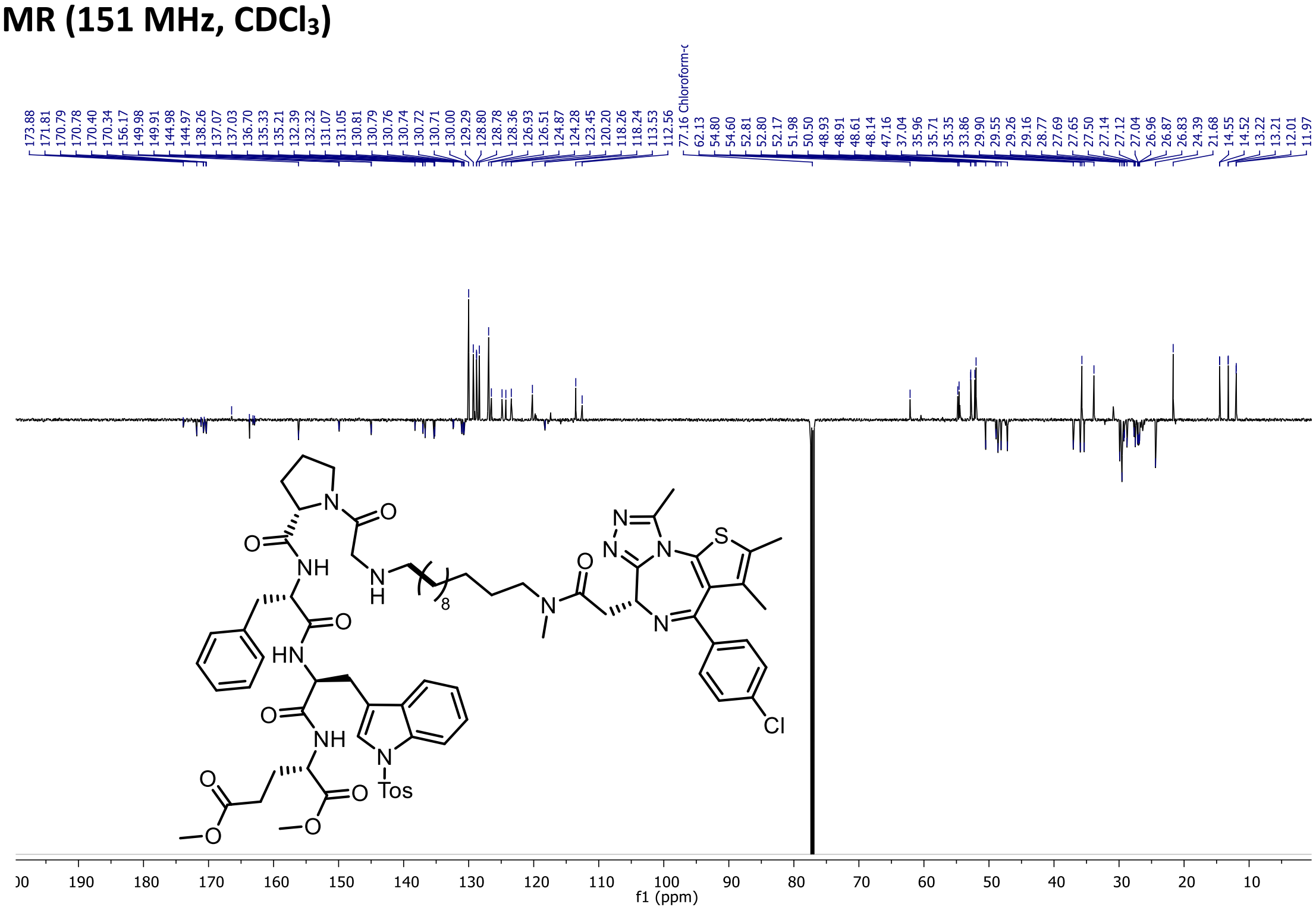

1-(2-Cyclohexylethyl)-1,3,3-trimethylurea (C1)
^{1}H NMR (400 MHz, $CDCl_3$)

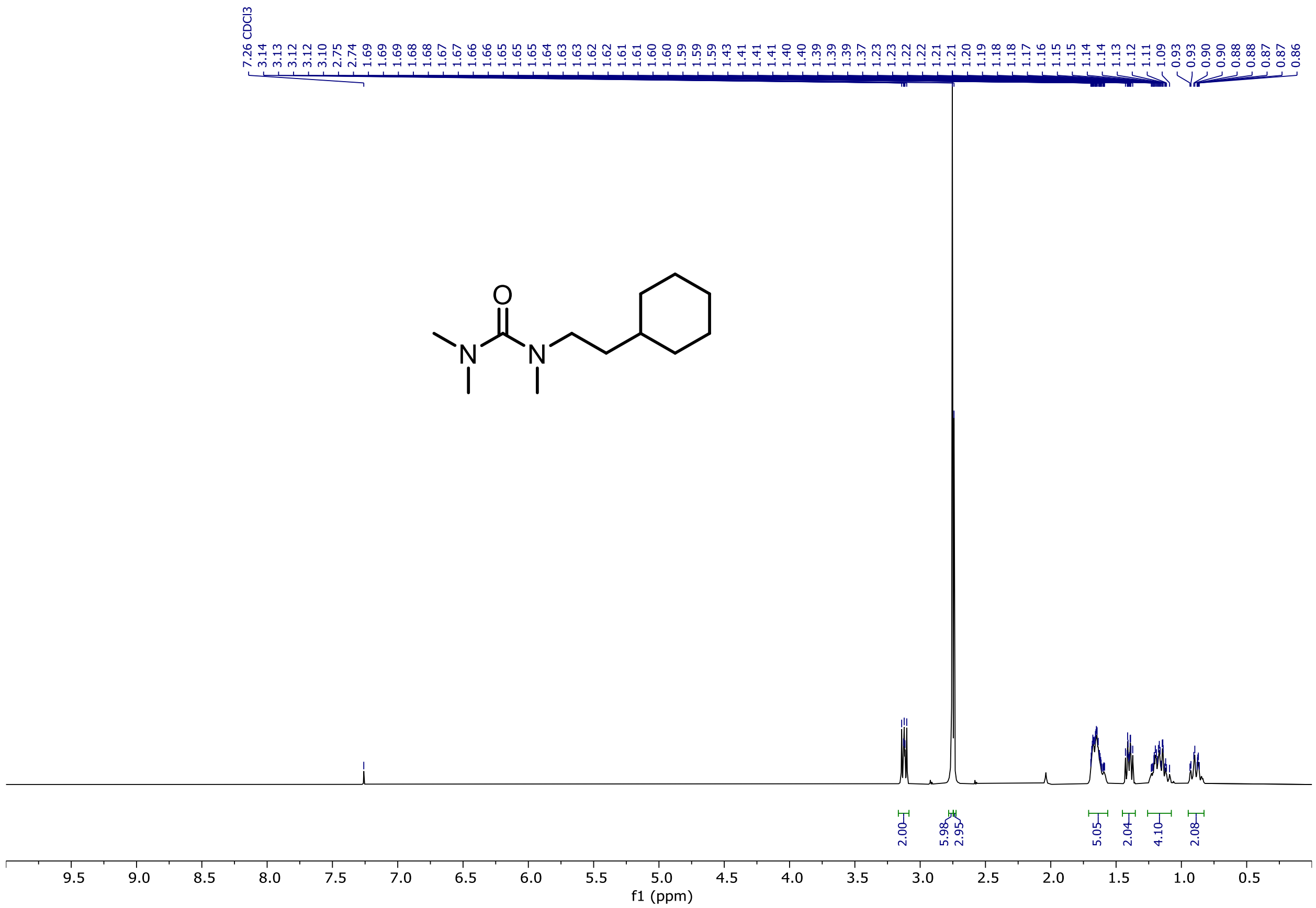

Supplement: Supplementary file 1 [file 41557_2026_2178_MOESM1_ESM.pdf]
